# Increased ONECUT2 induced by *Helicobacter pylori* promotes gastric cancer cell stemness via an AKT-related pathway

**DOI:** 10.1038/s41419-024-06885-2

**Published:** 2024-07-12

**Authors:** Mi Lin, Ru-Hong Tu, Sheng-Ze Wu, Qing Zhong, Kai Weng, Yu-Kai Wu, Guang-Tan Lin, Jia-Bin Wang, Chao-Hui Zheng, Jian-Wei Xie, Jian-Xian Lin, Qi-Yue Chen, Chang-Ming Huang, Long-Long Cao, Ping Li

**Affiliations:** 1 Department of Gastric Surgery, Fujian Medical University Union Hospital, Fuzhou, China; 2 Key Laboratory of Ministry of Education of Gastrointestinal Cancer, Fujian Medical University, Fuzhou, China; 3 Fujian Key Laboratory of Tumor Microbiology, Fujian Medical University, Fuzhou, China

**Keywords:** Gastric cancer, Cancer stem cells

## Abstract

*Helicobacter pylori* (HP) infection initiates and promotes gastric carcinogenesis. ONECUT2 shows promise for tumor diagnosis, prognosis, and treatment. This study explored ONECUT2’s role and the specific mechanism underlying HP infection-associated gastric carcinogenesis to suggest a basis for targeting ONECUT2 as a therapeutic strategy for gastric cancer (GC). Multidimensional data supported an association between ONECUT2, HP infection, and GC pathogenesis. HP infection upregulated ONECUT2 transcriptional activity via NFκB. In vitro and in vivo experiments demonstrated that ONECUT2 increased the stemness of GC cells. ONECUT2 was also shown to inhibit PPP2R4 transcription, resulting in reduced PP2A activity, which in turn increased AKT/β-catenin phosphorylation. AKT/β-catenin phosphorylation facilitates β-catenin translocation to the nucleus, initiating transcription of downstream stemness-associated genes in GC cells. HP infection upregulated the reduction of AKT and β-catenin phosphorylation triggered by ONECUT2 downregulation via ONECUT2 induction. Clinical survival analysis indicated that high ONECUT2 expression may indicate poor prognosis in GC. This study highlights a critical role played by ONECUT2 in promoting HP infection-associated GC by enhancing cell stemness through the PPP2R4/AKT/β-catenin signaling pathway. These findings suggest promising therapeutic strategies and potential targets for GC treatment.

## Introduction

Gastric cancer (GC) is one of the leading malignancy-related causes of mortality worldwide [1]. The major risk factors for GC include *Helicobacter pylori* (HP) infection, advanced age, excessive salt intake, and inadequate consumption of fruits and vegetables [2]. HP infection was the predominant environmental contributor. However, the exact mechanism through which HP initiates GC development remains unclear. Therefore, identifying effective strategies to stop the HP-mediated induction and progression of GC, deepening our understanding of GC pathogenesis, and identifying novel therapeutic and preventive targets are essential for improving GC treatment outcomes. Studies have suggested that HP infection facilitates the establishment of a chronic inflammatory microenvironment in the stomach [3–6]. Inflammatory factors released under these conditions can induce degeneration, proliferation, and abnormal differentiation of gastric epithelial cells, thereby promoting tumorigenesis [7, 8]. Long-term persistence of this chronic inflammatory microenvironment may induce a subset of cells to transform into cancer stem cells, driving tumor initiation and progression [9, 10].

According to the inflammation-atrophy-metaplasia-carcinoma theory of GC progression [11], long-term chronic HP infection may affect cancer stem cell populations in several ways, including altering the differentiation state of epithelial cells, triggering epigenetic changes such as DNA methylation, and initiating inflammatory and immune responses in the microenvironment [12–16]. Cancer stem cells exhibit exceptional self-renewal and proliferative capabilities and play critical roles in tumor initiation, maintenance, and progression. The regulatory mechanism by which inflammation affects GC stem cells is extremely complex, and the identification of key regulatory molecules may reveal promising targets for GC prevention and treatment.

We identified significant correlations between ONECUT2, HP infection, and GC. These relationships were uncovered by analyzing data collected from our research center and public databases, including single-cell RNA sequencing, spatial transcriptomics, high-throughput RNA sequencing, etc. ONECUT2, a member of the ONECUT transcription factor family, is characterized by a cut domain [17]. This transcription factor is predominantly expressed in the gallbladder, duodenum, liver, and small intestine and is minimally expressed in the brain, pancreas, stomach, and testes [17]. ONECUT2 is critical in various biological processes and diseases, including liver development, insulin secretion, spinal neuron development, and acquisition and maintenance of cancer stemness [17–23]. In recent years, research on ONECUT2 has intensified, deepening our understanding of its effects on cell fate determination and disease onset and progression. Accumulating evidence indicates that ONECUT2 expression significantly influences the development and progression of malignant tumors [24–26], suggesting that ONECUT2 may have significant potential for tumor diagnosis, prognosis, and treatment. Despite these findings, the role and specific mechanisms of action of ONECUT2 in HP infection-related GC remain unclear. We aimed to investigate the role and mechanism of ONECUT2 in developing HP infection-related GC and provide a scientific basis for the therapeutic targeting of ONECUT2 to treat this disease.

## Materials and methods

### Human samples

Human gastric carcinoma samples were collected from the Fujian Medical University Union Hospital, China. Written informed consent was obtained from all the participants. This study analyzed gastric cancer tissue from 349 patients collected from April 2013 to December 2022, including 75 cases for RT-qPCR, 92 cases for immunoblotting, 36 cases for RNA sequencing, two cases for spatial transcriptomics, nine cases for single-cell RNA sequencing, and 135 cases for tissue microarray. The inclusion criteria were as follows: (a) histologically diagnosed gastric cancer; (b) no history of the patients had received preoperative radiotherapy, chemotherapy, or other biological agents; (c) complete clinicopathologic data; and (d) more than 5 years of follow-up for the tissue microarray cases. This study’s design and reporting procedures were approved by the Institutional Review Board of Fujian Medical University Union Hospital (IRB number:2021KJT028-01).

### Mice

Mice were maintained at the Fujian Medical University animal facility in accordance with institutional guidelines. Ethical approval was obtained from the Fujian Medical University Animal Conservation and Use Committee (FJMU IACUC 2021-J-0065).

### Statistical analyses

Detailed descriptions of our materials, methods, and statistical analyses are provided in the “Supplementary Methods”. Full and uncropped western blots are presented in the Supplementary Material. The primer sequences are provided in Supplementary Table 4.

## Results

### ONECUT2 is associated with HP infection and GC

**Fig. 1:**
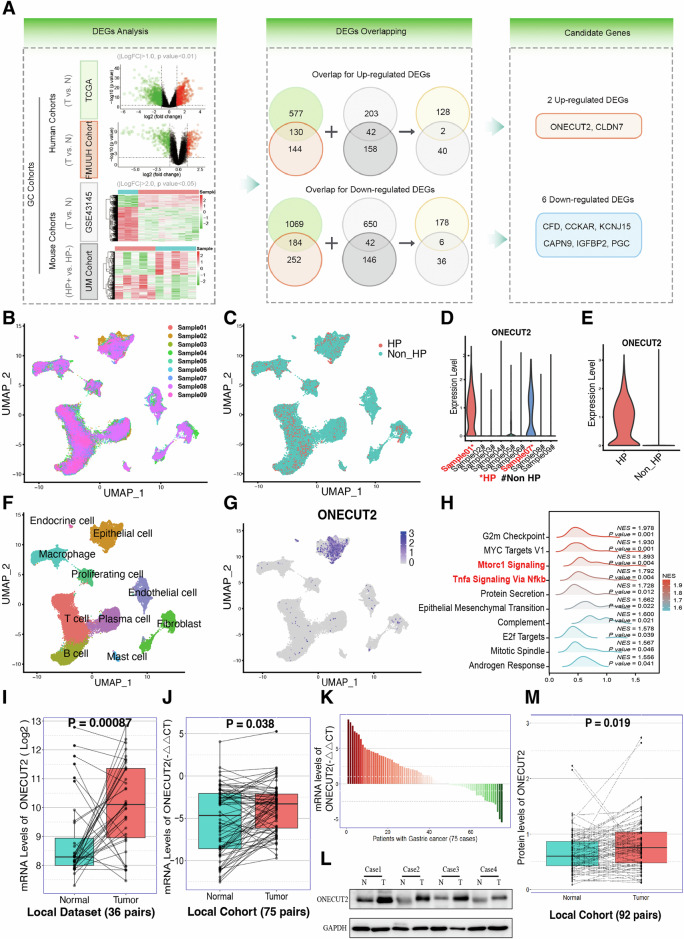
Association of ONECUT2 with *Helicobacter pylori* (HP) infection and gastric cancer (GC). **A** Volcano and heat map illustrations of overlap analysis for differential expressed genes (DEGs) filtered from human and mouse GC cohorts, respectively. The overlap of these genes identified eight candidate genes. **B** UMAP plot of nine human stomach adenocarcinoma (STAD) samples used for single-cell RNA sequencing. **C** UMAP plot displays two categories of human STAD samples: HP-infected and uninfected. **D** ONECUT2 mRNA expression in the nine human STAD samples. **E** ONECUT2 mRNA expression in human STAD specimens grouped by HP infection status. **F** UMAP plot visualization of 10 specific cell types. **G** Among these cell clusters, ONECUT2 expression is shown to be elevated in epithelial cells. **H** Genomic Set Enrichment Analysis (GSEA) shows enrichment of TNFα signaling via NFκB and mTORC1 signaling pathways in cancer cells expressing ONECUT2. **I** ONECUT2 mRNA levels show high expression in human GC tissue in our center’s sequencing data (*n* = 36). ***P < 0.001. **J**, **K** Comparison of ONECUT2 mRNA levels in human GC tissue and paired non-tumor tissue from our center further reveals high ONECUT2 transcript levels in GC tissue. (*n* = 75, P = 0.038). **L** Representative images showing ONECUT2 protein detection in GC tissue and paired non-tumor tissue by western blotting. **M** ONECUT2 protein expression was higher in human GC tissue compared with paired non-tumor tissue from our center (*n* = 92, P = 0.019).

To identify genes associated with HP infection and GC, we combined RNA-seq data from 36 GC cases and their matched para-carcinoma tissue from our research center with GC sequencing data from The Cancer Genome Atlas (TCGA) to comprise our human GC cohort. Our mouse cohort was assembled from RNA-seq data from five HP-infected and five uninfected mice, together with data from TNF KO and BALB/c conditionally mutant mouse models of inflammation-related GC (dataset GSE43145) obtained from the Gene Expression Omnibus (GEO). After overlapping, 130 upregulated and 184 downregulated DEGs in the human GC cohort and 42 upregulated and 42 downregulated differential expressed genes (DEGs) in the mouse cohort were obtained in our analysis. Untimately, we identified two commonly upregulated genes (ONECUT2 and CLDN7) and six commonly downregulated genes by overlapping human and mouse GC cohorts (Fig. 1A). Further TCGA GC database validation confirmed a significant association between ONECUT2 expression and disease-free survival (DFS) (P = 0.024). In contrast, the associations for the remaining seven genes were not statistically significant or showed an inverse trend (Supplementary Fig. 1A). Therefore, we investigated the role of ONECUT2 in HP infection and GC development.

Our review of the TCGA public database revealed a significant upregulation of ONECUT2 mRNA expression in most cancers, including gastric adenocarcinoma (STAD), compared to the corresponding non-tumor tissue (Supplementary Fig. 1B). These findings implicated ONECUT2 as a potential player in developing multiple tumor types. Further analysis using non-paired and paired GC datasets from the GEO and mouse GC datasets consistently showed increased ONECUT2 mRNA expression in cancerous tissue (Supplementary Fig. 1C–E). Furthermore, within the human HP-infected GC cell line (GSE70394) and HP-infected healthy gastric epithelial cell line (GSE74577) datasets, ONECUT2 was significantly overexpressed in HP-infected samples compared with those from healthy tissue (Supplementary Fig. 1F). These results indicate a close association between ONECUT2, HP infection, and the pathogenesis of GC.

To further clarify the mechanism by which ONECUT2 mediates HP-associated gastric carcinogenesis, we performed single-cell RNA sequencing of nine human STAD samples (two with and seven without HP infection) (Fig. 1B, C, and Supplementary Table 1). After excluding poor-quality cells, 56,693 cells were analyzed. ONECUT2 expression significantly increased in HP-infected human GC samples (Fig. 1D, E). These cell clusters were categorized into ten different cell types (Fig. 1F and Supplementary Fig. 2A, B), with ONECUT2 expression predominantly elevated in epithelial cells compared with other cell populations (Fig. 1G). We identified epithelial cell clusters and classified them as ONECUT2-positive or ONECUT2-negative based on the presence or absence of ONECUT2 expression. Genomic Set Enrichment Analysis (GSEA) revealed enrichment of the TNFα signaling pathway via the NFκB and mTORC1 signaling gene clusters within cancer cells expressing ONECUT2 (Fig. 1H and Supplementary data 2).

RNA-seq data from 36 GC cases showed high ONECUT2 mRNA levels in human GC tissue (Fig. 1I). We also obtained 75 and 92 sets of fresh GC and adjacent non-tumor tissue samples, respectively, from our research center. These samples were subjected to RT-qPCR and immunoblotting (Fig. 1J–M). Our data confirmed that ONECUT2 mRNA transcript levels were significantly elevated in a significant proportion of GC patients (65.33%; 49/75). We also observed increased ONECUT2 protein expression in GC patients (67.4%; 62/92). These results indicated a potential oncogenic role of ONECUT2 in HP-associated GC.

### HP infection upregulates ONECUT2 via NFκB

**Fig. 2:**
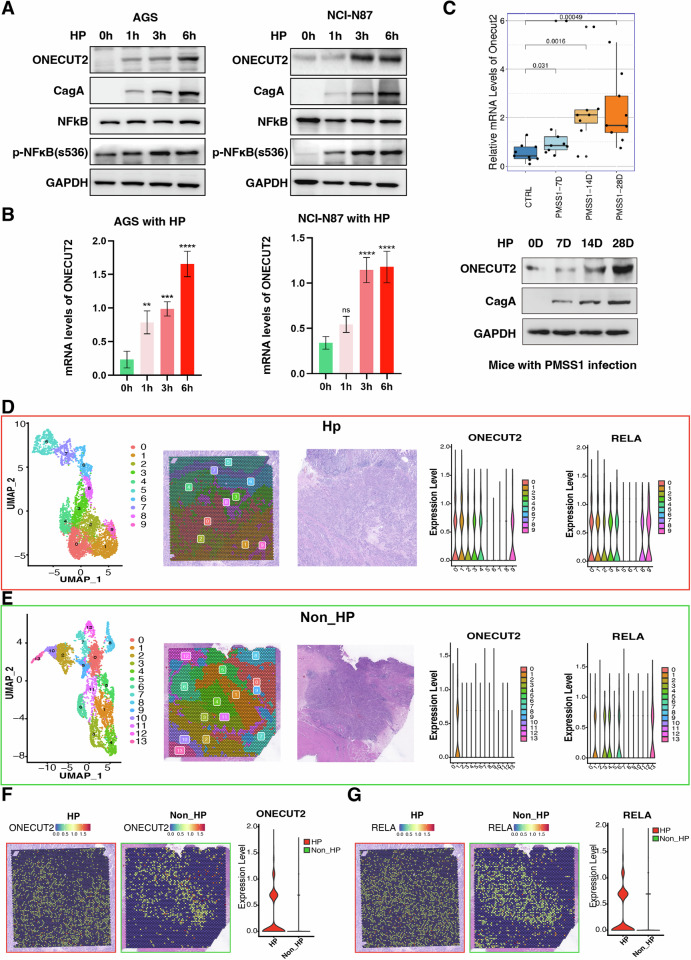
HP infection elevates ONECUT2 expression. **A** Time-dependent expression of ONECUT2, CagA, NFκB (p65), and p-NFκB (s536) proteins in AGS and NCI-N87 GC cells upon infection with carcinogenic HP (ATCC 43054) was assessed by western blotting. **B** ONECUT2 mRNA expression levels in HP-infected AGS and NCI-N87 GC cells was quantified by RT-qPCR (*n* = 3). **C** The mRNA and protein expression of ONECUT2 were measured in gastric tissue with the infection of mouse pathogenic HP (PMSS1). **D** Spatial transcriptome analysis of the Po1 section (HP-infected GC tissue) revealed ONECUT2 and RELA mRNA expression in 10 distinct clusters. Of these, clusters 0, 1, 2, 3, 4, and 9 showed increased ONECUT2 expression, correlating with an increase in RELA. **E** Spatial transcriptome analysis of the Ne1 section (non-HP-infected GC tissue) revealed ONECUT2 and RELA mRNA expression in 14 distinct clusters. Of these, cluster 1 showed increased ONECUT2 expression, which was associated with elevated RELA levels. **F**, **G** Spatial transcriptome analysis revealed that mRNA expression levels of ONECUT2 (**F**) and RELA (**G**) were higher in HP-infected GC tissue than in non-HP-infected GC tissue. ns, not significant; **P < 0.01; ***P < 0.001; ****P < 0.0001.

We investigated the association between HP infection and ONECUT2 expression in vivo and in vitro. Our experiments involved co-cultivation of a pathogenic HP strain (ATCC 43054) with AGS and NCI-N87 GC cell lines characterized by high ONECUT2 protein expression (Supplementary Fig. 3A). Data from HP-infected cells showed a time-dependent increase in the protein expression of CagA, a marker of HP infection, in parallel with an upward trend in ONECUT2 protein expression and mRNA transcription (Fig. 2A, B). Our in vivo study using HP-infected mice confirmed these findings; the duration of HP infection was positively correlated with ONECUT2 mRNA and protein expression in gastric tissue (Fig. 2C). Together, these results indicated the potential role of HP in enhancing ONECUT2 mRNA and protein expression.

To further investigate the spatial transcriptional regulation mechanism by which HP infection mediates gastric carcinogenesis via ONECUT2, we performed 10x Genomics Visium experiments on GC tissue sections from one HP-infected patient (Po1, Fig. 2D and Supplementary Table 2) and one uninfected patient (Ne1, Fig. 2E). From the Po1 and Ne1 sections, we obtained 4954 and 4854 spots, respectively, each representing 2–10 cells. As expected, ONECUT2 mRNA expression was higher in HP-infected GC tissue than in uninfected ones (Fig. 2F).

Our single-cell RNA sequencing analyses revealed enrichment of ONECUT2-positive tumor cells within clusters displaying TNFα signaling via NFκB (Fig. 1H). The spatial transcriptome results further revealed that RELA (a protein-coding gene of the NFκB subunit) expression was higher in HP-infected GC tissue than in uninfected tissue (Fig. 2G). In a time gradient assay, p-NFκB (s536) gradually increased with increasing duration of HP infection (Fig. 2A). These findings indicate NFκB’s link to ONECUT2 protein expression and its role in mediating HP infection-induced gastric carcinogenesis. To elucidate the spatial transcriptional relationship between ONECUT2 and RELA, spots from HP-infected and uninfected sections were grouped into 10 and 14 clusters using Seurat graph-based clustering analysis. Despite the challenge of identifying the exact composition of each cluster due to tumor infiltration and the potential mixing of different cells in the tumor microenvironment, clusters with higher ONECUT2 expression (clusters 0, 1, 2, 3, 4, and 9 in the HP section and cluster 1 in the uninfected section) were consistently associated with increased RELA expression in HP-infected and uninfected GC tissue (Fig. 2D, E).

**Fig. 3:**
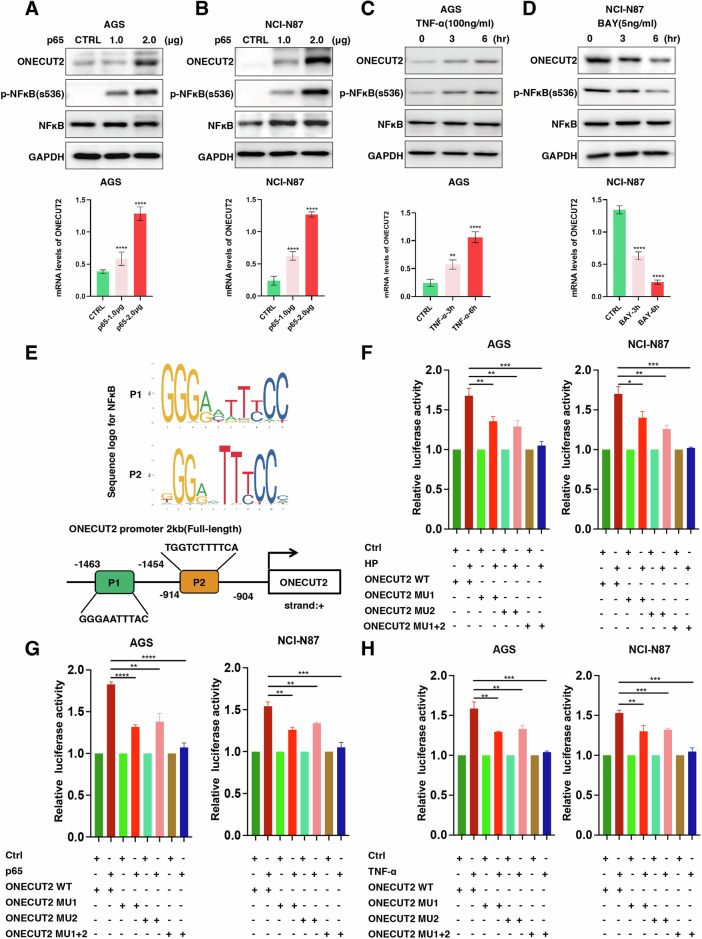
NFκB regulates ONECUT2 protein expression at the transcription level. **A**, **B** In AGS and NCI-N87 GC cells, transfection with 1 or 2 μg of p65 overexpression plasmid resulted in increased p-NFκB protein levels (s536) and increased ONECUT2 protein and mRNA (*n* = 3). **C** Protein levels of p-NFκB (s536) and ONECUT2, and ONECUT2 mRNA levels, increased over time with prolonged activation of the NFκB pathway in AGS GC cells (cells were co-cultured with 100 ng/ml TNF-α protein for 3 and 6 h to activate the NFκB pathway) (*n* = 3). **D** Protein levels of p-NFκB (s536) and ONECUT2, and ONECUT2 mRNA levels decreased over time with prolonged inhibition of the NFκB pathway in NCI-N87 GC cells (cells were treated with 5 ng/ml BAY protein to inhibit the NFκB pathway for 3 and 6 h) (*n* = 3). **E** Schematic representation of two predicted NFκB binding sites for ONECUT2 transcription according to JASPAR. **F** Dual-luciferase reporter assays showing that HP infection stimulated ONECUT2 promoter transcription in AGS and NCI-N87 cells. **G** Overexpression of NFκB in AGS and NCI-N87 cells was shown to stimulate transcriptional activity of the ONECUT2 promoter using dual-luciferase reporter assays. **H** Dual-luciferase reporter assays revealed that activation of NFκB by TNF-α could stimulate transcriptional activity from the ONECUT2 promoter in AGS and NCI-N87 cells. **P < 0.01; ****P < 0.0001.

Our previous research suggests that HP may modulate downstream targets via NFκB signaling [27]. We thus induced NFκB (p65) overexpression in GC cells or used an NFκB pathway activator, specifically 100 ng/ml TNF-α protein, and observed increased levels of phosphorylated NFκB (p-NFκB at s536), ONECUT2 mRNA, and ONECUT2 protein. These increases correlated with the elevated intensity of p65-overexpressing plasmid transfection and an increased duration of NFκB pathway activation (Fig. 3A–C). In contrast, a marked decrease in ONECUT2 mRNA and protein was observed upon application of 5 ng/ml of BAY protein (an NFκB pathway inhibitor) (Fig. 3D). These data confirm that HP infection can modulate ONECUT2 protein expression via the NFκB pathway.

NFκB is a transcription factor that primarily regulates downstream targets by modulating transcription. We used the JASPAR (http://jaspar.genereg.net/) website to predict two major NFκB binding sites within the ONECUT2 gene regulatory region (Fig. 3E) and constructed luciferase reporter plasmids using these sequences (Supplementary Fig. 3B–E). After evaluating NFκB (p65) in the GC cell lines AGS and NCI-N87 by overexpression, TNF-α-mediated activation, and induction of NFκB upregulation by HP infection, we performed dual-luciferase reporter assays. In GC cells transfected with ONECUT2 wild-type luciferase reporter plasmids, NFκB overexpression and NFκB activation via TNF-α or HP infection activated transcription from the ONECUT2 promoter **(**Fig. 3F–H**)**. However, in GC cells transfected with ONECUT2 mutation (MU1, MU2 and MU1+2) luciferase reporter plasmids, overexpression of NFκB, activation of NFκB by TNF-α, or HP infection significantly decreased ONECUT2 promoter transcriptional activity (Fig. 3F–H). Together, these results demonstrate that HP infection upregulates ONECUT2 protein expression through NFκB, activating ONECUT2 gene transcription.

### ONECUT2 promotes increased stemness in GC cells

**Fig. 4:**
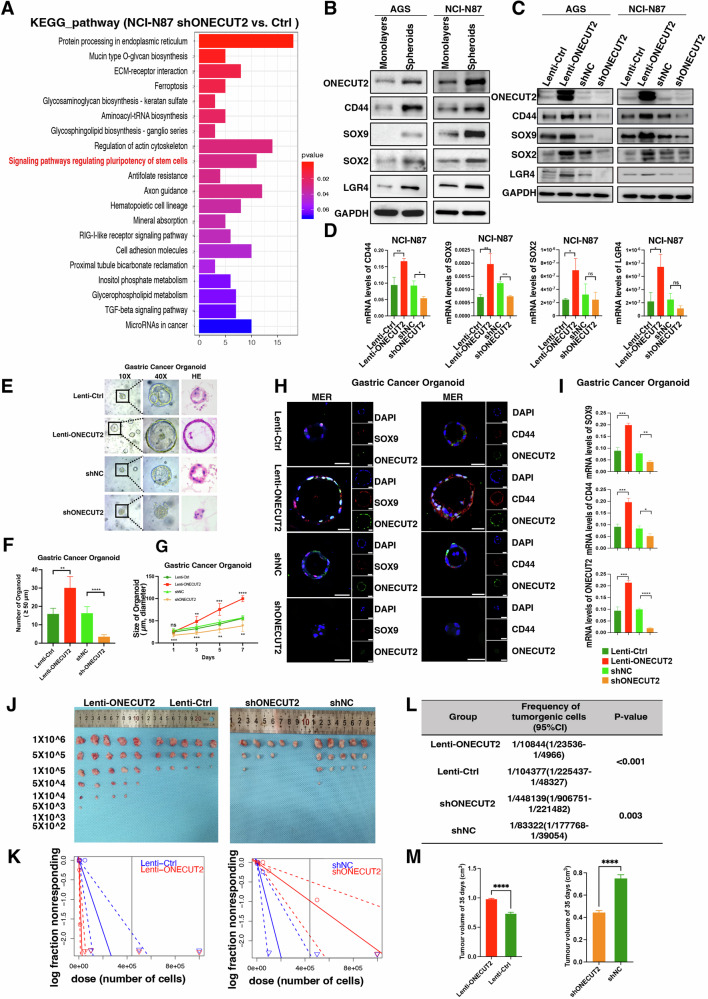
ONECUT2 enhances GC cell stemness. **A** KEGG pathway enrichment analysis of RNA-seq data indicated that ONECUT2 is associated with signaling pathways regulating stem cell pluripotency. **B** Expression levels of ONECUT2 and associated stemness marker proteins (CD44, SOX9, SOX2, LGR4) in 3D sphere-forming GC cells increased compared with those in monolayer (2D) GC cells. **C** Expression levels of the associated stemness marker proteins either increased or decreased in direct correlation with overexpression or downregulation of ONECUT2. **D** mRNA levels of the four stemness markers increased or decreased in correlation with ONECUT2 overexpression or downregulation in NCI-N87 cells (*n* = 3). **E** Bright-field images and hematoxylin and eosin (H&E) staining reveal growth variations in human GC organoids infected with lentivirus carrying overexpressed or downregulated ONECUT2. **F** The number of human GC organoids increased significantly after infection with lentivirus overexpressing ONECUT2, whereas organoids infected with lentivirus downregulating ONECUT2 were largely absent. **G** The diameters of human GC organoids infected with lentivirus overexpressing ONECUT2 increased, whereas organoids infected with lentivirus downregulating ONECUT2 typically measured less than 50 µm in diameter. **H**, **I** Immunofluorescence staining and mRNA expression of SOX9, CD44, and ONECUT2 in human GC organoids with indicated conditions. **J** An extreme limiting dilution assay revealed differences in subcutaneous tumor formation in mice injected with NCI-N87 cells overexpressing or downregulating ONECUT2 expression at different dilution levels. **K**, **L** A limiting dilution approach was used to evaluate the statistical difference in tumorigenic frequency between all groups. **M** Tumor volumes in mice injected with the highest number of cells (1 × 10^^6^) showed that the group overexpressing ONECUT2 had significantly higher tumor volumes than the control group. In contrast, the group with downregulated ONECUT2 expression showed significantly lower tumor volumes (*n* = 5). Scale: 25 μm. ns, not significant; *P < 0.05; ** P < 0.01; *** P < 0.001; **** P < 0.0001.

To investigate ONECUT2 oncogenic mechanisms, we analyzed RNA-seq sequencing data **(**Supplementary data 1**)** from a GC cell line (NCI-N87) with ONECUT2 gene silencing and a negative control using the Kyoto Encyclopedia of Genes and Genomes (KEGG) analysis. ONECUT2 was associated with signaling pathways that regulate cell stemness (Fig. 4A). In vitro experiments showed that compared with 2D GC cell culture, the expression of ONECUT2 protein and stemness-related markers CD44, SOX9, SOX2, and LGR4 simultaneously increased in 3D tumor stem cell spheroid culture (Fig. 4B). Overexpression or downregulation of ONECUT2 increased or decreased the expression of stemness markers, respectively (Fig. 4C), as confirmed at the mRNA level (Fig. 4D). In single-cell RNA sequencing data from nine patients with GC, these stemness markers were also highly expressed in epithelial cells (Supplementary Fig. 4A–D). Further analyses of spatial transcriptomic data revealed an increasing trend in these four stemness markers in HP-infected GC tissue in the Po1 and Ne1 sections (Supplementary Fig. 5A–D), and the Mki67 mRNA level was also elevated in HP-infected GC tissue (Supplementary Fig. 4G).

Human GC organoids were infected with lentiviruses designed to overexpress or downregulate ONECUT2 expression. ONECUT2 overexpression resulted in greater numbers of human GC organoids with increased diameters, whereas organoids infected with lentiviruses that downregulated ONECUT2 exhibited restricted growth (Fig. 4E–G). Immunofluorescence staining and the results of mRNA expression suggested increased expression of the stemness markers CD44 and SOX9 with ONECUT2 overexpression and decreased expression when ONECUT2 was downregulated (Fig. 4H, I). Evidence from both colony formation and cell proliferation assays supported the hypothesis that ONECUT2 stimulates AGS cell proliferation (Supplementary Figs. 6A–C). Our invasion and migration experiments consistently revealed the role of ONECUT2 in enhancing the invasive and migratory potentials of AGS cells (Supplementary Fig. 7A). Consistent with these findings, 3D spheroid experiments showed an increase in the size and number of spheroids, along with increased CD44 and SOX9 expression, when ONECUT2 was overexpressed. Conversely, the downregulation of ONECUT2 resulted in the absence of spheroids and a marked reduction in CD44 and SOX9 expression (Supplementary Figs. 6D–I).

During in vivo experiments, we identified a role for ONECUT2 in promoting the development of subcutaneous tumors in mouse models (Supplementary Fig. 6J). Using extreme limiting dilution analysis, we observed a markedly increased tumorigenic frequency in the ONECUT2-overexpressing cohort compared with the control cohort (1/10,844 vs. 1/104,377, P < 0.001). In contrast, the downregulation of ONECUT2 significantly reduced the tumorigenic frequency compared with the control group (1/448,139 vs. 1/83,322, P = 0.003) (Fig. 4J–L). Following the growth curves of tumor volumes within the highest cell count groups (*n* = 5), we found that ONECUT2 overexpression correlated with a significant increase in tumor volume compared with the control group. In contrast, its downregulation resulted in a significant decrease (Fig. 4M). Our mouse lung metastasis model further demonstrated that ONECUT2 facilitated lung metastasis (Supplementary Figs. 7B–C). Collectively, these findings highlight ONECUT2’s ability to enhance the stemness of GC cells.

### ONECUT2 promotes increased stemness in GC cells via PPP2R4/AKT/β-catenin

**Fig. 5:**
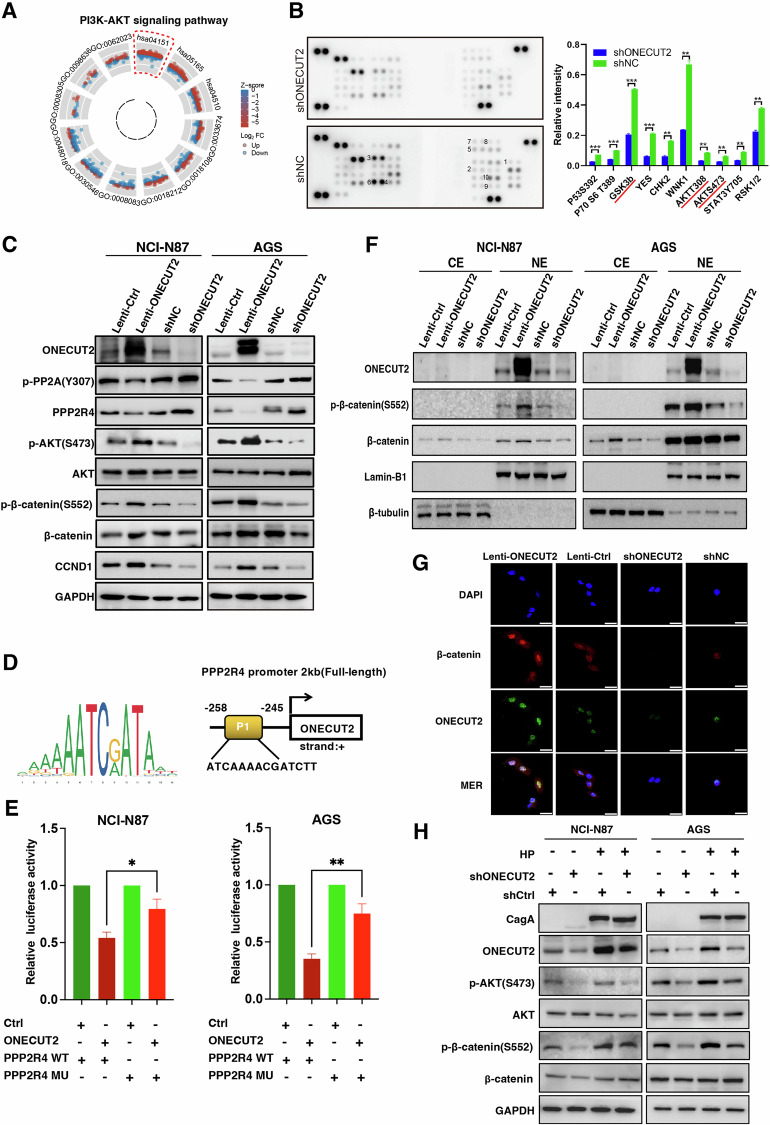
HP infection induces elevated ONECUT2 expression, promoting GC cell stemness via the PPP2R4/AKT /β-catenin axis. **A** A significant correlation between ONECUT2 and the PI3K-AKT pathway was identified in the TCGA public database by combined GO-KEGG and FC enrichment analyses. **B** Phosphokinase array results showed that NCI-N87 cells with downregulated ONECUT2 expression exhibited multiple downregulated phosphokinases, including GSK3β, AKT (T308), and AKT (S473), compared with that in the control group. **C** AKT pathway related proteins were detected by western blotting in GC cells overexpressing or downregulating ONECUT2. **D** Schematic representation of potential PPP2R4 transcriptional binding sites for ONECUT2 as predicted by JASPAR. **E** Dual-luciferase reporter assays showed that ONECUT2 could inhibit PPP2R4 transcription. **F** Nuclear-cytoplasmic fractionation experiments revealed that ONECUT2 overexpression in GC cells promoted β-catenin phosphorylation at S552, allowing p-β-catenin(S552) to accumulate in the nucleus. In contrast, the downregulation of ONECUT2 expression resulted in a decrease in nuclear p-β-catenin(S552). **G** Nucleocytoplasmic translocation experiments showed that ONECUT2 overexpression in AGS cells led to the translocation of β-catenin from the cytoplasm into the nucleus. Conversely, the downregulation of ONECUT2 expression in AGS cells decreased nuclear β-catenin. **H** HP infection could upregulate the reduction of AKT(S473) and β-catenin(S552) phosphorylation triggered by ONECUT2 downregulation via induction of ONECUT2. Scale: 25μm. **P < 0.01; ***P < 0.001; ****P < 0.0001.

To clarify the mechanism by which ONECUT2 enhances the stemness of GC cells, we performed RNA-seq analysis. Our analysis revealed a significant association between ONECUT2, PI3K/AKT, and other cancer-related pathways (Supplementary Fig. 8A). Single-cell RNA-seq analysis revealed the enrichment of mTORC1 signaling gene clusters in tumor cells expressing ONECUT2 (Fig. 1H), and AKT1 expression was observed in epithelial cells (Supplementary Fig. 4E). Therefore, AKT has been investigated as a potential therapeutic target. Using the TCGA public database, the Gene Ontology and Kyoto Encyclopedia of Genes and Genomes (GO-KEGG), and FC enrichment analyses revealed a significant correlation between ONECUT2 and the PI3K-AKT pathway (Fig. 5A and Supplementary Fig. 8B–D). Phosphatase microarray results showed that ONECUT2 downregulation in GC cells resulted in concomitant downregulation of several phosphatases, including GSK3β and AKT at both T308 and S473, compared with that in the control group (Fig. 5B). These results suggested a possible link between ONECUT2-induced stemness enhancement in GC cells and AKT signaling.

Analysis of proteins associated with the AKT pathway in GC cells overexpressing or downregulating ONECUT2 found that ONECUT2 can inhibit PP2A phosphatase activity and increase AKT(S473)/β-catenin(S552) phosphorylation, resulting in detectable changes in genes whose transcription is upregulated downstream of β-catenin, including CCND1 and the stemness-related factor LGR4 (Figs. 5C, 4C). Complementary single-cell RNA sequencing and spatial transcriptome analyses revealed increased CCND1 mRNA expression in epithelial cells and HP-infected GC tissue (Supplementary Fig. 4F, H). Notably, ONECUT2 possesses potential binding sites for the PPP2R4 subunit of PP2A, as indicated in the JASPAR database (Fig. 5D). A subsequent dual-luciferase reporter assay validated these sites’ functionality, showing that ONECUT2 can repress PPP2R4 transcription (Fig. 5E and Supplementary Fig. 3F–G), in turn decreasing PP2A activity and increasing AKT(S473)/β-catenin(S552) phosphorylation.

β-catenin becomes transcriptionally active by translocation to the nucleus. Data obtained from nuclear-cytoplasmic fractionation experiments showed that in NCI-N87 and AGS GC cell lines, ONECUT2 overexpression promotes β-catenin phosphorylation at S552, leading to nuclear accumulation of β-catenin. In contrast, when ONECUT2 is downregulated, β-catenin phosphorylation at S552 is inhibited, decreasing nuclear S552-phosphorylated β-catenin levels (Fig. 5F). Nuclear translocation experiments indicate that upon ONECUT2 overexpression, the cytoplasmic-to-nuclear shuttling of β-catenin is enhanced, leading to its nuclear localization. This process is attenuated in the nuclei of catenin cells in which ONECUT2 expression is suppressed, resulting in decreased levels of β-catenin (Fig. 5G). These results demonstrate that ONECUT2 can downregulate PPP2R4 transcription, thereby decreasing PP2A activity, leading to increased AKT(S473)/β-catenin(S552) phosphorylation, which promotes translocation of β-catenin to the nucleus to initiate transcription of downstream genes associated with stemness.

To determine whether this pathway is influenced by HP infection, we used HP to infect GC cells in which ONECUT2 expression was downregulated. Intriguingly, our results indicated that HP infection upregulated the reduction of AKT(S473) and β-catenin(S552) phosphorylation triggered by ONECUT2 downregulation via ONECUT2 induction (Fig. 5H). These observations provide strong evidence that the increase in ONECUT2 induced by HP infection enhances GC cell stemness through the PPP2R4/AKT/β-catenin signaling pathway, thereby promoting GC initiation and progression.

### ONECUT2 is an independent prognostic factor in GC

**Fig. 6:**
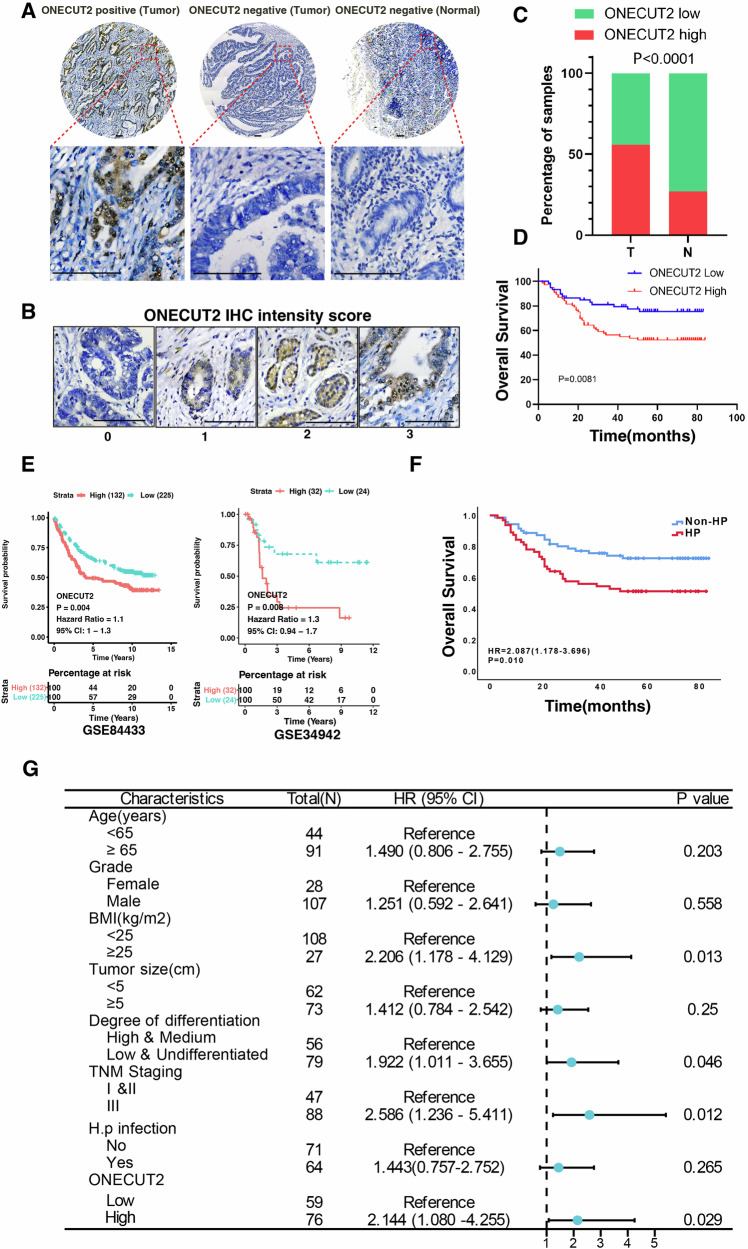
ONECUT2 correlation with GC patient prognoses. **A** Representative image of ONECUT2 immunohistochemistry (IHC) staining in human GC tissue microarrays at our center. **B** Standard scoring for ONECUT2 IHC staining intensity. **C** Distribution of ONECUT2 expression levels in GC and adjacent non-tumor tissue as assessed by IHC. **D** Association between high and low ONECUT2 levels and overall survival of 135 GC patients from our center (P = 0.008). **E** Comparison of overall survival rates of GC patients grouped by high or low expression of ONECUT2 mRNA, using public data (P = 0.004). **F** A K-M survival curve shows that patients with HP infection have a lower overall survival rate (HR = 2.087, P = 0.010) compared with those without HP infection. **G** Multivariable Cox regression analysis for overall survival in tissue microarray cohort. Scale: 100 μm.

To elucidate the relationship between ONECUT2 and GC prognosis, we performed immunohistochemical staining of paraffin-embedded tissue microarrays from 135 patients with GC at our center and examined the association between ONECUT2 staining intensity and survival rates. General clinicopathological characteristics of the patients are presented in Supplementary Table 3. High ONECUT2 expression was significantly associated with HP infection (Cramer’s V-test =0.418, P < 0.001). Figure 6A–B show representative images of ONECUT2 staining with different intensities, including negative controls. Our results showed that ONECUT2 was overexpressed in 56.30% of GC tissue, with expression levels in tumor tissue exceeding those in adjacent non-tumor tissue (Fig. 6C). Survival analyses suggested that patients with high ONECUT2 expression in GC tissue had lower overall survival rates than those with low-expression levels. The 5-year survival rate was significantly higher in the low-expression group (75.4%) than in the high-expression group (52.4%) (P < 0.05) (Fig. 6D). Consistent with our findings, analyses of the public GEO databases (GSE84433 and GSE34942) showed a significantly lower overall survival rate in patients with high ONECUT2 expression than in those with low expression (P < 0.05) (Fig. 6E). In addition, the 5-year survival rate was significantly higher in the non-HP infection group (72.5%) than in the HP infection group (51.3%) (P < 0.05) (Fig. 6F). Univariate and multivariate Cox regression analyses indicated that ONECUT2 served as an independent prognostic factor for 5-year overall survival in GC (HR = 2.144, 95% CI:1.080–4.255, P = 0.029) (Fig. 6G). In summary, our results demonstrated that high ONECUT2 expression may indicate poor prognosis in GC. Given the role of ONECUT2 in the pathogenesis of HP infection-related GC, we propose ONECUT2 as a potential therapeutic target for this GC subtype.

## Discussion

GC is a complex disease involving multiple genetic and environmental factors. Although the role of HP infection in the development and progression of GC is an area of ongoing investigation, the precise mechanism through which HP infection is associated with GC remains largely unknown. It is believed that this disease develops and progresses through multiple pathways. In this study, we identified a strong correlation among ONECUT2 transcription factors, HP infection, gastric tumorigenesis, and GC prognosis by analyzing single-cell RNA sequencing, spatial transcriptomics, high-throughput data from multiple sources, and fresh GC tissue samples. Our results suggest that HP infection triggers gastric tumorigenesis by upregulating the expression of ONECUT2.

The role of ONECUT2, a multifunctional transcription factor, in tumor pathogenesis is currently under investigation. Previous studies have suggested that ONECUT2 modulates tumor cell proliferation, migration, and differentiation by orchestrating key transcription factors and signaling pathways. In prostate cancer, ectopic ONECUT2 expression, in conjunction with hypoxia, inhibits androgen signaling and induces neuroendocrine plasticity, thereby promoting prostate neuroendocrine cell proliferation [28, 29]. ONECUT2 has been implicated in the regulation of epithelial-mesenchymal transition (in colorectal cancer) by controlling the expression of E-cadherin and vimentin, thereby enhancing colorectal cancer cell invasiveness and metastatic potential [30, 31]. Following chemotherapy for breast cancer, ONECUT2 induces tumor stemness and triggers the expression of stem cell-related genes, including SOX9, SOX2, and NOTCH1, thereby contributing to chemoresistance [25]. In lung cancer, ONECUT2 promotes stem cell properties and affects the progression of RAS-driven tumors [32, 33]. Furthermore, ONECUT2 promotes GC progression via epigenetic modifications [34, 35]. Despite these findings, the precise relationship between ONECUT2 and GC, as well as its association with HP infection, remains unclear. Therefore, a deeper exploration of the specific roles and mechanisms of ONECUT2 in HP infection-related GC may pave the way for the development of innovative therapeutic and preventive strategies.

In this study, we established an experimental paradigm using GC cells infected with carcinogenic HP strains, as well as a mouse model of HP infection. Using these models, we showed both in vitro and in vivo that HP infection upregulated ONECUT2 expression. This upregulation is accompanied by increased phosphorylation of the inflammatory marker NFκB, which is recognized as a key indicator of HP infection [36, 37]. This finding was further supported by single-cell RNA sequencing and GC spatial transcriptomics data from both HP–infected and uninfected GC patients. Previous studies suggested that HP infection is a major contributor to GC development, either directly or indirectly through inflammation [6, 38]. The inflammatory response induced by HP infection often involves the transcription factor NFκB, an observation consistent with our preliminary studies illustrating the ability of HP to manipulate downstream target pathways via NFκB. NFκB plays a central role in several cellular processes, including immunity, inflammation, and cell apoptosis [39, 40]. Upon activation, NFκB translocates to the nucleus to stimulate the transcription and expression of a number of genes. Thus, we postulated that increased ONECUT2 protein expression may be facilitated by transcriptional regulation mediated by HP infection. Our experimental data, which include changes in ONECUT2 expression levels following NFκB (p65) overexpression, activation, or inhibition, as well as results from the dual-luciferase reporter assay, collectively support our hypothesis. To date, the influence of HP infection on ONECUT2 expression via alternative pathways has not been investigated.

Tumor progression is closely linked to cancer stem cell activity, which can stimulate tumorigenesis through the reorganization of their neighboring niches [41, 42]. Several studies have highlighted the role of ONECUT2 in regulating stemness in various malignant tumors and, in turn, influencing their progression [25, 43]. Similarly, in the context of GC, our results indicated that ONECUT2 promotes GC cell stemness, a process linked to AKT signaling. As Li summarized, PI3K/AKT signaling, as well as TGF-β, Wnt/β-catenin and Notch are important oncogenic pathways that can induce cancer cell stemness, possibly through the activation of epithelial–mesenchymal transition (EMT) [44]. Studies have shown that AKT is dephosphorylated by PP2A and pleckstrin homology domain-containing leucine-rich repeat phosphatases. Although PP2A predominantly dephosphorylates Thr308 of AKT, it also targets Ser473 under certain conditions [45]. Our data further suggests that ONECUT2 may downregulate PP2A phosphorylation, thereby promoting AKT phosphorylation at S473. The ONECUT2 promoter has potential binding sites for the PP2A regulatory subunit PPP2R4, which assists in the biosynthesis of PP2A complexes [46]. Thus, we hypothesized that ONECUT2 may inhibit the transcriptional activity of the PP2A regulatory subunit PPP2R4, decreasing the presence of PP2A complexes and the subsequent downregulation of PP2A phosphorylation. This relieved the dephosphorylation pressure on the AKT substrate, culminating in increased AKT phosphorylation at S473.

Previous studies have shown that Wnt/β-catenin signaling can convert HP-infected gastric epithelial cells into stem-like progenitor cells that promote gland expansion and growth [47–49]. Owing to its ability to manipulate the progenitor and stem cell zones, HP colonization can activate stem cells, increase their populations, accelerate the proliferation of Lgr5(+) stem cells, and induce the expression of stem cell-related genes. These actions lead to changes in metabolic dynamics and glandular hyperplasia [48, 50]. The ability of Lgr4 to regulate Lgr5 expression is critical for HP-induced glandular hyperplasia and inflammation [51]. Our experiments further suggest that AKT phosphorylation at S473 enhances β-catenin phosphorylation at S552, allowing β-catenin to enter the nucleus and stimulate expression of stemness-associated genes, including SOX2, SOX9, CD44, and LGR4. This process ultimately modulates GC cell stemness. Whether cell stemness is related to EMT deserves further study. Taken together with the results of HP infection in ONECUT2 downregulated GC cells, we postulate that HP-induced upregulation of ONECUT2 promotes GC cell stemness via the PPP2R4/AKT/β-catenin signaling pathway, thereby promoting GC development.

**Fig. 7:**
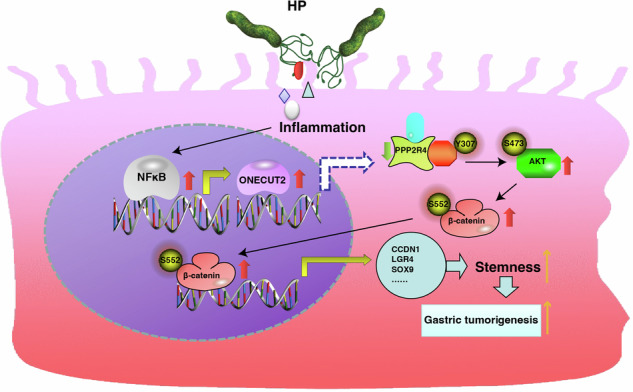
Schematic representation of the mechanism of ONECUT2 in the development of HP-induced gastric carcinogenesis. HP-induced upregulation of ONECUT2 enhances GC cell stemness via PPP2R4/AKT/β-catenin signaling, thereby promoting GC development.

In conclusion, our comprehensive study demonstrated the significant role of ONECUT2 in promoting HP infection-associated GC and revealed the underlying mechanisms. HP-induced upregulation of ONECUT2 enhances GC cell stemness via PPP2R4/AKT/β-catenin signaling, thereby promoting GC development (Fig. 7). The complex regulatory relationships among HP infection, ONECUT2, and GC require further investigation. These findings suggest promising therapeutic strategies and novel targets for GC.

### Supplementary information

Supplementary materials

## Data Availability

The data generated in this study are publicly available in the Genome Sequence Archive (GSA) database (accession numbers: HRA005558 and HRA005585) and the GEO database (GSE267263). The public data analyzed in this study were obtained from the NCI (https://portal.gdc.cancer.gov/) and the Gene Expression Omnibus (GEO) database (www.ncbi.n1m.nih.gov/gds/). All the other raw data generated in this study are available upon request from the corresponding author.
