# Supplementary material for: Increased ONECUT2 induced by Helicobacter pylori promotes gastric cancer cell stemness via an AKT-related pathway
Source: Cell Death Dis. 2024 Jul 12;15(7):497. doi: 10.1038/s41419-024-06885-2 (PMC11245518; doi:10.1038/s41419-024-06885-2)
Supplement: Supplementary file 1 — Supplementary materials [file 41419_2024_6885_MOESM1_ESM.pdf]

Supplementary Figures

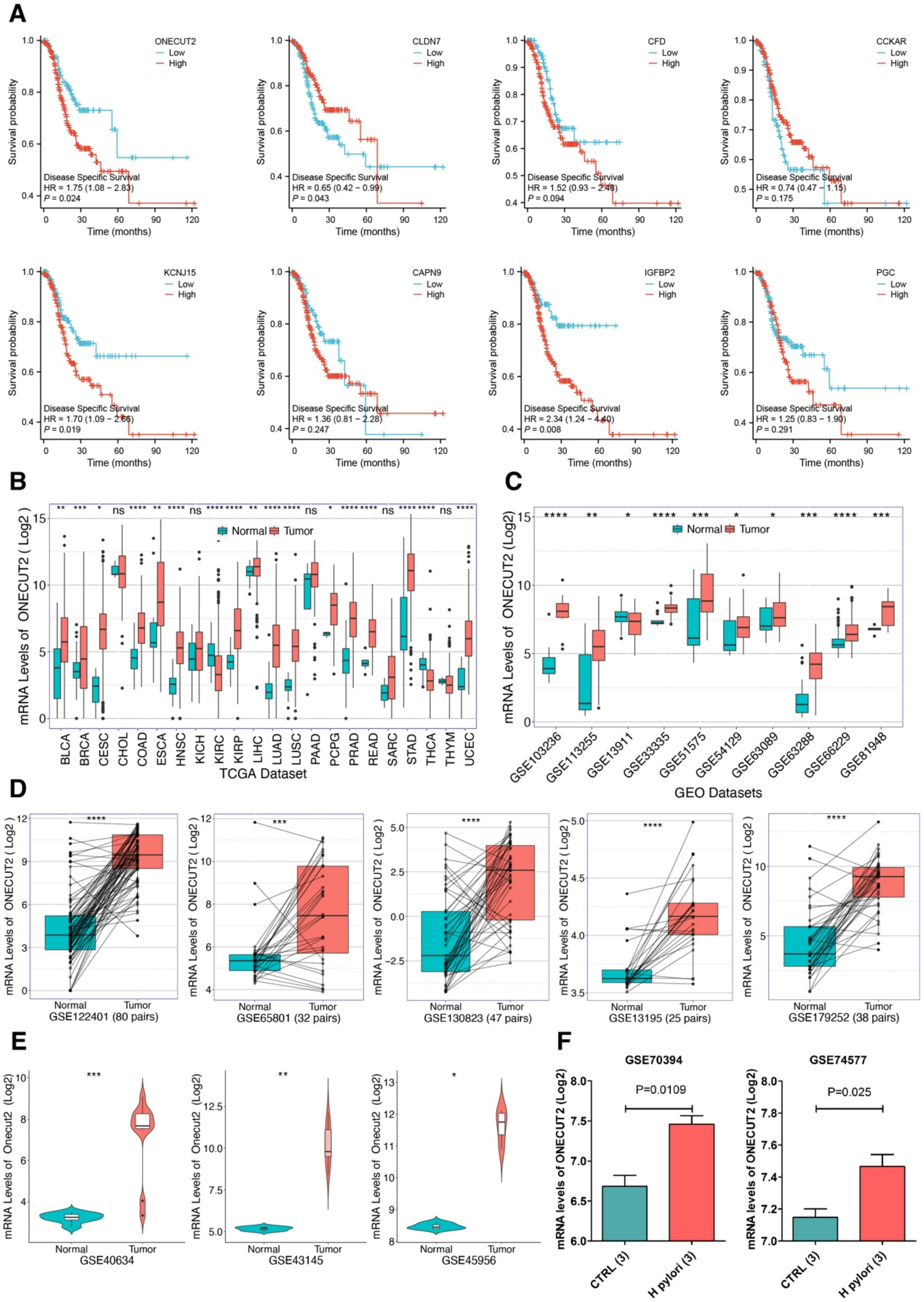

Supplementary Figure 1. Public database validation of ONECUT2 upregulation in GC and HP-infected tissue.

Related to Figure 1. (A) Disease-free survival curves corresponding to eight candidate genes. (B) Differential ONECUT2 mRNA expression levels were observed between tumor and healthy tissue in a variety of cancer types, as reported in the TCGA pan-cancer database. (C) Differential mRNA expression levels of ONECUT2 between tumor and healthy tissue, as reported in the GEO datasets. (D) Differences in ONECUT2 mRNA expression levels between GC and paired healthy tissue, as shown in the GEO datasets. (E) Differences in mRNA levels of ONECUT2 between tumor and healthy tissue according to the mouse GC database. (F) Differences in ONECUT2 mRNA levels between human HP-infected GC and healthy cell lines according to the relevant databases. NS, not significant; *P<0.05; **P<0.01; ***P<0.001; ****P<0.0001.

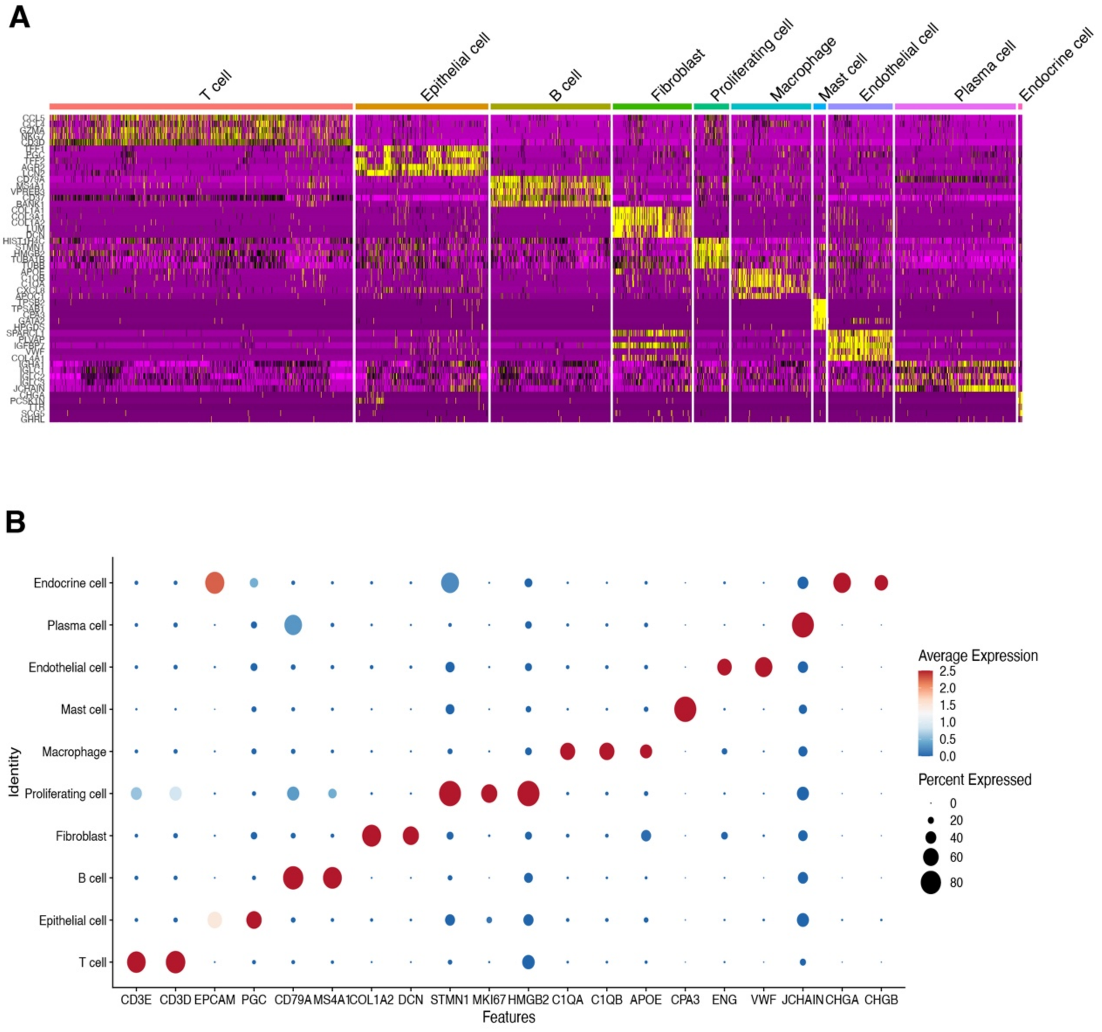

Supplementary Figure 2. Single-cell RNA sequencing of nine human gastric adenocarcinoma samples.

Related to Figure 1. Heatmap (A) and dotplot (B) showing the marker genes and clusters of ten different cell types.

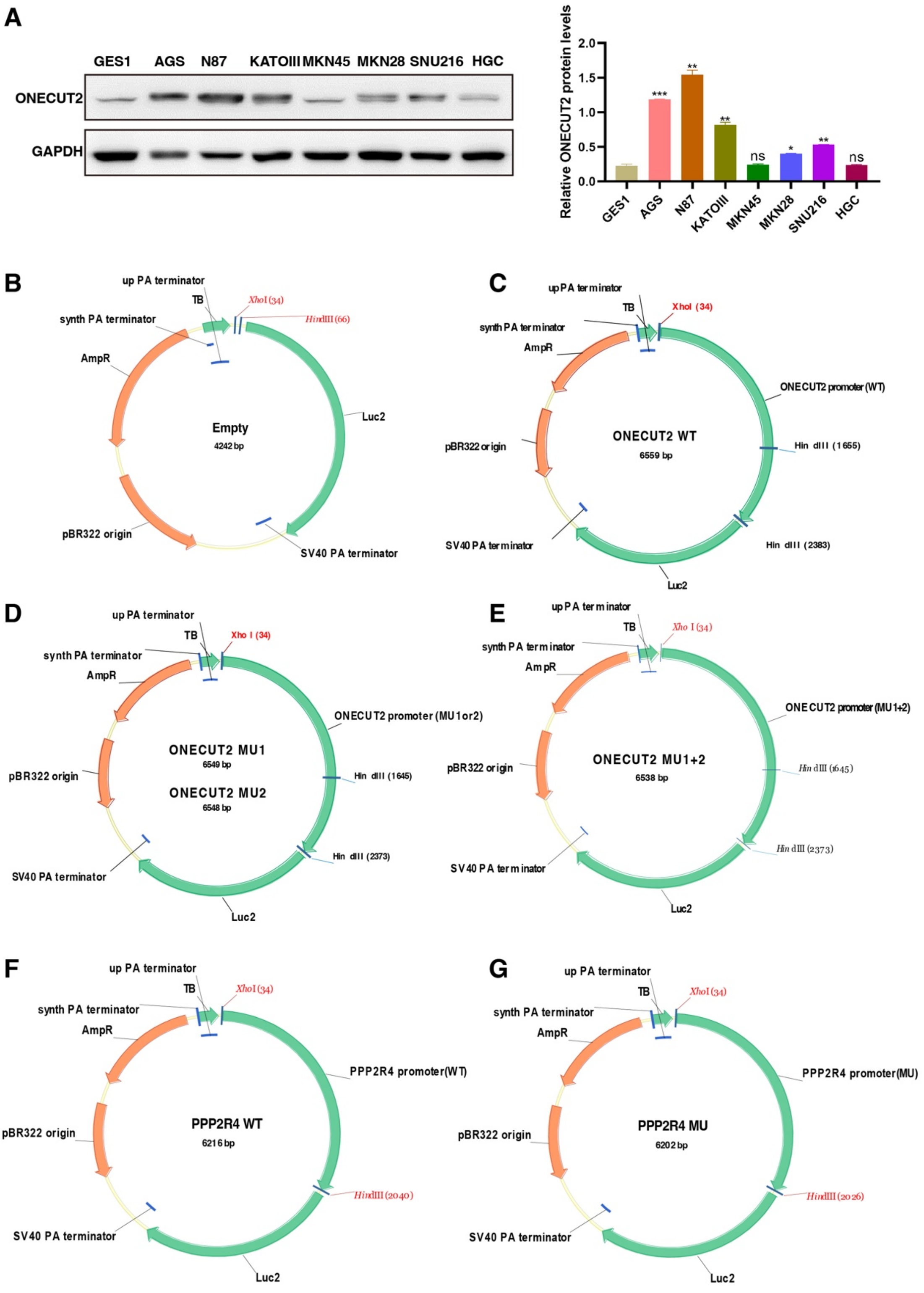

Supplementary Figure 3. Selection of GC cell lines and ONECUT2 luciferase reporter plasmid schematic.

Related to Figures 2 and 3. (A) Western blot analysis and quantification of ONECUT2 protein levels in the healthy gastric epithelial cell line GES-1 and a series of GC cell

lines, including AGS, NCI-N87, KATOIII, MKN45, MKN28, SNU-216, and HGC. (B-G) Luciferase reporter assay plasmid classes: empty luciferase reporter plasmid (B), luciferase reporter plasmids with the ONECUT2 promoter region (C), three variants with predicted binding site mutations (D and E), a luciferase reporter plasmid with the PPP2R4 promoter region (F), and a predicted binding site mutation (G).

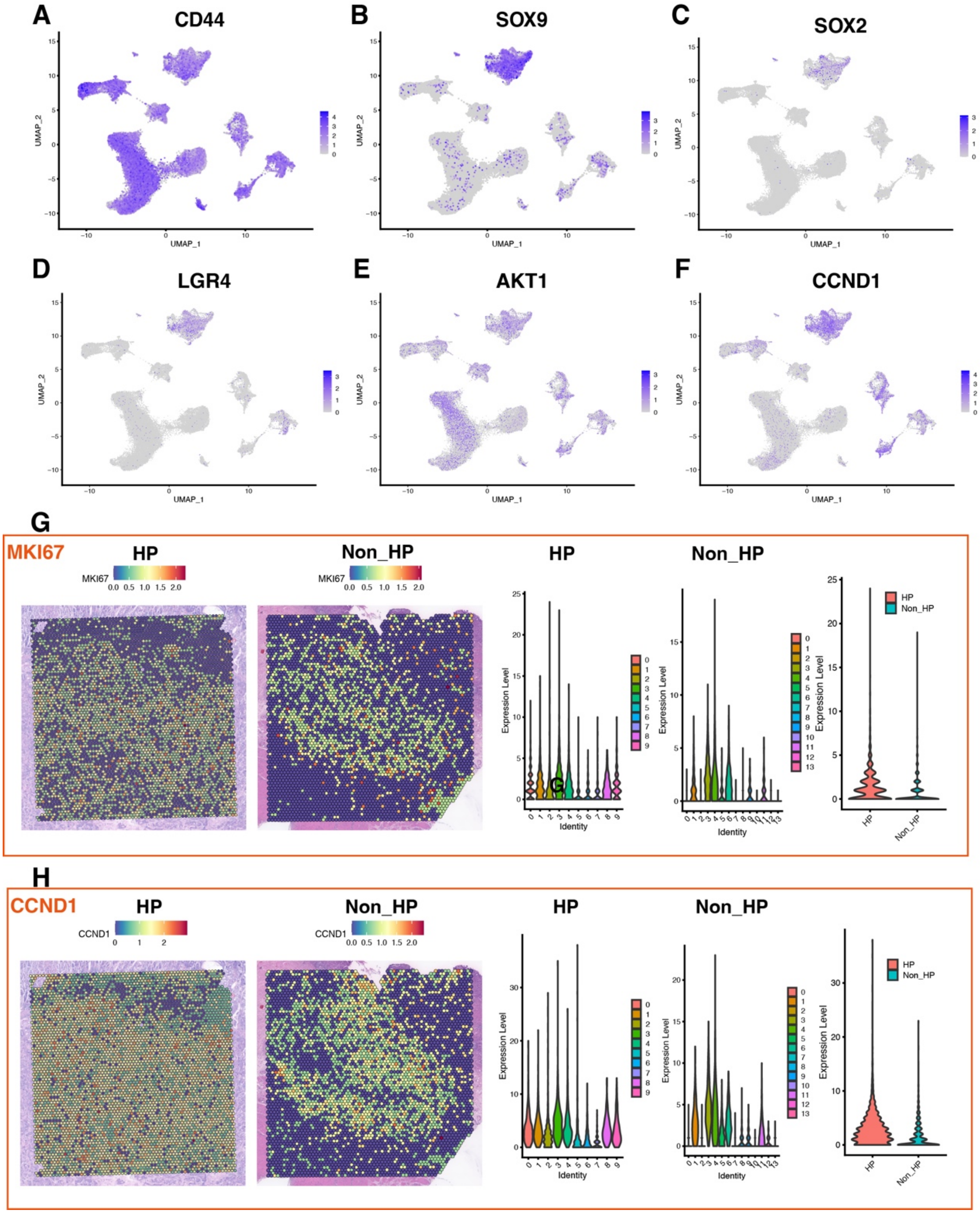

Supplementary Figure 4. Expression of various markers in nine human STAD samples and in Po1 and Ne1 sections derived from HP-infected and non-HP-infected GC tissue.

Related to Figures 4 and 5. (A-F) UMAP of nine human STAD samples showing the expression of CD44 (A), SOX9 (B), SOX2(C), LGR4 (D), AKT1 (E), and CCND1 (F) in the epithelial cell clusters. (G) Spatial transcriptome analysis of Po1 and Ne1 sections

revealed increased MK167 mRNA levels in HP-infected GC tissue. (H) Spatial transcriptome analysis of Po1 and Ne1 sections revealed increased CCND1 mRNA levels in HP-infected GC tissue.

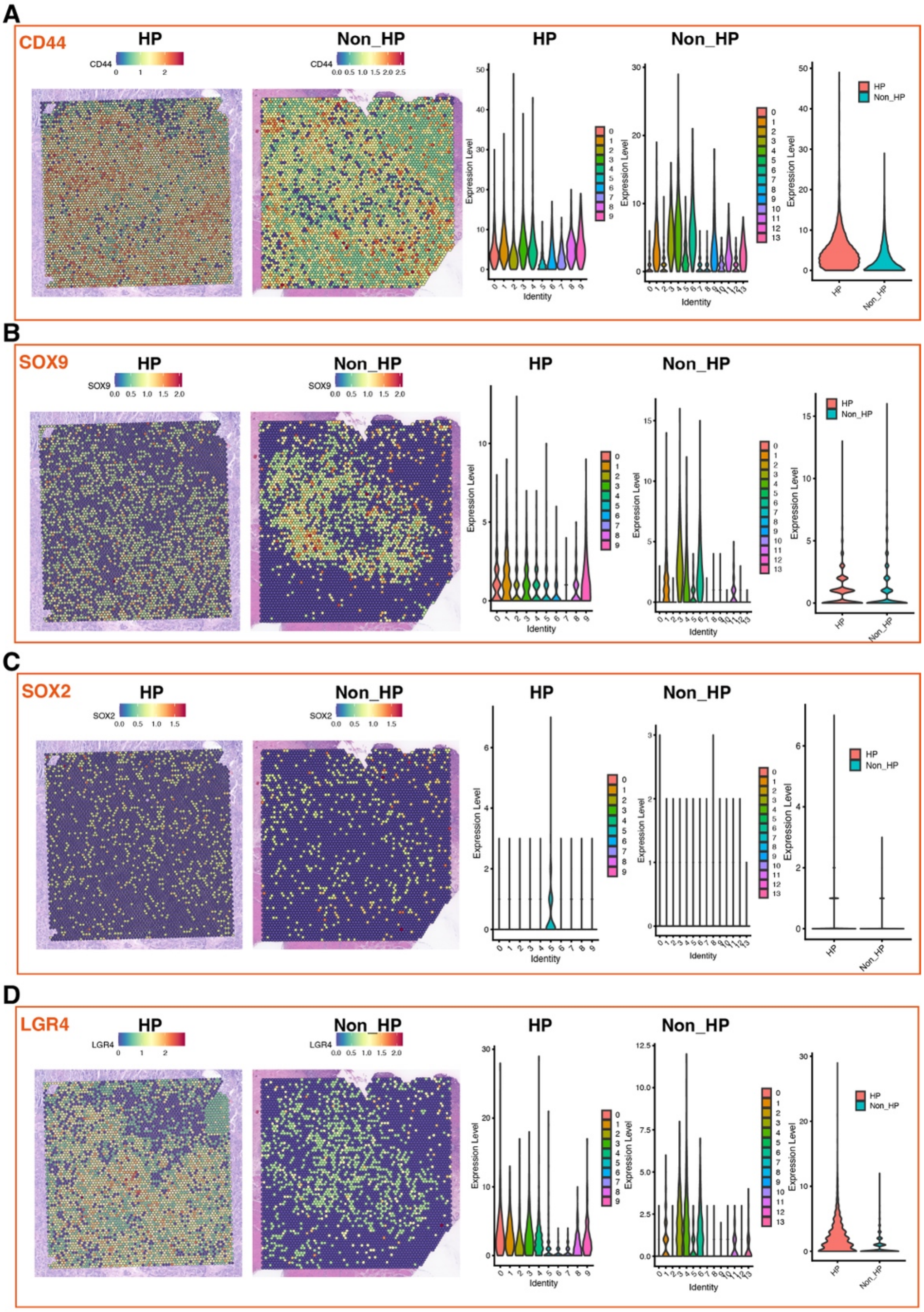

Supplementary Figure 5. Expression of various markers in Po1 and Ne1 sections of HP-infected and uninfected GC tissue.

Related to Figure 4. (A-D) Spatial transcriptome analysis of Po1 and Ne1 sections revealed upregulation of CD44 (A), SOX9 (B), SOX2 (C), and LGR4 (D) mRNA levels in HP-infected GC tissue.

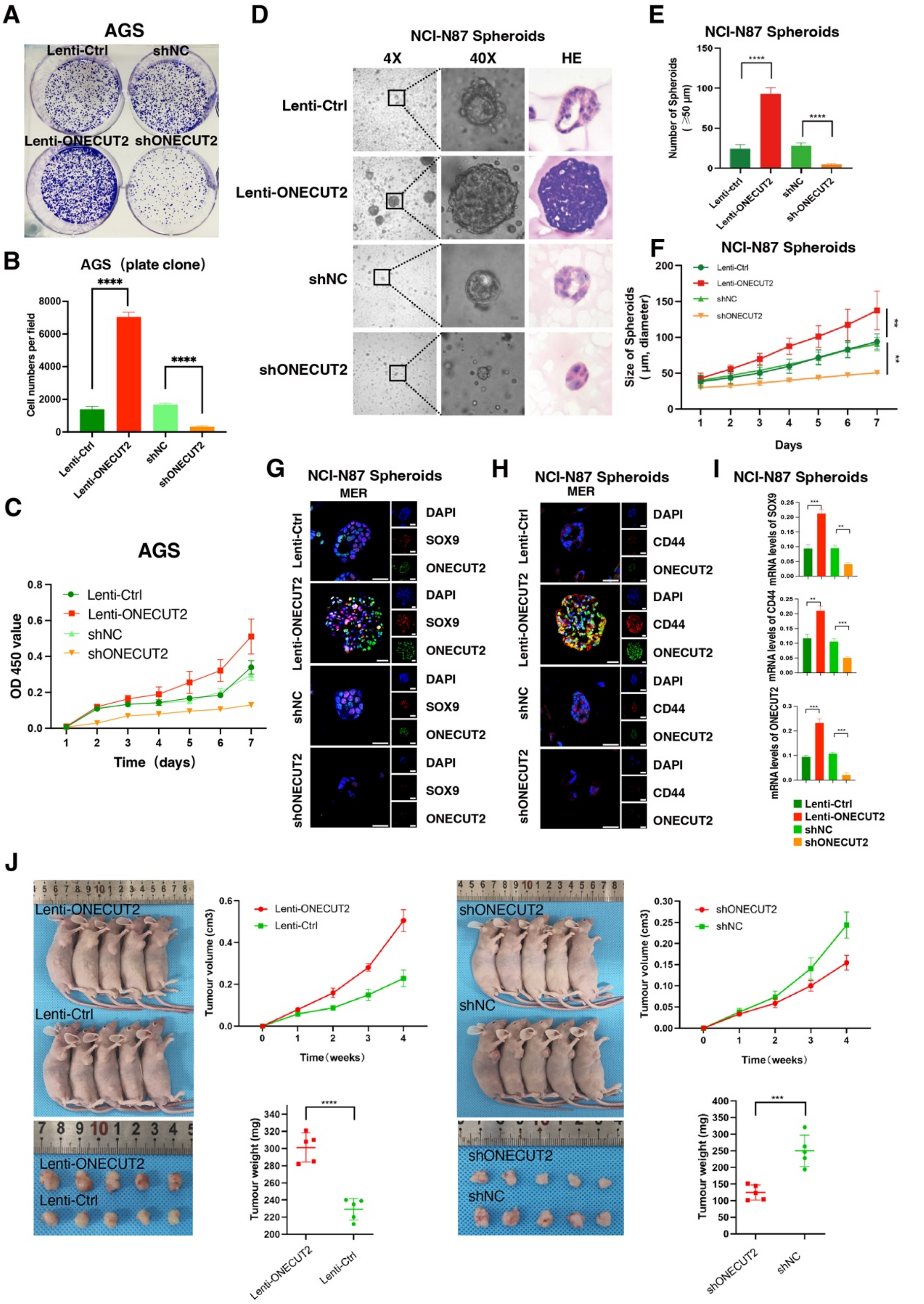

Supplementary Figure 6. Enhancement of GC cell stemness by ONECUT2.

Related to Figure 4. (A) Colony formation assay showing that ONECUT2 overexpression (Lenti-ONECUT) increased the proliferative capacity of AGS cells. (B) Significant differences between the control (Lenti-Ctrl and shNC) and ONECUT2

modified (Lenti-ONECUT2 and shONECUT2) groups were determined by the statistical analysis of colony counts. (C) Cell growth curves derived from CCK-8 assays highlight a significant increase in AGS cell proliferation upon ONECUT2 overexpression. (D) Bright-field microscopy and HE staining images demonstrate the effects of ONECUT2 overexpression and downregulation in NCI-N87 spheroids. (E) Quantification of spheroids revealed a marked increase in ONECUT2 overexpression and less downregulation in NCI-N87 cells. (F) Spheroid diameter analysis revealed that NCI-N87 spheroids were significantly larger when ONECUT2 was overexpressed, whereas spheroid formation was almost absent when ONECUT2 was downregulated. (G) Immunofluorescence staining of 3D NCI-N87 spheroids showing higher SOX9 expression in the Lenti-ONECUT2 group and lower SOX9 expression in the shONECUT2 group compared with the control group. (H and I) Immunofluorescence staining and mRNA expression of SOX9, CD44, and ONECUT2 in NCI-N87 spheroids under the indicated conditions. (J) Subcutaneous tumor formation in nude mice showed that ONECUT2 overexpression enhanced tumorigenesis (n=5). Scale: 20μm. $^{**}P<0.01$; $^{***}P<0.001$; $^{****}P<0.0001$.

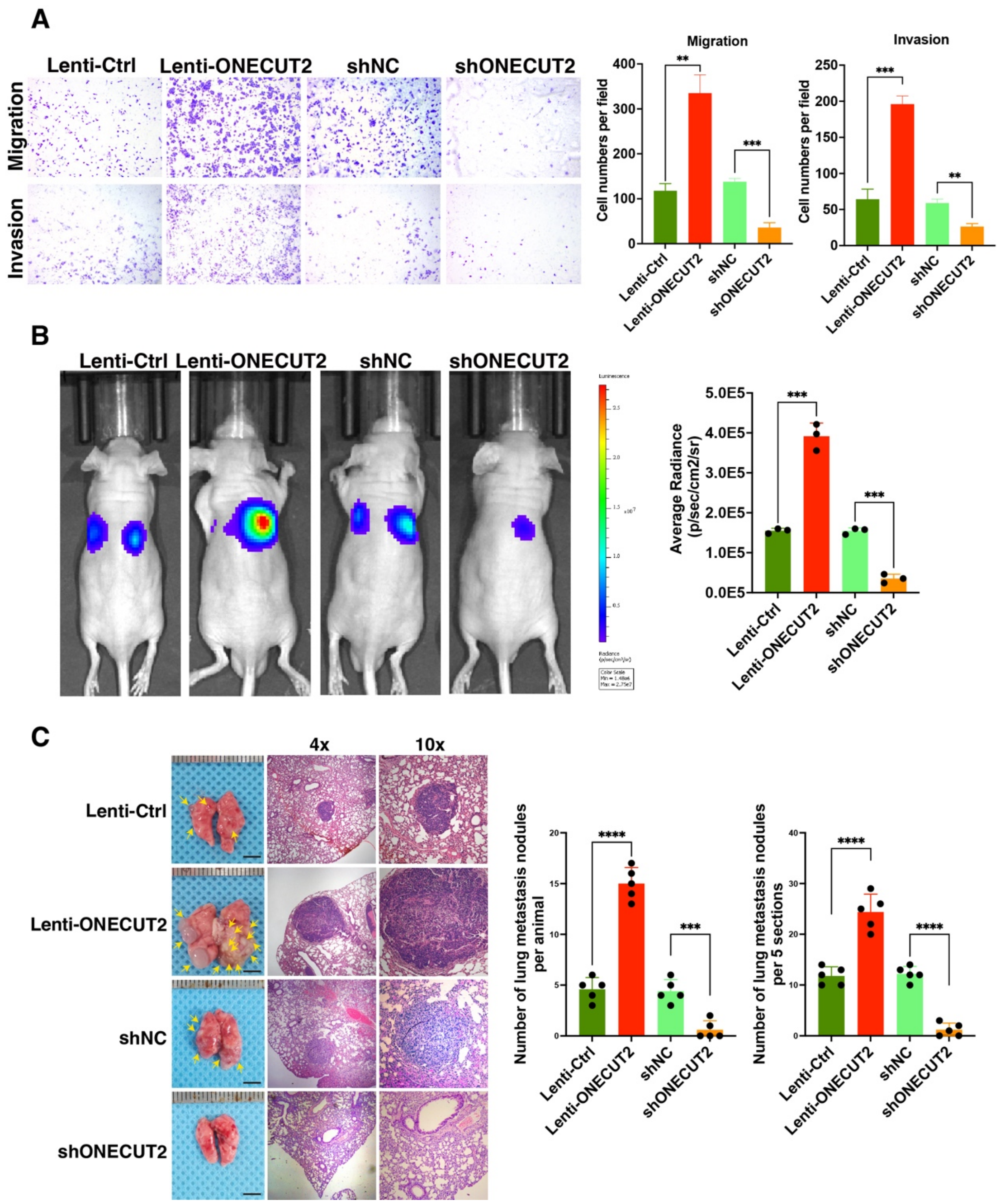

Supplementary Figure 7. ONECUT2 promoted GC cell invasion and metastasis.

Related to Figure 4. (A) Transwell migration and invasion assays showed that ONECUT2 promotes AGS cell migration and invasion. (B) Representative bioluminescence images of mice six weeks after tail vein injection of NCI-N87 cells with ONECUT2 overexpression, downregulation, or control are shown together with the quantification of the images. (C) Representative images of lung metastases and HE staining. The metastatic nodules were counted with and without a microscope and recorded. ONECUT2 overexpression in NCI-N87 cells significantly increased the

number of metastatic lesions in the lungs, while ONECUT2 downregulation in NCI-N87 cells significantly reduced the number of metastatic lesions in the lungs. Scale: 5 mm, ***P<0.001, ****P<0.0001.

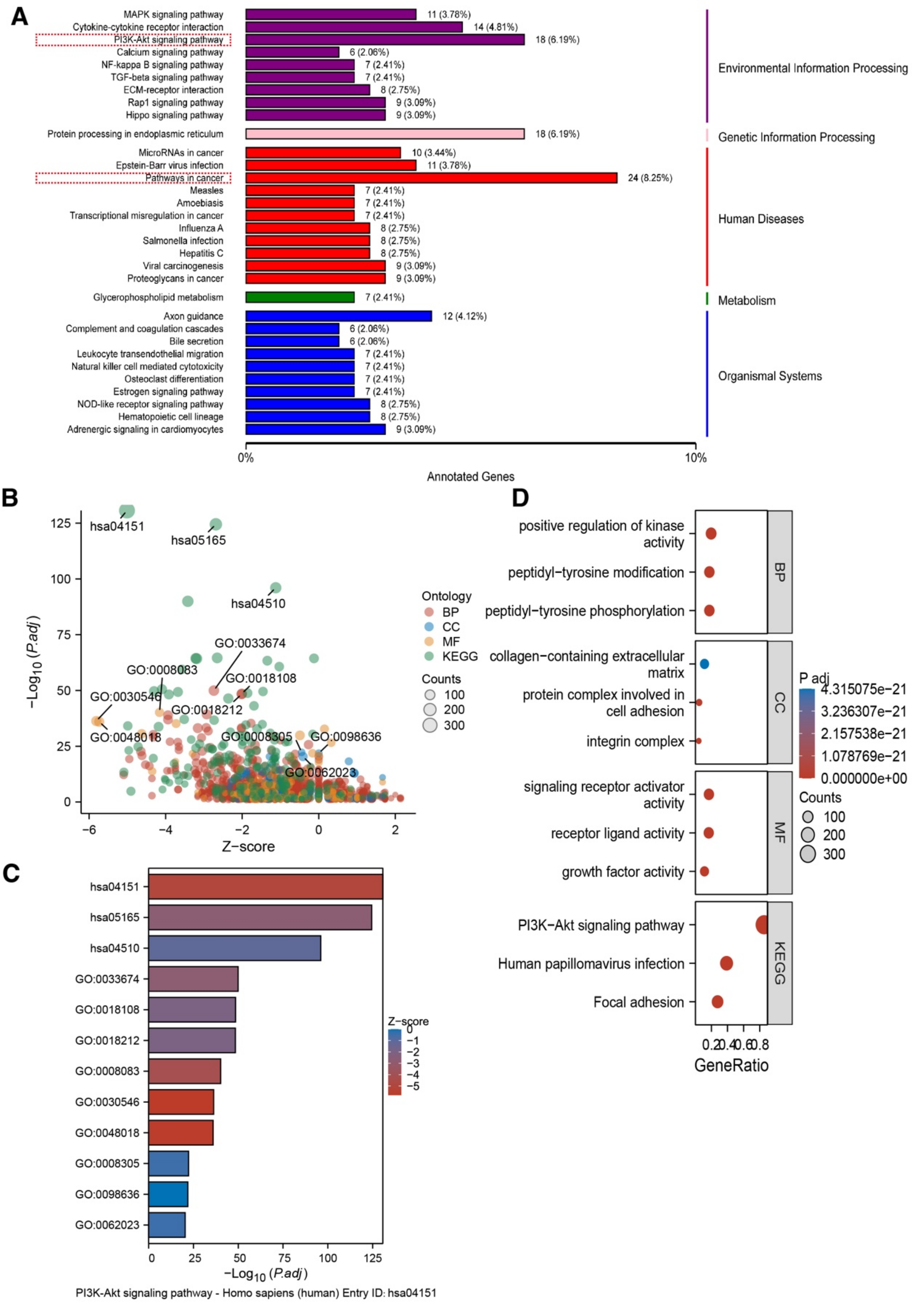

Supplementary Figure 8. ONECUT2 promotes GC cell stemness via an AKT-related pathway.

Related to Figure 5. (A) The Kyoto Encyclopedia of Genes and Genomes (KEGG) pathway classification analysis of RAN-seq data revealed that ONECUT2 is associated with the PI3K-AKT signaling pathway and pathways in cancer. (B-C) Bubble chart and histogram of GO-KEGG combined with FC enrichment analysis showing a significant correlation between ONECUT2 and the PI3K-AKT pathway (hsa04151) in the TCGA public database. (D) Bubble diagram of GO-KEGG enrichment analysis showing a significant correlation between ONECUT2 and the PI3K-AKT pathway in the TCGA public database.

Supplementary Table 1. Clinical characteristics of human gastric cancer samples used for single-cell RNA sequencing

Sample	Age	Sex	Tumor Location	Histopathological diagnosis	*Helicobacter pylori*	Lauren's classification	Pathological Staging
Sample01	55	F	Antrum	Moderately poorly differentiated adenocarcinoma	+	Mixed	pT2N3aM0
Sample02	70	M	Cardia	Poorly differentiated adenocarcinoma, partial signet ring cell carcinoma	-	Mixed	pT3N3aM0
Sample03	63	M	Antrum	Signet ring cell carcinoma	-	Diffuse	pT4aN2M0
Sample04	59	M	Corpus	Poorly differentiated adenocarcinoma	-	Diffuse	pT2N0M0
Sample05	65	M	Corpus	Moderately differentiated adenocarcinoma	-	Intestinal	pT2N0M0
Sample06	62	F	Corpus	Poorly differentiated adenocarcinoma	-	Diffuse	pT4aN1M0
Sample07	69	M	Corpus	Moderately differentiated adenocarcinoma	+	Intestinal	pT2N1M0
Sample08	62	M	Cardia	Moderately poorly differentiated adenocarcinoma	-	Mixed	pT3N2M0
Sample09	53	F	Antrum	Moderately differentiated adenocarcinoma	-	Intestinal	pT2N1M0

Supplementary Table 2. Clinical characteristics of human gastric cancer samples used in spatial transcriptomics

Sample	Age	Sex	Tumor Location	Histopathological diagnosis	*Helicobacter pylori*	Lauren's classification	Pathological Staging
Po1	73	M	Antrum	Moderately poorly differentiated adenocarcinoma	+	Intestinal	pT3N1M0
Ne1	44	F	Corpus	Poorly differentiated adenocarcinoma	-	Diffuse	pT3N0M0

Supplementary Table 3. Relationship between ONECUT2 expression and clinicopathological parameters

Variables	ONECUT2 expression		χ2	P value
	Low expression (n=59)	High expression (n=76)		
Age (years)			5.318	0.021
<65	13	31		
≥ 65	46	45		
Gender			2.568	0.109
Female	9	19		
Male	50	57		
BMI			0.610	0.435
≤25	49	59		

>25	10	17		
HP infection			23.576	<0.001
No	45	26		
Yes	14	50		
Tumor size (cm)			0.001	0.973
<5	27	35		
≥5	32	41		
Tumor Location			2.072	0.558
Upper	24	22		
Middle	8	13		
Lower	20	31		
Cross	7	10		
Degree of differentiation			0.791	0.374
High & Medium	27	29		
Low & Undifferentiated	32	47		
TNM Staging			0.028	0.867
I & II	21	26		
III	38	50		

Note: P<0.05 is statistically significant.

Supplementary Table 4. Sequences of Primers

Gene name	Sequences	
	Forward	Reverse
ONECUT2	AGTAAACTCAAATCTGGCAGGG	TGTTTGGTTCTTGCTCTTTGC
GAPDH	ACATCGCTCAGACACCATG	TGTAGTTGAGGTCAATGAAGGG
CD44	TCTTCAACCCAATCTCACACC	TCCTGTCCAAATCTTCCACC
SOX9	ACTTGCACAACGCCGAG	CTGGTACTTGTAATCCGGGTG
SOX2	CACACTGCCCCTCTCAC	TCCATGCTGTTTCTTACTCTCC
LGR4	ACTCAAAGTTCTAACGCTCCAG	TCCTTCAAAACTGTCCTCGG
ONECUT2 promoter	TGGCCGGTACCTGAGCTCGCTAGCCT<br>CGAGACTGACTCAAATCAT	GCTTTACCAACAGTACCGGATTGCC<br>AAGCTTTCAGTCCATCAGGGCCCG
PPP2R4 promoter	TAACTGGCCGGTACCTGAGCTCGCTA<br>GCCTCGAGAGTGGCGATGGCTGTGGT	GCTTTACCAACAGTACCGGATTGCC<br>AAGCTTGACTCGCCCTCGGCCC

Supplementary Methods

Datasets and bioinformatics analyses

The Cancer Genome Atlas (TCGA) pan-cancer dataset and the TCGA Stomach Adenocarcinoma (TCGA-STAD) cohorts were downloaded from the National Cancer Institute (NCI) (https://portal.gdc.cancer.gov/). A series of unpaired gastric cancer (GC) datasets (GSE103236, GSE113255, GSE13911, GSE33335, GSE51575, GSE54129, GSE63089, GSE63288, GSE66229, and GSE81948), paired GC datasets (GSE122401, GSE65801, GSE130823, GSE13195, and GSE179252), mouse GC datasets (GSE40634, GSE43145, and GSE45956), and human cell line datasets (GSE70394, GSE74577) were downloaded from the Gene Expression Omnibus (GEO) database (www.ncbi.n1m.nih.gov/gds/). The details and context of the RNA-seq data from 36 human GC cases and five HP-infected and five uninfected mice can be found in a previous study [1]. In the processing of raw datasets from Affymetrix® and Illumina®, the "Affy" and "lumi" packages in R language were respectively used for background adjustment via the Robust Multichip Analysis (RMA) algorithm [2, 3]. Non-biological technical biases were minimized using the "ComBat" algorithm [4]. When multiple probes represented a single gene, the mean value was used for the gene expression analysis. To assess the prognostic value of the candidate genes, we used data from the TCGA-STAD cohort with clinical follow-up information. Kaplan-Meier analysis was performed using an online visual platform (www. xiantao. love), using the minimum p-value approach for grouping. We identified ONECUT2-associated differentially expressed RNAs using TCGA-STAD data and applied the empirical Bayesian approach of the Limma R package to distinguish differential expressed genes (DEGs) for each modification pattern. Statistical criteria ($P<0.05$) were used to identify significantly differentially expressed genes. To identify the pathways enriched by ONECUT2, an enrichment analysis was conducted on DGEs using the clusterProfiler package. The z-score corresponding to each enriched entry was computed using the GOplot package based on the numerical values of the given molecules. The results of enrichment

analysis were visualized using the ggplot2 package. Data analyses were performed using R software (version 3.6.1; https://www.r-project.org/).

Processing of single-cell RNA sequencing data

Single-cell RNA sequencing was performed on nine human STAD samples, two with and seven without HP infection. Sequencing data from 10x Genomics were processed using CellRanger software (version 3.1) and aligned to the human reference genome (hg19). The Seurat R package (version 4.2.2) was used to preprocess and analyze the raw gene expression matrices. After applying specific gene and cell inclusion thresholds (including genes detected in at least three cells and cells with at least 200 genes), as well as additional preprocessing (nGene>250 and <5000, nUMI >500 and <15,000, and mito percentage <0.2.) A total of 56,693 cells were retained for further analysis. The data were then normalized. The top 2,000 variable genes were selected for principal component analysis (PCA), and the top 20 principal components were used for cluster analysis. The Harmony package (version 0.1.0) was used to mitigate batch effects among patients, and the COSG package (version 0.9.0) was used to identify marker genes. Cell clusters were manually annotated to known cell types using CellMarker 2.0, based on differential expressed genes (DEGs). These clusters were assigned to ten documented cell types: epithelial cells (EPCAM and PGC), endocrine cells (CHGA and CHGB), fibroblasts (DCN and COL1A2), endothelial cells (ENG and VWF), proliferating cells (STMN1, MKI67 and HMGB2), B cells (MS4A1 and CD79A), plasma cells (JCHAIN), mast cells (CPA3), T cells (CD3D and CD3E), and macrophages (APOE, C1QA, and C1QB). Epithelial cell clusters were further divided into ONECUT2^{+} and ONECUT2^{-} cells according to ONECUT2 expression. Finally, gene set enrichment analysis (GSEA) was performed on cell types using DEGs.

Spatial transcriptomics data processing

Spatial transcriptomics was performed on two human STAD samples: one HP-infected (Po1) and the other uninfected (Ne1). Raw 10× Genomics Visium data were processed by “spaceranger 1.2.1.” Downstream analyses were performed using the Seurat R package (version 4.2.2). Sctransform was performed on the “spatial” assay, followed by PCA (top 30 dimensions) using FindNeighbors, FindClusters, and RunUMAP with

default conditions. The SpatialFeaturePlot function was used to overlay the molecular data on the tissue histology. The VlnPlot function was used to compare the gene expression levels in different clusters or groups.

RNA Extraction and RT-qPCR

Total RNA was extracted using TRIzol reagent (Invitrogen, Carlsbad, CA, USA). After purification, the mRNA was transformed into cDNA according to the instructions provided with the PrimerScript RT Reagent Kit (Takara Bio Inc., Otsu, Shiga, Japan). For Semi-quantitative RT-PCR analyses, SYBR Premix Ex Taq (Takara Bio Inc., Otsu, Shiga, Japan) was utilized on a MiniAmp Plus Thermal Cycler (Thermo Fisher Scientific, Waltham, MA, USA) with preset thermal cycling parameters: an initial period at 95°C for 2 minutes, followed by 35 cycles with 45 seconds at 94°C, 30 seconds at 60°C, and 45 seconds at 72°C, and ultimately, 10 minutes at 72°C.

Quantitative real-time PCR (RT-qPCR) was performed with SYBR Premix Ex Taq (Takara Bio Inc., Otsu, Shiga, Japan) using an Mx3000p real-time PCR platform (Agilent Technologies Inc., Palo Alto, CA, USA). The preset thermal cycling parameters involved a start-up cycle at 95°C for 1 min, and thereafter, 40 cycles comprising 5 seconds at 95°C and 30 seconds at 60°C. Relative mRNA expression normalized to GAPDH mRNA expression was calculated utilizing the $2^{(-\Delta\Delta Ct)}$ method. The oligonucleotide sequences used in this study are listed in **Supplementary Table 4**.

Western blotting

Cell extracts were obtained using RIPA lysis buffer, a Cell Lysis Buffer designed for western blotting, or IP (Beyotime, Shanghai, China) containing PMSF solution (GenStar, Beijing, China) and PhosSTOP (Roche Molecular Biochemicals, Mannheim, BW, Germany). The bicinchoninic acid (BCA) assay (Thermo Scientific, Wilmington, DE, USA) was used to measure protein concentrations. Equal volumes of protein samples were separated via 6-12% SDS-PAGE gel electrophoresis (Bio-Rad, Hercules, CA, USA) and subsequently transferred onto PVDF membranes (Millipore Corp., Billerica, MA, USA).

After blocking with 5% nonfat milk, the membranes were incubated overnight at 4°C with primary antibodies diluted in a dedicated primary antibody dilution buffer

(Beyotime, Shanghai, China). The PVDF membranes were incubated with species-specific secondary antibodies (Huabio, Hangzhou, China) for 1 h at ambient temperature. Horseradish peroxidase (HRP) was detected using an ECL western blotting kit (Advansta, Menlo Park, CA, USA). Images of the protein bands were captured using an ImageQuant LAS 4000 Mini system (General Electric Company, Boston, MA, USA), and band intensities were quantified using ImageJ software (National Institutes of Health, Bethesda, MD, USA). Full and uncropped western blots are presented in the **Supplementary Material**. The primary antibodies used in the experiments included ONECUT2 (1:200, Proteintech, 21916-1-AP), NF-κB p65 (1:500, Cell Signaling Technology, 8242), p-NFκB(s536) (1:500, Cell Signaling Technology, 3033), CagA (1:500; GeneTex, B818M), PP2Aa (1:500, Cell Signaling Technology, 2041), p-PP2A (1:200, R & D Systems, AF3989), PPP2R4 (1:500, Proteintech, 10321-1-AP), AKT (1:1000, Cell Signaling Technology, 9272), p-AKT(S473) (1:1000, Cell Signaling Technology, 4060), β-Catenin (1:1000, Cell Signaling Technology, 8480), p-β-catenin (1:1000, Cell Signaling Technology, 5651), LGR4 (1:200, Proteintech, 20150-1-AP), SOX2 (1:500, Abcam, ab97959), SOX9 (1:500, Abcam, ab185966), CD44 (1:1000, Abcam, ab254530), and GAPDH (1:1000, Abcam, ab9485).

Cell culture

Human GC cell lines, including AGS, NCI-N87, KATOIII, MKN-45, MKN-28, SNU216, and HGC-27, and the human gastric mucosal epithelial cell line, GES-1, were acquired from Cellcook (Guangzhou, Guangdong, China). Cells were cultured in media recommended by the American Type Culture Collection (ATCC, Manassas, VA, USA), supplemented with 10% (v/v) fetal bovine serum (FBS), penicillin (100 units/mL), and streptomycin (100 g/mL). Cells were grown at 37°C in a humidified incubator with a 5% CO_2 atmosphere. BAY 11-7082 (Selleckchem, S2913) 5 ng/ml or human TNF-α (Peprotech, #AF-300-01A-10ug) 100 ng/ml were co-cultured with cells to inhibit or activate the NFκB pathway.

Establishment of cell lines

Lentiviruses for ONECUT2 (NM_004852) overexpression and knockdown and control lentiviruses were purchased from GeneChem Corporation (Shanghai, China). Transfection was performed according to the manufacturer's instructions. The cells were grown overnight at a density of 1×10^5 cells/well and then infected with the viral supernatant at a multiplicity of infection of 10. To isolate stable clones, puromycin (2 μg/ml, Sigma) was utilized for a minimum of 1 week. Cell clones that survived this process were subsequently analyzed using RT-qPCR and western blotting. The cells were collected at specified intervals for other experimental assessments.

HP infection model

We developed *in vitro* and *in vivo* infection models to study the effects of oncogenic HP. For the *in vitro* experiments, AGS and NCI-N87 cells were infected with HP (ATCC 43054). For the *in vivo* model, the gastric mucosa of C57BL/6 mice (The Jackson Laboratory, Bar Harbor, ME, USA) was infected with a rodent-adapted Cag+ HP strain (PMSS1). HP strains were initially cultured on trypticase soy agar with 5% sheep blood agar plates (BD Biosciences, Bedford, Massachusetts, USA) in a 37°C humidified incubator with 10% CO_2 for passage. Cells were then transferred to Brucella broth (BB; BD Biosciences) supplemented with 10% FBS (Invitrogen Life Technologies, Carlsbad, CA, USA) and grown overnight under similar conditions. We co-cultured the cells with the HP ATCC 43054 strain at a 100:1 infection ratio. We also used the PMSS1 strain to infect the gastric epithelial cells of mice by oral gavage, administering $1\text{x}10^9$ colony-forming units per mouse for one or two weeks.

Dual-luciferase reporter assay

A Dual-Luciferase Reporter Assay System (Promega) was used to conduct luciferase reporter assays following the manufacturer’s guidelines. We constructed luciferase reporter plasmids containing the promoter region and 5' UTR sequence of ONECUT2 gene. Briefly, the sequence from 2000 bp upstream of the transcription start site to the 5' UTR (a total of 2343 bp) was selected as the promoter region. Using the forward primer 5'-TGGCCGGTACCTGAGCTCGCTAGCCTCGAGACTGACTCAAATCAT-3' and

the reverse primer 5'-GCTTTACCAACAGTACCGGATTGCCAAGCTTTCAGTCCATCAGGGCCCG-3', this fragment was amplified by PCR and then inserted into the pGL4.10 vector (Promega) to construct the wild-type luciferase reporter plasmid. Based on this wild-type plasmid, two predicted sites (-1463 to -1454: GGGAATTTAC, -914 to -904: TGGTCTTTTCA) were deleted individually or simultaneously to generate three mutant luciferase reporter plasmids (MU1, MU2, MU1+2). Similarly, for PPP2R4, a 2000 bp region upstream of the transcription start site was selected and amplified using the forward primer 5'-TAACTGGCCGGTACCTGAGCTCGCTAGCCTCGAGAGTGGCGATGGCTGTGGT-3' and the reverse primer 5'-GCTTTACCAACAGTACCGGATTGCCAAGCTTGACTCGCCCTCGGCCC-3', then inserted into pGL4.10. A deletion at site -258 to -245 (ATCAAAACGATCTT) created one mutant luciferase reporter plasmid (MU). All constructed plasmids were validated by sequencing to ensure the accuracy of the target sequences and mutations. The primers used are listed in **Supplementary Table 4**. Luciferase reporter plasmids (**Supplementary Figure 3**) were transfected into AGS and NCI-N87 cell lines. The activity of firefly luciferase was normalized to that of Renilla luciferase to determine the transfection efficiency. Each experiment was performed in triplicate.

Bulk RNA sequencing

Three samples were extracted from stable ONECUT2 knockdown NCI-N87 cells and control cells. After RNA extraction, the samples were subjected to bulk RNA sequencing by Biomarker (Beijing, China). Sample libraries were sequenced using an Illumina NovaSeq 6000 sequencer (San Diego, CA, USA). Following sequencing, a series of data mining analyses were performed. Principal component analysis (PCA) was used to evaluate gene expression levels. In addition, a series of analyses were performed, including correlation, differential gene expression clustering, and significance enrichment for gene ontology (GO) functions and KEGG pathways. All these data-related processes are facilitated by biomarkers. Differential expression

analysis was performed using DESeq2. Criteria for identifying DEGs were established as an adjusted p-value less than 0.05 and a Fold Change equal to or greater than 1.

Cell viability assay

Cell survival was measured using the Cell Counting Kit-8 (CCK-8; Cellcook, Guangzhou, Guangdong, China) according to the manufacturer's instructions. AGS cells were cultured in 96-well plates at a density of 1,000 cells per well. To the culture medium, 10% (v/v) CCK-8 was introduced and incubated at 37°C for 1 h at the pre-established times. Subsequently, the relative cell viability was determined by recording the absorbance at 450 nm using an ELX800 microplate reader (BIO-TEK, Winooski, Vermont, USA). Relative cell viability is presented as a percentage of the CCK-8 values of the untreated synchronized cells. This experiment was conducted thrice, with each instance comprising three replicates.

Transwell migration and invasion assays

Cell migration and invasion were examined using Transwell assays. Initially, AGS cells were placed in the upper compartment of a Transwell insert (Corning, New York, NY, USA), with the lower compartment filled with medium supplemented with 10% fetal bovine serum. The inserts were uncoated for the migration and Matrigel-coated for the invasion assays. After a 24-hour incubation for migration assays or 48 h for invasion assays, cells that did not migrate or invade were wiped off the upper surface of the insert. Cells that moved to the lower surface were fixed and stained. The migrated or invaded cells were counted under a microscope. Each assay was performed in triplicate.

3D tumor stem cell spheroid culture

Stable NCI-N87 cells (control or cells subjected to ONECUT2 knockdown or overexpression) were placed in ultra-low attachment 6-well dishes (Corning, USA) and maintained in serum-depleted DMEM/F12 supplemented with 20 ng/ml epidermal growth factor (EGF), 10 ng/mL basic fibroblast growth factor (bFGF), 2% B-27 supplement (Life Technologies), and 2 mM L-glutamine (Life Technologies). Cells were then incubated with 5% CO_2 at 37°C for seven days. The culture medium was refreshed every three days. After incubation, a bright-field microscope was used to

count and measure the diameter of the tumor spheres in three random fields at 100x magnification.

Human GC organoid culture

GC organoids were cultured from human samples following methods established in previous studies [5, 6]. Briefly, tissue was collected from the antrum of the stomach and segmented into 2-3 mm pieces. Cells were digested with 2.5 mg/ml collagenase A (Sigma-Aldrich) for 30 min. Further tissue disruption was achieved by adding 5 mL of dissociation buffer of d-sorbitol and sucrose (both from Sigma), followed by stirring for 2 min. The resulting supernatant was filtered through a 70 μm sieve to collect the crypt fraction, which was then centrifuged at 150 × g for 5 min. After washing with ice-cold phosphate-buffered saline (PBS), the resulting gland pellet was resuspended in MatrigelTM (Trevigen) supplemented with growth factors, as described previously [5]. Finally, 50 μL MatrigelTM suspension was added to each well of a 24-well plate and supplemented with 0.5 mL of IntestiCult Organoid Growth Medium (STEMCELL Technologies, Cambridge, MA). The organoids were incubated in 5% CO_2 at 37°C, and the medium was refreshed every two weeks. Upon reaching the second passage, the organoids were infected with lentiviral particles carrying ONECUT2 knockdown, overexpression, or control plasmid sequences in 15 ml tubes. This process was repeated overnight. One week after infection, the diameter and number of organoids were recorded in three randomly selected fields at 100x magnification using a light microscope.

Tumor formation and metastasis assays

Male BALB/c nude mice aged 4–5 weeks were procured from Beijing Vital River Laboratory Animal Technology Co., Ltd. NCI-N87-knockdown, NCI-N87-overexpression, or control cells were suspended in 100 μL PBS for tumor xenograft formation. We subcutaneously administered 5×10^6 stably transfected NCI-N87 cells into the right axillary hollows of the nude mice. For the limiting dilution assay, 5×10^2, 1×10^3, 5×10^3, 1×10^4, 5×10^4, 1×10^5, 5×10^5, or 1×10^6 cells were injected subcutaneously into the right axillary hollow region (n=5 for each group, three replicates). The tumor volume was determined every three days using the equation V =

(L × W^2)/2 cm^3, where V is the tumor volume, L is the length, and W is the width. At 4–5 weeks post-injection, the mice were euthanized, and the tumors were weighed. The number of tumor-initiating cells was calculated using Extreme Limiting Dilution Analysis (http://bioinf.wehi.edu.au/software/elda/). For the lung metastasis model, we introduced 5 × 10^6 stably transfected NCI-N87 cells into the tail veins of nude mice. After 45 days, the mice were euthanized, and the lungs were examined to identify the histopathological metastatic sites. All animal procedures were approved by the Animal Experiment Ethics Committee of Fujian Medical University (approval no. FJMU IACUC 2021-J-0065).

Human phosphokinase array

According to the manufacturer's instructions, protein phosphorylation was evaluated using a Proteome Profiler Human Phospho-Kinase Array Kit (ARY003C; R&D Systems, Inc. USA and Canada). Equal amounts of protein (600 mg) extracted from NCI-N87 cells (ONECUT2 knockdown and control) were used to compare kinase activity with and without ONECUT2 knockdown.

Immunofluorescence staining

Tissue sections embedded in paraffin were deparaffinized using HistoClear (National Diagnostics, Atlanta, GA). Histopathological examination was performed using a graded series of ethanol solutions. A citric acid buffer was used for antigen retrieval. Following a blocking step using 10% goat serum (Solarbio, Beijing, China, SL050), the sections were incubated overnight at 4°C with primary antibodies. Subsequently, the membranes were incubated with secondary antibodies for 60 min at room temperature in the dark. The primary and secondary antibodies used were mouse anti-ONECUT2 (10 μg/mL, R&D Systems, MAB6294), rabbit anti-SOX9 (5 μg/mL, Abcam, ab185966), rat anti-CD44 (1:200, BD Biosciences, 553131) and rabbit anti-β-catenin (1:50, Huabio, 0417-16) followed by goat anti-mouse Alexa Fluor 488 (1:400, Thermofisher, A-11001), goat anti-rabbit Alexa Fluor 568 (1:400, Thermofisher, A-11011), goat anti-rat Alexa Fluor 568 (1:400, Thermofisher, A-11077) and goat anti-rabbit Alexa Fluor 568 (1:400, Thermofisher, A-11011), respectively. The nuclei were

then counterstained with DAPI (4′,6-diamidino-2-phenylindole dihydrochloride; Solarbio, Beijing, China, C0065), and digital images were captured using a laser confocal microscope (Leica TCS SP5, Wetzlar, Germany).

Nuclear and cytoplasmic extraction procedures

According to the manufacturer's instructions, nuclear and cytoplasmic fractions were isolated from the cells using an M5 Nuclear and Cytoplasmic Extraction Kit (Mei5 Biotechnology, Beijing, China). Briefly, after rinsing with PBS, the cells were collected by centrifugation and treated with 1 mL of Nc-Buffer A. Immediately before protein extraction, the cell pellet (approximately 1×10^7 cells) was treated with a protease inhibitor cocktail. After incubation on ice for 20 min, NC-buffer B was added, followed by incubation on ice for 1 min. After centrifugation at 12,000 rpm for 15 minutes at 4°C, the supernatant (cytoplasmic proteins) was transferred and stored at -20°C. The remaining pellet was resuspended in Nc-Buffer C containing protease inhibitors, vortexed periodically for 40 min, and centrifuged similarly. The resulting supernatant (nuclear protein) was stored at -20°C. The extracted proteins were subjected to western blot analysis.

Tissue microarray (TMA)

A series of TMA-containing GC samples was constructed. Pathologists briefly reviewed all GC tissue and representative areas free from necrotic and hemorrhagic material marked with paraffin blocks. A 1.5-mm core was punched from the donor blocks for each sample and transferred to the recipient paraffin block at defined array positions using a TMA instrument. Several serial sections (4 μm in thickness) were cut from all TMAs, and one section was stained with H&E (hematoxylin and eosin) as a reference.

Immunohistochemistry and scoring

Tissue microarrays consisting of 135 STAD specimens and their corresponding non-cancerous tissue were obtained from patients who underwent gastrectomy at Fujian Medical University Union Hospital between April 2013 and June 2015. Written informed consent was obtained from all patients before their inclusion in the study.

Ethical approval for the design and reporting of this study was granted by the institutional review board (IRB number:2021KJT028-01). Patients who received neoadjuvant chemotherapy, neoadjuvant radiotherapy, or other antitumor therapies before surgery were excluded. Paraffin blocks of tumor specimens were serially sectioned at 4 μm thickness and mounted on silane-coated slides for immunohistochemical (IHC) analysis. Two experienced pathologists evaluated IHC-stained sections independently, scoring them based on the staining intensity and percentage of positively stained cells. Images were captured using an SMZ1500 inverted microscope. Five fields were randomly selected to assess the intensity and percentage of positive cells in each slice. Staining intensity scores were defined as 0, 1, 2, and 3 for negative, weak, moderate, and strong staining, respectively (**Figure 6B**). The proportion score for the percentage of tumor cells with nuclear staining was 0 for no staining, 1 for 1-10%, 2 for 10-50%, and 3 for >50%. The final IHC score for each slide was expressed as intensity score × proportion score (from 0 to 9). Patients were categorized according to their IHC scores as low (≥3) or high (>3). The antibody for Immunohistochemistry was ONECUT2 (1:50; Proteintech, 26408-1-AP).

Statistical Analysis

Categorical variables were evaluated using the $\chi 2$ test or Fisher's exact test. Cramer's V test was used to correlate ONECTU2 expression with HP infection. Survival time was determined using the Kaplan-Meier method and subjected to log-rank test analysis. Univariate and multivariate Cox regression analyses were performed to identify the factors associated with survival using the Cox proportional hazards model. Statistical significance was set at $P<0.05$. Graphs were generated using GraphPad Prism 9 (La Jolla, CA, USA), and all statistical analyses were performed using SPSS (version 26.0; SPSS Inc., Chicago, IL, USA).

References

1 Cao L, Zhu S, Lu H, Soutto M, Bhat N, Chen Z, *et al.* Helicobacter pylori-induced RASAL2 Through Activation of Nuclear Factor-κB Promotes Gastric Tumorigenesis via β-catenin Signaling Axis. Gastroenterology 2022;162:1716-31.e17.

2 Gautier L, Cope L, Bolstad BM, Irizarry RA. affy--analysis of Affymetrix GeneChip data at the

probe level. Bioinformatics 2004;20:307-15.

3 Du P, Kibbe WA, Lin SM. lumi: a pipeline for processing Illumina microarray. Bioinformatics 2008;24:1547-8.

4 Johnson WE, Li C, Rabinovic A. Adjusting batch effects in microarray expression data using empirical Bayes methods. Biostatistics 2007;8:118-27.

5 Chen Z, Li Z, Soutto M, Wang W, Piazuelo MB, Zhu S*, et al.* Integrated Analysis of Mouse and Human Gastric Neoplasms Identifies Conserved microRNA Networks in Gastric Carcinogenesis. Gastroenterology 2019;156:1127-39.e8.

6 Mahe MM, Aihara E, Schumacher MA, Zavros Y, Montrose MH, Helmrath MA*, et al.* Establishment of Gastrointestinal Epithelial Organoids. Curr Protoc Mouse Biol 2013;3:217-40.

WESTERN BLOTS WITH BAND SIZE

(Index)

Figure 1L

WB WITH BAND SIZE

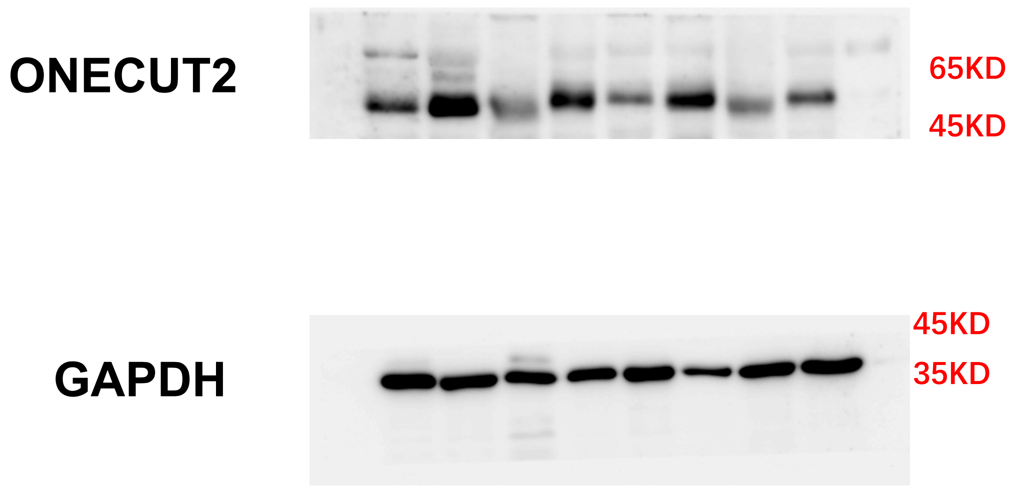

Figure 1L

ONECUT2

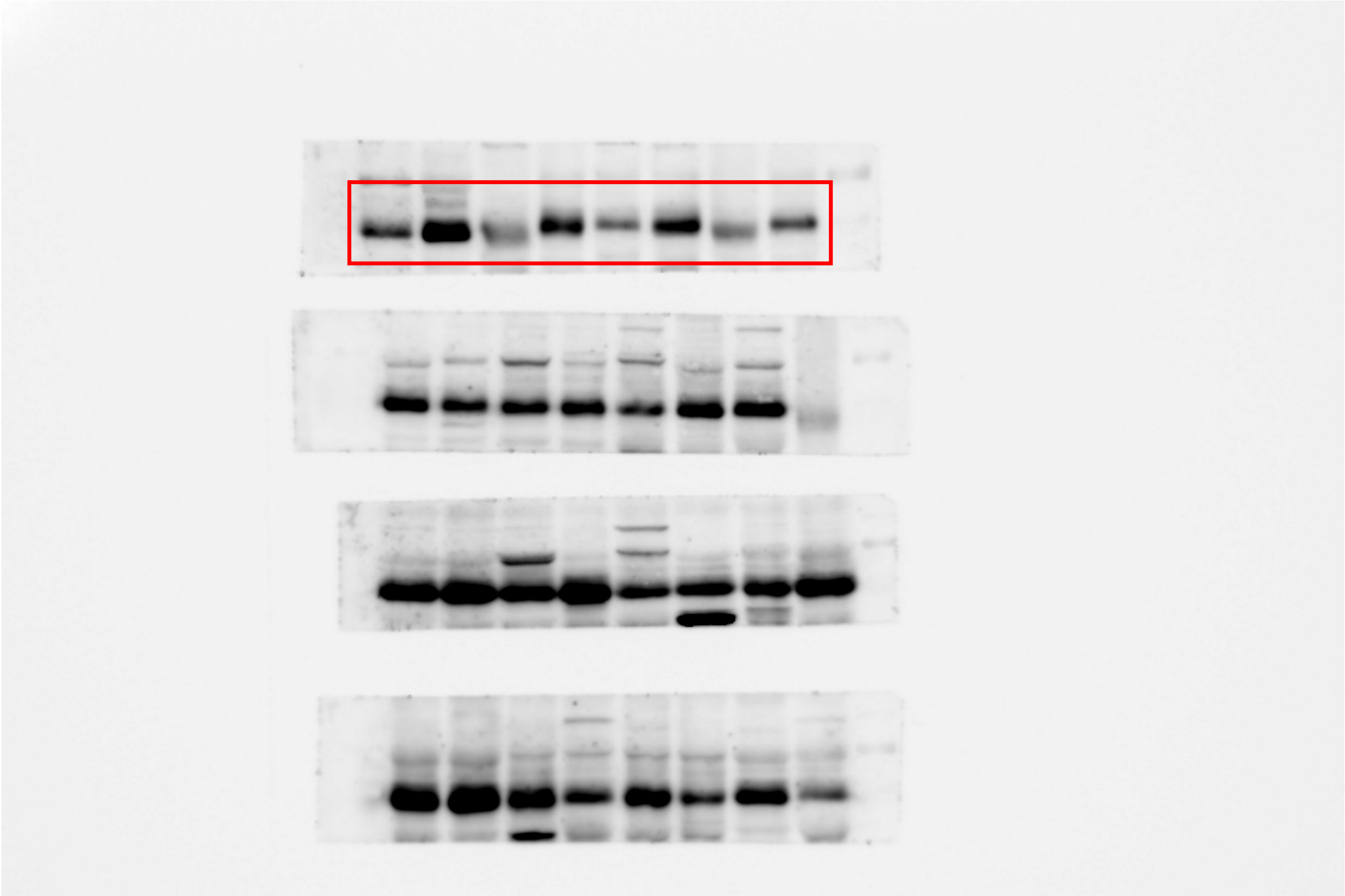

Full and uncropped western blots

Figure 1L

GAPDH

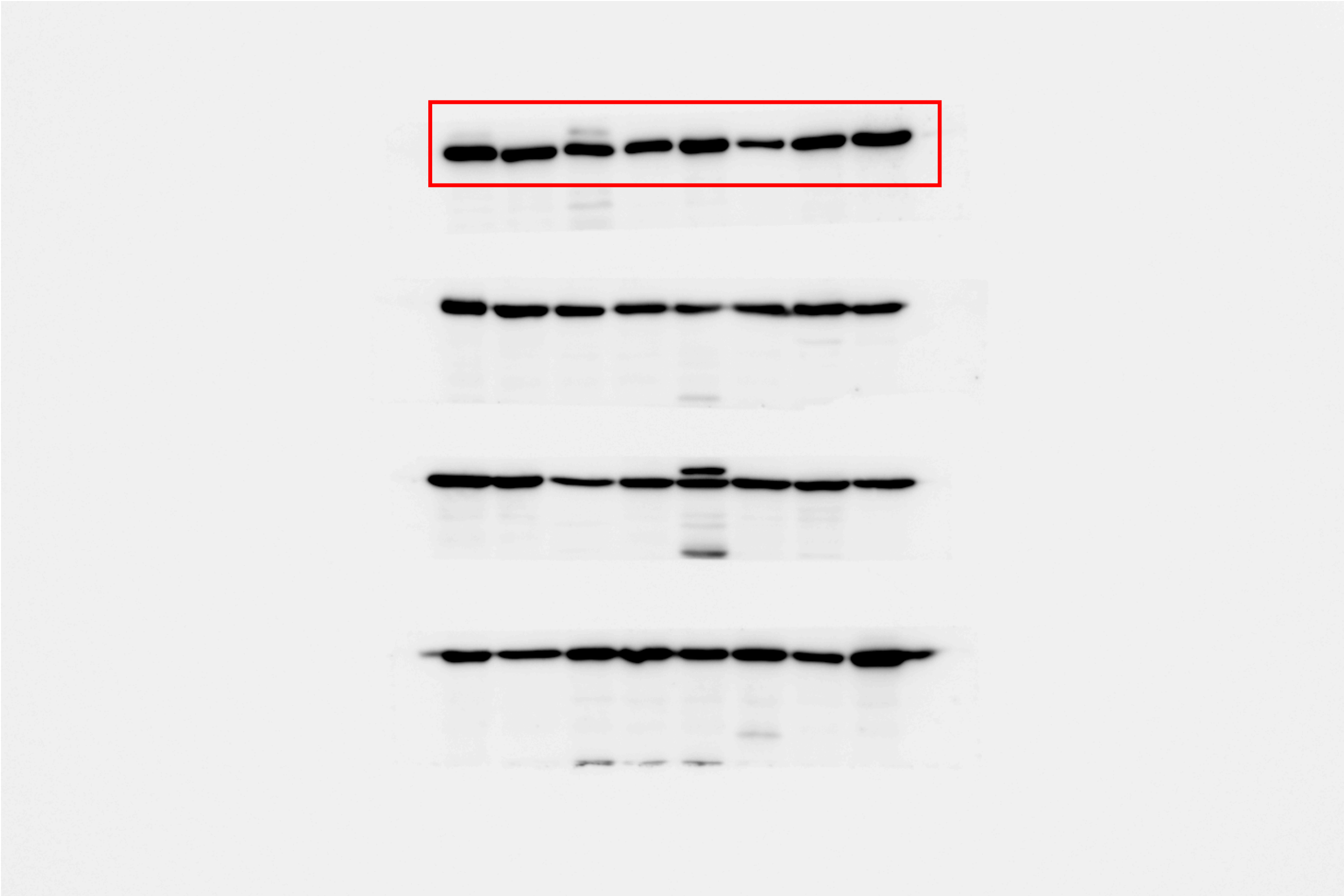

Full and uncropped western blots

Figure 2A

WB WITH BAND SIZE

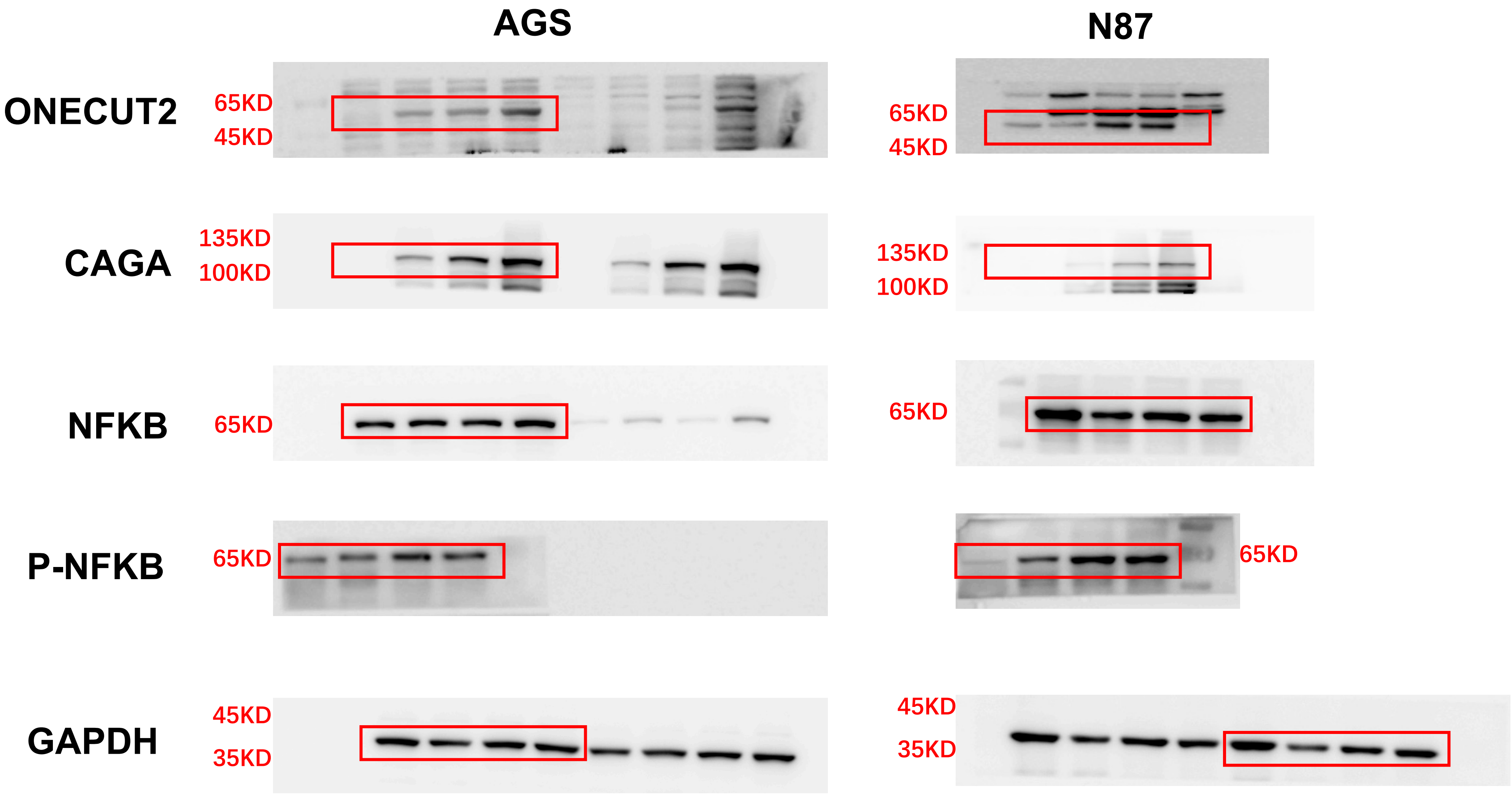

Figure 2A

AGS- ONECUT2

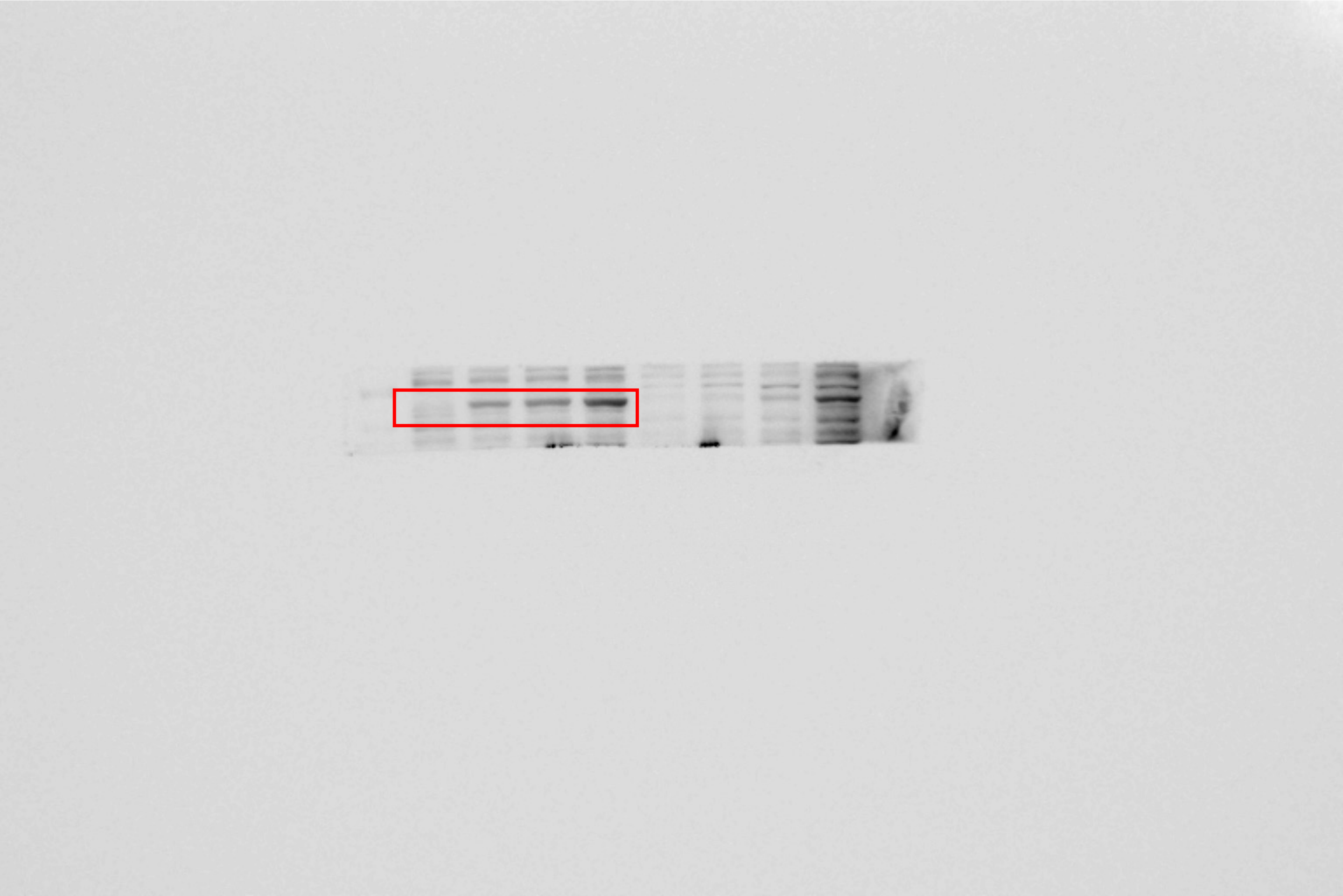

Full and uncropped western blots

Figure 2A

AGS-CAGA

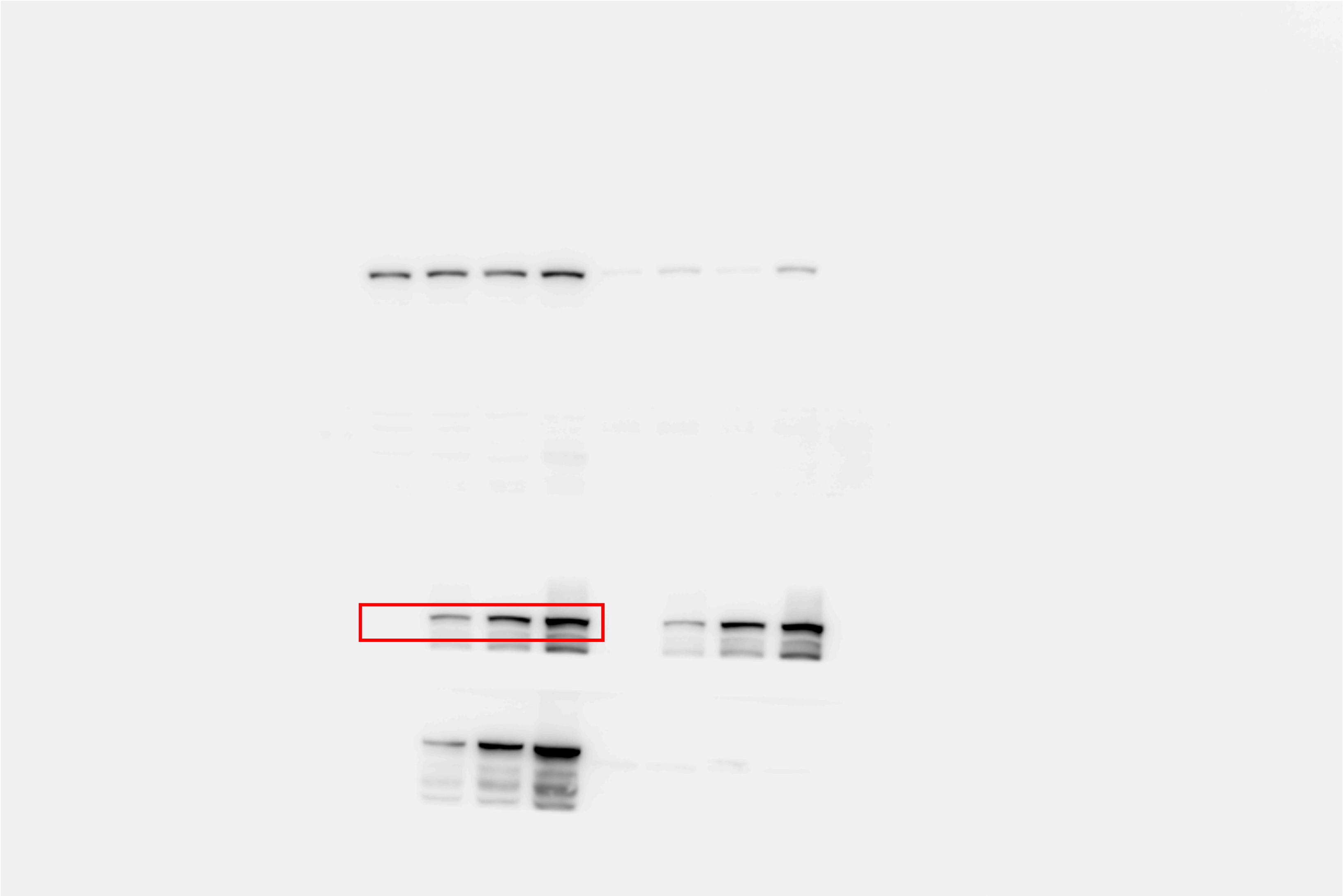

Full and uncropped western blots

Figure 2A

AGS- NFKB

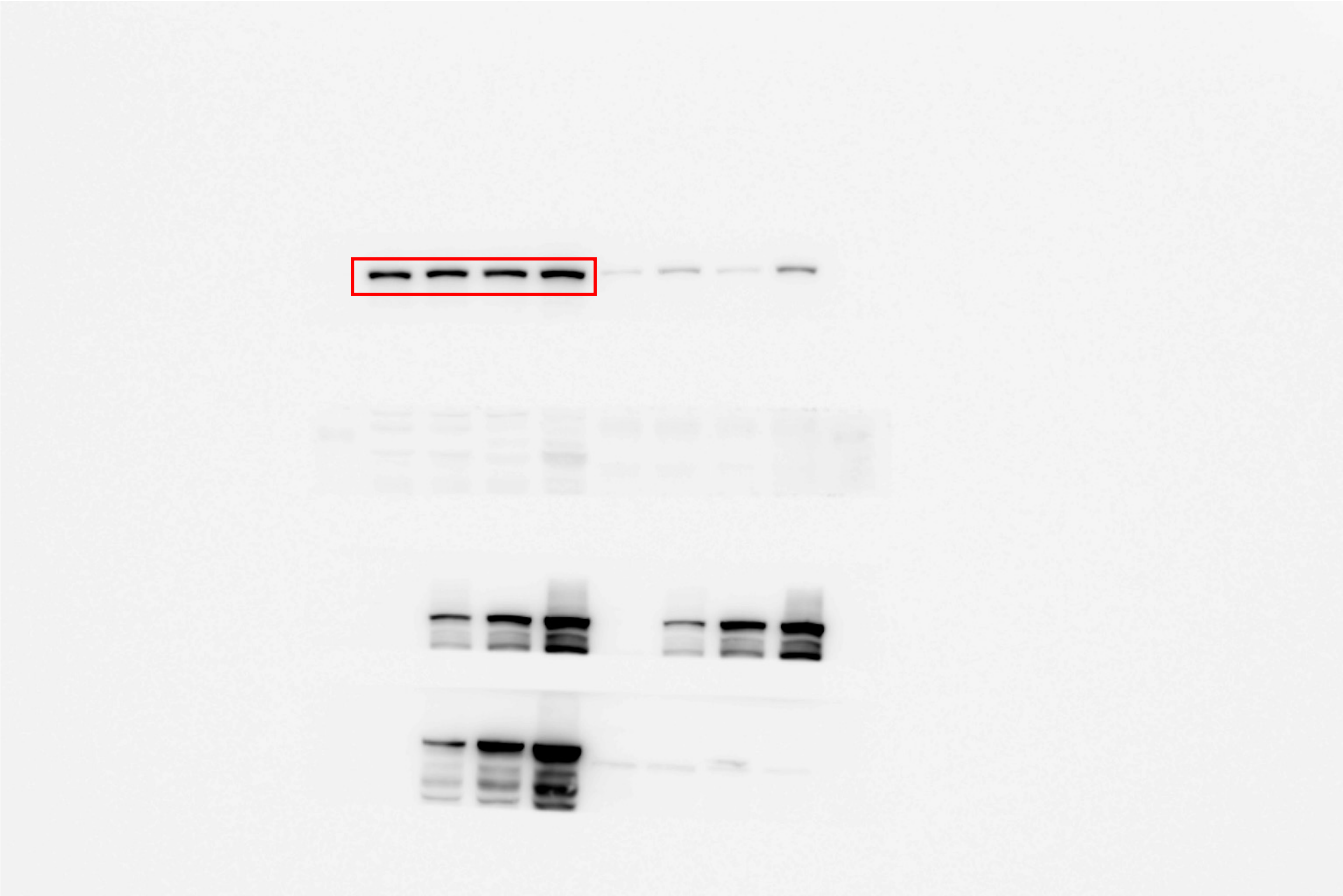

Full and uncropped western blots

Figure 2A

AGS- P- NFKB

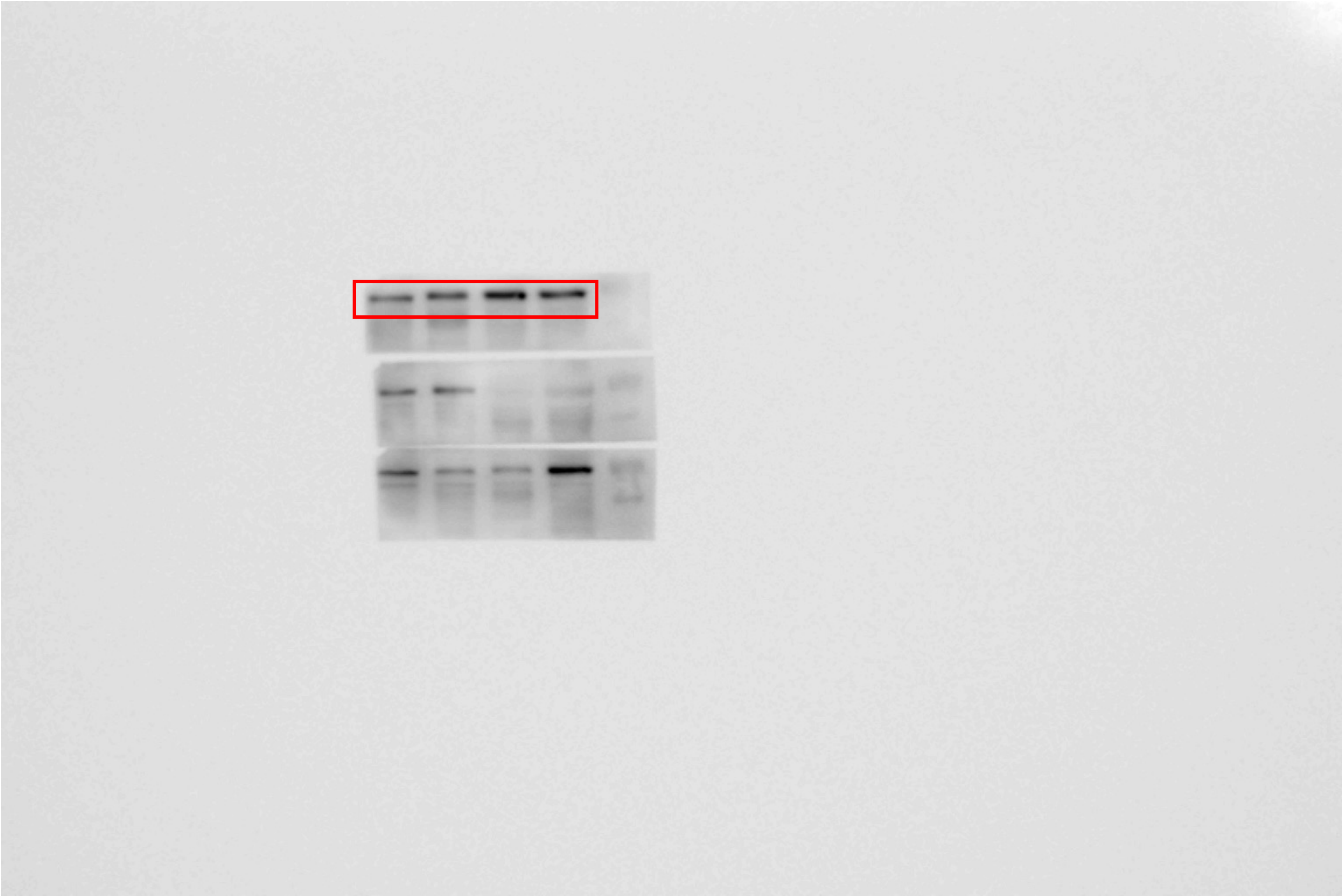

Full and uncropped western blots

Figure 2A

AGS- GAPDH

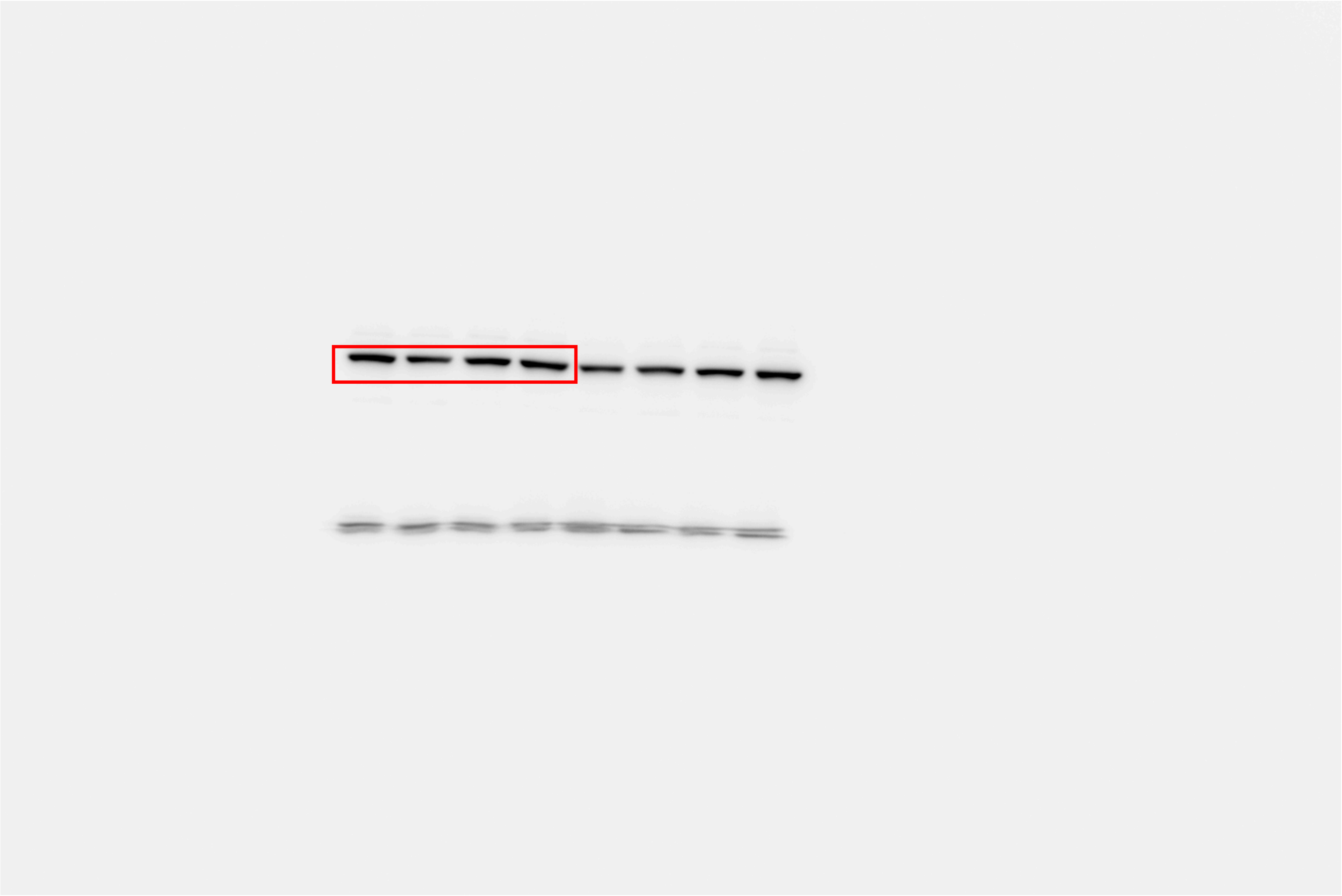

Full and uncropped western blots

Figure 2A

N87- ONECUT2

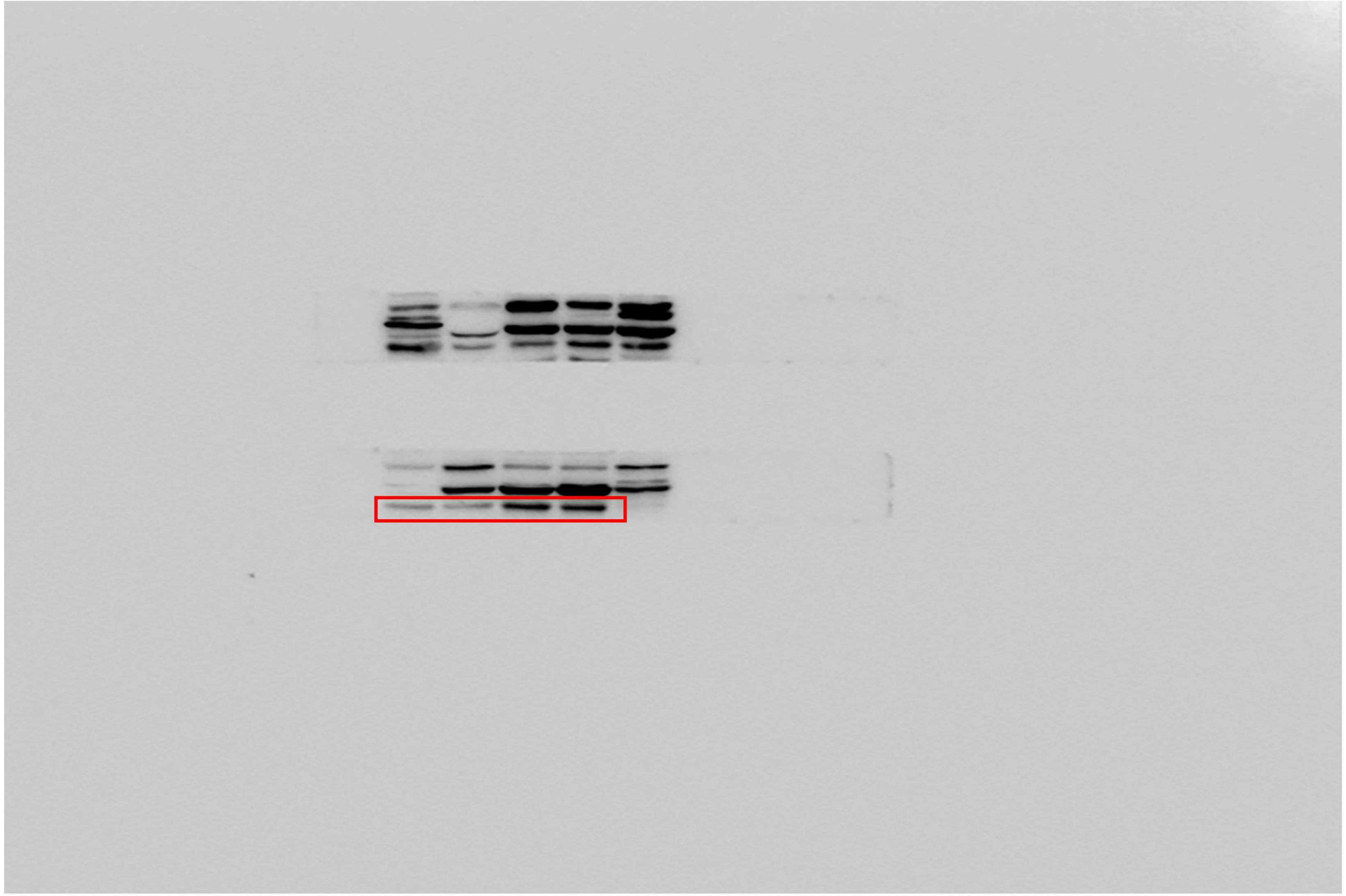

Full and uncropped western blots

Figure 2A

N87-CAGA

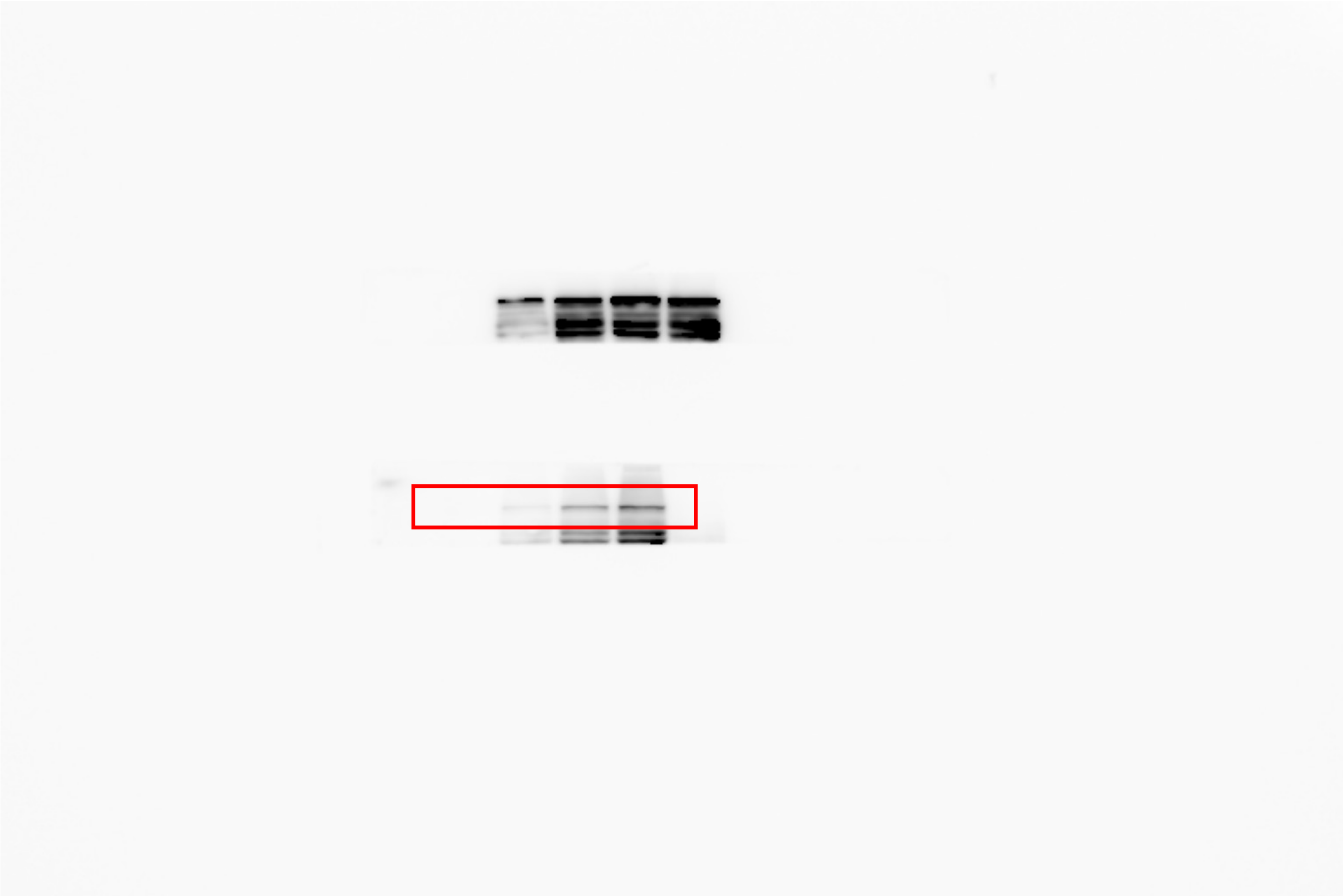

Full and uncropped western blots

Figure 2A

N87- NFKB

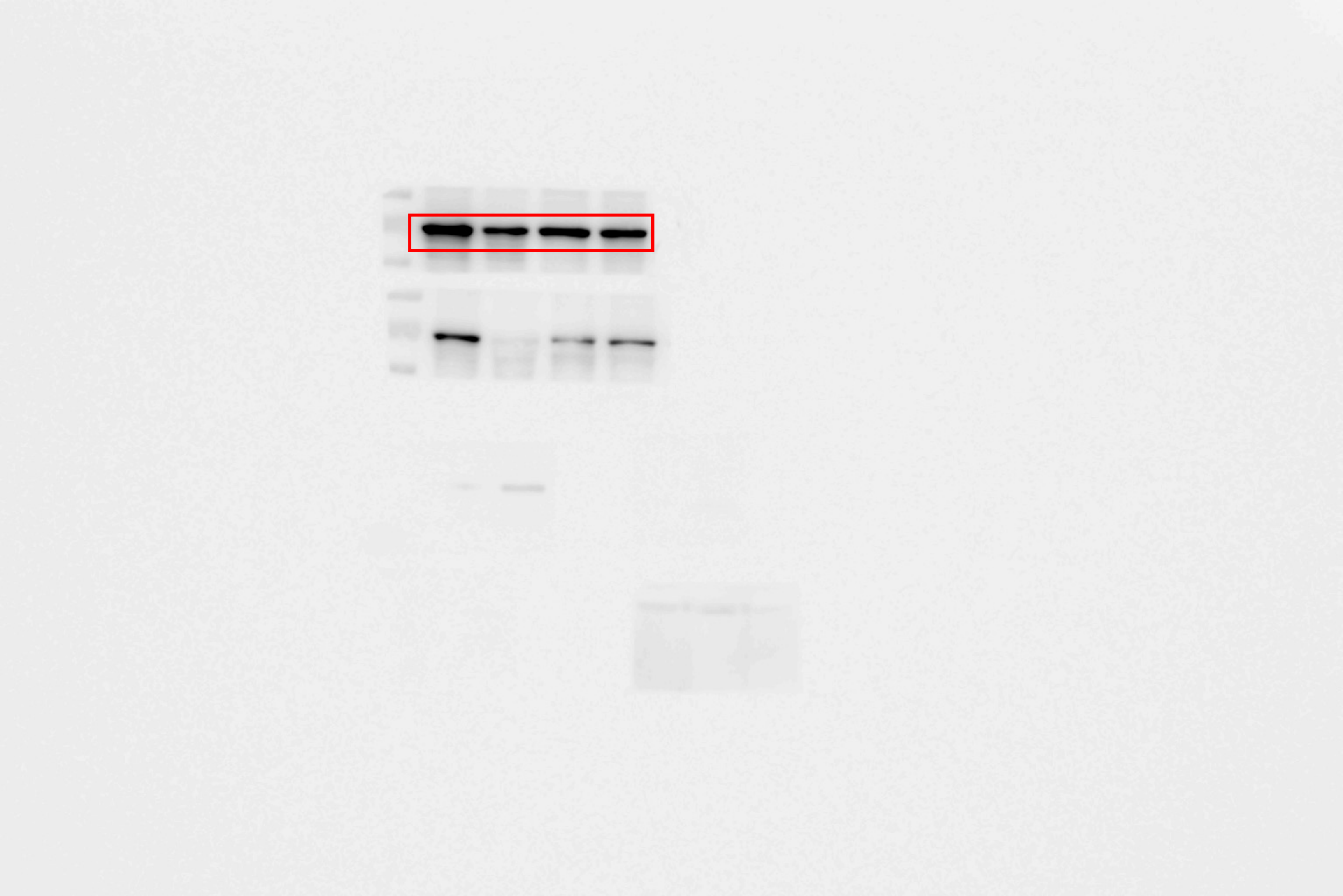

Full and uncropped western blots

Figure 2A

N87- P- NFKB

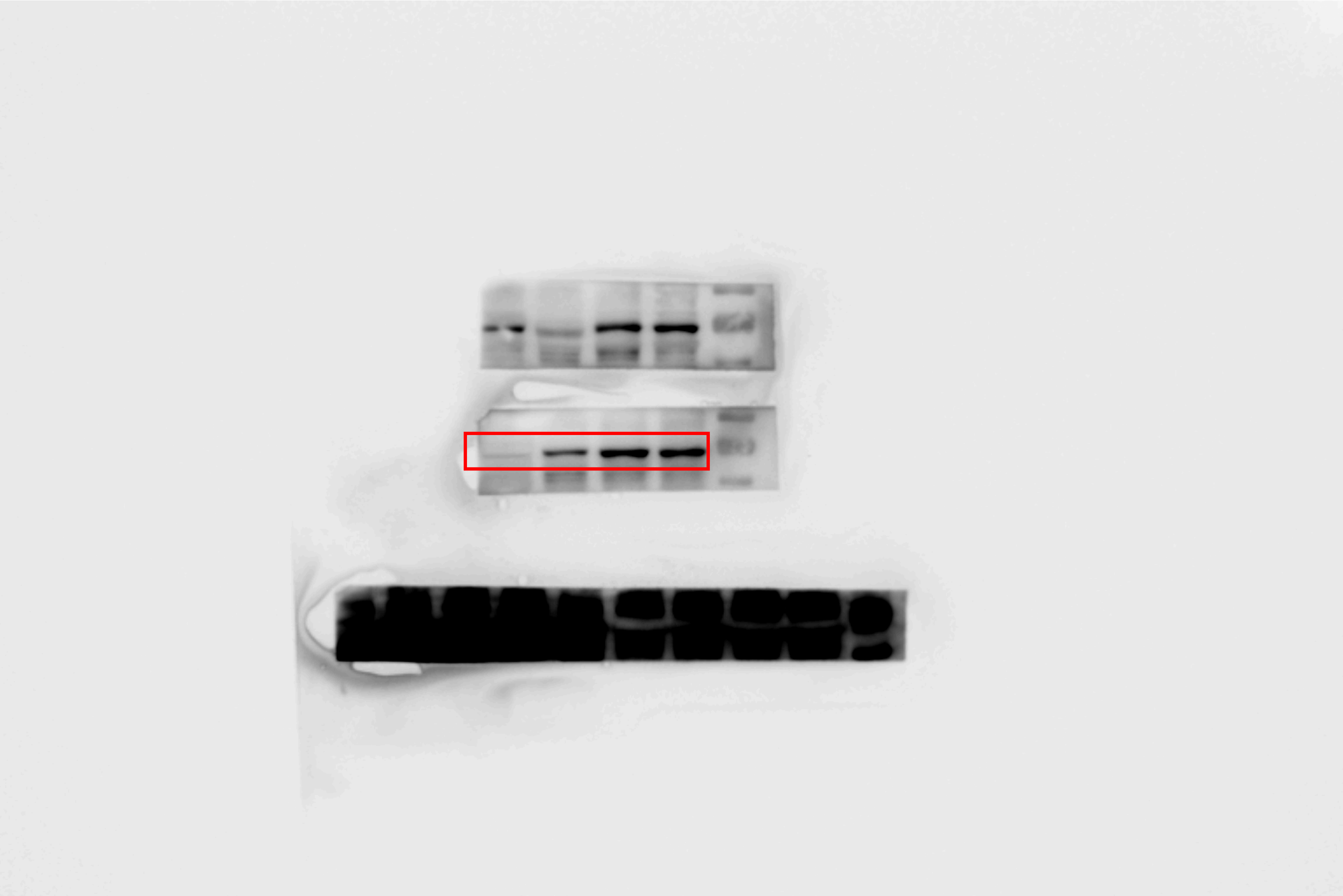

Full and uncropped western blots

Figure 2A

N87- GAPDH

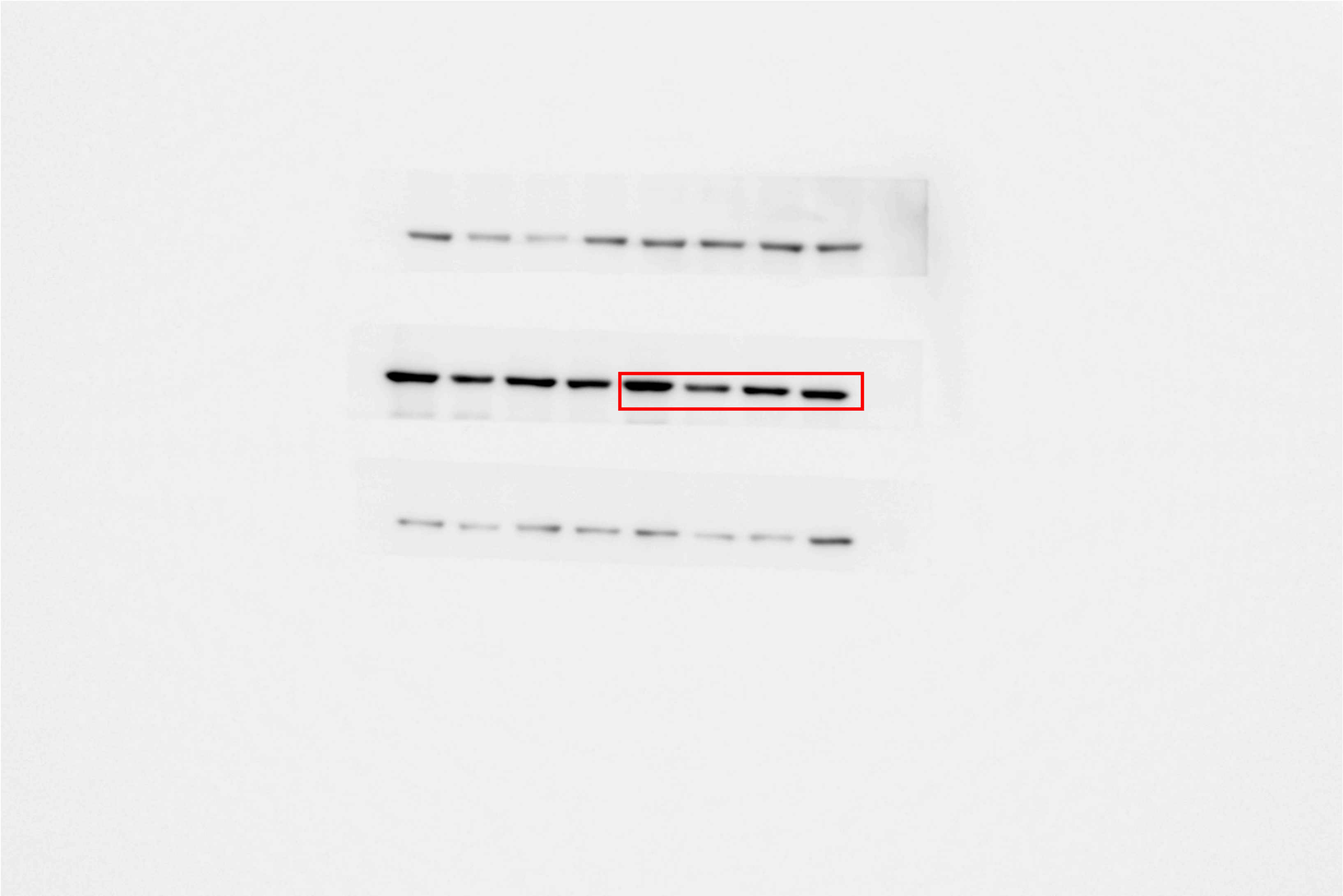

Full and uncropped western blots

Figure 2C

WB WITH BAND SIZE

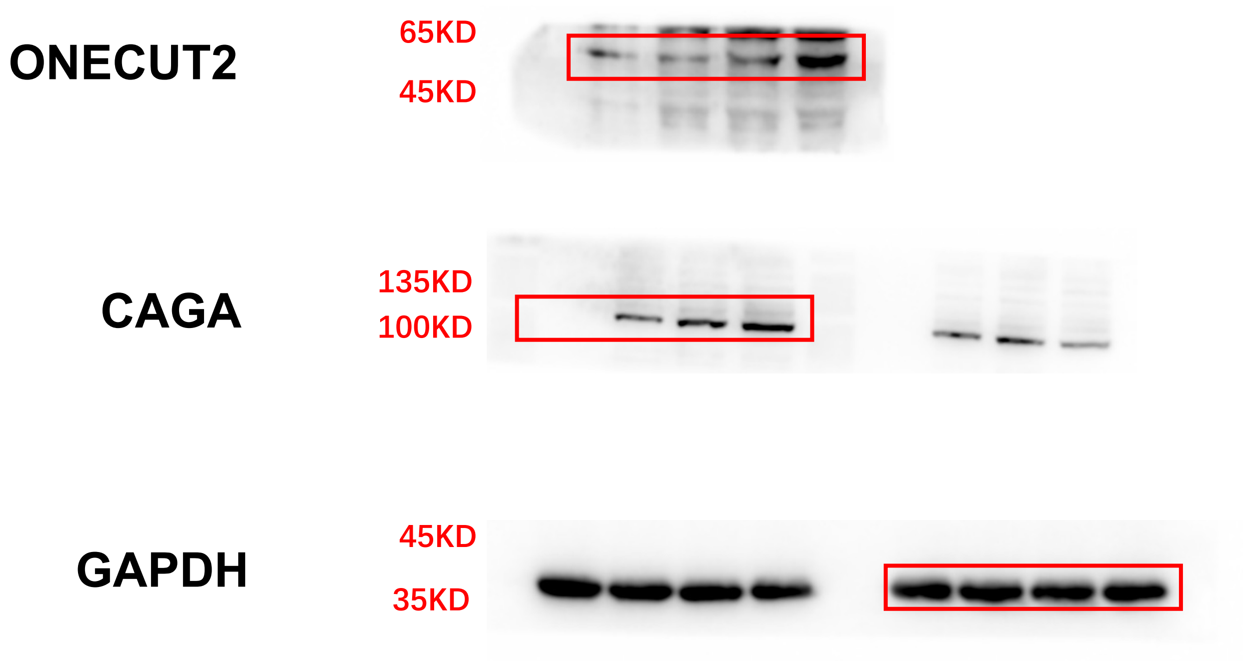

Figure 2C

ONECUT2

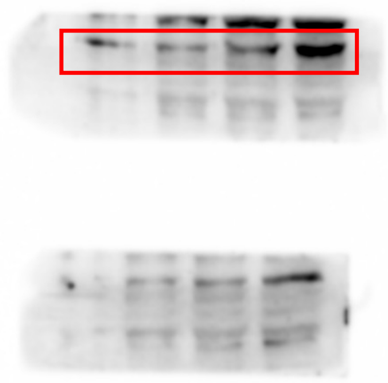

Full and uncropped western blots

Figure 2C

CAGA

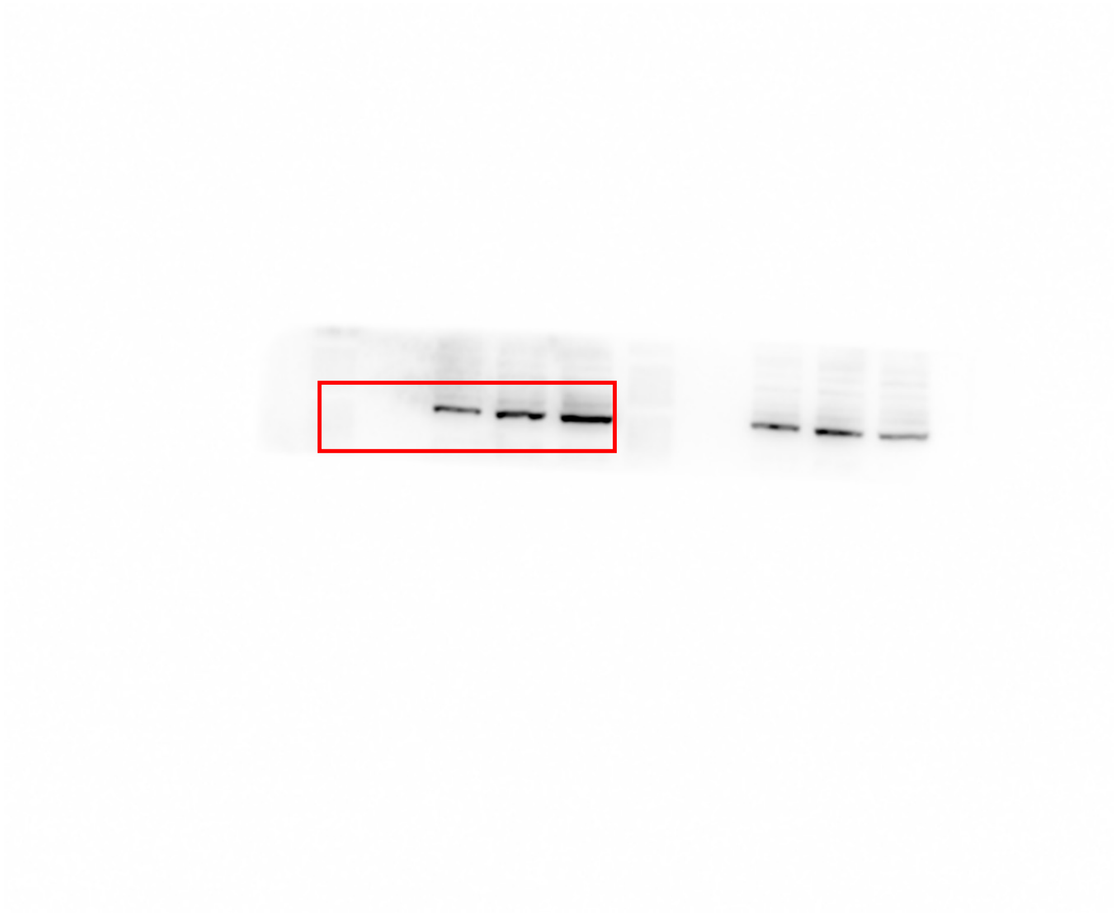

Full and uncropped western blots

Figure 2C

GAPDH

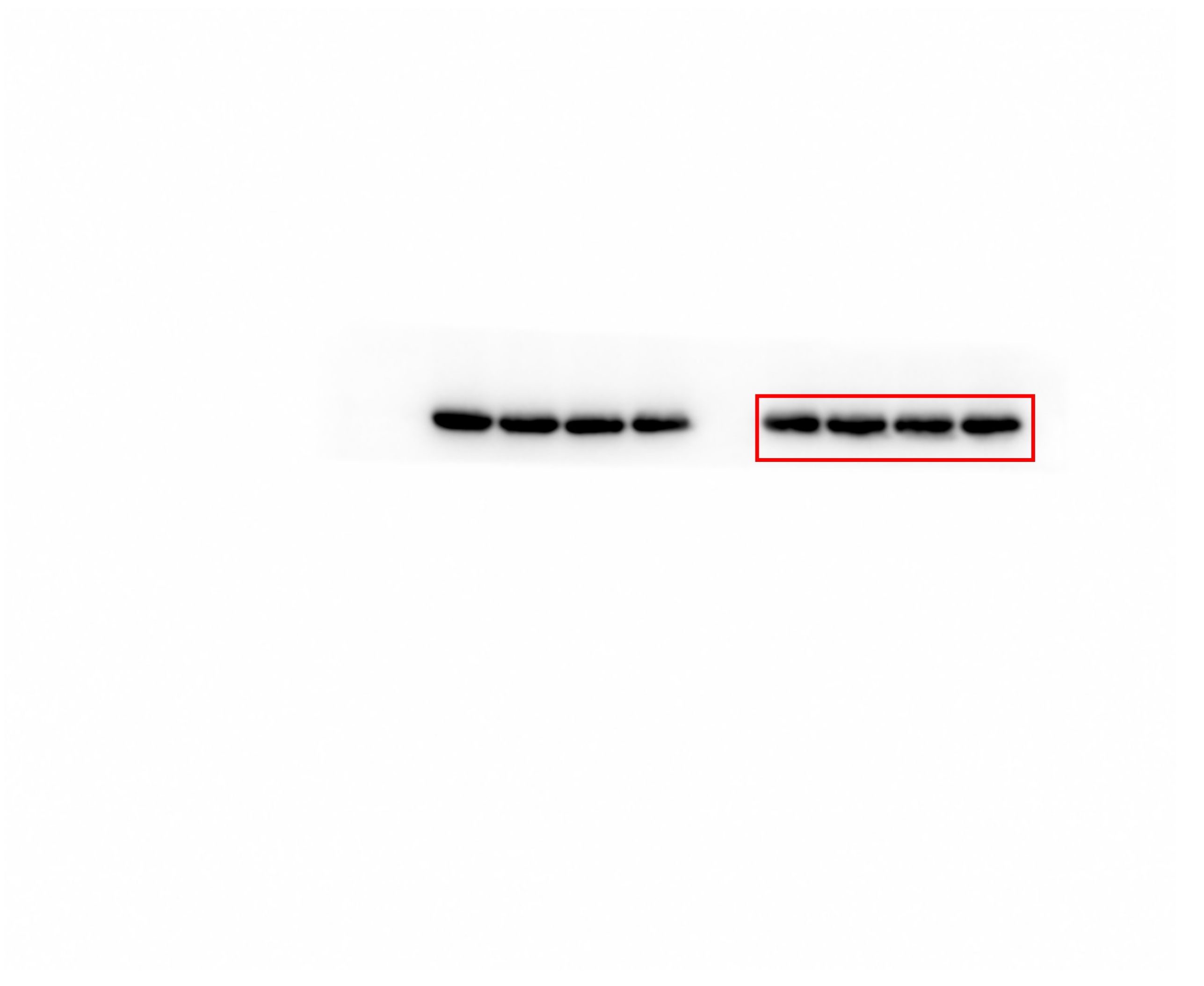

Full and uncropped western blots

Figure 3A

WB WITH BAND SIZE

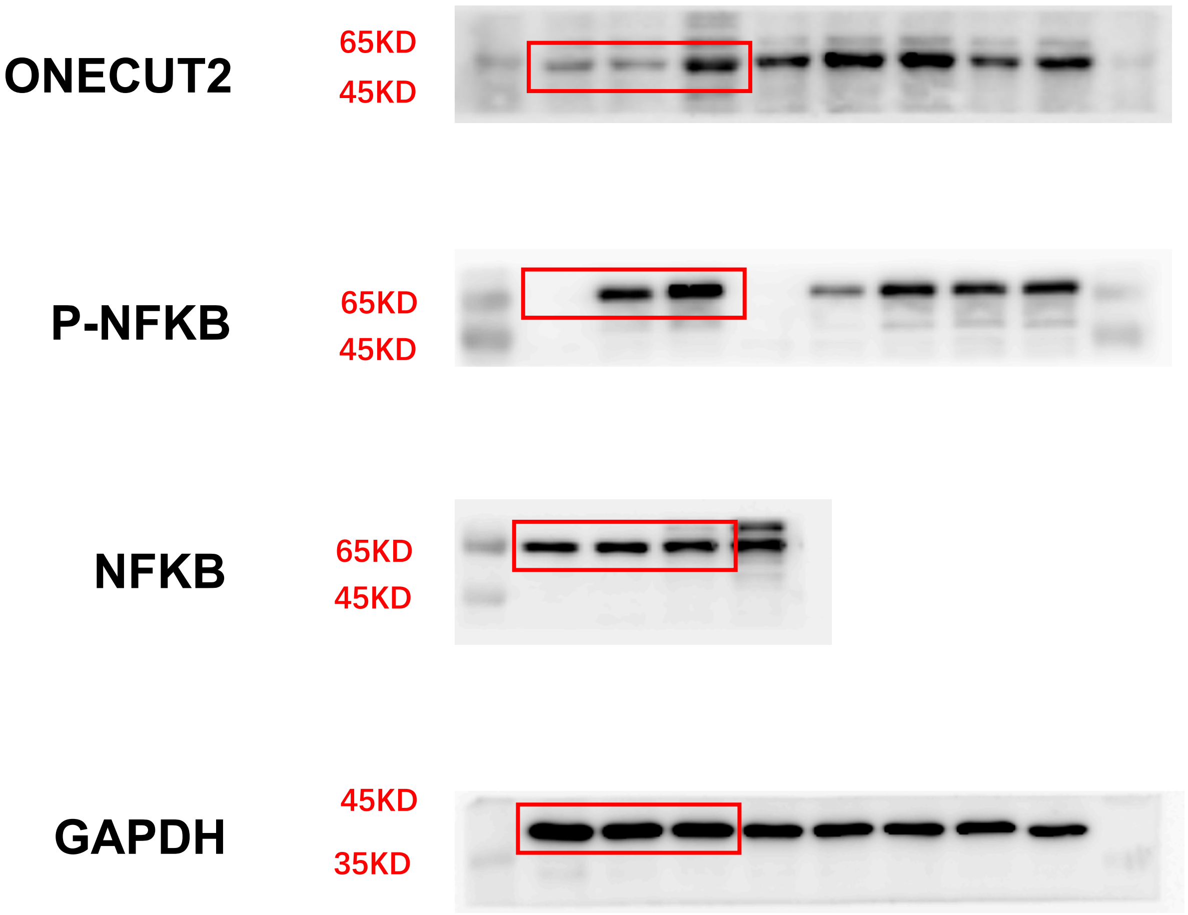

Figure 3A

AGS- ONECUT2

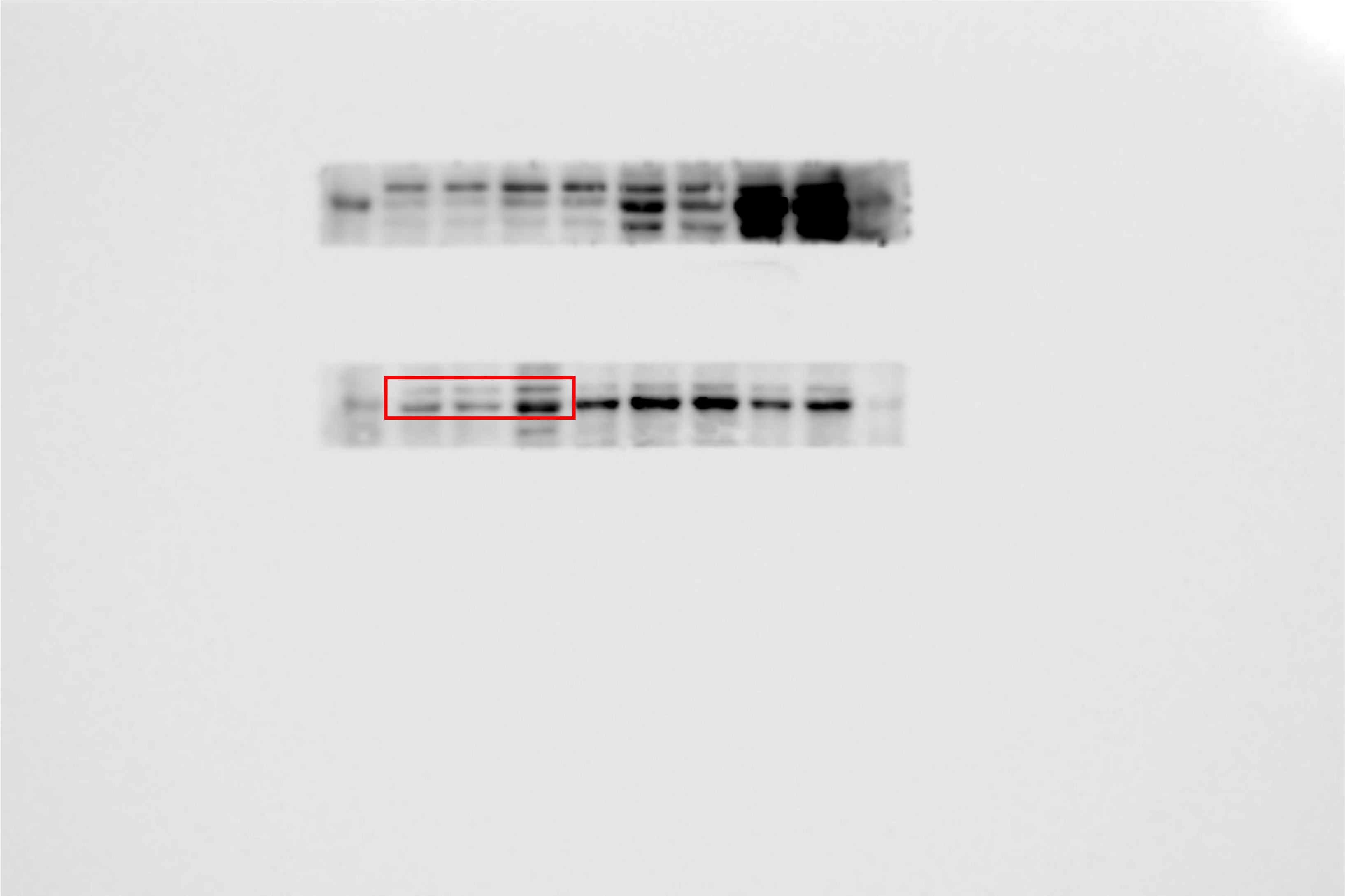

Full and uncropped western blots

Figure 3A

AGS- P-NFKB

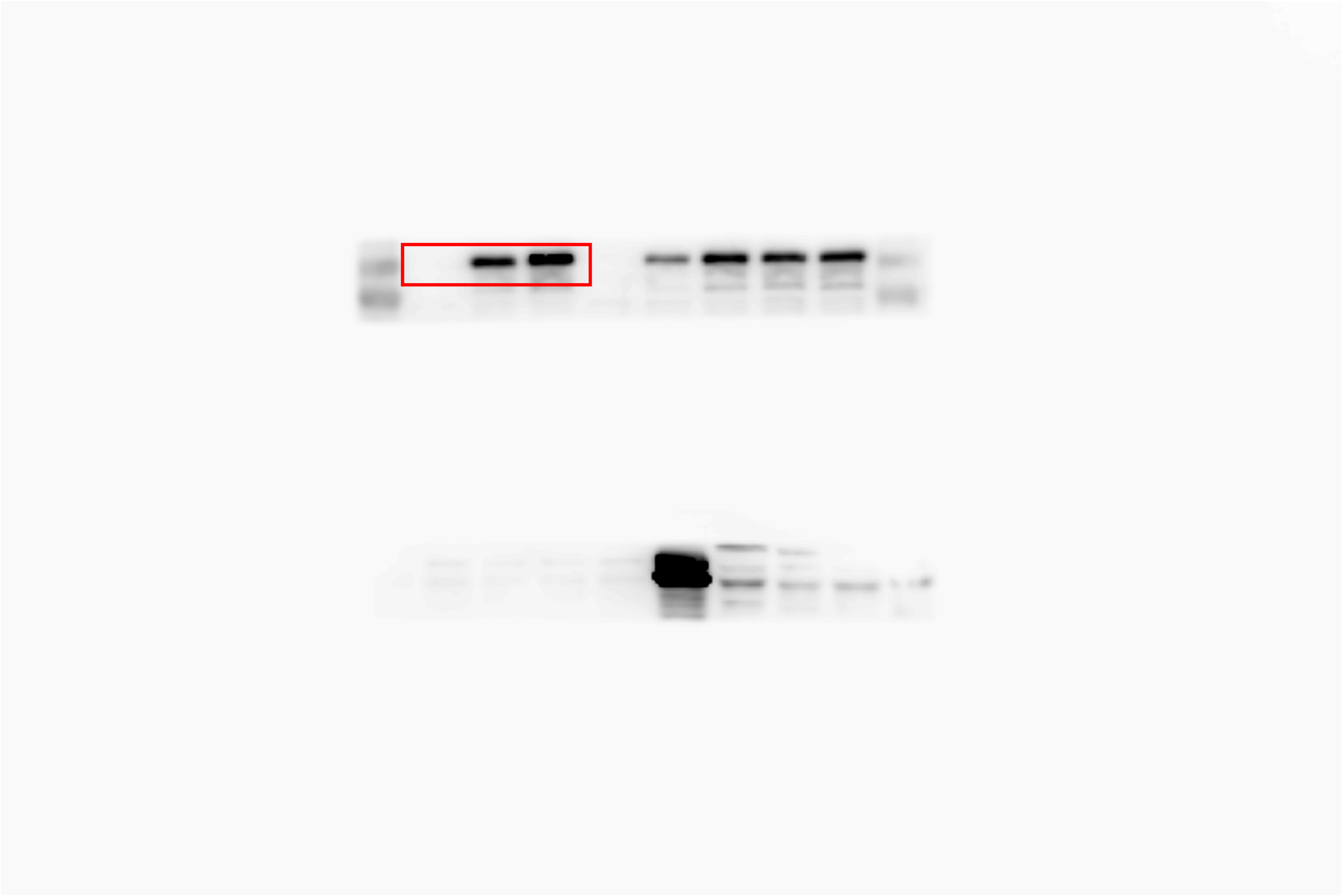

Full and uncropped western blots

Figure 3A

AGS- NFKB

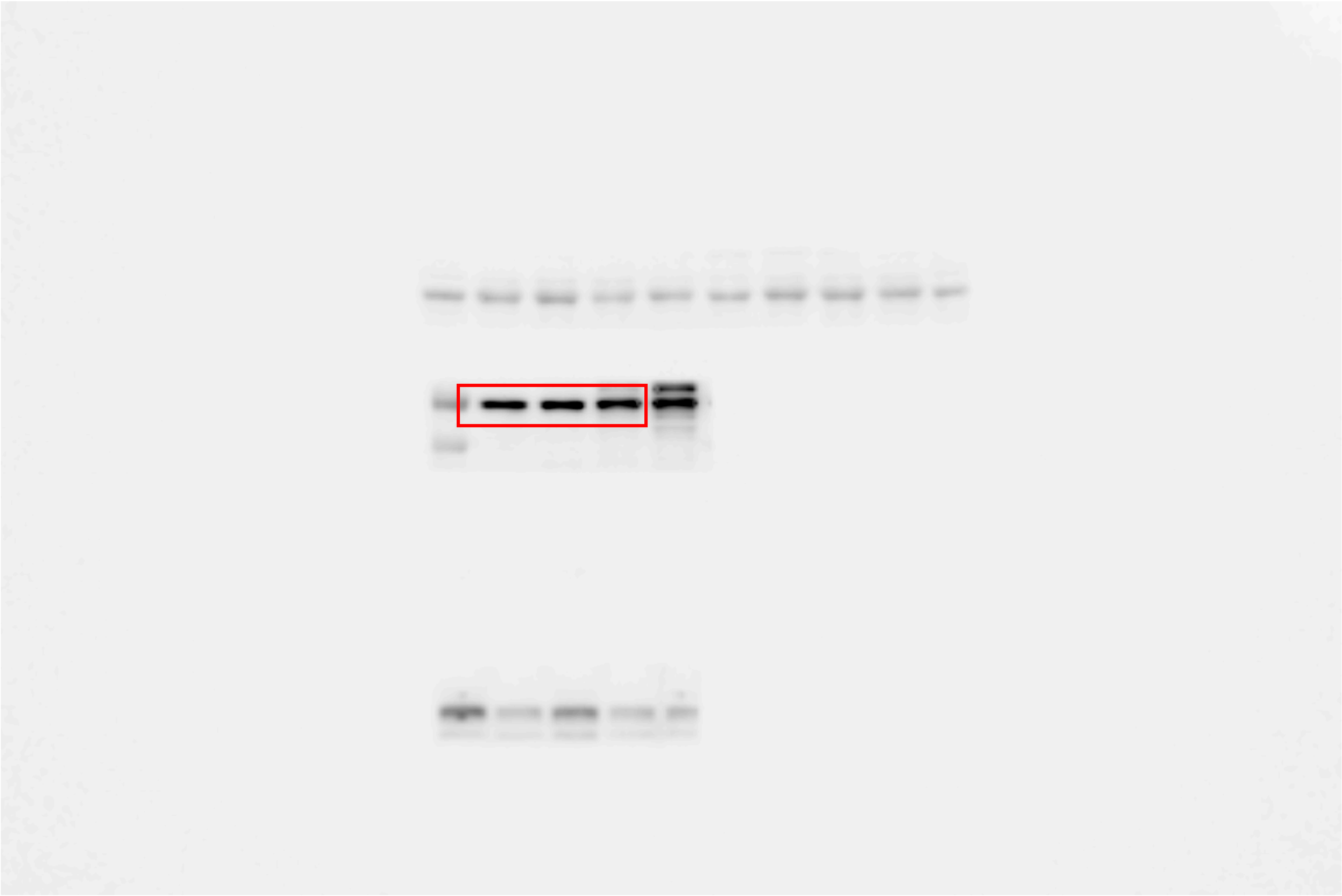

Full and uncropped western blots

Figure 3A

AGS- GAPDH

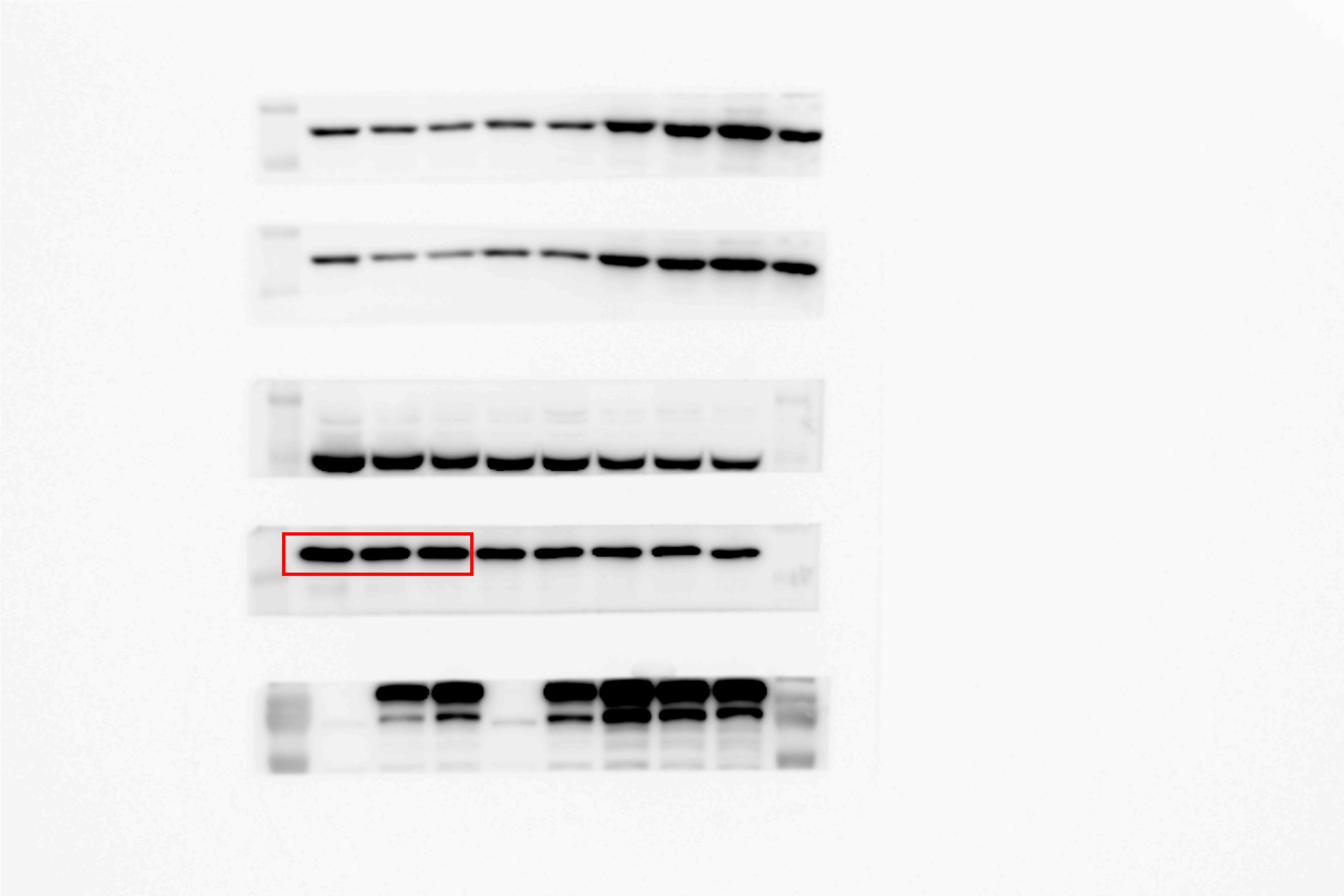

Full and uncropped western blots

Figure 3B

WB WITH BAND SIZE

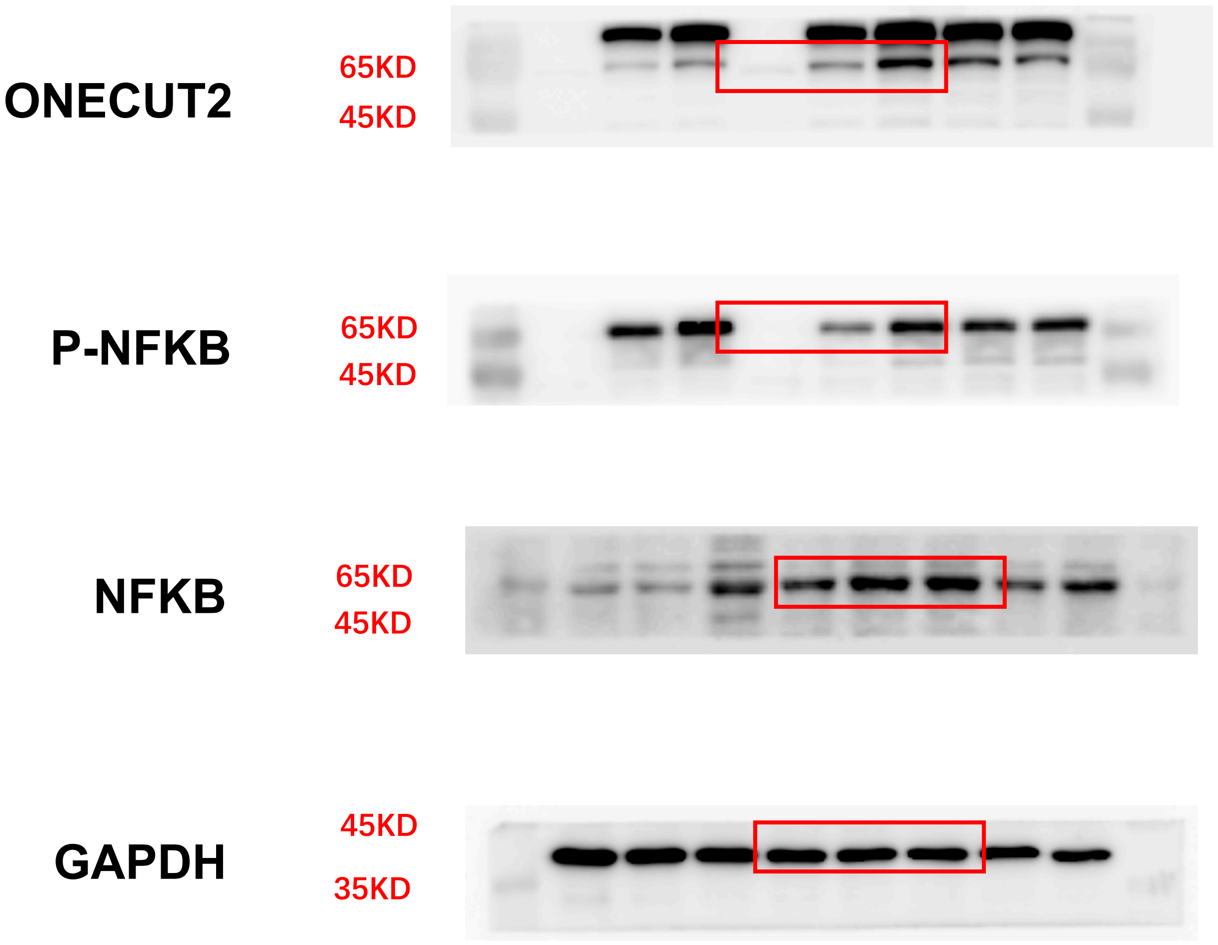

Figure 3B

N87- ONECUT2

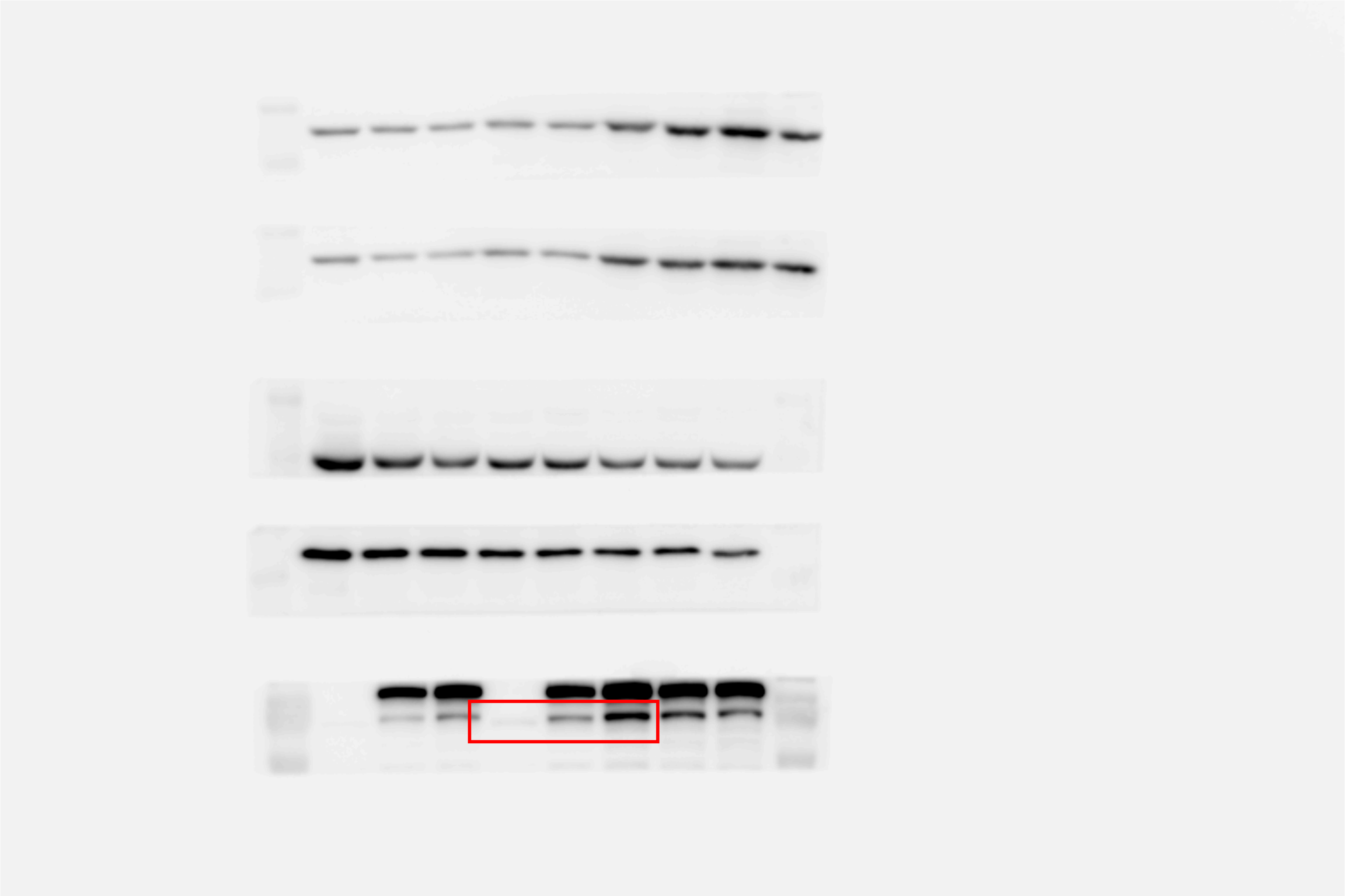

Full and uncropped western blots

Figure 3B

N87- P-NFKB

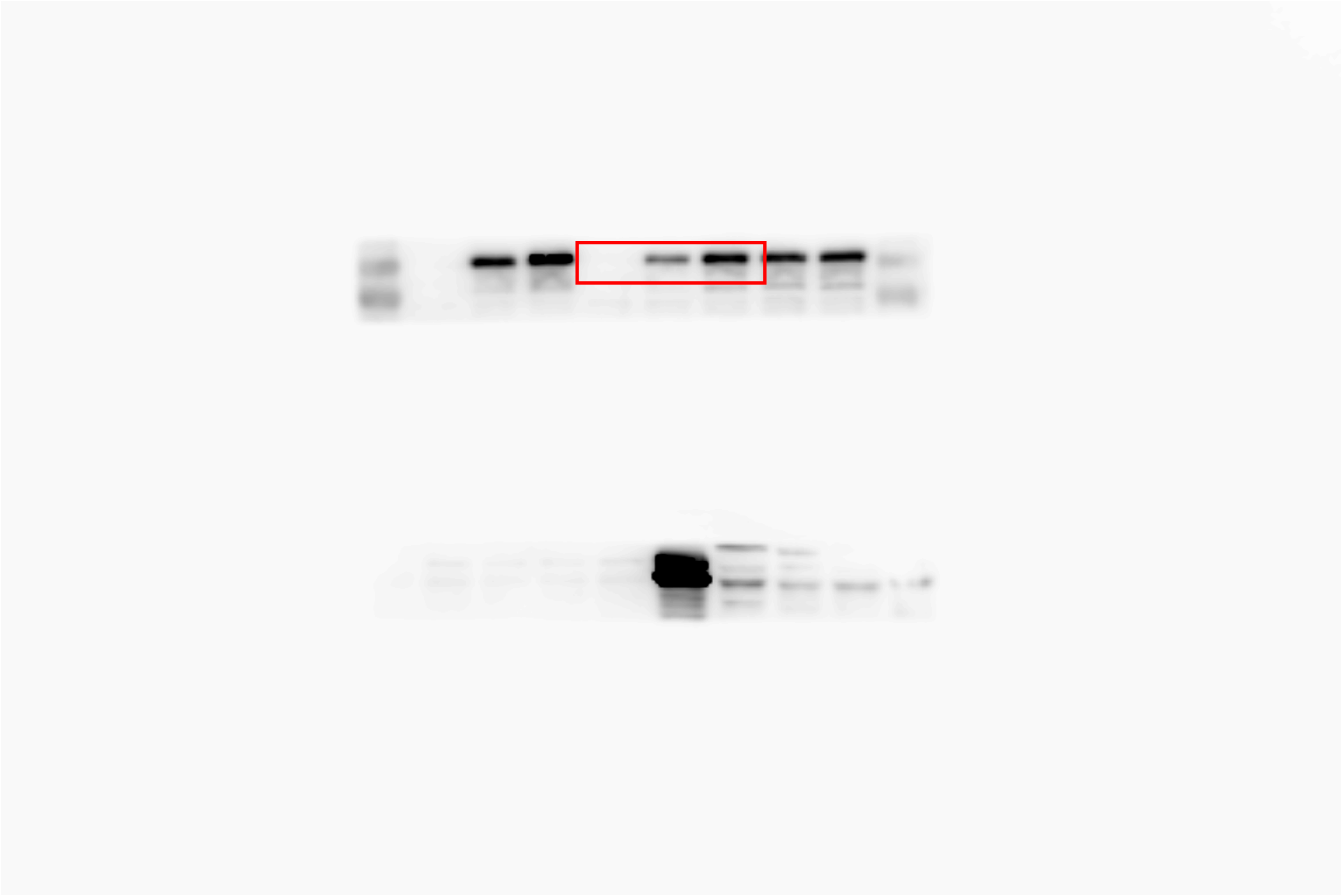

Full and uncropped western blots

Figure 3B

N87- NFKB

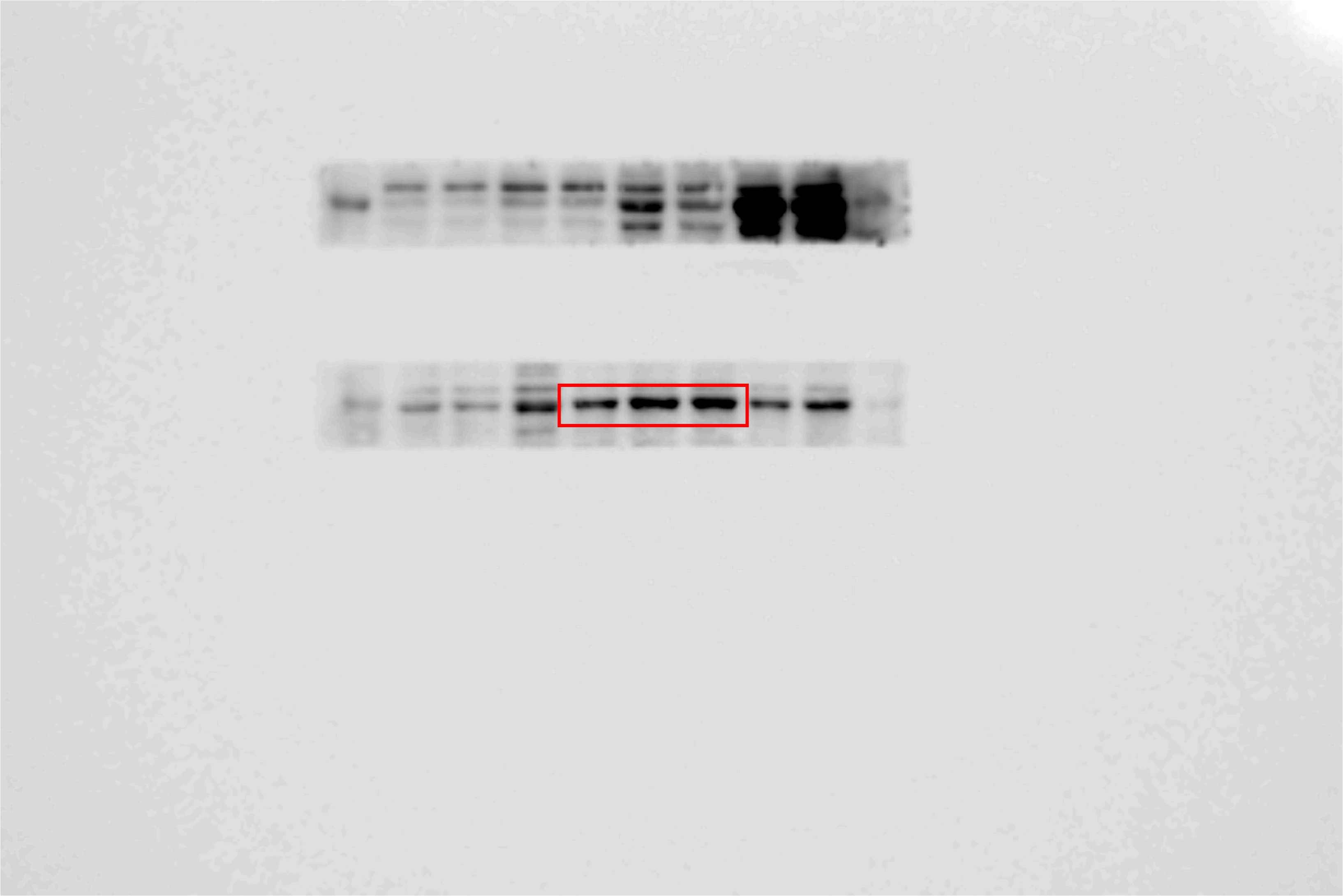

Full and uncropped western blots

Figure 3B

N87- GAPDH

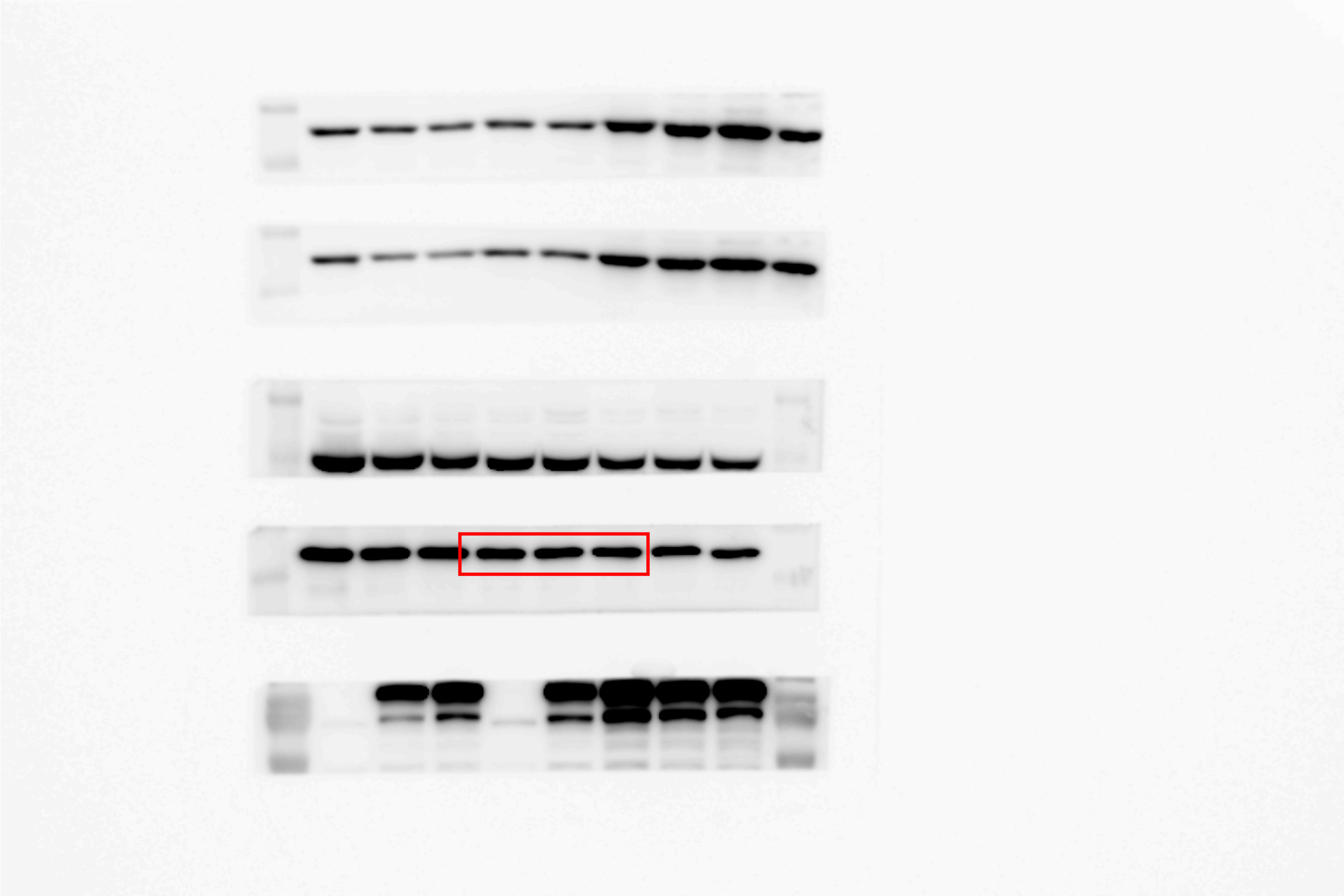

Full and uncropped western blots

Figure 3C

WB WITH BAND SIZE

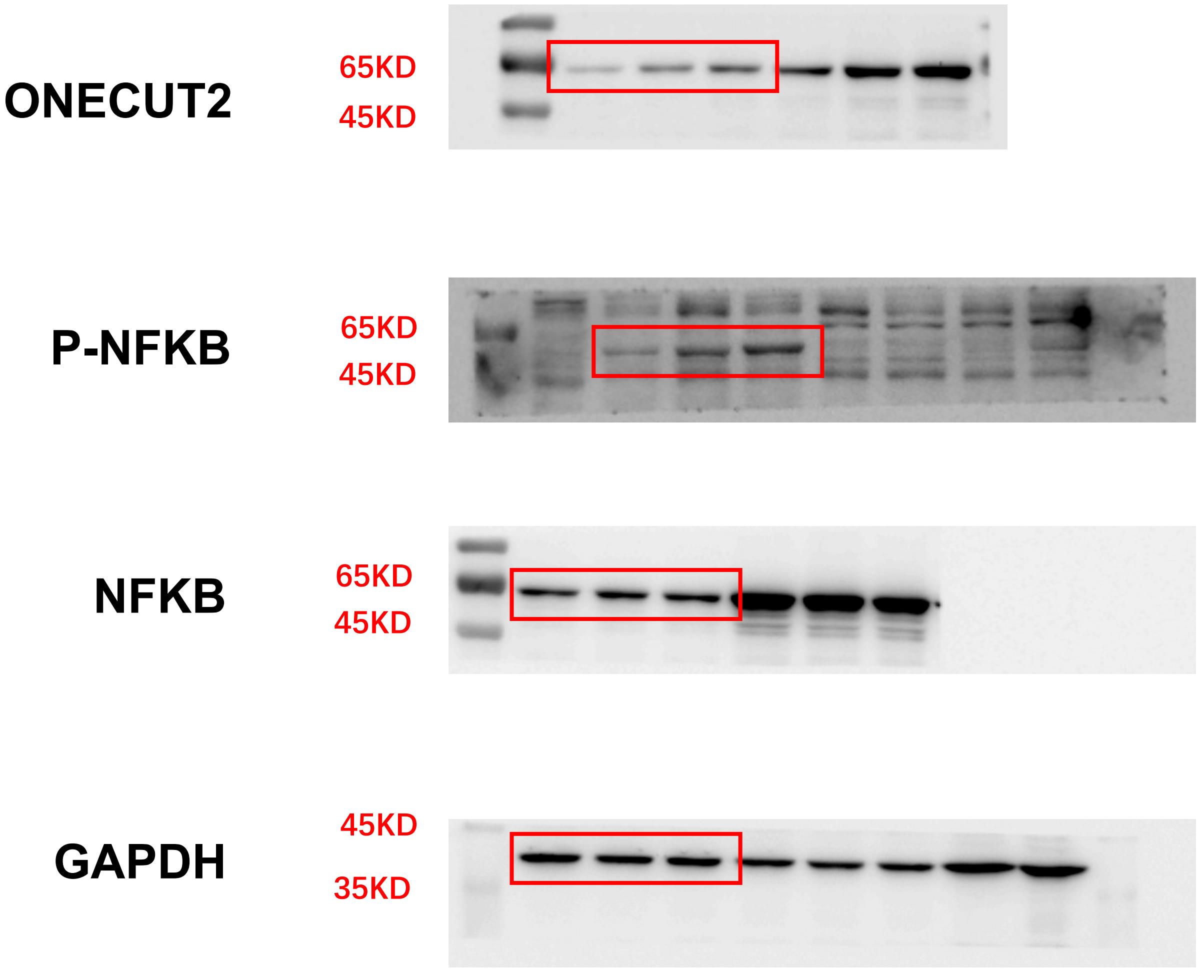

Figure 3C

AGS- ONECUT2

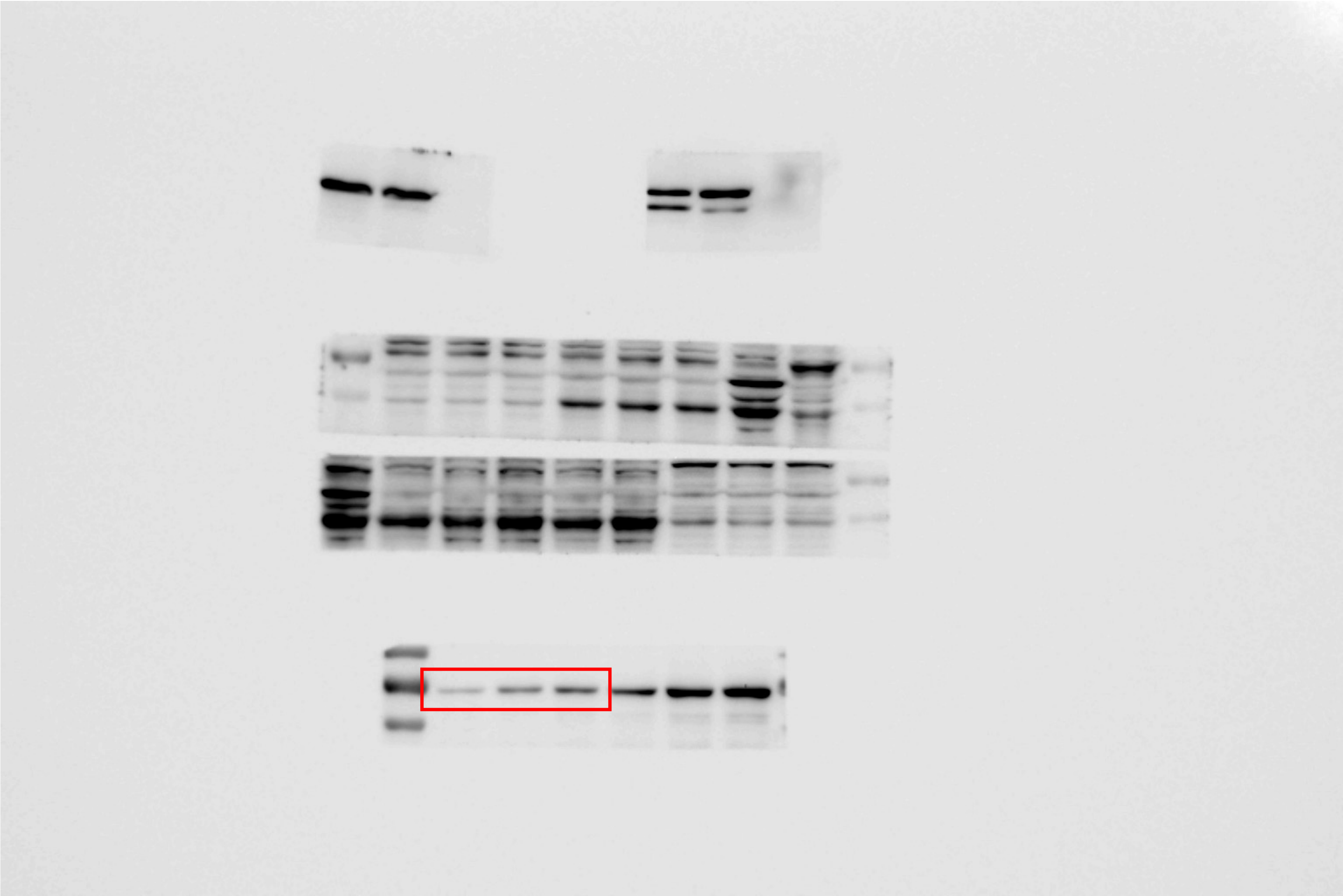

Full and uncropped western blots

Figure 3C

AGS- P-NFKB

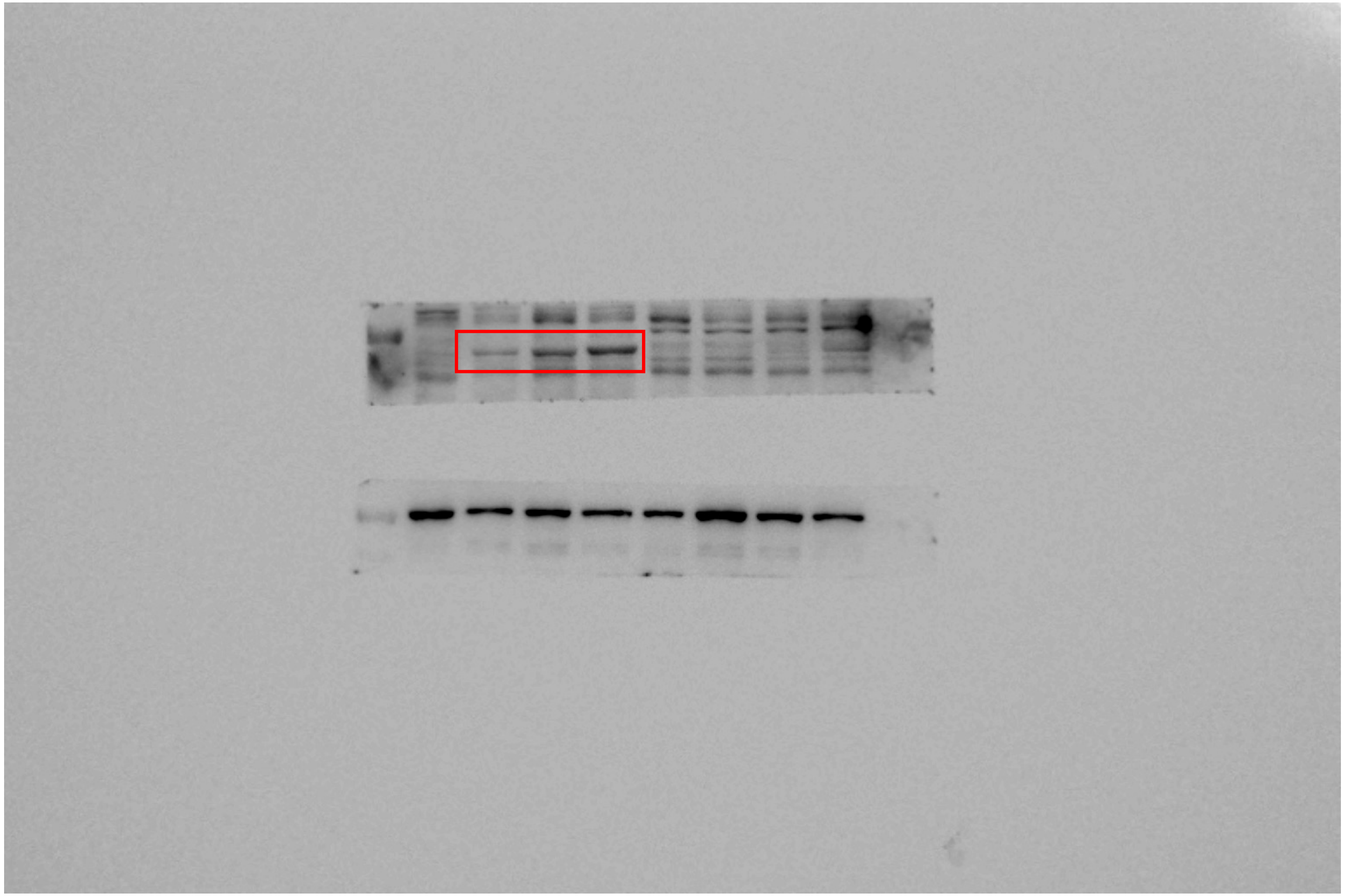

Full and uncropped western blots

Figure 3C

AGS- NFKB

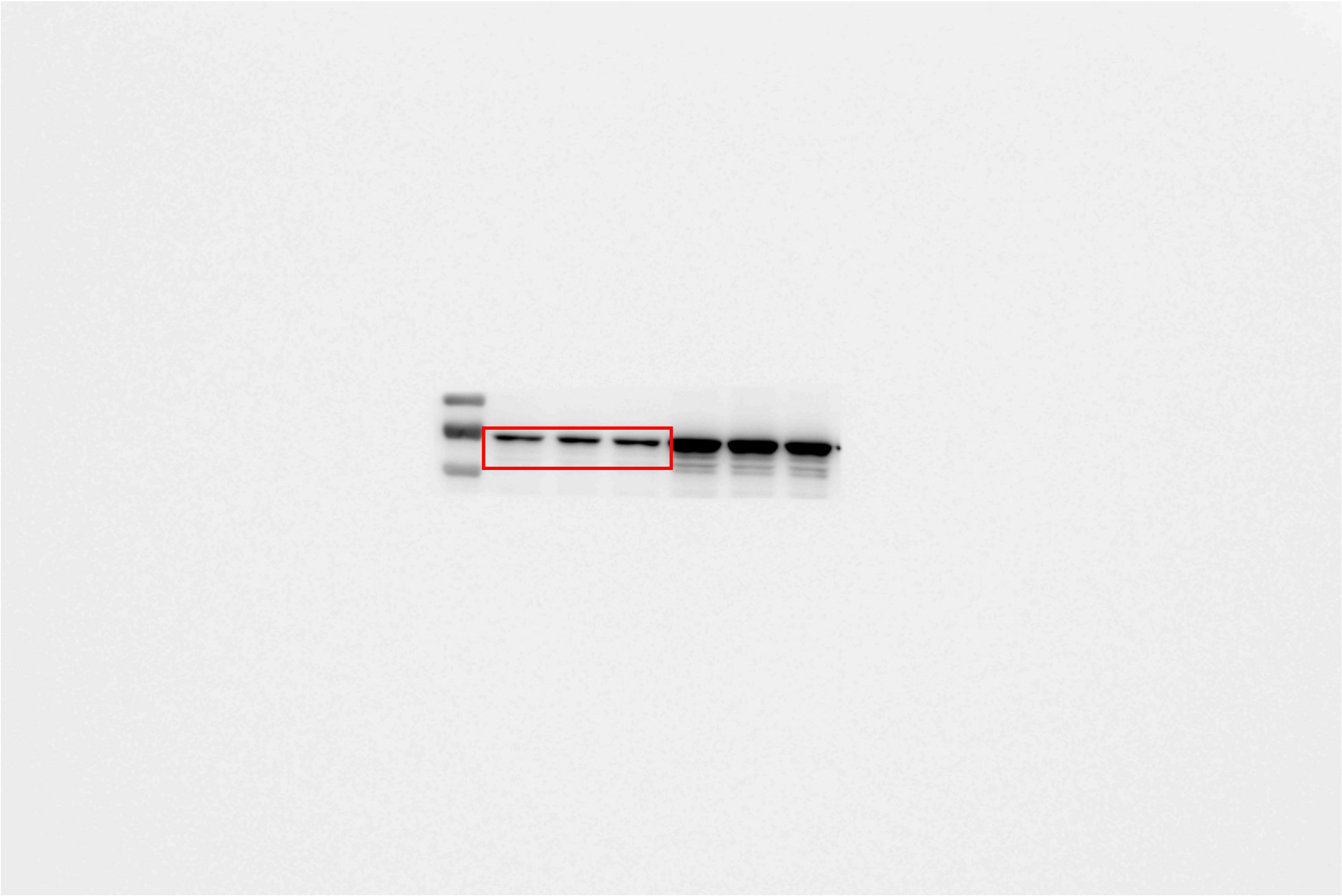

Full and uncropped western blots

Figure 3C

AGS- GAPDH

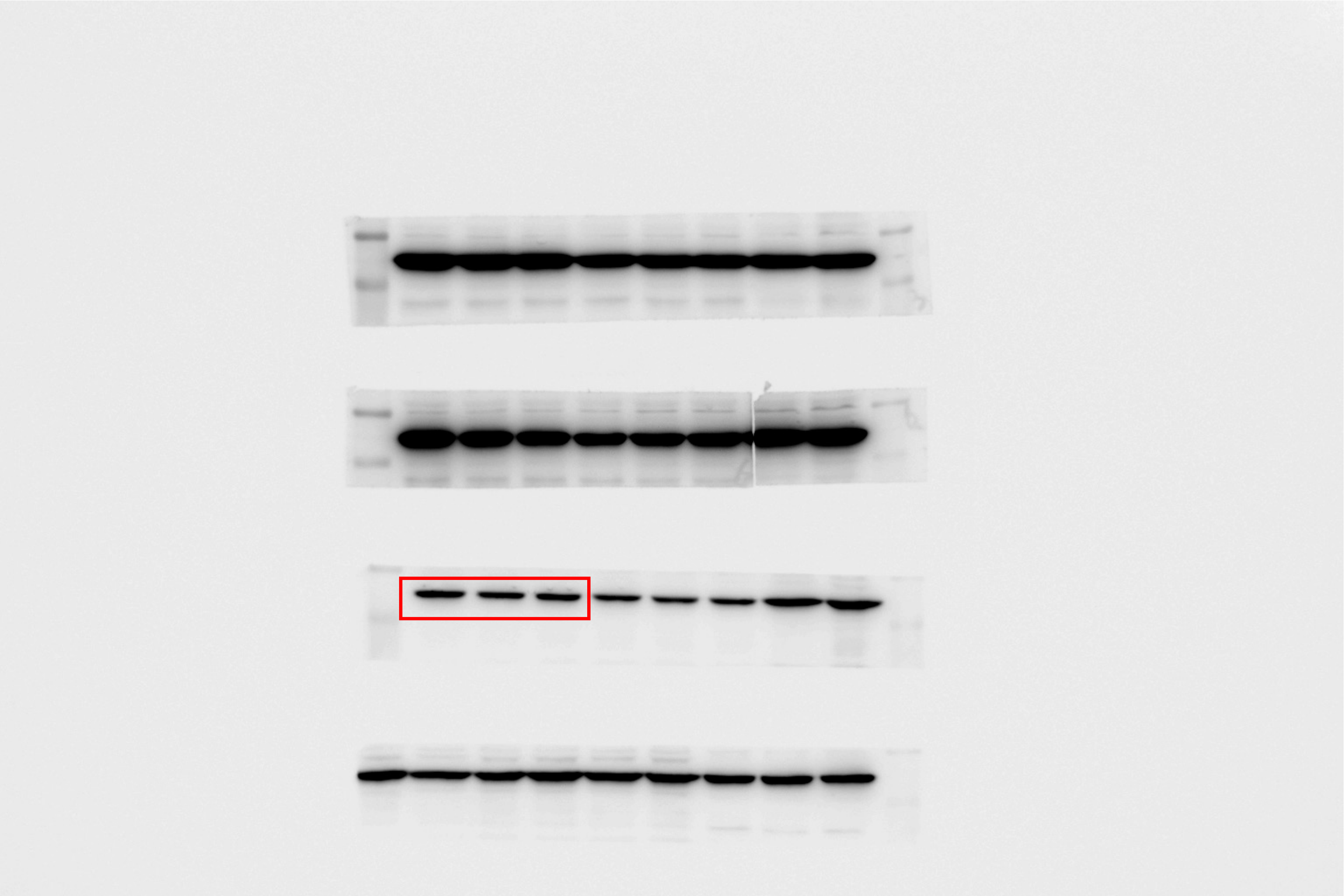

Full and uncropped western blots

Figure 3D

WB WITH BAND SIZE

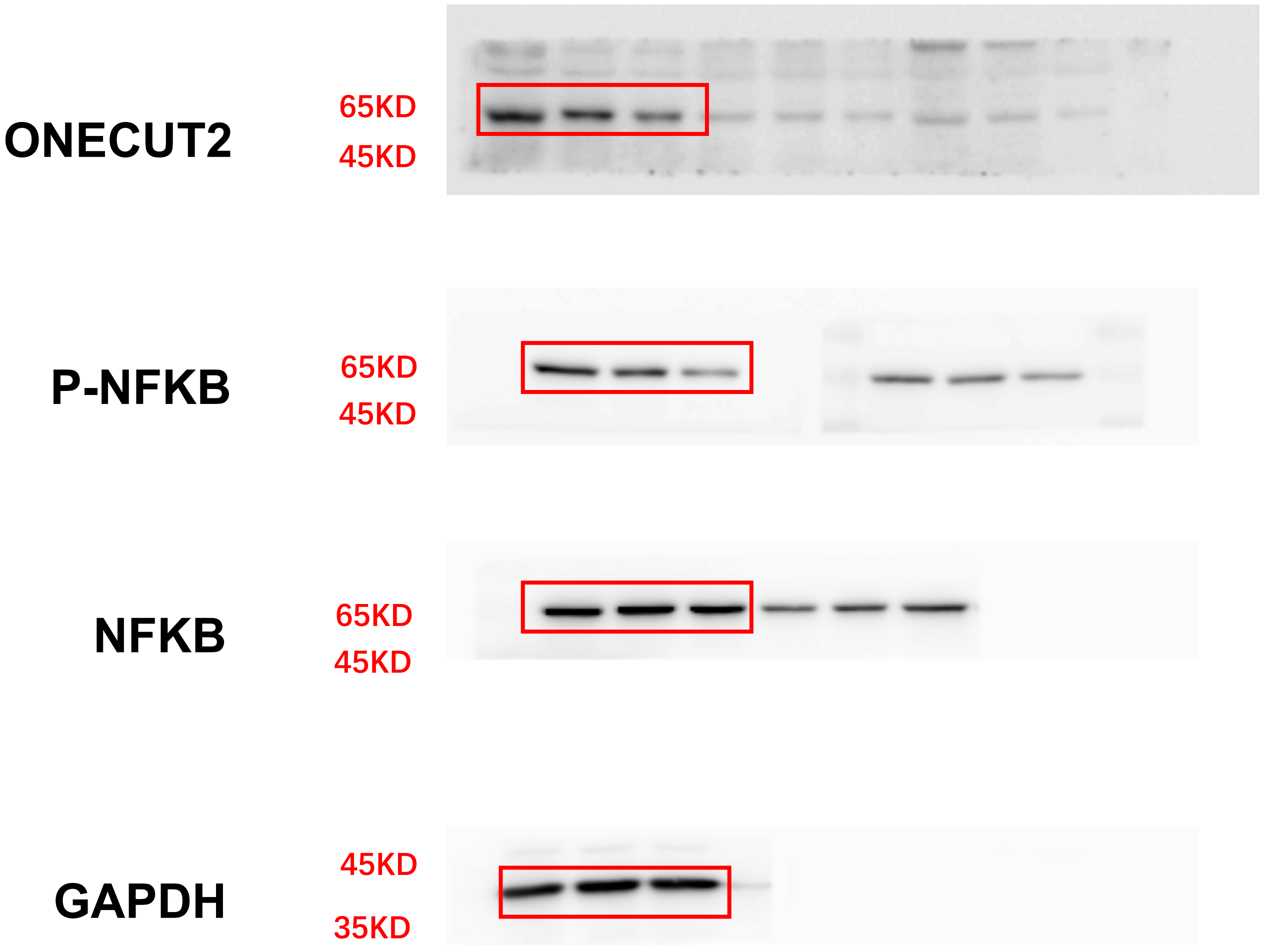

Figure 3D

N87- ONECUT2

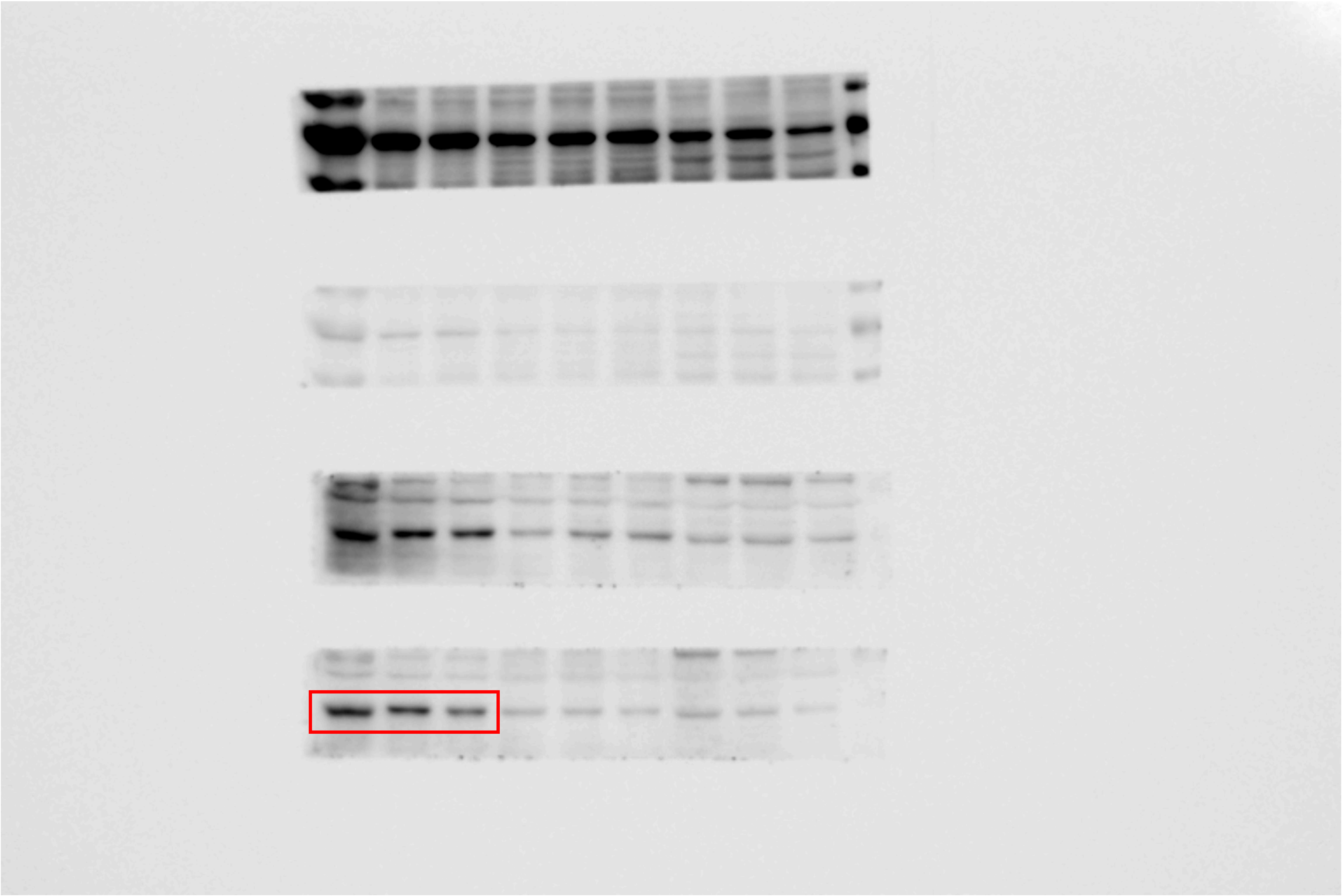

Full and uncropped western blots

Figure 3D

N87- P-NFKB

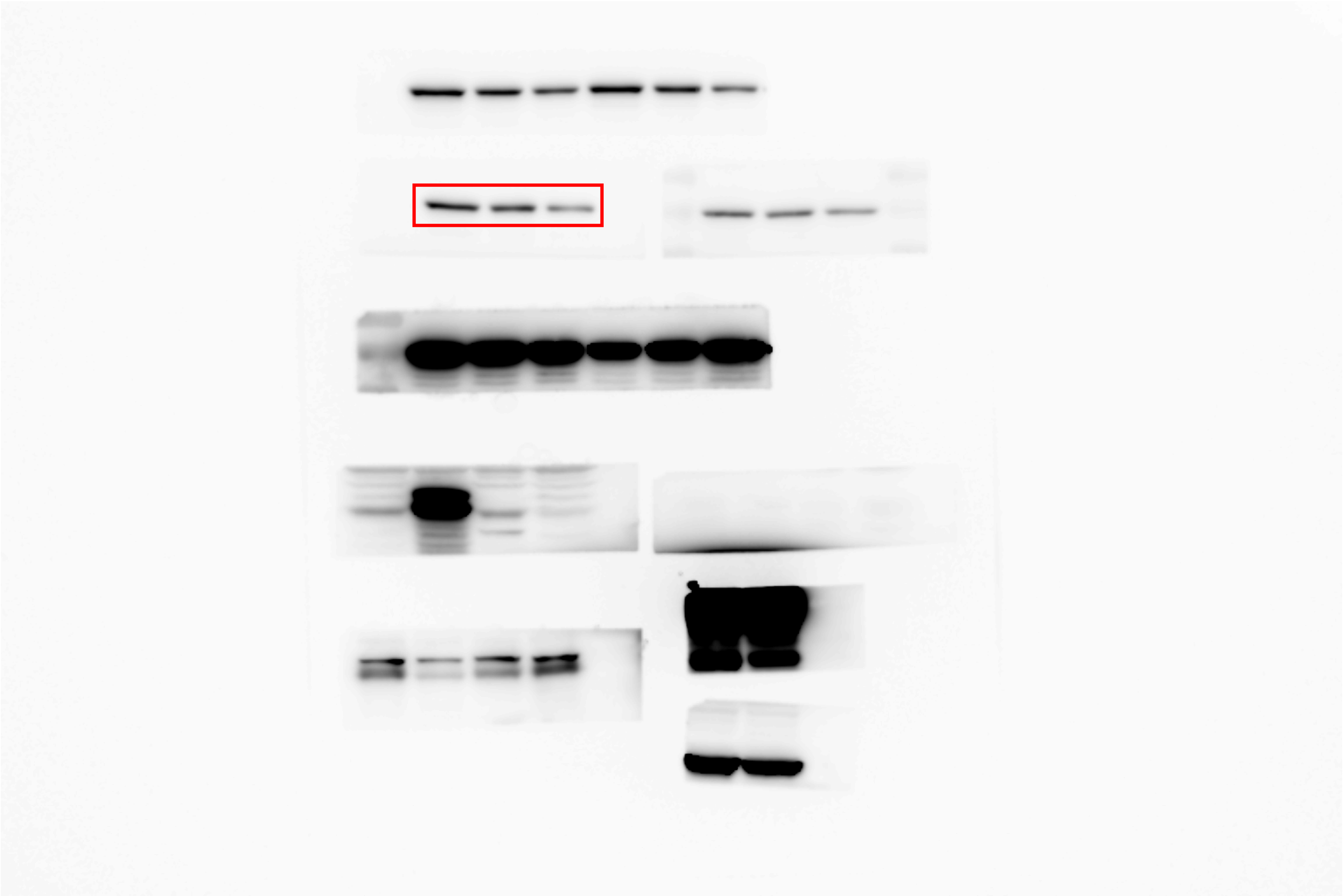

Full and uncropped western blots

Figure 3D

N87- NFKB

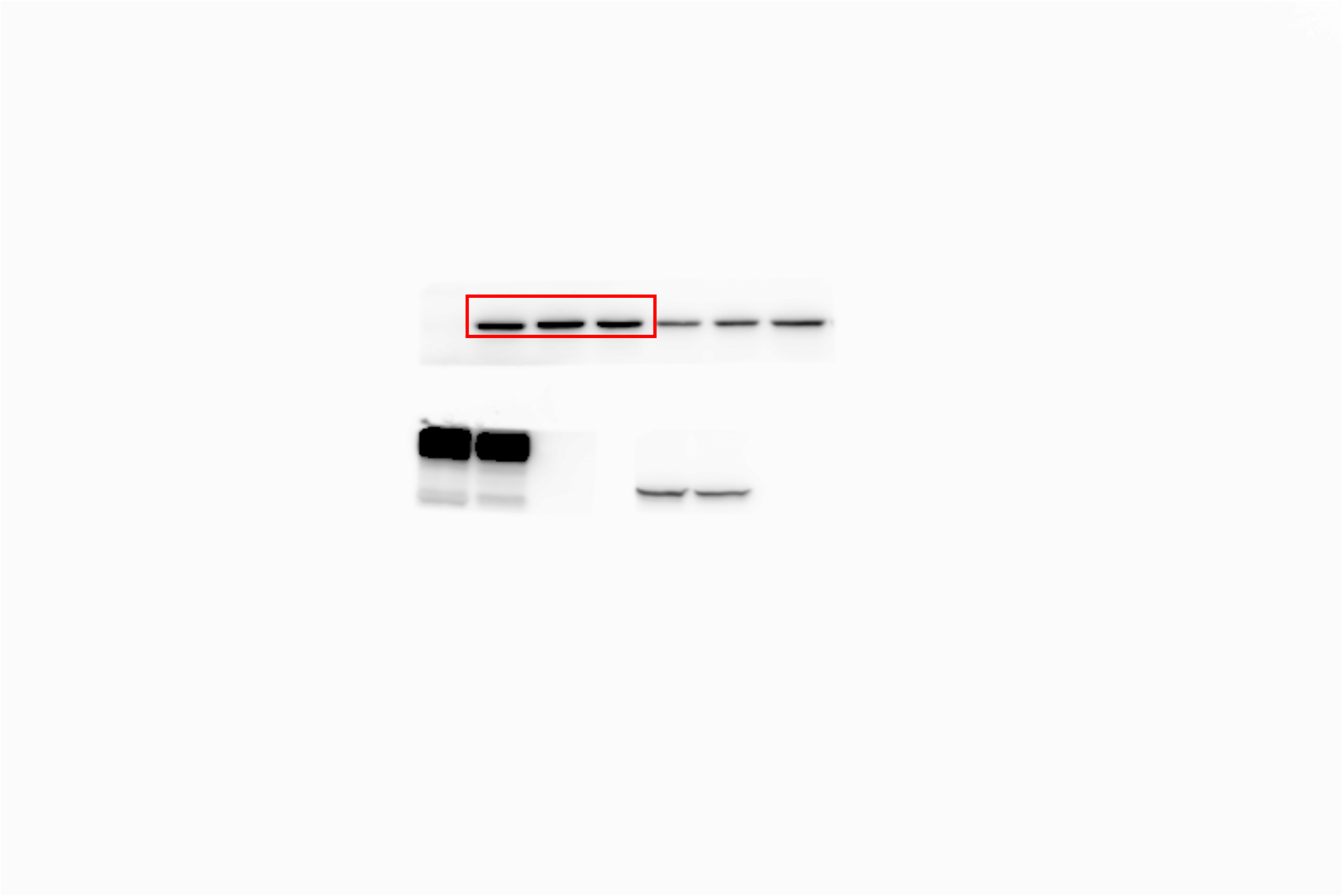

Full and uncropped western blots

Figure 3D

N87- GAPDH

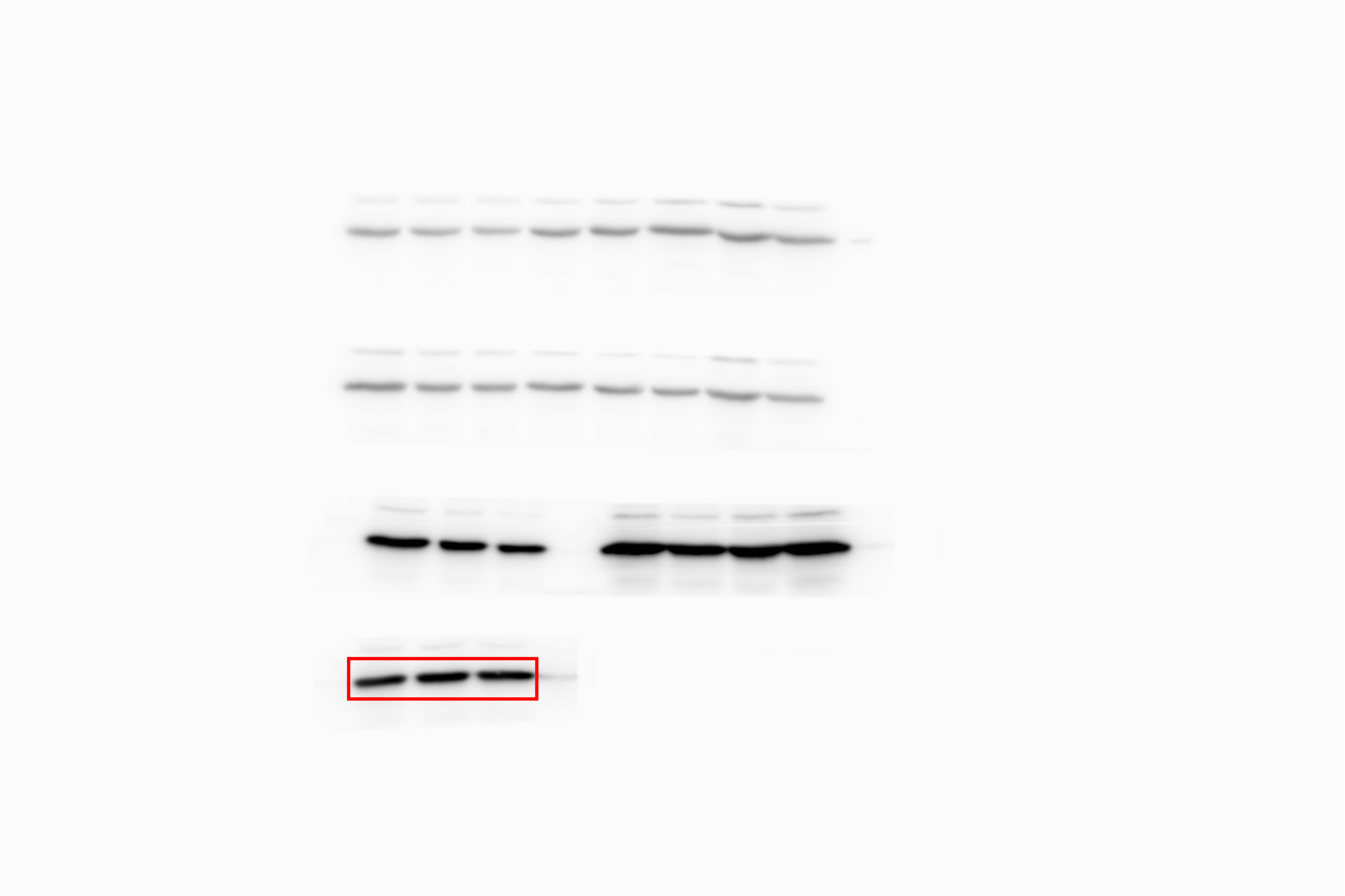

Full and uncropped western blots

Figure 4B

WB WITH BAND SIZE

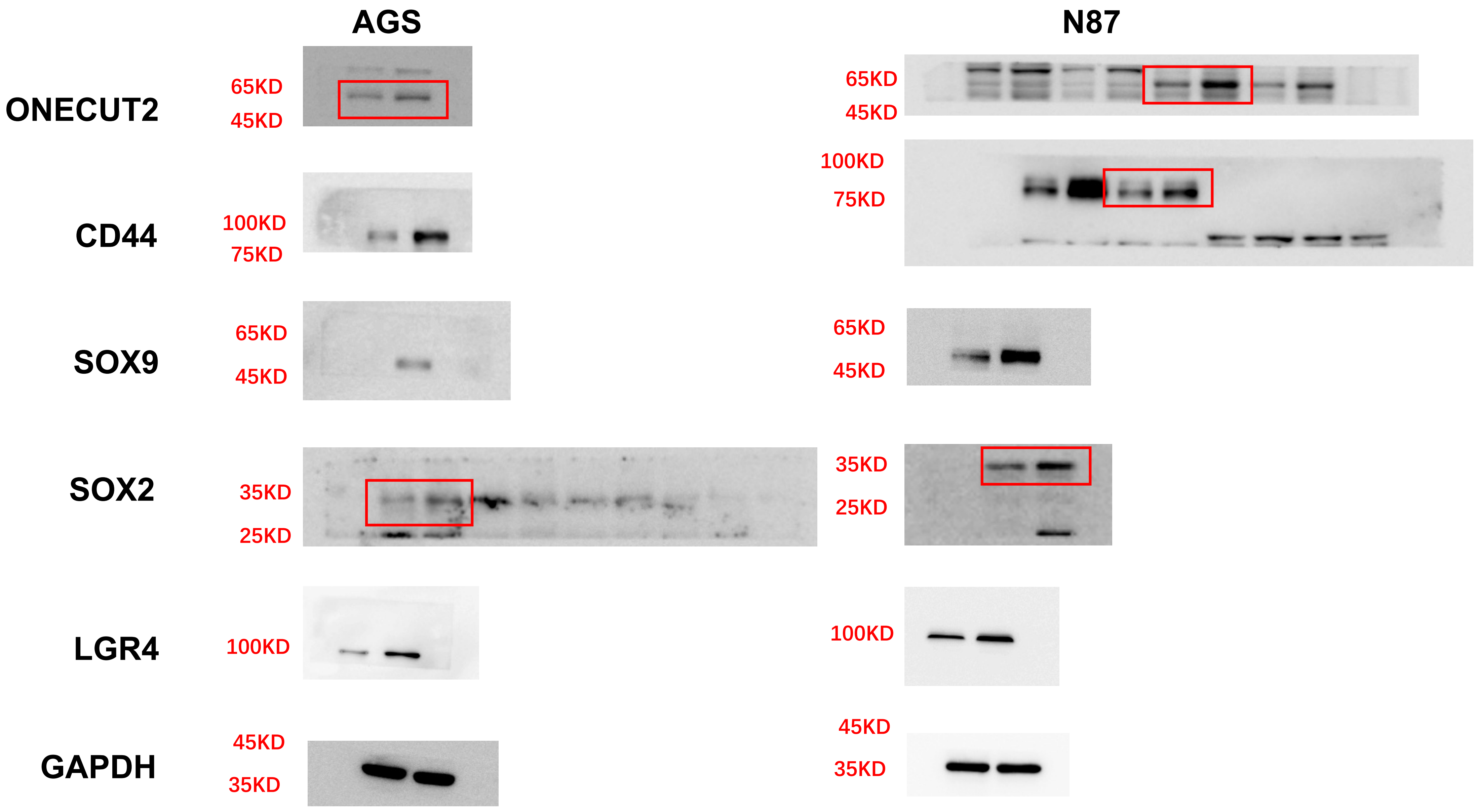

Figure 4B

AGS- ONECUT2

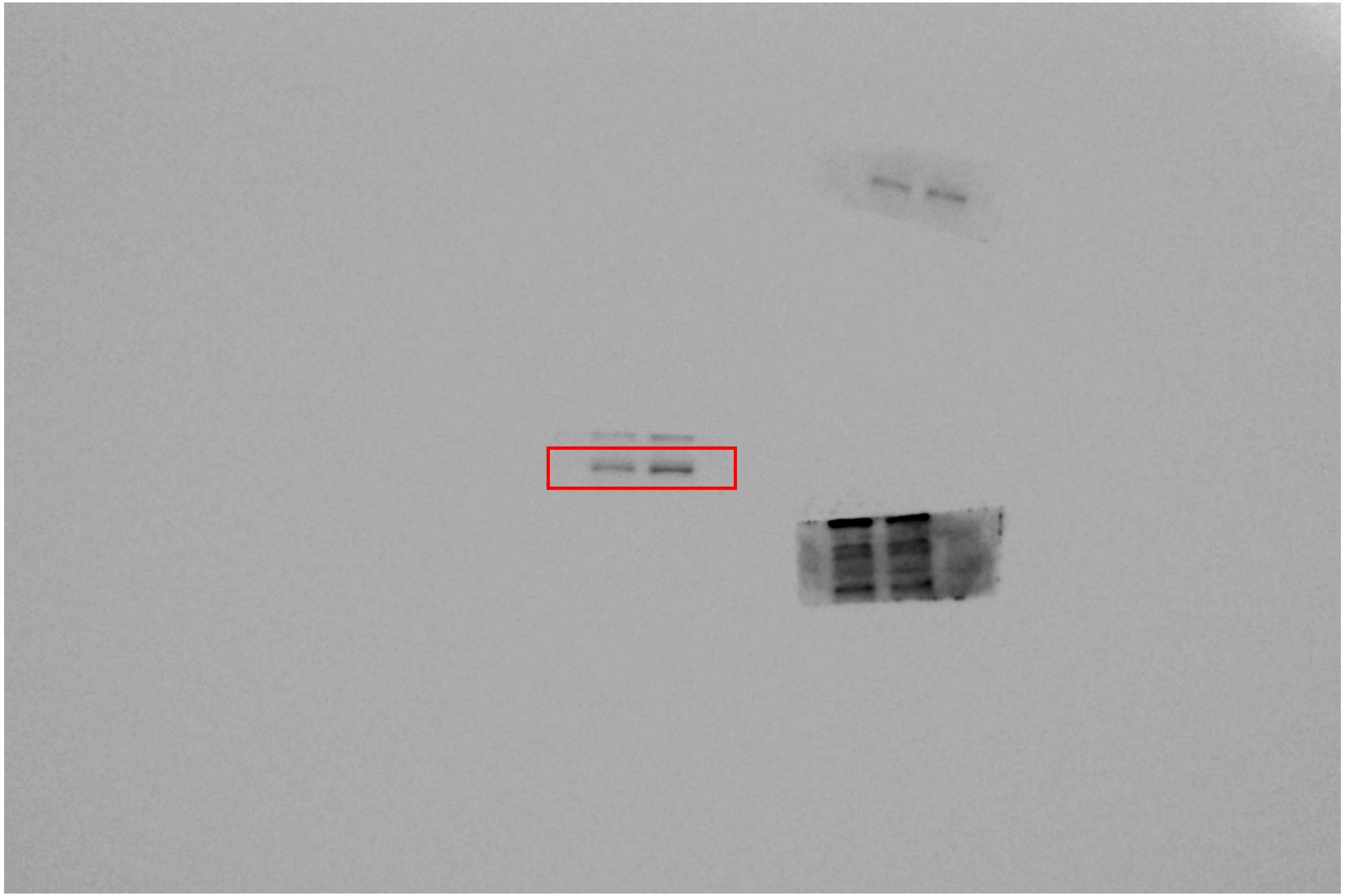

Full and uncropped western blots

Figure 4B

AGS- CD44

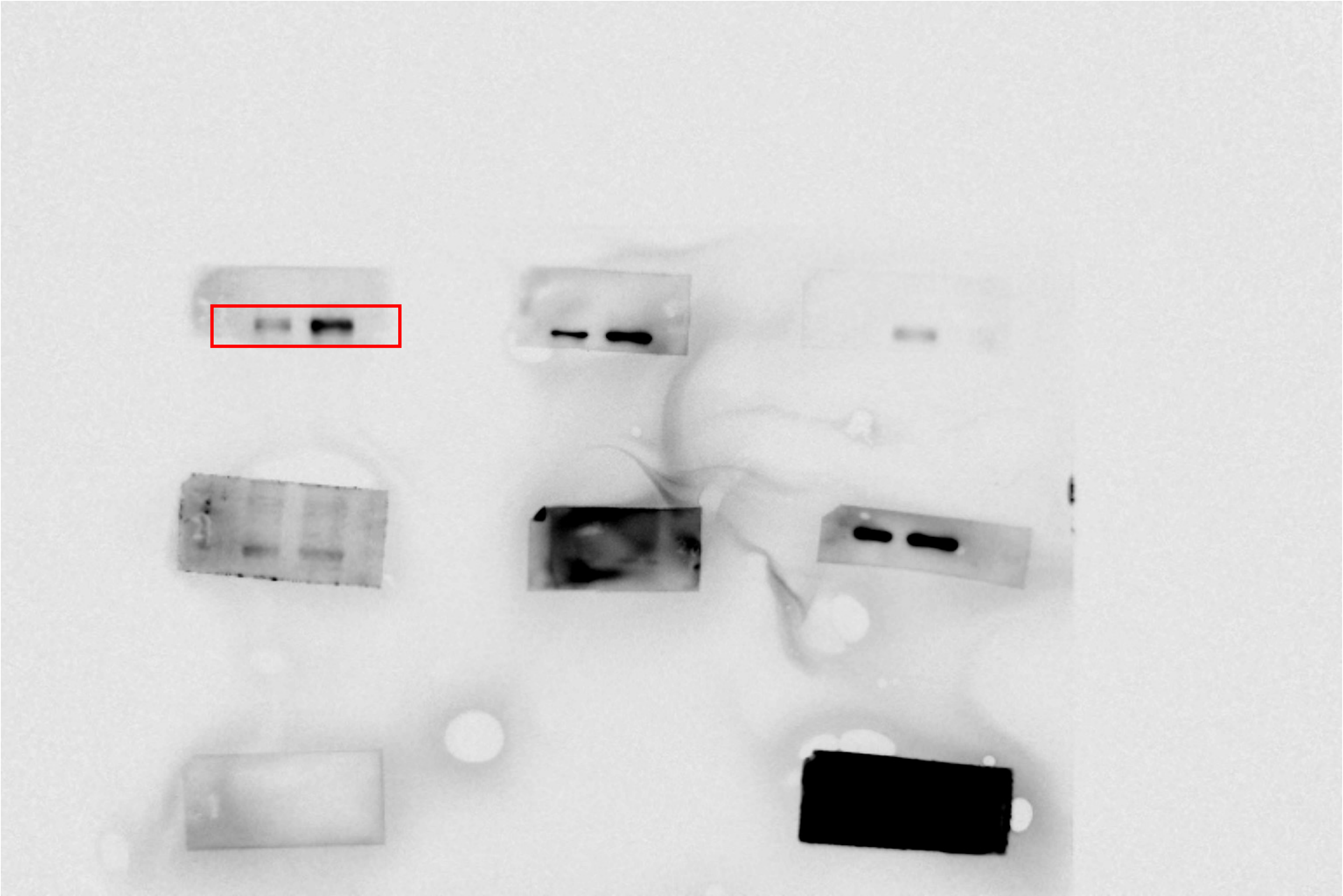

Full and uncropped western blots

Figure 4B

AGS- SOX9

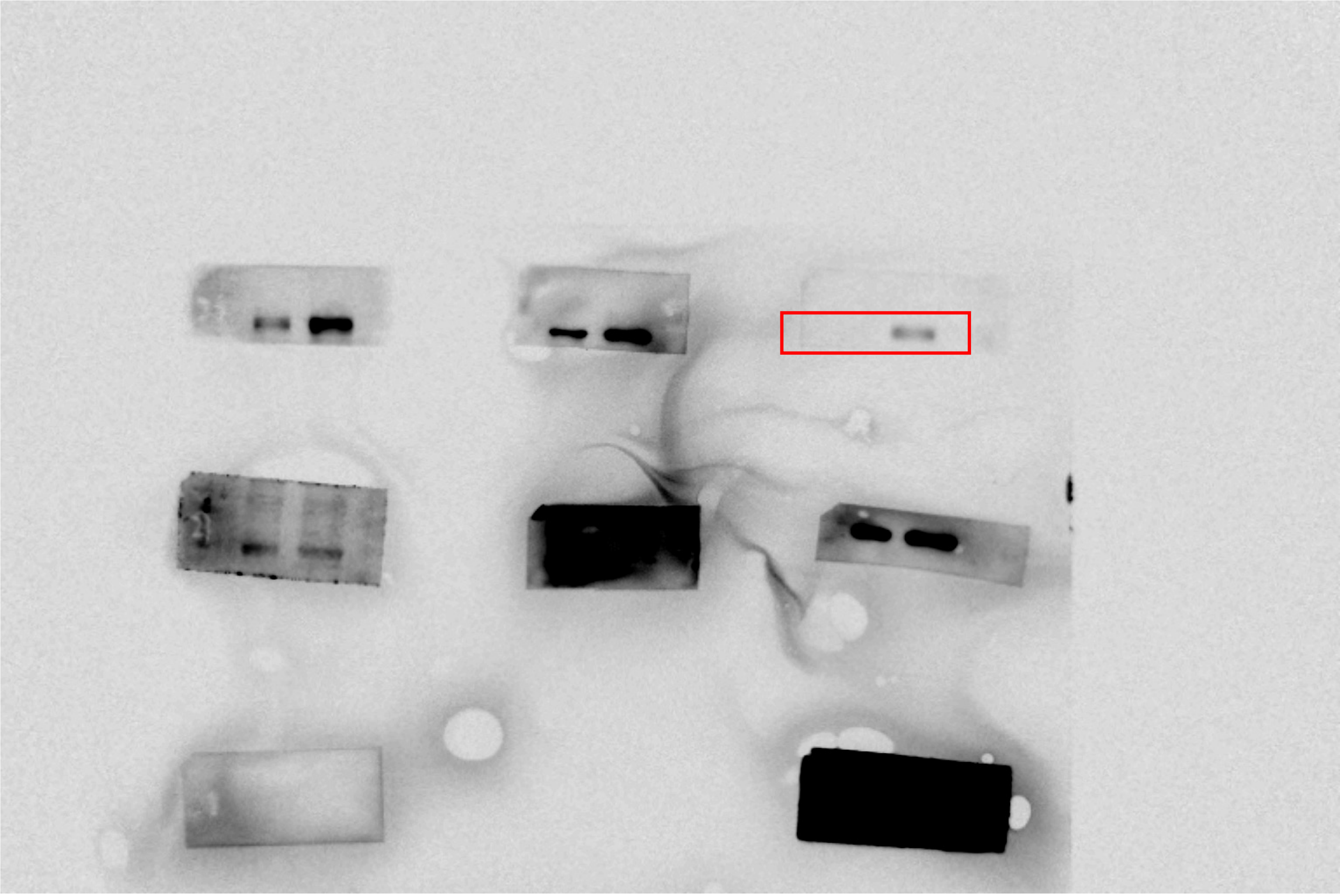

Full and uncropped western blots

Figure 4B

AGS- SOX2

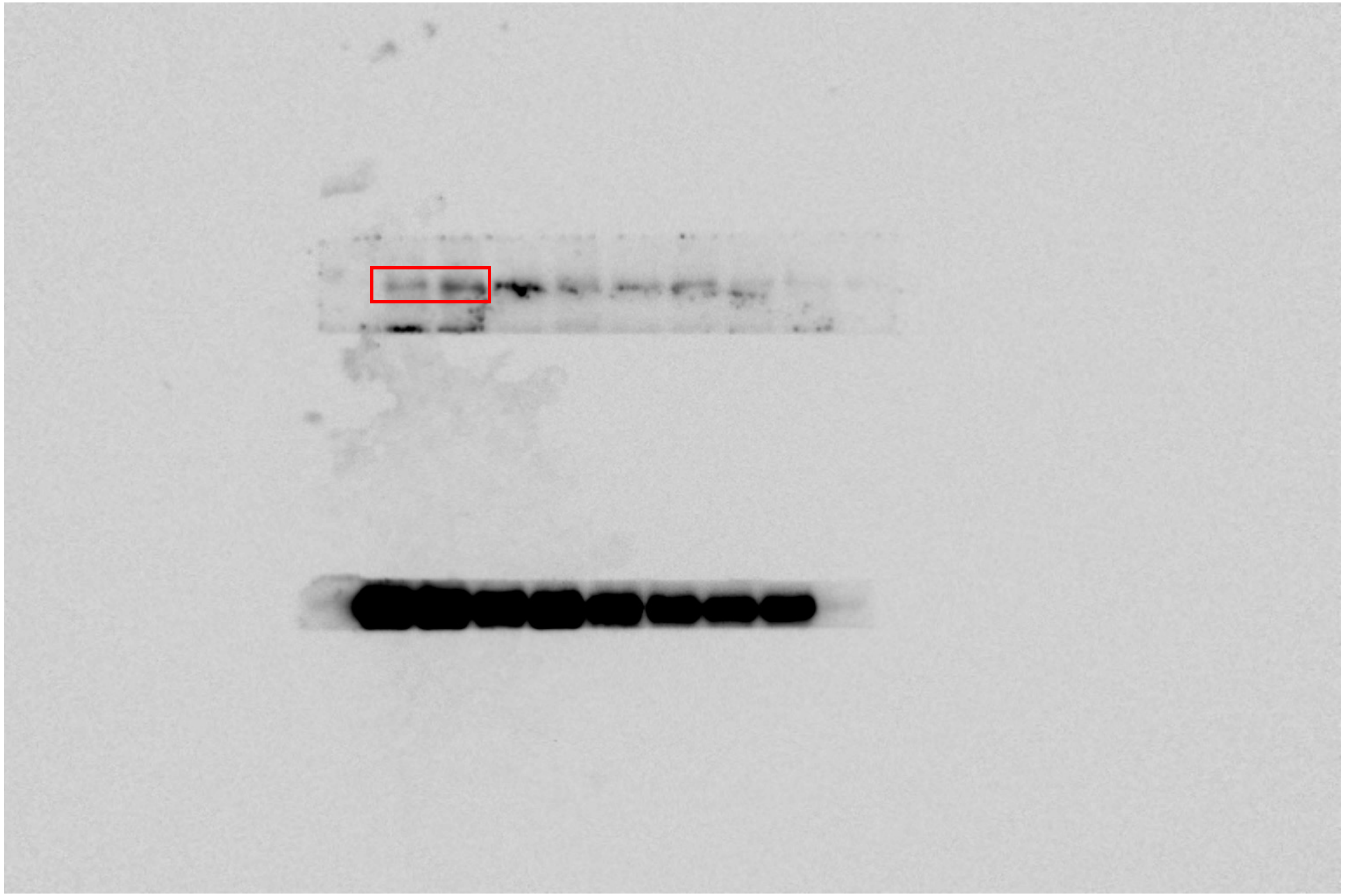

Full and uncropped western blots

Figure 4B

AGS- LGR4

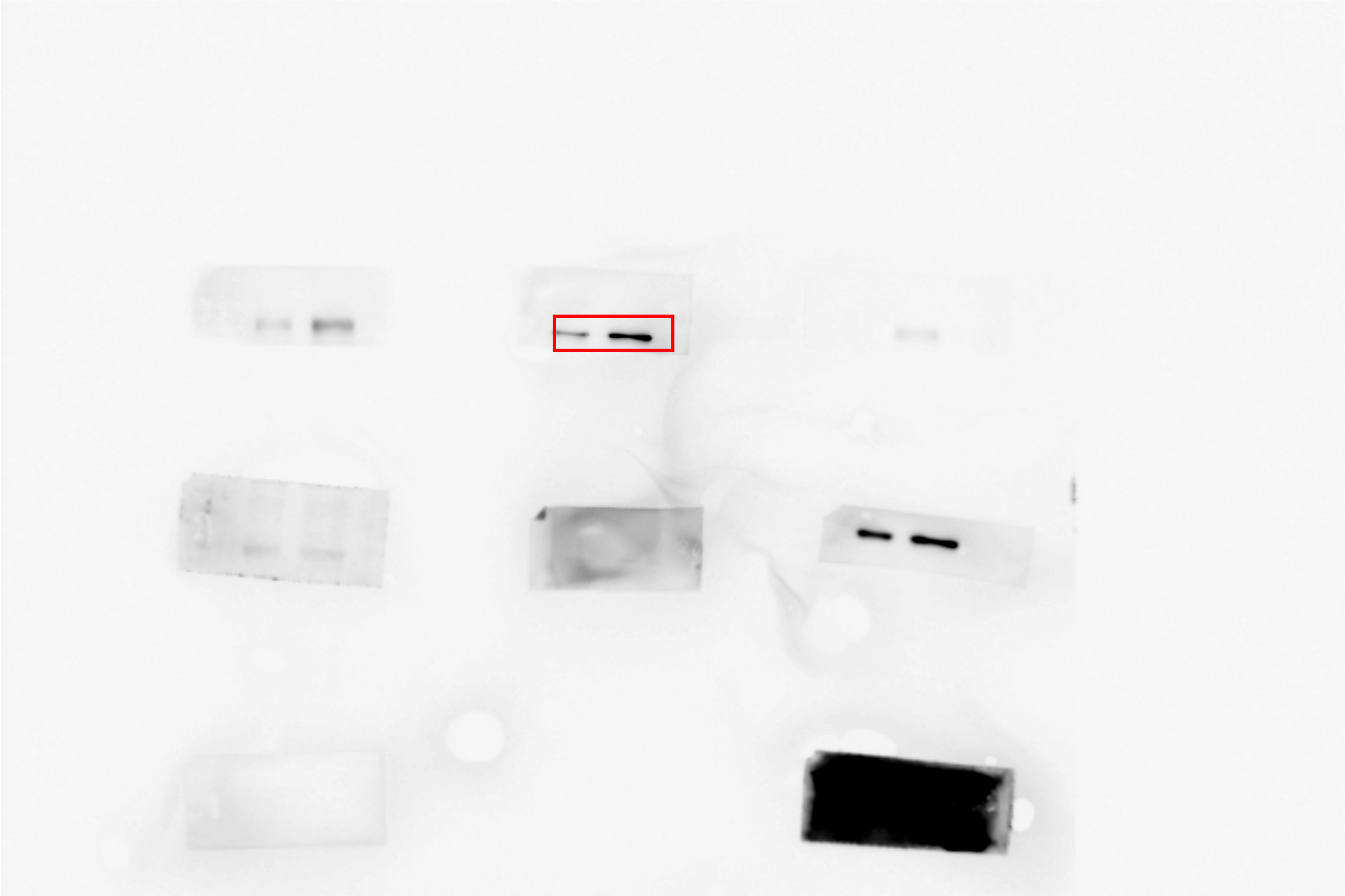

Full and uncropped western blots

Figure 4B

AGS- GAPDH

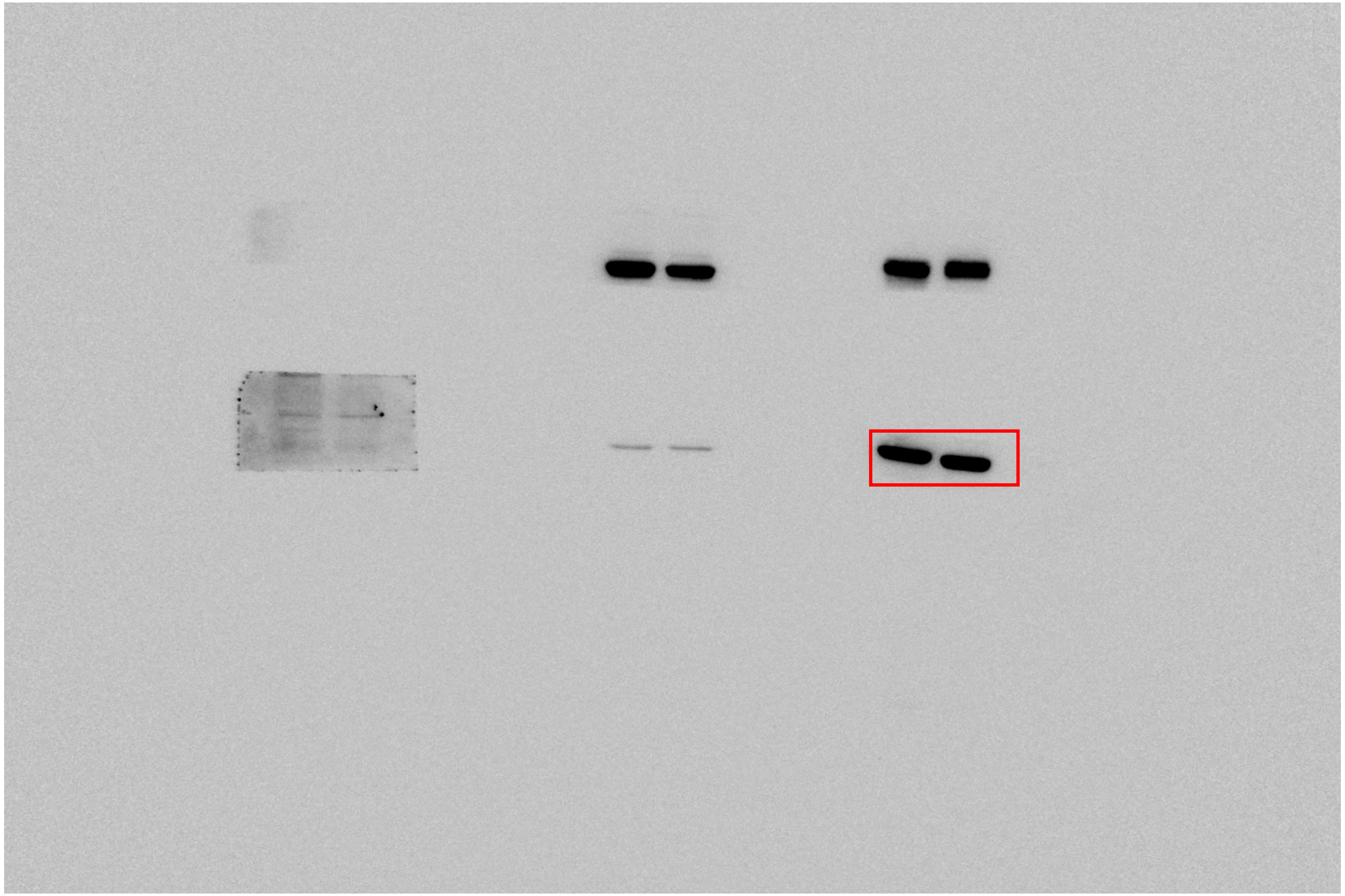

Full and uncropped western blots

Figure 4B

N87- ONECUT2

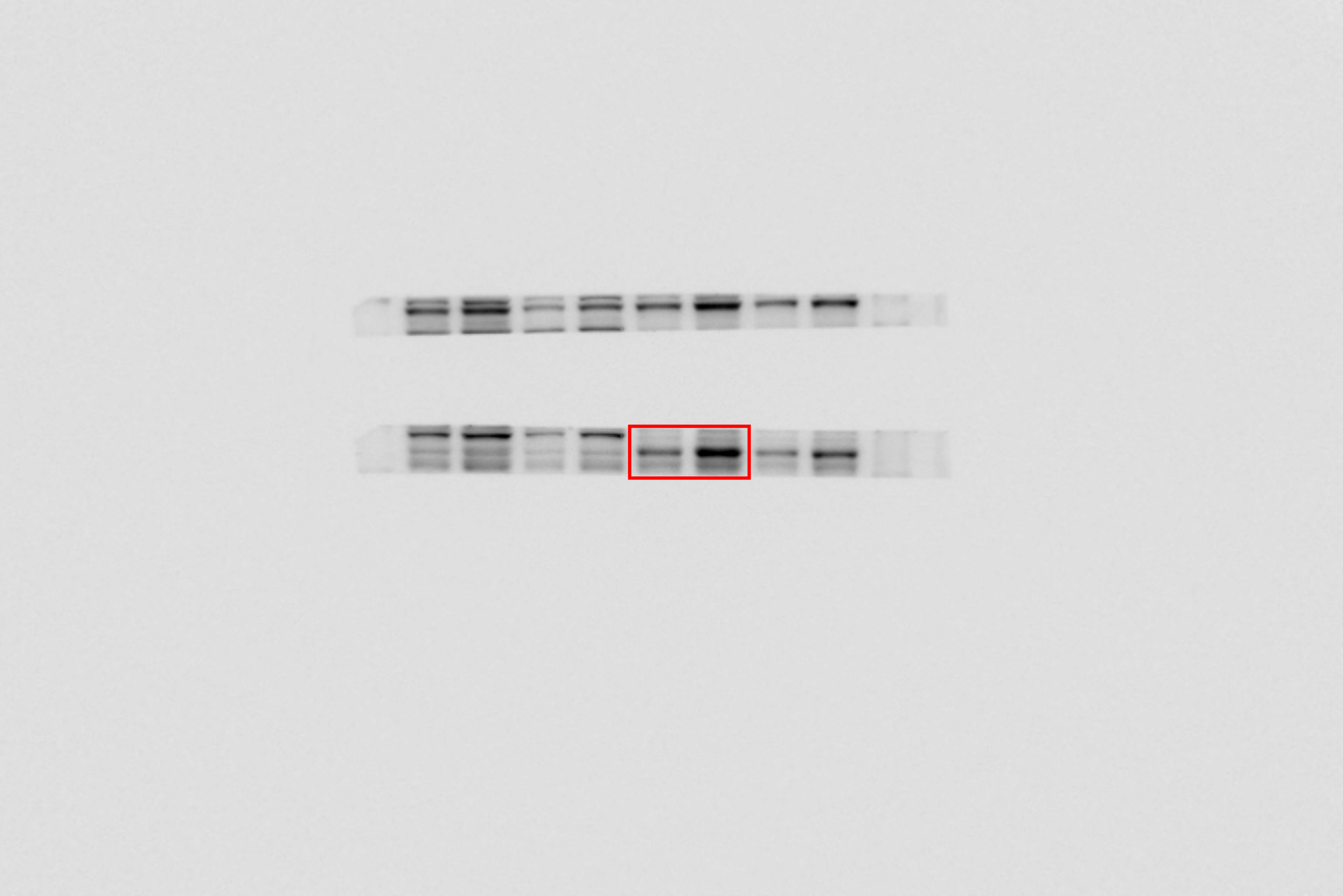

Full and uncropped western blots

Figure 4B

N87- CD44

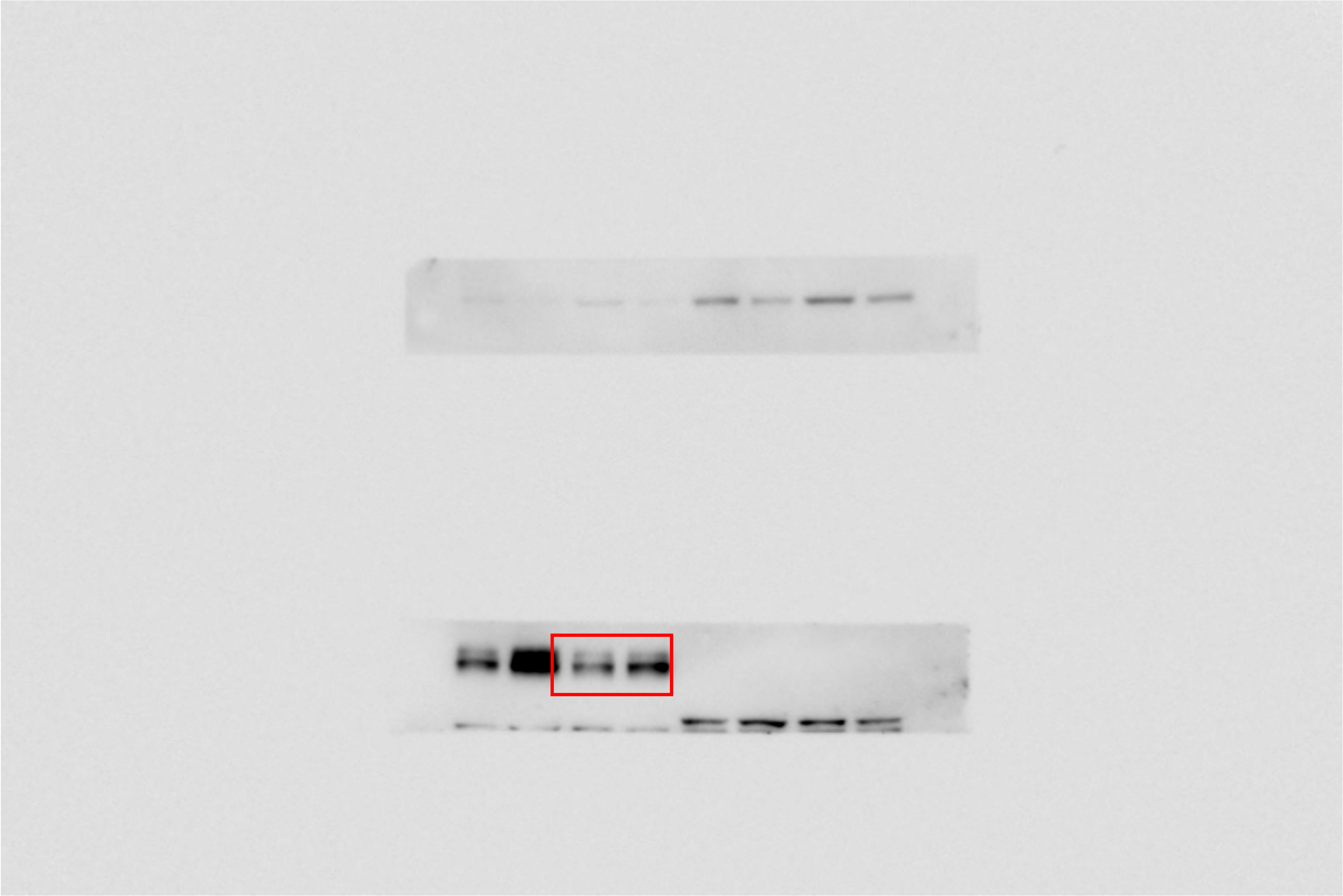

Full and uncropped western blots

Figure 4B

N87- SOX9

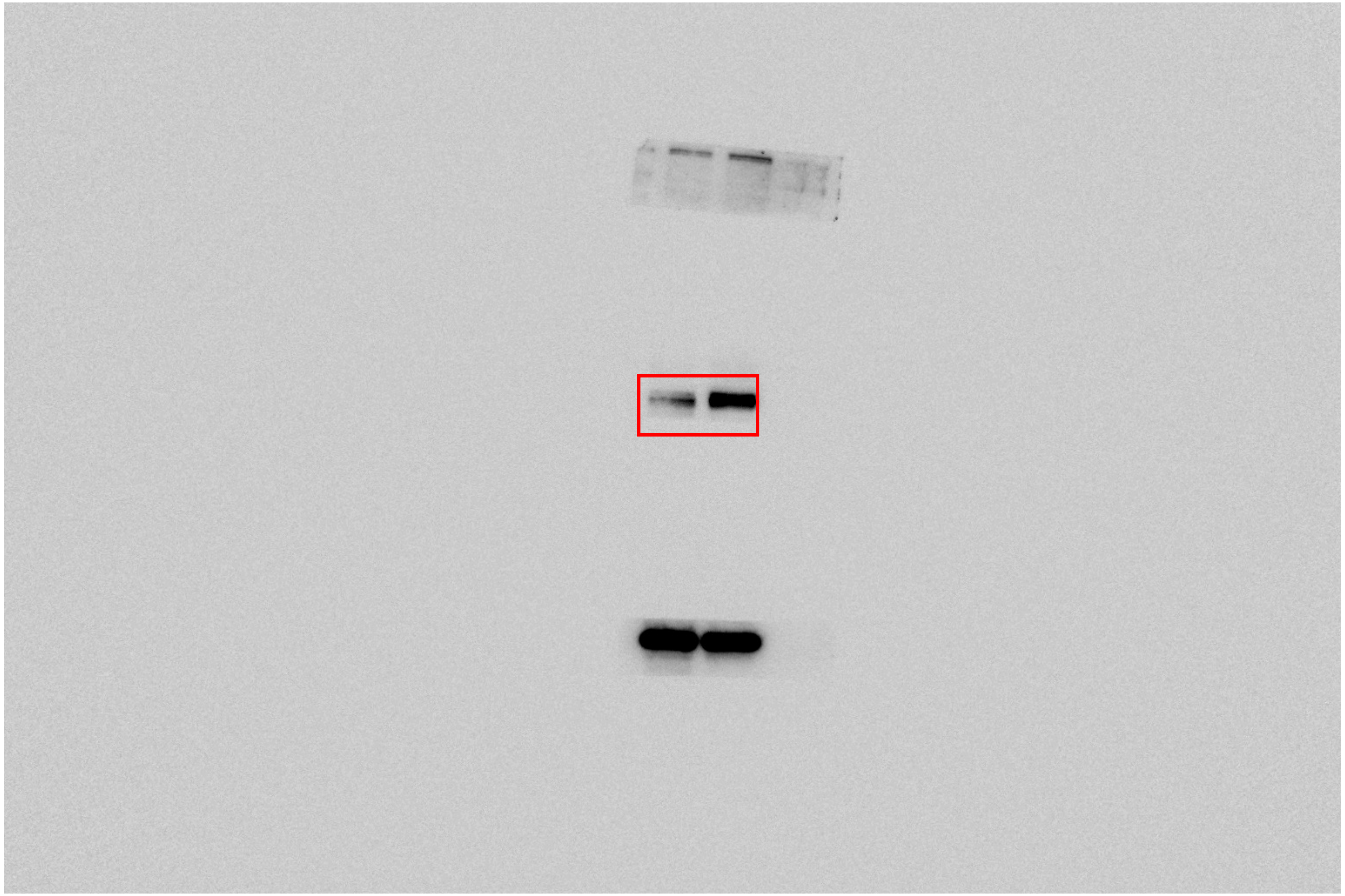

Full and uncropped western blots

Figure 4B

N87- SOX2

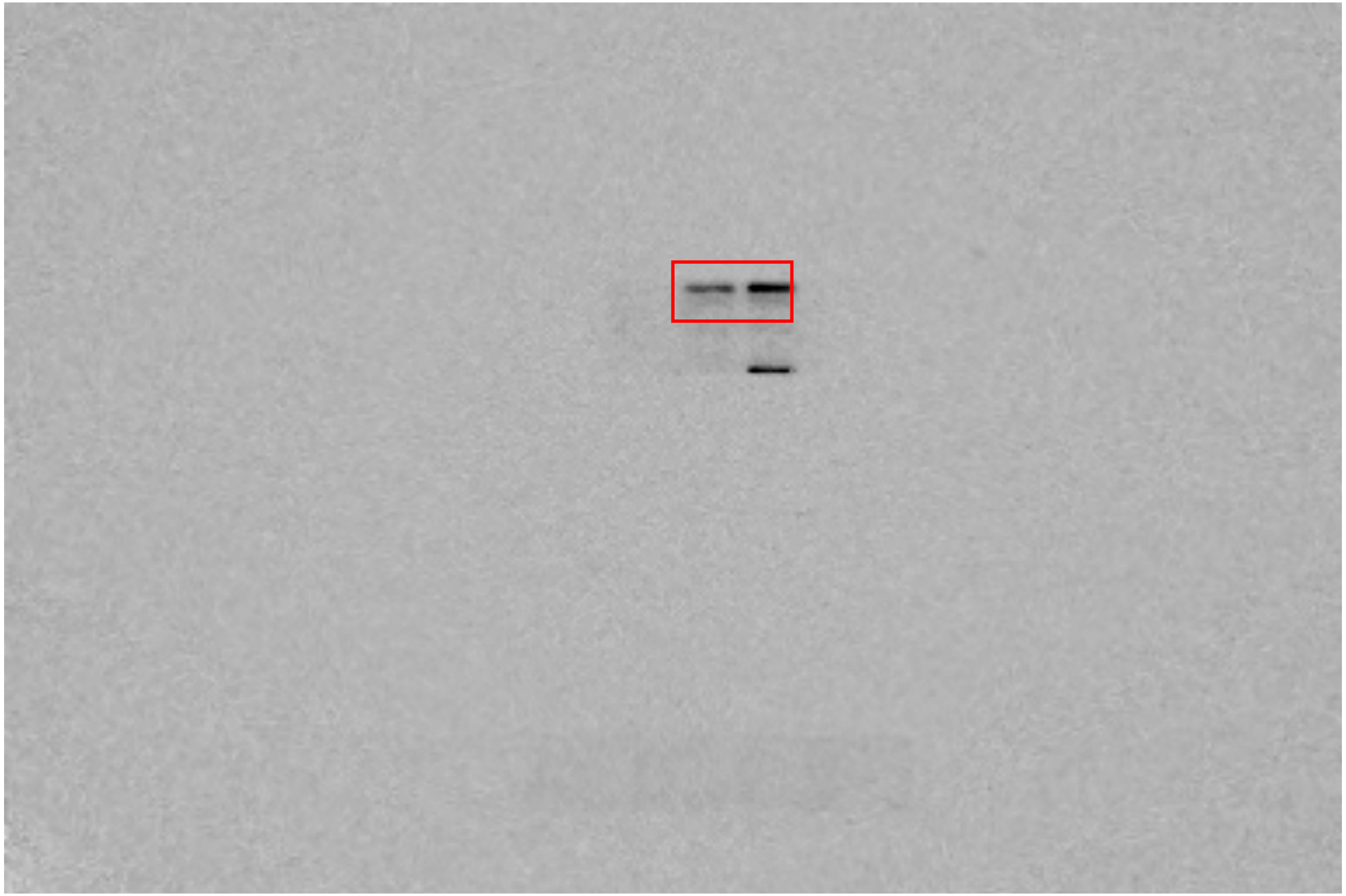

Full and uncropped western blots

Figure 4B

N87- LGR4

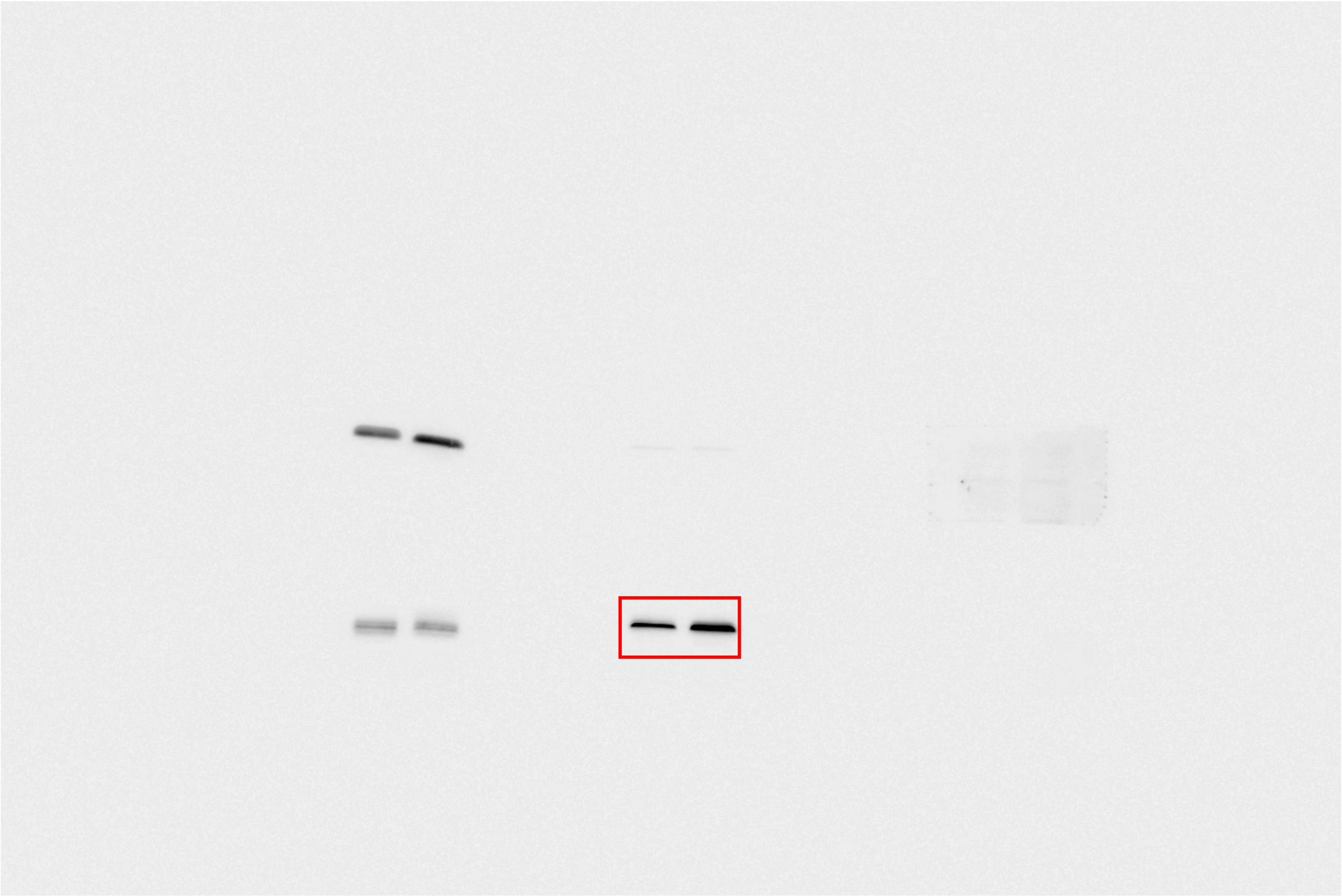

Full and uncropped western blots

Figure 4B

N87- GAPDH

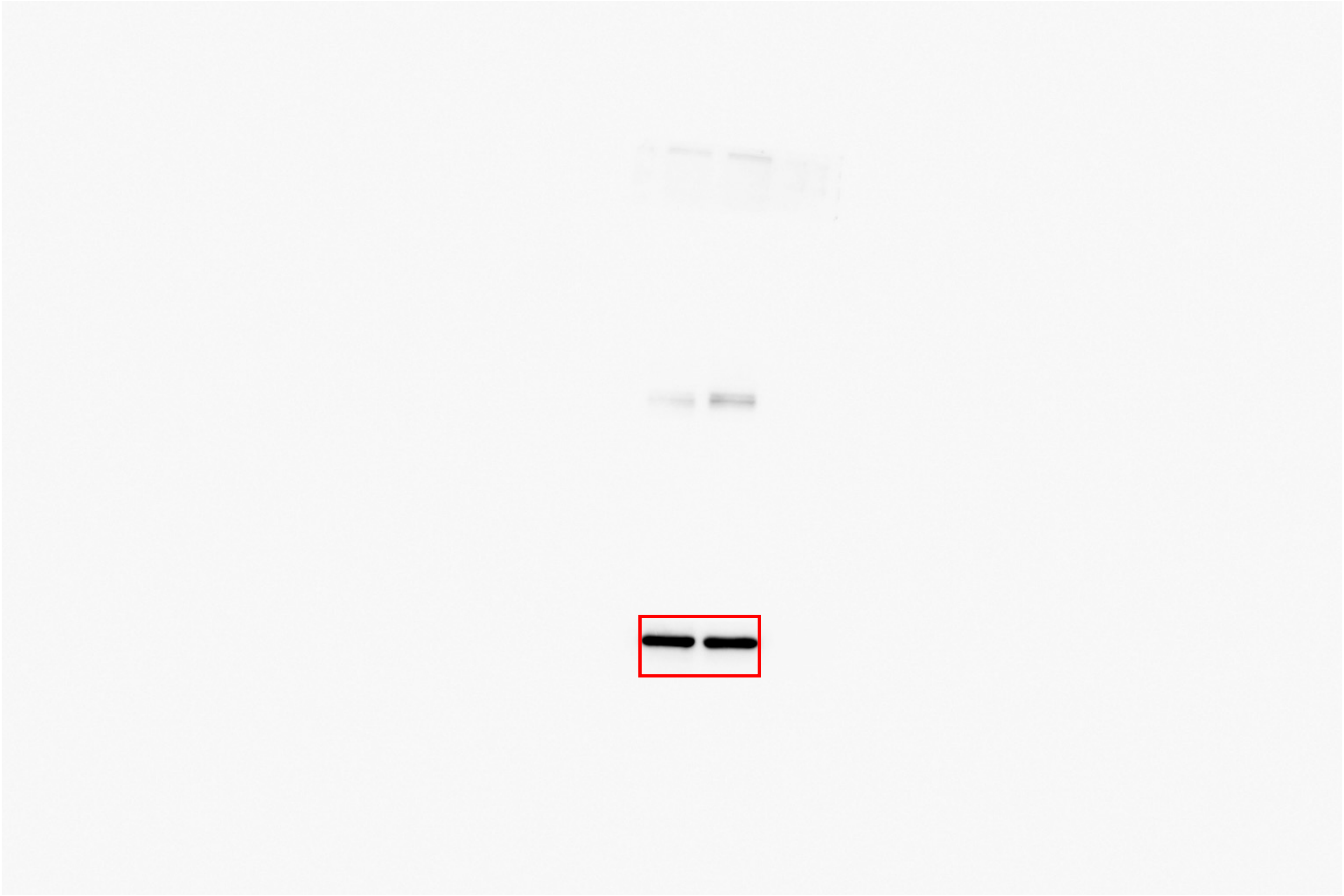

Full and uncropped western blots

Figure 4C

WB WITH BAND SIZE

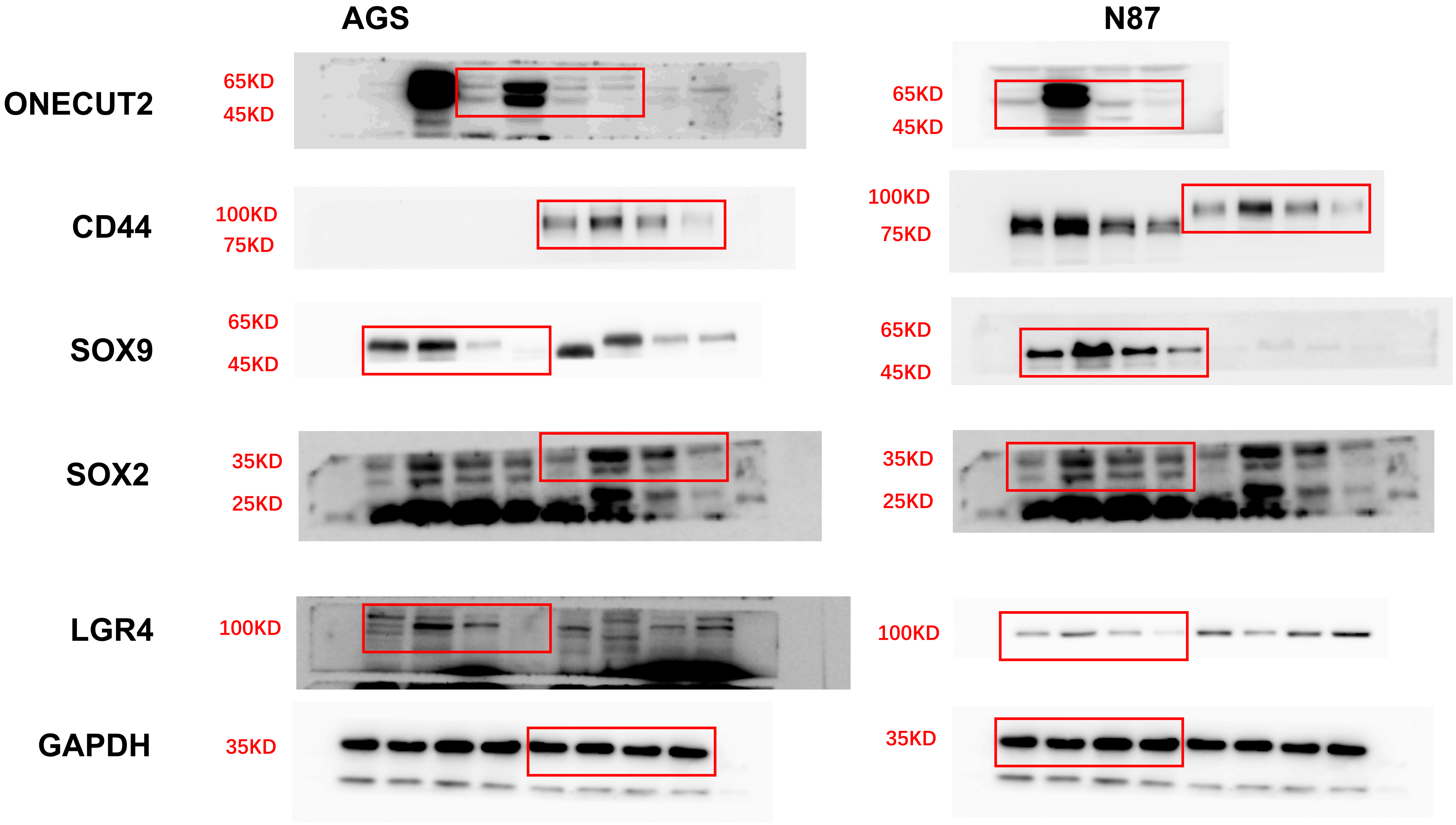

Figure 4C

AGS- ONECUT2

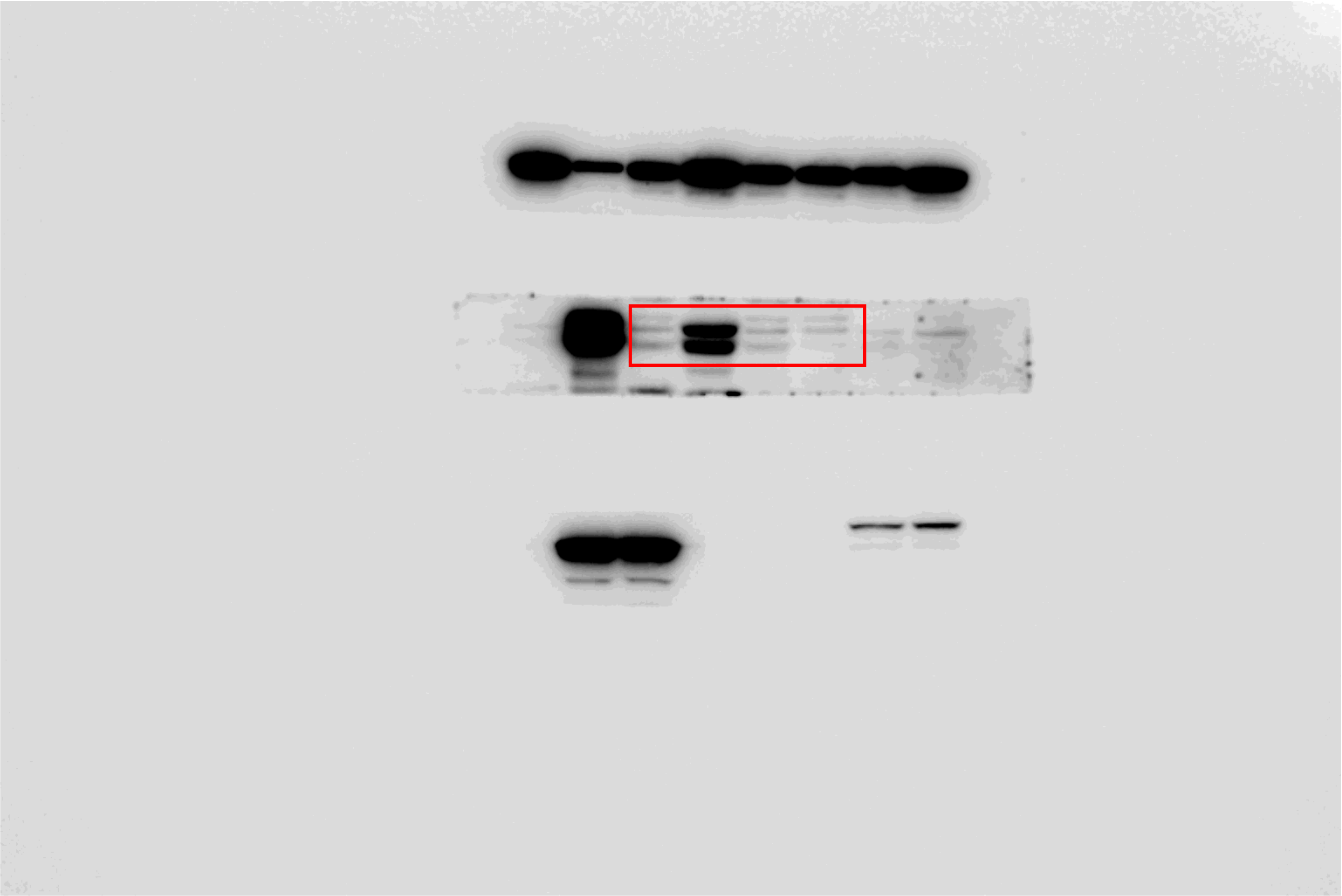

Full and uncropped western blots

Figure 4C

AGS- CD44

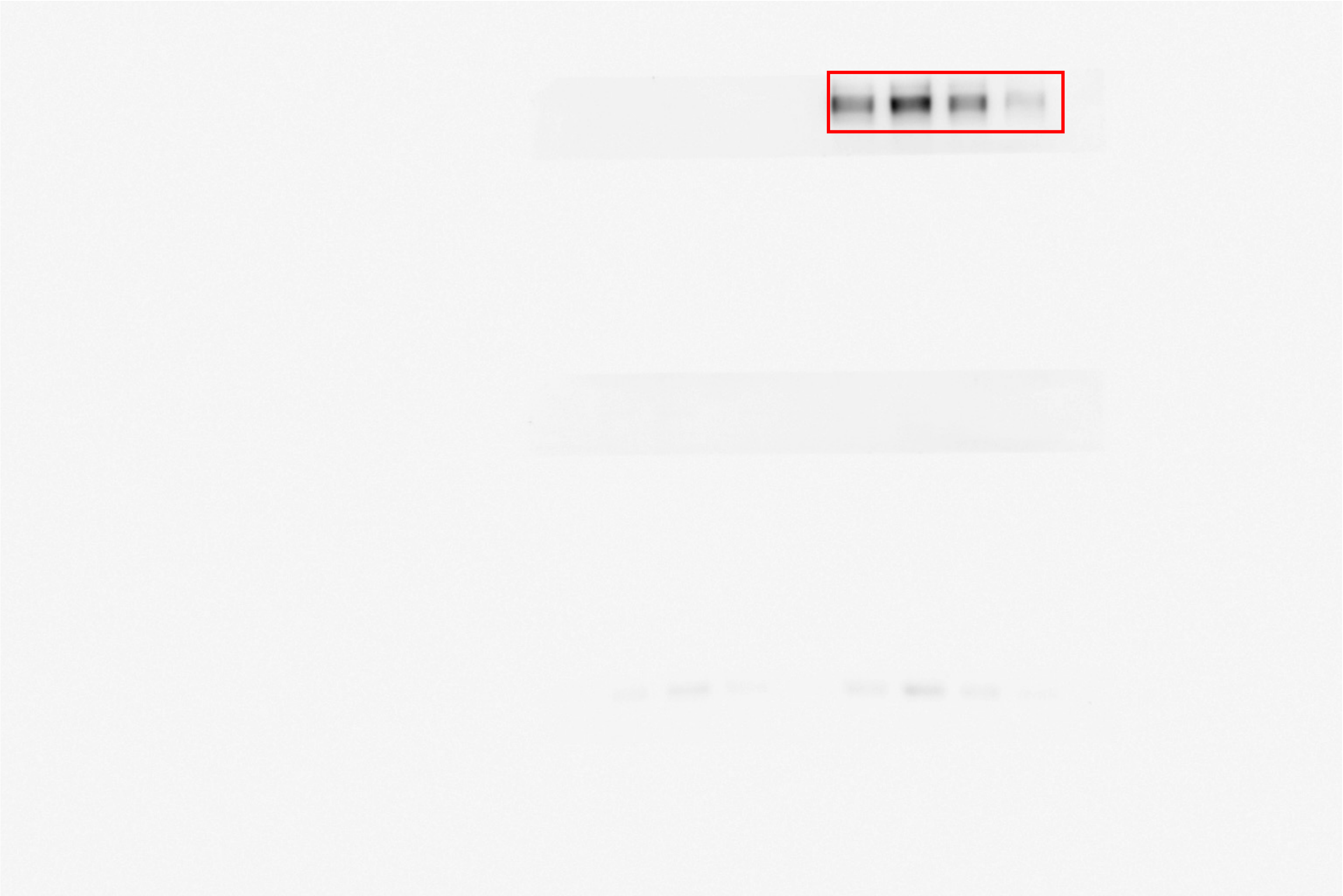

Full and uncropped western blots

Figure 4C

AGS- SOX9

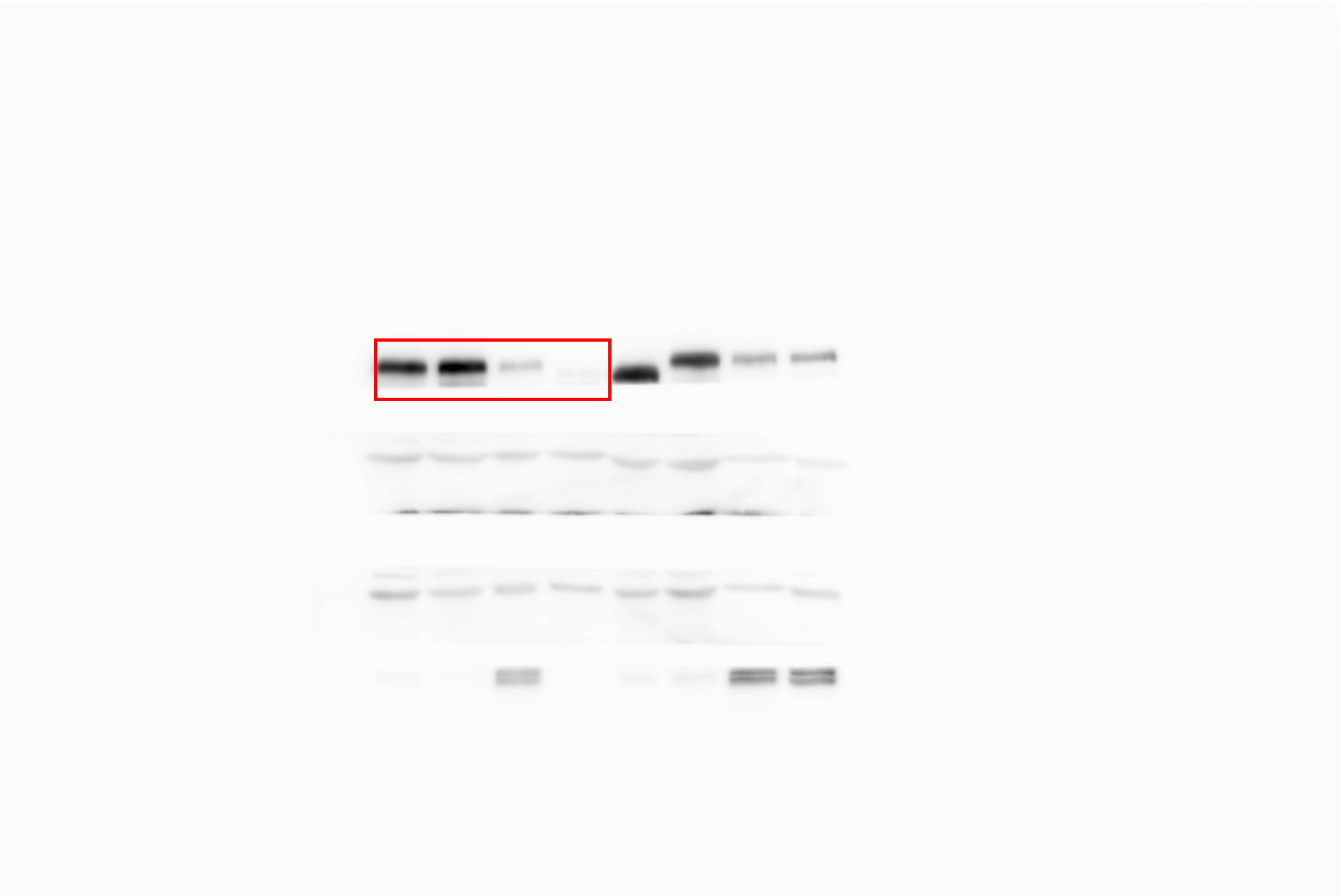

Full and uncropped western blots

Figure 4C

AGS- SOX2

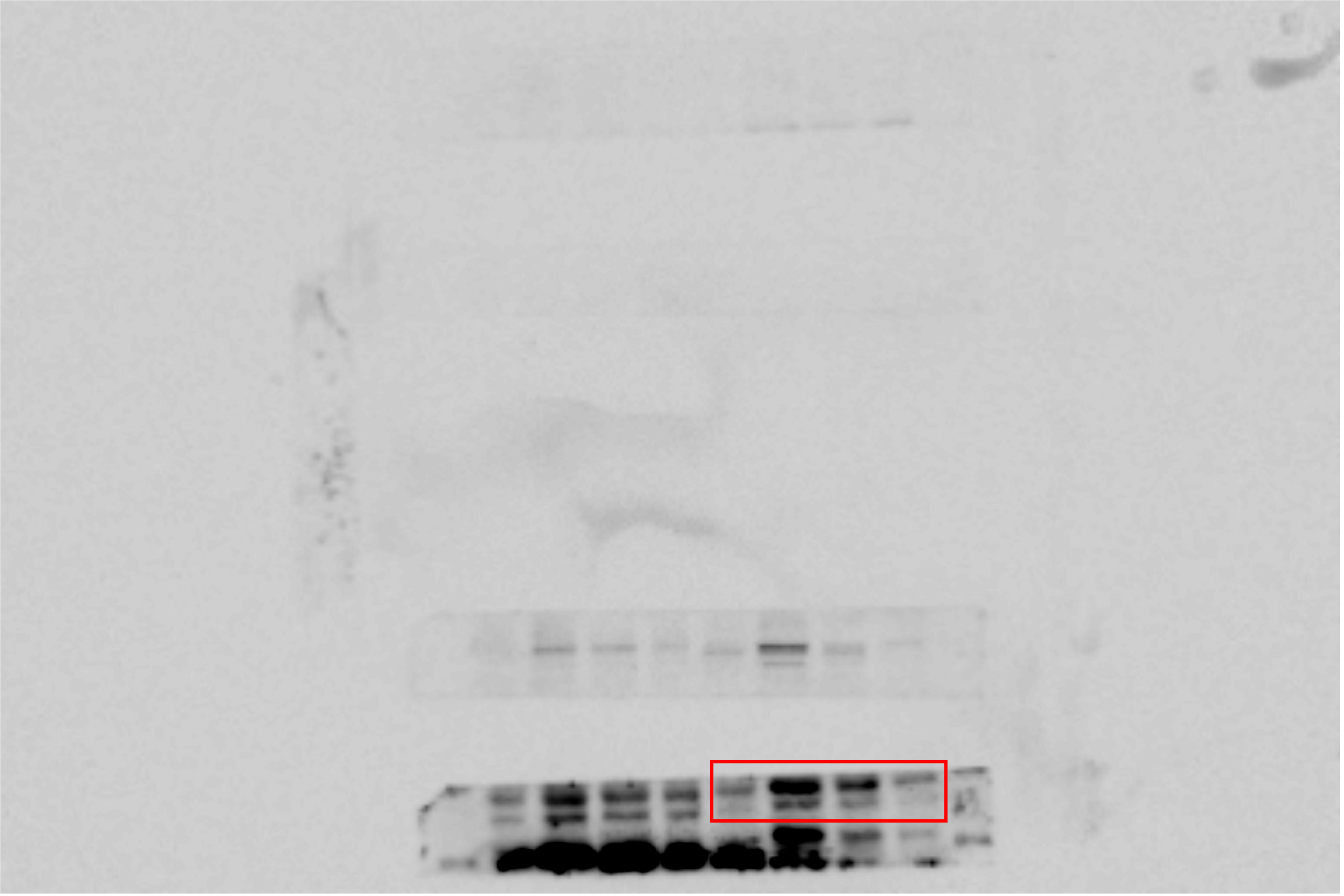

Full and uncropped western blots

Figure 4C

AGS- LGR4

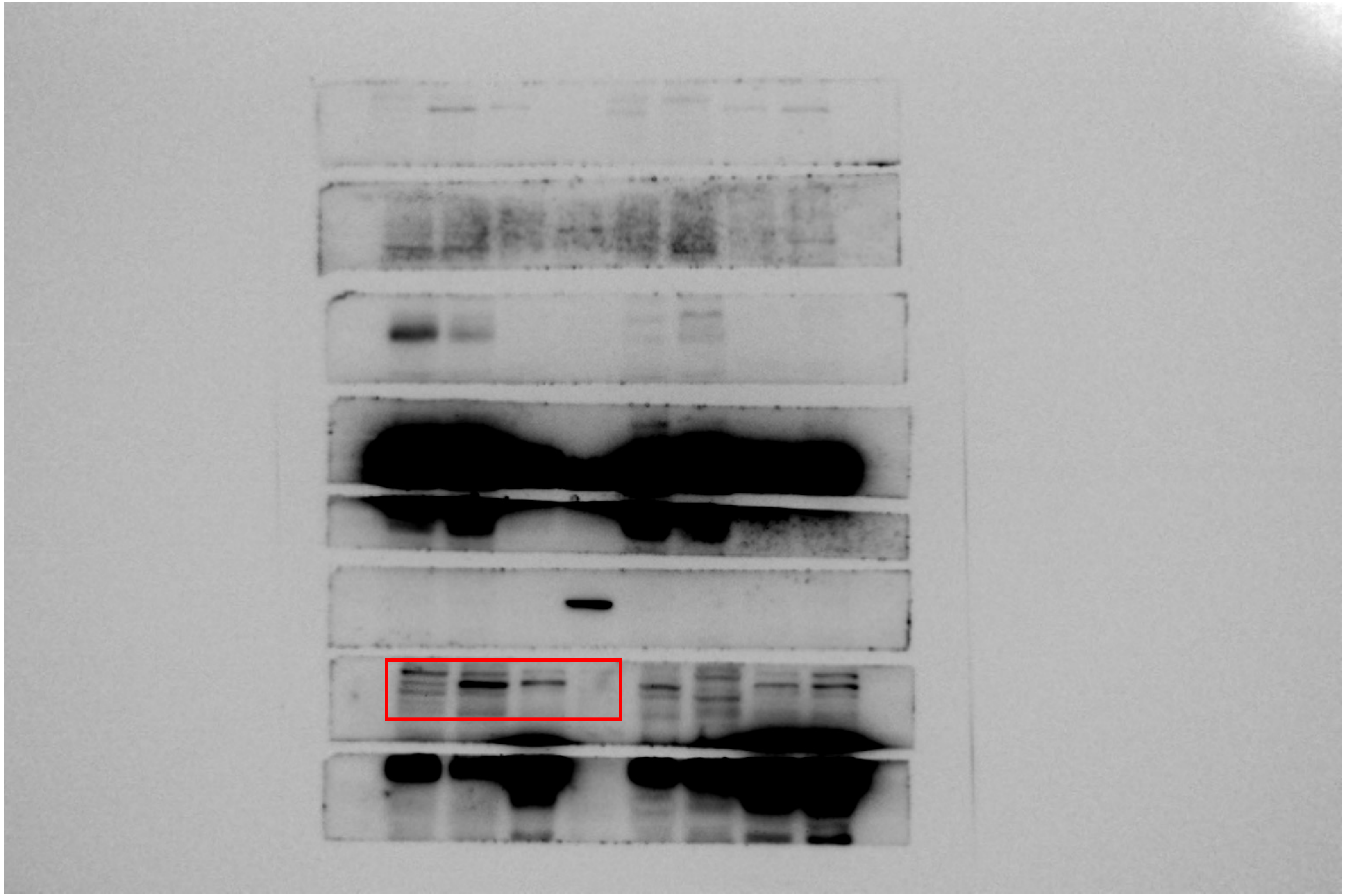

Full and uncropped western blots

Figure 4C

AGS- GAPDH

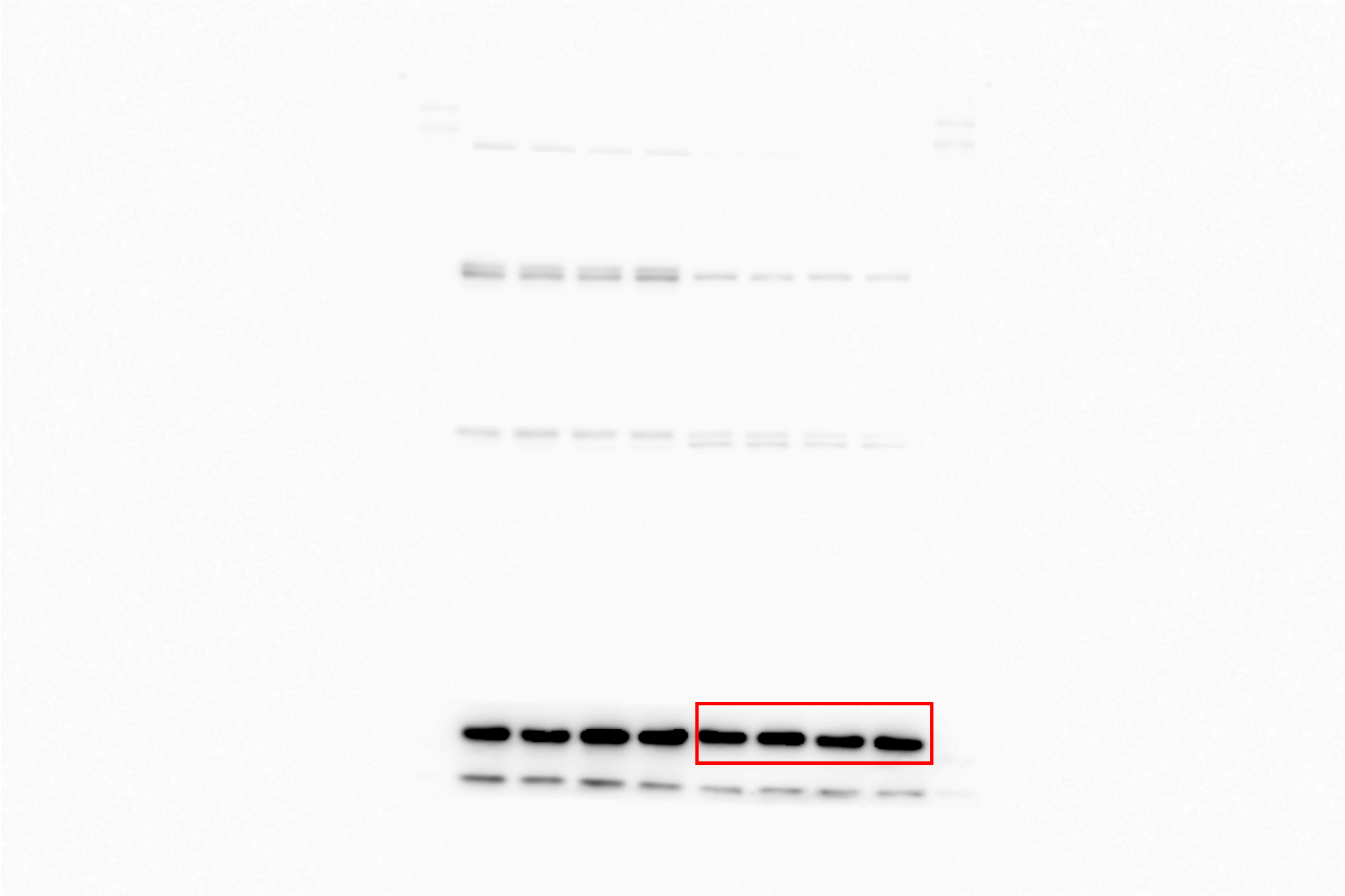

Full and uncropped western blots

Figure 4C

N87- ONECUT2

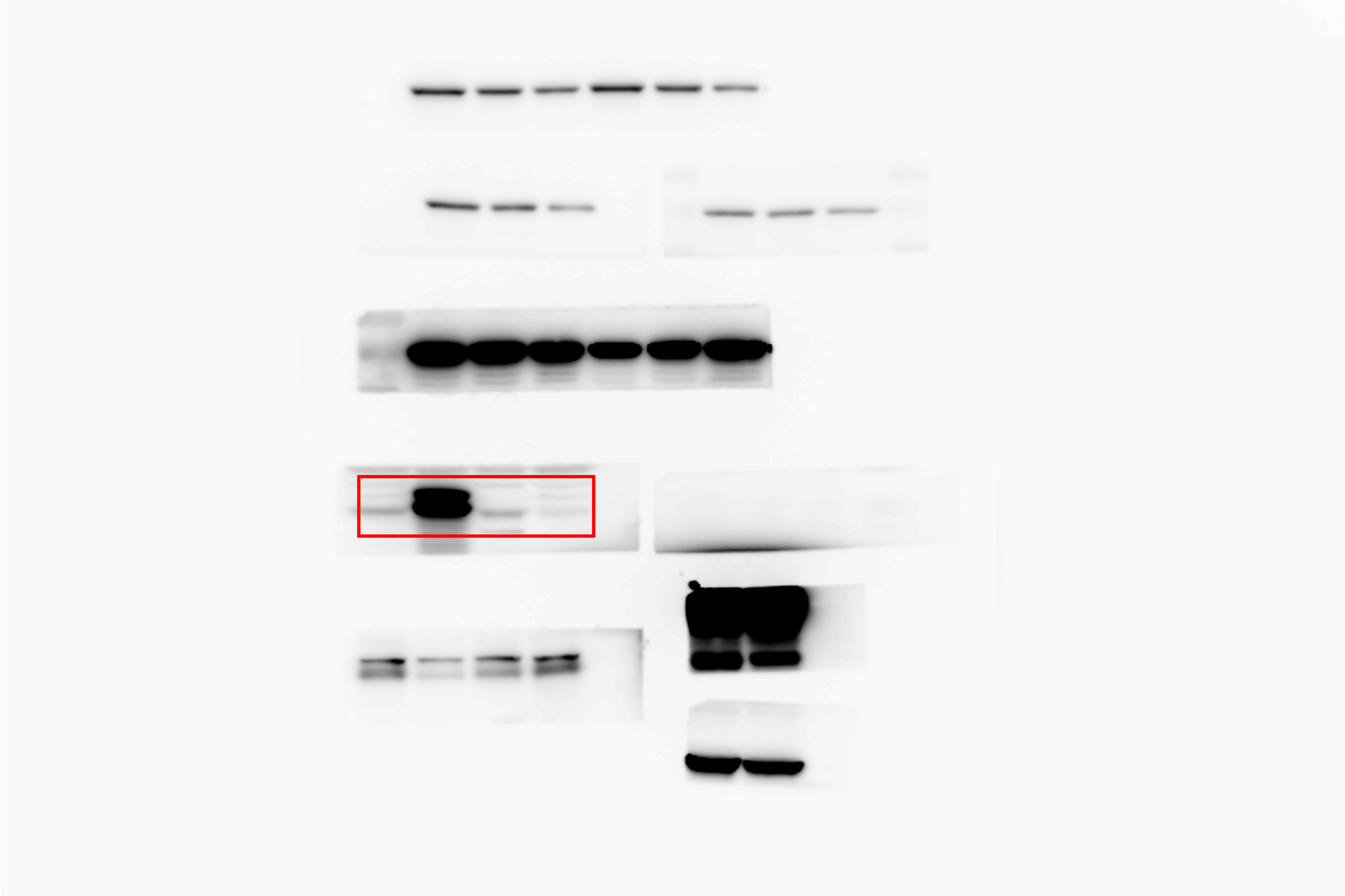

Full and uncropped western blots

Figure 4C

N87- CD44

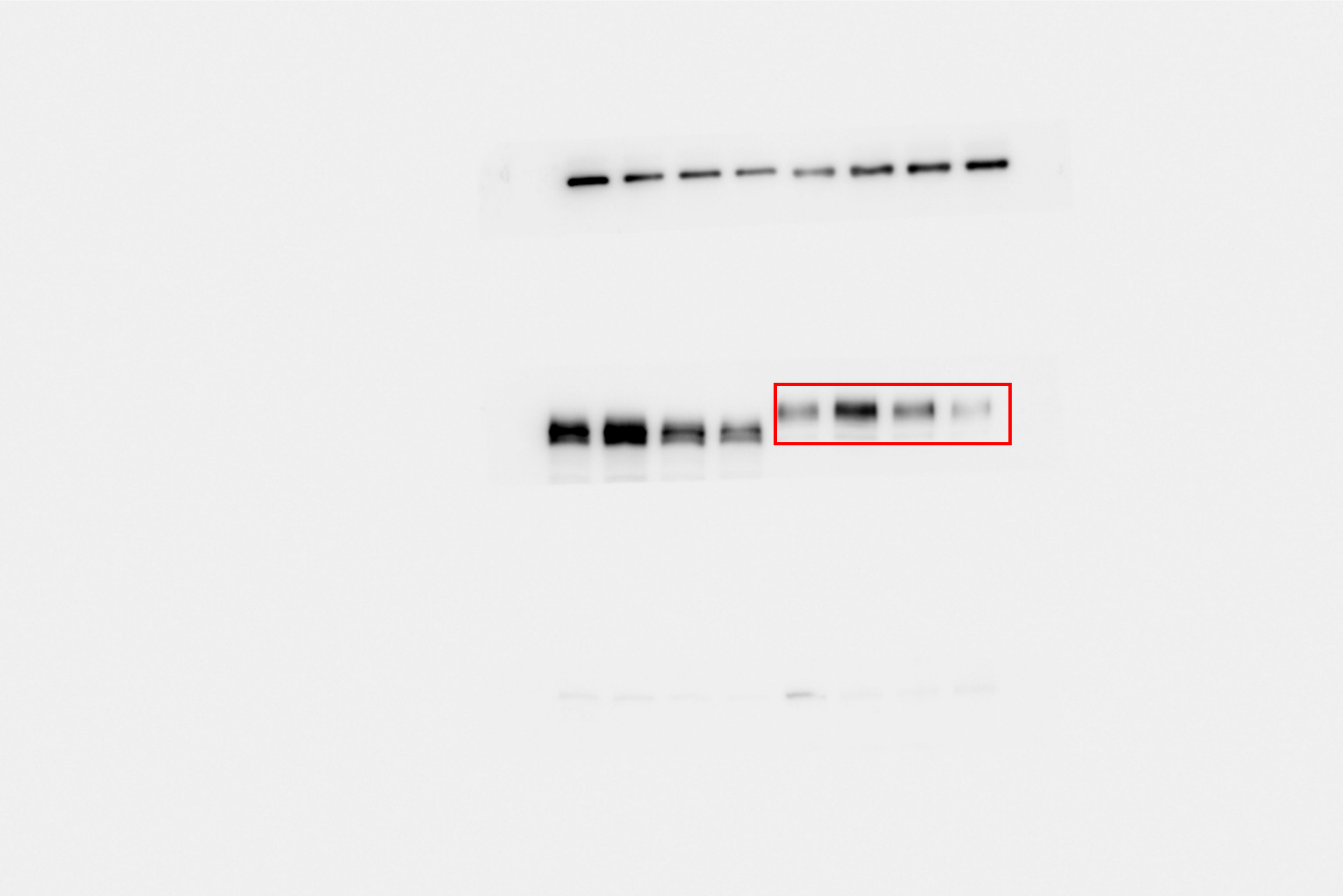

Full and uncropped western blots

Figure 4C

N87- SOX9

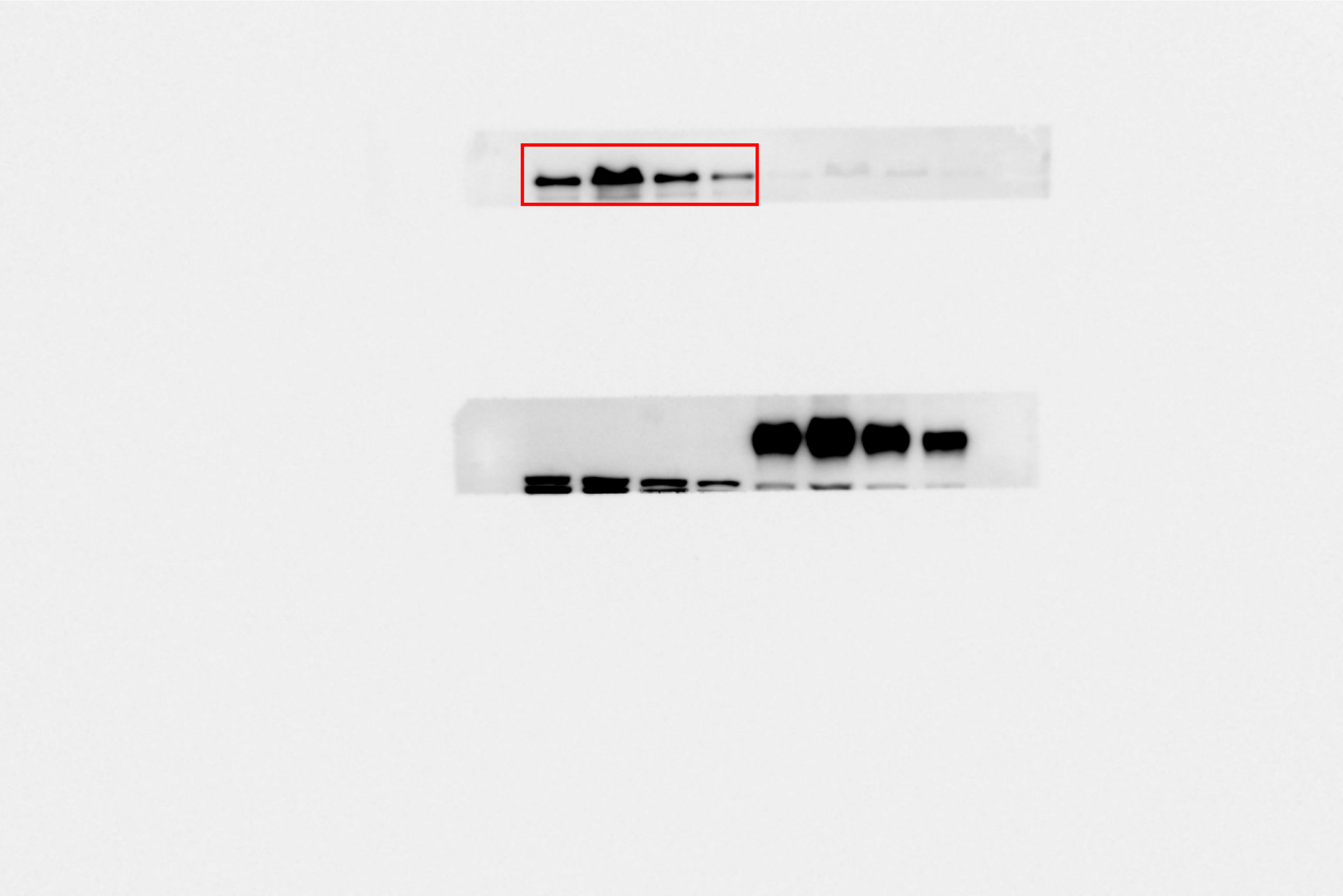

Full and uncropped western blots

Figure 4C

N87- SOX2

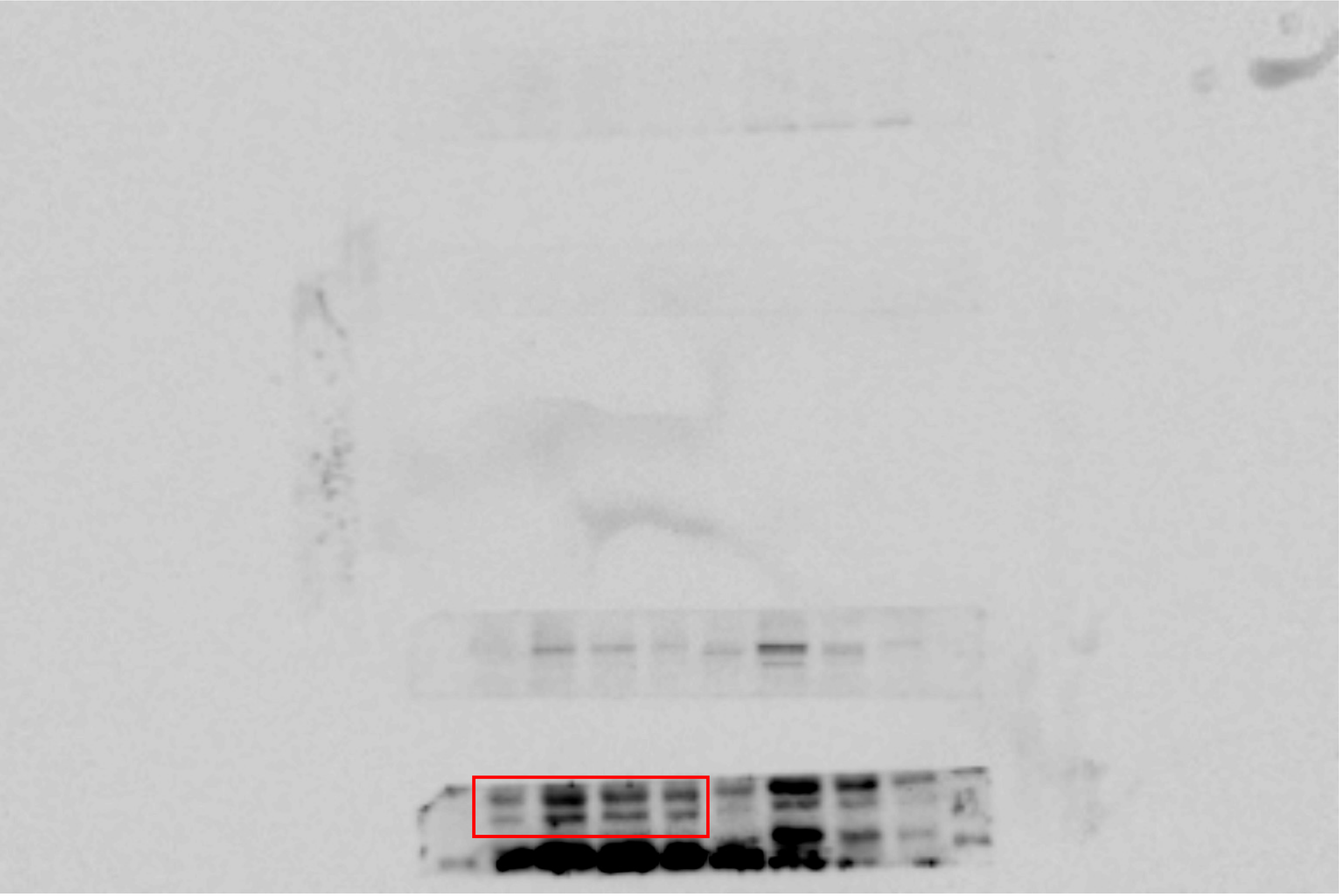

Full and uncropped western blots

Figure 4C

N87- LGR4

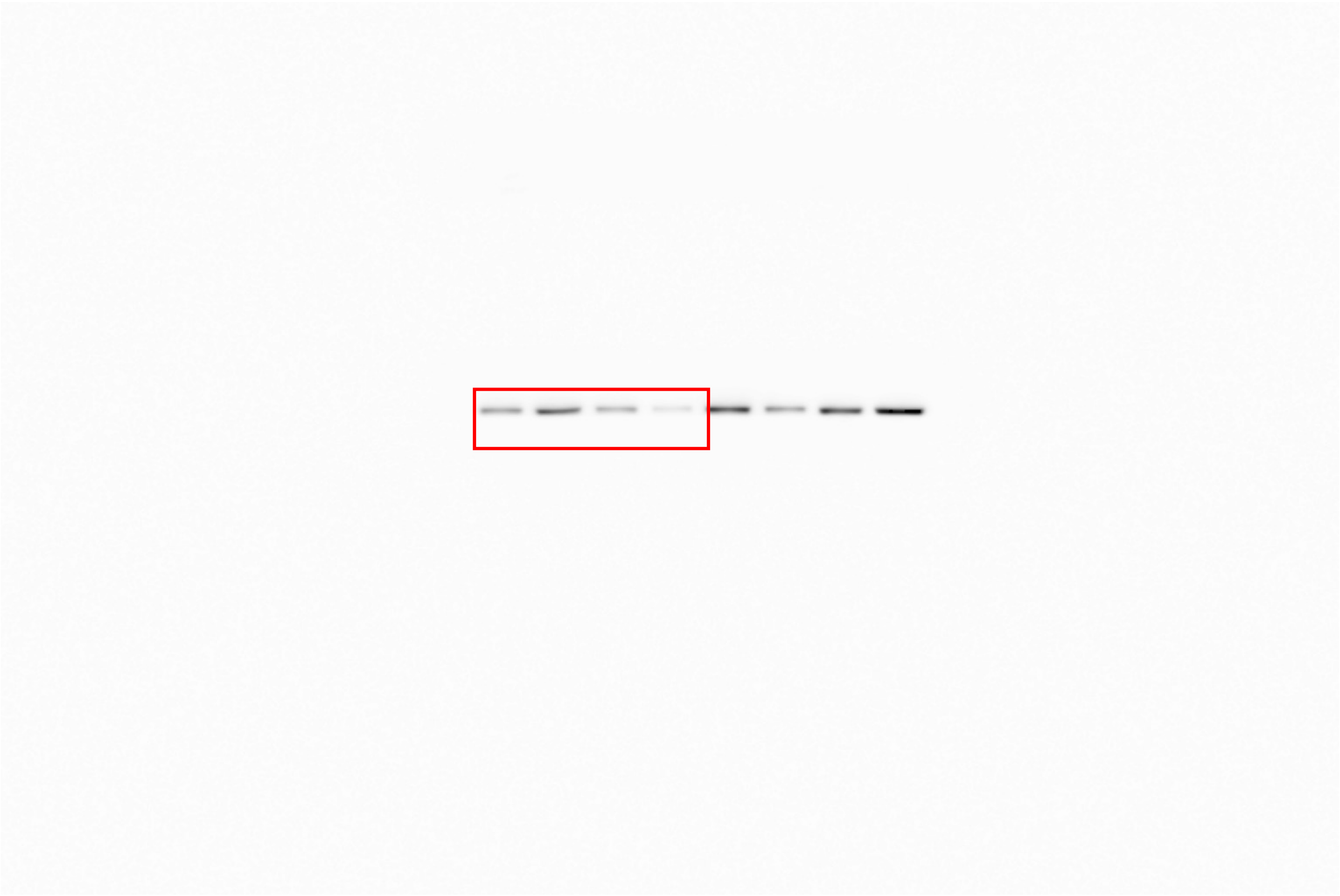

Full and uncropped western blots

Figure 4C

N87- GAPDH

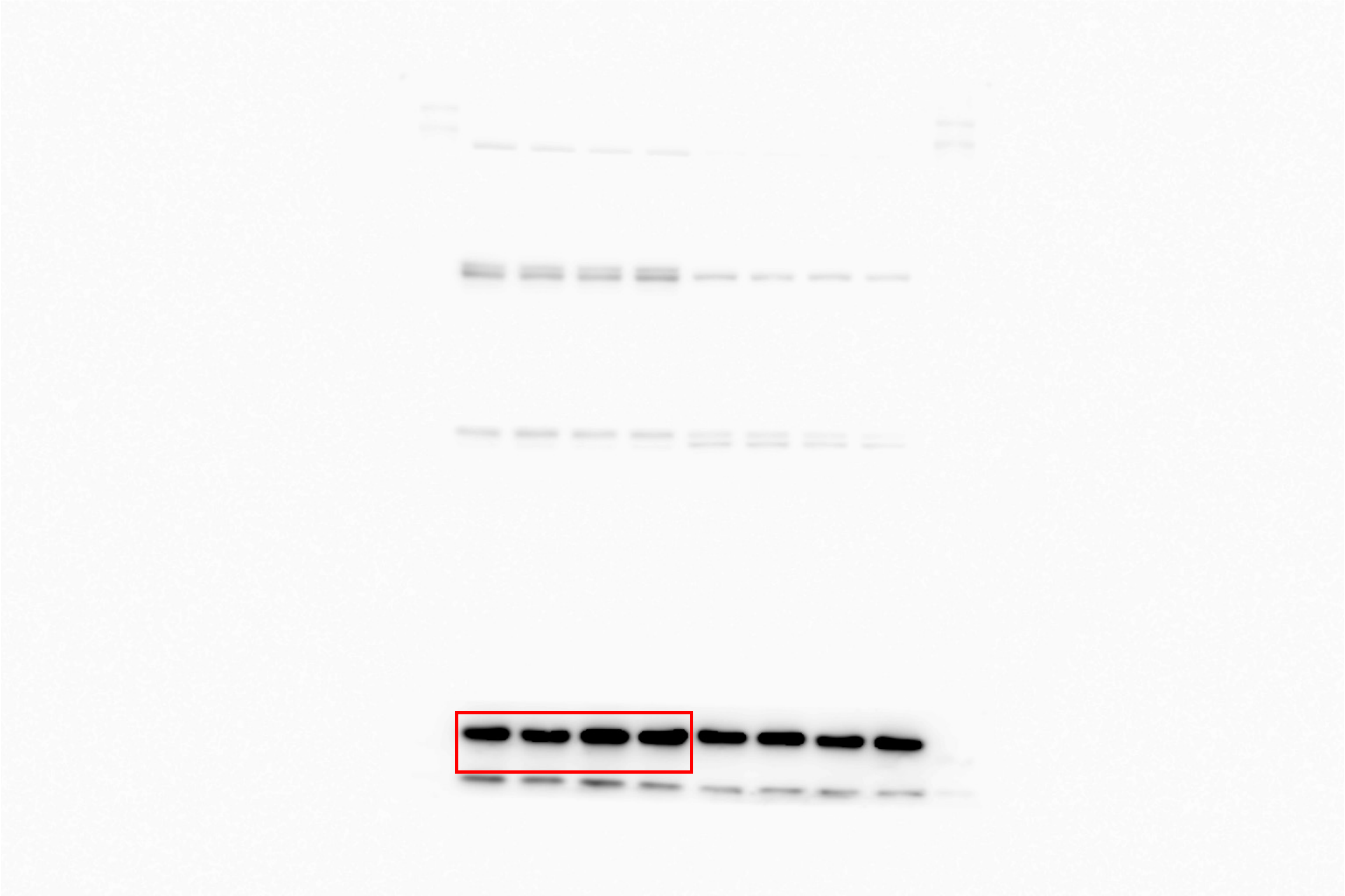

Full and uncropped western blots

Figure 5B

SH-ONECUT2

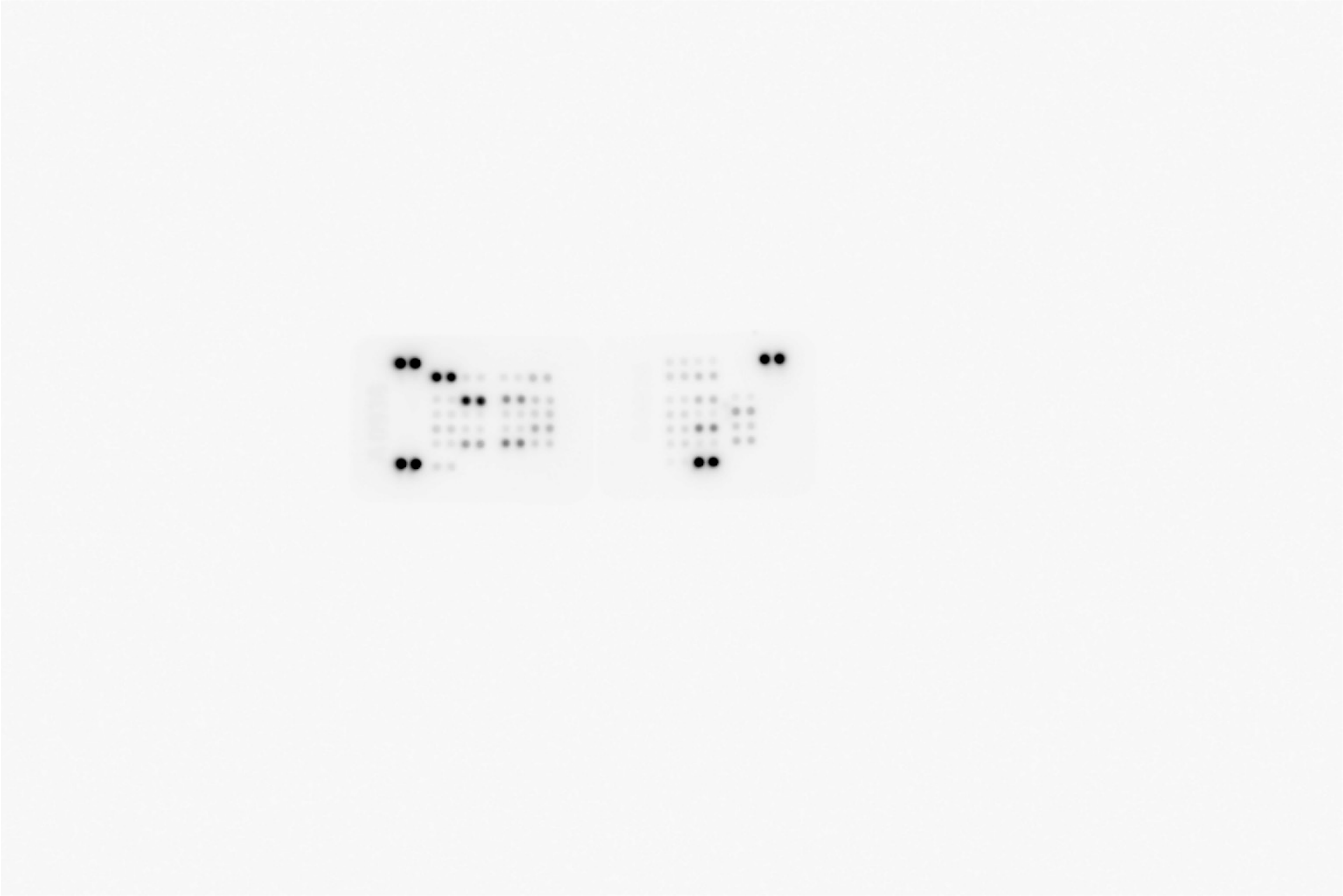

Full and uncropped western blots

Figure 5B

SH- NC

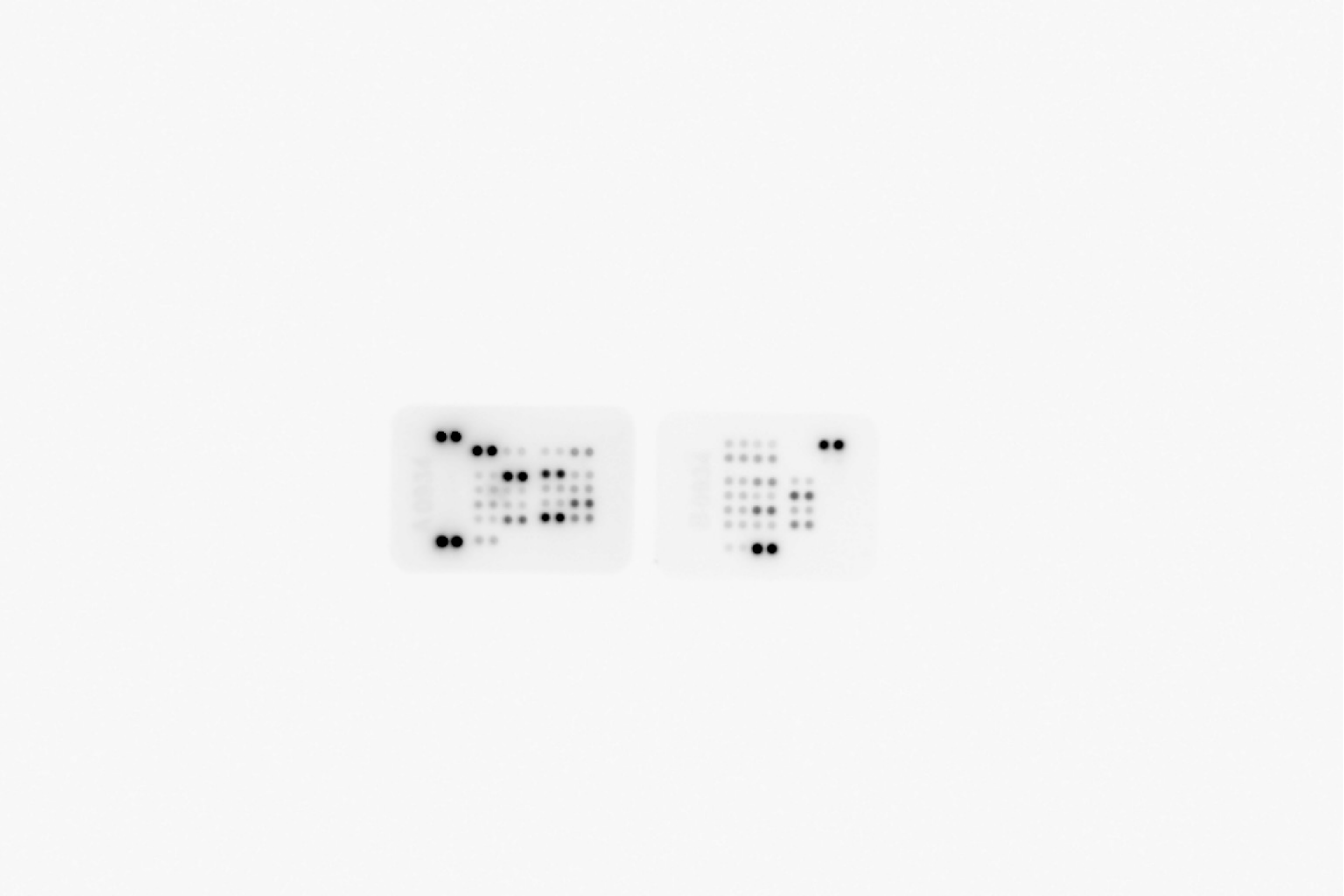

Full and uncropped western blots

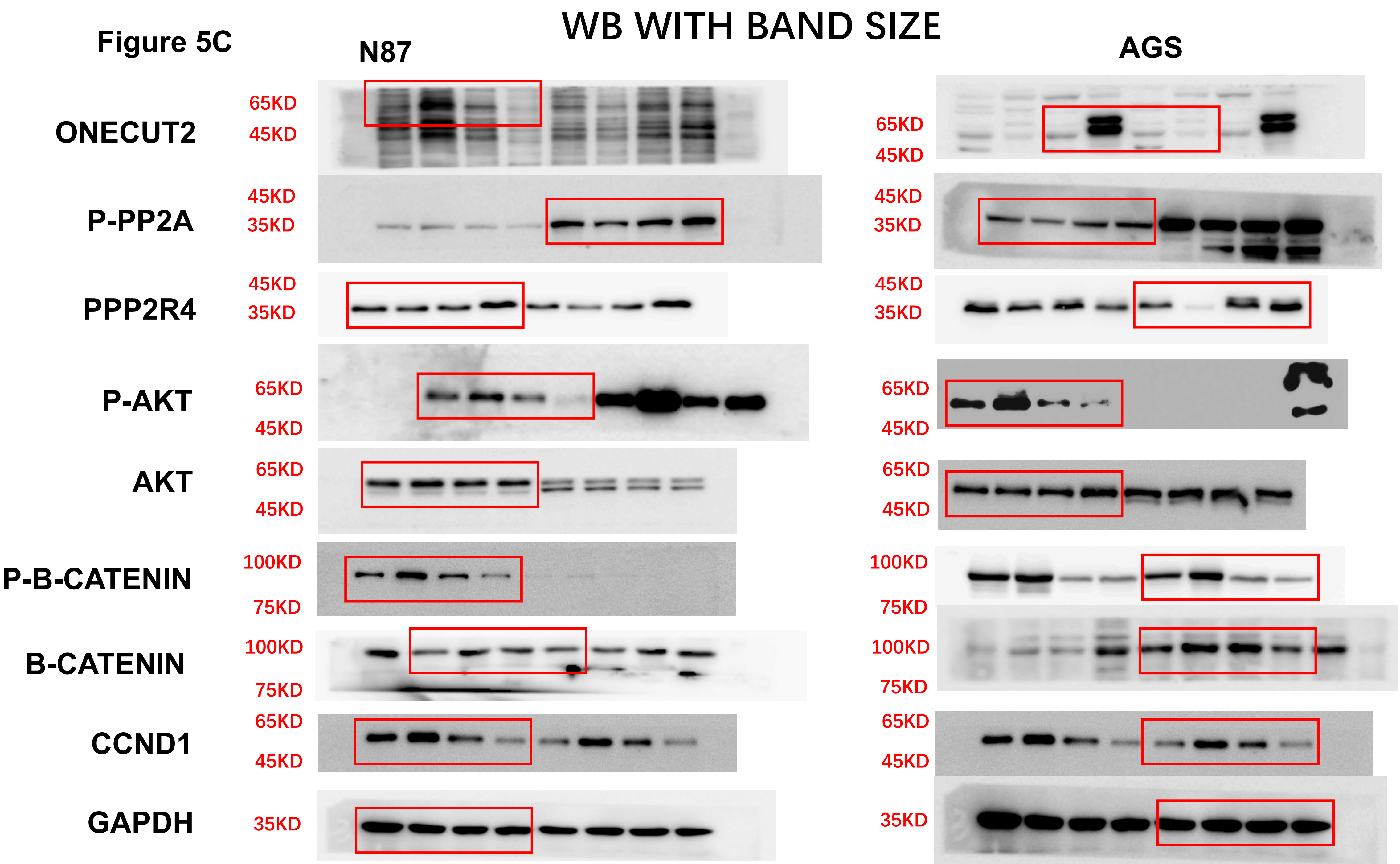
Figure 5C
WB WITH BAND SIZE
N87
AGS
ONECUT2
P-PP2A
PPP2R4
P-AKT
AKT
P-B-CATENIN
B-CATENIN
CCND1
GAPDH
65KD
45KD
35KD
100KD
75KD

Figure 5C

N87 – ONECUT2

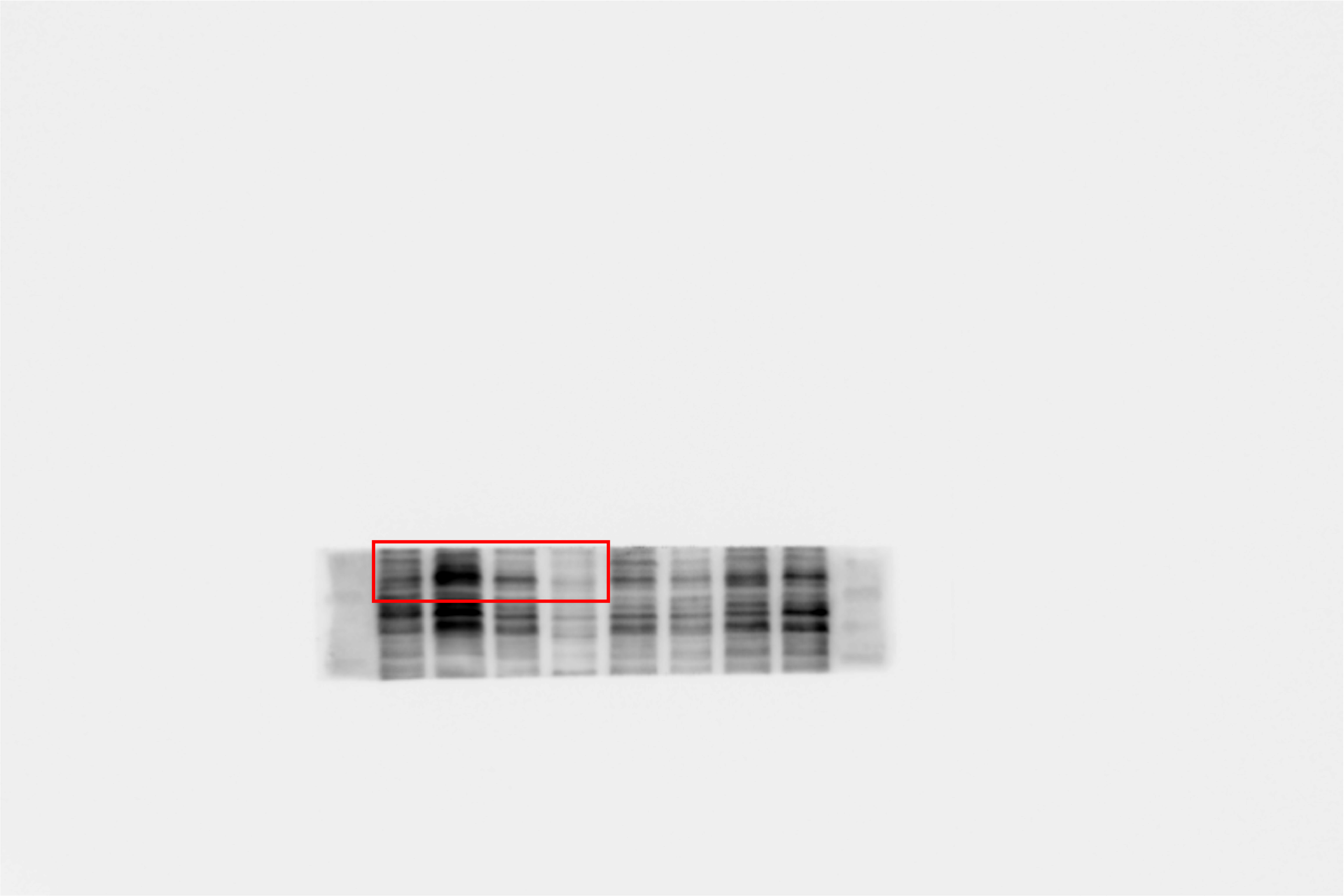

Full and uncropped western blots

Figure 5C

N87 – P- PP2A

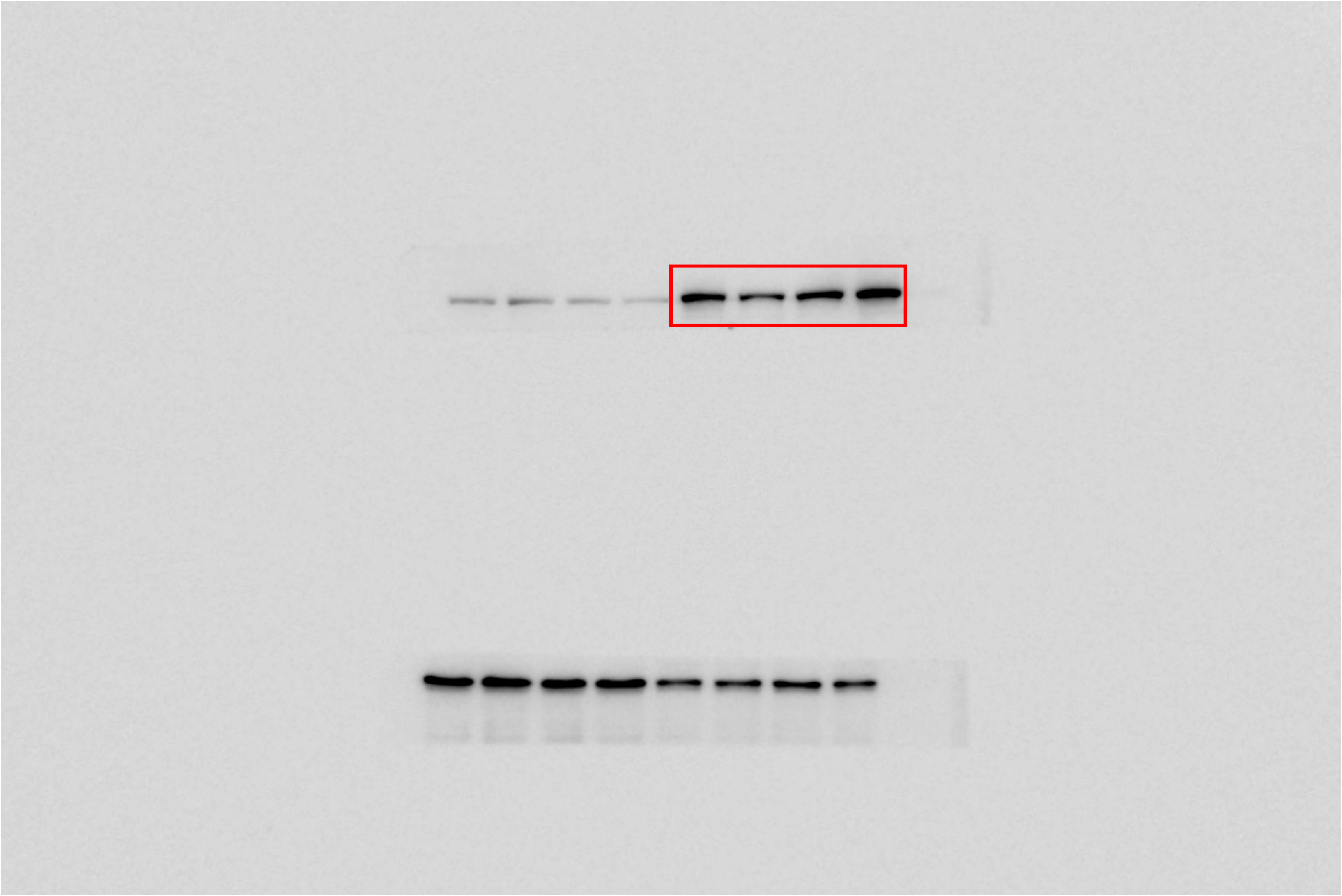

Full and uncropped western blots

Figure 5C

N87 – PPP2R4

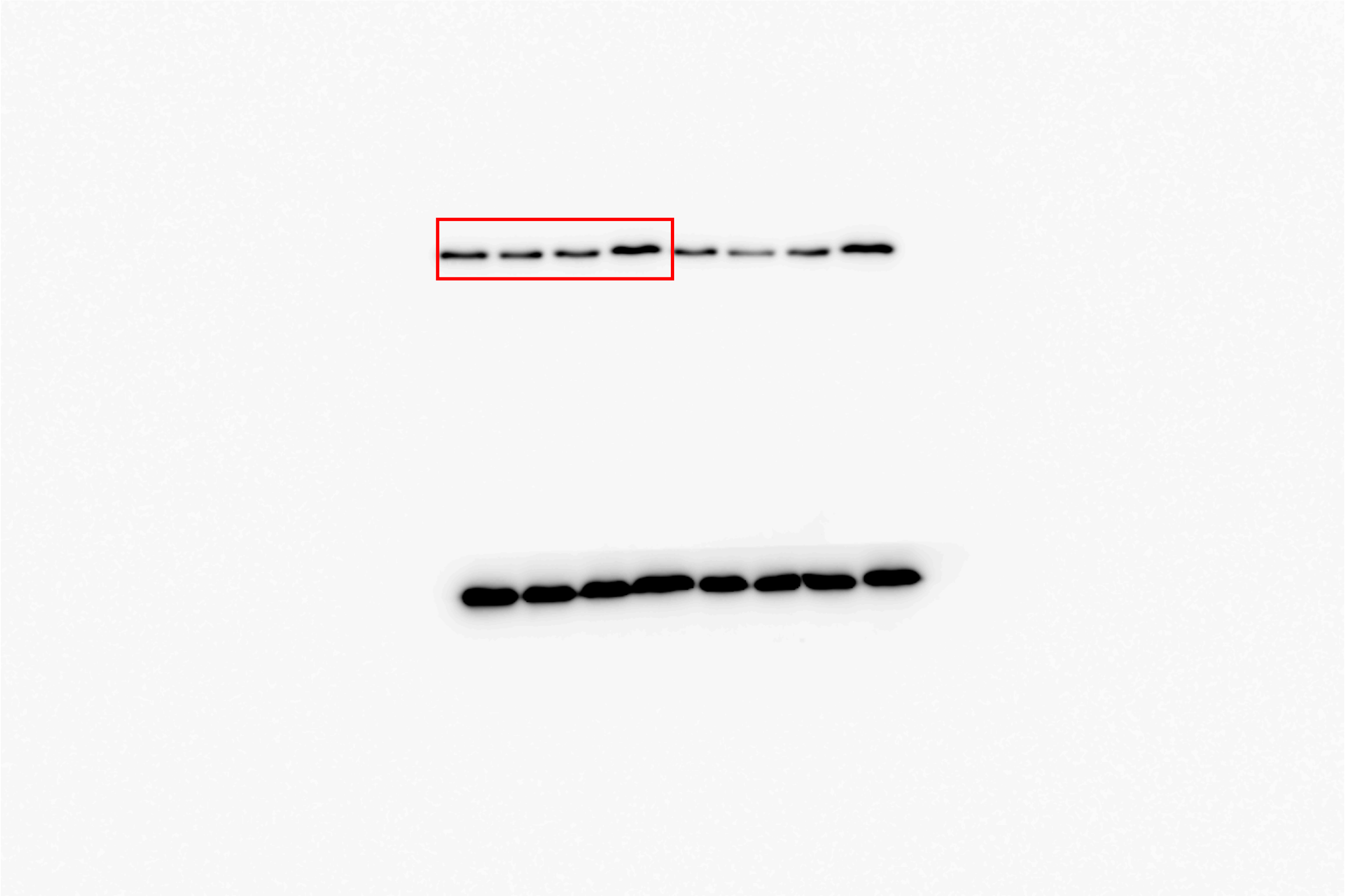

Full and uncropped western blots

Figure 5C

N87 – P- AKT

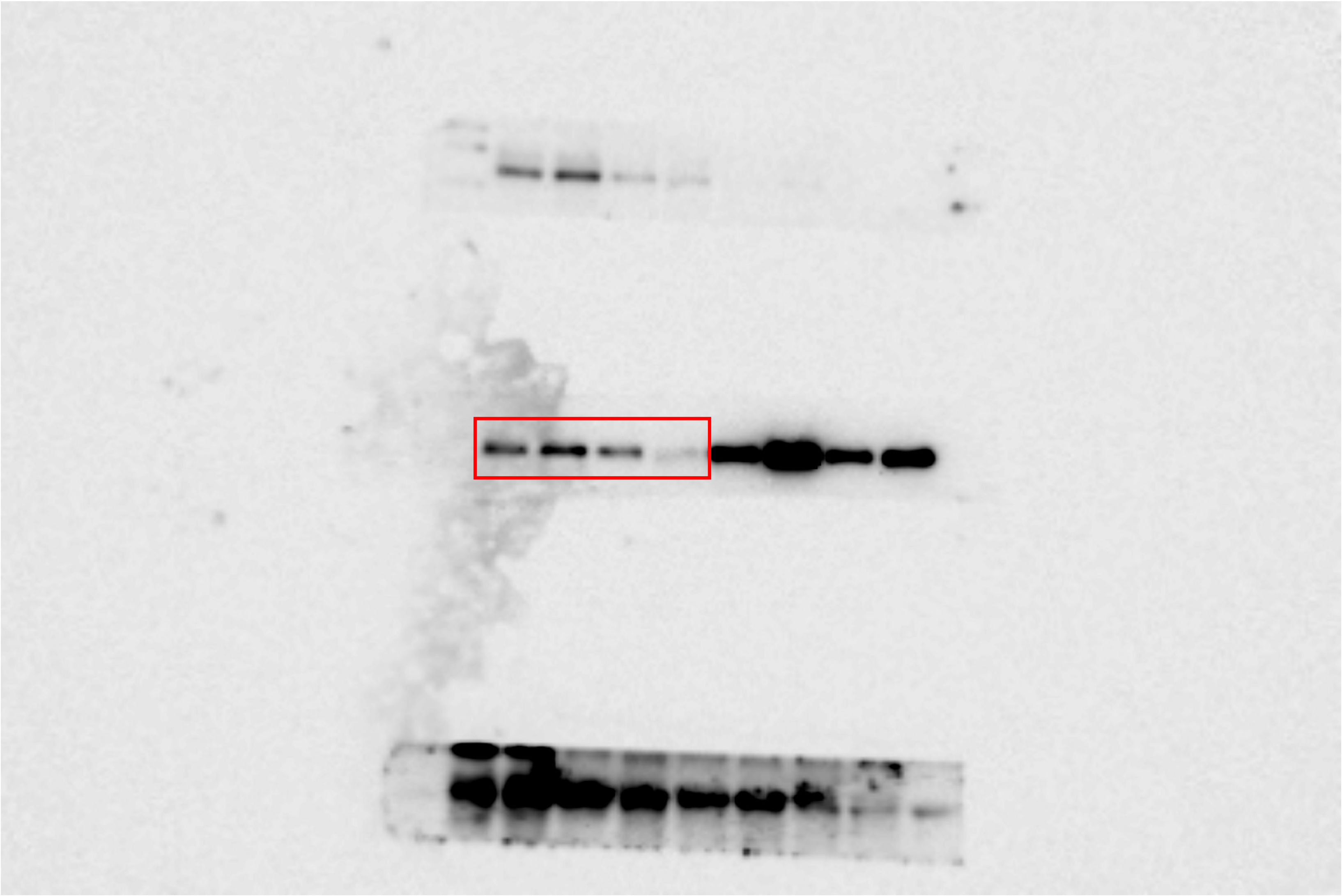

Full and uncropped western blots

Figure 5C

N87 – AKT

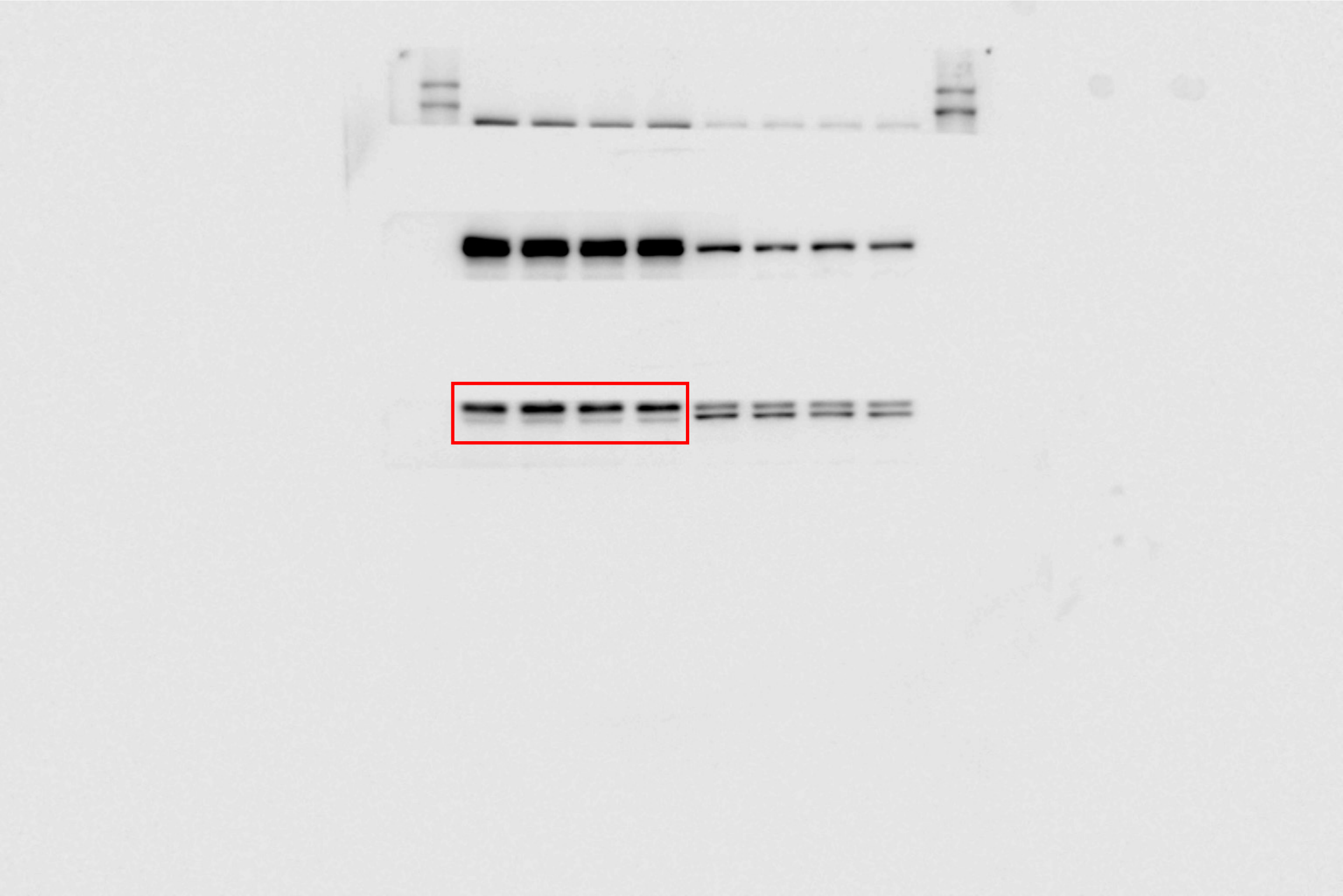

Full and uncropped western blots

Figure 5C

N87 – P-B-CATENIN

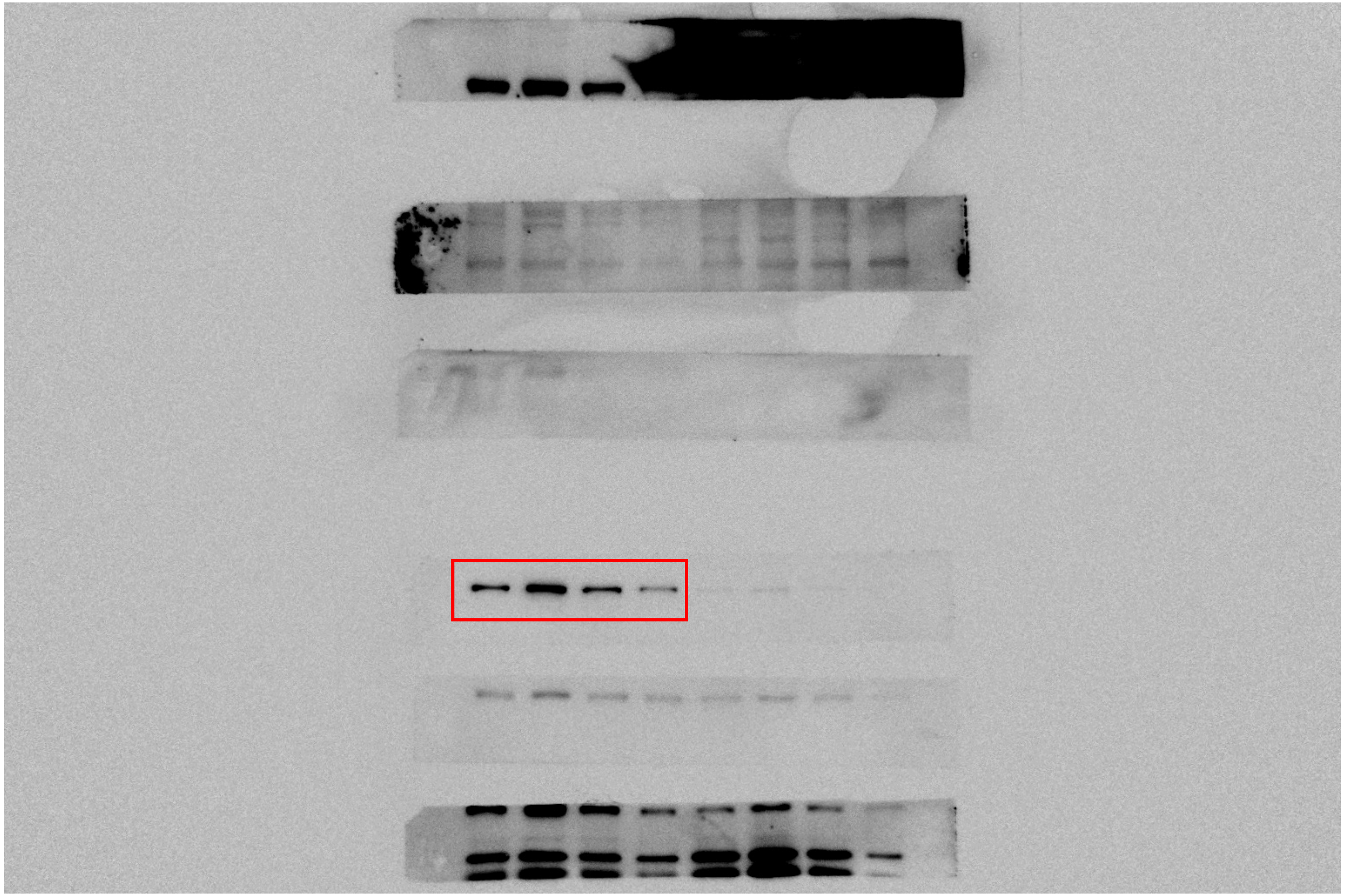

Full and uncropped western blots

Figure 5C

N87 – B-CATENIN

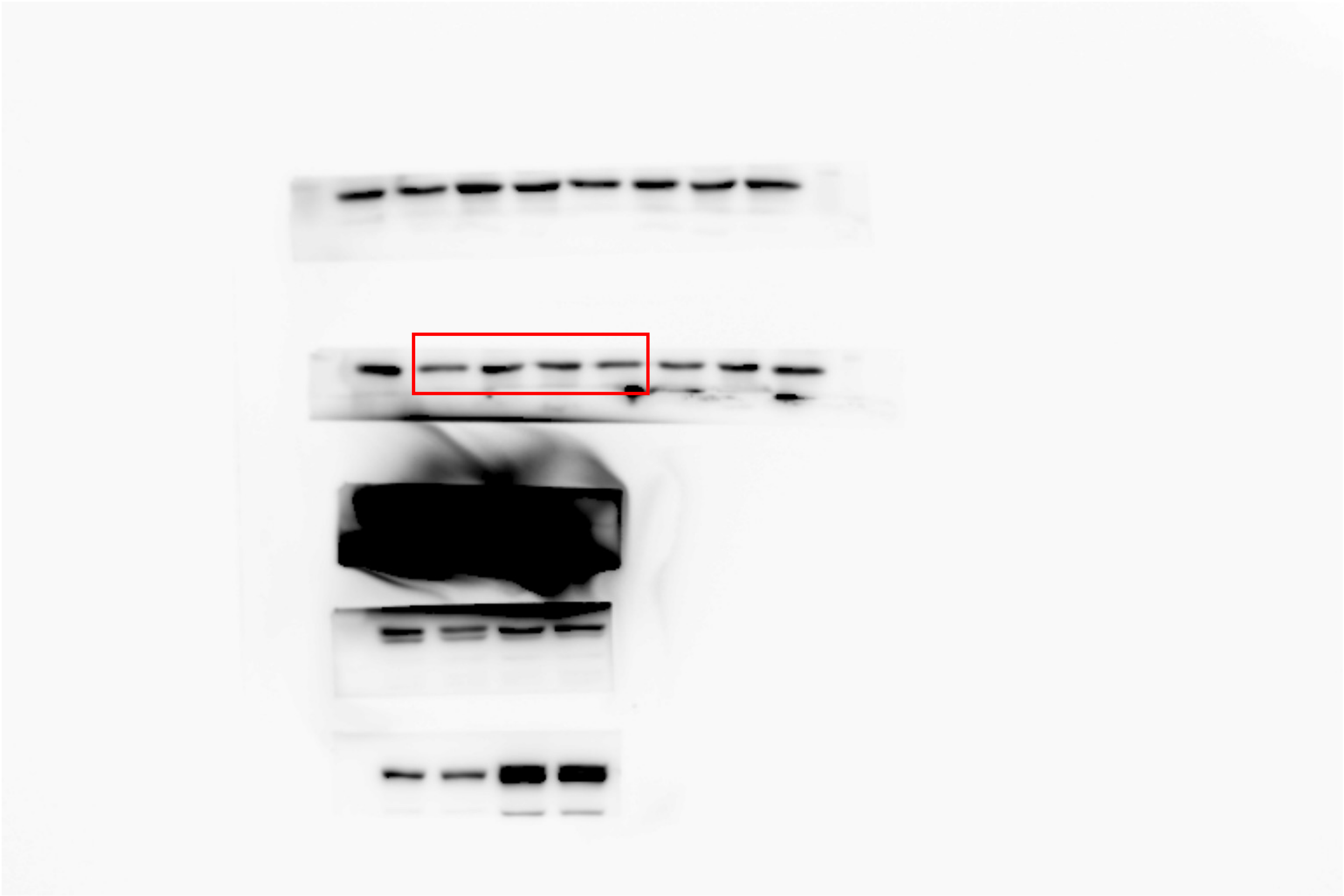

Full and uncropped western blots

Figure 5C

N87 – CCND1

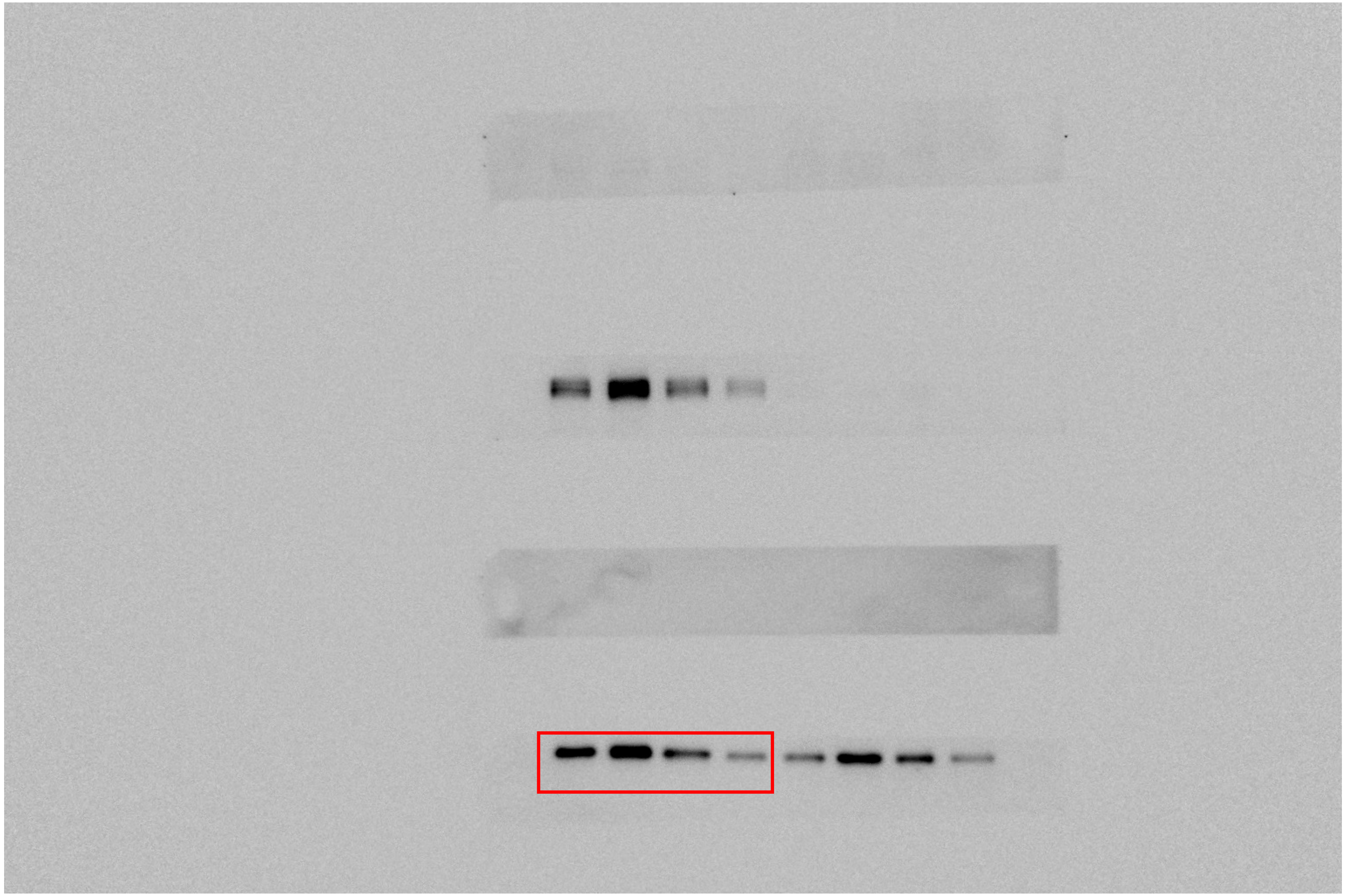

Full and uncropped western blots

Figure 5C

N87 – GAPDH

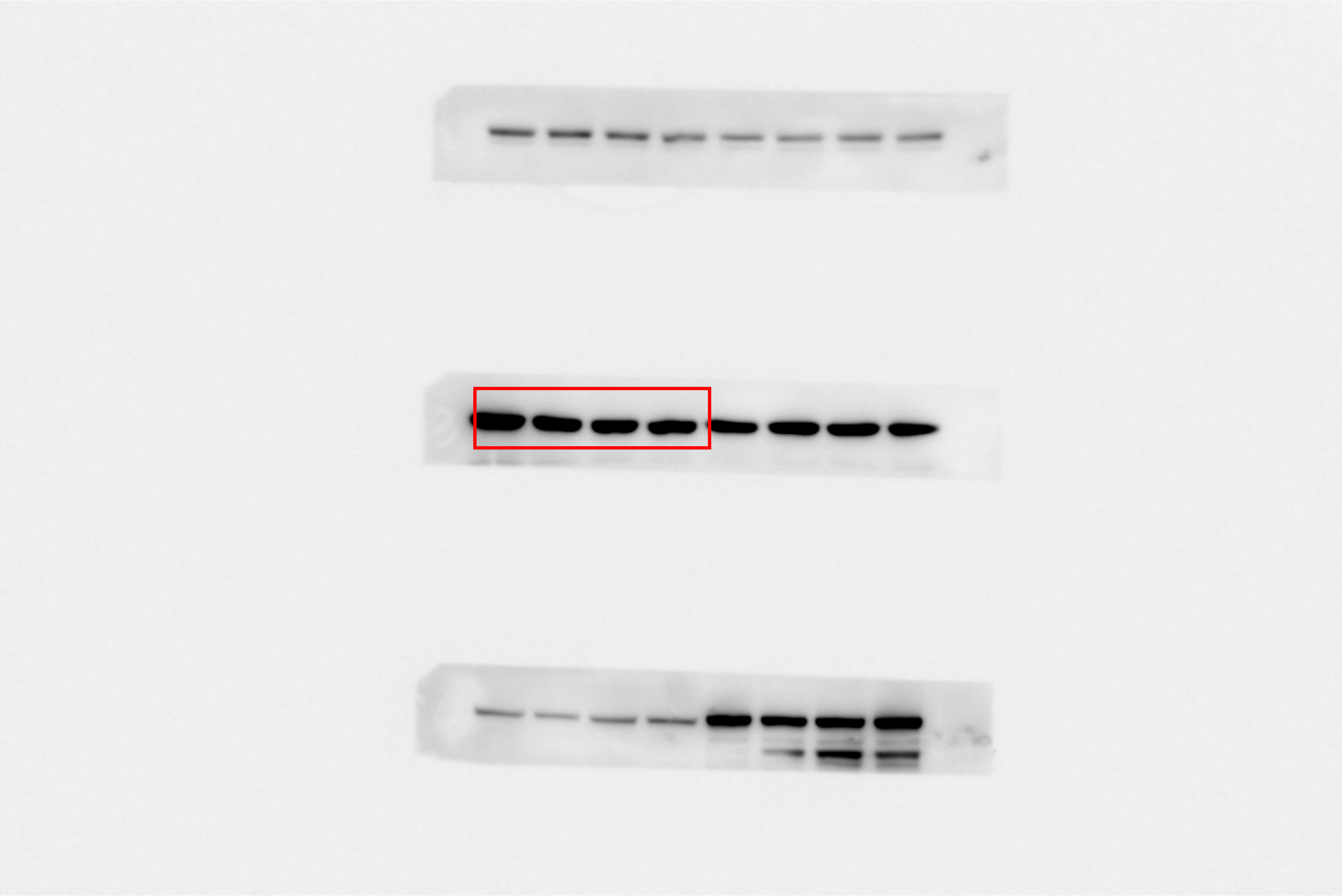

Full and uncropped western blots

Figure 5C

AGS – ONECUT2

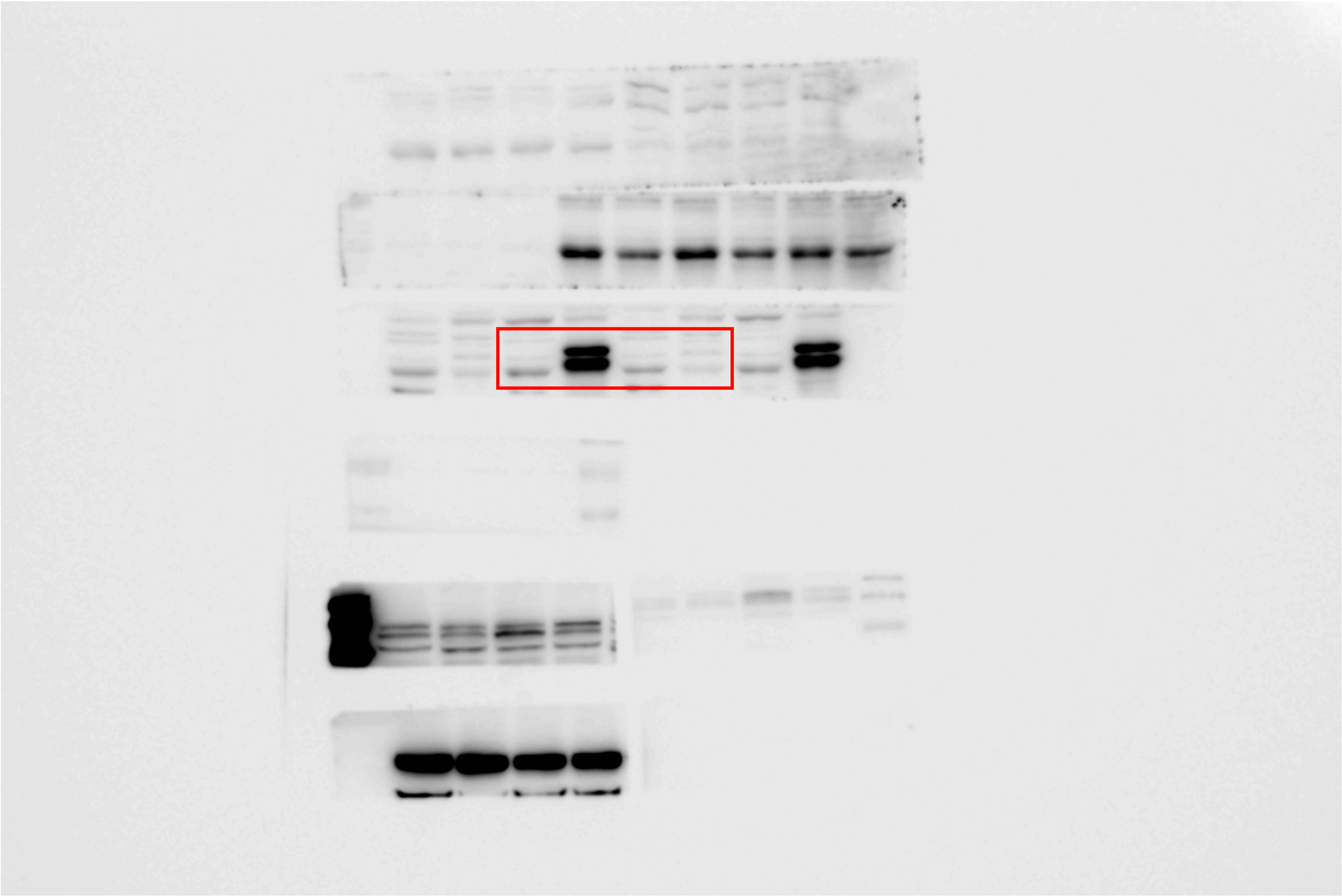

Full and uncropped western blots

Figure 5C

AGS – P- PP2A

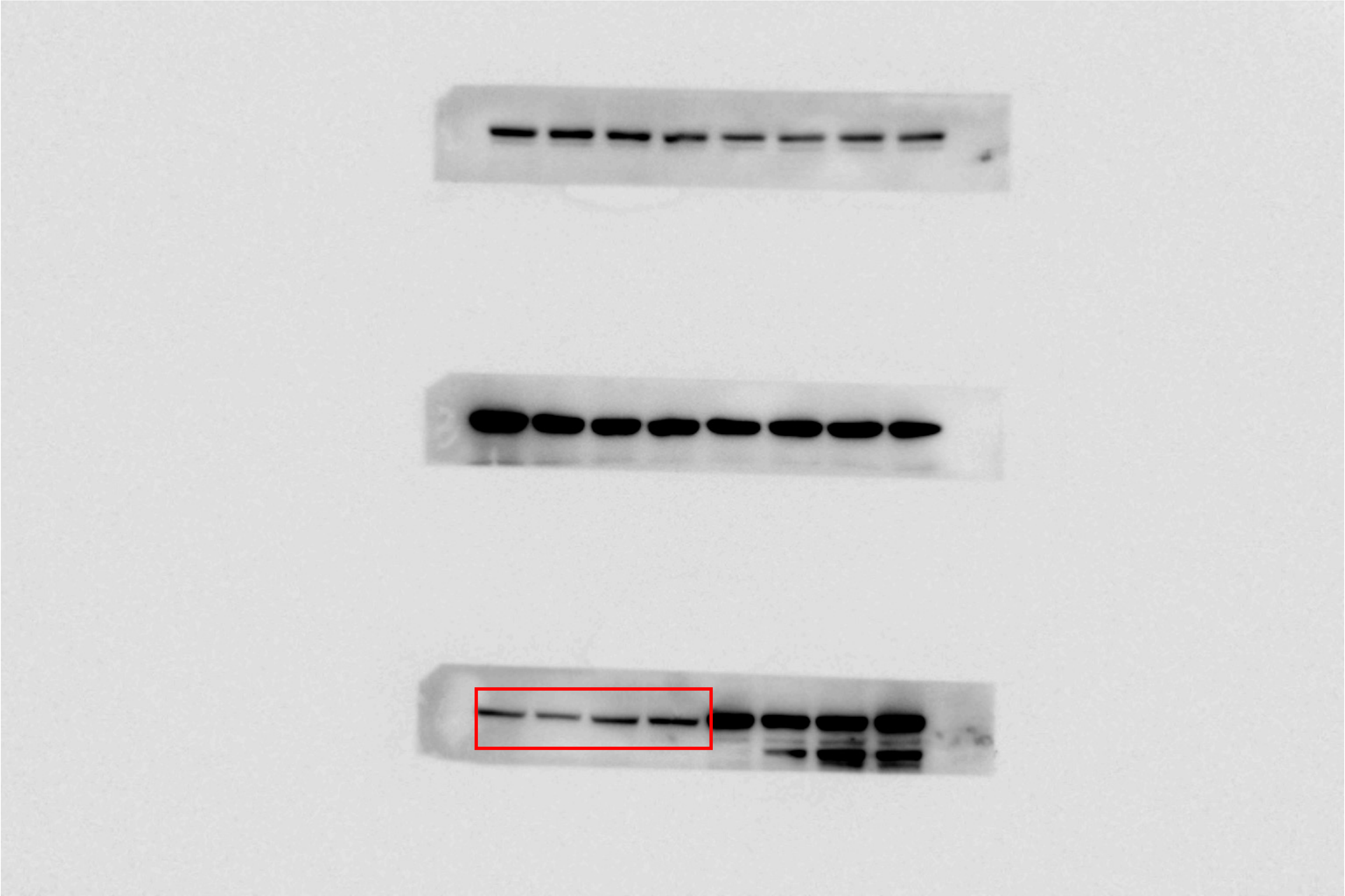

Full and uncropped western blots

Figure 5C

AGS – PP2A

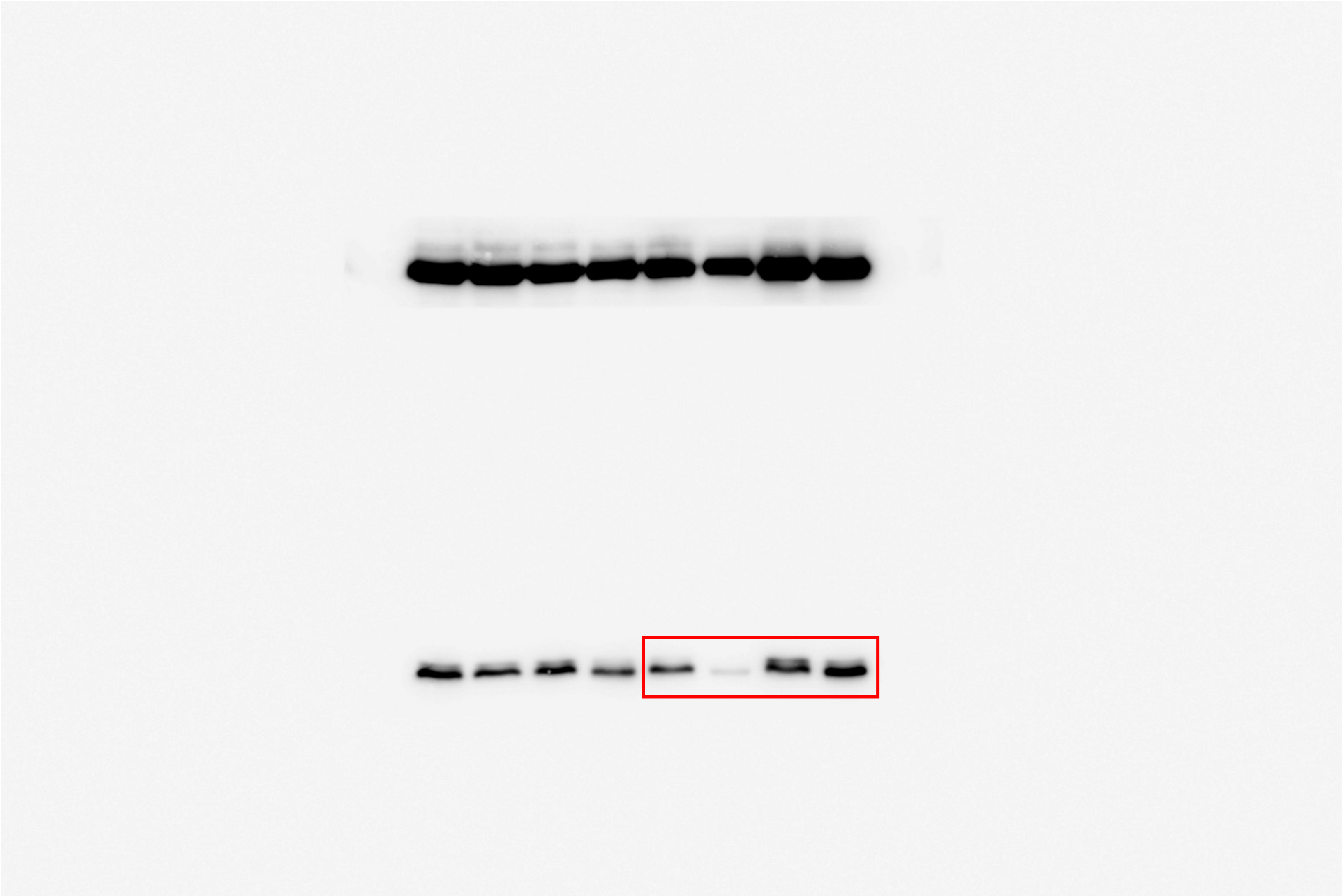

Full and uncropped western blots

Figure 5C

AGS – P- AKT

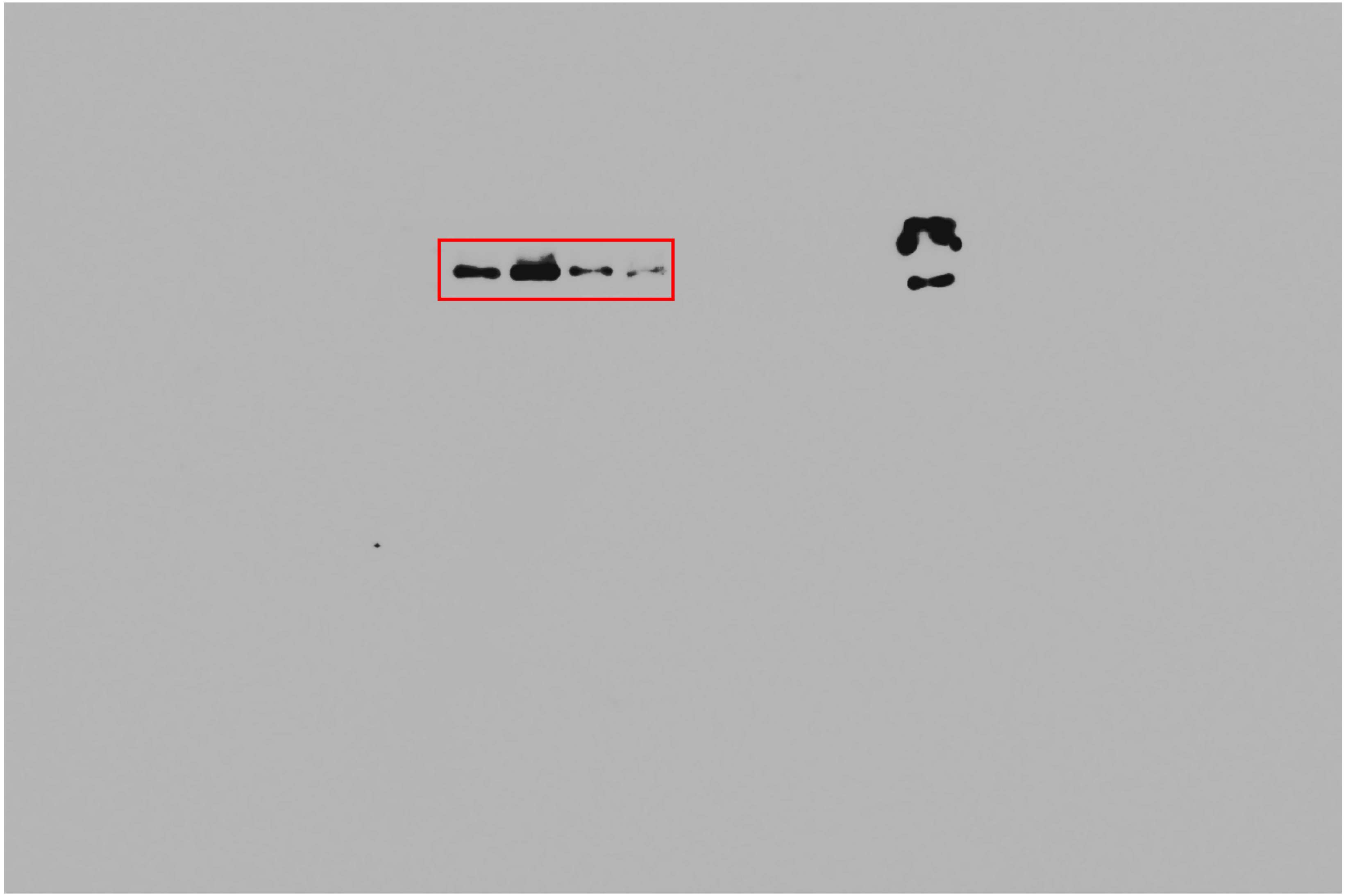

Full and uncropped western blots

Figure 5C

AGS – AKT

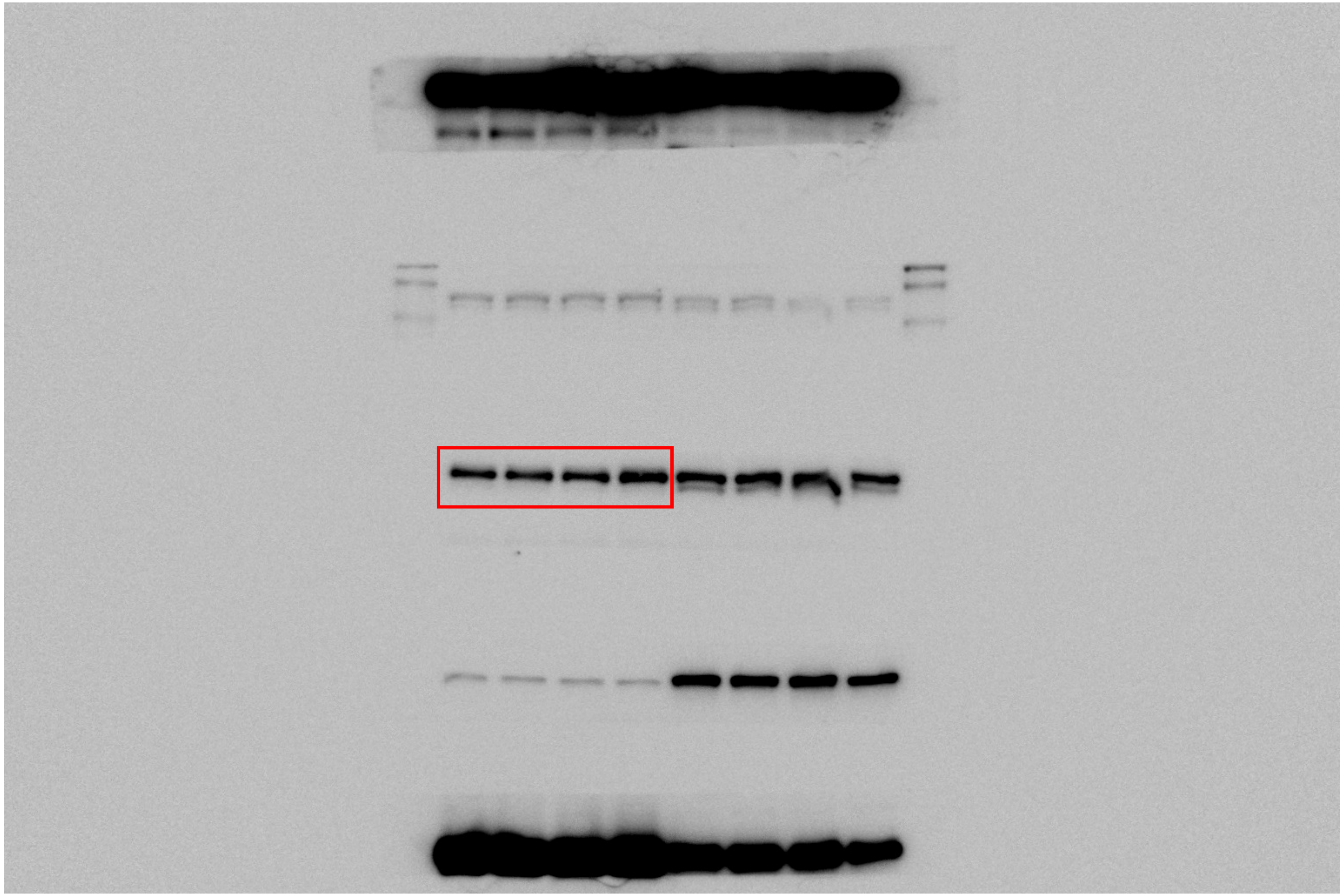

Full and uncropped western blots

Figure 5C

AGS – P- B-CATENIN

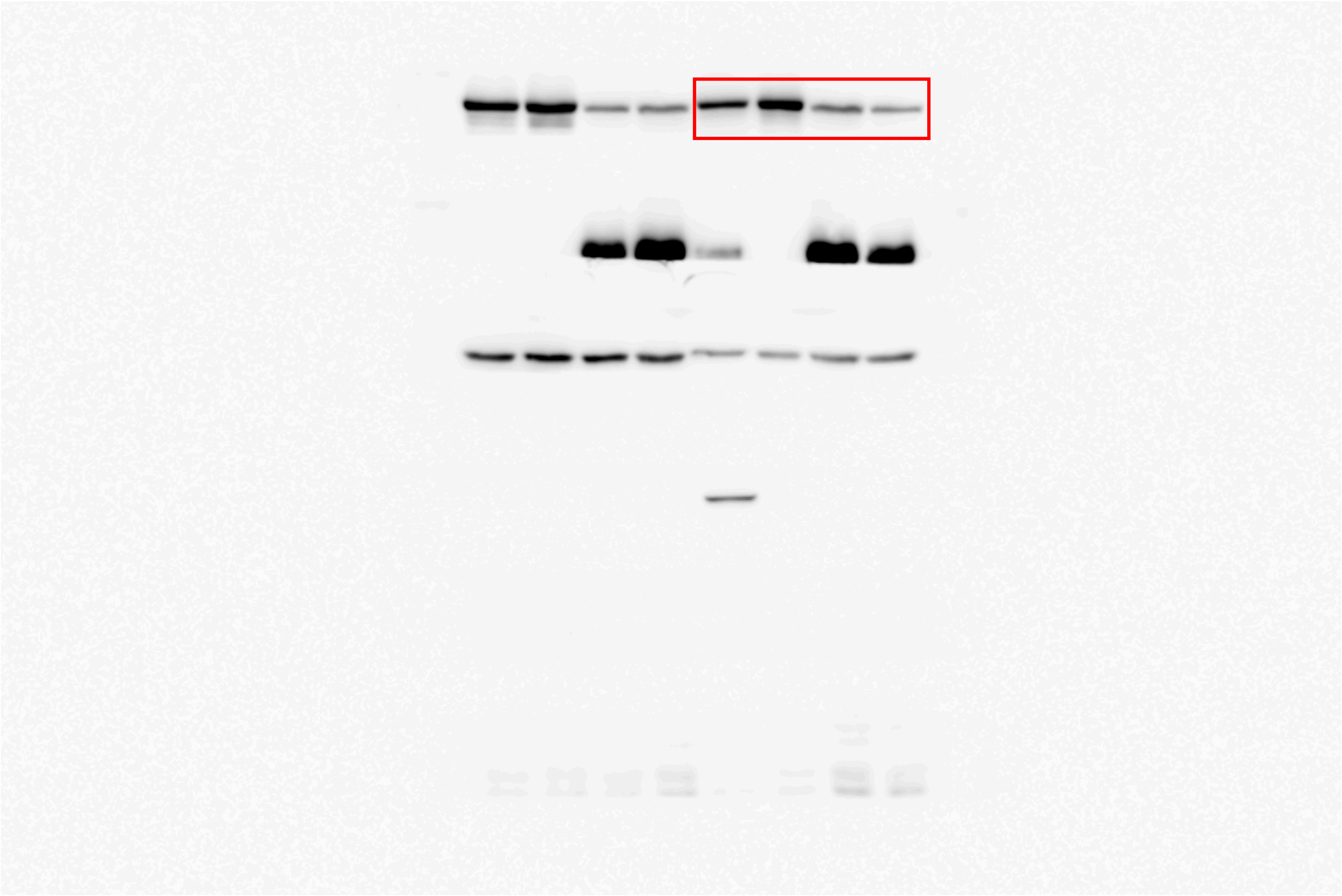

Full and uncropped western blots

Figure 5C

AGS – B-CATENIN

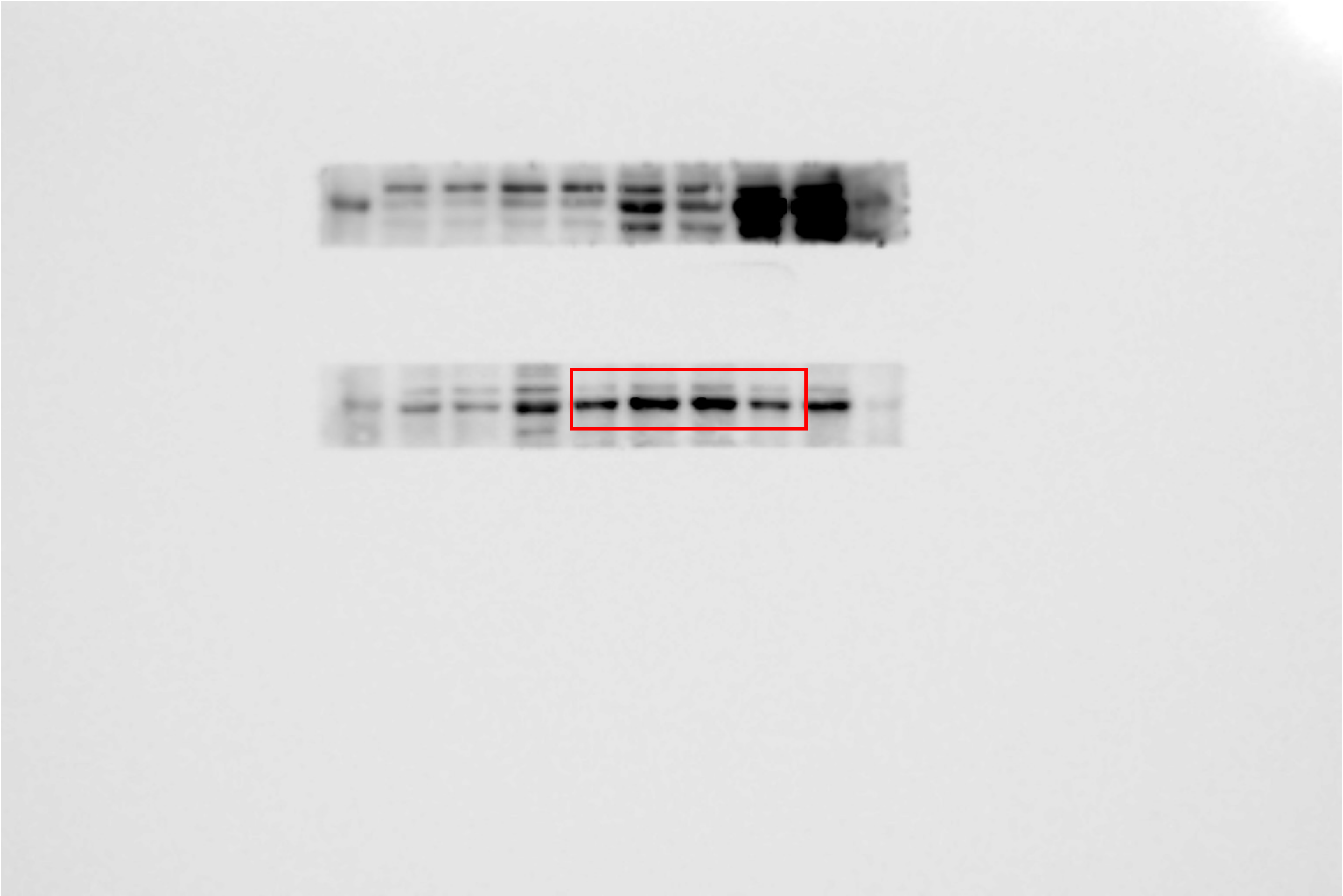

Full and uncropped western blots

Figure 5C

AGS – CCND1

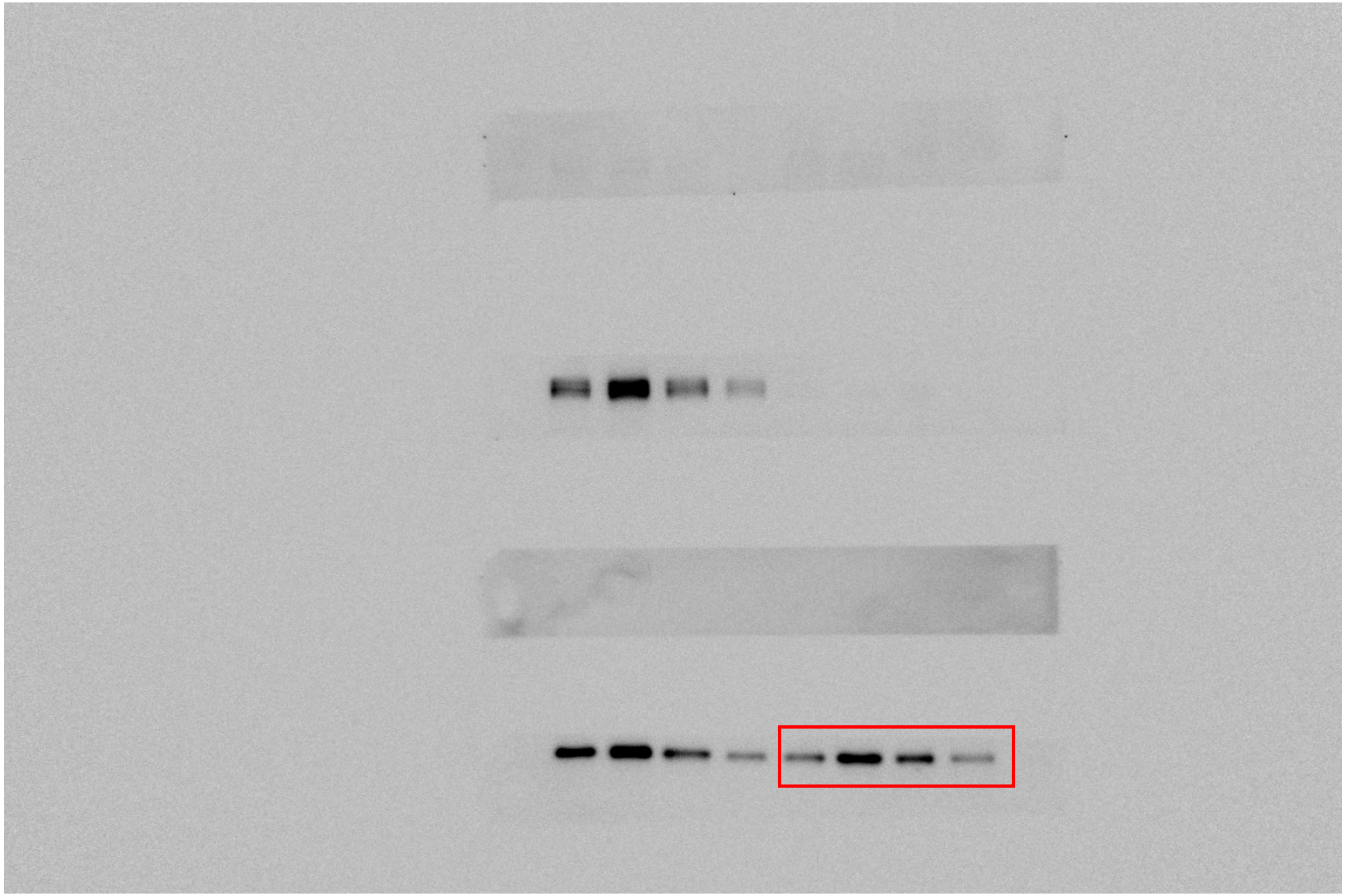

Full and uncropped western blots

Figure 5C

AGS – GAPDH

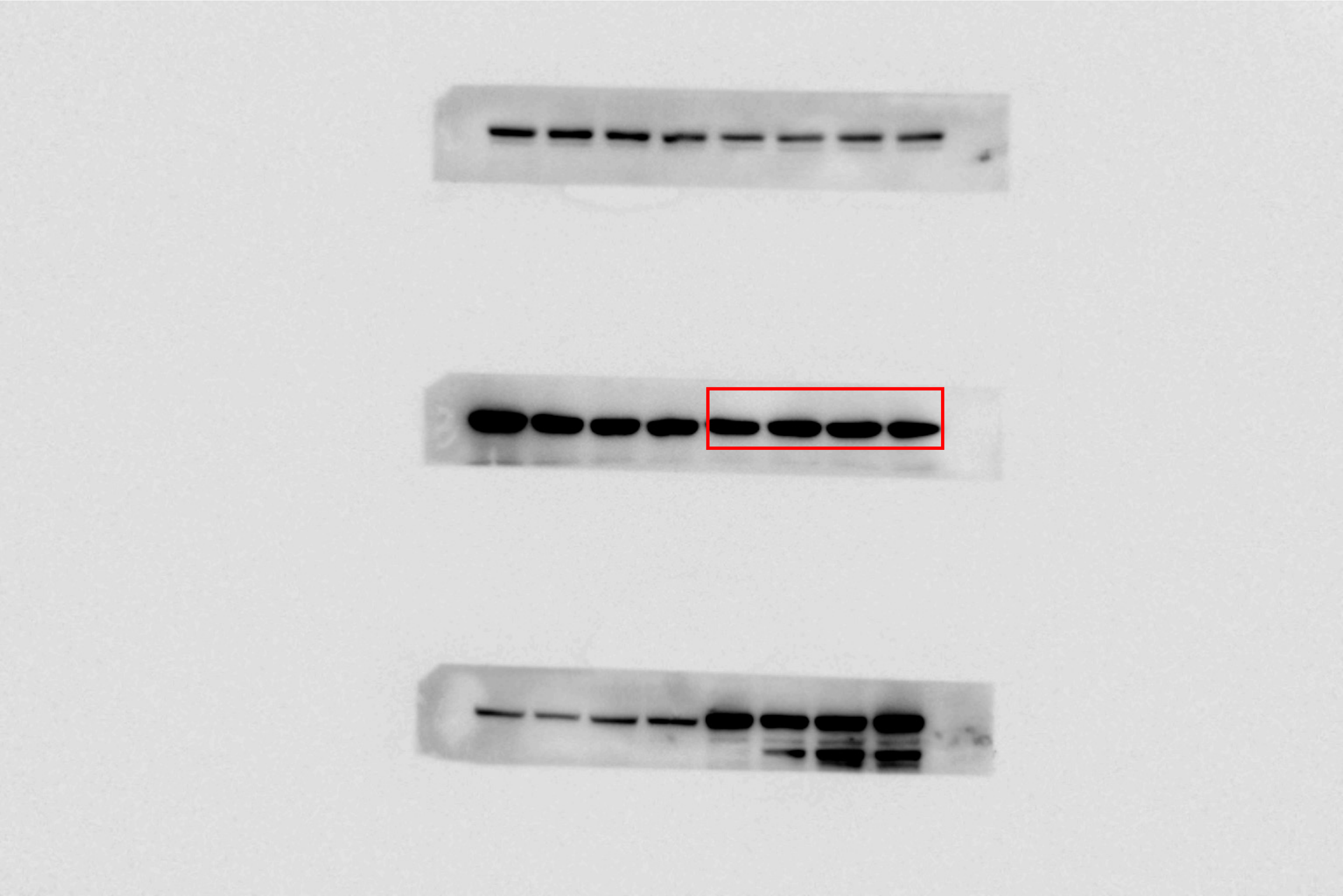

Full and uncropped western blots

Figure 5F

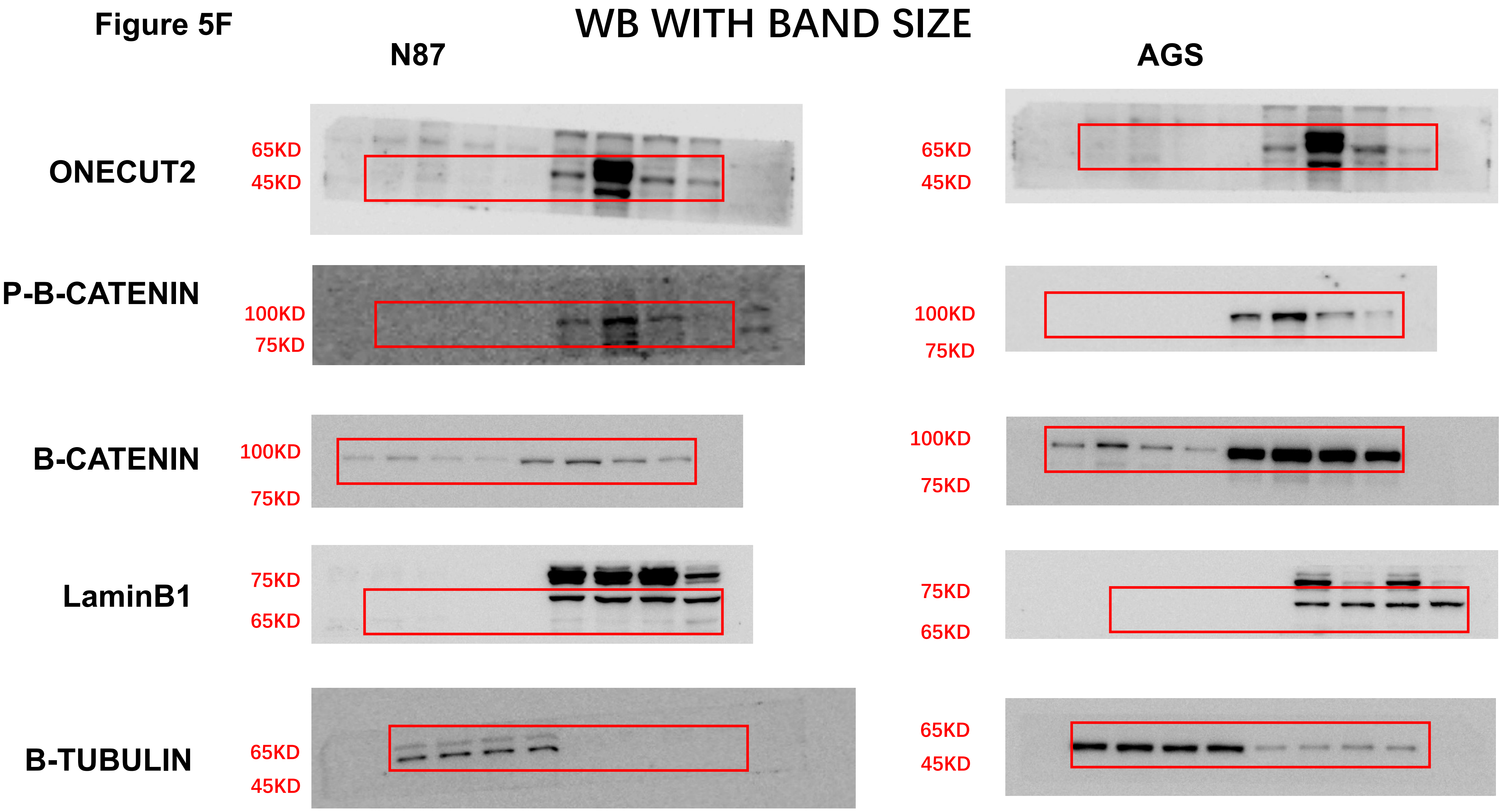
WB WITH BAND SIZE
N87
AGS
ONECUT2
65KD
45KD
65KD
45KD
P-B-CATENIN
100KD
75KD
100KD
75KD
B-CATENIN
100KD
75KD
100KD
75KD
LaminB1
75KD
65KD
75KD
65KD
B-TUBULIN
65KD
45KD
65KD
45KD

Figure 5F

N87 – ONECUT2

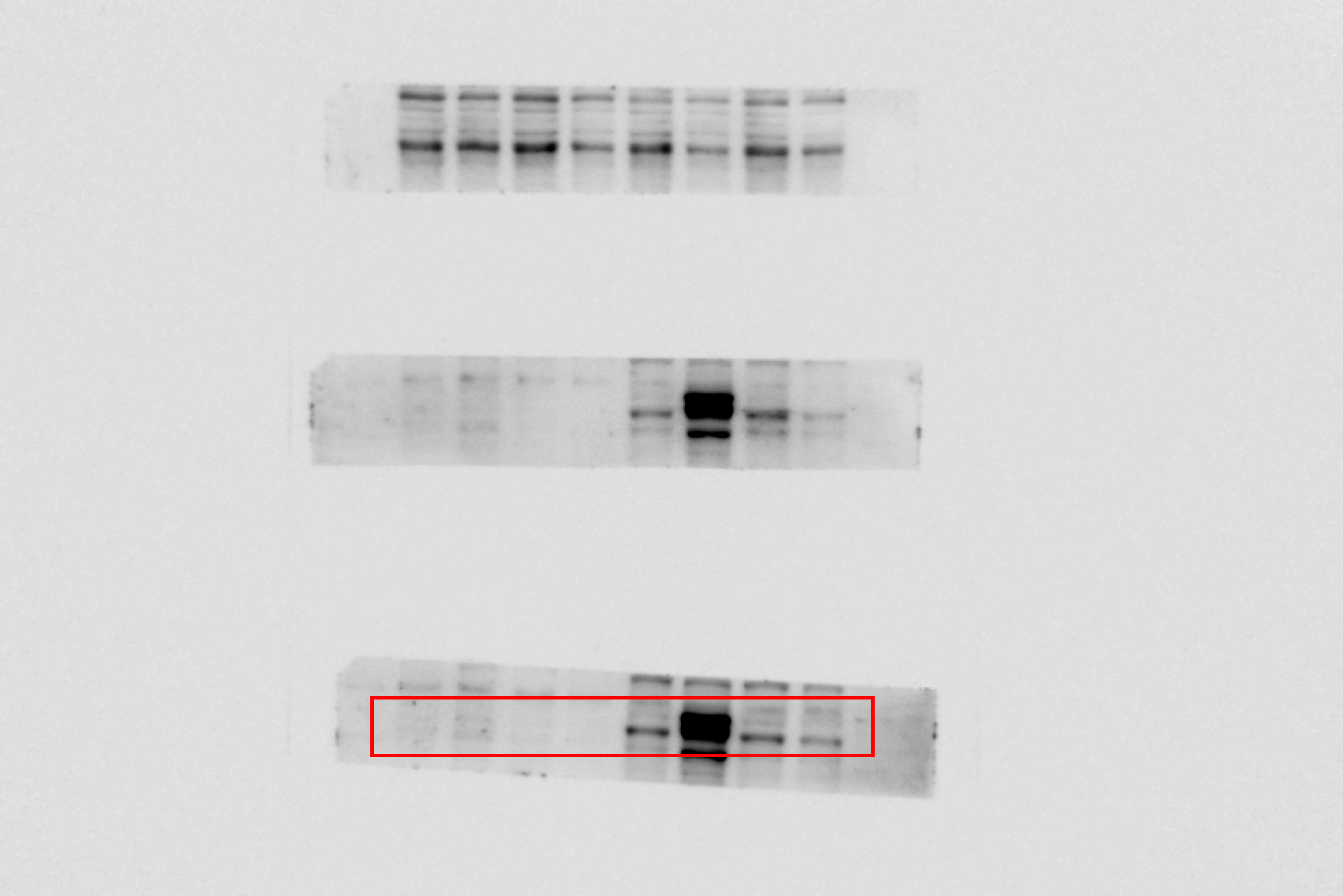

Full and uncropped western blots

Figure 5F

N87 – P- B-CATENIN

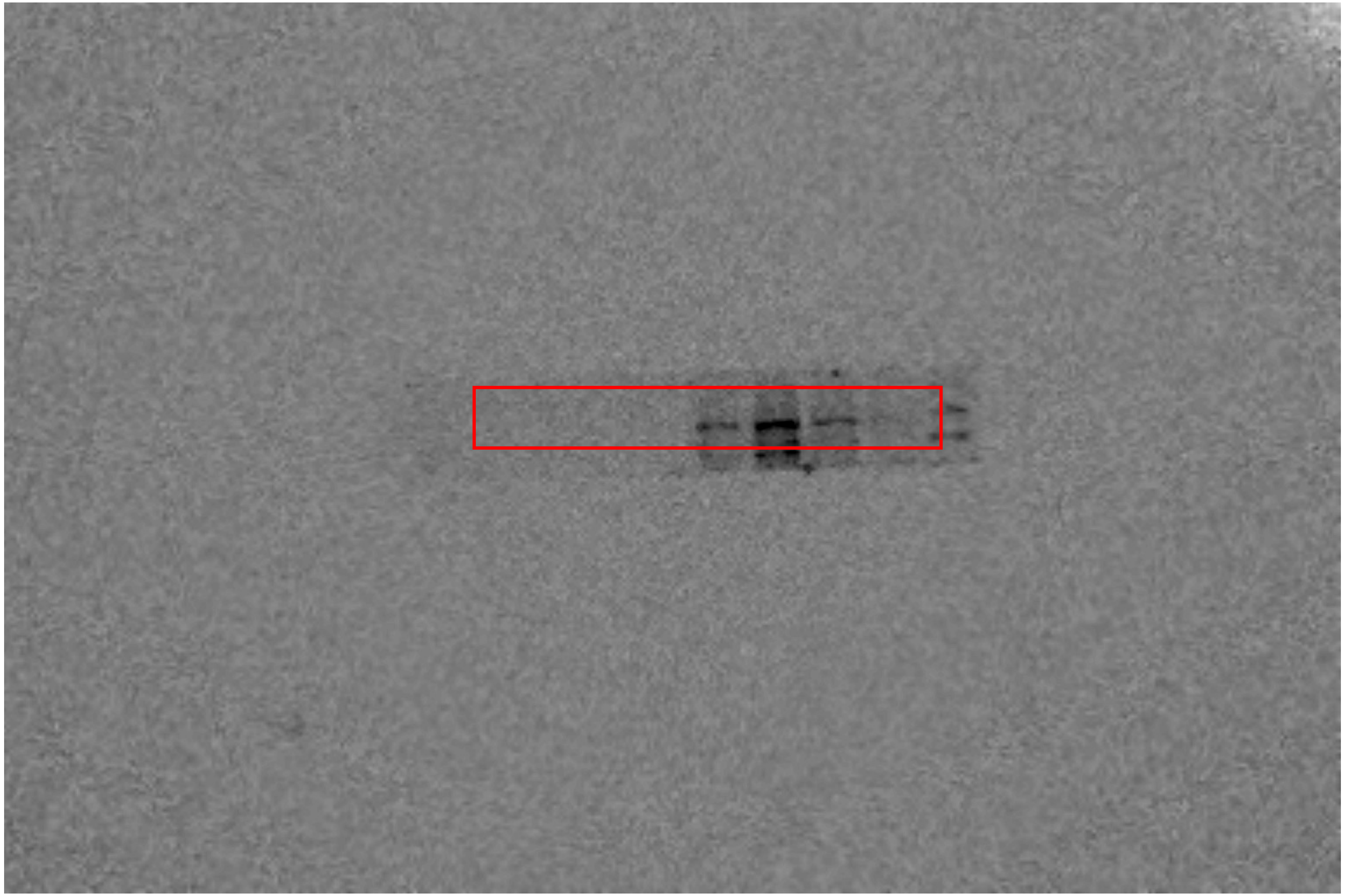

Full and uncropped western blots

Figure 5F

N87 – B- CATENIN

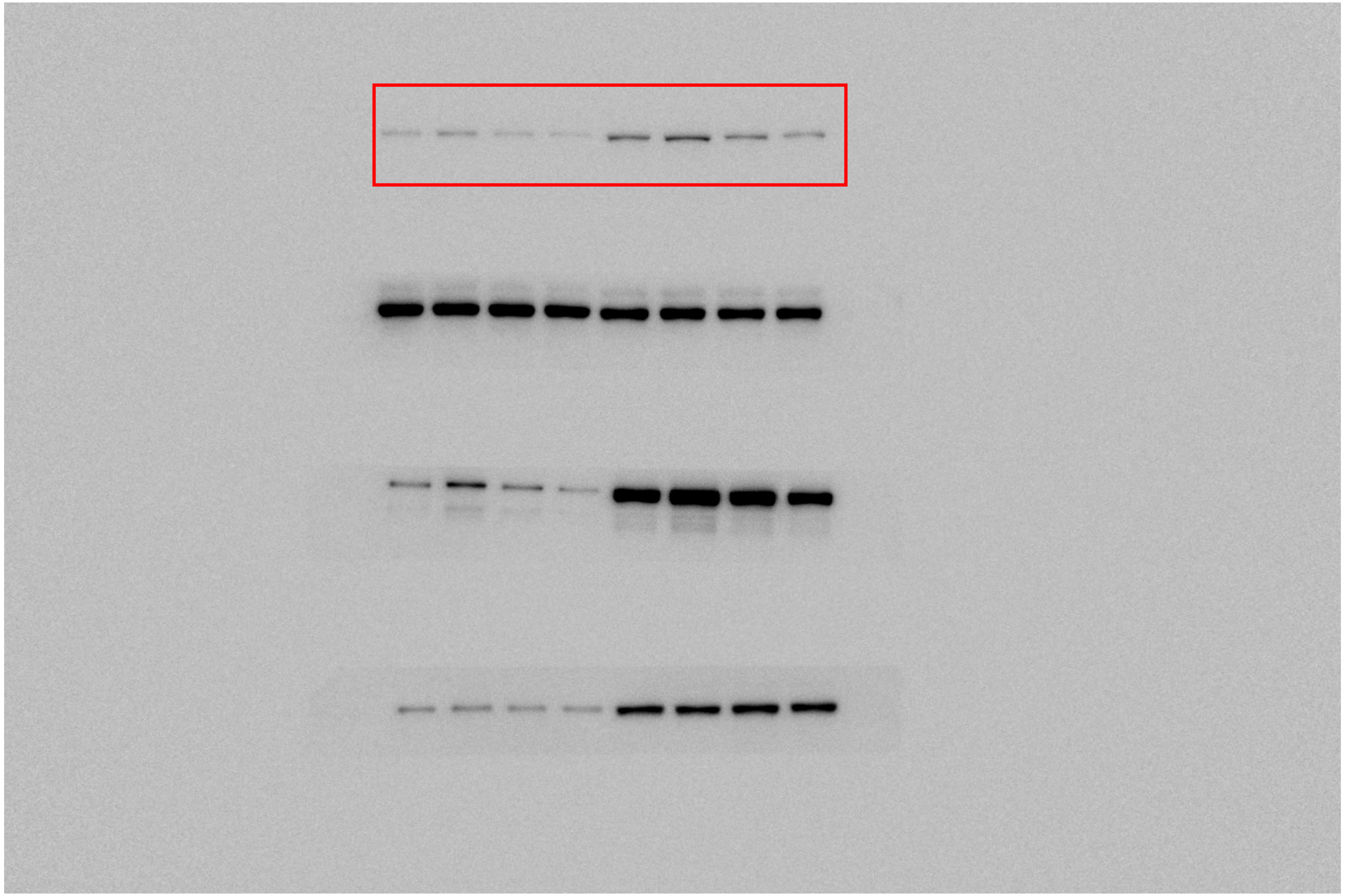

Full and uncropped western blots

Figure 5F

N87 – LaminB1

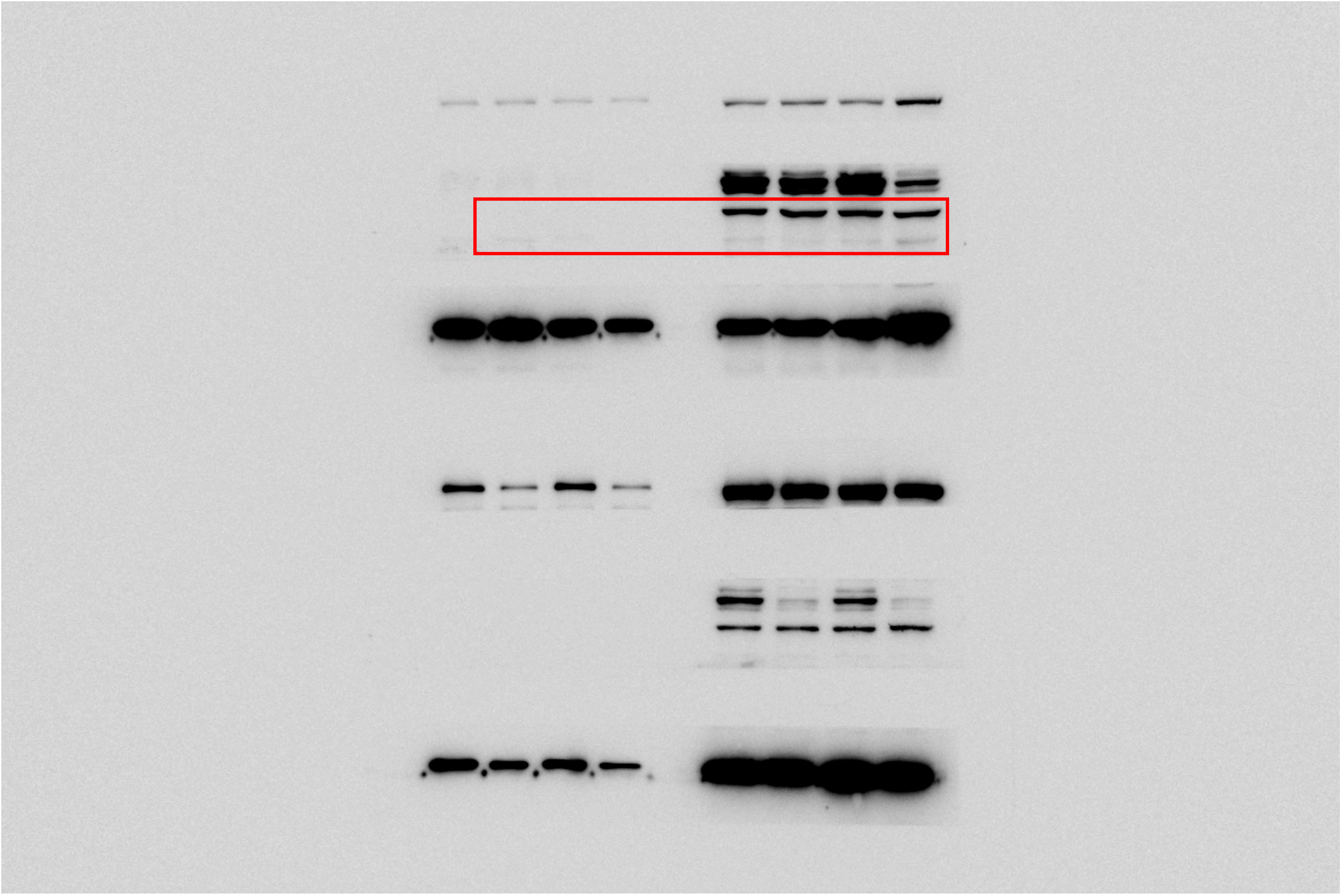

Full and uncropped western blots

Figure 5F

N87 – B-TUBULIN

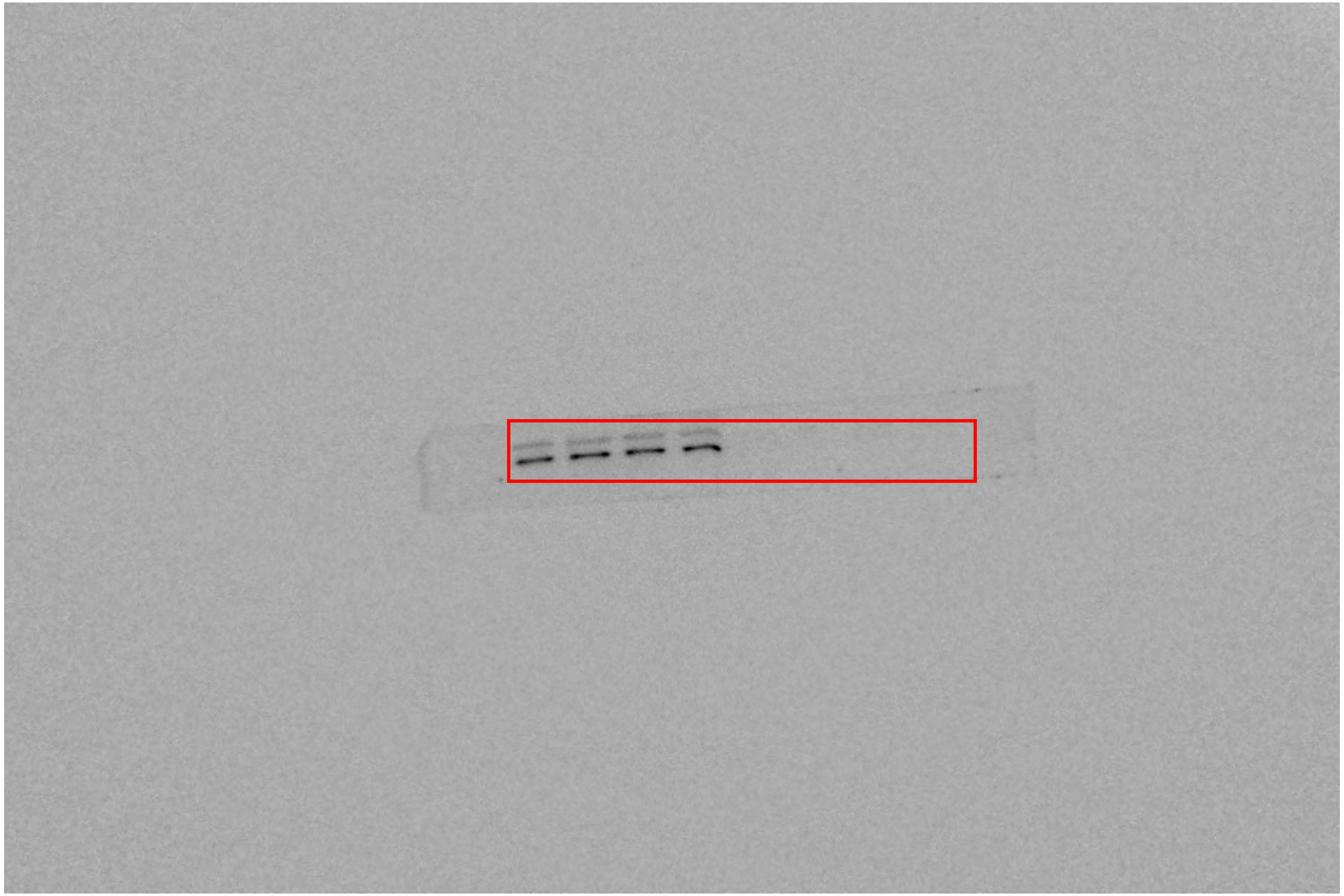

Full and uncropped western blots

Figure 5F

AGS – ONECUT2

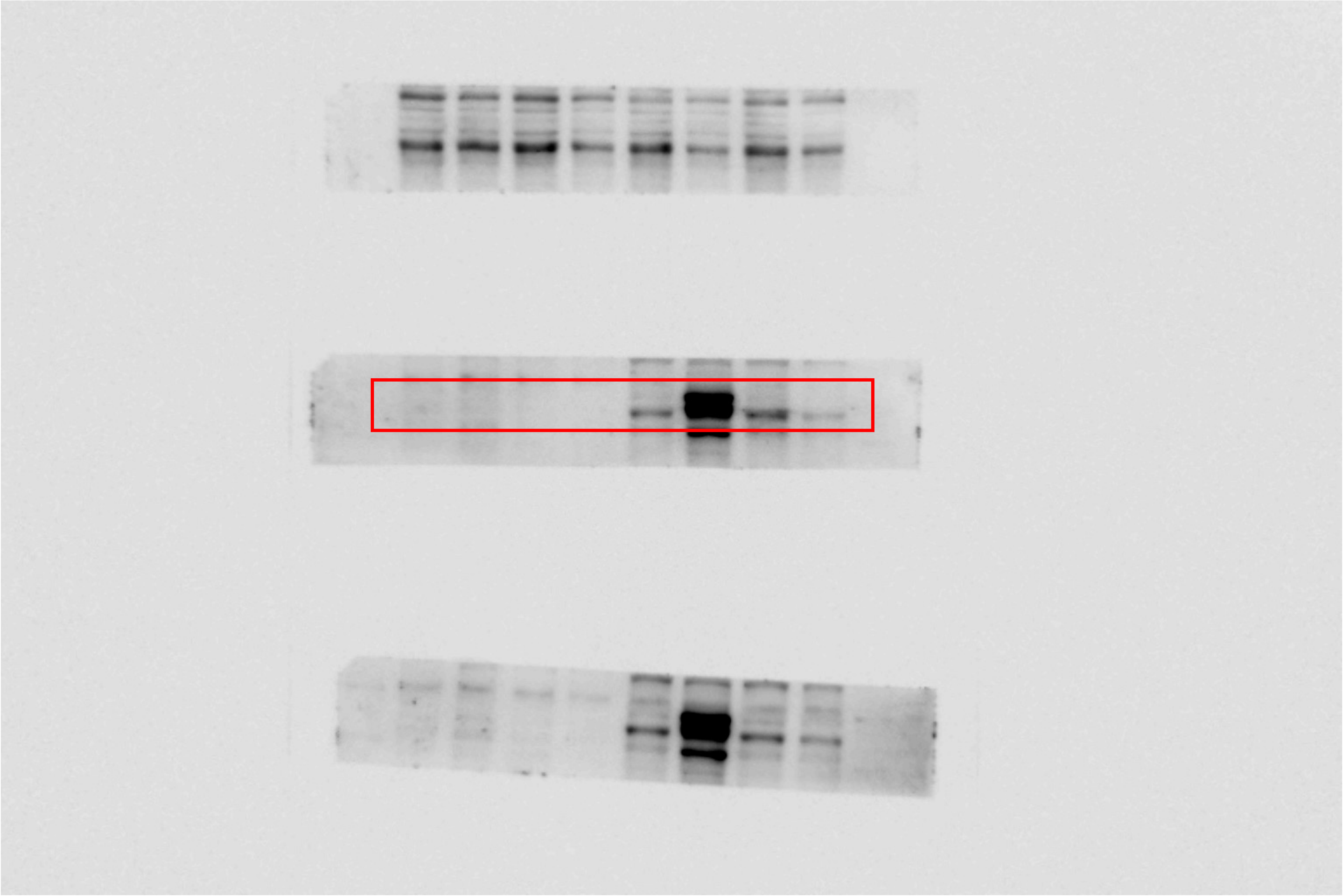

Full and uncropped western blots

Figure 5F

AGS – P- B-CATENIN

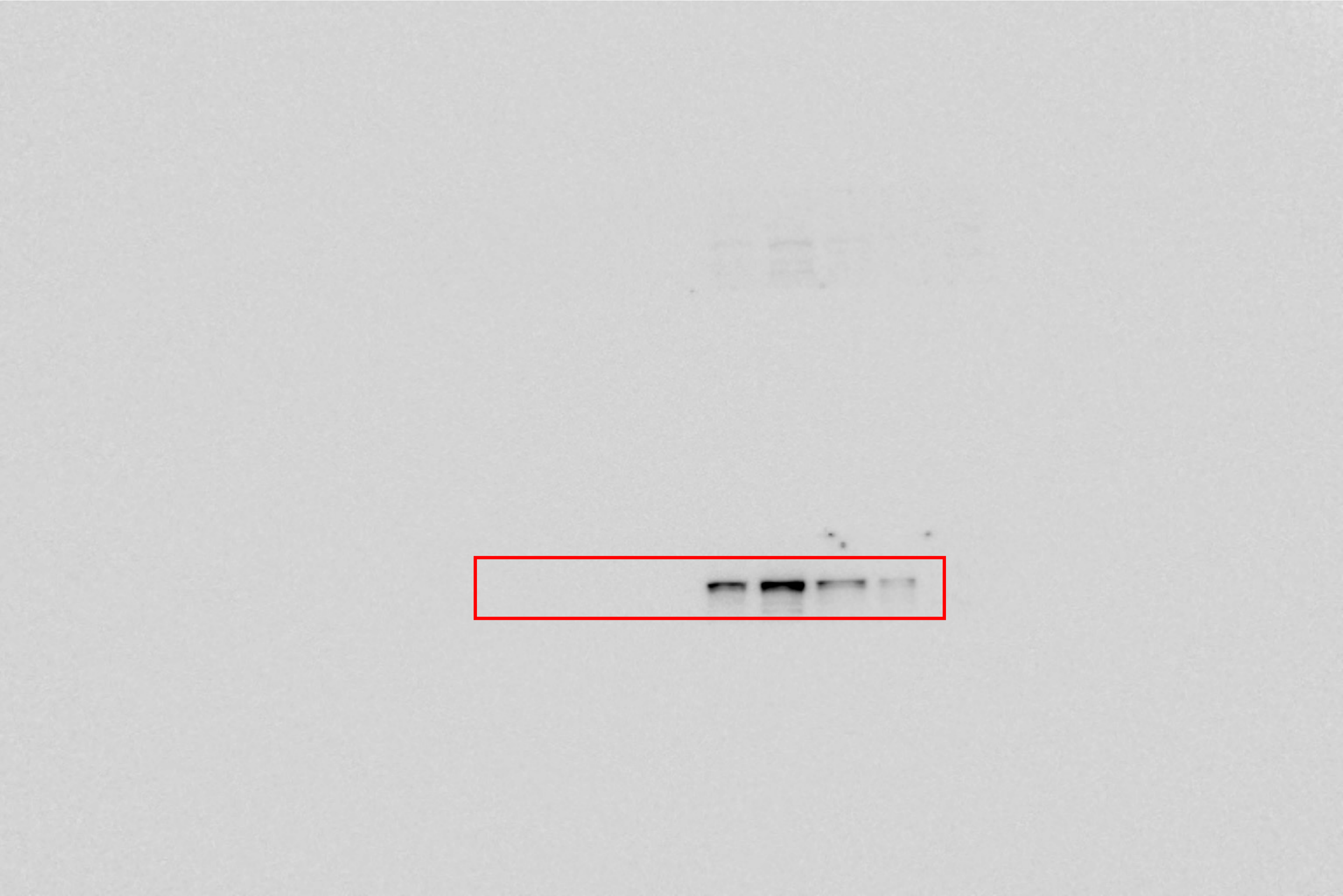

Full and uncropped western blots

Figure 5F

AGS – B- CATENIN

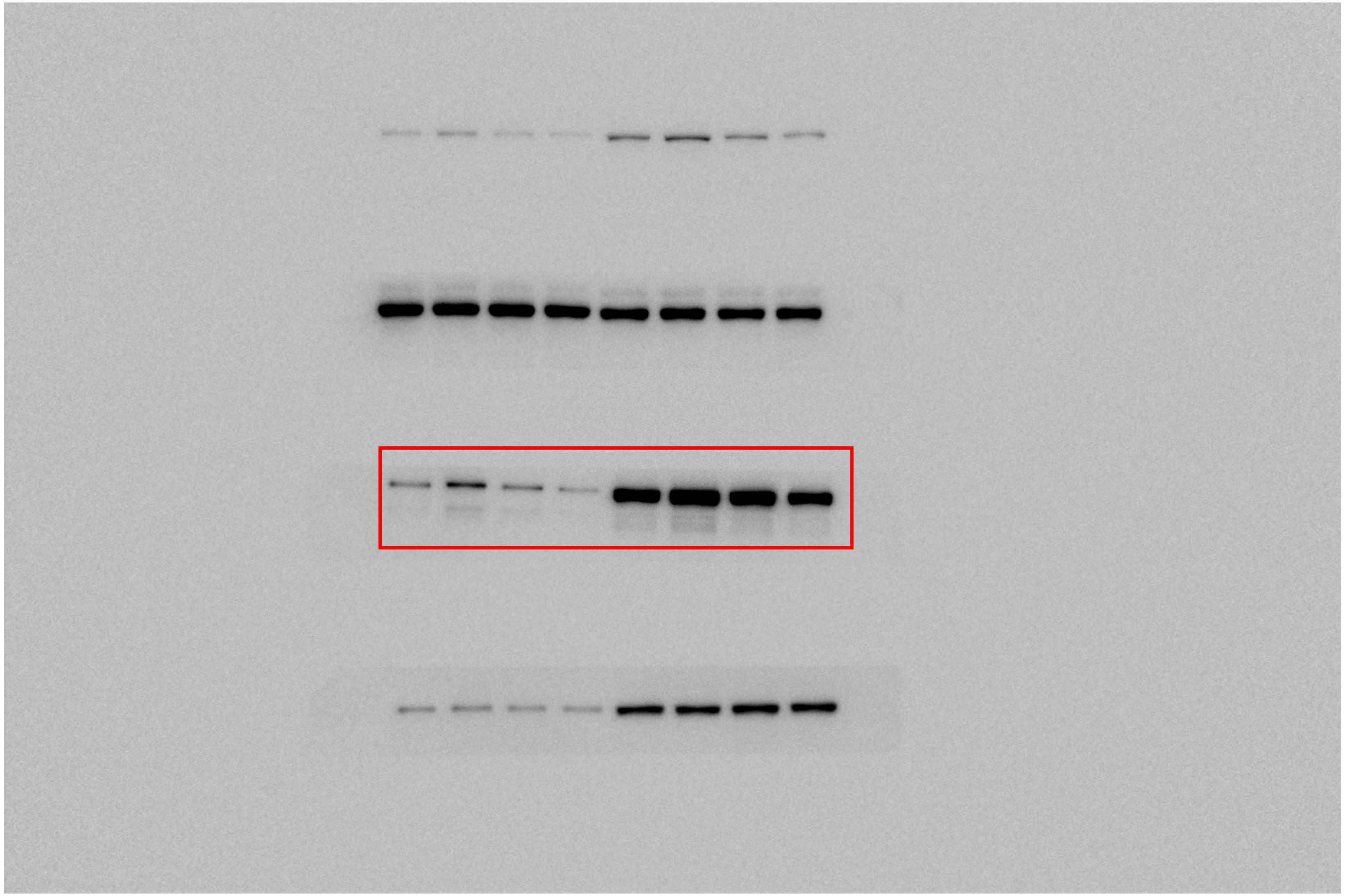

Full and uncropped western blots

Figure 5F

AGS – LaminB1

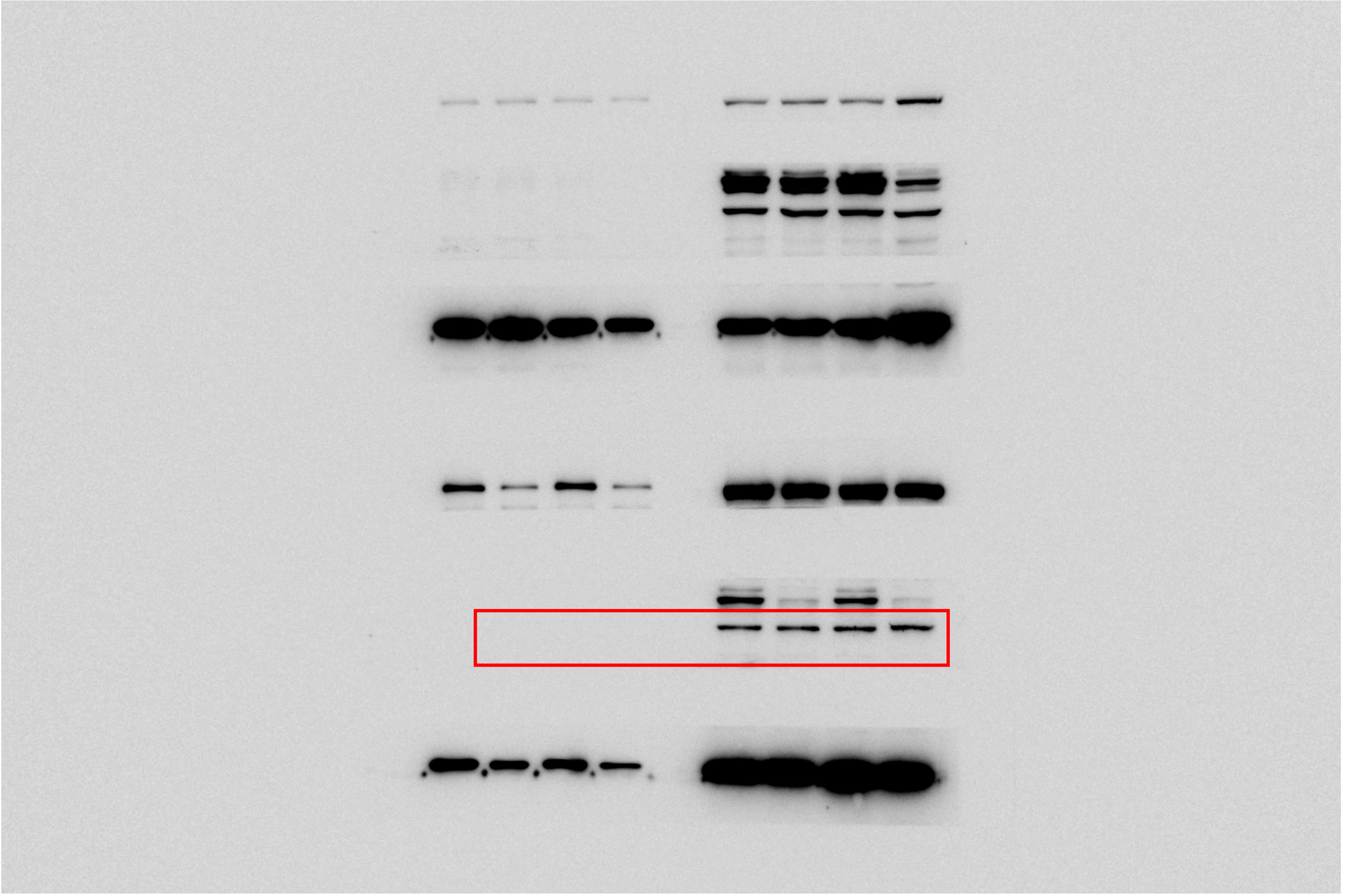

Full and uncropped western blots

Figure 5F

AGS – B-TUBULIN

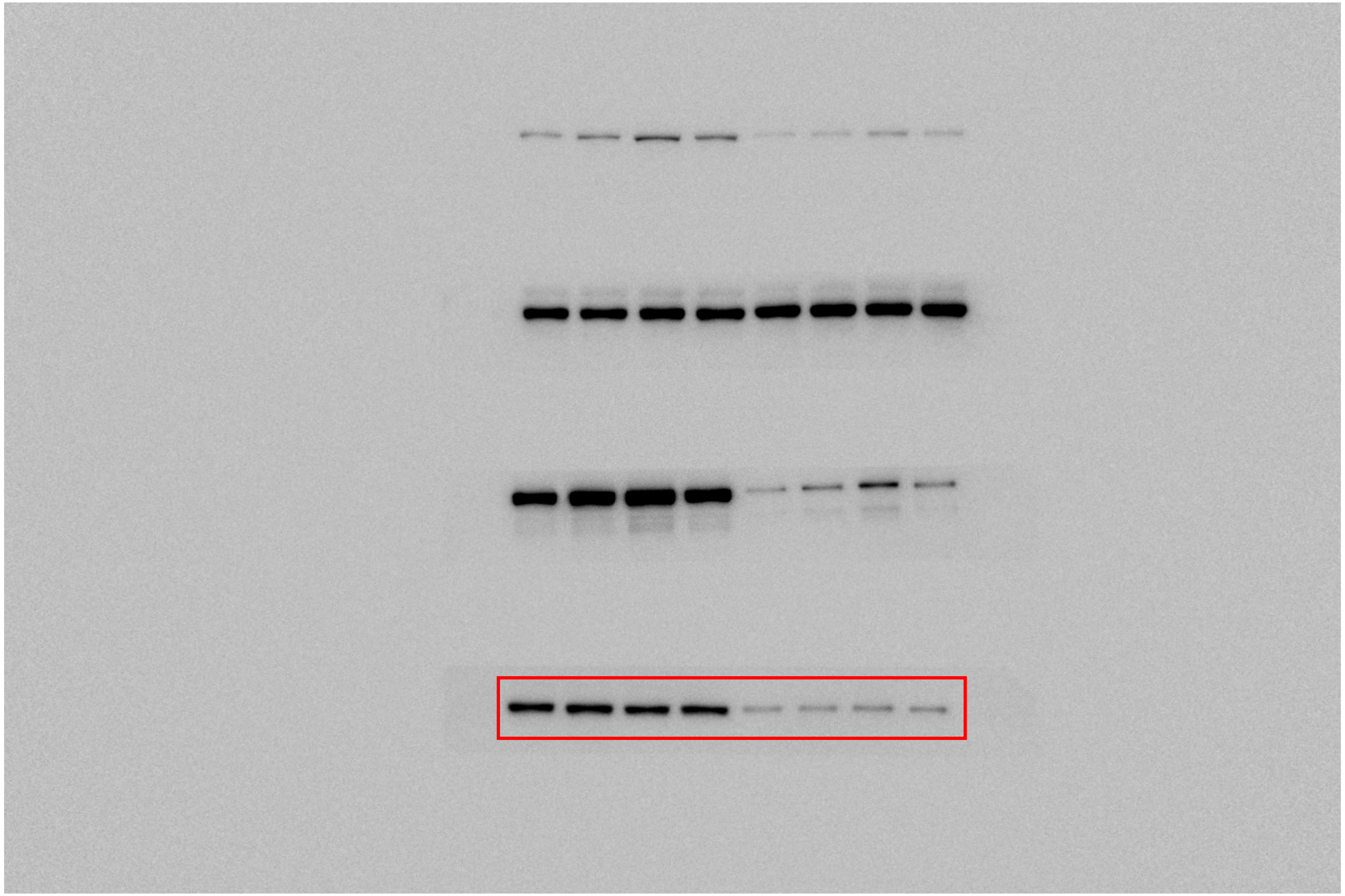

Full and uncropped western blots

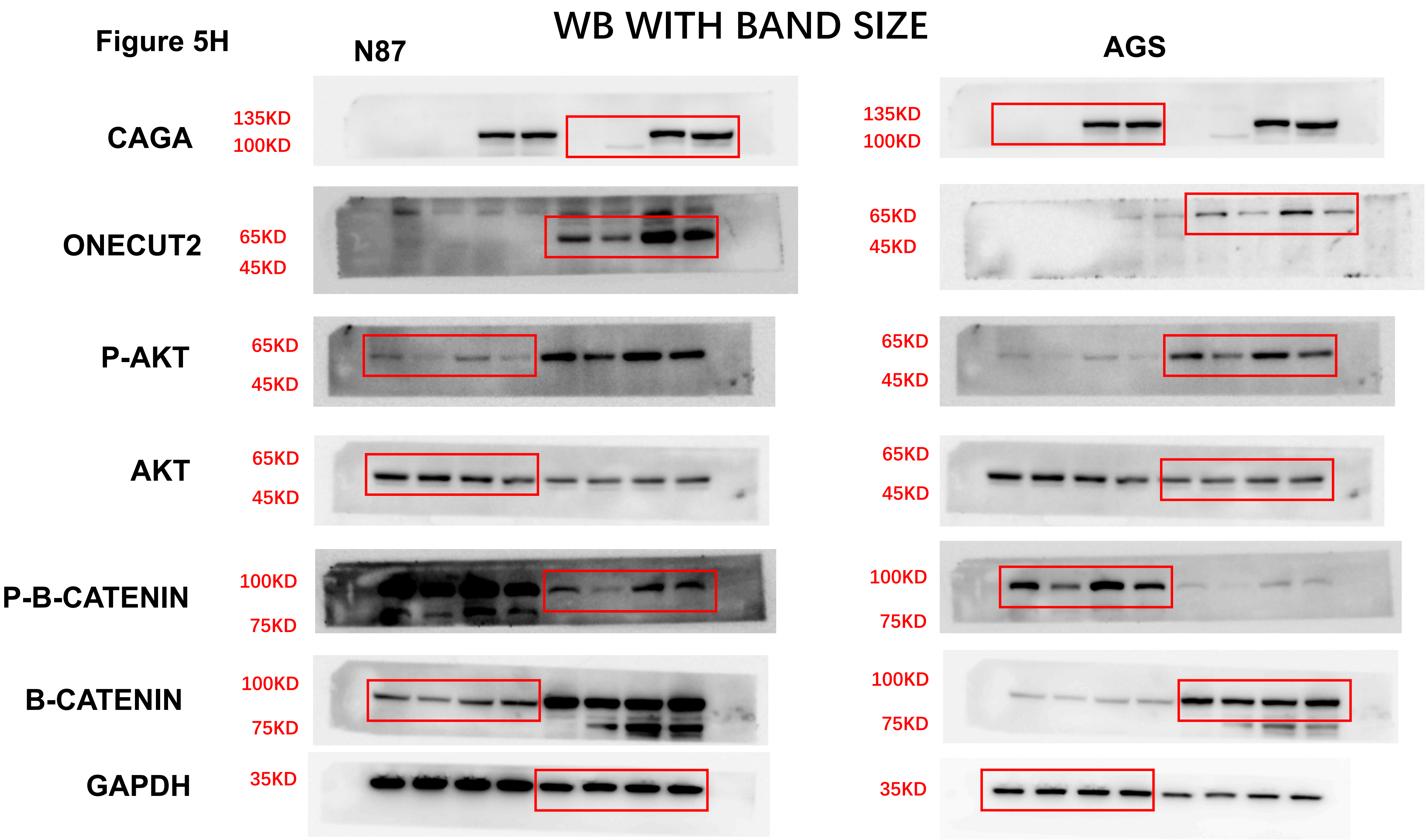

Figure 5H
WB WITH BAND SIZE
N87
AGS
CAGA
135KD
100KD
ONECUT2
65KD
45KD
P-AKT
65KD
45KD
AKT
65KD
45KD
P-B-CATENIN
100KD
75KD
B-CATENIN
100KD
75KD
GAPDH
35KD

Figure 5H

N87 – CAGA

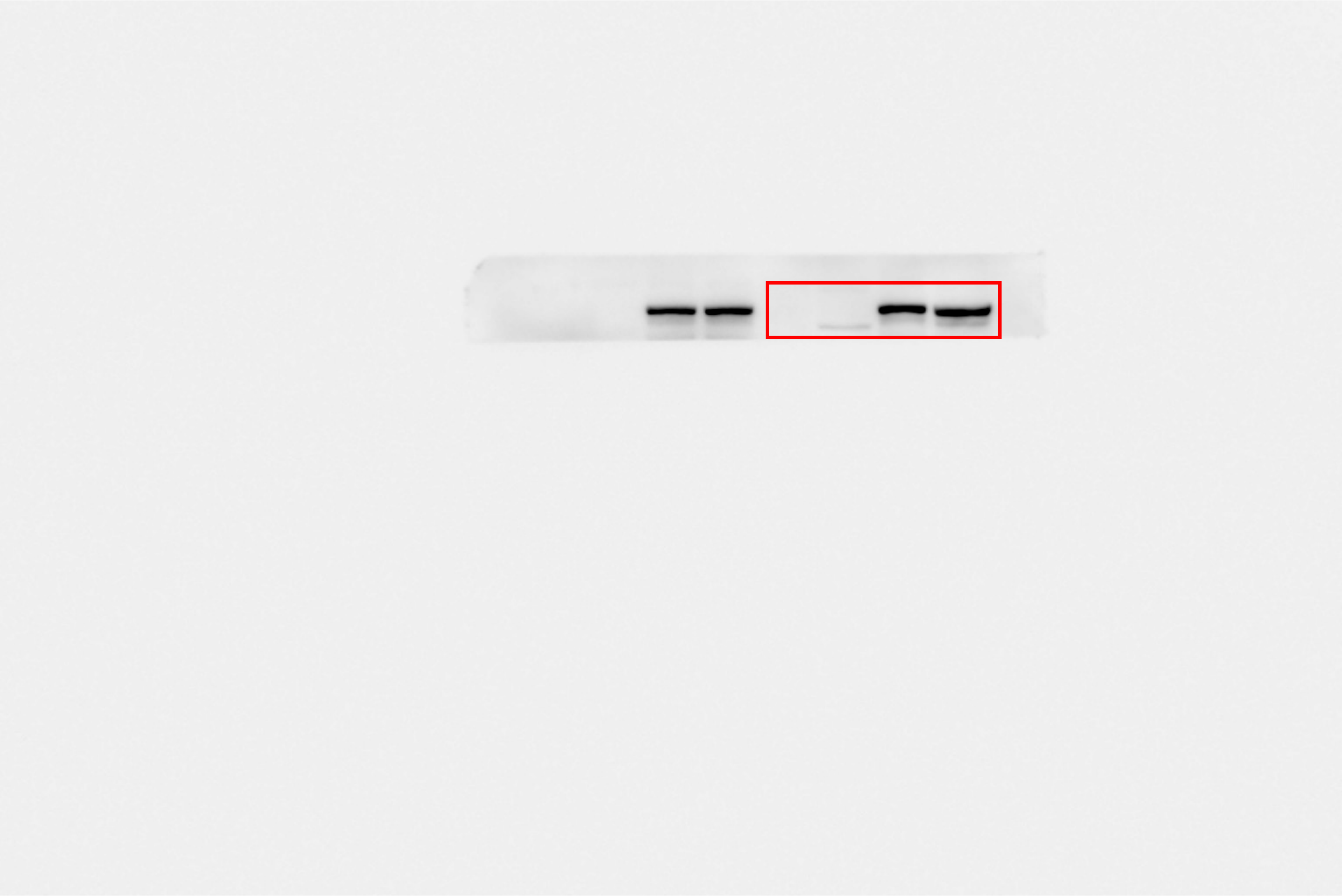

Full and uncropped western blots

Figure 5H

N87 – ONECUT2

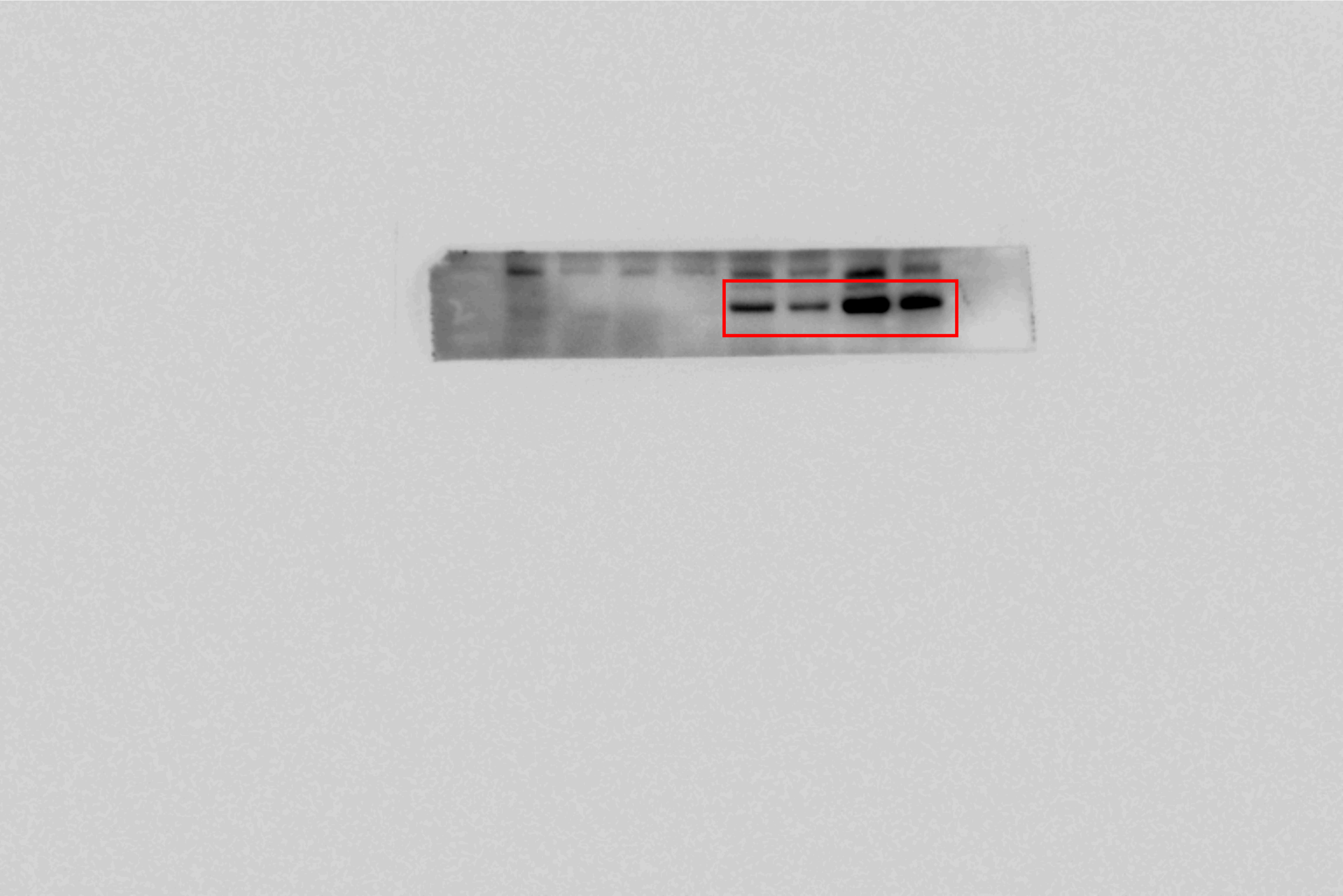

Full and uncropped western blots

Figure 5H

N87 – P- AKT

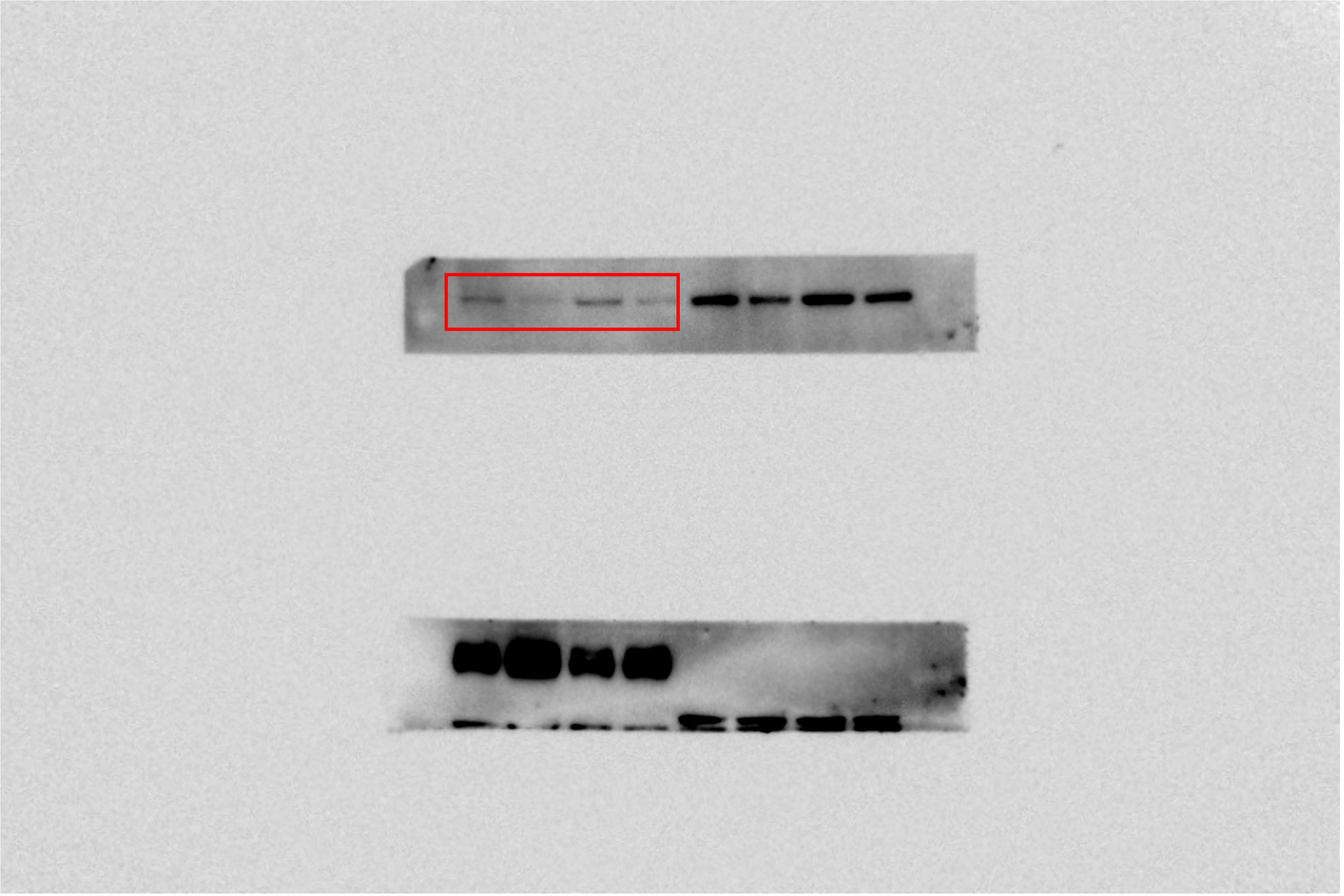

Full and uncropped western blots

Figure 5H

N87 – AKT

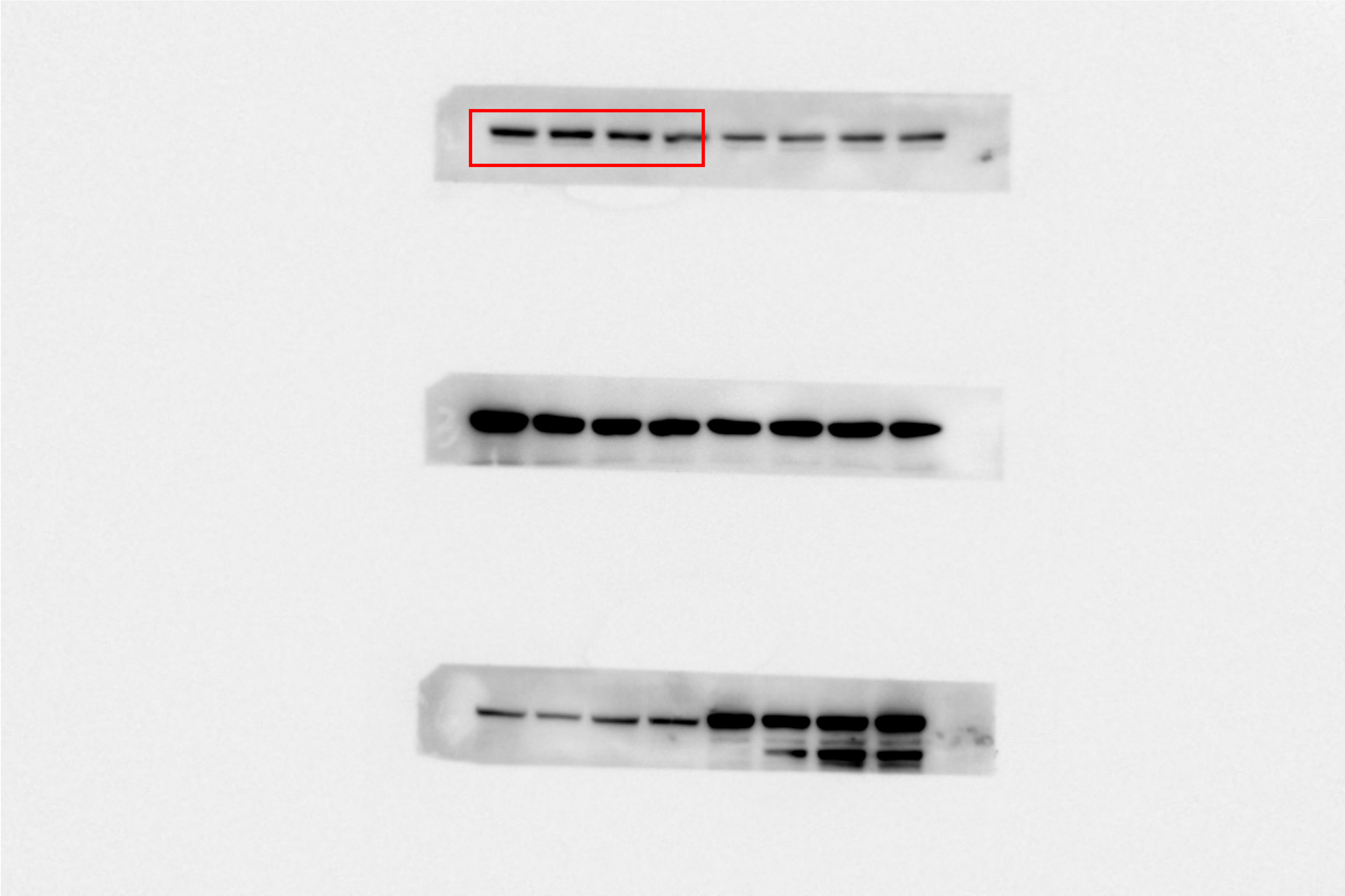

Full and uncropped western blots

Figure 5H

N87 – P- B-CATENIN

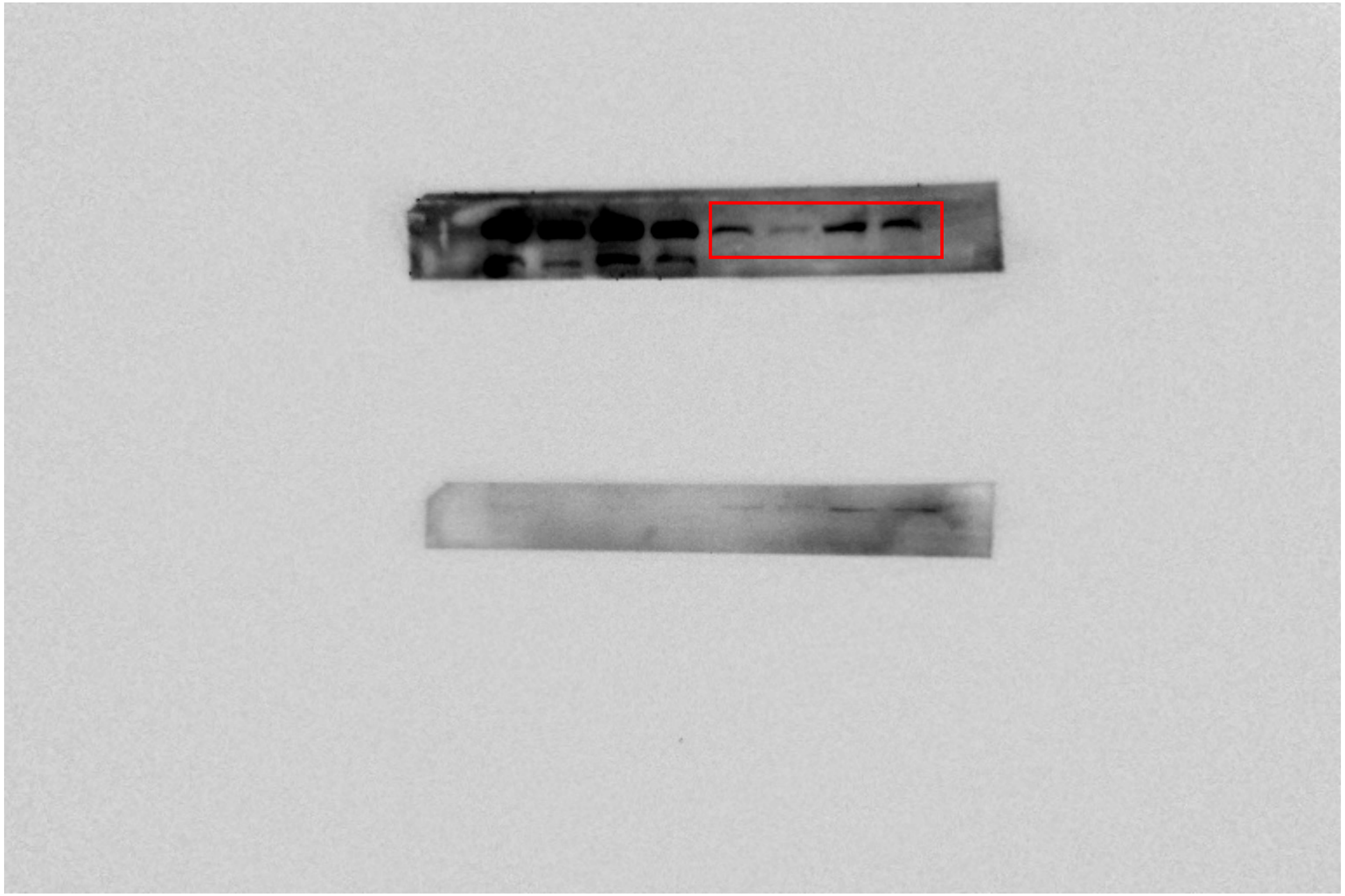

Full and uncropped western blots

Figure 5H

N87 – B-CATENIN

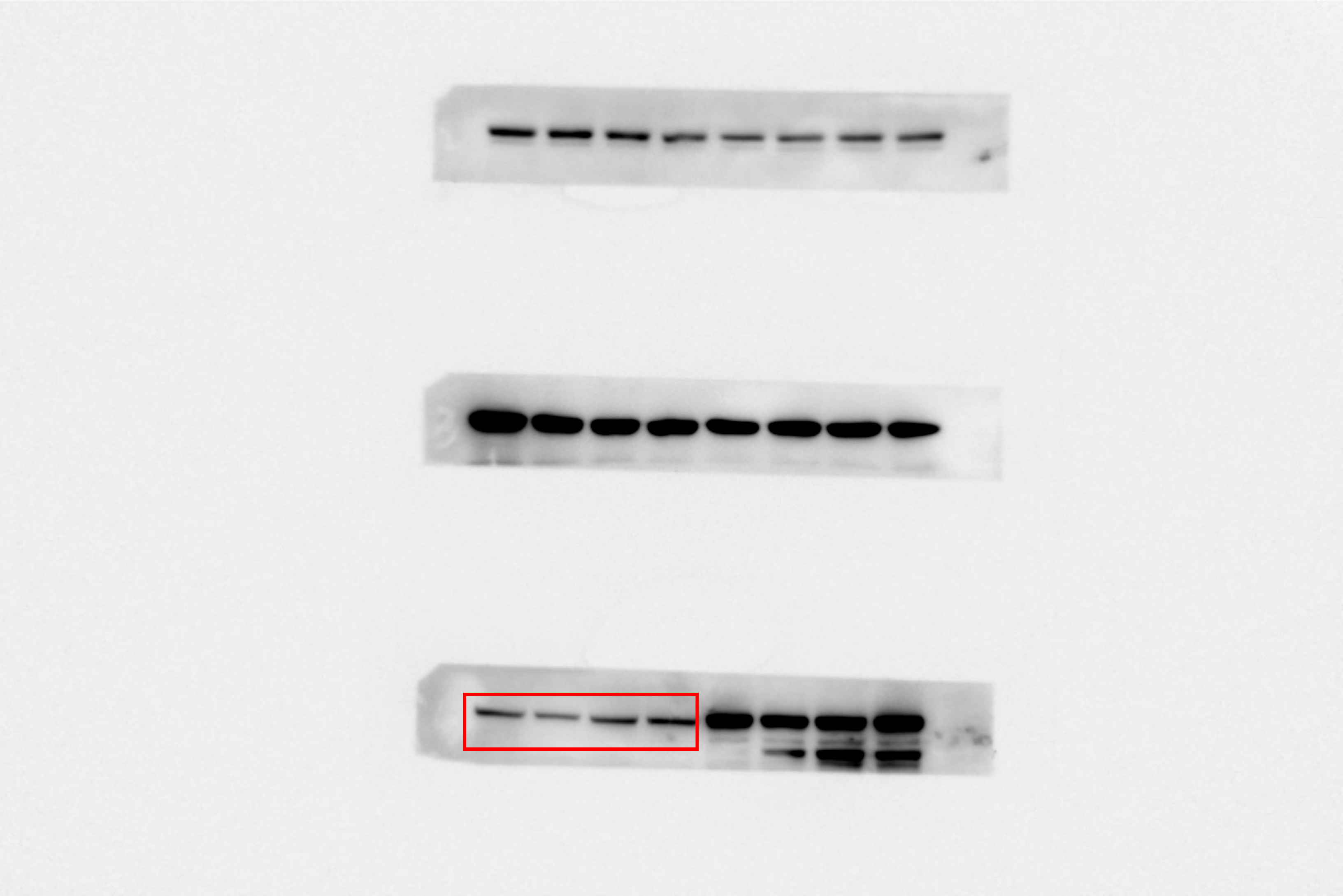

Full and uncropped western blots

Figure 5H

N87 – GAPDH

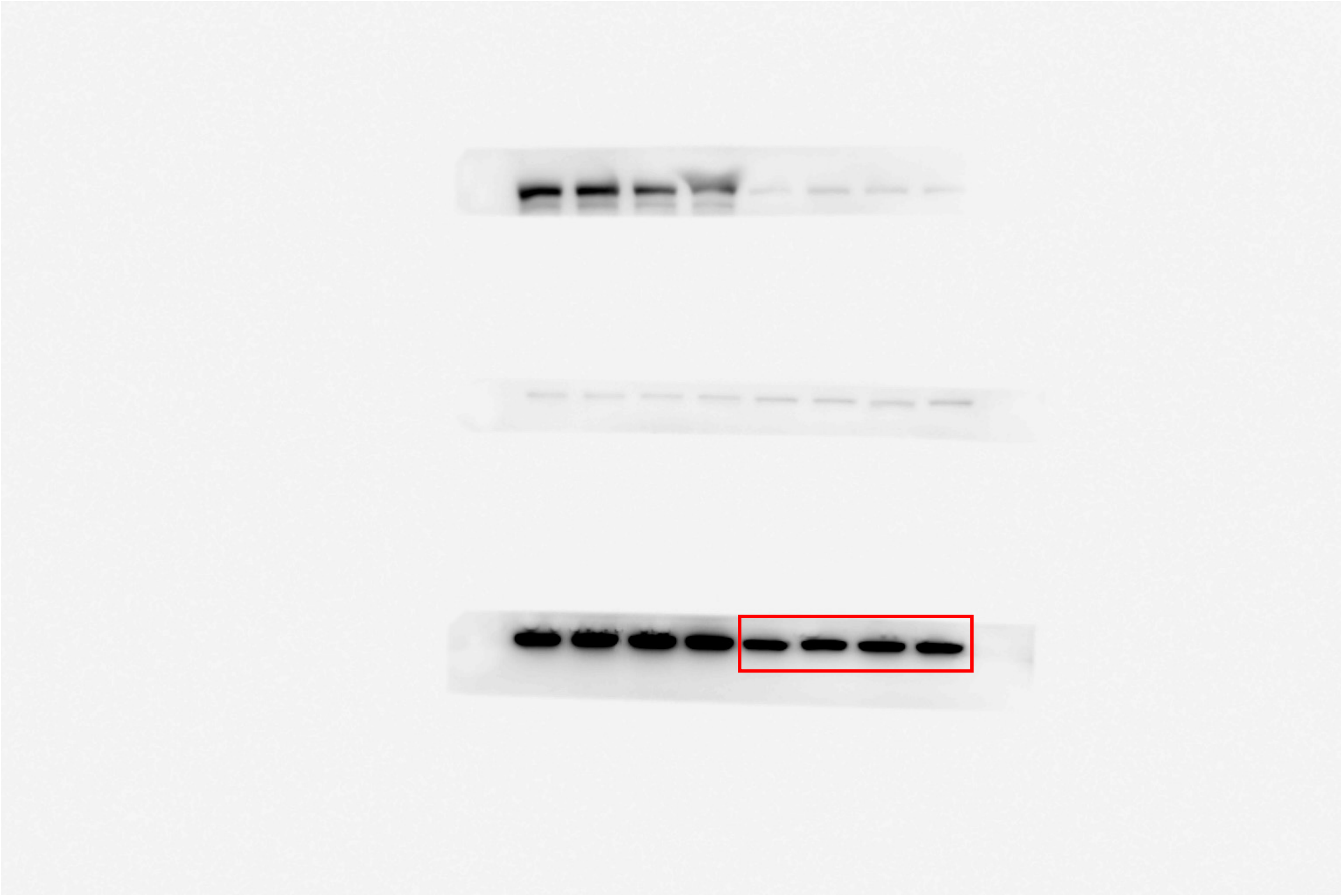

Full and uncropped western blots

Figure 5H

AGS – CAGA

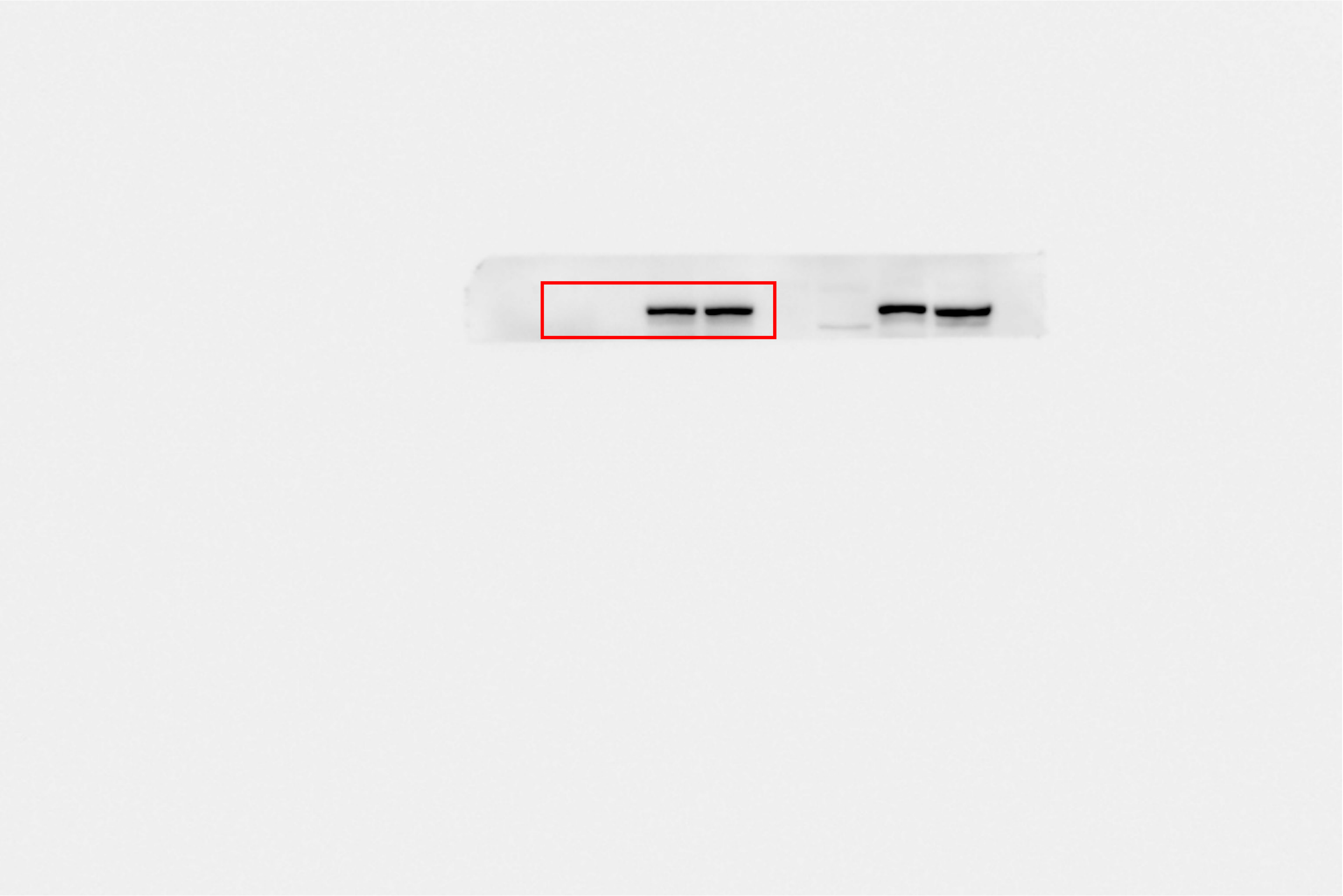

Full and uncropped western blots

Figure 5H

AGS – ONECUT2

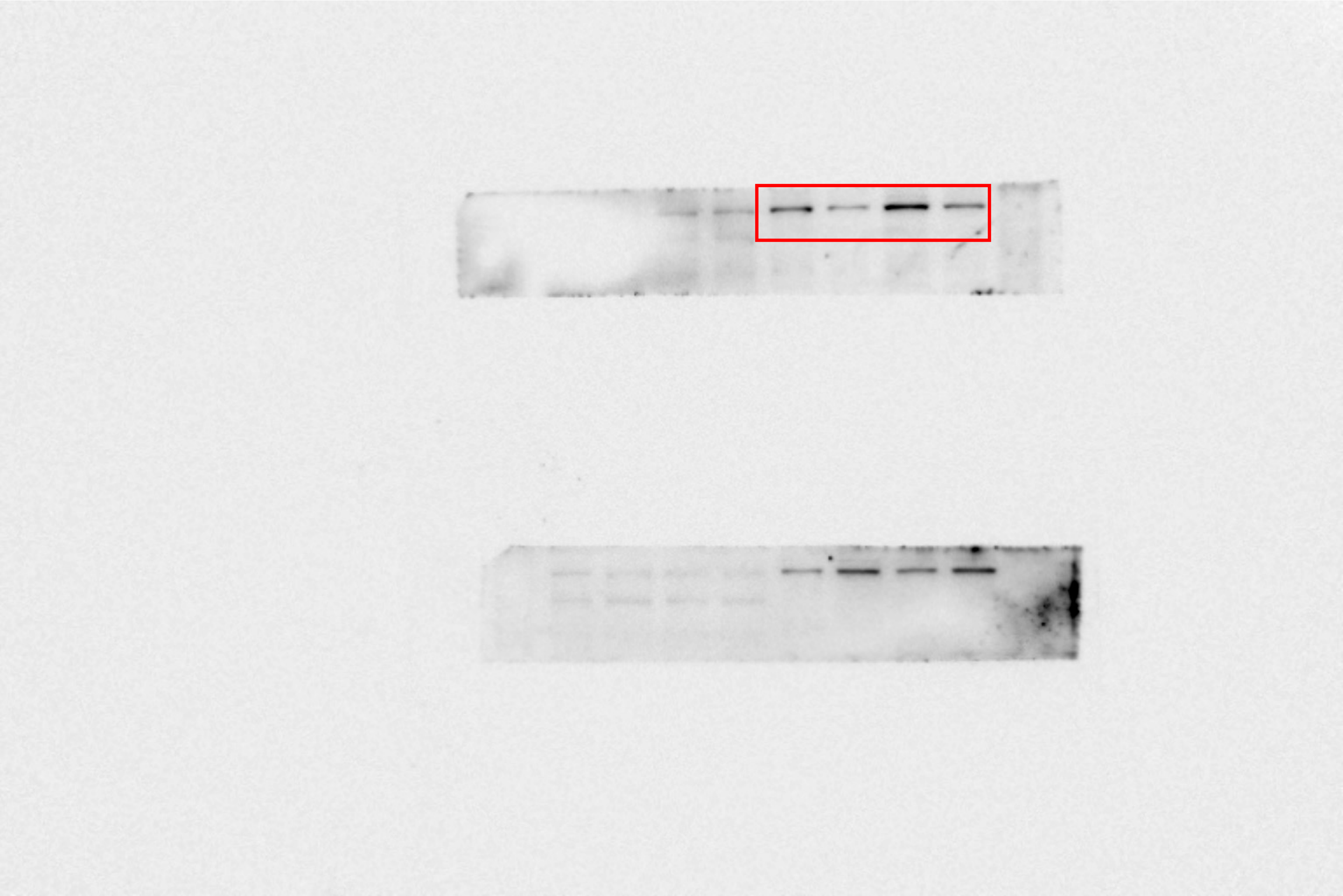

Full and uncropped western blots

Figure 5H

AGS – P- AKT

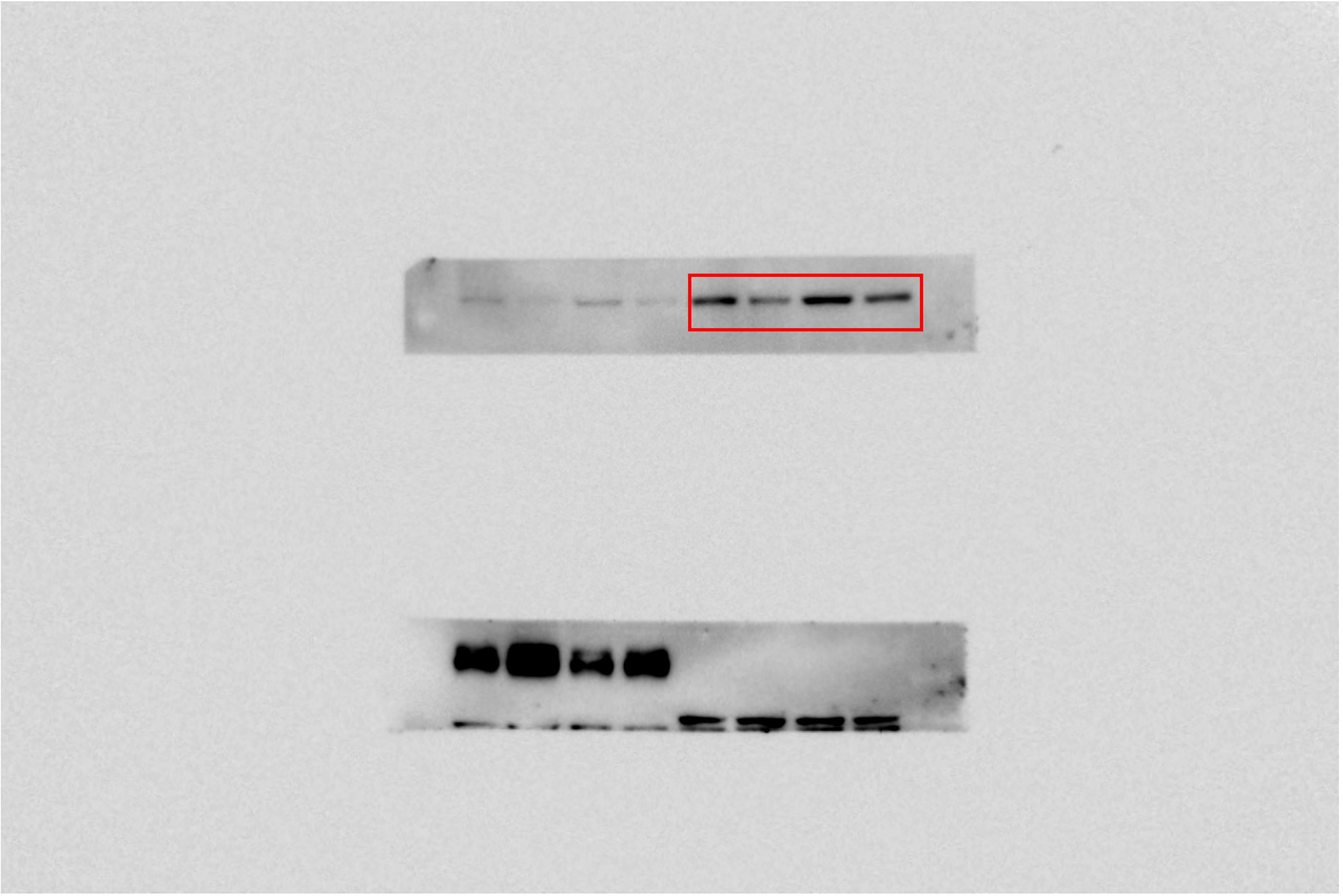

Full and uncropped western blots

Figure 5H

AGS – AKT

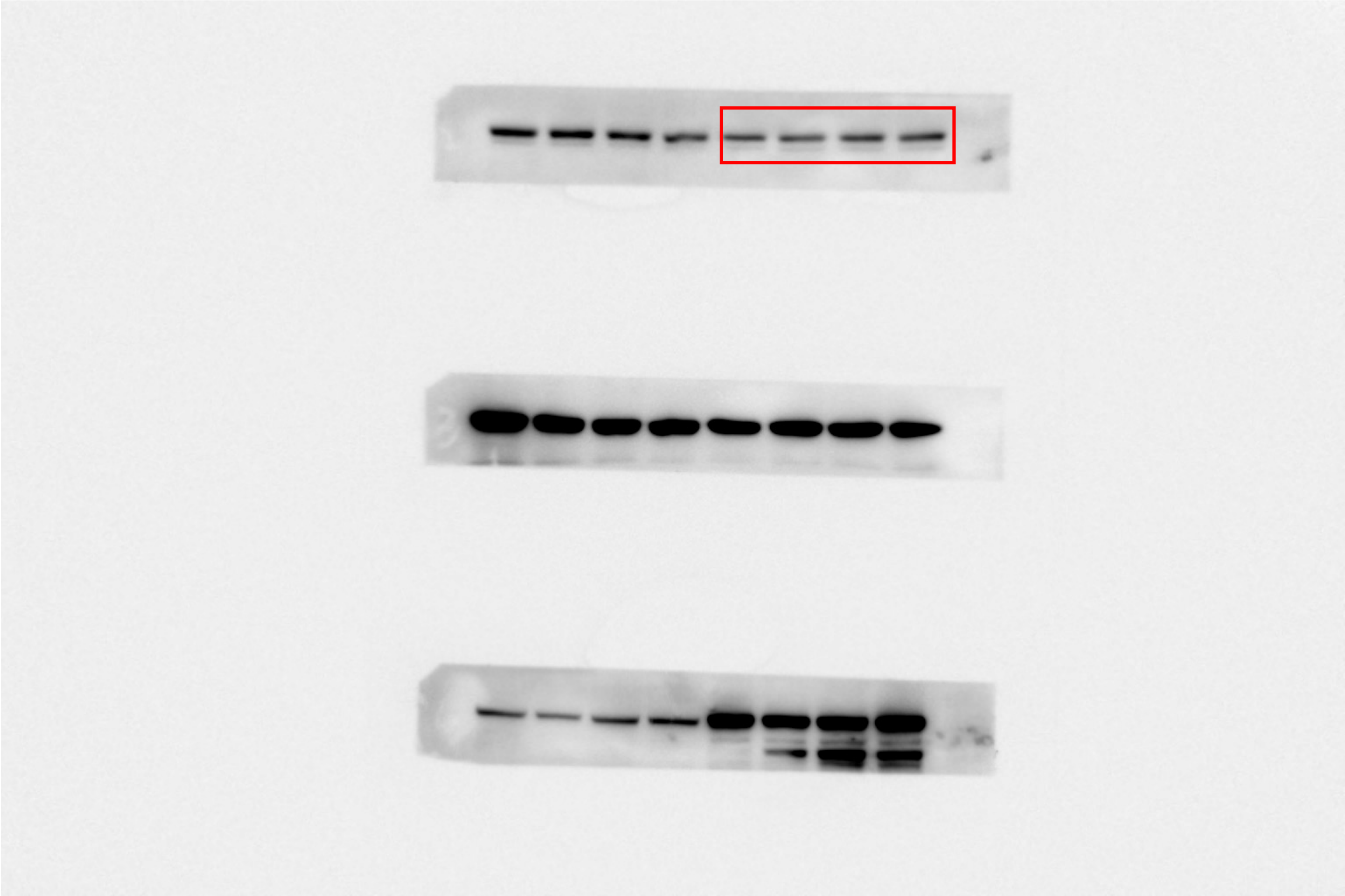

Full and uncropped western blots

Figure 5H

AGS – P-B-CATENIN

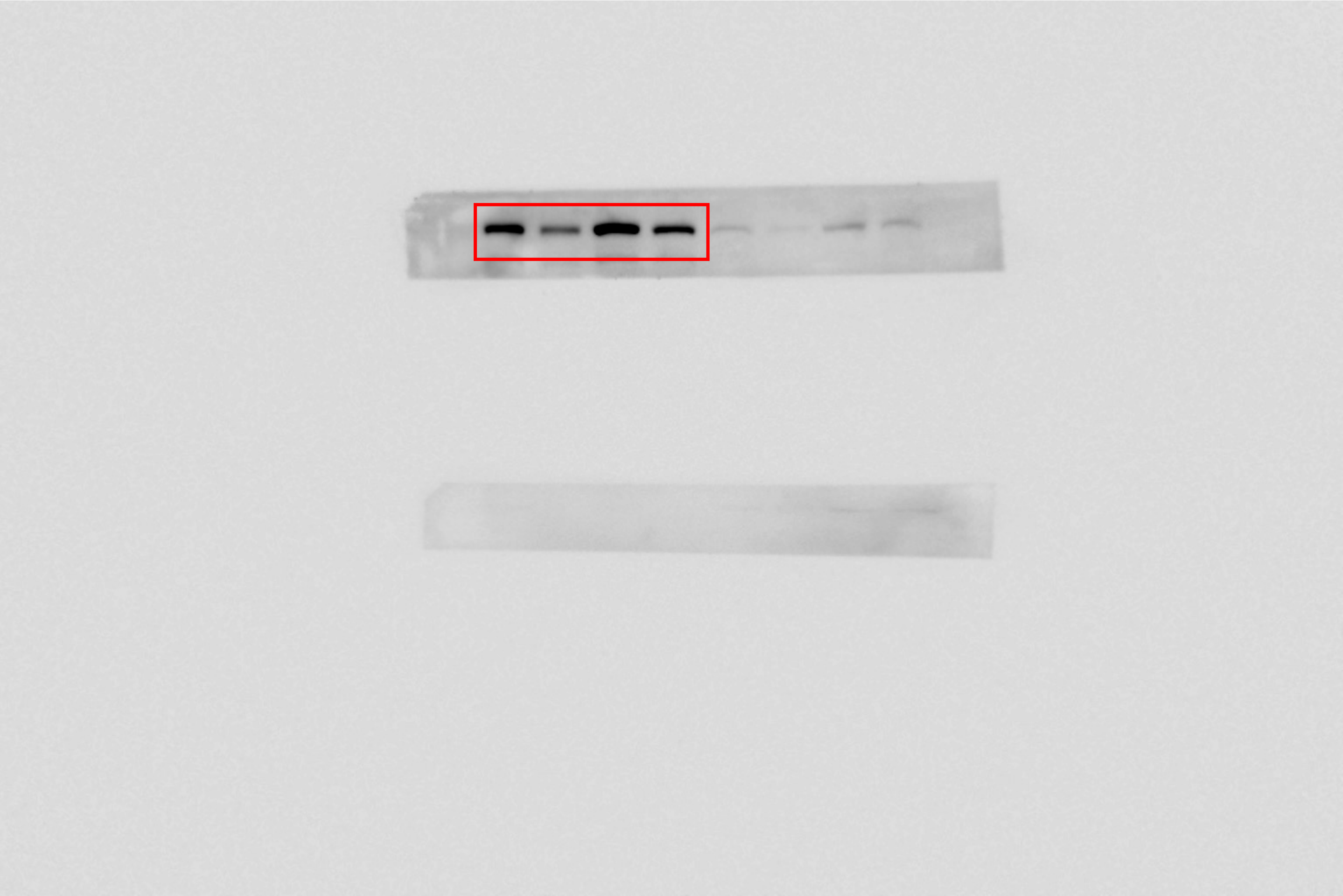

Full and uncropped western blots

Figure 5H

AGS – B-CATENIIN

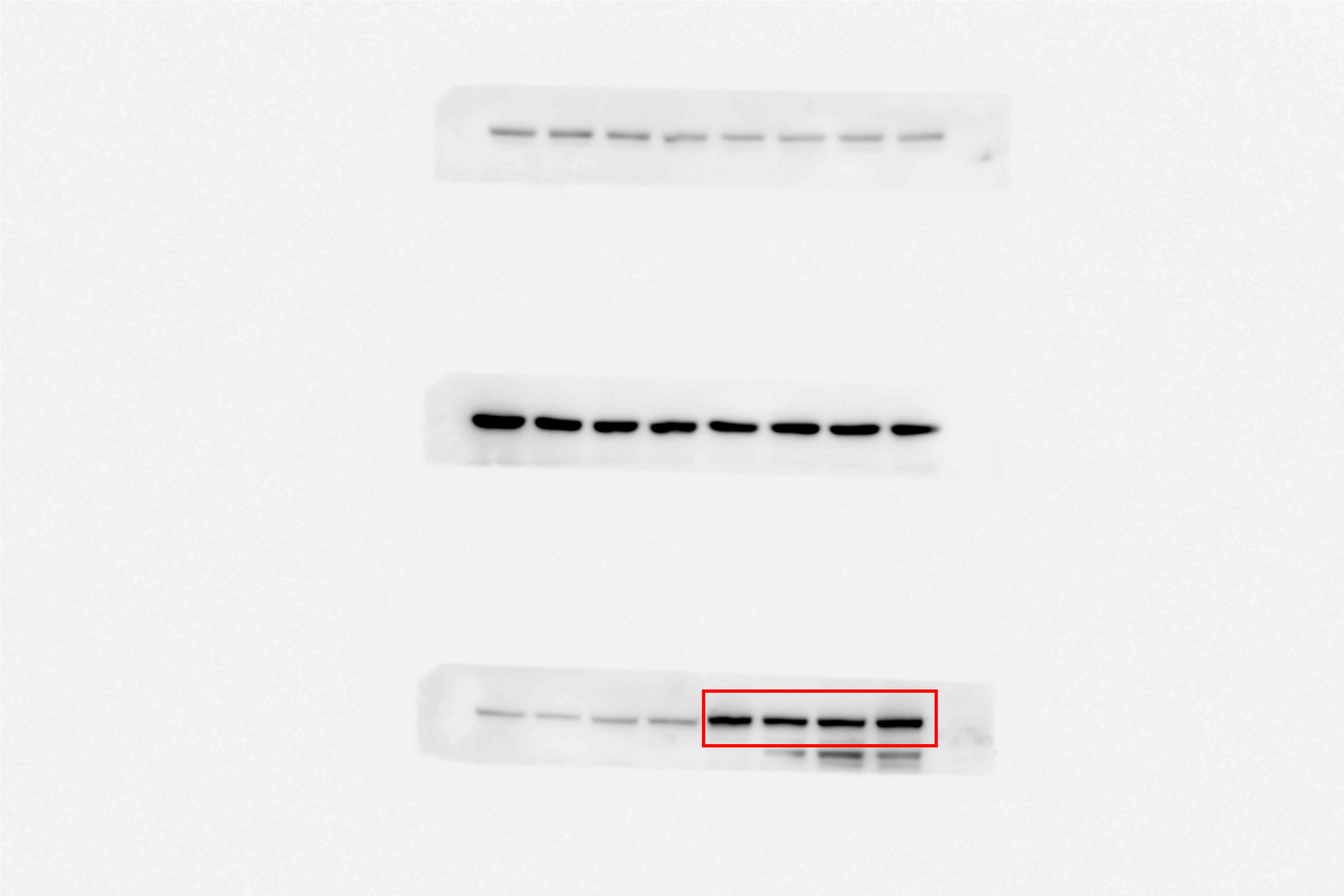

Full and uncropped western blots

Figure 5H

AGS – GAPDH

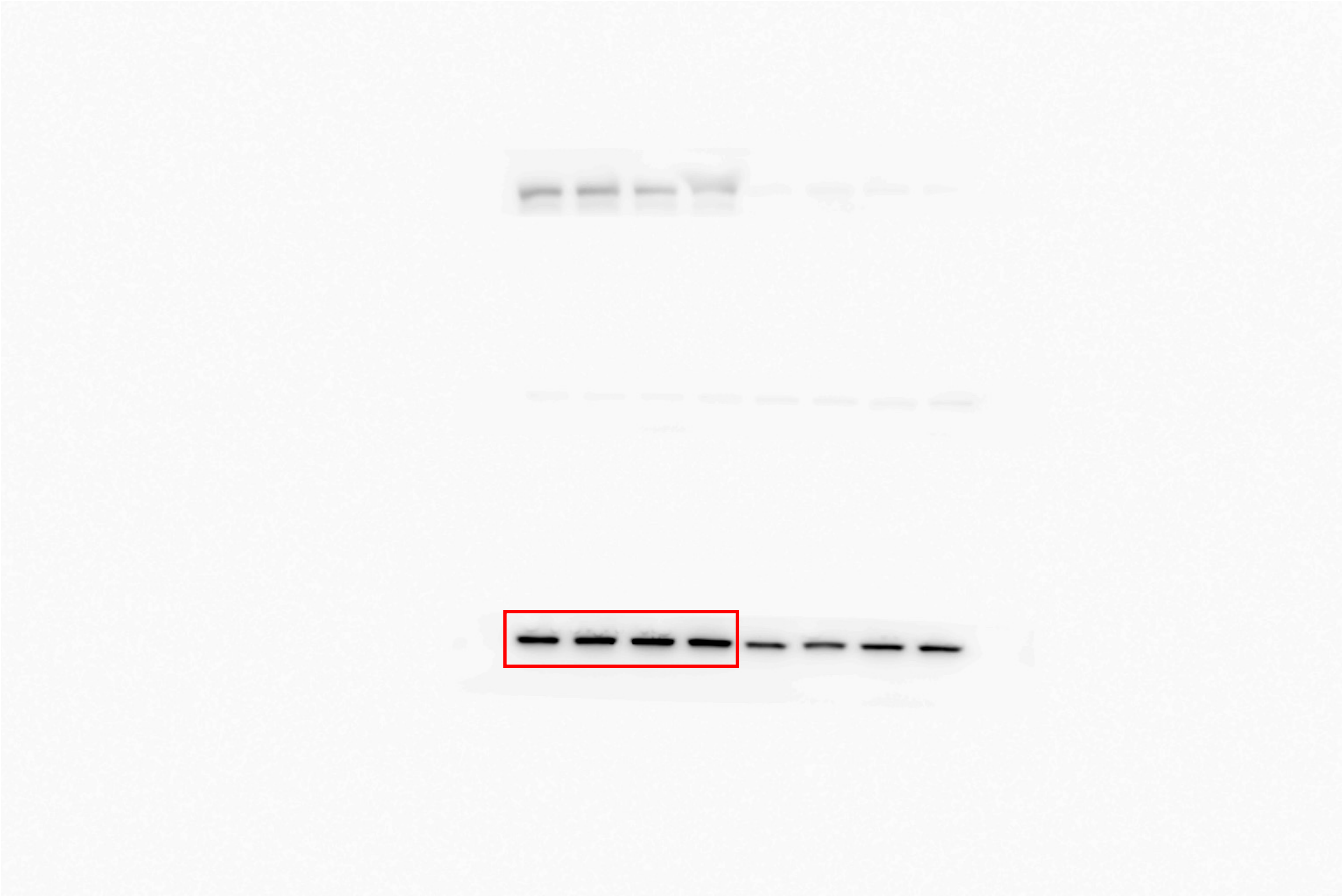

Full and uncropped western blots

sFigure 2A

WB WITH BAND SIZE

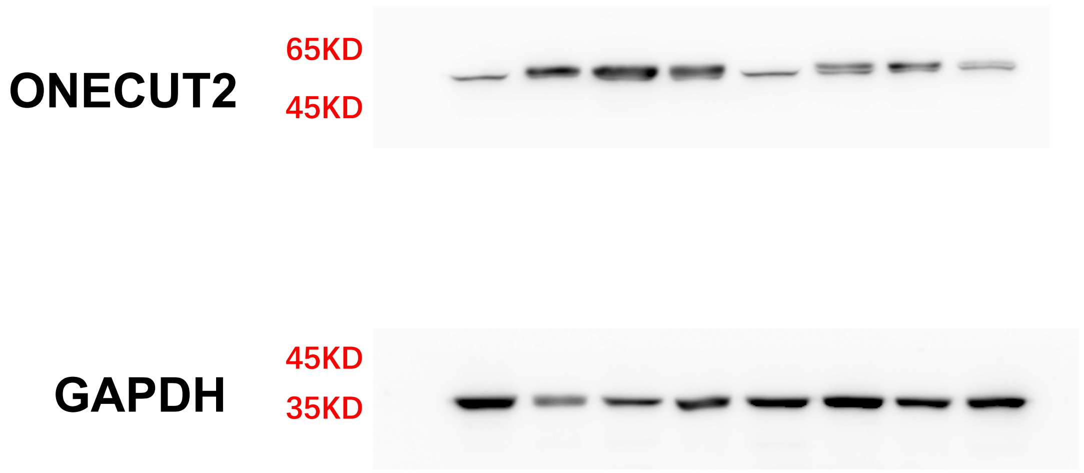

sFigure 2A

ONECUT2

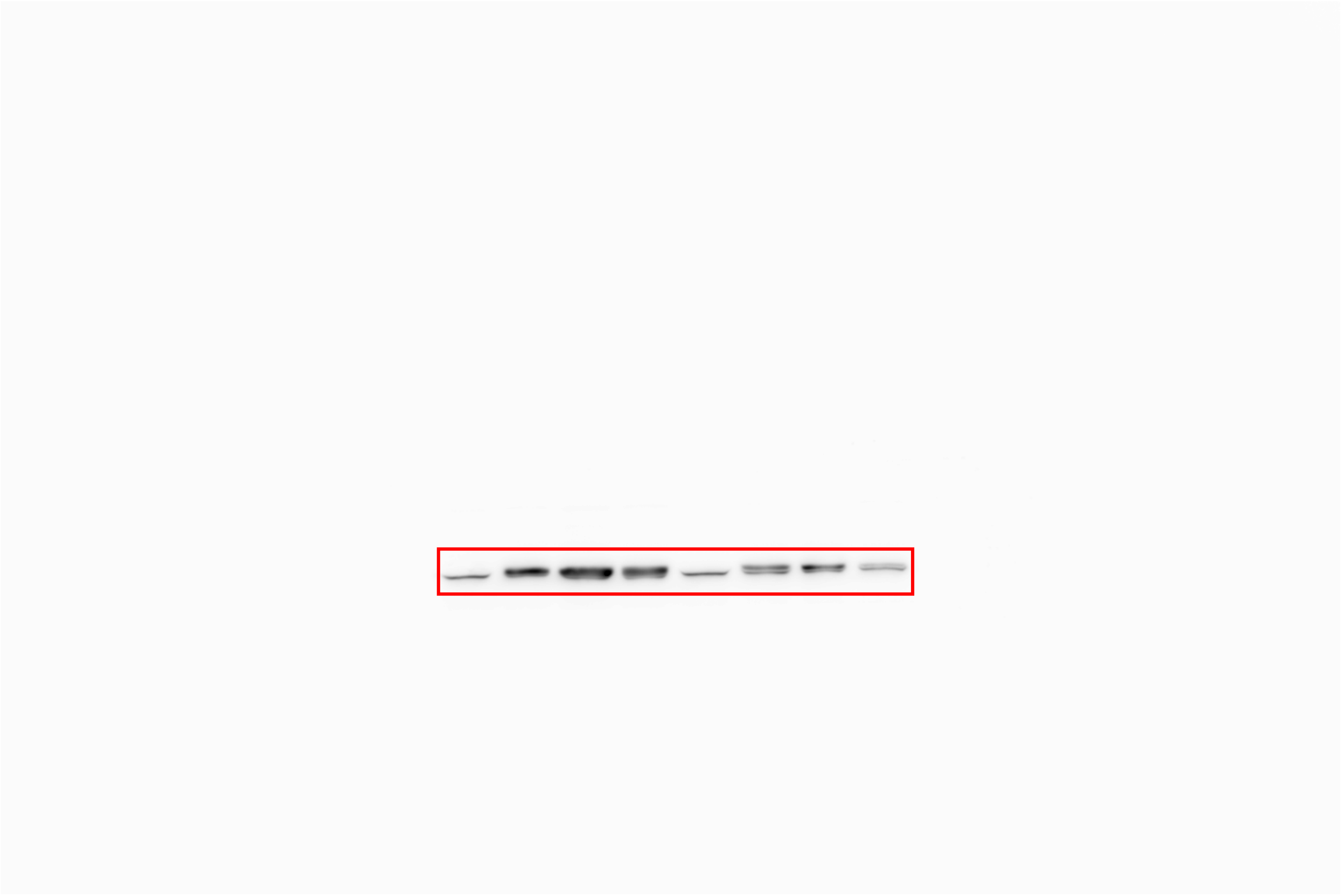

Full and uncropped western blots

sFigure 2A

GAPDH

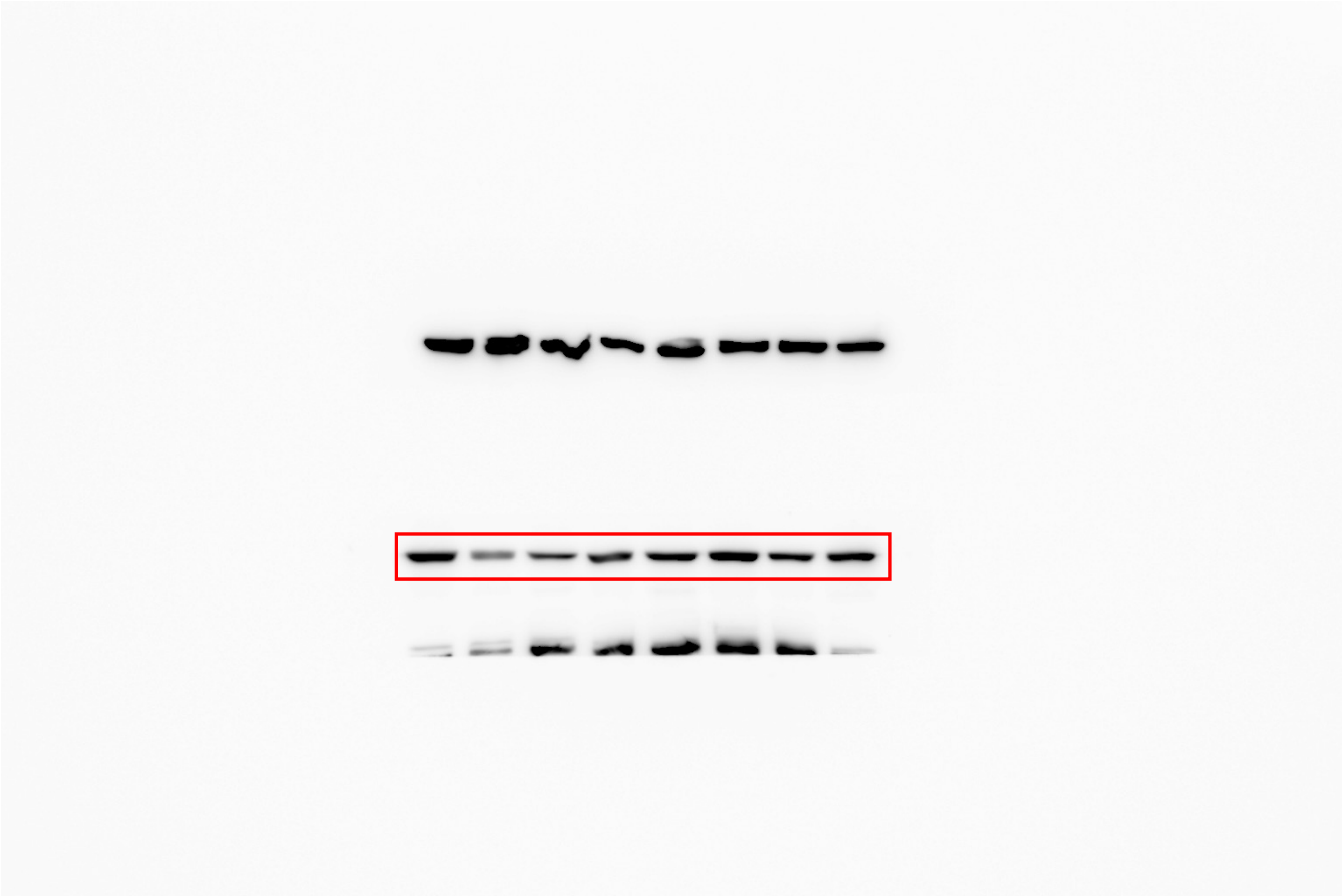

Full and uncropped western blots

Supplementary data 1

#ID	Symbol	shNC04_Cc	shNC05_Cc	shNC06_Cc	sh01_Coun	sh02_Coun	sh03_Coun	shNC04_FP
ENSG0000(	BIVM-ERCC	0	1	2	530	908	727	0
ENSG0000(	SERPINB5	1856	2624	2184	570	544	486	21.06372
ENSG0000(	IFI44	625	394	425	52	54	27	11.72855
ENSG0000(	CYP26B1	1885	2401	2187	1135	945	974	14.58241
ENSG0000(	CYP1B1	1066	1424	1103	573	499	511	6.418947
ENSG0000(	MYLK	440	545	414	1844	1363	2146	2.298915
ENSG0000(	NT5E	1013	1393	1055	461	482	407	10.24845
Homo_sapi	Homo_sapi	115	134	107	404	474	459	1.140823
ENSG0000(	SNPH	337	413	371	169	99	132	2.111733
ENSG0000(	PEG10	370	567	573	1339	1590	1961	1.821166
ENSG0000(	DHFR	1481	1978	1846	3765	3837	4545	12.48221
ENSG0000(	OAS2	206	79	84	2	5	2	1.463517
ENSG0000(	MYLK3	30	45	31	171	167	310	0.132554
Homo_sapi	Homo_sapi	20	24	64	384	244	326	0.261298
ENSG0000(	PIEZO2	163	253	192	563	692	993	0.52212
ENSG0000(	RASA4	270	274	359	94	98	136	2.414712
ENSG0000(	TINAGL1	967	1491	1560	557	677	620	13.90642
Homo_sapi	Homo_sapi	11	17	12	154	159	774	0.162145
ENSG0000(	SERPINB3	80	123	91	27	19	15	1.407027
ENSG0000(	BIRC7	588	694	638	1807	1954	1505	13.88693
ENSG0000(	TP53I11	689	851	793	512	371	429	8.037617
Homo_sapi	Homo_sapi	771	875	1028	511	328	339	28.40668
ENSG0000(	UGT1A7	160	423	290	46	23	6	2.155186
ENSG0000(	SVEP1	99	46	69	477	352	378	0.251014
ENSG0000(	PAPPA	248	298	206	114	97	115	0.711035
ENSG0000(	NCF2	420	219	212	77	28	57	5.942845
ENSG0000(	SEL1L3	44	68	60	12	9	5	0.302547
ENSG0000(	KCND1	220	317	276	161	104	118	1.588326
ENSG0000(	TBX1	778	976	964	1764	1611	1984	12.56824
ENSG0000(	KIAA0319	141	188	134	60	63	73	0.639495
ENSG0000(	AFAP1	1832	2039	1677	3806	4798	4532	7.666874
ENSG0000(	FAM167A	439	658	550	334	189	172	3.398797
ENSG0000(	GCNT3	1269	1675	1423	798	261	309	9.309979
ENSG0000(	KIAA0040	1819	1940	1790	1039	1175	1290	12.54262
ENSG0000(	GPR37	105	108	112	288	286	517	1.087474
Homo_sapi	Homo_sapi	224	220	193	107	62	46	5.329389
ENSG0000(	GNG4	147	175	166	356	367	408	1.114482
ENSG0000(	WISP2	7985	6820	6722	18800	14508	17785	166.8194
ENSG0000(	GREM2	37	42	43	88	603	510	0.275925
ENSG0000(	SERPINB4	39	70	99	11	14	5	0.699364
ENSG0000(	PRR15	161	109	88	16	14	41	3.609906
ENSG0000(	HHIPL2	757	760	632	403	345	494	9.24562
ENSG0000(	WRB	420	575	485	1062	835	1207	8.554723
ENSG0000(	RAB27B	925	1339	1222	722	790	825	3.998925
ENSG0000(	FABP3	462	610	630	1172	1167	1129	20.03514
ENSG0000(	IFIT1	1543	958	1017	388	587	402	25.0547
ENSG0000(	NECTIN1	203	321	300	157	64	87	1.123287
ENSG0000(	BACE2	3071	4024	4041	6790	11339	10674	25.01466
ENSG0000(	SLC7A11	1431	3262	2795	670	821	1476	4.677762

ENSG0000(	CNTD2	313	179	162	36	80	43	7.902846
ENSG0000(	MARCKSL1	981	1568	1294	2397	2514	2589	18.16643
ENSG0000(	PDZD2	1056	1540	1293	1000	880	884	2.838022
ENSG0000(	HIGD2A	1511	1953	1909	3144	3276	3507	71.1317
ENSG0000(	ACOXL	149	205	151	80	81	95	1.780615
ENSG0000(	SH2B3	412	678	549	291	338	352	2.397763
ENSG0000(	SECTM1	412	475	329	248	202	167	5.884927
ENSG0000(	WNT10B	1159	1178	1101	2198	3103	2909	16.08111
ENSG0000(	TMEM154	127	171	159	88	70	68	0.434277
ENSG0000(	USH1C	1091	1240	1586	2363	4674	6797	16.3233
ENSG0000(	GCLC	1722	2136	2194	1358	1478	1357	14.41866
ENSG0000(	HSPA5	134859	65804	67695	40071	35592	34483	1085.84
ENSG0000(	ID1	7195	9351	7483	6411	4973	4626	223.2156
ENSG0000(	GBP3	258	441	309	165	154	227	2.834992
ENSG0000(	PDIA4	24098	16417	17034	12304	8627	8040	261.3206
Homo_sapi	Homo_sapi	43717	58133	49013	88146	112756	101951	591.5791
ENSG0000(	SUCLG2	2903	3644	3215	5535	5074	6029	39.19871
ENSG0000(	RFTN1	217	246	186	539	400	550	2.30325
ENSG0000(	KLHDC7B	39	45	50	13	10	10	0.315925
ENSG0000(	ABHD14B	1524	2118	1965	3420	2974	3327	27.74334
ENSG0000(	ARNTL2	1500	1889	1608	1210	1168	1418	8.387278
ENSG0000(	PPAN-P2RY	438	1036	855	398	222	439	5.768629
ENSG0000(	TMEM170E	10	24	10	65	81	79	0.036175
ENSG0000(	IGSF3	2645	4088	3544	2590	2503	2494	11.60647
ENSG0000(	PCOLCE	5913	6291	5986	13333	10162	11320	109.3146
ENSG0000(	CLYBL	115	141	127	287	282	505	2.869494
Homo_sapi	Homo_sapi	182	242	219	82	97	144	4.014145
ENSG0000(	SLITRK5	511	846	661	1294	1156	1512	0.760643
ENSG0000(	PCDH12	86	128	130	62	36	51	0.407207
ENSG0000(	MAP4K4	2005	1861	1736	1384	1124	1263	9.468206
ENSG0000(	PCDH1	2471	2431	1929	1587	1431	1479	20.42381
ENSG0000(	IFIT2	1555	710	781	176	442	136	14.71451
ENSG0000(	AQP1	496	931	709	439	134	158	6.863142
ENSG0000(	STK17A	2763	3628	3427	2583	1885	2760	26.03541
ENSG0000(	DDX58	333	178	196	88	95	97	2.4015
ENSG0000(	VDR	708	1217	1135	753	522	679	6.032889
ENSG0000(	C1QL1	1756	2093	2156	3550	6358	4936	42.6511
ENSG0000(	GAST	113	87	96	50	23	25	7.529963
ENSG0000(	GCLM	1046	1692	1610	1069	647	1002	6.847328
ENSG0000(	MICB	1603	1992	1807	1513	1118	1059	20.83486
ENSG0000(	RTKN2	11	13	19	61	57	79	0.157835
ENSG0000(	CITED4	234	440	343	612	1116	1019	5.580827
ENSG0000(	TPD52L1	1279	1420	1426	1105	710	1024	31.01703
ENSG0000(	PHPT1	3970	5042	4723	8324	7482	7543	137.9259
ENSG0000(	GLS	2307	2870	2778	2015	2091	2026	16.11827
ENSG0000(	HSPH1	3523	4305	4070	3538	2750	3152	22.54082
ENSG0000(	HSP90B1	43446	58941	68571	27550	39966	35209	483.4135
ENSG0000(	MSMB	86	53	42	0	0	7	5.521278
ENSG0000(	TRANK1	344	414	353	283	211	257	1.041506
ENSG0000(	CDC42	5171	9145	7859	3274	3196	5905	77.96584

ENSG0000(	ARHGEF2	6971	6543	6229	5150	4288	4630	56.50996
ENSG0000(	TNS4	800	1169	1056	478	658	735	6.72309
ENSG0000(	IFI27	82	61	58	28	16	13	4.178466
ENSG0000(	GPAT3	141	206	186	110	43	44	1.676122
ENSG0000(	KCNK15	1142	929	687	3612	1666	2958	13.81516
ENSG0000(	SYNE3	328	417	434	216	273	243	1.674912
ENSG0000(	LPCAT2	376	574	539	371	315	344	2.356181
ENSG0000(	TPM3	22437	26095	23234	21015	13072	16122	298.8902
Homo_sapi	Homo_sapi	200	323	281	591	449	593	12.11946
ENSG0000(	RABL2B	375	436	318	839	672	791	5.294469
ENSG0000(	PCED1B	180	216	190	135	107	106	2.563593
ENSG0000(	CXCR4	873	859	756	1526	4023	2405	16.09319
ENSG0000(	AVPI1	3712	4828	4753	7185	7488	8447	80.14544
Homo_sapi	Homo_sapi	70	89	78	41	26	36	0.805829
ENSG0000(	SELENOI	1205	1298	1300	1006	862	1059	4.6789
ENSG0000(	BICDL1	355	695	670	148	248	359	4.174933
ENSG0000(	SERPINE2	2799	3550	2708	2154	1015	677	40.76504
Homo_sapi	Homo_sapi	283	268	270	180	160	178	7.390671
ENSG0000(	STEAP1	44	69	85	19	22	27	1.031851
ENSG0000(	ABCC2	115	153	148	87	32	48	0.623048
ENSG0000(	IFIT3	865	500	488	190	346	192	10.89862
ENSG0000(	SYT7	181	222	184	82	118	119	1.301213
ENSG0000(	ADSSL1	1123	1208	1189	1911	2813	3030	20.3549
ENSG0000(	RAET1L	75	39	45	8	6	18	2.000931
ENSG0000(	RTEL1-TNFI	287	273	310	183	133	210	1.87199
ENSG0000(	EFNA1	3783	4029	3407	5412	13142	16423	63.19146
ENSG0000(	SPAG4	386	461	516	714	1904	1533	8.805776
ENSG0000(	EXT2	3400	4174	3997	3489	2796	2955	32.99036
ENSG0000(	NPHP3-ACA	199	102	104	332	499	699	0.803288
ENSG0000(	BAG1	5404	6165	5119	9610	8999	10527	100.0881
Homo_sapi	Homo_sapi	129	179	202	320	386	489	11.04603
Homo_sapi	Homo_sapi	1267	2102	2117	1460	870	880	10.40026
ENSG0000(	RASSF8	1291	1694	1551	1015	1180	1200	7.413778
ENSG0000(	SLC16A6	1383	1578	1312	2039	5772	5487	12.45121
ENSG0000(	ITGA6	2143	2666	2423	2063	1900	1862	12.2328
ENSG0000(	TGFA	1047	1134	1013	881	738	812	7.83618
ENSG0000(	ANO2	93	64	85	282	155	454	0.785155
ENSG0000(	GRB10	127	274	206	67	112	96	0.853943
ENSG0000(	FBLN7	474	355	411	788	1028	1486	7.62294
ENSG0000(	TMEM37	343	555	517	841	854	1022	8.607439
ENSG0000(	UPF3B	635	771	686	1178	1126	1318	8.642941
ENSG0000(	FADS1	10178	12911	10639	18493	16715	19924	73.16958
ENSG0000(	CCNYL1	1447	1730	1642	1429	947	1213	12.35971
ENSG0000(	HEG1	1155	2547	2073	1412	958	1074	5.234112
ENSG0000(	TMEM40	168	89	136	70	32	42	2.900556
Homo_sapi	Homo_sapi	34	46	39	10	10	15	0.490867
ENSG0000(	NREP	210	441	428	727	751	1091	3.164797
ENSG0000(	TNFRSF21	1508	2077	2030	1625	1366	1418	13.19243
ENSG0000(	CFH	891	938	833	1351	2530	3185	8.363087
ENSG0000(	EPB41L3	308	430	387	628	697	809	2.363532

ENSG0000(	RARRES3	106	38	72	11	0	11	4.42644
ENSG0000(	KRT9	31	59	40	5	0	10	0.417073
ENSG0000(	FGF2	2526	3137	2531	1463	2084	1954	12.18243
ENSG0000(	SLC25A6	14783	22372	21171	31021	42517	40997	225.5806
ENSG0000(	PRPH	128	177	304	73	102	87	1.391217
ENSG0000(	IL1RL1	424	681	492	334	111	68	3.462666
ENSG0000(	HNF4A	248	414	368	612	766	727	1.613213
ENSG0000(	RASL10B	56	62	78	177	139	170	0.543774
ENSG0000(	DNPH1	2302	2887	2857	5117	3899	4636	105.468
ENSG0000(	LIMA1	4500	6314	5946	4811	2155	3091	39.39468
ENSG0000(	PIGZ	45	50	40	96	140	173	0.517715
Homo_sapi	Homo_sapi	124	125	110	79	36	45	4.810681
ENSG0000(	PARL	1060	1143	1189	958	694	918	25.81527
ENSG0000(	TRIM31	55	80	87	125	261	403	0.836416
ENSG0000(	HYOU1	12650	8010	7435	6671	4261	4292	94.28723
ENSG0000(	AHCY	8632	10237	9778	17293	14500	15411	126.7374
ENSG0000(	CDK2AP2	2632	2694	2841	2330	1964	1962	65.85378
ENSG0000(	RAB26	354	623	606	1026	994	1054	6.66384
Homo_sapi	Homo_sapi	119	149	142	160	879	794	0.995271
ENSG0000(	THRB	91	143	130	219	300	324	0.523203
ENSG0000(	CIRBP	785	1225	1136	1734	1804	2093	17.70317
ENSG0000(	UBASH3B	173	246	151	115	93	53	0.785268
ENSG0000(	ARHGAP28	87	117	101	232	190	218	1.190341
ENSG0000(	ANXA1	15376	20744	22186	17413	10944	13980	317.988
ENSG0000(	PLCL2	333	372	330	715	541	676	2.521425
ENSG0000(	AOX1	384	264	173	154	30	46	2.375948
ENSG0000(	CDH15	252	113	118	81	48	36	2.780765
ENSG0000(	SLC25A44	1249	1520	1360	1257	761	934	11.27262
ENSG0000(	CFB	2732	2237	1920	1829	1221	1209	30.12664
ENSG0000(	FARP1	2226	3509	3083	4844	4794	5222	8.749526
ENSG0000(	MYEOV	1209	1322	1381	1120	977	983	17.46872
ENSG0000(	NPHP3	125	284	218	139	51	73	1.223395
ENSG0000(	MEGF6	896	1306	1019	1695	2416	2231	5.888401
ENSG0000(	ULBP2	580	984	835	554	546	653	13.51964
Homo_sapi	Homo_sapi	155	137	133	266	361	379	1.429374
ENSG0000(	FNDC10	492	428	480	974	956	886	7.421543
ENSG0000(	AMIGO2	701	654	481	316	428	345	7.030476
ENSG0000(	SHC4	49	76	92	39	17	27	0.33445
ENSG0000(	HSH2D	49	58	101	30	19	29	1.04316
ENSG0000(	POLR1E	1014	1055	935	1785	1659	1891	17.04068
ENSG0000(	RNF125	56	131	172	45	25	59	0.28978
ENSG0000(	PXMP2	608	720	687	1551	958	1344	20.18939
ENSG0000(	RPUSD4	672	1047	954	1565	1820	1566	8.416308
ENSG0000(	TNPO1	6752	9270	8339	6691	6544	7604	20.23522
ENSG0000(	DELE1	1307	1305	1121	2436	2254	2238	11.90269
ENSG0000(	AGFG1	1741	2611	2384	1890	1615	1996	6.508155
ENSG0000(	NUCB2	2395	2814	3093	2280	2036	2477	36.74223
ENSG0000(	PTGDS	717	713	755	1035	2209	2441	28.67324
ENSG0000(	CLGN	208	294	225	118	143	188	2.141469
ENSG0000(	CPS1	11604	17402	15871	22069	30392	36739	63.02452

ENSG0000(	MATK	1473	1776	1519	3212	2445	2623	22.96848
ENSG0000(	CARS	4305	5764	5149	4455	2182	3563	53.54735
Homo_sapi	Homo_sapi	276	287	221	161	24	56	1.931999
ENSG0000(	TMEM45A	686	1088	1074	1368	3380	2648	13.45049
ENSG0000(	HES2	1923	3159	2992	1803	1907	2270	17.15263
ENSG0000(	ATP2B4	4190	6629	6109	4246	4401	4673	14.93863
ENSG0000(	SEC11A	3671	4937	4821	6884	7490	8094	92.05342
ENSG0000(	SORL1	58	45	70	126	172	189	0.166814
ENSG0000(	AC105052.	38	24	127	228	429	267	0.329207
ENSG0000(	TSPAN9	390	493	461	389	294	293	2.922243
ENSG0000(	FN1	3289	2417	2363	2096	1527	1320	12.35131
ENSG0000(	ALPG	13508	12329	11551	19329	29228	33982	170.4818
ENSG0000(	TSPAN13	2680	3902	4265	2854	2436	3063	40.28178
ENSG0000(	ACAD10	422	560	470	797	775	905	3.554316
ENSG0000(	CMIP	2028	3012	2960	4389	5004	4729	14.66264
ENSG0000(	WDR86	74	85	80	119	330	331	1.28372
ENSG0000(	RHOB	10174	12126	10024	16686	19295	20280	134.9537
ENSG0000(	NEO1	99	121	121	59	70	67	0.435292
ENSG0000(	PER3	289	511	471	736	762	1069	2.152747
ENSG0000(	ZFYVE28	199	301	328	174	172	202	1.553616
ENSG0000(	PAGE1	1462	1748	1604	1499	1039	1069	69.15653
ENSG0000(	BTBD10	691	828	771	594	546	701	9.883619
ENSG0000(	SCUBE1	55	72	81	78	452	415	0.222429
ENSG0000(	ITGB8	254	185	210	133	127	122	0.941109
ENSG0000(	EPS8L3	137	242	178	253	925	630	2.092911
ENSG0000(	SH3YL1	66	69	49	103	221	280	0.883474
ENSG0000(	SERPING1	85	94	85	17	42	45	1.331123
ENSG0000(	TCP11L1	909	1199	1047	1719	1554	1748	10.10618
ENSG0000(	POLL	467	525	506	802	1048	1006	6.660879
ENSG0000(	HIP1R	2468	2773	2593	2404	2000	2164	17.10481
ENSG0000(	SPEF2	292	408	325	506	860	927	2.706931
ENSG0000(	THG1L	490	630	614	910	1103	1120	5.224081
ENSG0000(	OSTM1	513	693	603	483	478	485	3.611014
ENSG0000(	PIEZO1	8915	13622	12238	18747	20039	19341	37.17727
ENSG0000(	SAMD9	750	794	736	610	396	641	3.47194
ENSG0000(	ASS1	53712	66467	68611	94106	119499	193388	1095.344
ENSG0000(	AKR1C1	241	410	353	234	58	149	1.242661
ENSG0000(	KYAT3	300	397	339	575	607	631	4.819142
ENSG0000(	RAB10	3931	5174	4943	4106	3768	4444	34.99046
ENSG0000(	DHRS13	558	632	509	1024	1008	991	8.977521
ENSG0000(	AC091167.	41	51	43	9	9	24	0.372924
ENSG0000(	MRPL57	1019	1451	1272	2390	1803	2059	14.26625
Homo_sapi	Homo_sapi	42	39	39	16	6	16	0.650701
ENSG0000(	INTS1	6991	8415	7199	13408	13964	11736	31.73601
ENSG0000(	RPL41	33705	36978	34150	56147	57171	59196	2192.352
ENSG0000(	TPCN1	2102	3053	2451	4287	3656	4129	12.59758
Homo_sapi	Homo_sapi	152	174	126	143	990	835	1.213022
ENSG0000(	NOP53	4664	7229	7091	9214	17237	14585	96.03291
ENSG0000(	IFI16	1649	2962	2815	1873	1649	1983	19.47103
ENSG0000(	SRRM1	2286	3283	2570	4298	4853	4602	20.23506

Homo_sapi	Homo_sapi	38	61	59	121	113	139	0.764645
ENSG0000(	RABL2A	133	99	111	257	246	282	3.026007
Homo_sapi	Homo_sapi	142	204	168	135	86	107	1.861863
ENSG0000(	COL17A1	70	49	48	24	17	26	0.420785
ENSG0000(	MESP1	68	77	79	162	175	156	0.903114
ENSG0000(	ZNFX1	4101	4442	3915	3924	2724	3032	17.8213
ENSG0000(	COL4A6	629	481	506	440	244	322	2.987923
Homo_sapi	Homo_sapi	57	81	59	190	135	135	1.530335
ENSG0000(	SIK1B	4661	5620	5077	7209	11926	11967	31.17044
ENSG0000(	TMEM50B	1218	826	782	584	559	653	14.41984
Homo_sapi	Homo_sapi	49	50	56	25	22	22	3.212302
ENSG0000(	FBLN1	13769	18249	18840	26993	27034	28049	185.7783
Homo_sapi	Homo_sapi	115	154	248	105	87	79	2.773624
ENSG0000(	AKR1C3	3666	6465	5199	4522	1585	2389	63.83611
ENSG0000(	DDX10	1156	1768	1520	1206	1072	1372	11.08023
ENSG0000(	SPX	46	54	43	25	17	18	0.630706
ENSG0000(	NOTCH2NL	1202	1682	1485	1269	1113	1367	19.31347
ENSG0000(	KIAA1217	269	460	394	211	234	311	1.173067
ENSG0000(	ATP6V1E2	257	410	370	283	215	257	4.767845
ENSG0000(	RPL23	19644	20764	19432	32440	31674	34401	477.6237
ENSG0000(	RRM2	4274	5135	4822	7119	7550	10056	38.94995
ENSG0000(	ABLIM3	646	923	927	763	440	479	4.682452
ENSG0000(	CD9	6475	7223	7077	6030	5443	6602	167.4075
Homo_sapi	Homo_sapi	82	144	93	219	191	276	2.705844
ENSG0000(	SLC50A1	3780	5543	5741	7578	9887	10118	104.1856
ENSG0000(	AKR1C2	296	506	432	319	118	244	2.485674
ENSG0000(	PPP1R3B	465	394	483	505	2097	1826	2.632865
ENSG0000(	SYNGR1	393	559	540	862	815	840	4.906978
ENSG0000(	DPM3	393	477	416	689	1061	847	27.18231
ENSG0000(	IL1B	254	202	130	53	100	114	5.08472
ENSG0000(	C4orf3	585	691	826	937	2534	1983	9.286173
ENSG0000(	SCMH1	671	883	795	1324	1128	1295	6.60131
Homo_sapi	Homo_sapi	122	324	279	111	133	139	3.522914
ENSG0000(	VPS51	2994	3972	3776	6112	6315	5499	38.70823
ENSG0000(	BCAM	561	1113	933	1998	1337	1681	6.497678
ENSG0000(	CRELD2	2768	1718	1946	1760	899	721	62.9408
ENSG0000(	BCAR3	4520	5621	4467	4133	4067	4225	44.83522
ENSG0000(	CHRNA4	276	357	434	161	263	217	2.483814
ENSG0000(	ITGA2B	109	158	179	257	377	341	1.04403
ENSG0000(	HIST1H3G	195	465	313	240	105	168	2.543561
Homo_sapi	Homo_sapi	570	734	630	1094	1103	1037	26.1145
ENSG0000(	APOL6	823	869	699	715	386	527	2.555622
ENSG0000(	PRR5L	341	435	362	1084	541	758	4.116403
ENSG0000(	DNAJC12	1693	3222	3219	1650	1613	2292	48.01136
ENSG0000(	ANXA9	137	154	132	333	263	253	2.354386
ENSG0000(	C1RL	357	463	353	646	631	872	3.867673
ENSG0000(	LYPD3	2546	3324	3199	2875	1734	1593	47.28366
ENSG0000(	TK1	3593	4753	4897	7050	6784	7265	69.69204
ENSG0000(	GPNMB	33	76	57	106	152	182	0.412552
ENSG0000(	CDT1	2007	2949	2937	3539	9456	6479	19.58376

ENSG0000(	C22orf39	577	818	656	1018	1416	1390	9.907947
ENSG0000(	TAF9B	675	666	674	997	2131	1576	7.820437
ENSG0000(	SRMS	363	402	361	306	280	260	7.521806
ENSG0000(	GPI	12723	15691	15196	22310	33617	26468	167.2497
ENSG0000(	LRRC8D	1588	2011	1705	1455	1292	1731	16.90171
ENSG0000(	PCLAF	1612	2221	2118	2890	3430	4190	24.5622
ENSG0000(	SUMF2	5375	6722	5760	11406	8367	9526	111.4978
ENSG0000(	GALNT6	197	289	269	203	115	182	1.418573
ENSG0000(	ARSG	891	987	1046	1491	1707	1926	7.652948
ENSG0000(	PXDN	3164	4435	4185	5798	8800	7570	14.92862
ENSG0000(	ATP10D	397	705	617	484	366	374	1.921482
ENSG0000(	YIPF1	967	1344	1167	1106	653	841	17.0827
ENSG0000(	CCDC191	31	36	31	68	82	158	0.238422
ENSG0000(	AKAP5	13	13	2	85	45	42	0.149124
ENSG0000(	EXOSC6	380	567	500	761	1014	900	2.319709
ENSG0000(	SPANXB1	40	59	74	30	6	18	2.677403
ENSG0000(	FRMD6	521	692	547	493	453	391	3.70968
ENSG0000(	SLC25A4	821	1083	942	1450	2019	1744	5.92444
ENSG0000(	GTF2I	2232	3640	3263	4828	5270	5288	16.0182
ENSG0000(	MANF	6263	3885	3808	3382	2682	2903	176.2563
Homo_sapi	Homo_sapi	151	190	206	306	343	393	6.523634
ENSG0000(	MCM4	6254	8490	7566	11713	10294	11557	48.57364
ENSG0000(	ADGRG6	2844	4192	4030	5288	6676	8492	13.6585
ENSG0000(	PTGS1	2078	2701	2452	2362	1719	1791	13.40311
ENSG0000(	ZNF358	596	1033	754	1365	1689	1356	9.596056
Homo_sapi	Homo_sapi	438	544	528	372	393	442	5.68779
ENSG0000(	TXNRD1	15553	24746	23976	18773	12294	18280	128.158
Homo_sapi	Homo_sapi	350	486	398	664	633	880	5.211534
ENSG0000(	NUDT14	201	300	251	507	435	411	7.453278
ENSG0000(	SH3BP5	1621	2048	2113	2689	4492	4297	18.11587
ENSG0000(	HEBP2	503	667	571	388	462	496	2.234431
Homo_sapi	Homo_sapi	63	94	76	131	183	222	0.881566
ENSG0000(	PPP1R3E	163	238	191	262	571	634	1.549206
Homo_sapi	Homo_sapi	38	101	65	153	176	166	0.709559
ENSG0000(	NLE1	938	1090	1031	1819	1725	1568	9.409922
ENSG0000(	ARNTL	609	514	544	364	433	347	7.267831
ENSG0000(	NUDT16L1	810	1224	893	2152	1727	1467	18.55843
ENSG0000(	PLEKHG5	710	942	951	694	724	662	5.948543
ENSG0000(	C9orf16	1350	1270	1318	2225	2139	2509	59.54676
Homo_sapi	Homo_sapi	138	166	125	299	220	363	4.00947
ENSG0000(	CYP2U1	39	72	49	115	110	149	0.243165
ENSG0000(	C21orf59-T	59	124	114	30	21	68	1.981762
ENSG0000(	TREX1	164	132	249	687	285	487	3.98003
ENSG0000(	LHX4	459	647	547	885	809	1086	2.479355
ENSG0000(	ARL4D	1437	1826	1628	1470	1352	1219	28.0392
Homo_sapi	Homo_sapi	433	589	517	507	1901	2282	0.992116
ENSG0000(	USP15	1167	1424	1390	1205	1090	1190	6.504344
ENSG0000(	EIF3L	5748	8427	7563	10332	13441	14082	64.65324
Homo_sapi	Homo_sapi	442	468	467	718	806	947	3.712305
ENSG0000(	SLFN5	1106	1939	1612	662	1129	1184	3.389863

ENSG0000(	ZNF581	376	467	439	652	1196	868	9.104215
ENSG0000(	TPGS1	151	206	183	356	347	299	4.244046
ENSG0000(	PCMT1	3384	4128	3938	3747	2974	3396	58.34031
ENSG0000(	PDE3A	1150	1426	1264	1946	2116	2142	4.773987
ENSG0000(	1-Mar	2656	4250	3977	6882	4789	7946	15.56393
ENSG0000(	SMDT1	253	284	287	445	472	552	5.559114
ENSG0000(	CENPM	702	922	832	1874	1108	1476	23.5906
ENSG0000(	SLIT2	53	78	78	35	40	22	0.306413
ENSG0000(	PRRX2	885	1301	1145	1071	607	651	21.23872
ENSG0000(	OASL	8329	3008	3097	386	733	104	86.96358
Homo_sapi	Homo_sapi	93	142	120	66	62	21	1.602232
ENSG0000(	CDK8	532	660	627	489	509	461	5.471994
ENSG0000(	DDX60	427	344	385	172	294	236	2.210824
ENSG0000(	RPL23A	15026	18003	17443	24413	28588	29865	306.3002
Homo_sapi	Homo_sapi	581	756	648	1173	967	1056	21.06865
ENSG0000(	LRRC56	112	134	120	226	250	231	1.267272
ENSG0000(	MMP24OS	218	252	221	380	614	451	5.442476
ENSG0000(	NALCN	319	449	307	664	522	796	3.450585
ENSG0000(	MYO1G	570	815	607	578	135	233	5.588856
ENSG0000(	FERMT2	1815	2532	2290	1948	1860	2067	17.15195
ENSG0000(	POLD3	1975	2160	2008	2978	3620	4237	17.52085
ENSG0000(	ENTPD7	410	410	386	358	261	280	3.627868
ENSG0000(	GALNT10	1089	1470	1163	1210	641	809	5.785656
ENSG0000(	PDK3	209	296	309	369	810	710	3.260989
ENSG0000(	KLHDC9	135	148	192	350	246	359	3.209657
ENSG0000(	IL6ST	1764	2024	1709	1298	1602	1401	6.137835
ENSG0000(	BCKDHA	2225	2708	2704	3774	5195	4564	33.28226
ENSG0000(	METTL26	3497	4214	4110	6846	7034	5843	164.0594
ENSG0000(	SRRM2	9471	14004	11537	21905	21430	16704	33.18563
ENSG0000(	SCARA5	2127	2439	2035	1880	1853	1896	16.89784
ENSG0000(	ZNF331	1721	2139	1851	1952	6041	7403	14.04377
ENSG0000(	EHD4	1215	1771	1637	1502	704	915	5.95436
ENSG0000(	EIF2S3B	367	539	431	704	737	780	7.198414
ENSG0000(	SH3RF2	121	172	207	133	72	95	1.255728
ENSG0000(	MPP1	667	1141	990	731	584	846	10.2776
ENSG0000(	ZNF81	253	320	301	222	203	259	1.080901
ENSG0000(	MPI	1383	1552	1474	2264	2202	2556	10.20812
ENSG0000(	ZNF467	70	73	56	39	35	26	0.86503
ENSG0000(	VKORC1	6121	6475	6773	11303	11163	9906	201.857
ENSG0000(	SYDE1	3421	4410	3687	5588	7948	6952	33.10381
ENSG0000(	WNT11	78	178	149	35	68	86	1.62899
ENSG0000(	C4BPB	2922	3089	2609	2797	1581	2052	91.64669
ENSG0000(	SH3BP4	959	1194	952	987	557	790	5.797281
ENSG0000(	RNF150	240	370	381	422	888	1018	0.844421
ENSG0000(	TUBB2A	1357	2125	1858	1720	753	765	26.75594
ENSG0000(	MARVELD1	1388	2115	1682	2459	3030	3448	13.55144
ENSG0000(	PLCXD2	170	171	209	83	96	156	0.691245
ENSG0000(	DRGX	56	81	49	40	21	15	0.636864
ENSG0000(	RPS29	2149	2561	2219	3461	3958	3906	210.2306
ENSG0000(	BDNF	109	94	89	180	205	253	0.821023

ENSG0000(	TRIM38	829	1170	1192	757	845	950	2.769245
ENSG0000(	NEGR1	183	201	171	383	323	319	0.449151
ENSG0000(	CLCF1	451	429	355	361	213	285	7.904652
ENSG0000(	PCED1A	821	1145	1099	1596	1569	1670	13.83892
ENSG0000(	ACCS	91	109	105	224	165	204	1.263238
ENSG0000(	RPS3	71274	85319	79007	117031	128944	125965	1740.282
ENSG0000(	FAM120AC	1281	1337	1149	2195	1858	2250	17.94888
ENSG0000(	TEF	129	281	306	511	407	601	0.924576
ENSG0000(	CHRAC1	1745	2473	2240	3214	3149	3575	22.75834
ENSG0000(	DNAJB11	2821	2071	2096	1701	1628	1792	52.57225
ENSG0000(	NPR3	1007	1473	1349	1777	2740	2585	5.952109
ENSG0000(	RTN4	10604	13956	13122	12382	10480	11526	142.1147
ENSG0000(	FAM162A	1877	2368	2649	3146	5811	4951	21.97848
ENSG0000(	HAUS4	1546	2193	2200	2779	3640	4126	31.43253
ENSG0000(	CDK18	1090	2027	1961	2355	4509	3816	12.05409
ENSG0000(	ANKRD33B	1697	2305	1912	1735	1588	1938	5.828272
ENSG0000(	ARHGAP29	911	1305	1316	1000	886	1087	4.754698
ENSG0000(	HDAC6	2118	3786	3527	4106	8113	7600	17.15535
ENSG0000(	REEP1	349	393	289	316	152	181	2.947551
Homo_sapi	Homo_sapi	39	52	50	15	25	23	0.586539
ENSG0000(	NRARP	529	652	601	825	1322	1264	9.388948
Homo_sapi	Homo_sapi	235	406	343	515	706	625	1.631435
ENSG0000(	PLXNA2	1905	2428	1979	2102	1223	1175	5.236767
ENSG0000(	PTPRE	317	468	383	357	266	276	1.96385
ENSG0000(	CRIPT	1048	1583	1368	1223	1066	1164	16.87788
ENSG0000(	PDE1A	2136	2822	2673	3745	3802	5528	26.21348
ENSG0000(	TMEM267	1080	1430	1318	1147	829	1226	16.34483
ENSG0000(	TMEM33	1539	2048	2112	1782	1488	1678	7.728608
ENSG0000(	SCN1B	78	92	130	56	35	72	1.369239
Homo_sapi	Homo_sapi	64	68	85	44	27	47	2.132794
ENSG0000(	LMO4	945	1028	908	759	721	918	6.182028
Homo_sapi	Homo_sapi	491	447	345	276	294	340	4.029689
ENSG0000(	CLDN4	3265	3038	2933	2758	2355	2024	28.01902
ENSG0000(	HHLA3	346	589	514	874	678	975	14.12474
ENSG0000(	ADRB2	163	135	155	98	85	116	1.480719
ENSG0000(	NFKBIA	1933	2770	2119	2271	6956	8455	39.09252
ENSG0000(	GATD1	1008	1367	1241	1887	1670	1938	9.610373
ENSG0000(	RPL37	58618	73134	67607	94936	112275	112333	318.4577
ENSG0000(	FBXO8	435	543	646	391	388	482	5.305209
ENSG0000(	NAA50	4858	6570	5615	5476	4894	4991	35.3116
ENSG0000(	STIM1	1662	2607	2368	2169	1432	1527	12.92655
ENSG0000(	CHUK	780	835	799	637	662	718	6.764938
ENSG0000(	ZNF580	183	261	206	336	530	424	4.128082
ENSG0000(	NPHP4	659	898	710	1106	1345	1374	4.128279
ENSG0000(	NRBP2	966	1218	1032	1565	1746	1891	8.723918
ENSG0000(	OXA1L	3505	4173	4075	6264	5610	5957	48.42026
ENSG0000(	PRODH	52	88	75	169	119	160	0.672303
ENSG0000(	TTC3	1341	1740	1580	2134	2766	3222	4.980544
ENSG0000(	CHORDC1	1720	1978	1826	1325	1566	1684	21.14654
ENSG0000(	NPIPB12	20	69	41	128	95	122	0.330201

ENSG0000(	CTSC	2249	3173	3219	2846	1949	2168	35.1138
Homo_sapi	Homo_sapi	79	127	142	176	269	350	1.115456
ENSG0000(	CACNG8	41	79	53	17	29	32	0.146412
ENSG0000(	CAMSAP2	1987	2749	2344	2190	2025	2209	9.521957
ENSG0000(	UGDH	2596	4342	3834	3452	2135	2761	26.20639
ENSG0000(	C16orf45	1865	2534	2364	2227	1721	2027	25.74946
ENSG0000(	INSIG1	1701	1565	1803	2361	4162	3759	18.95374
ENSG0000(	EIF2D	2793	3996	3433	5096	4711	5593	43.99255
ENSG0000(	ACBD4	592	957	836	1165	1365	1540	10.57202
ENSG0000(	MTMR2	2634	3508	2969	3042	2204	2714	22.33547
ENSG0000(	TDRD7	414	513	496	396	369	436	3.392646
ENSG0000(	SQOR	4081	4656	3956	4315	2474	2958	64.87163
ENSG0000(	MYZAP	294	283	268	199	195	241	3.985211
ENSG0000(	OAF	1131	1489	1455	1334	1034	1070	14.88493
ENSG0000(	ZBTB7C	313	452	309	322	231	223	2.039171
ENSG0000(	CACHD1	50	61	50	107	117	121	0.273298
ENSG0000(	DSEL	958	1262	1100	1395	2716	2629	3.160331
ENSG0000(	NPNT	1481	2044	1808	2371	3740	3674	10.33721
ENSG0000(	CTSH	589	673	643	1219	823	1235	8.626685
ENSG0000(	RPS6	40317	53087	48730	65641	79526	84576	922.6873
ENSG0000(	HMGCL	1245	1856	1644	2356	2311	2737	24.79439
ENSG0000(	PRKCSH	6633	9181	8044	12453	13402	11854	98.21412
ENSG0000(	ARHGAP44	98	112	120	209	184	226	0.732859
ENSG0000(	SH3D21	203	328	284	373	1032	581	2.411802
Homo_sapi	Homo_sapi	372	428	271	909	590	627	3.412852
ENSG0000(	FBXW9	530	687	654	1128	886	982	9.633885
ENSG0000(	TTC12	454	440	304	890	601	900	8.820327
ENSG0000(	HSD17B8	934	1039	1100	1668	1427	1869	29.67649
ENSG0000(	ZNF860	123	120	144	80	88	90	1.242862
ENSG0000(	ITGAD	17	28	20	77	59	48	0.130786
ENSG0000(	ITGA7	123	116	125	212	251	253	1.209586
ENSG0000(	PLXND1	3944	4944	4638	7107	8971	6959	17.5305
ENSG0000(	DCAKD	507	912	696	1316	976	1315	8.252637
ENSG0000(	OCIAD2	845	991	968	880	767	759	23.78158
ENSG0000(	IL7R	294	133	101	120	32	27	2.09205
ENSG0000(	FAM126A	159	160	164	92	105	134	0.443561
ENSG0000(	ALDH3B1	4628	5531	4526	11569	6295	8793	59.10807
Homo_sapi	Homo_sapi	104	193	126	99	89	87	0.639053
ENSG0000(	CMAS	1629	2572	2266	1934	1614	2019	28.90056
ENSG0000(	HR	3633	4104	3803	3776	3117	3059	19.04809
ENSG0000(	UNC5B	547	871	997	308	587	599	2.546964
ENSG0000(	SIPA1L2	419	702	666	472	469	440	2.417769
ENSG0000(	THEM6	1390	1752	1675	2685	2799	2358	19.35513
ENSG0000(	SLCO3A1	2881	3156	2664	2941	1819	1961	19.38256
ENSG0000(	BDH1	195	292	272	492	353	479	1.730935
ENSG0000(	LETMD1	1734	1958	1827	2177	5130	4745	31.22233
Homo_sapi	Homo_sapi	12	24	25	45	55	89	0.304677
Homo_sapi	Homo_sapi	27	38	80	77	181	219	0.862033
ENSG0000(	GRAMD1B	3016	5476	4705	2746	3639	3502	27.6746
ENSG0000(	BMF	479	553	602	503	382	309	3.41097

Homo_sapi	Homo_sapi	25	19	48	59	114	132	0.458182
ENSG0000(	ABI1	950	1236	1198	926	906	1111	8.555173
ENSG0000(	FZD10	2541	3408	2618	3819	6814	5761	24.35586
ENSG0000(	SPRYD4	601	674	705	1027	975	1456	1.75281
ENSG0000(	SH3BGRL	1277	1697	1407	2135	2163	2444	20.88659
ENSG0000(	PDP2	769	775	839	1228	1351	1435	3.805008
ENSG0000(	TMEM132/	1198	1321	1218	2488	2148	1793	11.33408
ENSG0000(	CCHCR1	1711	2194	2313	3244	2868	3615	19.34733
ENSG0000(	RNF223	131	82	148	135	784	378	2.164568
ENSG0000(	CDKN2AIP	594	768	730	992	1130	1469	6.973767
ENSG0000(	PAPLN	51	71	61	91	152	205	0.268391
ENSG0000(	ABCG2	586	571	638	556	137	278	4.241573
ENSG0000(	CHST15	1036	1420	1144	1215	774	873	7.143177
ENSG0000(	POM121C	1893	2173	1830	3800	3174	2873	10.55942
ENSG0000(	NMB	352	624	485	463	225	281	10.96617
ENSG0000(	LAT2	116	190	155	104	113	76	1.798489
ENSG0000(	THAP8	76	104	114	190	176	193	1.515186
ENSG0000(	NUAK2	459	541	384	741	756	921	4.194122
ENSG0000(	MCM5	3862	5079	4627	9379	6468	6696	34.60174
ENSG0000(	MEOX1	92	67	127	261	150	298	1.455417
ENSG0000(	ZBTB12	216	310	219	486	370	436	3.621546
ENSG0000(	KLHL23	303	413	353	503	647	761	2.330864
ENSG0000(	DHRS2	823	812	752	178	644	129	15.18548
ENSG0000(	LSR	265	310	333	202	251	148	3.906773
ENSG0000(	RORC	400	848	772	871	1944	1653	5.646338
ENSG0000(	RBM47	820	958	933	775	732	877	6.322841
ENSG0000(	FURIN	8418	11481	9805	10337	5770	5972	62.47202
ENSG0000(	FHDC1	166	237	191	151	101	172	0.789557
ENSG0000(	RNF227	150	216	205	277	407	413	1.703785
ENSG0000(	DHRS3	148	195	262	201	793	743	2.801775
ENSG0000(	TLE2	1896	2657	2338	3837	3029	3589	23.54421
ENSG0000(	PDIA3	31810	33737	33455	32250	27000	26287	268.7085
ENSG0000(	ANO7	41	22	69	97	156	156	0.310159
ENSG0000(	PROM2	243	460	438	343	98	109	1.616735
Homo_sapi	Homo_sapi	496	623	620	678	1742	1487	12.57403
ENSG0000(	TMEM94	1205	1980	1725	2736	2415	2593	7.755452
Homo_sapi	Homo_sapi	45	126	80	23	34	55	0.865966
ENSG0000(	XK	581	753	692	1050	931	1336	3.50916
ENSG0000(	TMEM50A	2590	3548	3456	3161	2508	2889	30.37272
ENSG0000(	IDH1	4398	6633	7203	4849	3781	5885	58.64783
ENSG0000(	AL355987.:	33	30	51	69	113	127	1.411297
Homo_sapi	Homo_sapi	21	50	73	295	77	128	1.906672
ENSG0000(	B4GALT5	4629	4523	3978	3766	3605	3663	30.84068
ENSG0000(	WWC2	748	1004	999	1167	2148	2068	2.912446
ENSG0000(	LURAP1L	532	470	410	305	388	310	9.533251
ENSG0000(	HERC5	2018	1934	1811	769	1590	1413	18.06674
ENSG0000(	NOX5	83	152	115	93	23	25	0.66102
Homo_sapi	Homo_sapi	771	1137	1059	1280	2126	2179	16.62472
ENSG0000(	NONO	14743	20326	18490	25511	26181	28409	177.6593
ENSG0000(	WFDC3	60	95	75	162	273	125	2.414901

ENSG0000(	WFDC1	82	62	98	89	322	352	1.702544
ENSG0000(	RPL6	36121	44475	40056	60379	54093	61294	1189.315
ENSG0000(	RPL29	35787	37969	33928	53387	58893	61551	1555.541
ENSG0000(	BMP2	2424	3109	3043	3326	6754	7617	21.17264
ENSG0000(	MCM6	2915	3862	3608	5056	4721	5956	24.55087
ENSG0000(	AVPR2	44	78	82	49	26	27	0.850847
Homo_sapi	Homo_sapi	22	27	31	60	76	67	0.755063
ENSG0000(	RETREG1	248	284	296	151	165	266	2.47649
ENSG0000(	SPINK5	569	673	602	994	869	2133	4.932953
ENSG0000(	PRDX2	12858	14473	13529	22807	18694	20856	422.6605
ENSG0000(	KCTD7	642	907	870	976	1923	2001	3.853291
ENSG0000(	ARHGAP26	176	262	269	216	131	122	0.905241
ENSG0000(	PCNX2	432	593	407	360	369	420	2.261999
ENSG0000(	CTXN1	230	279	244	496	409	389	5.656301
Homo_sapi	Homo_sapi	3951	4854	3949	7672	6858	6051	181.9875
ENSG0000(	RIMS3	152	341	466	178	117	239	0.65943
Homo_sapi	Homo_sapi	1566	2449	1891	2681	3265	4377	7.220347
Homo_sapi	Homo_sapi	284	239	240	269	916	865	2.383828
ENSG0000(	RAVER2	306	490	443	545	995	903	2.233253
ENSG0000(	SCPEP1	668	731	763	730	439	505	10.95458
Homo_sapi	Homo_sapi	347	344	332	471	691	854	3.671758
ENSG0000(	LAPTM4A	6742	9584	9512	7755	6967	8428	120.1843
ENSG0000(	OTOG	17	20	23	48	39	120	0.216989
ENSG0000(	METRN	1188	1364	1223	2608	1806	1918	24.84987
ENSG0000(	ACSF3	1650	2222	2088	3175	2838	2935	18.44128
ENSG0000(	BRINP3	95	116	127	218	198	203	1.751359
ENSG0000(	PSMD7	6746	8689	7338	7824	5458	6815	130.3565
ENSG0000(	LUM	801	676	807	764	311	405	8.374398
ENSG0000(	FCGRT	739	944	814	1254	1368	1313	14.63095
ENSG0000(	MAP2K6	4875	7070	5991	7336	11785	14743	21.76508
ENSG0000(	MFSD1	1184	1217	1180	1191	844	945	15.90272
ENSG0000(	MGRN1	1725	2428	2093	3432	3242	2984	12.50598
ENSG0000(	EIF4E	1416	1677	1479	1515	1071	1399	12.74639
Homo_sapi	Homo_sapi	97	189	197	262	411	332	2.819177
Homo_sapi	Homo_sapi	69	132	156	147	419	367	1.146068
Homo_sapi	Homo_sapi	359	375	388	211	313	294	15.81596
ENSG0000(	SLC6A14	18	38	29	35	107	148	0.121826
ENSG0000(	AL157935.:	968	1477	1552	693	734	1360	10.60167
ENSG0000(	AMFR	980	1041	1043	966	833	770	8.563464
ENSG0000(	RAB4A	994	1389	1262	1754	1780	2050	10.63048
ENSG0000(	CBFA2T3	1224	1806	1568	1436	1199	918	10.64621
ENSG0000(	CRLF2	222	174	152	155	36	32	4.23107
ENSG0000(	CDC37L1	752	900	787	721	703	727	6.754931
Homo_sapi	Homo_sapi	385	528	474	700	825	757	7.649493
ENSG0000(	MYO16	14	37	27	45	75	117	0.055067
ENSG0000(	SH3PXD2A	1459	2043	1708	2799	2289	2711	4.001461
ENSG0000(	C1QTNF6	690	853	664	2288	941	1326	8.062093
ENSG0000(	INF2	4078	5435	4764	8735	6849	6774	25.74181
ENSG0000(	LAMA3	631	922	822	1146	1239	1362	1.979881
ENSG0000(	CCDC14	956	1446	1440	1249	3934	3981	7.485459

ENSG0000(	RAPGEF3	591	856	844	537	661	639	4.040947
ENSG0000(	MOB3C	795	922	877	871	639	646	7.40782
ENSG0000(	RPL13A	53139	68453	69355	87804	116921	110665	1419.158
ENSG0000(	GRHL3	49	82	41	33	24	32	0.663342
ENSG0000(	FAM98A	1189	1552	1322	1384	959	1167	13.28743
ENSG0000(	CCDC22	987	1379	1238	1776	1853	1881	13.37985
ENSG0000(	ISG15	10312	5899	7789	2744	6979	3775	507.6144
ENSG0000(	KYAT1	969	1380	1622	2138	2016	2457	14.11691
ENSG0000(	PRKAA2	296	390	440	440	1020	1007	0.994846
ENSG0000(	BTF3L4	1545	2305	2121	3074	2655	3829	17.06062
ENSG0000(	DEAF1	825	787	670	1468	1197	1279	10.67883
ENSG0000(	C1orf115	174	304	367	432	588	652	1.596624
ENSG0000(	ANXA3	2872	3153	2815	2714	1548	2753	54.56351
ENSG0000(	ZFAND6	2584	3241	2873	2299	2280	3042	53.9603
Homo_sapi	Homo_sapi	162	204	306	127	157	161	4.006507
ENSG0000(	ABTB2	1311	1607	1499	1466	1202	1141	8.411695
ENSG0000(	HIST3H2BB	59	157	121	46	61	76	0.78523
ENSG0000(	KCTD1	521	636	559	555	447	469	6.351668
ENSG0000(	CRTAP	9918	14060	13414	17921	18845	19917	47.70528
ENSG0000(	WDR90	1360	1730	1704	2397	2567	2454	8.990385
ENSG0000(	WDR54	1320	1949	1711	2799	2390	2435	37.22017
ENSG0000(	SDF2L1	3353	2107	1779	2249	1141	1021	121.9459
ENSG0000(	FAM207A	1458	1519	1187	2264	3393	2254	50.66876
ENSG0000(	DMC1	21	17	31	49	61	93	0.271617
ENSG0000(	FOXF1	690	951	730	1186	1344	1275	6.167667
ENSG0000(	KCNC4	884	1118	963	969	807	897	4.289344
ENSG0000(	RAB29	2056	2756	2427	2036	1967	2505	21.95633
ENSG0000(	CYR61	2075	2270	2216	2448	5745	5670	28.51009
ENSG0000(	PTK2B	707	1176	1246	867	778	864	5.296657
ENSG0000(	AKAP12	1153	1735	1639	1322	5228	4815	5.230412
ENSG0000(	IL4R	1965	2617	2141	2284	1590	1370	18.23512
ENSG0000(	EIF3F	5680	8054	7408	10502	9955	10640	137.4584
Homo_sapi	Homo_sapi	38	41	37	18	4	21	1.051763
ENSG0000(	BTRC	1456	1943	1836	1128	1360	1754	7.57362
ENSG0000(	NBN	676	869	695	612	574	746	4.549384
ENSG0000(	CITED2	1583	1771	1884	1831	5383	4628	21.16201
ENSG0000(	TSPAN15	1388	1748	1458	1611	973	1072	25.73664
ENSG0000(	MB21D2	101	147	111	59	87	87	0.937875
ENSG0000(	CNKSR1	354	509	418	379	308	399	4.522308
ENSG0000(	BNIP3	2645	3613	4183	3244	14324	9043	28.96534
ENSG0000(	DAB2	2187	4320	3824	7835	4284	6853	15.79958
ENSG0000(	COL12A1	1181	1186	1031	3421	1648	1795	3.225781
Homo_sapi	Homo_sapi	65	98	57	38	36	54	1.330167
ENSG0000(	FN3KRP	1560	2278	2063	3029	2687	3059	27.08342
ENSG0000(	NRM	771	1205	1175	1857	1661	1566	15.30435
ENSG0000(	CACNA1C	44	54	41	29	13	27	0.101287
Homo_sapi	Homo_sapi	1167	1466	1320	1309	1000	1267	9.939991
Homo_sapi	Homo_sapi	46	91	90	93	312	212	0.727386
ENSG0000(	SPRED1	114	130	94	76	81	68	0.459047
ENSG0000(	DHRS11	242	437	388	706	512	617	4.970959

ENSG0000(	TYMP	1610	2164	1902	1999	1093	1007	32.04357
ENSG0000(	PDGFRL	39	42	43	88	89	90	0.644185
ENSG0000(	C12orf66	421	664	591	452	377	545	3.773402
ENSG0000(	CARD19	391	609	694	523	370	362	13.1724
ENSG0000(	WAS	22	52	35	66	92	109	0.360551
ENSG0000(	ARPC1A	3528	4459	4216	4369	2328	2838	70.92452
Homo_sapi	Homo_sapi	180	322	189	248	707	702	1.455424
ENSG0000(	MND1	205	322	325	451	446	554	6.677672
ENSG0000(	TMEM61	47	78	79	41	38	43	1.325185
ENSG0000(	ENAH	3999	4946	4601	4773	3297	3806	13.07872
ENSG0000(	NECTIN4	105	131	126	53	86	92	0.936851
ENSG0000(	FN3K	450	813	761	1200	1233	1037	9.852893
ENSG0000(	ANXA7	7623	8755	7582	8059	5291	7386	98.60012
ENSG0000(	HEATR5A	1421	2026	2017	1377	1332	1880	6.089987
ENSG0000(	TLR6	180	117	123	106	64	92	1.056953
ENSG0000(	C5orf15	1696	2114	1926	2684	2783	3252	22.63058
ENSG0000(	ASIC1	279	319	408	673	506	572	2.310822
ENSG0000(	TDP2	1669	2530	2377	1959	1806	2087	25.33871
ENSG0000(	TMEM97	1440	1854	1651	2566	2129	2624	17.79271
ENSG0000(	DNAJC3	4247	2923	2705	1504	2559	2564	23.90026
ENSG0000(	SIX1	188	177	161	368	271	324	1.738819
ENSG0000(	VPS50	473	587	495	436	349	520	4.179823
ENSG0000(	MTCL1	639	899	652	1287	993	1194	3.957358
ENSG0000(	RNASEH2A	6029	7750	7439	12769	9224	10690	69.32349
ENSG0000(	GNAZ	92	134	141	212	198	256	0.86798
ENSG0000(	RAB40A	30	31	26	59	55	137	0.296877
ENSG0000(	ERO1A	2573	3298	3518	2882	12503	7998	19.37014
ENSG0000(	RPL7A	87013	106259	105167	145293	146259	149828	3072.237
ENSG0000(	NES	1694	3185	3010	1827	2150	2143	9.588815
ENSG0000(	FIBCD1	1722	2438	2558	3543	3682	3403	23.30201
ENSG0000(	SLC6A9	1487	2007	1772	1803	1226	1582	17.00156
ENSG0000(	GOLM1	2476	3715	3228	4801	4186	4961	25.65687
ENSG0000(	KIF3B	1699	2417	2001	2028	1716	1757	8.756199
Homo_sapi	Homo_sapi	133	214	159	252	283	430	0.812599
ENSG0000(	TMEM101	841	960	1048	1312	2090	1727	17.16482
ENSG0000(	AKNA	360	562	539	336	368	458	1.73253
Homo_sapi	Homo_sapi	149	125	110	153	499	354	2.432737
ENSG0000(	SH2D3C	106	152	173	85	106	105	1.057599
ENSG0000(	MST1R	2185	2568	2331	2426	1888	2111	15.95382
ENSG0000(	RPL3	51136	62984	59676	78197	99787	99498	1150.154
ENSG0000(	B4GALT4	1553	1807	1603	1601	1419	1528	19.81775
ENSG0000(	RAB40C	1498	1465	1424	1722	5093	3091	17.98357
ENSG0000(	PDXP	957	884	908	1350	1833	1681	14.98549
ENSG0000(	FAM216A	743	900	720	1402	1248	1149	14.81014
ENSG0000(	TRIM5	520	737	720	544	530	633	5.435242
ENSG0000(	ARNT2	387	648	677	147	463	332	1.817438
ENSG0000(	SCARB1	4860	6053	5228	9450	8292	7432	56.89081
ENSG0000(	HP1BP3	4214	5395	4784	6225	8682	9083	51.26598
ENSG0000(	CCDC24	334	501	408	643	612	688	7.034351
Homo_sapi	Homo_sapi	59	46	82	156	137	125	2.239128

ENSG0000(	SUMF1	880	1253	1116	1589	1544	1726	12.86068
ENSG0000(	PHOSPHO1	598	903	941	829	214	348	9.680271
ENSG0000(	ALPI	336	384	461	393	7682	8352	4.360376
ENSG0000(	RPL28	8111	10006	10161	15527	15187	13232	110.3467
ENSG0000(	GOLGA5	1140	1459	1312	1185	1078	1337	12.11001
ENSG0000(	LAMB3	6878	8898	8301	6366	7560	7182	54.73017
ENSG0000(	ACTR3	7522	10266	9200	9117	6860	8899	36.43615
ENSG0000(	KLHL5	1349	1966	1498	1453	1123	1616	13.35363
ENSG0000(	PSMD14	2779	2843	2576	2820	1740	2244	44.93173
ENSG0000(	LIG1	1413	1752	1618	3118	2159	2373	11.82949
Homo_sapi	Homo_sapi	31	70	62	52	284	186	0.246135
ENSG0000(	ECM2	79	63	73	58	16	30	0.759388
ENSG0000(	ADGRE2	1199	1830	1485	1310	1090	528	8.172272
ENSG0000(	SIK1	4611	6371	5762	6475	13580	12065	30.18992
Homo_sapi	Homo_sapi	33	46	33	14	16	24	0.808554
ENSG0000(	TLE6	340	518	559	838	775	739	5.605236
ENSG0000(	ISCU	2311	3151	2874	2833	2326	2328	70.12903
ENSG0000(	CXorf38	2454	3468	3063	2950	2597	2849	18.49028
ENSG0000(	ZSCAN32	885	967	904	907	636	846	10.06831
ENSG0000(	EXD3	462	354	370	639	802	797	10.82116
ENSG0000(	INPP5K	636	903	941	787	614	730	7.675981
ENSG0000(	ARL4C	45	64	48	73	130	170	0.351434
ENSG0000(	CAP1	6707	9387	8523	7531	5736	8590	94.65776
ENSG0000(	FKBP9	3325	4415	3639	3917	2701	3523	31.31443
ENSG0000(	WDR25	338	536	526	444	330	314	5.063739
ENSG0000(	AC009779.	140	145	159	313	199	314	1.784152
ENSG0000(	DPP7	4753	5088	4888	8472	7208	7173	98.47365
ENSG0000(	GARS	7104	10460	9535	8101	5698	9295	85.48422
ENSG0000(	RACK1	47180	55910	55575	74623	79491	83316	1211.145
ENSG0000(	SLC11A1	62	58	37	91	117	171	0.504937
Homo_sapi	Homo_sapi	85	166	108	216	190	265	0.577967
ENSG0000(	MOB4	919	1164	1035	1017	876	993	8.292137
ENSG0000(	RASIP1	60	71	57	40	40	36	0.567225
ENSG0000(	SEL1L	1977	1345	1378	928	1256	1232	8.009946
ENSG0000(	SMIM26	503	729	625	919	938	983	28.54152
ENSG0000(	HLA-B	6126	6111	6678	9547	9583	10493	124.5767
ENSG0000(	MZT2B	1924	2211	2246	3902	3042	3068	66.40123
ENSG0000(	TRIM45	376	397	368	568	583	790	3.695538
ENSG0000(	SHLD1	103	124	102	70	78	86	3.837809
ENSG0000(	LRIG1	351	494	517	293	393	371	2.092722
ENSG0000(	UNG	3959	5335	4731	7323	6016	6967	59.41874
ENSG0000(	PAM	3129	4668	4132	5767	6424	6066	24.66128
ENSG0000(	ID3	4225	5859	5722	5482	1602	3182	106.2464
ENSG0000(	TACSTD2	734	745	636	1437	1003	1149	9.815528
ENSG0000(	SLC7A5	24985	31317	25030	54200	35620	41287	173.2768
Homo_sapi	Homo_sapi	2825	3081	3335	5624	5063	4289	145.2367
ENSG0000(	SFMBT2	442	406	441	656	682	920	1.8921
ENSG0000(	RECQL4	2377	3022	2914	3830	4331	4512	20.21821
ENSG0000(	USP37	416	623	437	721	992	829	1.736486
ENSG0000(	PIP4K2C	1748	2104	1827	1984	1458	1524	19.58204

ENSG0000(	EEF2	59965	88434	93973	95304	197827	171473	597.2706
ENSG0000(	RNF144B	129	124	144	113	65	99	0.805686
ENSG0000(	METTL7A	16	56	41	58	106	155	0.117804
ENSG0000(	DNAJC10	1924	1946	1937	1866	1503	1833	3.530227
ENSG0000(	CEP131	957	1295	1078	1753	1509	1662	8.670375
ENSG0000(	C20orf194	62	76	79	51	47	28	0.283525
ENSG0000(	HTRA1	484	583	523	815	720	1318	7.332616
ENSG0000(	ACTG1	82687	94873	94881	149314	130276	129420	1575.763
ENSG0000(	CD276	4133	5925	6003	5101	4566	3929	39.67359
ENSG0000(	PDE9A	548	608	557	762	1065	1140	8.939086
ENSG0000(	WARS2	517	645	572	1051	752	928	5.801101
Homo_sapi	Homo_sapi	106	45	33	37	14	23	3.103094
ENSG0000(	IFITM1	2034	2971	2961	4035	3909	4098	76.07267
ENSG0000(	VBP1	1357	1877	1817	1597	1222	1693	25.32449
ENSG0000(	MXRA7	2071	2418	2339	3455	3128	3436	20.61291
ENSG0000(	ACKR3	828	1017	931	1086	2185	2033	13.65485
ENSG0000(	PLEK2	883	1046	1026	963	846	802	17.91692
ENSG0000(	RBMS3	73	96	101	134	208	194	0.289452
ENSG0000(	SDHAF1	225	247	251	412	704	359	6.317609
ENSG0000(	VGLL4	962	1257	1060	1360	2410	2156	9.292095
ENSG0000(	TPST1	655	919	802	647	703	740	9.733468
ENSG0000(	EEF1G	44173	50022	52997	66271	84909	85761	912.6191
ENSG0000(	NTN4	1431	1911	1606	1212	1421	1642	12.57343
ENSG0000(	TNNC1	622	510	440	1867	595	1159	27.38018
ENSG0000(	GTF2E1	712	1070	1024	846	723	902	7.437074
ENSG0000(	TRABD2B	152	221	185	270	300	439	0.676081
ENSG0000(	TSPAN3	767	958	912	1198	1540	1520	4.786763
ENSG0000(	CISD2	1484	1883	1747	1705	1497	1545	15.69204
Homo_sapi	Homo_sapi	818	947	840	1114	2486	1586	22.60494
Homo_sapi	Homo_sapi	82	62	52	0	28	8	1.238069
ENSG0000(	BBS12	116	220	163	99	124	128	1.122648
ENSG0000(	SLC25A16	253	144	108	338	376	490	1.958569
ENSG0000(	SLC33A1	1460	1701	1627	1265	1381	1614	8.159484
ENSG0000(	EIF3H	8459	11102	10865	13149	17299	18606	69.82205
Homo_sapi	Homo_sapi	131	214	109	202	333	430	0.852891
ENSG0000(	GUSB	2403	2754	2777	3928	3634	4142	33.31617
ENSG0000(	PPP1R15B	5303	7040	5973	7154	11069	15880	31.92268
ENSG0000(	SH3RF1	479	501	542	353	429	467	2.856389
ENSG0000(	SMIM14	753	1386	1226	792	753	1117	3.729133
ENSG0000(	GAMT	1831	2559	2348	4977	3029	3287	48.13325
ENSG0000(	OTX1	275	313	293	530	414	475	3.171837
ENSG0000(	MUC5B	83	101	163	267	321	173	0.145473
ENSG0000(	CD58	870	1086	1170	978	894	920	24.81856
Homo_sapi	Homo_sapi	31	29	41	66	67	110	1.369846
ENSG0000(	EFNA3	539	530	637	651	1789	1249	9.512918
ENSG0000(	F8A2	32	60	137	297	122	224	0.898365
ENSG0000(	RAET1E	261	286	282	279	158	128	4.415599
ENSG0000(	SKAP2	737	1448	1403	809	892	1062	5.798473
ENSG0000(	ST6GALNA(	722	669	560	1506	1339	869	9.499747
ENSG0000(	MFAP3	713	905	691	548	669	713	8.78169

ENSG0000(	FUNDC2	2282	2770	2723	2603	2002	2603	11.16249
ENSG0000(	ATP1A1	16875	24817	22459	21950	17854	18282	144.9167
Homo_sapi	Homo_sapi	87	99	61	61	49	29	1.334031
ENSG0000(	FAM214B	611	610	475	432	497	325	6.505712
ENSG0000(	PPP2CB	1664	2048	1923	1740	1699	1857	26.26058
ENSG0000(	MTMR10	623	693	703	652	445	641	3.716763
ENSG0000(	RPL34	5134	5592	5120	7640	8076	8423	346.2098
ENSG0000(	SRPRA	6256	7443	6742	7218	5362	6317	64.27644
ENSG0000(	KLHL29	358	558	464	462	323	323	2.360221
ENSG0000(	NPAS2	2867	3664	2948	2870	2926	2427	24.02956
ENSG0000(	OGG1	1018	1214	1167	2217	1416	1834	16.83964
ENSG0000(	SMIM10	521	765	643	903	973	1109	10.66606
ENSG0000(	PLOD2	1575	1908	2062	1970	6022	3904	13.40605
ENSG0000(	ZBED3	556	775	724	1331	863	1114	2.833501
ENSG0000(	TAF13	1169	1558	1455	1386	1208	1305	41.37772
ENSG0000(	PABPC1	18760	30704	28957	33841	53129	47452	172.0314
ENSG0000(	DTL	2114	2666	2691	3098	4325	5413	14.58408
Homo_sapi	Homo_sapi	110	173	122	196	235	328	1.106657
Homo_sapi	Homo_sapi	229	169	175	173	23	29	15.62645
ENSG0000(	PAOX	86	145	141	257	194	215	1.516937
ENSG0000(	PIAS2	297	400	364	428	878	738	1.24835
ENSG0000(	SPIRE1	806	1033	957	757	856	864	4.565183
Homo_sapi	Homo_sapi	412	473	422	508	943	1085	3.563284
ENSG0000(	C21orf58	312	495	390	527	682	868	3.812753
ENSG0000(	GADD45A	662	928	836	430	717	687	14.16516
ENSG0000(	DHRS7	1350	1648	1572	1275	1403	1457	31.99193
ENSG0000(	C1QL4	102	195	177	233	310	333	1.546711
Homo_sapi	Homo_sapi	329	489	517	383	1301	1490	2.672538
ENSG0000(	SNCA	261	432	454	537	602	1060	5.781005
ENSG0000(	CLMN	45	77	62	26	36	45	0.110852
ENSG0000(	ARHGEF26	142	206	119	301	252	279	0.84604
ENSG0000(	VDAC1	19121	23720	20785	31569	30467	30304	325.2594
ENSG0000(	SPOPL	253	316	303	398	560	539	1.321049
ENSG0000(	PDCD7	799	931	872	1202	1553	1447	8.806116
ENSG0000(	HPCAL1	3332	4043	3904	5256	6453	5858	55.57755
ENSG0000(	IMPDH2	6953	9917	10528	14001	14233	13762	133.651
ENSG0000(	THAP3	836	898	914	1334	1402	1376	21.82853
ENSG0000(	TICAM2	179	120	90	44	94	76	1.841128
ENSG0000(	HPCAL4	40	147	85	38	37	63	0.253965
ENSG0000(	SGSM2	1013	1174	901	1884	1461	1574	7.264293
ENSG0000(	BAZ1A	1178	1407	1302	1300	1125	1172	6.291249
ENSG0000(	GAA	1318	1360	1183	2317	1889	1955	12.13188
ENSG0000(	KIAA1549	2275	2751	2104	2340	2005	1811	6.964967
Homo_sapi	Homo_sapi	115	127	153	239	196	264	1.028223
ENSG0000(	DES	416	555	451	495	257	355	5.822776
ENSG0000(	DIPK1A	42	50	37	17	22	30	1.274679
ENSG0000(	SRSF6	4984	6482	5481	7912	7542	9342	46.94989
Homo_sapi	Homo_sapi	34	26	24	41	83	116	0.326034
Homo_sapi	Homo_sapi	22	28	57	60	100	127	0.398227
ENSG0000(	MFAP2	695	1204	1279	1739	1679	1771	17.48147

ENSG0000(	SH3KBP1	101	150	143	118	67	96	0.698176
ENSG0000(	GPR1	133	66	92	81	16	14	1.82696
ENSG0000(	WARS	6429	4837	4125	5198	1632	3329	74.28784
ENSG0000(	AAGAB	1279	1633	1340	1421	1010	1387	15.12467
ENSG0000(	RPL15	67873	75076	75743	105193	104708	113281	1140.405
ENSG0000(	NRBP1	4193	4546	4645	4706	3550	3546	50.99635
Homo_sapi	Homo_sapi	67	148	88	146	190	313	0.538855
ENSG0000(	RPL10A	21679	24575	22627	30023	38342	41685	952.7157
ENSG0000(	TENT5A	976	1056	1065	1455	2058	1696	5.470122
ENSG0000(	BCL2L12	1229	1672	1349	1437	1042	1364	35.15929
Homo_sapi	Homo_sapi	39	49	45	140	63	98	0.959436
Homo_sapi	Homo_sapi	327	648	955	801	1569	1863	7.449625
ENSG0000(	ST3GAL3	365	660	521	797	853	832	5.326983
ENSG0000(	PRKD2	849	1122	1015	1070	685	778	9.094377
Homo_sapi	Homo_sapi	53	71	55	106	107	130	0.615673
ENSG0000(	DRAM1	573	879	699	699	525	667	5.261859
ENSG0000(	CCDC28A	235	342	429	199	294	212	5.9161
ENSG0000(	C12orf75	2894	3112	2879	3185	2155	2325	67.06886
ENSG0000(	TBC1D9	239	374	334	414	615	629	1.417313
ENSG0000(	RBBP4	5131	6872	6699	8081	9729	11900	21.66603
Homo_sapi	Homo_sapi	255	255	223	193	175	225	4.672885
ENSG0000(	TMPRSS4	41	85	87	35	22	57	0.283754
ENSG0000(	MMAB	1140	1354	1231	2503	1569	1949	17.50573
ENSG0000(	GSTZ1	361	360	351	860	451	643	9.191453
ENSG0000(	LHPP	324	426	413	550	566	840	6.669582
ENSG0000(	TUSC3	446	821	753	622	476	541	8.695318
ENSG0000(	PLEKHB1	29	32	23	63	46	107	0.415145
ENSG0000(	SCAND1	1612	2136	1825	3049	2442	2697	40.57683
ENSG0000(	PARD6A	267	304	304	758	377	488	6.645208
ENSG0000(	EIF2S3	13043	18193	17281	19971	29972	30801	143.6581
ENSG0000(	VPS33B	630	818	709	772	372	532	7.255054
ENSG0000(	DDAH1	533	769	652	1017	826	1214	4.267944
ENSG0000(	RBKS	282	425	340	570	478	575	4.650387
ENSG0000(	RPL35A	11602	13730	12207	18845	17987	17971	515.2687
ENSG0000(	SLC25A24	2860	3647	3175	3335	2513	3221	21.34759
ENSG0000(	PTDSS2	5764	6682	5952	7256	11968	13191	74.41327
ENSG0000(	ILVBL	3759	4771	4575	6094	6766	6643	55.30372
ENSG0000(	CHD1L	2292	2739	2023	3867	3243	3744	24.34364
Homo_sapi	Homo_sapi	17	36	31	54	83	64	0.436779
ENSG0000(	LAMC3	937	1029	814	2075	1264	1440	4.860501
ENSG0000(	IFT140	1040	1636	1293	1907	1834	2226	6.406927
ENSG0000(	DLX3	211	224	259	241	109	119	2.564697
ENSG0000(	SULT1A1	675	771	682	1339	1013	1029	14.44132
ENSG0000(	CUX1	2747	3735	3352	4741	4419	4826	17.53495
Homo_sapi	Homo_sapi	168	210	155	258	285	439	4.017443
ENSG0000(	GPR157	854	1023	1003	941	842	875	5.095273
Homo_sapi	Homo_sapi	356	496	391	563	660	808	1.872522
ENSG0000(	CLDN1	884	1328	1131	328	812	996	7.990664
Homo_sapi	Homo_sapi	152	166	176	246	345	294	2.204565
ENSG0000(	RPL13	66580	81112	78723	107637	123479	111541	522.8871

ENSG0000(	C19orf48	2981	2842	2671	4650	4240	4509	60.32992
Homo_sapi	Homo_sapi	64	109	121	120	245	279	0.43324
ENSG0000(	SCD5	7	23	28	47	62	55	0.076143
ENSG0000(	DDI2	961	1331	1244	1416	2556	2294	2.84428
ENSG0000(	VASH1	1546	2032	1866	1787	1647	1717	8.645741
ENSG0000(	KRBOX4	561	704	499	844	1039	1008	7.750932
ENSG0000(	AC013394.	602	815	797	961	1271	1378	23.42308
ENSG0000(	RDH10	156	217	179	99	135	171	1.256548
ENSG0000(	IL1A	153	127	91	16	90	58	2.260197
ENSG0000(	ENDOD1	321	571	502	421	287	417	2.148441
ENSG0000(	KRT16	229	223	201	180	179	137	4.923499
Homo_sapi	Homo_sapi	20	39	23	53	64	73	0.353888
ENSG0000(	IRS1	460	677	598	737	1385	1029	1.491
ENSG0000(	SHISAL1	300	375	286	329	193	235	1.397939
ENSG0000(	ST3GAL4	2085	2942	2788	3597	3927	4120	35.98457
ENSG0000(	ANXA10	76	52	48	40	4	3	1.681121
ENSG0000(	PKP4	899	1332	1241	1595	1734	1966	6.135075
ENSG0000(	GJB3	3167	2837	2340	3098	1343	1218	45.74445
ENSG0000(	SLC35C1	1670	2251	1611	2052	810	1141	13.82399
ENSG0000(	STX1A	342	352	404	294	320	187	6.338867
ENSG0000(	PRDX1	31643	40056	35952	39623	19280	28675	1030.368
ENSG0000(	CDH13	1099	1540	1324	1423	904	1103	4.370392
ENSG0000(	DENND1B	320	355	321	253	231	343	1.435639
Homo_sapi	Homo_sapi	920	993	945	1116	2155	2013	46.89916
ENSG0000(	EDN2	125	149	141	233	205	254	3.164346
ENSG0000(	TMEM256	1089	1483	1210	1903	1843	1820	70.4113
Homo_sapi	Homo_sapi	278	247	212	300	497	770	0.742383
ENSG0000(	PM20D2	355	458	413	570	636	708	2.367211
ENSG0000(	TMEM206	1010	1121	1213	1130	863	1035	12.84836
ENSG0000(	TTYH3	3247	5661	5429	6420	10733	7975	21.9994
ENSG0000(	ITGB2	323	308	420	398	1060	781	4.166531
ENSG0000(	HES4	698	1023	826	1102	1800	1463	23.54361
ENSG0000(	PDIA6	13649	15901	15957	16403	12123	12633	183.6347
ENSG0000(	FBXO38	820	1039	897	859	659	961	6.191044
ENSG0000(	LINS1	263	370	328	264	200	327	2.065189
ENSG0000(	PYGO1	341	498	440	594	666	743	1.591053
Homo_sapi	Homo_sapi	5221	5934	6268	10147	8884	7911	36.67371
ENSG0000(	LIFR	2012	2777	2536	2876	4336	5497	7.699403
ENSG0000(	GPRC5B	56	118	117	218	174	166	0.333028
ENSG0000(	TRAPPC2	397	459	422	584	789	739	4.91973
Homo_sapi	Homo_sapi	347	642	588	517	1237	1580	3.731416
ENSG0000(	ZCCHC24	374	552	495	354	427	414	2.381455
Homo_sapi	Homo_sapi	128	137	169	124	475	488	0.702113
ENSG0000(	CMTM6	1027	1304	1142	964	1043	1159	8.364806
ENSG0000(	WDR31	72	137	108	181	154	268	0.456609
ENSG0000(	CTNNBL1	2896	3417	3159	3277	2670	3088	48.43979
ENSG0000(	C5	35	22	22	56	47	134	0.200484
ENSG0000(	DUSP3	1448	1765	1385	2451	2072	2348	11.9618
ENSG0000(	POLA1	1840	2580	2431	2989	3433	4310	10.56438
Homo_sapi	Homo_sapi	86	92	101	80	332	315	2.582917

ENSG0000(	RTL8C	953	1404	1361	1728	2170	1970	25.39072
Homo_sapi	Homo_sapi	346	453	354	486	656	913	1.246486
ENSG0000(	ORAI3	224	393	397	342	1214	728	4.256122
ENSG0000(	UGGT1	5578	5898	5464	5953	4498	4944	18.50002
ENSG0000(	LOXL4	178	239	166	440	258	337	1.532728
ENSG0000(	PDE7B	71	56	66	105	142	151	0.410128
ENSG0000(	RNF13	780	926	904	802	699	906	9.854757
ENSG0000(	CARD6	1111	1797	1370	1263	1292	1167	8.275105
ENSG0000(	STC2	6919	7302	5393	2193	5744	5120	42.54155
ENSG0000(	FOLR1	109130	112023	114524	156203	188040	181408	3405.739
ENSG0000(	GATAD1	1116	1219	994	1673	1667	1805	8.996975
ENSG0000(	B3GNT3	1211	1622	1440	1584	923	916	17.33951
ENSG0000(	LOXL2	7336	7771	7273	10419	15322	11751	65.72429
ENSG0000(	ABCC8	106	86	71	40	66	56	0.756336
ENSG0000(	JUND	1977	2152	1787	2500	3968	3719	33.46971
ENSG0000(	RPL18A	40353	44805	44874	62956	64498	64431	1898.751
ENSG0000(	GSTT2B	338	314	275	762	439	504	10.42909
ENSG0000(	ERGIC1	24467	30217	27693	37664	36707	44414	268.9731
ENSG0000(	GABPB1	1310	1618	1443	1460	1247	1465	16.57286
ENSG0000(	SERPINB8	434	515	403	469	295	353	4.215591
ENSG0000(	RND1	28	41	39	49	116	95	0.521126
ENSG0000(	DOK3	45	61	66	48	25	16	0.647001
ENSG0000(	TMEM56	329	597	556	659	934	953	1.517416
ENSG0000(	FAM149B1	929	1286	1020	1522	1467	1861	5.954559
ENSG0000(	PDE2A	2719	4193	3660	6341	6248	4490	24.47282
ENSG0000(	HIST2H2BF	115	139	122	103	93	98	1.962724
ENSG0000(	PARP9	764	1119	879	952	597	828	6.60491
Homo_sapi	Homo_sapi	323	390	438	445	913	834	2.894659
ENSG0000(	NAV1	324	448	496	404	251	377	1.449463
ENSG0000(	SIRT6	786	1107	1001	950	848	855	15.82837
ENSG0000(	LNPEP	842	628	642	469	600	576	2.076445
Homo_sapi	Homo_sapi	467	696	649	681	1027	1946	3.534012
ENSG0000(	NDUFAF1	806	1182	917	1042	548	764	20.2396
ENSG0000(	NPIPA7	75	146	85	165	193	207	1.7709
ENSG0000(	MTMR6	956	1226	1170	1055	957	1149	5.31033
ENSG0000(	AMDHD2	625	822	771	1315	1006	1060	8.857088
ENSG0000(	RARG	5131	6009	5599	6304	3509	4233	61.10388
ENSG0000(	FBLN5	181	254	203	370	298	346	2.414162
ENSG0000(	ST3GAL1	1062	1745	1371	1413	1055	1226	5.24516
Homo_sapi	Homo_sapi	16	45	20	60	57	80	0.136027
Homo_sapi	Homo_sapi	319	343	347	443	603	678	2.78817
ENSG0000(	KCNQ1	1733	2122	1990	2706	2940	2975	25.48656
ENSG0000(	PLIN4	210	309	344	829	382	452	1.022048
Homo_sapi	Homo_sapi	23	30	31	83	55	53	1.303993
ENSG0000(	MCCC1	267	420	385	512	549	636	3.286573
ENSG0000(	ZNF410	1758	1813	1682	1861	1307	1467	23.65865
Homo_sapi	Homo_sapi	48	82	57	87	147	150	1.06504
Homo_sapi	Homo_sapi	170	279	238	264	465	595	2.217052
ENSG0000(	EIF2S2	6360	8299	7996	7072	5736	8237	75.34167
ENSG0000(	HSP90AA1	21574	36203	32332	28738	23012	29200	201.2376

ENSG0000(	NOL8	643	1013	898	797	692	814	4.651889
ENSG0000(	MTA1	1755	2393	2067	3158	2840	2919	21.00893
ENSG0000(	APOL1	1263	1684	1444	1615	904	681	13.80715
ENSG0000(	MSN	15481	21787	19494	21443	11582	13853	123.5027
ENSG0000(	RPL37A	17561	20772	19110	29597	27950	26460	163.1071
ENSG0000(	SELENOK	2567	2361	2355	2029	2100	2322	85.57663
Homo_sapi	Homo_sapi	116	151	151	97	110	118	1.064787
ENSG0000(	GPR160	78	112	93	109	263	230	1.195696
ENSG0000(	MGME1	621	713	736	992	954	1235	8.554333
ENSG0000(	RPL9	11354	15760	16205	20028	22648	22511	353.6111
ENSG0000(	TMEM91	210	365	352	303	1130	655	5.773784
ENSG0000(	GCH1	289	339	348	178	268	305	3.084924
ENSG0000(	CLCNKA	13	39	14	46	74	57	0.147475
ENSG0000(	NECAB3	1386	1690	1416	2743	1969	2183	22.58959
ENSG0000(	PRSS16	97	235	243	62	96	173	1.12197
ENSG0000(	SNAPC1	372	399	432	316	335	388	4.400379
ENSG0000(	FAM86B1	90	161	164	241	205	287	1.178141
ENSG0000(	LMNB1	2903	3539	3220	4787	4022	5094	31.64168
ENSG0000(	FANCC	579	837	723	967	1036	1282	5.159138
ENSG0000(	TRIM2	153	158	156	93	96	157	0.951425
Homo_sapi	Homo_sapi	347	408	358	601	475	685	2.816185
ENSG0000(	AGL	1071	1677	1713	1276	1178	1466	4.568336
ENSG0000(	JPT2	6519	9764	9007	11465	12591	13432	58.3176
ENSG0000(	AP5M1	429	681	658	544	417	562	1.210272
ENSG0000(	YIPF5	2181	2718	2398	2451	2090	2513	21.9345
ENSG0000(	PSME4	2619	3795	3773	2961	3032	3323	11.60516
ENSG0000(	B3GNT2	607	723	623	576	603	566	6.905887
ENSG0000(	ZNF843	37	52	69	28	15	42	0.358869
ENSG0000(	ID2	95	110	110	105	39	53	2.176108
ENSG0000(	USP36	2304	3112	2628	3482	4607	4423	16.32461
ENSG0000(	FAM57A	822	887	785	1630	1099	1274	10.93191
ENSG0000(	CHAC1	1463	658	520	249	542	724	29.56104
ENSG0000(	CLTC	9349	13159	12331	10897	10249	12119	36.59459
ENSG0000(	ZNF280D	566	756	797	1011	1001	1228	4.510403
ENSG0000(	TSPYL4	752	998	953	1094	1494	2035	5.660061
ENSG0000(	UPP1	2263	2359	2264	2417	1871	1893	57.43355
Homo_sapi	Homo_sapi	52	58	42	78	104	137	1.407399
ENSG0000(	ADM	1308	863	868	1154	3459	2220	23.20106
ENSG0000(	GOLGA8B	804	901	607	790	1894	2037	5.837743
ENSG0000(	RBMS1	1195	1615	1489	1228	1252	1500	9.212595
Homo_sapi	Homo_sapi	51	65	33	30	10	34	0.529154
ENSG0000(	GABPB2	305	453	400	540	551	782	1.545946
ENSG0000(	STOX1	23	31	54	69	98	84	0.192899
ENSG0000(	ZNF468	464	522	513	1184	718	697	5.24652
ENSG0000(	ABCC10	642	637	588	682	385	400	4.005453
ENSG0000(	TRIM16	1540	2288	1970	2177	1172	1309	19.04236
ENSG0000(	RCOR2	65	127	93	164	205	158	0.782735
ENSG0000(	PDLIM1	1198	1523	1618	1363	1281	1386	25.95787
ENSG0000(	PLCXD1	2158	2995	2485	3914	3800	3499	13.30955
ENSG0000(	NARS	3981	5233	4564	4315	3920	4985	41.19883

ENSG0000(	CASP2	1033	1383	1138	1934	1495	1790	7.746189
ENSG0000(	GMPR2	1317	1870	1701	2236	2276	2631	22.23736
ENSG0000(	SLC22A31	874	949	1017	1061	591	588	14.76415
ENSG0000(	CEBPD	392	582	434	674	666	835	5.660022
ENSG0000(	SLC37A3	868	1133	1127	690	930	1014	8.25528
ENSG0000(	ALAS1	6111	7420	6206	7298	4417	5623	87.856
ENSG0000(	CLCN5	562	736	685	508	542	697	2.019412
ENSG0000(	MEST	735	815	822	998	1318	1640	9.544401
ENSG0000(	GSN	1979	2267	2315	2356	1762	1760	26.49632
ENSG0000(	SNAI1	294	386	360	345	243	149	5.473138
ENSG0000(	SPINT2	343	324	271	329	177	197	7.118319
ENSG0000(	IRS2	2062	2444	2133	2143	7331	4456	7.970272
ENSG0000(	ZFP62	542	734	663	760	1107	1569	5.313004
ENSG0000(	SEC14L2	278	263	208	417	472	415	2.434258
ENSG0000(	RTL10	1591	2099	1758	2751	2203	3502	7.663746
ENSG0000(	MCM3	6989	9229	8579	11438	10659	13551	72.50974
ENSG0000(	RPLP0	92519	98514	100460	140400	143378	147126	2708.409
ENSG0000(	TNXB	705	1081	934	1047	335	384	2.672489
Homo_sapi	Homo_sapi	13567	14987	16901	24635	23539	21089	169.2483
ENSG0000(	MT4	117	118	109	3	67	49	12.45011
ENSG0000(	LEPROTL1	1261	1666	1544	1531	1144	1508	12.9539
Homo_sapi	Homo_sapi	78	85	112	52	43	87	1.013699
ENSG0000(	MTUS2	40	111	61	57	28	29	0.694369
ENSG0000(	DR1	3373	4483	3998	3577	3770	3888	10.45615
Homo_sapi	Homo_sapi	184	296	329	252	776	723	2.638784
ENSG0000(	SPSB1	905	1122	890	959	851	707	9.545273
ENSG0000(	ACSF2	704	851	804	988	1291	1559	10.10746
ENSG0000(	FUT11	731	720	701	675	2533	1536	10.76127
ENSG0000(	LOX	110	231	203	162	1444	889	0.676562
ENSG0000(	FGF13	69	100	112	193	147	161	0.285289
ENSG0000(	FYN	134	235	279	76	150	184	1.174113
ENSG0000(	PCBP2	20473	27830	26073	31924	41121	40499	425.6516
ENSG0000(	MYO15B	1926	2442	2419	3246	3870	3270	12.31993
ENSG0000(	ING4	247	472	389	407	807	891	6.043222
ENSG0000(	FAM210B	307	518	438	547	714	826	3.20125
ENSG0000(	IFT20	607	882	872	643	657	797	19.70438
ENSG0000(	C9orf78	3061	3956	3616	3930	2645	3090	53.65606
ENSG0000(	ENOSF1	1480	1612	1175	2462	1807	2699	16.19686
ENSG0000(	NAT14	298	484	361	586	584	591	6.166931
ENSG0000(	RAB9A	1519	1794	1925	2359	2612	2974	26.52619
ENSG0000(	ZNF280A	32	43	54	19	27	27	0.464234
ENSG0000(	ZNF618	290	340	297	301	248	261	1.061932
ENSG0000(	TIGD2	338	439	448	534	706	752	3.309574
ENSG0000(	ZBED6CL	265	257	239	383	443	440	2.841271
ENSG0000(	AKR7A2	1816	2327	2288	4032	2788	3050	42.17585
ENSG0000(	VAMP2	583	770	854	930	1466	1338	9.148612
ENSG0000(	PFKP	11629	14318	12846	19399	19974	17719	139.0839
Homo_sapi	Homo_sapi	98	195	211	241	455	300	2.850988
ENSG0000(	TREX2	99	96	36	7	0	43	1.396638
ENSG0000(	BMPR1A	608	822	729	524	634	723	1.863554

Homo_sapi	Homo_sapi	125	103	98	103	37	67	2.460121
ENSG0000(	TM2D2	952	1000	991	1046	649	875	9.335012
ENSG0000(	SLC1A5	6995	9031	7848	8807	6329	6728	77.84924
ENSG0000(	TMEM115	1712	2075	1841	2056	1468	1589	24.50713
ENSG0000(	ALDOC	1818	2632	3234	3396	5522	4387	32.13164
ENSG0000(	SLC45A4	983	1341	1074	1335	1939	2510	5.066511
Homo_sapi	Homo_sapi	67	55	67	33	29	56	2.9252
ENSG0000(	PPM1J	178	218	235	138	198	131	2.810473
ENSG0000(	PLAU	541	664	464	591	357	414	7.243439
ENSG0000(	KATNAL2	48	63	48	83	91	153	0.910601
ENSG0000(	HELLS	671	693	652	906	1029	1372	6.377825
ENSG0000(	RAVER1	1301	2235	1841	3125	2645	2477	11.82348
ENSG0000(	IFT81	370	564	483	620	715	974	3.820099
ENSG0000(	CXXC5	3500	3907	3324	7403	5296	4748	52.83175
ENSG0000(	CALHM3	104	107	137	118	25	28	1.964274
ENSG0000(	MAGEA3	1347	1504	1274	1485	965	1196	25.12513
ENSG0000(	MAPRE3	303	500	523	435	247	365	5.105655
ENSG0000(	SRD5A3	349	499	377	696	502	727	2.613653
ENSG0000(	C11orf98	994	1316	1214	1328	771	795	47.99266
ENSG0000(	RPL8	84168	92747	90870	141601	127744	124240	2609.928
ENSG0000(	DUSP2	236	289	246	427	391	384	4.387589
ENSG0000(	MATR3	5302	2501	2718	904	2372	3100	44.5436
ENSG0000(	KLF10	1765	2447	2099	2102	4967	4783	17.91882
ENSG0000(	PTPN9	822	959	970	1240	1479	1498	3.769621
ENSG0000(	PIGG	674	905	812	856	619	667	7.187681
ENSG0000(	GNPAT	1551	2347	1923	3011	2368	3180	18.50387
ENSG0000(	AGAP1	1540	2044	1834	2484	2793	2695	4.739383
ENSG0000(	SPC24	943	1370	1331	1598	1856	2152	13.77047
ENSG0000(	CEP55	1259	1855	2083	1262	1518	1623	15.03559
ENSG0000(	SLC9A5	157	235	233	180	163	176	1.41411
ENSG0000(	GATA1	11	39	40	16	135	191	0.220983
ENSG0000(	SLC2A11	129	148	129	207	240	235	1.96428
ENSG0000(	TM7SF2	498	625	532	1276	671	868	9.39426
ENSG0000(	DMAP1	1055	1271	1144	1995	1458	1727	21.63363
ENSG0000(	KLHL22	393	413	409	861	515	652	4.956287
ENSG0000(	SYCE2	253	209	205	346	324	618	6.611319
ENSG0000(	CACUL1	1136	1532	1389	1129	1244	1366	3.158819
ENSG0000(	LIF	1661	1332	1148	1170	1210	902	13.3049
ENSG0000(	TMEM69	1218	1639	1624	2034	2082	2523	15.71158
ENSG0000(	MRPS27	4789	6841	5952	10154	7176	8647	56.51374
ENSG0000(	EFR3B	44	142	96	140	203	239	0.184546
ENSG0000(	IL15RA	332	457	365	398	270	331	6.493109
ENSG0000(	ECI1	2785	3259	3387	4990	3981	4749	85.08201
ENSG0000(	PKDCC	139	181	190	379	181	410	1.74048
ENSG0000(	SMIM11A	285	330	244	523	511	403	10.03908
ENSG0000(	SLC25A27	91	137	165	130	328	409	1.092427
ENSG0000(	TBC1D2	1614	2206	1978	3125	2972	2560	15.50996
ENSG0000(	ANGPTL2	77	117	73	86	30	21	0.652388
ENSG0000(	UPK3B	94	62	56	273	90	159	1.422358
ENSG0000(	LRRC73	15	22	39	51	72	63	0.220751

ENSG0000(	IL18	1617	1982	1882	3538	1989	3424	48.56639
ENSG0000(	RAB2A	2790	3771	3316	3434	2710	3383	23.43467
ENSG0000(	C19orf71	479	702	512	730	869	1176	22.33954
ENSG0000(	PNO1	1609	1910	1520	1874	1102	1335	22.14062
Homo_sapi	Homo_sapi	127	189	160	166	83	84	1.205624
ENSG0000(	8-Mar	1010	1305	1171	1136	1048	1167	6.205861
ENSG0000(	RNF145	3552	3710	3470	2833	3394	3396	30.95311
ENSG0000(	QARS	6717	8411	8083	10009	12387	12558	90.66509
ENSG0000(	ADAMTS13	69	63	40	89	131	151	0.477979
ENSG0000(	USP13	459	514	503	861	630	778	1.735223
ENSG0000(	EIF4B	24042	42937	41155	41076	74948	72957	191.9613
ENSG0000(	VWA7	204	322	277	260	207	176	2.222653
ENSG0000(	SLC45A3	827	978	950	965	772	810	7.782797
ENSG0000(	MARS	6689	7966	7107	7863	3317	6247	72.66624
ENSG0000(	UPF3A	1688	2199	1900	2576	2743	3099	23.36804
ENSG0000(	SCYL2	831	1335	1242	899	971	1127	4.865996
ENSG0000(	ANTXR1	2260	2724	2180	4145	2923	3781	14.8779
ENSG0000(	CLEC2B	165	295	394	425	648	520	3.045628
ENSG0000(	KCNH5	41	31	48	66	89	110	0.11414
Homo_sapi	Homo_sapi	47	46	37	37	16	11	2.183616
ENSG0000(	EXTL2	960	1402	1304	1152	830	1286	9.03019
ENSG0000(	GLIPR1	108	117	120	104	47	93	0.591459
ENSG0000(	ATG4A	478	453	438	468	254	395	6.486684
ENSG0000(	TMEM184A	1245	1256	1103	1958	1846	1781	6.706549
ENSG0000(	DDX41	3566	4144	3857	5779	5572	5401	45.70169
Homo_sapi	Homo_sapi	36	55	38	62	116	97	0.994624
ENSG0000(	RAB6B	127	155	140	99	106	132	0.721285
Homo_sapi	Homo_sapi	124	172	126	220	283	224	4.603394
Homo_sapi	Homo_sapi	310	404	348	296	65	291	4.992583
Homo_sapi	Homo_sapi	1690	2579	2321	2999	3785	3330	15.22542
ENSG0000(	GPRIN2	325	437	405	617	578	561	5.195995
ENSG0000(	TOMM20	6954	8305	7945	9464	13436	13927	64.59622
ENSG0000(	SRGAP3	40	35	33	45	85	145	0.142595
ENSG0000(	SARAF	4262	5701	5454	4504	4936	4999	66.55365
ENSG0000(	LHX6	275	320	246	380	434	641	3.21648
ENSG0000(	AK4	605	818	668	727	1763	1445	2.763967
ENSG0000(	BCKDK	2345	2394	2310	4493	3321	3290	36.77482
ENSG0000(	CMTM1	47	60	66	21	41	41	1.573192
ENSG0000(	ATF6	3253	4648	4037	3916	3219	4255	13.65161
ENSG0000(	HMGB3	1688	2489	2067	2118	1707	2098	16.4294
ENSG0000(	MZT1	1724	2367	2216	2462	4099	3919	23.58897
ENSG0000(	IFIH1	351	392	407	227	360	323	3.228138
ENSG0000(	SYTL2	53	67	62	57	15	27	0.342005
ENSG0000(	PABPN1	3377	3916	3444	5352	5227	5064	48.69321
Homo_sapi	Homo_sapi	134	121	96	100	447	328	3.018577
ENSG0000(	NR4A3	103	162	190	185	328	362	0.564101
Homo_sapi	Homo_sapi	63	77	59	45	30	58	1.527182
ENSG0000(	EDN1	236	258	163	314	442	428	3.650754
ENSG0000(	RAB40B	473	695	613	834	846	954	4.099039
ENSG0000(	EGR1	194	59	53	131	3401	1306	1.939299

ENSG0000(	TPI1	26696	32338	33986	45357	51163	43521	681.4586
ENSG0000(	SDHAF4	196	298	264	337	415	482	5.155579
ENSG0000(	MED9	750	892	687	791	570	730	10.72023
ENSG0000(	HCN3	221	282	187	287	520	458	1.869468
ENSG0000(	C16orf91	948	1254	1038	1040	989	1047	30.46901
ENSG0000(	MAFB	462	422	405	450	307	261	4.280226
ENSG0000(	TESC	27	21	26	54	70	49	0.796771
ENSG0000(	ZFAND2A	1500	1257	1043	1072	1093	994	53.70834
ENSG0000(	DECR1	740	944	778	1229	1032	1384	19.37696
ENSG0000(	IGBP1	1516	2573	2514	2174	5159	5116	27.32666
ENSG0000(	AFMID	669	766	794	1092	1079	1124	21.05219
Homo_sapi	Homo_sapi	142	179	234	152	1357	912	1.967307
ENSG0000(	BCO2	237	366	393	399	544	937	2.65328
ENSG0000(	MYH10	41	80	76	38	36	53	0.166181
ENSG0000(	ELMO2	1381	1963	1886	1787	1400	1722	12.40576
ENSG0000(	RPL32	29563	33404	31711	42652	45927	49527	455.4324
ENSG0000(	CTDSPL	795	959	806	1221	1265	1293	6.087815
ENSG0000(	VTN	479	321	315	298	243	327	7.00861
Homo_sapi	Homo_sapi	190	281	236	303	377	533	0.922278
ENSG0000(	PHLPP2	570	759	666	623	526	703	2.728371
ENSG0000(	RPL4	55606	68616	62094	76975	97013	112465	631.9279
Homo_sapi	Homo_sapi	30	39	24	32	82	144	0.219157
ENSG0000(	CARD16	2575	2963	2904	4213	3440	4929	129.0979
ENSG0000(	PFKL	4585	6946	6775	9322	8498	8825	49.53275
ENSG0000(	DZIP3	97	78	68	139	136	204	0.567414
ENSG0000(	PNRC2	2229	2507	2281	2702	4349	4803	30.53831
ENSG0000(	STPG1	652	863	815	778	488	779	7.529518
Homo_sapi	Homo_sapi	34	26	34	50	85	81	0.793295
ENSG0000(	CCZ1	2506	3132	2669	3023	2176	2600	43.68516
ENSG0000(	NIPSNAP3A	540	708	711	1093	819	1018	10.39206
ENSG0000(	CD44	12543	14929	14131	15737	10092	8326	133.8123
ENSG0000(	ZNF589	626	744	560	866	995	1214	5.841304
ENSG0000(	PI4K2A	1900	1851	1734	1789	1615	1550	14.26778
ENSG0000(	BTBD2	1965	2923	2559	3956	3569	3357	24.67785
ENSG0000(	TONSL	1325	2084	2079	3067	2609	2608	9.322564
ENSG0000(	MID1	117	128	161	214	197	311	0.56407
ENSG0000(	LENG9	271	360	266	418	520	500	4.448236
ENSG0000(	GPAM	630	765	681	558	574	744	3.114481
Homo_sapi	Homo_sapi	30	55	43	71	99	86	1.160931
ENSG0000(	RMC1	667	868	738	825	587	598	9.693725
ENSG0000(	PMM1	1071	1821	1944	2166	3155	2694	25.96716
ENSG0000(	ADAM8	44	59	74	32	41	41	0.413245
ENSG0000(	RAB43	799	1635	1671	1357	917	897	6.116007
ENSG0000(	LARP6	100	101	119	70	94	53	1.593132
ENSG0000(	GDF15	2620	2071	2040	1783	2062	1776	68.83485
ENSG0000(	BLVRA	2707	3251	2849	3104	1933	2913	79.26953
ENSG0000(	SLC41A2	138	211	189	113	151	157	2.112592
ENSG0000(	RNF157	326	423	415	612	479	733	2.169089
Homo_sapi	Homo_sapi	251	234	203	260	454	663	1.630192
ENSG0000(	ACAP1	55	202	142	51	80	98	0.699504

ENSG0000(	CNTLN	294	326	279	424	485	519	1.740229
Homo_sapi	Homo_sapi	435	574	455	557	831	1254	1.81673
ENSG0000(	POLA2	1902	2957	2744	5004	2830	4373	17.24421
ENSG0000(	PCIF1	1164	1544	1458	1930	1923	2093	13.39778
Homo_sapi	Homo_sapi	248	338	229	384	391	622	0.901948
ENSG0000(	EIF3E	5326	7211	7508	8132	10928	12863	113.0837
ENSG0000(	CTU1	76	61	75	163	113	130	1.134001
ENSG0000(	C16orf74	611	888	813	1111	1164	1134	22.05371
ENSG0000(	GINS2	1594	2004	1758	3037	2215	2613	19.45434
ENSG0000(	PHF10	1506	2448	2050	2118	4202	4273	19.05316
ENSG0000(	TCTN2	585	791	807	1194	1061	1015	7.110841
ENSG0000(	NR1H3	1067	1767	1577	2436	1917	2219	21.26964
ENSG0000(	PHACTR2	216	294	262	185	59	251	0.911304
ENSG0000(	GCHFR	2167	2626	2738	3758	3358	3659	87.94033
ENSG0000(	ACSL5	46	85	40	45	3	14	0.431033
ENSG0000(	CDCA7L	3927	6153	5575	6604	8809	8802	44.84055
ENSG0000(	LRPPRC	5124	6146	5480	6941	9352	9692	25.88294
ENSG0000(	YWHAQ	11461	14807	13002	14508	10064	12429	163.4952
ENSG0000(	SLC11A2	4246	4447	3964	3980	3648	4319	35.47761
ENSG0000(	ARF4	9875	13928	12825	12691	9927	12632	189.7258
ENSG0000(	C8orf82	270	338	262	630	472	376	4.373238
ENSG0000(	N4BP2	173	132	123	117	95	112	0.597075
ENSG0000(	DCUN1D5	2629	3309	2990	3377	2002	2551	64.49682
Homo_sapi	Homo_sapi	485	584	467	506	1332	1145	3.514829
ENSG0000(	ALG6	310	435	426	578	514	707	3.655091
ENSG0000(	HIST1H2BD	948	1456	1009	1275	701	811	41.41779
ENSG0000(	PSEN1	1450	1845	1655	1457	1460	1779	10.85402
ENSG0000(	GDF11	710	1063	947	1542	1159	1331	2.703835
ENSG0000(	SOAT1	1735	2098	2103	2854	2464	3555	7.98041
Homo_sapi	Homo_sapi	80	56	49	28	31	53	0.987648
ENSG0000(	BCKDHB	327	416	405	513	649	637	4.29813
ENSG0000(	CFD	4447	5891	6254	6256	11705	10398	116.5078
ENSG0000(	TRERF1	768	1032	934	953	790	804	3.398803
ENSG0000(	RGS2	932	1088	990	1261	1786	1740	21.63638
ENSG0000(	SIAE	1127	1302	1194	1646	1691	1998	6.351717
Homo_sapi	Homo_sapi	27	38	34	53	73	79	0.650368
ENSG0000(	DPP8	646	907	691	636	587	805	4.388713
ENSG0000(	VGF	40	69	91	45	40	43	0.484719
ENSG0000(	RCC2	8303	10737	9058	9991	7256	9590	63.9314
ENSG0000(	ZNF395	1801	1619	1436	1779	3759	3543	13.34526
ENSG0000(	PTGES3L-A	115	54	87	50	24	74	1.707985
ENSG0000(	NR4A2	296	318	295	239	1138	704	2.731772
ENSG0000(	UBQLN1	5881	7022	6099	6128	5627	6786	45.25923
ENSG0000(	VTI1B	1922	2527	2218	2387	1871	2178	19.02256
ENSG0000(	NPHP1	194	207	203	375	277	321	5.768833
ENSG0000(	ETV5	1715	2189	2042	1183	1854	1908	13.60843
ENSG0000(	ACOT2	531	629	488	869	780	834	9.796405
ENSG0000(	SMAD6	440	533	353	470	288	314	3.623405
ENSG0000(	FAM13A	922	835	925	749	2762	2226	6.933386
ENSG0000(	PTGR2	239	420	283	368	580	720	2.20181

ENSG0000(	RPS3A	40658	47211	46769	57733	65321	78237	1390.529
ENSG0000(	RHPN1	295	510	492	819	595	654	2.517762
ENSG0000(	NXPE3	367	664	695	513	428	495	1.992566
ENSG0000(	ZFP30	306	430	296	312	273	319	1.654914
ENSG0000(	CNIH4	3334	4501	4437	4054	3533	4289	28.72594
ENSG0000(	KIF21B	1667	2253	2039	1377	1951	1817	5.736792
ENSG0000(	ORC1	1445	1664	1718	2417	2389	2267	14.4379
ENSG0000(	MAFK	7979	9091	8071	8196	21196	17741	77.70579
ENSG0000(	FGFBP3	39	66	57	107	83	109	0.474689
ENSG0000(	GAPDH	99099	112814	117981	158043	178042	155823	1776.63
ENSG0000(	FAM83A	7626	9609	8147	7654	8303	8112	53.08521
ENSG0000(	LIPH	431	458	369	298	310	448	3.573883
ENSG0000(	EMP1	2233	2949	2695	2969	1882	2260	11.92713
ENSG0000(	TMEM52	197	315	366	417	523	527	6.78389
ENSG0000(	MGAT5B	878	969	888	2300	1012	1520	6.552333
ENSG0000(	RASA2	368	474	411	492	867	787	2.060496
ENSG0000(	TSPAN17	2707	3134	3057	3484	1802	2181	35.20711
ENSG0000(	CNTF	55	70	44	26	16	53	0.909201
ENSG0000(	CKMT1B	35	54	39	29	19	31	0.59025
ENSG0000(	HARBI1	231	281	209	238	141	211	3.791394
ENSG0000(	NETO2	734	1020	773	827	675	851	4.949854
ENSG0000(	1-Sep	50	35	55	78	113	105	0.984168
ENSG0000(	ATG13	3472	4205	3664	4192	3036	3433	29.56036
Homo_sapi	Homo_sapi	127	301	244	307	466	426	3.157252
ENSG0000(	MB	436	459	383	448	297	354	16.8598
ENSG0000(	DGKQ	359	615	664	420	475	449	2.445205
ENSG0000(	RCCD1	1904	3235	2822	3648	3991	4223	22.86548
ENSG0000(	FAM172A	380	640	649	722	924	1079	2.974263
ENSG0000(	TMEM143	271	339	290	520	370	507	3.421525
ENSG0000(	LRP11	1607	2834	2508	1962	2109	2033	15.51669
ENSG0000(	TSPAN31	970	1230	1052	1493	1427	1838	14.11845
ENSG0000(	TCTN1	582	873	793	1392	921	1137	8.252862
ENSG0000(	WASHC4	1129	1696	1435	947	1215	1493	6.737629
ENSG0000(	PGK1	58389	71226	72057	74881	144265	124518	375.9478
ENSG0000(	TRIM73	65	64	55	94	132	126	1.520366
Homo_sapi	Homo_sapi	117	202	114	223	252	264	1.444106
ENSG0000(	OSBPL5	1890	2951	2640	2865	1593	1721	16.11971
ENSG0000(	DBP	269	450	475	565	660	693	6.580274
ENSG0000(	ZMYND11	630	790	631	1086	876	1056	4.979993
ENSG0000(	RPGRIP1L	257	312	194	408	387	462	1.920043
ENSG0000(	BCAP31	7177	8966	8883	9113	7140	6644	136.8975
ENSG0000(	DOCK4	84	103	131	63	76	94	0.362521
ENSG0000(	NAP1L1	10116	15749	15646	16824	24244	24953	97.06198
ENSG0000(	SVIL	564	916	737	678	2046	1603	2.633481
ENSG0000(	CRAT	2207	3160	3271	4550	3759	4283	27.5761
ENSG0000(	SPAG16	292	428	429	530	547	726	7.486908
ENSG0000(	OSBPL11	676	1012	783	750	706	839	5.075349
Homo_sapi	Homo_sapi	41	52	75	26	37	41	0.828258
ENSG0000(	DNAJB5	354	339	292	293	225	322	4.748776
ENSG0000(	MBOAT7	3529	5043	4587	4730	3724	3337	47.9953

ENSG0000(	PSMC3IP	430	434	491	567	1009	793	10.73579
ENSG0000(	FMN2	42	46	56	69	92	136	0.201068
ENSG0000(	INTS6	1337	1525	1030	1362	925	1067	9.562714
ENSG0000(	PYGB	1590	2006	1791	2109	1141	1254	12.11775
ENSG0000(	GRK5	555	949	948	491	738	722	6.577291
ENSG0000(	ELF2	674	888	728	957	1209	1398	6.290045
ENSG0000(	KLF4	511	478	442	335	471	342	5.749405
ENSG0000(	FAM210A	531	648	660	677	1589	1113	4.540979
ENSG0000(	MAGT1	3839	3900	3892	2922	3591	3925	31.62006
ENSG0000(	TMEM107	492	800	709	997	921	1035	11.83519
ENSG0000(	GABRA3	107	184	207	129	115	141	0.900013
Homo_sapi	Homo_sapi	1384	1496	1255	1596	2684	2757	8.607081
ENSG0000(	RELL2	136	147	122	132	94	84	1.858889
ENSG0000(	PPARG	1180	1488	1444	1474	958	1335	21.92557
Homo_sapi	Homo_sapi	112	143	110	195	169	254	2.198008
ENSG0000(	WIPF1	694	1194	942	1441	1387	1373	5.884926
ENSG0000(	EIF1AX	5216	5949	5533	5646	4809	5899	37.27107
ENSG0000(	CCDC84	735	756	698	792	322	609	19.16506
ENSG0000(	ZNF496	1412	1539	1146	2260	1952	2087	8.344711
ENSG0000(	MICAL2	1359	1556	1167	1478	1046	1001	12.18583
ENSG0000(	SMPD3	80	80	95	97	178	258	0.459572
ENSG0000(	ENDOG	413	563	398	994	662	615	11.06326
ENSG0000(	EPS8L2	3372	4130	3737	6844	4831	5154	35.33857
ENSG0000(	ZNF622	3875	4683	4225	4718	3418	4041	71.75983
ENSG0000(	KRT15	32	46	38	24	6	0	0.326285
ENSG0000(	ACOT13	2512	3376	2703	3087	1877	2778	19.11016
ENSG0000(	PSMC1	3176	3217	3047	3419	2257	2927	22.46427
ENSG0000(	CARD14	457	933	843	1094	1123	1346	4.145611
ENSG0000(	PI3	571	694	570	712	318	414	31.08533
ENSG0000(	C15orf38-A	161	193	104	110	61	146	2.07191
ENSG0000(	IGFBP3	4207	2849	3570	2555	32264	20153	58.08477
ENSG0000(	EEFSEC	942	1106	1048	1657	1292	1546	13.34293
ENSG0000(	ZNF579	232	360	211	489	498	366	2.715766
ENSG0000(	NLRP1	275	444	391	385	272	281	1.871826
ENSG0000(	ADAM22	66	129	139	143	206	304	0.532913
ENSG0000(	SLCO1B3	44	89	68	48	25	55	0.480075
ENSG0000(	DDX21	9349	12191	11066	10054	9879	11885	62.43062
ENSG0000(	HNRNPA1P	458	543	471	700	669	821	9.151101
ENSG0000(	RELB	261	377	231	275	229	195	3.599656
Homo_sapi	Homo_sapi	270	374	270	315	230	215	6.455346
ENSG0000(	TMEM141	2004	1987	2200	3559	2784	3044	82.08409
ENSG0000(	AADAT	209	209	229	544	290	324	2.952514
ENSG0000(	FFAR4	55	75	66	120	90	145	0.473318
ENSG0000(	MAST1	216	373	322	332	706	621	3.863996
ENSG0000(	SPRYD3	3265	3967	3930	4151	2961	3376	35.08755
ENSG0000(	OR51B5	106	258	222	403	289	331	3.266656
ENSG0000(	ALKBH8	232	238	259	229	71	207	2.036529
ENSG0000(	EP400	1990	2955	2636	3581	3877	3568	5.394338
Homo_sapi	Homo_sapi	6433	7462	7413	12281	10742	9090	145.2063
ENSG0000(	HAL	240	206	319	106	3527	3076	2.272327

ENSG0000(	SIDT2	619	902	796	1117	1113	1118	4.527135
ENSG0000(	RPL36A	14334	13612	13383	19820	20461	22354	583.7293
ENSG0000(	TVP23B	1046	1149	1007	933	971	1077	15.68375
Homo_sapi	Homo_sapi	59	89	91	82	194	228	0.734248
ENSG0000(	TPMT	2090	2623	2537	2639	1713	2359	20.6667
ENSG0000(	CHTF18	1010	1412	1214	1766	1617	1735	10.5087
ENSG0000(	PPARGC1A	258	302	328	250	821	774	1.307078
ENSG0000(	COMMD6	1191	1290	1380	1654	1941	2421	27.28387
ENSG0000(	HILPDA	856	829	849	807	4664	2761	20.52735
ENSG0000(	APOL2	869	970	856	994	696	691	10.95588
Homo_sapi	Homo_sapi	92	203	115	42	121	75	0.824226
ENSG0000(	MNS1	30	17	29	44	70	70	0.458996
ENSG0000(	DNM1	183	268	215	303	329	434	1.552635
ENSG0000(	WDR27	316	415	363	410	761	727	3.049482
ENSG0000(	PLCD1	186	290	298	230	198	232	1.982793
ENSG0000(	RPP25L	394	508	442	778	614	626	12.8969
ENSG0000(	METRNL	1466	1755	1427	1960	3172	2473	26.52063
ENSG0000(	IGF2BP2	399	656	618	766	879	921	3.612431
ENSG0000(	HKDC1	1486	2605	2314	3268	3119	3120	12.67302
ENSG0000(	SOD2	3917	5070	4810	4094	13105	9499	15.95217
ENSG0000(	ZNF33B	259	329	289	326	547	676	1.402018
ENSG0000(	ELF3	5868	5980	5195	8976	9897	7792	54.98735
ENSG0000(	SPOCK2	473	829	667	730	384	473	2.776446
Homo_sapi	Homo_sapi	22	50	38	59	59	131	0.229467
ENSG0000(	L1CAM	10118	15700	13539	13897	11262	9314	64.80537
ENSG0000(	PTH1R	19	59	36	99	57	90	0.274298
ENSG0000(	MUC16	1603	2493	2028	2476	4211	3306	2.505981
ENSG0000(	ATG16L2	263	563	462	510	870	868	3.252943
ENSG0000(	NISCH	2243	2524	2524	3895	3211	3422	16.35676
ENSG0000(	AGA	371	340	348	588	474	629	5.526442
ENSG0000(	C6orf132	301	266	217	288	127	164	1.889863
ENSG0000(	LUZP1	2373	3277	2773	3143	2241	2439	9.011825
ENSG0000(	ESYT3	174	245	235	305	410	346	1.282313
ENSG0000(	IPO13	1254	1880	1831	1822	1208	1392	10.30625
ENSG0000(	CCNY	1190	1211	1304	1725	1942	1939	9.404871
ENSG0000(	SYNPO	2535	3299	2827	6017	3247	4517	14.09258
Homo_sapi	Homo_sapi	16	37	43	46	80	95	0.26399
ENSG0000(	AMDHD1	282	246	305	207	745	939	3.91524
Homo_sapi	Homo_sapi	466	532	444	333	1880	1086	4.125854
ENSG0000(	ATRN	868	997	911	1319	1390	1356	3.813493
ENSG0000(	GON7	285	406	386	528	464	654	7.757057
Homo_sapi	Homo_sapi	211	280	216	282	476	458	3.310027
ENSG0000(	PRPF3	1903	2868	2735	2524	2129	2452	24.60386
ENSG0000(	AJM1	608	730	678	1076	897	956	4.358424
ENSG0000(	PCBP4	956	1003	1101	1112	767	902	14.72001
ENSG0000(	RETREG3	754	809	893	1336	1190	1156	6.689341
ENSG0000(	CCDC169	25	31	14	44	56	62	0.411813
ENSG0000(	IQSEC1	946	1327	1196	1756	1548	1616	6.070634
ENSG0000(	UTP6	1435	1894	1711	1738	1382	1774	10.58619
ENSG0000(	MPST	1618	2440	2221	3124	3362	2826	33.70986

ENSG0000(	MFSD12	12012	16871	17455	18796	25299	27572	234.4428
ENSG0000(	ZNF185	876	1424	1284	1274	851	1068	6.805091
ENSG0000(	FRAT2	1155	1385	1462	1601	2499	2339	16.4153
ENSG0000(	SPRY4	879	891	757	293	874	549	6.444928
ENSG0000(	LRP1	4068	6335	6109	6810	14071	8035	8.606203
ENSG0000(	MAP2K5	256	385	392	514	517	539	3.532223
ENSG0000(	PEX11G	115	251	245	337	342	352	3.218018
ENSG0000(	NBPF4	112	144	56	94	43	59	1.412198
ENSG0000(	DHX58	323	344	284	351	167	239	4.001779
ENSG0000(	SPIN4	242	393	316	365	588	664	1.846992
ENSG0000(	AC000093.	165	122	305	167	117	124	1.579537
ENSG0000(	TSC2	1700	2258	1749	3879	2334	2731	9.90433
ENSG0000(	ARGLU1	2971	3368	2974	3337	5752	6811	27.57578
ENSG0000(	CA9	497	951	1496	756	8205	4952	9.6584
ENSG0000(	SLC5A3	828	1187	989	815	3076	2167	2.250549
ENSG0000(	PROCA1	80	91	108	127	157	229	2.614264
ENSG0000(	PTPN14	1435	1998	2041	2371	2593	3257	3.47641
Homo_sapi	Homo_sapi	145	291	296	295	628	445	4.266647
ENSG0000(	LRRC27	182	252	183	447	269	313	3.021731
ENSG0000(	EFNB1	317	519	435	453	311	264	3.008985
ENSG0000(	ACSM3	91	146	125	178	166	300	1.399831
ENSG0000(	SNED1	546	811	745	474	1651	2469	3.179994
ENSG0000(	HID1	220	385	319	310	209	260	2.364322
ENSG0000(	MARCKS	786	1002	1014	1441	1426	1266	5.781005
ENSG0000(	MNT	666	1074	1008	1096	1827	1587	4.188703
ENSG0000(	SLC29A2	757	866	726	1520	964	1187	9.667698
ENSG0000(	FAM131C	286	348	391	525	496	529	5.294036
ENSG0000(	TMEM198	109	142	101	336	138	203	1.560452
ENSG0000(	ZBTB11	936	1264	1110	1015	982	1180	4.952092
ENSG0000(	BCR	1830	2436	2390	2508	1734	1658	8.130244
ENSG0000(	GYS1	2437	3048	2777	4947	3823	3565	21.534
Homo_sapi	Homo_sapi	43	54	42	29	30	34	1.202073
ENSG0000(	PCGF6	549	704	584	659	487	549	7.718388
ENSG0000(	PCSK6	1528	1945	1447	2275	2534	2479	11.53081
ENSG0000(	PKD1L2	39	74	130	121	225	177	0.250085
ENSG0000(	TMED2	9947	10913	10319	9464	9609	11021	122.4427
ENSG0000(	C1orf226	1198	1408	1227	1157	1203	1289	9.265236
ENSG0000(	SDR39U1	434	566	410	807	569	802	11.83991
ENSG0000(	PPL	4096	6734	6358	8095	9688	8224	20.65899
ENSG0000(	PLCG1	2519	3691	2990	5188	4322	3984	13.57333
ENSG0000(	CC2D2A	213	320	271	428	355	425	1.81839
Homo_sapi	Homo_sapi	88	132	100	118	230	262	0.980676
ENSG0000(	GARNL3	88	118	119	127	221	253	1.033175
ENSG0000(	LPAR5	189	288	207	252	127	145	1.870297
Homo_sapi	Homo_sapi	5519	6243	6062	10176	8875	7621	114.4758
ENSG0000(	SS18L1	630	671	614	831	1253	1030	5.222031
ENSG0000(	KCNJ12	246	380	280	309	162	268	1.423849
ENSG0000(	ZC3HAV1	4135	3499	3390	3450	3114	3406	24.50681
Homo_sapi	Homo_sapi	75	86	95	175	154	122	1.197699
ENSG0000(	TUBB3	1564	2141	2064	2193	1409	1105	27.01171

ENSG0000(	DAB2IP	1603	2085	2030	2186	1409	1547	7.56402
ENSG0000(	CAPN5	111	199	171	170	61	101	0.788131
ENSG0000(	CTPS2	1402	1757	1580	2178	1999	2608	11.43227
ENSG0000(	HSPA2	277	318	316	417	412	678	3.144626
ENSG0000(	C11orf71	100	111	112	122	190	340	1.587118
ENSG0000(	SPATS2L	848	793	601	782	529	588	9.260294
ENSG0000(	PSPH	1629	2315	1869	2009	914	1879	29.66711
ENSG0000(	TUT1	558	522	491	530	364	494	5.736138
ENSG0000(	TMCO6	582	786	678	1089	822	1064	11.38999
ENSG0000(	CERK	1502	1751	1785	2518	2367	2337	10.61578
ENSG0000(	TMEM38A	1083	1315	1122	1251	977	1148	12.85911
ENSG0000(	RPS16	22084	23912	23390	34341	34575	31979	1249.334
ENSG0000(	DPCD	607	703	576	1024	951	866	22.51238
Homo_sapi	Homo_sapi	7	34	39	52	106	55	0.167768
ENSG0000(	SGMS2	713	1021	925	714	810	911	4.143263
ENSG0000(	WTIP	162	197	160	525	254	217	0.371578
ENSG0000(	STX4	1566	1944	1775	1946	1501	1399	35.85653
ENSG0000(	APC	461	711	651	548	473	640	2.146624
ENSG0000(	KLHL30	126	183	154	146	124	94	1.055791
ENSG0000(	ITSN2	460	549	464	380	450	489	2.992451
ENSG0000(	LARP4B	1516	1708	1443	2303	2113	2327	6.602594
ENSG0000(	ATXN2L	3476	4705	3850	6075	5832	5315	30.06434
ENSG0000(	SLC25A38	1188	1466	1315	1853	1962	1884	17.7266
ENSG0000(	POLR1D	1868	2934	2797	3154	4384	4201	65.27603
Homo_sapi	Homo_sapi	84	82	46	86	173	199	1.316972
ENSG0000(	STAG3	74	96	70	104	154	184	0.694007
ENSG0000(	SLC38A1	7830	10813	9741	7610	8838	10041	31.65572
ENSG0000(	KCNH3	40	55	74	120	82	118	0.317169
ENSG0000(	NFE2L1	9273	12196	10131	11474	9410	9693	63.31498
ENSG0000(	SLC9A3	1670	2069	1776	2364	2709	3004	10.22185
ENSG0000(	NBAS	699	1112	1002	1125	1561	1752	3.031374
ENSG0000(	MFHAS1	255	357	408	430	723	579	1.250594
ENSG0000(	GTF2A2	3656	4203	4110	4690	2702	3234	79.96223
ENSG0000(	MCM7	13717	15056	13837	21279	18366	20913	144.9943
ENSG0000(	SLC35B1	3256	2782	2283	3335	1266	1718	78.29267
ENSG0000(	LTBP4	2772	3820	3373	4105	5478	5381	18.61517
ENSG0000(	CLINT1	3585	3841	3373	3633	3141	3626	34.67357
ENSG0000(	COL4A4	516	676	633	650	1282	1209	1.619073
ENSG0000(	CIPC	208	290	249	329	383	454	1.457937
ENSG0000(	COL9A3	333	603	557	702	745	860	4.191106
ENSG0000(	SH2D3A	702	997	1053	846	792	911	11.67165
ENSG0000(	ITPRIP	1571	1671	1416	1828	3410	2684	10.20035
ENSG0000(	IER3IP1	1170	1740	1662	2085	2324	2267	10.19612
ENSG0000(	ZFP36L1	1339	1545	1248	1560	3332	2347	13.52138
ENSG0000(	VWDE	44	62	44	60	110	139	0.261866
Homo_sapi	Homo_sapi	234	373	297	305	553	822	3.237153
ENSG0000(	MLF1	599	897	1021	1143	1330	1429	9.551827
ENSG0000(	DNTTIP2	1994	2769	2783	2517	2237	2522	10.6381
ENSG0000(	SNX33	3416	3322	3289	3050	9716	6727	13.41867
ENSG0000(	CD55	42838	55972	57850	34184	49723	52237	537.0102

ENSG0000(	TMEM164	1581	2480	2249	2127	1798	2044	9.215879
ENSG0000(	DYRK1B	140	151	132	293	201	214	1.808625
ENSG0000(	SSH1	1189	1914	1585	1369	1457	1511	3.351199
Homo_sapi	Homo_sapi	391	520	441	694	568	703	9.258967
Homo_sapi	Homo_sapi	49	86	86	55	247	221	0.480356
ENSG0000(	EEF2K	1882	2571	2538	2844	3935	3919	8.146369
ENSG0000(	NR4A1	2718	3759	3801	2826	14487	13129	35.97306
ENSG0000(	SLC26A6	1373	1853	1844	2109	3826	2522	16.98482
ENSG0000(	SRXN1	2180	3632	3272	3217	2291	2756	25.41925
ENSG0000(	TROAP	2465	2896	2684	3109	1901	2300	71.88767
ENSG0000(	P4HTM	1692	2282	2378	2813	3007	3429	25.06718
ENSG0000(	NPR1	3146	3776	3403	6739	4097	5005	23.36522

shNC05_FP	shNC06_FP	sh01_FPKM	sh02_FPKM	sh03_FPKM	Pvalue	log2FC	regulated
0	0.068779	2.24006	4.064103	3.335955	1.65E-67	3.863552	up
24.31536	22.36196	4.411798	4.89938	4.065672	2.79E-44	-2.02092	down
6.11095	7.290907	0.653913	0.790367	0.36936	1.04E-20	-2.10449	down
15.17245	14.87437	6.358267	6.022599	5.667847	3.76E-20	-1.19722	down
7.006735	5.981751	2.34466	2.378518	2.293852	1.18E-18	-1.25649	down
2.344361	1.539333	5.253052	4.481961	8.457801	4.98E-18	1.43302	up
10.61644	9.300777	3.135156	4.340328	3.224411	3.85E-17	-1.35098	down
1.104028	0.986603	2.947379	3.724631	3.421723	1.30E-13	1.349769	up
2.109226	2.215578	0.720078	0.457322	0.74291	2.60E-13	-1.36477	down
2.207115	2.494567	4.689453	6.017769	7.206932	4.33E-12	1.215336	up
14.9344	14.46008	21.20305	25.60511	27.59851	8.78E-12	0.884556	up
0.550565	0.567788	0.008209	0.036847	0.022362	1.92E-11	-1.49401	down
0.165153	0.124375	0.531861	0.604662	1.040485	3.62E-11	1.46889	up
0.258122	0.78182	3.996766	2.714979	3.321996	3.90E-11	1.51319	up
0.751436	0.63876	1.272683	1.820985	2.743497	8.98E-11	1.26757	up
1.309595	2.431252	0.475988	0.580805	0.915739	2.17E-10	-1.26893	down
17.57342	20.32126	5.477861	7.748421	6.581325	2.33E-10	-1.09747	down
0.225064	0.189555	1.892432	2.375202	10.42821	2.81E-10	1.412274	up
1.77461	1.456418	0.322138	0.254934	0.18348	3.52E-10	-1.41942	down
13.31151	13.5888	29.19392	36.65119	25.89415	3.87E-10	1.041328	up
8.821202	8.740656	4.940784	3.361538	3.903726	6.06E-10	-0.93426	down
25.85993	33.72118	12.71328	9.356689	9.012302	7.92E-10	-1.13377	down
4.665949	3.522767	0.417619	0.24214	0.052255	1.41E-09	-1.37251	down
0.095503	0.156835	0.840877	0.789296	0.777758	2.19E-09	1.33819	up
0.698312	0.533668	0.221919	0.219421	0.243878	5.44E-09	-1.11732	down
2.521607	2.693381	0.726151	0.300052	0.582523	9.08E-09	-1.32091	down
0.423931	0.419293	0.055709	0.049707	0.023827	4.54E-08	-1.25343	down
1.936822	1.79368	0.792091	0.613591	0.638258	6.55E-08	-1.02551	down
12.78595	13.88574	19.3467	20.42989	23.19519	1.22E-07	0.682822	up
0.699901	0.553491	0.18459	0.228355	0.244198	1.44E-07	-1.07059	down
6.976615	6.339812	10.88556	15.99494	14.01225	1.50E-07	0.861668	up
4.14258	3.835928	1.773637	1.190771	1.01143	2.03E-07	-1.06466	down
9.493301	8.959979	3.811881	1.347682	1.541722	2.25E-07	-1.16075	down
11.2606	11.25154	4.902738	6.536199	6.812205	3.10E-07	-0.78545	down
0.913372	1.054231	2.047284	2.365986	3.975816	5.74E-07	1.04905	up
4.445937	3.502329	1.861139	0.989569	0.631914	6.71E-07	-1.11001	down
0.99011	1.750355	2.254247	1.991672	2.31185	7.59E-07	0.82713	up
114.5064	120.8915	262.0733	235.6632	270.4323	7.90E-07	0.829796	up
0.254615	0.286588	0.451064	3.617955	2.83911	8.50E-07	1.105367	up
1.018303	1.601026	0.129541	0.193832	0.063882	9.15E-07	-1.1093	down
1.727171	1.903341	0.20202	0.198453	0.710676	9.22E-07	-1.12279	down
7.591036	6.982238	3.364728	3.352773	4.451586	1.01E-06	-0.85793	down
9.56112	8.467174	15.16697	13.53177	17.47838	1.03E-06	0.733893	up
4.731483	4.784218	2.130725	2.716213	2.632258	1.14E-06	-0.70554	down
21.62049	24.69076	35.27586	40.34518	36.74419	1.26E-06	0.711669	up
12.22024	13.33796	3.471389	6.187878	3.808599	1.39E-06	-1.05335	down
1.569249	1.482998	0.721791	0.345836	0.431594	1.42E-06	-1.05999	down
30.05494	32.0642	39.84864	74.66011	67.18493	1.52E-06	0.907887	up
8.705254	8.241019	1.492077	2.12906	3.556257	1.83E-06	-1.03051	down

4.341427	3.930113	0.413077	1.626636	0.891107	2.12E-06	-1.08989	down
23.75048	21.66475	30.33588	37.04376	35.41295	2.56E-06	0.671272	up
3.385577	3.141621	1.836505	1.881313	1.754492	2.61E-06	-0.6425	down
75.22721	81.2983	101.2316	122.7839	122.0032	2.77E-06	0.603687	up
2.209838	1.796979	0.648742	0.764403	0.857545	2.81E-06	-0.91709	down
3.32751	2.884394	1.155954	1.563264	1.51074	3.08E-06	-0.7943	down
5.940407	4.228753	2.394573	2.276626	1.734436	3.10E-06	-0.91787	down
13.37206	13.82201	20.95476	34.47903	30.10991	3.20E-06	0.832563	up
0.410478	0.422178	0.17621	0.162363	0.146664	3.58E-06	-0.9258	down
14.94343	21.05749	23.63685	55.20871	75.23463	4.02E-06	1.015595	up
15.44378	16.99605	8.437535	9.882026	8.677239	4.09E-06	-0.66827	down
433.437	492.8051	220.5479	228.029	205.0976	4.21E-06	-1.01241	down
236.9784	209.516	135.8029	122.7282	106.0554	4.38E-06	-0.70812	down
3.905769	2.901739	1.161573	1.277714	1.753107	5.52E-06	-0.86209	down
145.5715	166.926	91.16449	74.53473	64.56771	6.38E-06	-0.92076	down
643.4398	602.2976	815.2809	1235.105	1018.106	7.67E-06	0.687689	up
40.08494	39.09491	51.04134	54.51025	59.96042	8.80E-06	0.502633	up
2.285473	2.025502	3.887197	3.574455	4.33932	9.04E-06	0.782596	up
0.278407	0.325796	0.075684	0.080277	0.064737	9.05E-06	-1.01883	down
33.07122	31.60029	44.03808	45.62368	45.66989	9.25E-06	0.52793	up
9.003659	9.036255	7.984029	5.836627	9.17064	1.05E-05	-0.57305	down
10.02052	9.136571	3.210992	2.087996	3.829251	1.09E-05	-0.93291	down
0.071664	0.033274	0.165964	0.241735	0.21825	1.09E-05	1.010979	up
14.6247	14.00628	7.708229	8.673704	8.040031	1.15E-05	-0.59033	down
95.13131	100.0485	168.4925	149.4793	154.5936	1.16E-05	0.619733	up
1.970155	2.320116	2.897349	5.076365	3.455118	1.22E-05	0.899202	up
4.482529	3.964692	1.305955	1.571159	2.203143	1.36E-05	-0.88962	down
1.030807	0.890457	1.318043	1.370978	1.664582	1.36E-05	0.660796	up
0.570744	0.563883	0.204235	0.151192	0.283141	1.37E-05	-0.94571	down
7.616216	7.930808	5.665179	4.057352	4.155045	1.44E-05	-0.70317	down
15.25781	13.19758	8.964022	9.344281	8.575527	1.55E-05	-0.71802	down
5.59295	7.114464	1.139639	3.330292	1.006459	1.56E-05	-0.98837	down
11.16142	8.996481	3.722525	1.500958	1.420128	1.74E-05	-0.98035	down
27.72754	29.05979	16.56796	14.0777	19.04314	1.81E-05	-0.60641	down
1.052577	1.280437	0.435115	0.541749	0.517721	1.98E-05	-0.96123	down
6.928145	7.262729	3.544824	2.98507	3.4389	2.33E-05	-0.72269	down
41.58582	47.34877	58.95804	122.9155	88.58108	2.36E-05	0.829894	up
4.760864	5.773039	2.265454	1.224192	1.216632	2.43E-05	-0.96851	down
9.365916	9.90169	4.76806	3.471248	4.984553	2.69E-05	-0.74193	down
21.13662	21.23994	13.42334	11.52717	10.14232	2.71E-05	-0.67088	down
0.04902	0.151776	0.413949	0.51082	0.465159	2.71E-05	0.964527	up
8.60169	7.397459	9.998219	21.23221	17.99654	2.73E-05	0.877143	up
23.86225	27.28678	15.97559	10.78782	20.09213	2.76E-05	-0.67426	down
143.5933	148.5297	198.3621	206.9103	194.9782	2.81E-05	0.499158	up
16.68103	17.82682	9.726879	11.73681	10.6269	2.91E-05	-0.54736	down
22.8715	24.16643	16.20985	16.0575	16.43834	2.91E-05	-0.52286	down
531.4463	688.015	207.644	352.6885	286.692	2.93E-05	-0.76139	down
2.78425	2.421028	0	0	0.332129	3.05E-05	-0.82318	down
1.00976	0.852954	0.583534	0.447461	0.506257	3.17E-05	-0.68212	down
113.4584	108.1217	37.42335	41.14324	68.5509	3.35E-05	-0.81075	down

43.06057	45.82736	29.72404	27.68849	28.04711	3.49E-05	-0.64215	down
7.690983	7.730175	2.597506	4.135388	4.619591	3.51E-05	-0.73976	down
2.593946	3.076063	1.023149	0.530131	0.542066	3.70E-05	-0.94663	down
2.004523	2.002358	0.898019	0.403467	0.384429	3.86E-05	-0.93724	down
9.192864	7.512842	29.88519	16.04417	26.45458	3.93E-05	0.881181	up
1.274672	1.439183	1.112757	1.188262	0.785335	4.05E-05	-0.73667	down
2.960176	3.529988	1.723123	1.531249	1.569087	4.06E-05	-0.64847	down
235.6916	231.9255	157.6817	138.6865	158.5155	4.11E-05	-0.65297	down
16.0252	15.42061	24.5095	21.68627	26.58977	4.26E-05	0.677183	up
4.902637	3.949729	8.127016	7.39966	8.13949	4.28E-05	0.670836	up
3.018818	2.714201	1.585997	1.516853	1.157169	4.33E-05	-0.77218	down
13.09779	12.6823	19.38856	59.66608	33.15261	4.51E-05	0.908488	up
85.28456	92.80216	106.0597	128.6699	134.7562	4.64E-05	0.523497	up
0.94955	1.04501	0.318896	0.280254	0.378246	5.13E-05	-0.89908	down
4.117697	4.556072	2.66725	2.658315	3.033076	5.21E-05	-0.55793	down
6.82323	7.508369	1.227611	2.410441	3.324221	5.43E-05	-0.88994	down
42.23334	35.83721	21.40232	11.68549	7.238962	5.53E-05	-0.90141	down
5.431758	6.497351	3.053496	3.951584	3.880505	5.62E-05	-0.73327	down
1.352129	1.830189	0.290358	0.41609	0.474216	5.90E-05	-0.92128	down
0.721524	0.900233	0.377742	0.135812	0.319914	6.38E-05	-0.90539	down
5.152579	5.553285	1.636011	3.46062	1.784834	6.39E-05	-0.90937	down
1.201246	1.0933	0.391586	0.60626	0.593855	6.79E-05	-0.80916	down
17.89187	19.66662	23.90171	40.83611	40.46497	7.13E-05	0.727432	up
0.771905	0.984775	0.132405	0.108335	0.319636	7.21E-05	-0.87828	down
1.456237	1.826643	0.81529	0.689014	1.008932	7.39E-05	-0.76021	down
55.22593	51.65956	61.86262	175.3067	202.8044	7.58E-05	0.879401	up
9.257041	12.91862	12.83042	38.91806	29.27302	8.09E-05	0.875437	up
33.87976	34.52697	23.02406	21.22964	21.5326	8.28E-05	-0.50863	down
0.336063	0.379824	0.91595	1.604519	2.084571	8.32E-05	0.898435	up
88.0977	79.55611	123.5061	125.6433	140.7027	8.48E-05	0.515489	up
12.49473	15.59878	18.70619	26.33295	30.96389	8.57E-05	0.766845	up
13.82076	15.13405	7.873195	5.517859	5.058201	8.58E-05	-0.76164	down
8.540722	8.092336	3.974279	5.388249	5.392031	8.75E-05	-0.56834	down
11.80672	10.27498	12.22112	40.55013	35.8772	8.85E-05	0.875083	up
12.34329	12.61547	8.00585	8.635386	7.892115	9.14E-05	-0.49533	down
6.951966	6.882223	4.556119	4.412847	4.484315	9.61E-05	-0.56383	down
0.435284	0.647584	1.624263	1.041136	2.832401	0.000103	0.885637	up
2.597637	1.912681	0.286646	0.554924	0.613971	0.000105	-0.85325	down
4.272797	5.465726	8.601351	11.87759	15.82861	0.000106	0.81108	up
8.460167	12.38113	14.04641	17.98341	14.06555	0.000109	0.62178	up
8.538836	8.47045	10.93199	12.1619	13.25143	0.000113	0.508719	up
77.39573	70.31456	89.81473	96.99927	106.5325	0.000116	0.444894	up
12.19747	12.60983	8.326999	6.636663	8.050962	0.000117	-0.58327	down
8.924457	8.004292	4.1156	3.204451	3.033488	0.000121	-0.73598	down
1.339999	2.245219	0.774301	0.344108	0.476677	0.000123	-0.8821	down
0.574501	0.515078	0.093244	0.108152	0.156835	0.000124	-0.87917	down
5.017089	5.306524	6.754296	9.071687	11.17186	0.000126	0.7726	up
14.86518	16.06064	9.717793	9.510025	9.163474	0.000133	-0.51778	down
9.319534	8.618702	12.39843	25.6042	30.83246	0.000135	0.809967	up
2.756789	2.567433	3.608909	4.294948	4.731305	0.000136	0.60447	up

1.245031	2.666943	0.248511	0	0.313261	0.000139	-0.76964	down
0.661855	0.48534	0.046474	0	0.099745	0.00014	-0.78532	down
13.00242	11.01706	4.862416	7.879403	7.139011	0.00014	-0.65933	down
279.2791	292.0974	323.5875	516.2601	462.1418	0.000144	0.64174	up
1.697203	2.90091	0.480405	0.855745	0.713056	0.000145	-0.8549	down
4.536673	3.58135	1.868457	0.693839	0.390203	0.000147	-0.87193	down
2.393061	1.82827	2.623593	3.897647	3.468804	0.000152	0.673839	up
0.494586	0.684209	1.183459	1.081303	1.227914	0.000154	0.776083	up
104.55	114.0999	159.6959	138.1432	156.7488	0.00017	0.483539	up
45.36746	46.40556	28.70891	14.71456	19.56354	0.00017	-0.73962	down
0.47302	0.417808	0.756589	1.291043	1.487103	0.00017	0.839191	up
4.208808	3.525377	2.17342	1.518845	1.29979	0.000172	-0.84622	down
22.90701	26.18195	16.85582	12.63871	16.24956	0.000176	-0.56654	down
0.997415	1.199662	1.306324	3.18908	4.579293	0.000185	0.85668	up
48.72631	47.73096	32.24597	24.18708	22.80454	0.000185	-0.79802	down
122.9613	129.797	173.5659	169.4057	167.1489	0.000186	0.450488	up
52.64734	62.83986	38.24423	38.06288	35.10157	0.00019	-0.5523	down
9.737496	10.54104	13.30893	15.10801	14.89431	0.000194	0.628292	up
0.73736	0.864581	1.200625	4.568031	3.738742	0.000197	0.851134	up
0.878736	0.757663	0.883277	1.21107	1.384779	0.000223	0.741558	up
23.24314	21.48681	27.3671	32.49935	33.45526	0.00023	0.548987	up
0.914874	0.620687	0.355525	0.333718	0.175097	0.00024	-0.82123	down
1.064514	1.035835	1.828755	1.811251	1.868135	0.000242	0.673129	up
353.6995	412.4321	248.6424	181.5003	214.5694	0.000254	-0.59379	down
2.323382	2.311981	3.707125	3.269805	3.786638	0.000262	0.570581	up
1.337476	0.969382	0.652704	0.146194	0.208227	0.000281	-0.81773	down
1.013679	1.171958	0.606276	0.41938	0.286468	0.000283	-0.83199	down
11.21645	11.0925	7.754083	5.465554	6.227503	0.000286	-0.60941	down
20.13507	19.08482	13.77442	10.74618	9.85011	0.000288	-0.71946	down
9.031222	8.289436	14.62829	11.26248	14.61222	0.000294	0.487224	up
15.90687	18.15297	10.99584	11.36942	10.58889	0.000297	-0.5181	down
1.893652	1.597248	1.173422	0.81422	0.571137	0.000303	-0.82328	down
6.880606	6.000019	7.530758	12.32705	10.55618	0.000306	0.632112	up
18.75979	17.60624	8.822727	10.12496	11.24683	0.00031	-0.57346	down
1.12067	1.107815	1.92732	2.767076	4.332505	0.000327	0.730989	up
5.282915	6.54608	10.03873	11.47692	9.876961	0.000332	0.628009	up
5.170973	4.000615	2.104415	2.856817	2.443574	0.000332	-0.72826	down
0.442546	0.61473	0.210971	0.230171	0.137965	0.000337	-0.82356	down
0.624394	1.486901	0.256663	0.193222	0.265635	0.000342	-0.82319	down
14.49755	14.20082	20.49513	22.19475	23.46133	0.000343	0.520081	up
0.557301	0.811049	0.160309	0.10334	0.225882	0.000349	-0.82194	down
19.97771	20.75983	36.41984	26.63857	32.9579	0.000355	0.587229	up
10.7208	10.79632	13.39318	18.13115	14.48692	0.000358	0.581328	up
22.30872	22.9068	14.07336	15.27619	16.82056	0.000361	-0.41846	down
7.1802	8.701351	15.03337	13.92149	13.63646	0.000364	0.56115	up
9.830037	9.822038	5.073863	4.907445	5.914295	0.000366	-0.46862	down
37.7166	46.12207	24.53948	25.88701	27.6709	0.000372	-0.47602	down
22.98736	27.2625	27.93404	69.49681	73.1513	0.000382	0.767354	up
2.47341	2.10182	0.832106	1.175236	1.441761	0.000385	-0.6977	down
77.38014	77.94168	82.20401	131.2695	147.3237	0.000386	0.63242	up

22.18906	20.93277	33.79597	29.87913	29.48752	0.000388	0.501781	up
58.67858	57.68263	37.4225	21.11874	32.69456	0.000389	-0.65435	down
1.642291	1.400253	0.771826	0.132047	0.286364	0.000389	-0.80506	down
17.87526	18.12731	18.223	50.3912	36.97986	0.000415	0.77228	up
22.84548	23.39125	11.21757	13.89114	16.4189	0.000415	-0.55808	down
20.22259	19.89142	10.42917	12.83777	12.81828	0.000433	-0.4993	down
96.80606	109.7955	121.2696	151.0755	141.2885	0.000442	0.471294	up
0.10458	0.182515	0.248538	0.392652	0.400783	0.000443	0.780168	up
0.166272	1.010366	1.368225	3.003474	1.736506	0.000443	0.79035	up
2.968071	3.136782	2.204294	1.80523	1.682783	0.000451	-0.58423	down
7.343973	8.022969	5.457224	4.492088	3.626356	0.000453	-0.71266	down
127.2985	131.8087	166.7686	293.5347	316.839	0.000456	0.68441	up
47.97972	57.96554	29.32679	29.13162	34.00995	0.000457	-0.52782	down
3.724366	3.526138	4.677957	4.979611	5.326579	0.000457	0.487696	up
17.86696	19.3719	21.68156	28.88324	25.32348	0.000457	0.531843	up
1.167511	1.285019	1.391198	4.864162	4.334094	0.000466	0.801312	up
131.5912	120.2237	151.3062	203.6685	198.7349	0.00047	0.506491	up
0.469108	0.485429	0.17109	0.250103	0.226894	0.000473	-0.72772	down
2.246589	3.09987	2.691403	3.410509	4.352586	0.000486	0.640165	up
2.033947	2.551821	1.008336	1.268357	1.229968	0.000488	-0.64755	down
67.63543	68.60363	48.48312	39.10827	37.34449	0.000506	-0.55925	down
8.892762	9.202236	5.353156	6.032406	6.555979	0.000511	-0.49223	down
0.191285	0.232284	0.282818	1.269298	0.980379	0.000531	0.783225	up
0.567787	0.731535	0.465588	0.391117	0.354926	0.000534	-0.72366	down
2.752145	2.35863	2.412303	10.86284	6.750662	0.000538	0.794339	up
0.691309	0.543271	1.17653	3.280613	2.954956	0.000546	0.791864	up
1.17402	1.143995	0.159016	0.571717	0.508188	0.000556	-0.79331	down
11.10561	10.49964	13.93184	13.87064	14.55118	0.000572	0.410527	up
5.988126	6.555064	7.569661	11.42356	9.119907	0.000573	0.587228	up
15.72225	16.2506	11.38981	11.029	11.0783	0.00058	-0.44877	down
2.90346	2.768903	3.257938	5.635326	5.813768	0.000581	0.696628	up
5.218186	5.759568	6.944796	10.2996	8.371791	0.000582	0.544434	up
3.991506	3.835223	2.325518	2.675937	2.520635	0.000598	-0.48772	down
45.79072	44.95245	53.34153	64.00006	58.50945	0.000623	0.475193	up
3.01738	3.092861	1.955359	1.4675	2.181255	0.000625	-0.59644	down
1108.65	1261.56	1312.125	1920.783	2910.654	0.000627	0.666566	up
1.645613	1.495684	0.74917	0.213559	0.513224	0.000629	-0.78165	down
5.073144	4.790555	6.435803	7.668293	7.225726	0.000649	0.512498	up
37.67537	39.77443	24.97837	26.68317	29.21775	0.000675	-0.39003	down
8.308575	7.502908	11.4062	13.00117	11.83073	0.000681	0.52154	up
0.374232	0.349687	0.050266	0.060207	0.157449	0.000682	-0.76895	down
16.62221	16.10503	22.87931	20.09109	21.30726	0.0007	0.463875	up
0.702502	0.541887	0.167773	0.07044	0.180998	0.000704	-0.76718	down
31.18858	29.57766	41.50402	50.28543	39.25633	0.000726	0.499769	up
1986.097	1998.838	2480.794	2998.834	2850.96	0.000732	0.440327	up
14.94772	13.77516	17.676	17.83628	18.56314	0.000739	0.410042	up
1.000911	0.785269	0.664481	3.489052	3.060692	0.000746	0.762866	up
121.7774	132.0186	129.6927	282.437	221.8942	0.00075	0.677486	up
28.29817	30.24967	15.1841	15.37932	17.29206	0.000753	-0.54863	down
24.26403	20.47375	25.68402	36.85298	31.87313	0.000759	0.482571	up

0.984486	1.272908	1.652473	2.208756	2.393153	0.000765	0.709757	up
1.57905	2.033204	3.680864	4.138951	3.821748	0.000788	0.683727	up
2.195948	2.033126	1.254994	0.891182	1.113679	0.000796	-0.66253	down
0.226782	0.235904	0.089506	0.073821	0.112376	0.000812	-0.76971	down
0.827619	0.939912	1.462841	1.847807	1.529318	0.000823	0.673555	up
15.68206	15.24778	11.67492	9.350126	9.615613	0.000828	-0.52599	down
1.866594	2.129981	1.428398	0.923	1.107521	0.000859	-0.68781	down
1.647629	1.361309	3.413585	3.180879	2.762468	0.000867	0.699654	up
30.74497	30.69503	32.95499	63.46326	59.12003	0.00087	0.626546	up
7.101783	7.653247	4.797468	5.540059	5.914423	0.000872	-0.67782	down
2.861939	3.746999	1.212624	1.209857	1.090362	0.000874	-0.76157	down
193.7434	232.3582	251.0896	290.2395	269.643	0.000895	0.427281	up
2.82624	4.977434	1.837574	1.577231	1.296256	0.00093	-0.73208	down
92.0958	81.91491	53.81764	21.95277	30.72196	0.000939	-0.71763	down
13.894	13.21484	7.892307	8.179114	9.720186	0.000949	-0.45546	down
0.606418	0.533716	0.228999	0.203664	0.176272	0.000957	-0.75877	down
20.82674	20.3825	13.41377	12.49133	14.75574	0.000958	-0.41156	down
1.894818	1.598881	0.612539	0.832368	0.984993	0.000973	-0.6191	down
6.151325	6.316563	3.717351	3.084803	3.542677	0.000973	-0.56529	down
375.6189	449.0207	518.8451	605.2474	546.0955	0.000981	0.435745	up
38.04068	39.72114	44.48087	55.50249	68.15953	0.000983	0.493726	up
5.711241	6.265234	3.773338	2.576673	2.6427	0.000986	-0.62456	down
151.2388	160.9697	105.4839	111.9858	125.3456	0.000987	-0.4061	down
3.211199	2.433498	4.415063	4.503116	6.052371	0.000992	0.659518	up
124.5705	141.1356	142.097	216.8424	206.9337	0.000998	0.554293	up
3.462946	3.307371	1.821455	0.783689	1.509031	0.001001	-0.7174	down
1.826915	2.472191	1.955448	9.479169	7.649625	0.001002	0.75607	up
6.092956	6.022424	8.617815	7.789239	7.419793	0.001009	0.474531	up
28.13235	26.90546	33.58703	61.0968	45.19497	0.001012	0.623671	up
3.170774	2.286125	0.721049	1.549846	1.640058	0.001023	-0.75181	down
8.278038	11.05735	9.453004	27.79874	20.91528	0.001033	0.729711	up
7.260298	7.026469	8.955441	8.829685	9.400916	0.00104	0.409051	up
7.817178	8.644401	2.466128	3.70816	3.172456	0.001042	-0.71855	down
39.48096	45.45006	51.99607	59.9708	48.69653	0.001046	0.464576	up
10.30479	9.70317	15.47566	12.08269	14.82992	0.001049	0.592739	up
32.29604	40.4438	27.5886	16.30534	12.66148	0.001061	-0.73428	down
45.61812	40.04484	28.01855	32.08575	30.94968	0.001063	-0.42244	down
1.647084	2.307462	0.620647	1.771315	1.434706	0.001066	-0.67794	down
1.299619	1.714651	1.827712	3.018179	2.475042	0.001094	0.66786	up
4.961207	3.688648	2.136201	1.083278	1.61417	0.001095	-0.72124	down
26.87423	24.40203	33.51772	36.91994	30.27062	0.001124	0.462706	up
2.209066	1.962581	1.51863	0.954775	1.209594	0.001126	-0.62584	down
4.316368	4.01437	10.24219	5.549898	6.454041	0.001146	0.631667	up
72.26609	79.68843	31.15191	35.67929	46.12293	0.001147	-0.60447	down
2.167053	2.056075	3.912745	3.605013	3.215082	0.001155	0.607926	up
4.154519	3.335721	4.805232	5.252288	6.778058	0.001171	0.540756	up
50.49565	53.70972	36.49407	25.62795	21.84541	0.001176	-0.61195	down
74.31661	85.92111	93.9225	104.3658	102.4383	0.00118	0.410306	up
0.756235	0.690256	1.020154	1.510842	1.71927	0.001196	0.728083	up
23.49214	25.84109	23.53755	73.1623	46.59429	0.001197	0.71348	up

12.2698	11.72843	14.02486	18.7003	16.85752	0.001206	0.56508	up
6.311157	7.062741	7.898573	19.66209	13.50098	0.00125	0.687641	up
6.818644	6.764712	4.327763	4.616151	3.983881	0.001254	-0.54326	down
176.0462	177.8218	189.1652	387.1856	277.0279	0.001261	0.574132	up
14.10516	21.98011	9.128937	8.969517	10.58052	0.001269	-0.43356	down
27.65699	39.14206	32.59085	42.35922	52.32823	0.001276	0.513841	up
102.7486	92.27247	133.82	113.4579	130.3775	0.00128	0.43415	up
1.646746	1.829976	1.114033	0.607774	1.110256	0.001285	-0.63053	down
6.530394	7.401164	8.072628	10.75342	10.64457	0.001299	0.499537	up
17.13231	18.164	18.58932	33.06222	26.2168	0.001329	0.572767	up
2.761204	2.693197	1.579269	1.391147	1.340882	0.001331	-0.57204	down
19.08173	18.24947	13.31998	8.992603	10.84767	0.00134	-0.54594	down
0.248461	0.217999	0.393925	0.866166	0.954744	0.001341	0.735876	up
0.121927	0.021876	0.291871	0.428111	0.366945	0.001342	0.685525	up
2.829374	2.757166	3.176001	4.924025	4.056746	0.001348	0.559205	up
3.214096	4.428586	1.354167	0.269064	0.866014	0.001355	-0.71748	down
3.831831	3.322219	2.326939	2.437925	1.978621	0.001374	-0.52558	down
6.313579	6.087803	7.1793	19.88412	9.246632	0.001381	0.548459	up
21.29193	21.20956	23.55474	30.02664	27.84378	0.001393	0.477701	up
86.80722	98.78352	63.66848	59.81095	58.56657	0.001428	-0.65945	down
6.798148	8.070155	9.222028	11.99054	12.67651	0.001479	0.570499	up
53.44578	52.91133	62.31857	63.30637	65.8384	0.001485	0.341954	up
16.03844	17.52747	17.34132	25.16213	29.46757	0.001512	0.551638	up
14.3765	14.89918	10.64088	9.28572	8.701054	0.001512	-0.46851	down
13.70178	10.96598	15.02469	21.63134	16.12851	0.001532	0.56326	up
5.776571	6.203043	3.483166	4.064043	4.299051	0.001541	-0.48259	down
165.9033	173.5041	105.7581	78.56128	109.1408	0.001547	-0.5176	down
4.087424	4.503067	4.516892	7.280383	8.35496	0.001572	0.50638	up
9.128161	8.45027	12.90356	12.87549	11.31623	0.001576	0.529659	up
18.69076	21.4018	20.51024	40.39868	35.52366	0.001582	0.602098	up
1.916553	1.929006	1.078324	1.189207	1.293989	0.001585	-0.50886	down
0.90815	0.780642	1.029257	1.822507	1.858615	0.001591	0.673544	up
1.916019	1.869326	1.632217	3.9496	3.988525	0.001603	0.697601	up
1.500116	0.967036	2.147314	2.666696	2.753688	0.001608	0.69638	up
8.020054	7.982082	12.61919	12.75407	9.579856	0.00161	0.454653	up
5.209546	5.694777	3.010247	4.035043	2.994694	0.001616	-0.6074	down
22.35649	18.58671	33.16027	31.7254	24.63424	0.001638	0.542746	up
6.066573	6.706006	3.55135	4.645067	3.705295	0.001659	-0.47639	down
45.84448	52.5508	67.09457	75.0928	81.77703	0.001662	0.483826	up
4.027735	3.275623	6.414884	5.142388	7.953407	0.001665	0.610356	up
0.374283	0.279317	0.499191	0.554067	0.697714	0.001671	0.677768	up
3.435886	3.480134	0.682204	0.559908	1.677961	0.001674	-0.72143	down
3.549589	7.562042	11.01413	6.919427	8.321408	0.001679	0.703598	up
2.858381	2.671132	3.297149	3.476446	4.335585	0.001692	0.461705	up
29.15144	28.73134	19.61369	20.9936	17.56783	0.001697	-0.44659	down
1.72467	0.968137	0.758404	3.57949	3.754907	0.001697	0.720828	up
5.034573	6.000488	4.041693	4.377003	4.469133	0.001711	-0.38967	down
84.86802	73.15632	76.22686	146.8751	119.2358	0.001725	0.503008	up
3.254908	3.434875	3.986481	5.465909	5.700249	0.001737	0.514267	up
4.959974	4.490019	1.473496	2.868077	2.731817	0.001774	-0.62835	down

9.236702	9.601249	10.80127	22.90978	15.57396	0.001792	0.637402	up
4.748662	4.664977	6.854596	7.793055	6.234445	0.00183	0.550954	up
58.75459	72.76887	43.61504	41.02922	51.47524	0.001837	-0.3813	down
4.842011	4.746223	5.524769	6.990817	6.569485	0.001865	0.421131	up
30.44816	31.18688	23.4547	20.62038	39.52393	0.001869	0.522998	up
4.871334	7.371255	8.605802	8.371987	8.612511	0.001872	0.509571	up
25.04512	25.34039	42.95162	29.49132	36.62408	0.001875	0.523335	up
0.364302	0.517522	0.143342	0.154903	0.08816	0.001892	-0.71106	down
25.52544	24.83082	17.5576	11.5835	11.53435	0.001911	-0.58471	down
26.56566	31.31776	2.797198	6.222167	0.787169	0.00193	-0.56391	down
2.010182	1.86899	0.77886	0.852991	0.259808	0.001939	-0.71246	down
5.554848	5.830777	3.441899	4.164621	3.507561	0.001947	-0.47379	down
1.455607	1.803695	0.604287	1.205838	0.924895	0.001964	-0.65934	down
300.881	320.8953	342.0308	461.7845	448.1159	0.001966	0.436376	up
22.42413	21.25196	29.08539	27.91592	28.31023	0.00197	0.414859	up
1.24452	1.228485	1.751272	2.254894	1.938618	0.00198	0.574699	up
5.508752	4.919178	6.739391	13.25327	8.601884	0.002041	0.624257	up
3.781182	2.804742	4.048028	2.940475	3.243408	0.002065	0.536632	up
6.493817	5.325369	3.873069	1.034495	1.677616	0.002085	-0.70554	down
19.97342	19.70814	12.552	14.04035	14.46498	0.002096	-0.36963	down
15.77438	15.80411	17.37126	24.64852	26.99918	0.002166	0.49742	up
3.036722	3.078377	2.194835	1.853604	1.852357	0.002168	-0.54663	down
6.416516	5.610231	4.379372	2.704792	3.167653	0.002177	-0.5715	down
3.930119	3.559102	3.602604	8.766785	6.777068	0.002204	0.666313	up
2.88605	4.143862	5.682454	4.673529	6.32766	0.002218	0.589344	up
5.756835	5.395524	3.130102	4.441833	3.6195	0.002276	-0.49541	down
33.2372	36.7221	38.80028	61.8901	50.44056	0.002286	0.510539	up
167.7311	179.4016	224.3532	259.6936	195.7301	0.002309	0.453475	up
40.50402	36.83663	53.22681	60.12247	43.33181	0.002335	0.487046	up
15.97686	15.39235	10.26994	11.71881	10.98441	0.00236	-0.41747	down
14.72114	14.00472	11.29847	38.98874	41.82514	0.002383	0.690717	up
7.103593	7.256832	5.032159	2.745593	3.312104	0.002387	-0.60245	down
8.66024	7.64811	9.450625	11.51315	11.30859	0.002391	0.452468	up
1.414811	1.89572	0.900986	0.560409	0.723472	0.002401	-0.65346	down
14.37315	13.81855	7.831267	7.159712	9.621934	0.002403	-0.50193	down
1.237062	1.324422	1.223951	0.795216	1.046862	0.002434	-0.49789	down
11.01581	10.70694	14.21779	14.1016	13.77874	0.002449	0.395873	up
0.762466	0.654065	0.323342	0.336388	0.235572	0.002455	-0.6901	down
173.9549	197.8031	248.8975	294.2851	242.6252	0.002464	0.447662	up
35.29756	32.47528	37.54338	62.28312	50.29944	0.002467	0.514384	up
2.665729	2.369993	0.378998	0.870747	1.027017	0.002468	-0.69358	down
79.45911	74.7742	61.01264	39.57015	48.1047	0.002501	-0.5463	down
5.928322	5.756494	4.105365	2.682725	3.533708	0.002523	-0.53564	down
1.071224	1.163893	1.108396	2.420888	2.541696	0.00253	0.662717	up
34.28443	33.1233	23.18425	11.8159	11.13549	0.002536	-0.64638	down
16.8989	14.84935	16.41463	23.55099	24.88063	0.002572	0.487826	up
0.568468	0.770044	0.229119	0.308573	0.469514	0.002583	-0.64884	down
0.754762	0.501664	0.309998	0.189008	0.12549	0.002609	-0.69016	down
181.7205	171.5572	221.2591	244.8799	220.0871	0.002611	0.429305	up
0.574405	0.601261	0.946905	1.258919	1.439151	0.002648	0.624625	up

3.198689	3.602068	1.729033	2.246891	2.345111	0.002649	-0.47017	down
0.403162	0.378617	0.642638	0.630816	0.577737	0.002671	0.531159	up
6.278224	5.643201	4.344691	2.969576	3.688766	0.002691	-0.59183	down
15.52222	15.5075	19.23014	20.23167	19.94083	0.002692	0.396352	up
1.255556	1.262685	2.200808	1.840177	2.101538	0.002752	0.566149	up
1445.976	1717.346	1685.353	2660.957	2328.548	0.002791	0.392797	up
16.20749	14.36993	22.82116	19.77642	23.59677	0.002794	0.439593	up
1.640846	1.977988	2.493735	2.316028	3.175537	0.002823	0.622049	up
26.64188	26.14918	28.44319	33.13368	34.26112	0.002827	0.368379	up
31.57443	35.29206	21.63674	24.13845	24.60625	0.002846	-0.56146	down
7.40924	7.280897	8.045299	12.44404	10.45714	0.002858	0.546823	up
142.4039	154.4192	111.314	112.6166	115.0727	0.00286	-0.33674	down
22.32217	28.70573	25.26541	50.51645	39.63994	0.002878	0.596867	up
38.16252	42.49134	39.42656	59.43052	63.46441	0.002912	0.509094	up
18.8551	20.97173	17.11867	41.28418	32.50041	0.002921	0.623278	up
6.488162	5.917742	4.068412	4.325658	4.913996	0.002925	-0.3658	down
5.601954	6.175937	3.541705	3.67422	4.189361	0.00295	-0.42255	down
24.18692	24.62757	22.74031	51.7969	44.65672	0.002998	0.620608	up
2.704822	2.419395	1.909738	1.204949	1.090783	0.003027	-0.63097	down
0.549791	0.584164	0.12828	0.253091	0.347664	0.003074	-0.68048	down
9.476153	9.653024	10.02441	18.68787	16.59321	0.003075	0.561225	up
2.146566	1.877628	2.477959	3.72159	3.297534	0.003108	0.556403	up
5.459713	4.919447	3.949206	2.67452	2.385346	0.003115	-0.56325	down
2.258774	2.028623	1.436768	1.248841	1.20323	0.003121	-0.50218	down
20.85507	19.92227	13.46292	13.66357	13.84765	0.003124	-0.39184	down
29.40226	30.80518	27.63088	31.68407	51.09601	0.003149	0.469195	up
17.15795	15.88877	10.17373	8.364465	12.0873	0.00317	-0.43599	down
10.63948	10.5592	7.065516	6.040521	7.305836	0.003198	-0.39185	down
1.282635	2.114065	0.648942	0.388197	0.809923	0.003213	-0.66548	down
2.010435	2.067254	1.070443	0.653083	1.146569	0.003222	-0.66537	down
4.916317	4.78482	3.879108	3.560924	3.947891	0.003234	-0.44444	down
2.965626	2.43281	1.712478	1.89356	2.100843	0.003267	-0.57223	down
28.50637	41.96046	30.70541	31.46607	21.6153	0.00327	-0.51077	down
19.35577	18.6084	24.28148	22.02717	29.27285	0.003297	0.490641	up
1.006724	1.274652	0.611571	0.613863	0.782156	0.003308	-0.61064	down
45.98035	38.80865	31.46299	112.327	126.88	0.003343	0.666665	up
10.53378	11.38025	13.38615	12.81735	12.93688	0.003357	0.349486	up
322.8873	373.1546	536.2529	520.488	479.7313	0.003362	0.409604	up
5.474444	7.130689	3.263352	3.767749	4.346093	0.003376	-0.49825	down
34.75297	30.38014	24.8187	22.49127	22.64593	0.003396	-0.34725	down
16.66482	16.69069	11.74275	8.864856	8.904158	0.00342	-0.49654	down
5.927182	6.267428	3.7762	4.568686	4.59764	0.003424	-0.43706	down
4.663978	3.995609	5.14751	9.873601	7.326992	0.003443	0.584152	up
4.70316	4.084744	4.812692	6.963758	6.381628	0.003454	0.461947	up
8.92453	8.269459	9.653325	12.26014	12.31367	0.003459	0.416727	up
45.87657	48.36949	54.47676	56.11331	74.91176	0.003475	0.34308	up
0.906124	0.900623	1.480766	1.193938	1.498779	0.003497	0.602175	up
5.184893	5.071674	5.371542	9.011604	8.772277	0.003501	0.486791	up
18.71286	24.78583	12.58442	18.38327	15.24051	0.00351	-0.44119	down
1.339287	0.746199	1.970065	1.777367	2.167301	0.003515	0.667399	up

39.04086	44.28593	29.62601	22.3413	23.35278	0.003565	-0.46362	down
1.74083	1.934066	2.195893	3.445387	4.214576	0.003618	0.636766	up
0.232153	0.169173	0.040673	0.081542	0.082887	0.003628	-0.66865	down
12.38127	11.04552	7.894873	7.535489	8.748516	0.003643	-0.33895	down
35.33512	34.79537	23.71971	18.82624	21.58925	0.003645	-0.4935	down
28.39669	29.12462	20.57006	18.98913	20.89608	0.003656	-0.37291	down
14.7192	17.83152	17.91029	36.14915	31.16784	0.003662	0.584042	up
51.76193	48.94171	54.81175	59.0528	65.1892	0.003675	0.341726	up
14.35047	13.71916	14.20858	19.88026	20.50188	0.00369	0.475463	up
25.03047	22.5305	17.92841	14.11524	16.70128	0.003735	-0.38741	down
3.441507	3.678011	2.22149	2.406025	2.63762	0.003744	-0.42235	down
60.33448	56.66926	46.69593	31.07826	34.53911	0.003788	-0.51289	down
3.10375	3.245168	1.83881	2.088657	2.424983	0.003794	-0.52939	down
15.51658	17.61502	11.44329	10.24341	10.47707	0.003832	-0.41996	down
2.414169	1.819441	1.436289	1.19809	1.06937	0.003838	-0.54444	down
0.295037	0.262939	0.432854	0.530652	0.492302	0.003883	0.608497	up
3.406445	3.28219	3.148184	7.133808	6.410987	0.003919	0.589189	up
11.89551	11.56156	11.3422	20.77781	19.00483	0.003961	0.529763	up
7.907431	8.384883	12.06371	9.785899	13.45094	0.004019	0.460648	up
988.8516	1004.241	1032.356	1439.837	1423.364	0.004031	0.417858	up
30.6651	30.08991	32.38168	36.51254	40.52833	0.004047	0.382598	up
110.6578	108.1883	125.2526	160.5331	131.6856	0.004055	0.399136	up
0.72272	0.940641	1.1354	1.167851	1.394937	0.004067	0.533182	up
3.509698	3.307175	3.15687	10.75398	5.634243	0.004074	0.649483	up
3.55498	2.287928	5.988714	4.197033	4.301322	0.00408	0.56578	up
10.17628	10.72393	13.99311	12.76898	13.15787	0.004088	0.402479	up
7.446478	6.073078	10.09983	9.011413	12.26063	0.004099	0.561744	up
27.00361	31.60183	36.22828	36.07187	43.87111	0.004103	0.403537	up
0.992871	1.320422	0.554609	0.703937	0.673428	0.004117	-0.59221	down
0.180119	0.13929	0.420257	0.376452	0.279664	0.004128	0.658855	up
1.000912	1.110603	1.573434	1.814852	1.904754	0.004164	0.561041	up
18.0062	18.69622	21.63174	31.68857	22.85276	0.0042	0.46939	up
11.91961	10.2172	14.3915	12.566	15.59344	0.004211	0.470427	up
22.97848	25.832	18.61636	17.68143	16.30936	0.004238	-0.40493	down
0.739965	0.630421	0.617947	0.375096	0.13507	0.004253	-0.62523	down
0.396705	0.562703	0.331196	0.226209	0.302204	0.004281	-0.57912	down
58.13352	51.7675	98.34182	62.52278	82.67814	0.004292	0.503709	up
1.129732	0.595617	0.486502	0.421351	0.483431	0.0043	-0.59205	down
37.36446	36.08556	23.57882	22.64783	26.33507	0.0043	-0.39306	down
17.52535	18.14296	13.62906	12.5674	12.11798	0.004314	-0.40221	down
3.333747	4.143731	0.979187	2.145829	2.099723	0.004325	-0.61091	down
2.978198	2.94054	1.598237	1.7529	1.525549	0.00434	-0.48539	down
19.96313	21.08407	25.56551	31.01289	24.26231	0.004355	0.424474	up
17.74509	16.36676	13.37325	9.673382	9.456366	0.004397	-0.50527	down
2.811632	2.365579	4.304436	3.153756	3.655956	0.004423	0.481891	up
28.42677	31.60469	27.17959	81.68452	67.76986	0.004423	0.612544	up
0.532851	0.593741	0.821565	1.179878	1.775831	0.004457	0.652508	up
0.404157	0.900236	0.600108	1.634126	1.935866	0.004486	0.6484	up
41.60375	38.58876	17.45222	28.33034	23.34608	0.004493	-0.50367	down
3.188886	3.811359	2.650419	2.212286	1.613705	0.004504	-0.53651	down

0.17434	0.630631	0.810882	1.101431	1.348329	0.004511	0.648761	up
9.069934	9.743259	5.663704	6.474035	7.379283	0.004519	-0.38827	down
26.7193	22.68781	25.02619	51.97787	40.79888	0.004525	0.554675	up
1.609535	1.858488	2.047661	2.262474	3.137845	0.004628	0.473486	up
22.70585	20.80999	23.87019	28.15933	29.53425	0.004629	0.362454	up
3.122543	3.863122	4.121532	5.312581	5.528972	0.004638	0.444274	up
10.1072	10.23268	15.98965	16.1073	12.71471	0.004648	0.466327	up
19.77288	22.69806	25.19646	26.54426	31.01959	0.004665	0.375913	up
1.107968	2.211648	1.524288	10.31853	4.612254	0.004665	0.63755	up
7.31359	7.770416	7.969173	10.56572	12.81828	0.004679	0.466718	up
0.307048	0.289917	0.330162	0.641814	0.82445	0.0047	0.637011	up
3.389102	4.24499	2.95034	0.84537	1.543545	0.004738	-0.64465	down
8.151908	7.866008	5.651707	4.331969	4.438652	0.00476	-0.47314	down
10.04779	9.526329	15.66325	14.78202	12.2371	0.004768	0.43951	up
15.89567	13.61918	9.829495	5.565339	6.442566	0.004806	-0.58353	down
1.965825	1.780656	0.905411	1.181614	0.996905	0.004844	-0.59578	down
1.645372	1.876199	2.321587	2.460765	2.505688	0.004894	0.542183	up
4.046545	3.171148	4.630243	5.497077	6.21756	0.004903	0.476719	up
37.07404	37.33942	57.08103	46.27593	44.33349	0.004924	0.436373	up
0.752574	1.431193	2.227142	1.383388	2.947811	0.004925	0.630635	up
4.25036	3.318558	5.573507	4.93489	5.393994	0.004969	0.475598	up
2.60149	2.679418	2.83915	4.594681	4.749102	0.00498	0.501553	up
12.2531	12.55448	2.240053	9.48991	1.760768	0.004981	-0.63933	down
3.465922	4.414151	2.086369	2.888329	1.63704	0.004996	-0.58105	down
9.361813	10.00099	8.512342	19.68631	14.00427	0.005006	0.618484	up
6.156079	6.386389	4.018908	4.498348	5.031837	0.005008	-0.38032	down
70.78708	66.82846	53.23012	34.57404	33.2379	0.005022	-0.52291	down
0.925092	0.822702	0.492751	0.383371	0.605983	0.005024	-0.54934	down
1.962469	2.042395	2.087279	3.626729	3.427953	0.005037	0.551985	up
3.007779	4.428201	2.630424	12.0782	11.10644	0.00505	0.644479	up
26.50702	26.54467	32.3253	29.40048	32.10741	0.005058	0.345218	up
233.0585	255.4028	186.1444	181.4001	163.9653	0.005074	-0.40112	down
0.99657	0.501319	0.504008	0.999172	0.874112	0.00508	0.643097	up
2.507467	2.645538	1.560319	0.535549	0.532426	0.005097	-0.64375	down
12.9264	14.23547	11.76143	35.1843	27.8865	0.005126	0.616304	up
10.20512	9.944357	11.75032	12.84833	12.6626	0.00519	0.396244	up
1.645789	1.166159	0.242728	0.42825	0.646655	0.005238	-0.64123	down
3.719748	3.779201	4.33524	4.470558	5.958569	0.005303	0.418836	up
34.03389	36.63821	25.33979	23.3993	25.02365	0.005306	-0.35866	down
72.28685	86.41473	44.21632	40.51138	57.60009	0.005318	-0.4688	down
1.037931	1.939266	1.998962	3.815716	3.994337	0.005322	0.638956	up
3.783127	6.031929	18.64093	5.653972	8.717579	0.005339	0.629993	up
24.65243	23.96217	17.1524	19.11436	18.02907	0.005343	-0.43093	down
3.684616	3.516115	3.43665	7.328599	6.040393	0.005391	0.5603	up
6.888332	6.648939	3.741731	5.531945	4.103051	0.005405	-0.55218	down
14.1682	14.6619	4.702805	11.33109	9.345651	0.005413	-0.5872	down
1.008723	0.407801	0.232619	0.064518	0.080228	0.005501	-0.62937	down
17.15149	17.22012	17.2771	32.88734	30.55839	0.005519	0.539019	up
196.0165	201.5747	210.435	250.1544	249.2511	0.005553	0.332408	up
2.436901	3.126432	4.149415	8.8254	2.876385	0.005599	0.629289	up

1.031725	1.815531	1.256114	5.362471	5.599371	0.005626	0.633798	up
1151.963	1148.148	1373.237	1389.72	1455.831	0.00563	0.293091	up
1350.437	1333.876	1598.34	2037.945	1976.844	0.005668	0.402417	up
22.22054	24.0373	19.86577	46.95478	49.16758	0.005679	0.580782	up
26.60351	27.46724	29.1085	31.63356	37.05497	0.005693	0.341018	up
1.183077	1.419349	0.627473	0.370055	0.336591	0.005716	-0.63448	down
0.53299	0.927705	1.369154	1.353412	1.223544	0.005758	0.629265	up
2.314125	2.663612	1.020664	1.530986	1.957774	0.005795	-0.55566	down
4.826577	4.754011	5.90845	5.996991	13.66678	0.005864	0.588813	up
388.5716	390.8242	511.8139	475.022	506.0113	0.005946	0.341558	up
5.166319	4.837373	4.253309	9.520619	9.365887	0.006011	0.573017	up
1.999669	2.3389	1.394933	0.989134	0.494043	0.006086	-0.57478	down
2.502812	1.7912	1.190676	1.275245	1.537604	0.006089	-0.45764	down
5.622811	5.437127	8.372828	8.019848	7.084185	0.006103	0.462365	up
182.0451	164.3292	241.5034	232.3461	194.5579	0.006109	0.407918	up
1.211994	1.830209	0.526911	0.404828	0.767152	0.006119	-0.62075	down
9.123449	7.716365	8.316583	12.13432	14.48738	0.006142	0.482605	up
1.880123	1.812022	1.900275	5.917783	5.86717	0.006151	0.628517	up
2.91247	2.948505	2.726248	5.889748	4.920609	0.006203	0.562885	up
9.767881	11.23476	8.068197	5.736266	6.102761	0.006222	-0.49572	down
2.767421	3.584538	4.339109	5.804997	10.06907	0.006239	0.548483	up
139.7742	153.3056	94.50662	98.82809	110.9934	0.00626	-0.35037	down
0.084875	0.097906	0.102084	0.110793	0.506192	0.006283	0.619018	up
23.30515	19.67559	43.13657	31.93097	29.38742	0.006337	0.435771	up
18.7906	18.88479	22.51175	23.39912	24.11479	0.006398	0.333978	up
1.589527	2.018318	2.208564	2.626802	2.240375	0.006472	0.508983	up
134.7934	125.8513	101.5198	82.55433	96.13803	0.006522	-0.37439	down
5.783065	7.627657	5.461948	2.580754	3.122306	0.006533	-0.59145	down
15.49595	14.54724	16.77196	21.36645	19.26143	0.00654	0.386526	up
23.62263	20.11199	21.30482	33.49694	40.56186	0.00657	0.532528	up
13.87204	16.32135	11.6403	10.32818	9.754455	0.006574	-0.4381	down
14.07078	12.66889	18.74629	19.19118	14.98221	0.006576	0.370344	up
10.69589	6.069582	7.314972	3.613679	3.087479	0.006618	-0.39091	down
4.513055	5.209129	5.247092	9.581303	7.176605	0.006625	0.584317	up
1.606952	2.335698	1.908765	5.338258	4.453793	0.006636	0.623322	up
14.05268	15.60702	6.342863	11.16625	9.585449	0.006638	-0.5267	down
0.21508	0.181649	0.165731	0.591233	0.761072	0.006721	0.609961	up
11.6926	13.93662	4.442928	5.80989	10.14144	0.00675	-0.55013	down
7.43683	8.238384	5.855439	5.788013	4.969908	0.006761	-0.42507	down
12.15269	12.14142	12.76262	15.06743	16.09968	0.00682	0.356806	up
11.81187	11.4709	7.921302	7.725759	5.051653	0.006846	-0.4795	down
2.720902	2.62341	2.021091	0.549641	0.446077	0.006854	-0.60733	down
6.650047	6.389478	4.424006	5.042503	4.829713	0.006873	-0.37122	down
8.614998	8.251751	9.186498	13.4342	11.05682	0.006886	0.431198	up
0.12768	0.101616	0.129801	0.251434	0.364705	0.00691	0.617425	up
4.584116	4.234656	5.248736	4.996563	5.494244	0.006912	0.328368	up
8.153851	7.260404	18.36217	8.934121	11.0396	0.006917	0.564337	up
28.63737	29.91173	34.02692	33.95389	28.93705	0.006957	0.377285	up
2.342627	2.195176	2.395263	3.080773	3.110244	0.006957	0.390273	up
8.186355	11.07107	6.556219	24.85586	23.89507	0.007009	0.611333	up

5.537807	5.537696	2.630344	4.198978	3.465679	0.007009	-0.44957	down
6.474332	7.996271	6.094941	3.804932	3.874484	0.007018	-0.4319	down
1495.8	1675.147	1602.902	2490.326	2186.777	0.007056	0.433397	up
0.916529	0.732777	0.361669	0.219715	0.315318	0.007098	-0.61763	down
14.17912	13.34665	10.56422	8.525242	9.629347	0.007106	-0.39403	down
15.29761	15.18242	16.46529	19.99748	18.84485	0.007109	0.356799	up
236.9414	345.7525	91.38837	272.6091	135.9921	0.00713	-0.61209	down
16.18431	21.75692	22.41153	22.90832	27.2196	0.007147	0.436973	up
1.072082	1.337213	1.010788	2.731405	2.502459	0.007195	0.59167	up
18.30188	16.39178	22.4307	20.20802	23.61723	0.007222	0.398762	up
8.154542	7.637471	12.96511	13.21013	11.9202	0.007259	0.45318	up
2.288083	3.052512	2.721975	4.312656	4.43974	0.007312	0.556363	up
49.43849	48.32529	35.21692	23.42312	38.62967	0.007342	-0.47947	down
55.36935	52.64986	32.9935	37.94738	45.39422	0.007367	-0.37937	down
3.921163	6.555422	2.206065	2.898329	2.790079	0.00737	-0.56706	down
8.43468	8.699109	6.430885	6.138551	5.408768	0.007372	-0.39263	down
1.702585	1.445183	0.409506	0.642963	0.739356	0.00738	-0.60949	down
6.246848	5.940024	4.546031	4.044349	4.203423	0.00745	-0.40012	down
55.04272	60.9814	58.60045	71.59305	70.19585	0.007511	0.347653	up
9.398686	9.534164	10.69522	13.33954	11.57301	0.007525	0.366574	up
44.09352	42.52096	53.20363	53.26274	50.51213	0.007561	0.35786	up
62.6844	58.50938	55.92241	33.00348	27.43381	0.007621	-0.60063	down
43.09573	37.15087	53.81678	93.9702	58.03567	0.007634	0.529005	up
0.175917	0.369128	0.477076	0.702665	0.988324	0.007636	0.612552	up
6.962256	5.901852	7.249733	9.568027	8.423892	0.007665	0.406037	up
4.853628	4.06431	4.026462	2.483412	2.508816	0.00774	-0.34721	down
23.25267	22.7415	15.03529	16.33792	19.97874	0.007763	-0.34828	down
25.51372	27.52285	22.98801	62.8102	57.54805	0.007846	0.574119	up
6.956147	8.367208	4.279748	4.421291	4.587613	0.007854	-0.44753	down
6.663278	6.795394	4.301187	19.49155	16.50547	0.007858	0.608988	up
19.57093	17.2326	13.69543	11.66662	8.966917	0.007894	-0.47567	down
160.4009	163.1486	175.0423	192.401	148.295	0.007922	0.312004	up
0.761598	0.708233	0.238108	0.056502	0.392899	0.007944	-0.58401	down
8.194075	8.651554	3.958347	5.570277	6.7765	0.007945	-0.44576	down
4.827287	4.24106	2.813953	3.073744	3.703098	0.007975	-0.39325	down
19.3663	22.79083	16.73654	57.36677	45.69452	0.00801	0.592653	up
26.48872	24.48006	20.40596	14.33986	14.66589	0.008028	-0.46563	down
1.116985	0.930861	0.3768	0.644978	0.600439	0.008087	-0.56833	down
5.185291	4.540458	3.328657	3.161222	3.780471	0.008136	-0.40604	down
32.29543	41.3485	24.27932	126.2587	73.45676	0.008243	0.606584	up
24.91825	24.49666	38.60213	24.34127	35.67694	0.00826	0.508833	up
2.603079	2.506453	6.304018	3.51675	3.584605	0.008343	0.545239	up
1.750664	1.142049	0.515024	0.665852	0.821395	0.008417	-0.59512	down
32.00422	32.03196	35.54457	36.96116	38.68026	0.008425	0.32336	up
19.37269	19.83996	24.50173	25.86274	22.99683	0.008439	0.412517	up
0.106539	0.139415	0.046116	0.023435	0.046087	0.008454	-0.6051	down
10.3404	10.49529	7.680261	7.002973	8.034981	0.00846	-0.34594	down
1.082712	1.322222	0.995999	3.781669	2.341583	0.008462	0.60488	up
0.427765	0.340907	0.208257	0.257909	0.202708	0.008468	-0.56083	down
7.371934	6.952064	9.694588	8.061026	9.254088	0.008481	0.464999	up

34.61095	34.01675	26.69289	17.11456	14.92425	0.008539	-0.52446	down
0.556265	0.635398	0.982475	1.160285	1.092557	0.008572	0.573051	up
5.206961	5.028837	2.728017	2.706793	3.693459	0.008575	-0.4332	down
17.30092	21.29435	11.69641	9.361795	9.073512	0.008613	-0.50454	down
0.799934	0.607399	0.861519	1.453283	1.796501	0.008617	0.600621	up
73.33856	76.63361	60.03685	37.22909	42.14	0.008619	-0.47906	down
1.805852	1.175675	1.516503	3.380683	2.757762	0.008633	0.599013	up
8.802902	9.638575	10.02659	11.602	13.32036	0.008678	0.454739	up
1.812294	2.035816	0.797375	0.849659	0.902237	0.008702	-0.58701	down
14.22707	15.4224	11.14155	9.286764	9.151052	0.008715	-0.37867	down
0.956785	1.01997	0.325448	0.612482	0.604412	0.00874	-0.5719	down
14.5804	15.09446	17.98949	21.52506	16.79835	0.008765	0.469138	up
94.99513	88.35866	72.99762	55.21158	71.13135	0.008773	-0.39791	down
7.322209	7.807921	4.02981	4.790079	5.966547	0.00889	-0.41335	down
1.022701	1.184333	0.386818	0.542459	0.725292	0.008895	-0.58627	down
23.07511	23.23618	24.47488	29.54843	32.05719	0.00893	0.341061	up
2.160384	3.101037	3.882187	3.354185	3.522241	0.008933	0.461577	up
31.43103	32.64391	20.33184	21.80444	23.3988	0.008934	-0.35122	down
18.68469	18.62884	21.48521	20.86271	23.74998	0.008946	0.309518	up
13.45526	13.76347	5.784332	11.4598	10.6582	0.00896	-0.56317	down
1.202214	1.67949	2.101388	1.829849	2.052661	0.008994	0.489922	up
4.243069	3.959639	2.648807	2.465837	3.520052	0.009003	-0.42123	down
4.625699	3.349288	5.431296	4.838518	5.520802	0.009071	0.386246	up
73.1886	77.59741	100.4057	84.69374	90.87899	0.009088	0.352946	up
1.032793	1.196722	1.365431	1.483241	1.779003	0.009181	0.495353	up
0.254174	0.235593	0.399593	0.434225	1.184831	0.00919	0.598216	up
16.19074	19.07955	13.56198	70.3136	35.86258	0.009249	0.596964	up
3069.197	3357.488	3506.699	4109.461	3908.23	0.009277	0.311805	up
14.75032	15.40608	7.06803	9.683681	8.960011	0.009279	-0.46265	down
26.24125	29.28554	32.32359	37.79122	31.43102	0.009294	0.39134	up
19.02325	18.36898	14.26886	11.67494	13.54088	0.009309	-0.37717	down
31.50055	30.23679	34.01389	34.51947	37.95758	0.009371	0.318357	up
10.19323	9.325591	7.145378	7.036435	6.688966	0.009385	-0.34154	down
1.197818	1.061811	1.254766	1.731935	2.105182	0.009392	0.524371	up
15.04356	19.36372	18.17444	33.05485	26.36147	0.009397	0.492357	up
2.225525	2.372457	1.027794	1.247947	1.542654	0.009448	-0.45263	down
1.666898	1.711414	1.761944	6.549262	4.350691	0.009457	0.596199	up
1.381775	1.778309	0.580342	0.945386	0.861624	0.009457	-0.53987	down
16.10212	15.50248	12.21051	10.81375	11.2092	0.009463	-0.34334	down
1143.029	1206.697	1175.07	1772.088	1638.468	0.009531	0.394799	up
17.48805	16.19464	13.82541	14.66618	14.00927	0.009554	-0.3305	down
14.84099	15.62852	14.56142	49.01891	27.03581	0.009638	0.581638	up
11.35117	12.85262	14.44574	22.82887	19.44196	0.009659	0.470921	up
14.83549	13.06898	19.12436	19.8006	17.05466	0.009668	0.397363	up
6.613449	6.822604	3.922605	4.717538	4.998303	0.009755	-0.38392	down
2.492361	2.875545	0.469567	1.732318	1.152269	0.009792	-0.58874	down
57.91159	55.50107	76.94988	77.79417	64.5839	0.009822	0.369336	up
53.29249	51.39008	44.94539	70.562	71.91305	0.009859	0.433656	up
7.270932	7.579532	10.88541	8.767997	9.996307	0.009869	0.376222	up
1.206497	2.288403	3.766731	3.120317	2.796063	0.00992	0.572819	up

15.57618	14.91843	16.2705	17.67722	19.15021	0.009936	0.328572	up
12.92333	15.55331	9.865628	2.892481	4.567746	0.009999	-0.58788	down
3.943621	5.238649	3.436657	79.62441	80.10706	0.010081	0.431158	up
93.24849	128.9244	238.2476	253.6143	293.6409	0.010098	0.367409	up
12.64561	12.61559	8.579197	9.147063	10.52463	0.01024	-0.32254	down
56.87294	58.89934	34.00918	46.87163	41.18965	0.010255	-0.36601	down
39.33557	39.81431	29.9391	25.5666	30.80592	0.010321	-0.32073	down
16.47845	14.99212	10.18164	8.743099	11.57807	0.01038	-0.3786	down
37.45155	37.49667	31.08774	22.28404	26.68514	0.01041	-0.43605	down
11.8425	12.50982	17.42713	13.9794	14.52618	0.010413	0.38785	up
0.3745	0.602614	0.202276	1.45473	1.142287	0.010421	0.573597	up
0.496785	0.636974	0.381922	0.121867	0.214796	0.010423	-0.58561	down
10.2623	7.178466	5.98189	5.417337	5.694543	0.010476	-0.55385	down
34.12664	34.11197	28.98115	70.75779	58.36413	0.010607	0.527631	up
0.980244	0.738583	0.266215	0.497248	0.423033	0.010706	-0.58505	down
7.021675	8.504587	9.577834	10.69429	9.222986	0.010717	0.43222	up
78.52581	79.35672	59.57525	55.715	51.7893	0.010747	-0.34343	down
21.60929	20.57912	15.50759	15.48896	15.47711	0.010785	-0.29864	down
10.43743	9.023432	7.07995	6.598761	7.523915	0.010828	-0.3936	down
6.476474	7.950729	10.09341	16.12152	15.30292	0.010895	0.501625	up
8.632785	9.753527	6.038005	5.535977	6.100708	0.010909	-0.38875	down
0.409586	0.336366	0.389347	0.860698	1.030796	0.010921	0.579789	up
109.9556	106.0755	73.31362	64.50055	86.29767	0.010958	-0.36111	down
34.0268	34.70129	25.97857	20.18385	24.13746	0.010964	-0.35863	down
6.976945	7.399037	4.600199	3.913312	3.419315	0.011038	-0.46549	down
1.102553	1.388642	2.353625	1.837894	2.296853	0.011145	0.493606	up
85.96914	91.33112	119.5727	116.9296	107.8791	0.011173	0.354241	up
102.67	103.4385	66.15748	54.51902	82.44403	0.011245	-0.39998	down
1264.136	1391.35	1326.005	1722.936	1703.857	0.011264	0.322422	up
0.386832	0.270407	0.508087	0.755296	1.043868	0.011265	0.576936	up
0.924607	0.704907	1.225978	1.037906	1.350204	0.011291	0.510689	up
8.059609	7.927686	6.831126	5.940263	6.942241	0.01137	-0.31518	down
0.54865	0.484021	0.259614	0.302464	0.24931	0.011446	-0.56608	down
4.411242	5.028081	2.610618	3.988915	3.644265	0.011454	-0.5211	down
33.74274	32.23828	35.69119	42.72938	40.86783	0.011466	0.353259	up
101.7706	122.7948	132.7265	155.1958	157.7132	0.011473	0.361255	up
61.4765	71.22679	92.43114	83.09737	79.36802	0.011496	0.367611	up
3.03147	3.000623	3.570664	4.366326	5.439124	0.011508	0.434705	up
3.423918	2.98373	1.609849	2.292755	2.208298	0.011517	-0.52057	down
2.402285	2.78751	1.19293	1.865901	1.632914	0.011528	-0.46401	down
65.55095	64.1607	74.66013	71.37968	77.31469	0.011534	0.285786	up
30.10294	33.49778	36.48152	47.71244	40.19958	0.0116	0.358468	up
120.5536	130.1228	94.24044	32.05636	59.11935	0.011607	-0.55698	down
8.147581	7.692553	13.14127	10.67416	11.35587	0.011644	0.429579	up
177.6778	156.9477	256.9551	196.5669	211.5238	0.011682	0.392283	up
129.5645	155.0118	197.629	207.135	162.8795	0.011703	0.400049	up
1.42268	1.584526	1.853329	2.213506	2.811348	0.011806	0.45255	up
20.94476	23.30297	22.23359	29.7201	27.66037	0.011837	0.345834	up
2.055489	1.589631	2.051116	3.100759	2.435731	0.011838	0.462225	up
18.71375	17.88897	14.63784	12.63215	12.17561	0.011852	-0.37696	down

719.4639	844.9625	647.8942	1565.474	1259.733	0.011869	0.524201	up
0.632526	0.80882	0.480448	0.319155	0.454399	0.011877	-0.53444	down
0.419811	0.3161	0.303863	0.703913	0.97632	0.011966	0.573889	up
2.900273	3.007276	2.760458	2.452582	2.810268	0.012038	-0.36135	down
9.640477	9.092024	11.00095	10.794	10.98628	0.01206	0.311081	up
0.281247	0.3267	0.157008	0.167867	0.092067	0.012106	-0.57092	down
7.110589	7.011548	8.449107	8.548716	14.45381	0.012173	0.471232	up
1464.394	1437.601	1702.095	1926.895	1780.629	0.012227	0.322347	up
47.81523	53.56886	35.16059	35.88638	28.4984	0.012246	-0.39462	down
7.802722	7.85538	8.494097	13.22236	13.45912	0.012302	0.453423	up
5.916682	5.792604	8.054652	6.703594	7.677397	0.012312	0.37004	up
0.964004	0.751859	0.794633	0.276205	0.425267	0.012343	-0.54677	down
90.62401	98.52219	101.5075	114.4796	113.0058	0.012385	0.340907	up
28.64596	30.64761	20.37346	18.13305	23.33203	0.012422	-0.35265	down
20.08828	20.34025	26.92709	28.18184	26.56552	0.012532	0.296113	up
12.81517	13.01702	11.67193	26.27962	22.52516	0.012535	0.515998	up
17.41888	18.82203	13.35323	13.71423	12.03151	0.012565	-0.36294	down
0.413354	0.510587	0.363078	0.65623	0.79974	0.012568	0.531496	up
5.68306	6.378608	7.933166	15.76332	7.468372	0.012569	0.542498	up
9.701314	9.46903	9.161861	18.36688	14.83535	0.01257	0.48847	up
11.16831	10.77537	6.568059	8.312943	8.126122	0.012586	-0.36019	down
845.4543	989.9664	935.9521	1395.889	1308.91	0.012607	0.395204	up
13.83273	12.89199	7.205646	9.908028	10.62301	0.01266	-0.38029	down
18.31244	17.49001	56.20181	20.8184	37.70161	0.012678	0.559171	up
9.138369	9.69438	6.101151	6.009793	7.195939	0.012687	-0.36097	down
0.808761	0.746413	0.825459	1.066275	1.448628	0.012751	0.482825	up
4.247887	5.743149	3.984553	6.957541	8.125932	0.012904	0.401324	up
24.69676	20.76696	19.1898	19.80416	14.61801	0.012957	-0.30911	down
21.42788	19.71168	22.43508	65.44589	34.50563	0.013053	0.532986	up
0.750099	0.715017	0	0.320743	0.080558	0.013257	-0.45362	down
1.740315	1.42236	0.653939	0.956183	0.912491	0.013263	-0.51099	down
3.325561	2.714642	1.295034	2.798527	3.424888	0.013265	0.561293	up
8.243331	8.434123	5.425015	6.954007	6.487553	0.013385	-0.35729	down
72.41787	78.54321	71.70626	109.9173	109.7891	0.013385	0.400222	up
1.349994	0.905461	1.314083	2.610214	3.030458	0.013441	0.549218	up
31.13482	34.82992	37.80229	39.71018	42.26049	0.013476	0.300557	up
34.66732	32.50477	29.43924	53.01689	70.61427	0.013495	0.496666	up
2.444773	2.92403	1.467095	2.169567	2.056916	0.013568	-0.43032	down
5.835592	5.879683	2.62664	3.113271	4.147135	0.013614	-0.44643	down
56.60314	56.17733	91.21446	65.49429	65.90877	0.013636	0.427282	up
2.814385	2.944322	4.019431	3.614679	3.990591	0.013679	0.388378	up
0.144604	0.257783	0.320039	0.448648	0.224085	0.01369	0.55477	up
25.51706	30.46695	19.30163	20.24136	19.40066	0.013818	-0.34932	down
0.800029	1.340415	2.777074	1.883363	2.772305	0.013866	0.562042	up
7.652284	10.16434	7.84998	25.12405	16.28146	0.013872	0.550553	up
1.363128	3.484004	5.716465	2.718582	4.662015	0.0139	0.562902	up
4.259041	4.45087	3.172073	2.405123	1.551714	0.013911	-0.5315	down
9.316619	9.981228	4.347406	5.583357	6.171185	0.01398	-0.45932	down
7.072752	6.58012	13.3953	13.89552	8.43591	0.014149	0.501395	up
9.048256	7.832201	4.417696	6.220194	6.56681	0.014167	-0.40868	down

15.5495	12.10023	8.793256	7.766623	10.36317	0.014218	-0.31697	down
174.8212	174.7573	129.031	122.0445	116.2954	0.014255	-0.32929	down
1.25557	0.844934	0.638112	0.599965	0.331034	0.014284	-0.56191	down
5.464894	4.495324	2.915422	4.265182	2.373563	0.014401	-0.4933	down
26.37218	27.26588	18.66058	21.34568	21.62307	0.014432	-0.29557	down
3.293303	3.752184	2.722842	2.042036	2.780671	0.014473	-0.39545	down
267.7861	236.1787	263.3704	319.1881	367.7949	0.01448	0.334899	up
62.48165	62.59434	50.72197	44.06069	47.87638	0.014521	-0.32009	down
2.913532	2.796741	2.034884	1.767359	1.595583	0.014597	-0.43665	down
24.88877	21.93443	16.15698	19.17563	14.89798	0.014607	-0.37257	down
16.02056	17.27766	24.91121	18.4224	22.05136	0.014608	0.384046	up
12.8177	11.90226	12.63142	15.85797	16.76839	0.014661	0.361971	up
13.32454	15.98836	12.18823	40.94273	24.67678	0.01471	0.547505	up
3.224666	3.336907	4.636064	3.498026	4.18823	0.014765	0.39063	up
45.14144	46.92696	33.79711	33.86006	34.38015	0.014776	-0.30181	down
246.4351	282.438	243.9676	391.6898	341.8174	0.014843	0.456067	up
15.00647	16.79845	14.62808	23.71091	27.53294	0.014852	0.444976	up
1.418391	1.104462	1.346521	1.877255	2.435737	0.014894	0.498584	up
9.64162	10.89974	8.14475	1.254928	1.50944	0.014937	-0.52675	down
2.000153	2.20407	3.12083	2.589373	2.752513	0.014993	0.475134	up
1.720197	1.8553	1.709025	3.493076	2.469463	0.015006	0.514153	up
4.802293	4.920894	2.93615	3.836316	3.596323	0.015011	-0.3535	down
3.154155	3.451395	2.773748	6.076766	6.689289	0.015011	0.511189	up
4.89377	4.660795	4.697452	6.901632	8.665915	0.01503	0.456733	up
16.31494	16.06842	6.310329	12.05692	10.67304	0.015074	-0.47194	down
31.97731	33.66813	20.52316	26.4673	25.54239	0.015128	-0.33465	down
2.412939	2.430785	2.416201	3.741375	3.733791	0.015184	0.49712	up
2.306635	2.568559	1.616829	5.102029	5.40139	0.015187	0.556654	up
7.60618	9.134556	8.375941	10.64734	17.33615	0.015191	0.506999	up
0.154383	0.136456	0.042658	0.069245	0.081476	0.015239	-0.55385	down
1.151158	0.855586	1.43434	1.411502	1.23337	0.015322	0.46903	up
331.5824	320.3214	366.9322	414.0245	382.4288	0.015357	0.287414	up
1.359171	1.444121	1.431003	2.410503	2.083021	0.015372	0.446524	up
8.404252	8.694001	9.060012	13.62398	11.79449	0.015391	0.3967	up
54.36642	57.64106	60.59523	85.6425	71.04109	0.015438	0.365478	up
155.5928	183.0349	183.6411	217.5553	195.3767	0.015454	0.353819	up
18.76326	22.25746	23.90419	29.13736	26.79994	0.015506	0.354777	up
1.00639	0.835442	0.305234	0.768248	0.573113	0.015535	-0.55621	down
0.778105	0.493927	0.164688	0.186822	0.29812	0.015577	-0.55564	down
6.679373	5.824147	8.859813	8.050799	7.986878	0.015589	0.376392	up
6.265737	6.525502	4.6875	5.085145	4.811691	0.015599	-0.31424	down
10.28731	9.993762	15.23938	13.71922	13.42083	0.015612	0.374515	up
6.933168	5.398032	4.905096	4.300999	4.144361	0.015632	-0.38163	down
0.880234	1.153982	1.413556	1.367034	1.608597	0.015682	0.456894	up
6.348796	5.706995	4.730221	2.853886	3.667812	0.015709	-0.46443	down
1.265256	0.843274	0.414607	0.469227	0.601482	0.015779	-0.55484	down
46.74424	42.31322	45.16066	53.23074	59.92424	0.015843	0.29449	up
0.198348	0.205396	0.268643	0.634447	0.821333	0.015875	0.551021	up
0.424608	1.099957	0.760209	1.702571	1.838186	0.015995	0.552457	up
24.77837	29.00819	30.19826	33.8179	33.08051	0.01602	0.414134	up

0.900102	1.020857	0.536831	0.350121	0.467545	0.016075	-0.50538	down
0.901671	1.282094	0.793085	0.285907	0.126448	0.016118	-0.51359	down
42.50989	41.65681	41.09222	14.2282	26.60774	0.016122	-0.54021	down
15.22867	13.96746	11.22123	9.015097	11.96906	0.016159	-0.34885	down
1032.32	1404.716	1200.7	1485.301	1268.603	0.016226	0.301121	up
45.10306	49.9835	38.0028	33.47491	31.3033	0.016265	-0.3677	down
0.806452	0.626401	0.851359	1.339525	1.714405	0.01627	0.539958	up
883.4797	899.035	901.9188	1340.77	1353.266	0.016288	0.382507	up
4.842537	5.386836	5.595658	9.172605	7.003926	0.016305	0.428317	up
32.48648	33.35865	28.68683	23.27774	22.64297	0.016345	-0.33803	down
1.085468	1.055901	2.29594	1.329732	1.740231	0.016367	0.543779	up
11.64481	19.07094	14.26933	28.61041	31.02446	0.016387	0.543987	up
8.032992	6.373873	7.473589	9.908187	8.810248	0.016425	0.402951	up
9.607574	9.434068	7.436538	5.585536	6.063875	0.016427	-0.39785	down
0.692374	0.546372	0.820941	0.942832	1.234187	0.016459	0.501768	up
6.629006	6.047039	4.43691	3.829687	4.435435	0.016622	-0.36001	down
7.05244	9.681507	3.311974	5.827281	3.863664	0.016629	-0.50314	down
58.8523	60.05383	50.46903	39.62151	39.61649	0.016647	-0.39004	down
1.812467	1.786059	1.674507	2.901161	2.753588	0.016669	0.462154	up
23.5371	30.20372	26.56994	32.52819	41.63965	0.01673	0.381327	up
2.977639	3.20594	1.805905	2.112174	2.608734	0.016736	-0.44131	down
0.393432	0.442514	0.134638	0.097144	0.23581	0.016745	-0.54984	down
20.6055	19.51668	31.58424	21.37338	27.98986	0.016772	0.386153	up
7.794664	7.993426	14.77113	9.752325	13.15478	0.016775	0.466068	up
7.945691	7.969097	7.982495	10.37061	13.38053	0.016795	0.423564	up
13.1144	10.88187	7.700181	4.741214	5.902051	0.016836	-0.42117	down
0.499551	0.46869	0.706354	0.554534	1.700787	0.016864	0.549316	up
48.20499	44.80081	64.29814	50.94331	49.19619	0.016983	0.299696	up
6.194261	6.836043	12.97304	7.501057	8.996396	0.016997	0.478895	up
163.9422	172.0404	150.3116	262.5821	250.5167	0.017089	0.424763	up
8.035428	7.279708	6.136673	3.363156	4.464414	0.017098	-0.46418	down
5.047922	4.727004	5.57265	5.27321	7.194754	0.017122	0.363473	up
5.489578	4.960052	6.170654	6.153858	6.858772	0.017243	0.359138	up
396.6951	419.5334	447.8307	465.0969	408.4011	0.017318	0.290751	up
22.48103	21.67486	17.25695	15.10711	17.99387	0.01733	-0.30221	down
70.71577	69.44841	64.31079	123.7685	126.4774	0.01753	0.456514	up
56.40103	59.49333	60.28921	78.2104	71.46648	0.017542	0.317587	up
24.00304	19.4833	28.03504	27.36087	29.35932	0.017556	0.342469	up
0.814379	0.821008	1.143403	1.908105	1.477996	0.017567	0.545599	up
4.35351	3.796321	7.359726	5.157482	5.524979	0.01763	0.431312	up
8.32826	7.344694	8.296666	9.148095	10.106	0.017649	0.331864	up
2.236711	2.868538	2.017416	1.065927	1.11187	0.017695	-0.5226	down
12.81914	11.52282	19.55201	14.62771	14.46703	0.017788	0.372539	up
17.90893	17.64057	21.94891	19.22965	18.79647	0.017798	0.265568	up
4.146933	3.473541	4.125824	4.828473	7.212916	0.017839	0.4773	up
4.995227	5.446715	3.844153	4.005112	3.867578	0.017851	-0.31514	down
2.421597	1.979001	2.05026	2.759136	2.947515	0.017855	0.404647	up
9.814775	9.243311	2.020592	5.833994	6.645774	0.017868	-0.52896	down
1.957601	2.14538	2.512559	3.745967	2.962613	0.017869	0.466636	up
552.2471	535.4131	610.5683	754.5023	648.2418	0.017886	0.334927	up

46.14224	49.49495	65.93694	69.60212	67.97634	0.018091	0.359151	up
0.6074	0.745773	0.604391	1.571444	1.675728	0.018103	0.536201	up
0.190812	0.255719	0.339552	0.494459	0.408473	0.01827	0.534467	up
3.22421	3.329742	2.865847	6.023891	5.018301	0.018344	0.465964	up
9.350472	9.519241	6.932854	7.439569	7.043182	0.01847	-0.28306	down
9.048923	7.619239	9.690391	12.55465	13.36207	0.018507	0.406168	up
25.93625	28.0488	25.58061	39.37576	39.62088	0.018539	0.404316	up
1.438099	1.325232	0.551457	0.87174	1.052212	0.018649	-0.48397	down
1.538338	1.213534	0.161558	1.056919	0.631602	0.018669	-0.52553	down
3.133039	3.044007	1.929202	1.530656	2.063761	0.01874	-0.42676	down
3.880719	3.781968	2.731219	2.830008	2.110022	0.018748	-0.47146	down
0.595952	0.382938	0.667311	0.942501	1.007312	0.018762	0.538591	up
1.795393	1.752391	1.631915	3.572551	2.464269	0.018789	0.481042	up
1.427598	1.20493	1.045431	0.713492	0.808403	0.018875	-0.45143	down
41.77234	43.92577	42.51721	55.38745	52.63435	0.018946	0.32135	up
0.939306	0.945575	0.594484	0.063846	0.03598	0.019012	-0.44879	down
7.782065	7.880049	7.67277	9.878704	10.23479	0.019043	0.345292	up
33.68872	31.0121	30.84359	15.26799	12.78991	0.019049	-0.51986	down
16.0034	12.71449	12.53033	5.508155	6.851672	0.019182	-0.49473	down
5.360755	6.773019	3.630401	4.757768	2.617972	0.01922	-0.48578	down
1053.482	1046.995	853.5748	481.3198	696.5048	0.019237	-0.43252	down
6.058318	5.208261	4.079578	3.437769	3.453142	0.019285	-0.37645	down
1.268786	1.324797	0.871463	0.841566	1.183353	0.019321	-0.41746	down
42.17638	44.596	39.48957	89.02528	77.45456	0.019329	0.479955	up
3.08391	3.219086	4.029841	4.134308	4.748938	0.019421	0.412303	up
78.42908	70.74133	84.1609	94.84991	86.93458	0.019437	0.305668	up
0.465857	0.520933	0.56501	1.088864	1.373073	0.019463	0.519845	up
2.501612	2.490645	2.604016	3.381052	3.491098	0.019489	0.362195	up
11.71694	13.98233	9.815802	8.742428	9.714642	0.019515	-0.34048	down
30.88994	33.41043	29.47259	56.95965	39.47056	0.019521	0.464487	up
4.063004	5.01294	4.285167	10.14593	7.109392	0.019524	0.525363	up
28.61267	25.43731	25.79592	48.32087	36.55692	0.019561	0.445855	up
175.0053	194.1572	150.9034	129.8252	125.5806	0.019611	-0.33853	down
6.203357	5.937949	4.24085	3.755921	5.120498	0.019639	-0.34501	down
2.305307	2.488483	1.524041	1.311011	2.073342	0.019702	-0.4183	down
2.002139	1.632482	2.163843	2.510961	2.405877	0.01971	0.369322	up
34.1001	39.80832	48.72875	49.65824	41.05132	0.019737	0.349394	up
9.982388	9.069232	6.906273	13.02477	15.30537	0.019804	0.445738	up
0.580717	0.637274	0.895824	0.833053	0.737388	0.019825	0.499598	up
9.209681	6.790853	4.984656	9.81042	8.651108	0.019841	0.410148	up
5.979581	5.821572	4.418014	11.15429	12.76069	0.01986	0.524943	up
2.877864	2.85449	1.542482	2.166023	1.948279	0.019909	-0.39046	down
0.656985	1.206761	0.754081	2.044074	2.034898	0.019939	0.534867	up
8.687052	8.40478	5.364442	6.756249	6.969204	0.019954	-0.32397	down
0.716028	0.717672	0.802033	0.811089	1.374187	0.019968	0.492313	up
46.25473	46.71279	37.63331	34.7336	37.36309	0.020009	-0.28186	down
0.102557	0.113139	0.217102	0.215147	0.569269	0.020032	0.526908	up
11.82904	10.34355	13.13401	13.63358	14.46179	0.020043	0.312597	up
12.12201	12.637	11.72431	15.66598	18.26357	0.02007	0.366579	up
2.516052	2.589654	1.378682	8.814714	8.460093	0.020211	0.532283	up

30.58776	32.77865	31.47043	45.99618	38.76184	0.020235	0.380746	up
1.602953	1.244767	1.245235	2.039858	2.486704	0.020362	0.457435	up
5.973608	6.604842	4.652208	19.09876	9.403757	0.020532	0.531201	up
15.08971	16.19955	12.89125	11.32634	10.98115	0.020583	-0.33846	down
1.678928	1.290159	2.588875	1.765765	2.144084	0.020591	0.451969	up
0.262172	0.347588	0.496742	0.697654	0.651687	0.020606	0.510164	up
9.784702	9.70772	6.363249	6.781473	7.733284	0.020679	-0.31866	down
10.95741	9.22904	6.431263	7.661612	6.425917	0.02068	-0.35663	down
36.42224	29.38143	8.949511	27.72174	22.80646	0.020697	-0.51256	down
2865.174	3227.141	3327.885	4660.353	4169.299	0.020765	0.358237	up
8.30846	7.416679	9.302857	10.70711	11.337	0.0208	0.343825	up
19.23791	18.6601	15.9207	10.5069	9.756019	0.02085	-0.43429	down
56.94184	58.70852	63.85932	109.7123	77.85	0.020939	0.419327	up
0.559468	0.454707	0.190489	0.332862	0.20936	0.02099	-0.52669	down
29.82272	27.35871	28.8552	53.42763	46.42543	0.020995	0.436273	up
1732.891	1904.899	2021.208	2412.625	2238.954	0.021014	0.299443	up
7.876505	7.258225	15.72106	10.28959	11.23353	0.02104	0.462664	up
271.4687	274.5584	283.8022	320.4654	360.6089	0.021084	0.274349	up
16.22578	15.18178	9.774218	9.389868	14.40095	0.021236	-0.2782	down
4.635518	3.467522	3.474421	2.311581	2.500132	0.021272	-0.41725	down
0.616731	0.647757	0.626988	1.813468	1.309055	0.021291	0.529344	up
0.931979	1.18768	0.829762	0.478383	0.29277	0.021317	-0.52685	down
2.252549	2.32191	2.074672	3.459527	3.246201	0.021355	0.445902	up
6.646918	5.560308	6.476923	7.620219	8.699449	0.021357	0.320672	up
37.82584	29.05995	37.39093	46.42386	29.56635	0.021431	0.400887	up
2.018467	1.978639	1.156105	1.407242	1.243143	0.021436	-0.44835	down
7.635126	6.611521	5.579189	3.63799	5.098068	0.021441	-0.3795	down
1.836489	3.257226	2.012209	5.391413	4.270114	0.021442	0.490325	up
1.433869	1.86593	0.824025	0.523942	0.964836	0.021507	-0.42445	down
18.13685	17.91861	13.10851	13.21035	12.38791	0.021529	-0.31326	down
1.267108	1.431587	0.790526	1.176988	1.048242	0.021713	-0.45938	down
3.870454	3.859744	3.25659	5.833178	6.359656	0.021762	0.504636	up
22.68536	20.39765	17.30064	10.41582	13.25858	0.021764	-0.42633	down
3.19447	1.860825	2.653635	5.425053	4.161095	0.021796	0.480831	up
5.572992	5.881015	4.007292	4.233401	4.718389	0.02183	-0.28926	down
9.996924	9.553995	11.67089	9.981635	9.530934	0.021912	0.335871	up
57.34849	60.53	49.39952	32.78021	35.01921	0.021943	-0.40646	down
2.869012	2.455905	3.441181	3.144543	3.494887	0.022086	0.375409	up
6.707163	5.922028	4.510912	4.071572	4.393649	0.022174	-0.34661	down
0.311773	0.152954	0.471961	0.794557	0.539118	0.022216	0.525473	up
2.235328	2.72741	2.618301	3.494909	3.910384	0.022246	0.426676	up
21.93912	22.99312	26.18664	25.88279	24.13471	0.022261	0.303255	up
1.226402	1.515519	2.748725	1.473258	1.626448	0.022345	0.488257	up
1.388811	1.564294	3.342479	2.604376	2.29282	0.022376	0.522339	up
4.232539	4.26175	4.305759	5.390885	5.79752	0.02244	0.377947	up
19.96165	22.30877	18.26116	13.97809	14.90036	0.022443	-0.36674	down
1.654101	1.134644	1.542605	2.605149	2.346892	0.022568	0.510498	up
3.25074	2.931878	2.649993	4.980485	6.910867	0.02268	0.4926	up
80.43031	85.64372	57.26615	54.06576	72.07932	0.022687	-0.30865	down
276.5485	272.3273	183.1233	170.6888	200.9592	0.022721	-0.33037	down

6.062642	6.091067	3.805541	4.102016	4.256683	0.022794	-0.32765	down
22.93587	22.25564	25.82963	26.54487	25.40088	0.022833	0.274905	up
15.20726	14.66096	12.07962	8.08845	5.656148	0.022858	-0.47933	down
142.1965	140.6109	116.9431	73.52196	81.64417	0.022944	-0.41132	down
177.2267	161.2645	252.703	202.4757	177.7239	0.022966	0.291499	up
60.51144	76.55552	45.77731	52.06897	56.48644	0.022967	-0.36356	down
1.239344	1.344472	0.648522	0.873325	0.872719	0.02302	-0.44637	down
1.412174	1.286904	1.149706	3.219353	2.907863	0.023082	0.514947	up
7.965667	9.120455	9.177509	10.36558	12.48335	0.023104	0.337066	up
388.5253	430.7402	422.6326	606.3664	526.1403	0.02311	0.330169	up
7.693081	8.251026	4.99915	23.49861	11.75862	0.023139	0.52144	up
3.072156	3.487253	1.553925	2.316855	2.572098	0.023148	-0.45324	down
0.381715	0.153032	0.456114	0.711701	0.50764	0.023228	0.514409	up
23.16604	20.60507	31.25985	26.13792	26.59189	0.023278	0.336913	up
2.222383	2.540068	0.488742	0.877493	1.477634	0.023284	-0.52096	down
3.858999	4.626944	2.558223	3.154086	3.39074	0.023286	-0.37866	down
1.836759	1.951963	2.260478	2.320668	2.948671	0.023319	0.451712	up
31.65668	31.37725	35.61572	32.75308	41.0107	0.023321	0.268218	up
5.38813	5.342838	5.381201	5.868465	7.150587	0.023418	0.346423	up
0.637284	0.75	0.412513	0.466377	0.628212	0.023504	-0.47689	down
3.433398	2.239179	3.008364	2.883232	12.26517	0.023544	0.361432	up
5.857949	6.641085	3.721677	4.000733	4.62042	0.02361	-0.35225	down
71.34562	72.69488	69.2165	89.1217	87.52295	0.02361	0.316123	up
1.625027	1.636611	1.022461	0.910719	1.116811	0.023623	-0.37219	down
22.54625	22.1165	16.94862	17.30512	19.22569	0.023642	-0.263	down
13.75932	15.12007	8.970518	10.69341	10.87811	0.023707	-0.31301	down
6.730005	6.410604	4.483497	5.459397	4.753715	0.023769	-0.34214	down
0.349738	0.561895	0.176889	0.095897	0.250801	0.023852	-0.51762	down
1.663131	2.002109	1.683323	0.669692	0.879136	0.023931	-0.51557	down
19.90831	15.13025	16.37786	28.7889	22.26827	0.023935	0.361302	up
9.469444	9.257768	14.62908	11.4583	12.28783	0.023981	0.371309	up
10.6835	9.136084	3.411705	8.646442	10.76659	0.024099	-0.51771	down
40.558	42.13579	28.89979	31.28126	34.10341	0.024108	-0.26949	down
5.246057	5.735326	5.601633	6.408125	7.567427	0.024125	0.338662	up
6.144289	6.484229	5.626436	8.945228	11.31524	0.024222	0.427912	up
52.06714	55.01988	45.08423	39.20734	38.44944	0.024253	-0.347	down
1.200615	1.150852	1.478192	2.518884	3.083797	0.024309	0.505251	up
12.38338	13.59572	13.73195	49.46837	28.19819	0.024328	0.514368	up
5.349915	3.983234	3.923168	11.00903	10.94382	0.024335	0.501186	up
10.14444	10.31154	6.582452	7.666237	8.856789	0.02435	-0.30479	down
0.555339	0.307674	0.210481	0.082708	0.263061	0.024363	-0.50848	down
1.397351	1.318139	1.643682	1.963683	2.305706	0.024375	0.388985	up
0.214345	0.557282	0.432842	0.671493	0.537251	0.024406	0.517007	up
3.849617	5.144081	5.963434	5.968939	5.225314	0.024453	0.430217	up
3.24804	3.285093	2.907316	1.899798	1.828678	0.024495	-0.44863	down
22.43309	21.25802	17.27383	9.961893	9.958999	0.024507	-0.42609	down
1.254227	1.007303	1.348139	1.967549	1.405873	0.024529	0.477819	up
26.99896	31.6858	20.18319	22.08674	22.17702	0.024615	-0.30258	down
15.11462	13.83816	17.97277	18.54464	16.17968	0.024649	0.303055	up
44.04247	42.37096	30.37485	32.16814	37.93481	0.024849	-0.26903	down

8.420693	7.685494	9.908654	8.854601	10.21221	0.024857	0.294707	up
25.25048	24.46105	27.16151	32.4975	33.3698	0.024866	0.295787	up
13.10326	15.51634	12.22997	7.926599	7.335059	0.024891	-0.44315	down
6.871597	5.660302	6.653135	7.645785	8.911135	0.02494	0.350764	up
8.830392	9.932463	4.433763	7.113437	7.244852	0.024996	-0.38885	down
86.82309	80.27894	71.27219	49.66962	58.96245	0.025016	-0.3655	down
2.386395	2.574735	1.328818	1.565968	1.704769	0.025135	-0.35496	down
8.577682	9.563771	8.773581	13.54577	15.61571	0.025188	0.40573	up
26.26251	28.81064	21.36696	17.40257	17.84804	0.025256	-0.3441	down
5.884163	6.064175	4.38908	3.601829	2.045781	0.025364	-0.48244	down
5.524399	5.135957	4.924146	3.132446	2.747276	0.025448	-0.47087	down
7.72877	7.454075	5.662939	22.55397	12.72703	0.025517	0.506343	up
5.470325	6.358815	5.238217	8.249407	11.41127	0.025571	0.446783	up
1.657333	1.656373	2.107944	3.016489	2.697734	0.025609	0.431227	up
8.271206	7.623549	9.42439	8.499286	12.38985	0.025648	0.345156	up
82.22037	85.86709	82.3916	95.29692	112.4644	0.025722	0.27153	up
2373.591	2595.339	2796.858	3390.389	3268.983	0.025816	0.296992	up
4.391307	4.044652	3.365056	1.09183	1.265485	0.025953	-0.50386	down
152.8718	190.775	209.9294	233.7218	194.3111	0.025987	0.331516	up
10.30353	10.54569	0.171686	5.640049	3.808211	0.026024	-0.45734	down
14.38553	14.22433	10.86665	9.263673	11.32331	0.02626	-0.29844	down
0.911922	1.324077	0.4658	0.44214	0.838373	0.026268	-0.50151	down
1.155612	0.702229	0.62589	0.259403	0.217746	0.026317	-0.50984	down
11.36952	11.20593	7.580667	9.300971	8.905248	0.026351	-0.2763	down
3.445012	4.306134	2.467655	8.958152	7.703435	0.026361	0.507489	up
9.458243	8.454962	6.936215	7.238554	5.562697	0.026422	-0.37029	down
10.15515	10.55217	9.648483	14.68253	16.56819	0.02646	0.38903	up
8.670468	9.599757	6.978469	30.69555	17.12058	0.026511	0.507762	up
1.28979	1.271337	0.747317	7.64377	4.396241	0.026521	0.426249	up
0.508684	0.48291	1.112238	1.778109	0.704729	0.026548	0.448972	up
1.799514	2.558717	0.499213	1.035665	1.17829	0.026757	-0.50233	down
468.09	442.1092	446.204	667.7569	568.2369	0.026792	0.342322	up
11.18305	13.62835	14.07744	20.07555	15.18585	0.026812	0.343548	up
9.778082	8.681327	6.364058	15.57573	17.44168	0.026816	0.481937	up
4.803651	4.450667	4.066077	6.109377	6.708207	0.026824	0.410092	up
24.69119	25.85187	14.71355	17.88763	19.9253	0.026848	-0.34196	down
56.7292	57.30439	47.08264	36.89127	40.01108	0.026898	-0.32794	down
12.04005	9.350187	13.59433	11.45678	14.76726	0.026943	0.380137	up
8.409185	6.832105	8.49616	9.692112	9.109692	0.026946	0.353331	up
25.85224	30.69017	27.0992	37.35593	38.14648	0.02696	0.326827	up
0.513867	0.713896	0.186456	0.308116	0.282118	0.027011	-0.50827	down
1.376027	1.041499	0.756484	1.066938	0.943546	0.027083	-0.36419	down
3.518524	3.971111	3.565471	5.510447	5.437912	0.02712	0.392302	up
2.250104	2.317544	2.803717	3.780294	3.485129	0.0272	0.399768	up
44.75833	47.77884	64.19642	51.36221	52.15898	0.027213	0.336924	up
9.882808	11.98883	9.687887	17.88335	15.02999	0.027268	0.423121	up
140.031	138.8722	157.8679	190.0328	156.3653	0.027285	0.300715	up
4.640301	5.533141	4.783076	10.52247	6.448599	0.027296	0.493362	up
1.106453	0.455883	0.108662	0	0.441007	0.027333	-0.38558	down
1.962311	2.172047	1.189894	1.468228	1.682581	0.027336	-0.35946	down

2.48964	2.209504	1.890425	0.891559	1.229088	0.027342	-0.50521	down
10.68603	9.04648	9.873752	7.458829	9.504642	0.027402	-0.37364	down
82.03312	78.79185	67.01008	55.35956	55.1795	0.027416	-0.31854	down
24.30673	23.83214	20.12867	16.7261	16.80893	0.027445	-0.32934	down
36.59107	50.06506	40.35686	74.51334	55.56185	0.02747	0.440243	up
5.647895	5.075088	4.748473	7.982942	9.358573	0.0275	0.422184	up
2.742175	2.648027	0.996011	1.12896	1.858256	0.027513	-0.50521	down
2.88459	3.509544	1.581222	2.593403	1.593178	0.027657	-0.45921	down
7.647863	5.79931	5.548225	3.78496	4.073937	0.027701	-0.41728	down
1.112696	0.940415	1.155095	1.541299	2.622011	0.027704	0.492687	up
5.372114	5.722419	6.406671	7.298726	8.867587	0.02773	0.386566	up
16.60235	15.08242	19.34023	19.10944	16.59066	0.027737	0.35106	up
4.69665	4.624595	4.590283	6.076421	7.822412	0.027758	0.3936	up
45.46051	43.99564	75.00785	62.99808	53.09343	0.027763	0.3829	up
1.652409	2.346915	1.530057	0.367485	0.393226	0.027811	-0.49315	down
22.83722	21.55452	18.56309	14.14543	16.23498	0.027817	-0.36034	down
6.897494	8.043401	4.960708	3.29796	4.51634	0.027875	-0.43137	down
3.061106	2.556838	3.570927	2.997573	4.032402	0.027948	0.35835	up
51.84309	52.95809	43.67177	29.56975	28.4526	0.027991	-0.40954	down
2330.034	2548.567	3002.845	3190.1	2871.774	0.028017	0.290936	up
4.394322	4.132376	5.430815	5.788722	5.27878	0.028093	0.352899	up
18.84473	21.87613	6.95525	14.65962	20.92318	0.028104	-0.50476	down
19.71286	18.92652	14.45228	39.6338	35.26497	0.028121	0.472923	up
3.337801	3.563756	3.552116	4.82075	4.52395	0.028122	0.338213	up
7.989105	7.777048	6.138097	5.358111	5.589745	0.028179	-0.33776	down
23.01334	20.87912	24.70421	22.89953	28.54461	0.028401	0.298637	up
5.57032	5.20318	5.429354	6.577471	5.938039	0.028402	0.303009	up
16.60707	17.20588	16.12105	20.85757	23.06352	0.028574	0.347042	up
18.12379	22.4893	10.29666	14.41922	14.37162	0.028792	-0.37722	down
1.609816	1.768394	1.107601	1.086855	1.111351	0.028795	-0.39502	down
0.668692	0.756655	0.226063	2.264264	3.013553	0.028847	0.444489	up
1.9846	1.94098	2.336522	3.002396	3.07177	0.028853	0.409676	up
10.18384	9.686909	17.6605	10.70004	12.29741	0.028876	0.412209	up
21.96245	21.51043	28.27733	23.80897	26.68907	0.028888	0.305511	up
4.173229	5.111794	7.30304	5.154134	6.138705	0.028895	0.395711	up
4.453843	4.829546	6.188068	6.734954	11.76704	0.028897	0.466197	up
3.49015	3.491465	2.151569	2.849406	2.811049	0.028915	-0.30552	down
8.680228	8.224168	6.35087	7.627014	5.357054	0.029004	-0.43699	down
17.28782	18.93544	17.93362	21.36634	24.04063	0.029036	0.305745	up
63.58229	60.987	79.45181	65.40297	73.3948	0.029088	0.301615	up
0.490149	0.373166	0.402607	0.681553	0.744656	0.029225	0.495849	up
8.214256	7.470584	6.386479	4.760822	5.104069	0.029236	-0.36842	down
79.2575	94.30447	83.02951	67.30274	109.5162	0.029248	0.277931	up
1.861838	2.156685	3.253937	1.802153	3.806185	0.02931	0.464968	up
9.707793	7.812451	12.92668	14.65642	10.35896	0.029321	0.407114	up
2.714753	2.591128	2.323314	5.276322	7.033702	0.029351	0.499312	up
17.49947	17.16236	20.57267	22.91562	18.51879	0.029388	0.31859	up
0.810269	0.556354	0.495664	0.198934	0.125702	0.02953	-0.49371	down
0.734704	0.825981	3.397082	1.401647	2.429275	0.029545	0.500234	up
0.257049	0.520793	0.51443	0.848447	0.689776	0.029573	0.49904	up

51.85664	46.76137	73.66782	48.97659	75.27204	0.029585	0.379871	up
25.72541	24.78176	19.46035	17.98645	20.54871	0.029586	-0.26157	down
26.7916	21.6104	23.28431	32.26096	40.5249	0.02964	0.395127	up
21.533	18.91146	17.62676	12.06774	13.57262	0.029668	-0.3839	down
1.468195	1.369044	1.072901	0.622005	0.588131	0.029674	-0.4785	down
7.026033	6.937972	4.795056	5.189339	5.632101	0.029732	-0.26384	down
26.48967	27.32147	16.90117	23.60674	21.846	0.029799	-0.34026	down
91.29499	103.4587	90.10373	128.6433	121.7681	0.0298	0.323569	up
0.328077	0.230487	0.495523	0.66547	0.753788	0.02984	0.493711	up
1.589839	1.718059	2.224106	1.908859	2.17252	0.029906	0.330995	up
286.4779	300.7445	220.9807	472.3688	421.0237	0.029931	0.443899	up
2.870797	2.731196	1.938063	1.796876	1.412918	0.029961	-0.41543	down
7.531957	8.08872	6.207779	5.782667	5.628538	0.029991	-0.30986	down
70.62876	69.93068	58.66741	28.37733	49.96231	0.030069	-0.43148	down
24.41649	23.49226	24.16244	29.89442	31.25335	0.030181	0.285912	up
6.146599	6.321147	3.480084	4.866862	4.681597	0.030223	-0.34428	down
14.03941	12.88073	19.46079	14.45547	17.3838	0.030235	0.317163	up
4.530306	6.614214	5.378285	9.715708	7.115824	0.03025	0.469484	up
0.069951	0.136043	0.12432	0.196318	0.225652	0.030303	0.494676	up
1.682204	1.56581	1.159934	0.57145	0.355192	0.030332	-0.48506	down
10.11422	11.23169	7.182688	6.312926	8.681445	0.030335	-0.34265	down
0.541162	0.576262	0.38803	0.211274	0.375442	0.03036	-0.47926	down
5.009407	5.34867	4.24105	2.701739	3.856172	0.030371	-0.42324	down
5.454319	5.623523	7.049889	8.074998	7.502066	0.030384	0.338711	up
45.69761	44.95309	50.7455	58.9336	54.34446	0.030386	0.277513	up
1.293472	0.752706	1.182916	2.613174	1.700628	0.030405	0.494153	up
0.742402	0.72007	0.382449	0.478531	0.555238	0.030463	-0.42387	down
5.120306	3.449746	5.687811	7.528347	5.113648	0.030551	0.429777	up
5.407877	5.151048	2.855646	0.957083	3.062262	0.030565	-0.49692	down
18.77776	20.95602	19.41564	25.99797	21.81747	0.030658	0.350618	up
5.721665	5.868357	6.758306	7.367655	6.636508	0.030735	0.322611	up
63.11212	66.73177	60.09802	99.31648	95.5727	0.030751	0.368532	up
0.101526	0.107102	0.109784	0.244652	0.38852	0.030778	0.494713	up
73.21424	77.02071	48.50197	59.14516	58.13625	0.030814	-0.28664	down
3.259575	2.761062	3.534862	4.092518	5.042958	0.030896	0.421029	up
3.056816	2.757614	2.272207	6.412641	4.87723	0.030898	0.469063	up
30.7817	32.72902	47.5526	41.88399	38.10619	0.03092	0.350995	up
1.69	1.952996	0.707922	1.610314	1.185734	0.03095	-0.49574	down
15.9591	15.31891	11.23638	10.74897	13.19395	0.030963	-0.27142	down
20.68697	19.15606	15.09212	13.8833	15.35923	0.03103	-0.27539	down
26.49535	27.42028	23.02872	44.64115	39.61625	0.031113	0.407987	up
2.971402	3.522468	1.453229	2.752542	2.268497	0.031114	-0.42496	down
0.412899	0.319729	0.204237	0.067489	0.214834	0.031137	-0.49184	down
44.97945	45.61441	55.21842	58.83039	53.69222	0.031164	0.283549	up
2.156096	1.897226	1.548733	8.260485	5.262212	0.03117	0.493247	up
0.726916	0.964697	0.889617	1.804519	1.855393	0.031218	0.473731	up
1.24912	1.071391	0.928946	0.533471	0.945718	0.031267	-0.48692	down
3.265166	2.279042	3.316782	5.443058	4.890852	0.031271	0.44467	up
5.577546	5.227056	5.590405	6.148915	6.718514	0.031503	0.307209	up
0.482145	0.47422	0.897121	27.13159	9.6687	0.031625	0.31856	up

672.3668	780.1878	792.1054	1037.713	819.7652	0.031662	0.323357	up
6.411795	6.26794	6.055036	8.676305	9.35614	0.031736	0.391409	up
10.64804	8.798584	7.754125	6.576224	7.982428	0.031742	-0.34123	down
1.95061	1.429312	1.657046	3.496549	2.86039	0.031805	0.455821	up
33.00243	30.16816	22.85624	25.30361	24.87899	0.032118	-0.27503	down
3.197951	3.387506	2.849086	2.259435	1.783112	0.032126	-0.43433	down
0.501108	0.685062	1.123185	1.644467	1.067858	0.03213	0.492558	up
36.5893	33.69762	26.45063	31.48819	26.14539	0.032177	-0.40611	down
19.88186	18.44173	22.02947	20.18167	25.37183	0.032215	0.297537	up
35.63771	42.06493	25.2339	69.35884	68.20737	0.032253	0.468623	up
13.73235	18.52642	17.58581	20.22481	19.44385	0.032258	0.297795	up
2.011338	2.915593	1.428972	14.93335	9.308148	0.032258	0.413291	up
3.300717	4.12395	3.303271	5.049679	9.706248	0.032261	0.462915	up
0.263189	0.275895	0.104989	0.114084	0.17639	0.032307	-0.48637	down
15.17762	15.1364	11.30281	9.926317	11.51448	0.032313	-0.28812	down
427.9831	433.7303	465.6269	558.3104	555.7347	0.032356	0.28274	up
5.731847	5.185735	5.959187	7.266592	6.752733	0.032358	0.298511	up
3.836401	4.15742	2.975537	2.820071	3.527822	0.032372	-0.44914	down
1.073291	0.942306	1.197015	1.294641	1.895806	0.032412	0.42117	up
2.954702	2.90788	2.030398	1.668436	2.250232	0.032473	-0.30302	down
638.0226	638.0338	597.9645	877.5231	944.3663	0.032496	0.338571	up
0.232738	0.157657	0.157821	0.555258	0.83183	0.032528	0.480335	up
121.4176	131.1156	144.6252	136.8109	181.9857	0.032561	0.298686	up
61.35652	66.11541	68.82165	72.97378	70.38887	0.032596	0.292652	up
0.42927	0.363604	0.555039	0.650333	0.935949	0.032631	0.467339	up
27.06906	27.70517	24.47434	45.60986	46.87175	0.032644	0.409079	up
8.232978	8.699376	6.44428	4.52282	6.793893	0.032682	-0.35936	down
0.503953	0.723038	0.810137	1.607775	1.432055	0.032721	0.490641	up
44.70236	42.08811	35.98138	30.18077	33.53475	0.032739	-0.29428	down
11.15178	12.37169	14.38525	12.55042	14.49031	0.032768	0.30946	up
122.1874	128.3158	112.5726	91.00134	72.01178	0.032813	-0.40524	down
5.924496	4.676736	5.668333	7.667132	8.310604	0.032848	0.365289	up
11.3717	11.77223	9.182338	9.647399	8.599985	0.032862	-0.33706	down
31.3635	29.88332	35.29325	37.39265	32.83736	0.032871	0.297085	up
11.92045	13.20141	14.74454	14.51248	13.49332	0.032873	0.329625	up
0.550228	0.800724	0.762924	0.807514	1.196164	0.032891	0.432034	up
4.832127	3.940384	4.687433	6.78582	6.057028	0.032902	0.377622	up
3.100025	3.039703	1.881942	2.257854	2.716276	0.033063	-0.33061	down
1.974122	2.06616	2.173293	3.368817	2.024177	0.033197	0.479328	up
10.55595	9.934033	8.321955	7.115664	6.425796	0.033271	-0.34738	down
36.10351	42.83078	36.04603	60.84749	48.25219	0.033274	0.409984	up
0.483655	0.635394	0.215388	0.312317	0.289866	0.033295	-0.4846	down
9.934465	10.94454	6.951298	5.194795	4.820387	0.033312	-0.4282	down
0.676236	1.154562	0.892965	1.315513	0.681833	0.033314	-0.47696	down
44.49654	48.38737	31.9663	43.07124	34.42682	0.033331	-0.40153	down
77.60287	75.25476	62.10425	44.35795	63.01003	0.033342	-0.33826	down
1.900941	2.339706	0.746624	1.493131	1.281764	0.033461	-0.42419	down
2.317702	2.427436	2.701718	2.517769	3.580811	0.033489	0.349346	up
0.916093	1.197873	0.78013	1.540775	2.42671	0.033505	0.472014	up
2.183524	1.677119	0.434015	0.820508	0.93146	0.033537	-0.48859	down

1.607118	1.486219	1.755918	2.312695	2.288872	0.033583	0.362314	up
1.958303	1.717801	1.59052	2.760304	3.868467	0.033586	0.442378	up
21.07998	23.02845	30.48675	19.49846	28.35036	0.03359	0.3725	up
14.533	15.10798	15.16074	17.55363	17.78027	0.033709	0.265185	up
1.022053	0.704684	0.952719	1.152268	1.771679	0.033778	0.414992	up
128.0189	145.8142	123.023	188.1018	205.3478	0.033785	0.371183	up
0.741281	1.020658	1.669778	1.351989	1.442371	0.033827	0.457608	up
26.42054	26.12673	27.75098	32.56729	30.49314	0.033856	0.305726	up
19.40816	18.77308	24.72413	21.23078	22.99072	0.033936	0.288468	up
26.52996	24.91668	18.64642	36.07327	35.30797	0.033936	0.440639	up
7.87852	8.590058	9.732184	9.843631	8.837629	0.03407	0.317062	up
26.81465	27.57814	31.48461	28.77019	31.4743	0.034081	0.315663	up
1.002375	0.862021	0.513745	0.661645	0.725352	0.034117	-0.48544	down
87.14009	100.4301	104.301	108.287	109.7091	0.034121	0.262731	up
0.645174	0.334469	0.279711	0.017482	0.091139	0.034128	-0.42897	down
59.12751	56.35994	48.24724	90.95314	74.81271	0.034191	0.354653	up
25.5493	25.18005	23.82469	37.63962	36.19007	0.034258	0.346766	up
173.4498	168.3877	141.7398	114.1138	130.8444	0.034484	-0.28929	down
33.23856	31.34517	25.61248	25.45818	30.31362	0.034626	-0.29373	down
218.5661	222.8662	168.8394	151.04	178.0833	0.034676	-0.26122	down
4.806621	4.232726	7.599647	6.441588	5.091806	0.034684	0.411103	up
0.347056	0.357086	0.257331	0.241927	0.266335	0.034817	-0.45237	down
66.50713	74.13706	59.46525	40.35008	52.76232	0.03483	-0.34807	down
3.196361	2.757535	2.60603	7.619723	5.108849	0.034888	0.468433	up
5.27474	5.134145	5.083836	5.778935	6.500239	0.034898	0.336177	up
53.74998	36.43276	34.86537	24.75708	23.90446	0.034932	-0.40342	down
9.665228	8.712351	6.505358	7.140814	8.819167	0.034958	-0.27849	down
3.314577	3.163778	3.914586	3.665105	3.762852	0.035052	0.305884	up
7.921129	8.7444	8.992544	9.020524	12.07951	0.035138	0.303422	up
0.562008	0.545302	0.235653	0.303156	0.476882	0.035151	-0.48411	down
3.604437	4.441083	4.590218	5.721903	5.226669	0.035163	0.357216	up
126.2507	148.1377	114.6609	247.3138	201.2288	0.035193	0.420879	up
3.622081	3.927779	3.258072	3.16606	2.564283	0.035291	-0.29413	down
20.66871	20.79072	20.0254	33.01707	29.86219	0.035515	0.366712	up
5.999511	6.082086	6.344468	7.580025	8.33379	0.035588	0.290665	up
0.673801	0.642208	0.828549	1.345022	1.282953	0.035634	0.477248	up
3.86735	3.274553	3.209748	2.542306	3.122661	0.035782	-0.32553	down
0.685212	0.997815	0.369323	0.384659	0.380823	0.03581	-0.47902	down
67.6557	63.08195	52.60292	44.45668	54.56121	0.035886	-0.27508	down
9.550011	9.092049	8.681413	21.41808	20.4748	0.035933	0.450793	up
0.813446	1.204068	0.511286	0.277331	0.958151	0.035937	-0.47853	down
2.544864	2.457095	1.71685	9.016096	5.144655	0.036062	0.48172	up
44.17246	42.44707	32.37932	34.49732	38.66728	0.036096	-0.25112	down
14.65338	13.44816	15.05255	11.97284	14.07009	0.03612	-0.2594	down
6.010335	7.060172	8.693255	7.709742	6.92213	0.036151	0.366939	up
14.67772	14.66619	6.527201	12.16847	11.25855	0.036196	-0.38711	down
9.706624	8.330067	11.12862	11.25717	11.69993	0.036197	0.314238	up
3.639708	2.635177	2.724293	2.104337	1.901914	0.036211	-0.41327	down
5.9663	7.200896	4.445565	15.69146	11.5852	0.036227	0.479047	up
3.438238	2.585671	2.430425	4.530216	5.133657	0.036309	0.437457	up

1326.27	1581.929	1337.307	1938.019	1965.677	0.036313	0.306773	up
3.558795	3.794429	4.777447	4.044038	4.126122	0.036356	0.373254	up
3.244548	3.76387	2.261781	1.874653	2.132533	0.036465	-0.38289	down
2.019102	1.65642	1.260686	1.34167	1.371727	0.036487	-0.35093	down
29.28774	30.90578	22.53204	22.50881	26.10149	0.036523	-0.25449	down
6.195695	6.977617	3.334294	6.154541	4.716532	0.036623	-0.35636	down
13.63177	15.49228	16.50884	19.02451	16.75952	0.036631	0.288913	up
72.16031	70.53007	54.1715	164.7138	129.011	0.03666	0.455498	up
0.658317	0.633156	0.895934	0.815575	0.989272	0.036679	0.448803	up
1627.466	1898.579	1977.918	2520.56	2056.47	0.036706	0.308817	up
51.64237	55.391	33.79472	44.17684	45.0295	0.036713	-0.27426	down
3.023823	2.608367	1.606202	1.926079	2.884763	0.03672	-0.3927	down
12.83431	13.07123	10.81712	7.986745	8.969336	0.036754	-0.32959	down
8.79053	11.22147	9.750204	14.74688	13.34213	0.036834	0.406523	up
5.810122	5.841332	11.28995	5.764253	8.222946	0.03688	0.419956	up
2.169395	2.077736	1.882638	3.870965	3.254669	0.036922	0.418309	up
33.26905	35.34304	30.62007	18.60865	20.5157	0.036939	-0.39244	down
0.94711	0.658415	0.2874	0.203934	0.64121	0.036964	-0.47636	down
0.801084	0.625598	0.341846	0.24816	0.516077	0.037026	-0.47901	down
3.73709	3.064977	2.765822	1.938184	2.571927	0.037062	-0.40723	down
5.413971	4.884486	3.850009	4.269588	4.879832	0.037157	-0.29622	down
0.565274	0.972038	1.04801	1.766578	1.526083	0.037279	0.473917	up
29.58296	28.33358	23.84309	20.16583	21.76337	0.037505	-0.29196	down
7.498663	6.074889	6.838448	11.66381	10.25847	0.037509	0.444912	up
14.29235	12.04885	10.4303	7.364564	10.78171	0.037627	-0.37615	down
3.41429	4.202702	2.124735	2.708535	2.278173	0.037681	-0.38842	down
31.90645	32.23038	31.09889	39.91993	39.38632	0.037701	0.319907	up
4.267677	4.624155	4.103116	5.670924	6.807635	0.037738	0.391279	up
3.591462	3.238791	4.422753	3.643783	4.619866	0.037778	0.338072	up
22.41	21.90868	12.95248	16.22275	14.50943	0.03778	-0.33498	down
14.19738	12.66744	13.16404	14.92902	17.94311	0.037802	0.285096	up
11.12759	10.63897	13.62931	10.43536	12.25257	0.037867	0.335115	up
7.683997	7.075097	3.658803	5.357703	6.097879	0.037908	-0.36199	down
375.1646	419.473	329.5752	739.1211	592.2556	0.037924	0.414715	up
1.206017	1.151247	1.4837	2.447733	2.185674	0.03793	0.455062	up
2.094393	1.369063	2.014945	2.792309	2.283771	0.037945	0.417524	up
21.07801	20.27871	17.08199	10.70976	10.82794	0.038038	-0.39219	down
7.998271	8.535552	8.045047	8.567283	9.09488	0.038086	0.380377	up
5.118951	4.804163	5.904888	5.343885	6.241549	0.038184	0.289764	up
2.254875	1.516234	2.977842	2.213703	3.404368	0.038207	0.385339	up
140.7182	156.3246	118.1424	109.4066	93.50335	0.038265	-0.31305	down
0.331007	0.463071	0.167577	0.23606	0.270653	0.038269	-0.44982	down
145.4356	138.2927	101.1089	197.0412	152.2283	0.038308	0.373534	up
3.489354	3.113399	2.170816	7.60531	5.569856	0.038323	0.466213	up
31.71705	37.11623	37.68688	35.93552	37.95084	0.038331	0.287491	up
8.720273	9.722328	9.003441	10.97426	13.42003	0.038397	0.355104	up
6.212944	5.314769	3.847123	4.212507	4.653311	0.038452	-0.293	down
0.880031	1.940422	0.386106	0.860191	0.638761	0.038511	-0.47523	down
3.679249	3.398101	2.62906	2.32725	3.242169	0.038558	-0.38475	down
62.86012	54.92326	50.60151	43.41712	32.44561	0.038574	-0.32663	down

8.634822	10.9773	9.841292	18.78361	13.90661	0.038608	0.423479	up
0.181077	0.247146	0.227665	0.357654	0.48864	0.038634	0.468706	up
9.145393	6.577351	6.943511	3.801159	6.534592	0.038668	-0.37177	down
12.51222	12.33719	10.97173	6.91898	7.083603	0.038695	-0.38996	down
9.195032	10.15243	3.97175	6.960068	6.321465	0.038732	-0.40486	down
6.924616	6.365893	6.829964	9.79196	10.31992	0.03879	0.347883	up
4.439622	4.537349	2.617511	4.213237	2.927818	0.038837	-0.41198	down
4.493762	4.870224	3.557994	9.817758	6.378416	0.038867	0.447803	up
26.60458	29.04337	16.77361	23.56897	23.25338	0.038884	-0.33669	down
17.0895	19.33135	19.33322	19.33548	21.7616	0.038934	0.305405	up
1.270561	1.584065	0.745918	0.771371	0.878038	0.038946	-0.42462	down
6.830646	6.598802	6.68922	12.76299	11.33572	0.039004	0.406657	up
1.5985	1.63132	1.232698	0.985355	0.907142	0.039011	-0.43572	down
21.05012	24.18142	18.25982	14.28885	18.41273	0.039026	-0.31828	down
2.345031	1.870384	2.430752	2.314837	3.668161	0.039087	0.401007	up
7.864011	7.099205	8.197875	8.914364	8.328939	0.0392	0.315675	up
34.73561	35.55463	27.94877	27.47809	31.07231	0.039216	-0.24941	down
16.13408	16.46572	14.1551	6.66131	11.77004	0.039341	-0.43088	down
7.549225	6.107038	9.179465	9.160375	9.231248	0.039387	0.327209	up
11.17707	8.471772	8.663948	7.481671	5.514111	0.039396	-0.36713	down
0.375833	0.494569	0.381029	0.813632	1.27205	0.039416	0.468616	up
12.34844	9.647525	18.23998	14.12687	12.19703	0.039554	0.389734	up
34.94799	31.16611	46.07203	39.78681	36.4845	0.039612	0.306153	up
70.94369	70.7417	59.7204	50.3634	55.27587	0.039636	-0.27816	down
0.239124	0.26073	0.098069	0.027602	0	0.03972	-0.36839	down
20.93013	18.54088	16.05239	11.32105	15.57282	0.039769	-0.33221	down
18.74163	19.52925	16.54532	12.70244	15.29429	0.03979	-0.33086	down
7.086198	7.5525	7.264691	8.236506	9.168521	0.039797	0.377371	up
30.92466	28.10079	26.53834	13.77763	16.66815	0.039829	-0.42509	down
2.035593	1.20712	0.961772	0.617122	1.387604	0.039834	-0.46456	down
32.29449	44.80661	24.23169	356.4191	206.3223	0.039843	0.370553	up
12.85039	13.48748	16.03336	14.484	16.13265	0.039906	0.272567	up
3.540407	2.194978	3.619849	5.03697	3.398504	0.04005	0.408776	up
2.255023	2.212821	1.715879	1.327969	1.271725	0.040077	-0.37223	down
0.478628	0.427856	0.443063	0.646316	1.231744	0.040118	0.460544	up
0.802754	0.675109	0.359249	0.212089	0.448631	0.040198	-0.47015	down
66.59529	66.78278	45.85803	52.48176	58.62644	0.040209	-0.24478	down
8.887502	8.522717	9.570012	10.64186	12.13771	0.040283	0.299417	up
4.219636	2.862195	2.65362	2.491453	1.975064	0.040344	-0.40375	down
7.573872	5.859546	5.384146	4.249011	3.894546	0.040348	-0.38625	down
66.56715	81.45932	99.65537	90.75266	92.10609	0.040355	0.310666	up
2.437437	2.895514	5.327221	3.243347	3.368632	0.040414	0.423374	up
0.531202	0.514486	0.71048	0.619337	0.93305	0.040511	0.432745	up
3.039162	4.09149	3.770968	8.771722	5.372437	0.040526	0.444932	up
34.82776	38.13124	30.44508	25.43172	27.26297	0.040691	-0.29103	down
6.481992	6.177211	8.46181	7.039567	7.492118	0.040784	0.427545	up
1.641324	1.947452	2.031343	0.87433	1.212554	0.040863	-0.46333	down
6.389237	6.37786	6.500106	8.090869	7.639286	0.040957	0.2921	up
138.7987	151.1724	189.2976	192.7062	151.5345	0.041187	0.31528	up
1.600301	2.350392	0.581301	26.64703	20.3184	0.041225	0.319665	up

5.314019	5.192976	5.492084	6.373197	5.899949	0.041262	0.281696	up
666.1746	511.6913	531.5682	667.8621	665.8614	0.041286	0.309717	up
14.22139	13.22201	9.510186	11.28239	13.00478	0.041289	-0.30064	down
0.900929	1.020768	0.698441	1.920331	2.100157	0.041402	0.467195	up
21.22199	22.68585	17.84048	13.47852	17.23536	0.04155	-0.30765	down
11.86745	11.35166	12.38425	13.3774	13.31734	0.041551	0.249419	up
1.305029	1.486704	0.846163	3.426043	3.058445	0.041563	0.465624	up
25.1463	30.24854	26.04537	36.95738	33.24375	0.041586	0.339393	up
16.13154	18.26966	13.41325	87.73072	48.78282	0.041608	0.427047	up
10.16245	9.956492	8.48659	7.334472	6.734355	0.041666	-0.34878	down
1.421932	1.034144	0.358821	0.780122	1.216967	0.04172	-0.46807	down
0.205084	0.407666	0.458898	0.862814	0.793689	0.041831	0.466538	up
2.014931	1.75427	1.88906	2.467588	2.965212	0.041851	0.367967	up
3.134725	3.083359	2.632768	5.375568	5.338589	0.041907	0.41912	up
2.512014	2.854652	1.668897	1.672092	1.817675	0.041952	-0.36534	down
13.60708	12.62638	16.66543	16.03474	15.20381	0.041972	0.311781	up
28.00961	25.10963	24.25524	49.49611	35.70355	0.042059	0.38621	up
4.6982	4.834736	4.568753	6.18115	6.009026	0.042069	0.342138	up
18.17145	17.84165	19.05327	21.16514	19.65348	0.042069	0.315117	up
12.27523	15.93063	11.27086	33.10149	25.2766	0.042203	0.456317	up
1.599304	1.936127	1.240752	3.126869	3.216681	0.042232	0.423319	up
44.78789	42.33098	58.28552	72.53346	54.67513	0.042342	0.344992	up
3.918572	3.482605	2.924551	1.761518	2.016266	0.04243	-0.40133	down
0.458773	0.399669	0.42284	0.511363	1.016751	0.042705	0.465701	up
86.89278	82.43809	61.06503	60.14807	45.44168	0.042706	-0.34005	down
0.708745	0.477063	1.00497	0.667441	1.003768	0.042779	0.465509	up
2.524055	1.695101	1.564374	3.567942	2.480879	0.042852	0.39134	up
6.144116	5.438382	4.733609	8.799663	8.08654	0.04295	0.427975	up
15.04572	16.37933	18.933	18.92291	18.83578	0.043139	0.26644	up
4.146993	4.694558	5.996955	5.617374	6.93225	0.043159	0.349439	up
1.415753	1.322475	1.79897	0.725972	1.047276	0.0432	-0.44736	down
10.59615	9.570246	8.25218	6.71957	6.856995	0.043226	-0.29609	down
1.524554	1.57858	1.526268	2.552043	1.953329	0.043233	0.381822	up
12.50576	13.41385	10.17343	7.840301	8.314547	0.043344	-0.33269	down
7.858669	9.09669	9.192528	12.07519	11.11377	0.043386	0.313533	up
15.79298	14.45638	24.11975	15.04213	18.62094	0.043393	0.353874	up
0.489542	0.631858	0.519221	1.042681	1.160764	0.043429	0.463061	up
2.808532	3.828101	1.969961	8.310435	9.683947	0.04343	0.464218	up
3.849097	3.553858	2.012357	13.24207	7.101053	0.043448	0.463141	up
3.673433	4.512798	3.709384	4.752518	4.036825	0.043587	0.285797	up
9.052465	9.504221	9.860984	10.05509	13.17311	0.043668	0.327378	up
3.472043	2.729334	2.839807	5.322862	4.293702	0.043697	0.412597	up
30.33512	31.9701	22.3062	21.90814	23.42425	0.04378	-0.27168	down
4.27885	4.390653	5.272018	5.115735	5.061997	0.043829	0.275808	up
12.9468	15.97385	12.27799	9.444427	10.58371	0.043849	-0.32731	down
6.012856	6.836377	8.016536	8.263119	7.455592	0.0439	0.305123	up
0.419326	0.173161	0.690294	0.742169	0.505996	0.043954	0.462525	up
7.937215	6.836367	7.796461	7.88571	8.406736	0.043978	0.257637	up
11.15736	11.52846	8.690033	8.144716	9.557574	0.044102	-0.25238	down
42.80051	43.82496	47.25417	60.06262	47.04165	0.044247	0.311782	up

262.7095	296.2289	249.5911	378.5336	391.3583	0.044522	0.343819	up
8.890621	9.24907	6.716406	4.93174	5.987515	0.044694	-0.33003	down
16.09849	18.78917	15.55268	28.27246	24.56062	0.04471	0.371318	up
5.278163	5.363171	1.402464	4.951029	2.907187	0.044712	-0.4539	down
11.03737	11.85021	9.916618	23.75356	12.58191	0.044792	0.427543	up
4.607288	5.156163	4.934064	6.01388	5.608392	0.0449	0.32888	up
5.461019	5.863402	6.56248	7.703162	6.944202	0.044952	0.407769	up
1.517578	0.638535	0.807169	0.416303	0.546372	0.044977	-0.46083	down
3.620006	3.305896	2.96687	1.899295	2.287239	0.045001	-0.41663	down
2.455626	2.181201	1.904561	3.574996	3.745105	0.045005	0.408085	up
0.806436	2.141593	0.873088	0.716037	0.876644	0.045006	-0.45519	down
10.89463	9.192245	15.4508	10.69616	11.76504	0.045031	0.344169	up
25.5741	24.95607	21.1717	42.47789	46.69761	0.045052	0.402252	up
15.12402	26.29968	10.04503	126.9553	71.13968	0.04507	0.361577	up
2.638053	2.42998	1.51378	6.652636	4.349991	0.045132	0.457431	up
2.474059	3.254558	2.996361	4.240727	5.531589	0.045133	0.431798	up
3.959046	4.470607	3.927161	4.998537	5.829978	0.045191	0.313996	up
7.03585	7.857165	5.948474	14.80267	9.738884	0.045267	0.446858	up
2.954102	2.873389	5.217089	3.539244	3.878283	0.045307	0.388473	up
4.028869	3.726714	2.934008	2.346337	1.845773	0.045318	-0.39421	down
1.910836	2.126462	2.433179	2.643365	3.669528	0.045405	0.42173	up
3.376642	3.877066	1.860793	7.283606	8.980354	0.045406	0.459907	up
3.157897	3.222941	2.248889	2.024355	2.065337	0.04542	-0.36998	down
6.033401	6.745904	7.249737	8.350798	6.879274	0.045472	0.297936	up
5.531599	5.737554	4.715405	9.181127	7.380167	0.045517	0.391892	up
8.919767	8.256076	13.30609	9.71737	11.10718	0.045537	0.336389	up
5.277858	6.551804	6.658932	7.315918	7.243929	0.04555	0.316638	up
1.757968	1.311676	3.238172	1.55429	2.285368	0.045576	0.442659	up
5.510369	5.302684	3.727483	4.302005	4.821535	0.045774	-0.26075	down
8.854158	9.599536	7.625305	6.127352	5.43869	0.045861	-0.33706	down
22.11674	22.22078	30.05044	26.79451	23.23984	0.045865	0.304788	up
1.242274	1.069308	0.548618	0.652911	0.690341	0.045908	-0.45509	down
8.116201	7.438517	6.352572	5.468253	5.811527	0.045941	-0.30461	down
12.56502	10.71412	11.85501	15.39882	13.77853	0.045944	0.300726	up
0.518617	2.005507	1.537025	2.804566	2.242132	0.045985	0.458702	up
109.8466	114.7555	79.59103	94.12936	100.1939	0.046124	-0.26298	down
9.004202	8.620655	6.303284	7.522767	7.389431	0.046149	-0.27303	down
12.24428	9.920578	14.92124	13.04739	15.72523	0.046205	0.330269	up
27.78532	28.99504	27.91174	38.88476	30.64132	0.046226	0.33283	up
16.15409	13.289	20.28363	15.32521	17.00553	0.04623	0.295077	up
1.797617	1.98831	2.445072	1.894647	2.429855	0.046278	0.314358	up
1.265718	1.053283	0.971605	2.036059	2.430006	0.046354	0.444652	up
1.458223	1.097878	1.173739	2.497426	2.505584	0.046555	0.435583	up
2.336822	2.007284	1.702465	1.002871	1.059727	0.046568	-0.42293	down
105.9325	113.6731	144.2754	146.4791	117.0399	0.04666	0.305184	up
4.345333	4.644525	4.457739	8.423115	6.078877	0.046671	0.374423	up
1.800886	1.464955	1.224636	0.746782	1.145248	0.046749	-0.39548	down
14.97658	16.24869	11.59358	13.19715	12.49378	0.046849	-0.33633	down
1.112624	1.360589	1.903339	1.952928	1.433711	0.046854	0.415982	up
30.25538	32.39045	25.64556	19.64449	14.47193	0.04687	-0.39179	down

8.404613	9.029551	7.184615	5.366267	5.52617	0.046895	-0.32752	down
1.163715	1.111121	0.82692	0.343519	0.529721	0.046929	-0.44938	down
11.77649	11.39087	11.9938	12.80473	14.71936	0.047048	0.258776	up
2.953816	3.247725	3.238631	2.49408	5.694574	0.047052	0.3772	up
1.434028	1.60708	1.318656	2.393405	3.988207	0.047103	0.449599	up
8.585722	6.253429	7.130503	5.543911	5.589962	0.047161	-0.38263	down
37.1156	31.19581	23.64795	13.80433	27.084	0.047193	-0.39234	down
4.343181	4.458844	3.77543	2.946999	3.718037	0.047195	-0.35459	down
12.29946	11.80585	13.81134	12.08607	14.8274	0.047306	0.276641	up
10.12609	11.40725	12.17028	13.31546	12.20604	0.047407	0.262259	up
12.77659	12.04997	10.155	9.233659	10.06764	0.047414	-0.26791	down
1105.353	1192.421	1342.11	1554.532	1339.667	0.047464	0.27537	up
21.44756	19.34431	26.22101	28.08012	24.05568	0.047504	0.311361	up
0.470233	0.685177	0.697219	1.926578	0.55513	0.047559	0.434729	up
4.550362	4.734314	2.67502	3.716937	3.797134	0.047737	-0.30271	down
0.371812	0.331769	0.825707	0.464857	0.408706	0.047774	0.439871	up
36.10598	35.96919	29.07154	26.85026	25.33386	0.047829	-0.30809	down
1.852187	2.019173	1.330317	1.13555	1.644794	0.047911	-0.3097	down
1.261688	1.168625	0.84133	0.83146	0.582466	0.04793	-0.40701	down
2.777947	2.5915	1.611141	2.223384	2.147605	0.047961	-0.32876	down
6.59376	6.829863	7.072793	7.463272	8.120665	0.04797	0.265917	up
34.83751	27.44432	33.55935	41.80166	32.34832	0.047974	0.268347	up
17.79255	17.76448	18.98203	23.76539	20.80969	0.04798	0.267803	up
83.62943	90.35214	75.9659	122.1862	111.0019	0.048012	0.346584	up
0.962678	0.52537	1.111866	1.876942	2.115688	0.048032	0.453956	up
0.685761	0.570268	0.650606	1.197587	1.197019	0.048096	0.431082	up
36.09676	35.93398	21.40723	28.56623	30.10646	0.048102	-0.28541	down
0.359815	0.535163	0.659396	0.524886	0.705234	0.048121	0.437033	up
67.80974	61.89335	53.09684	51.13025	48.8206	0.048133	-0.25198	down
10.35187	9.832052	9.889428	13.22393	13.57018	0.048148	0.286346	up
3.932768	3.91595	3.323515	5.41209	5.601056	0.04816	0.359971	up
1.432454	1.805659	1.440563	2.821048	2.097352	0.048217	0.405066	up
76.6188	83.69846	70.91809	46.51882	51.05229	0.048245	-0.3437	down
131.0622	133.0002	154.7105	154.8273	164.3796	0.048286	0.24553	up
52.1064	42.94718	53.03382	22.14765	28.01309	0.048308	-0.43398	down
20.55623	20.03492	18.47329	28.72489	25.83337	0.048333	0.317763	up
30.24612	28.36649	22.03743	21.96115	25.36544	0.048356	-0.26815	down
1.73559	1.7964	1.393545	3.202071	2.840869	0.048574	0.408738	up
1.676384	1.577219	1.573912	2.134489	2.349227	0.048632	0.345568	up
6.224512	6.345687	6.054082	7.477034	8.012763	0.048757	0.345388	up
12.66831	14.3114	8.711973	9.66779	10.13824	0.048774	-0.29364	down
8.028265	7.751936	7.544624	16.94866	12.38455	0.048789	0.399834	up
12.24996	12.82555	12.16583	15.78225	14.40952	0.048799	0.292957	up
12.56035	11.23639	10.65765	26.3064	17.28189	0.048856	0.416205	up
0.288349	0.34923	0.214829	0.550726	0.653091	0.048926	0.450442	up
3.958123	3.434301	2.98191	5.744455	7.741053	0.048983	0.434109	up
12.23451	14.71668	11.99376	17.5428	17.80786	0.049012	0.344248	up
12.08298	13.50078	9.180492	9.649724	9.938528	0.049306	-0.25318	down
10.74973	11.8055	8.190398	30.41789	19.64445	0.049361	0.443203	up
640.0033	733.501	334.5708	529.8975	511.7738	0.049461	-0.34614	down

11.56939	11.63176	8.25219	8.171721	8.733258	0.049496	-0.26915	down
1.715801	1.544226	2.705597	2.145063	2.164951	0.049551	0.382961	up
4.195751	4.505302	2.849976	3.105935	3.109909	0.049562	-0.2869	down
10.21898	9.271223	11.59908	10.81128	12.44156	0.049642	0.276363	up
0.433704	0.437471	0.434931	1.230754	1.050117	0.049681	0.44633	up
9.10877	9.858832	8.435668	13.52402	12.45332	0.049783	0.333091	up
37.58777	42.44061	24.52795	148.2594	124.1836	0.049828	0.418262	up
18.45395	20.05865	17.64767	37.02107	22.74129	0.049846	0.396412	up
34.64467	34.49749	25.64612	21.26035	23.74271	0.049914	-0.30734	down
61.34529	68.23922	46.43767	34.46462	33.55535	0.049932	-0.32289	down
27.88455	31.93704	29.7975	36.46869	38.50978	0.049981	0.28311	up
22.94102	22.85335	34.2173	24.21104	27.46137	0.049986	0.320371	up

Supplementary data 2

	p_val	avg_log2FC	pct.1	pct.2	p_val_adj
ONECUT2	3.24E-61	2.079758	1	0	8.63E-57
SLBP	1.08E-31	1.171029	0.864	0.181	2.88E-27
DUXAP8	2.10E-31	0.827558	0.772	0.083	5.60E-27
WDR72	4.24E-31	0.653862	0.777	0.076	1.13E-26
PSMC6	4.91E-30	1.014887	0.879	0.243	1.31E-25
TRIP11	9.30E-30	0.769279	0.85	0.153	2.48E-25
NELFA	9.84E-30	0.874796	0.825	0.16	2.62E-25
LETM1	7.92E-28	0.896266	0.83	0.188	2.11E-23
CCNK	1.14E-27	0.661829	0.786	0.146	3.03E-23
PPP6R3	1.34E-27	0.664364	0.811	0.174	3.56E-23
YY1	4.29E-27	0.947018	0.903	0.34	1.14E-22
EBLN3P	5.49E-27	0.702952	0.888	0.271	1.46E-22
ARFGEF2	6.12E-27	0.517123	0.733	0.104	1.63E-22
YWHAG	7.91E-27	0.528531	0.85	0.174	2.11E-22
PAPOLA	8.26E-27	1.154958	0.922	0.493	2.20E-22
UBAP2	1.40E-26	0.457697	0.816	0.146	3.72E-22
TC2N	1.49E-26	0.852213	0.869	0.264	3.98E-22
PFN2	1.58E-26	0.370801	0.675	0.056	4.20E-22
HSPD1	1.73E-26	1.49493	0.927	0.472	4.61E-22
ATP1B3	2.23E-26	0.439821	0.791	0.146	5.95E-22
UNC93B1	2.32E-26	0.915557	0.854	0.229	6.19E-22
ABHD17C	2.56E-26	0.861349	0.85	0.236	6.81E-22
SNRPA1	5.05E-26	0.670973	0.816	0.181	1.35E-21
AC139887.	5.79E-26	0.601989	0.583	0.014	1.54E-21
CTBP1	7.60E-26	0.93303	0.883	0.347	2.02E-21
CTAG2	9.40E-26	1.002658	0.786	0.139	2.50E-21
PCGF3	9.61E-26	0.727212	0.859	0.194	2.56E-21
POLR2J3	1.05E-25	0.810795	0.859	0.229	2.79E-21
RAB3IP	1.17E-25	1.000243	0.796	0.167	3.13E-21
GTPBP4	1.75E-25	0.567738	0.786	0.153	4.65E-21
LRP5	2.44E-25	0.647436	0.738	0.132	6.51E-21
HOXB6	3.10E-25	0.642111	0.646	0.049	8.27E-21
HDGFL3	4.68E-25	0.473589	0.704	0.083	1.25E-20
MEG8	4.69E-25	0.665606	0.636	0.056	1.25E-20
NORAD	5.13E-25	1.013751	0.898	0.354	1.37E-20
PARD6B	5.26E-25	0.845666	0.864	0.222	1.40E-20
ITPK1	6.19E-25	0.455603	0.806	0.16	1.65E-20
SMG1	8.85E-25	0.693789	0.859	0.229	2.36E-20
MAEA	9.19E-25	0.551466	0.874	0.236	2.45E-20
DICER1	1.12E-24	0.72909	0.791	0.174	2.98E-20
MAP4K5	1.22E-24	0.432364	0.733	0.132	3.26E-20
PIGG	2.14E-24	0.401346	0.762	0.132	5.70E-20
NSD2	2.61E-24	1.04404	0.791	0.201	6.95E-20
GAK	2.76E-24	1.047477	0.937	0.424	7.36E-20
FGFRL1	3.29E-24	0.412619	0.684	0.09	8.77E-20
SEZ6L2	8.23E-24	0.728052	0.777	0.167	2.19E-19
TOB1	8.26E-24	1.297137	0.893	0.354	2.20E-19
ADAM10	8.52E-24	0.518724	0.816	0.194	2.27E-19
SULF2	1.27E-23	0.580232	0.655	0.09	3.38E-19

RCN2	1.52E-23	0.646537	0.85	0.243	4.06E-19
PYCARD	1.78E-23	0.890674	0.908	0.299	4.76E-19
HOXB7	2.16E-23	0.367513	0.738	0.111	5.75E-19
KLF5	2.34E-23	0.890358	0.908	0.319	6.23E-19
EXOSC3	2.78E-23	0.272158	0.675	0.076	7.40E-19
TMX1	3.16E-23	0.450217	0.825	0.215	8.42E-19
DAAM1	3.67E-23	0.623292	0.801	0.208	9.77E-19
CLSTN1	4.04E-23	0.76472	0.82	0.222	1.08E-18
CLDN4	4.12E-23	1.206207	0.961	0.438	1.10E-18
PPFIA1	5.06E-23	0.473729	0.743	0.146	1.35E-18
RHPN2	6.45E-23	0.77663	0.816	0.215	1.72E-18
ZDHHC24	6.59E-23	0.383385	0.752	0.16	1.76E-18
POFUT1	6.79E-23	0.456985	0.534	0.021	1.81E-18
SDHAF3	6.86E-23	0.447426	0.641	0.083	1.83E-18
SETD3	6.96E-23	0.349426	0.709	0.111	1.85E-18
ATXN3	9.01E-23	0.5775	0.699	0.132	2.40E-18
CLMN	9.30E-23	0.645064	0.854	0.243	2.48E-18
PDZD8	1.07E-22	0.622228	0.782	0.181	2.86E-18
DDX27	1.18E-22	0.551439	0.816	0.215	3.15E-18
HOXB3	1.20E-22	0.488369	0.597	0.056	3.21E-18
DEGS2	1.61E-22	0.618786	0.801	0.194	4.30E-18
AGFG1	1.67E-22	0.502763	0.811	0.194	4.46E-18
ARHGAP5	1.71E-22	0.721062	0.874	0.285	4.54E-18
CEBPB	1.77E-22	0.974993	0.898	0.347	4.71E-18
KTN1	1.82E-22	1.027794	0.947	0.535	4.84E-18
PRKDC	1.94E-22	0.532049	0.699	0.132	5.17E-18
TMEM129	2.09E-22	0.553508	0.748	0.132	5.56E-18
RNF128	2.16E-22	0.491158	0.733	0.153	5.75E-18
TMEM265	2.43E-22	0.740081	0.728	0.146	6.48E-18
ADAM9	2.58E-22	0.756799	0.825	0.243	6.88E-18
TCEAL4	2.79E-22	0.382231	0.791	0.188	7.44E-18
RIN1	2.95E-22	0.389151	0.583	0.056	7.87E-18
CCS	2.97E-22	0.390598	0.879	0.292	7.90E-18
KIF5B	3.69E-22	0.804106	0.908	0.396	9.85E-18
SEC11A	4.44E-22	0.845386	0.903	0.389	1.18E-17
ARID4A	5.00E-22	0.601319	0.733	0.153	1.33E-17
NET1	5.09E-22	0.812119	0.903	0.333	1.36E-17
CPNE3	5.18E-22	0.466705	0.757	0.167	1.38E-17
THOC2	5.67E-22	0.660733	0.869	0.264	1.51E-17
TMEM176I	5.92E-22	0.793892	0.806	0.181	1.58E-17
KDM2A	6.04E-22	0.456332	0.811	0.222	1.61E-17
MYO1E	6.12E-22	0.52135	0.806	0.181	1.63E-17
AIP	6.16E-22	0.495511	0.777	0.174	1.64E-17
RPIA	6.72E-22	0.268547	0.684	0.111	1.79E-17
UBE2Q2	7.11E-22	0.382357	0.704	0.111	1.89E-17
PURA	7.25E-22	0.475894	0.752	0.167	1.93E-17
CDIPT	7.45E-22	0.361866	0.699	0.125	1.98E-17
CHD6	7.52E-22	0.530829	0.748	0.16	2.00E-17
TAF15	7.69E-22	0.386132	0.82	0.188	2.05E-17
CSNK2A1	7.71E-22	0.516807	0.82	0.229	2.05E-17

CTSH	7.91E-22	0.979573	0.927	0.444	2.11E-17
ANP32A	8.53E-22	0.697325	0.893	0.312	2.27E-17
ARMCX3	8.56E-22	0.627633	0.811	0.222	2.28E-17
AGTRAP	9.33E-22	1.073069	0.689	0.132	2.49E-17
CPNE1	1.02E-21	0.682197	0.869	0.306	2.71E-17
DPP3	1.04E-21	0.474151	0.718	0.139	2.77E-17
SGCB	1.15E-21	0.433461	0.631	0.083	3.07E-17
BUD23	1.18E-21	0.580596	0.883	0.299	3.13E-17
PSMA3	1.21E-21	0.846566	0.883	0.361	3.22E-17
CASK	1.22E-21	0.340404	0.748	0.167	3.26E-17
MRPL11	1.27E-21	0.837646	0.854	0.326	3.39E-17
PGRMC1	1.34E-21	0.670779	0.845	0.278	3.58E-17
CTBP1-DT	1.35E-21	0.386992	0.626	0.069	3.60E-17
GSKIP	1.45E-21	0.565156	0.84	0.25	3.87E-17
EDN1	1.69E-21	1.283454	0.66	0.118	4.50E-17
SERPINH1	1.93E-21	1.122155	0.714	0.16	5.14E-17
RCN1	1.95E-21	0.571183	0.796	0.208	5.20E-17
XPO1	2.02E-21	0.558304	0.752	0.167	5.39E-17
TOGARAM1	2.26E-21	0.374032	0.573	0.049	6.02E-17
CLDN3	2.56E-21	1.001052	0.908	0.312	6.83E-17
SLCO3A1	2.57E-21	0.591174	0.534	0.028	6.85E-17
HACD3	2.64E-21	0.815178	0.84	0.285	7.04E-17
ARIH1	2.69E-21	0.533839	0.84	0.222	7.17E-17
DNAJA1	2.86E-21	1.107494	0.947	0.535	7.63E-17
LAMP2	2.92E-21	0.617976	0.874	0.326	7.79E-17
SPR	3.07E-21	0.398392	0.723	0.146	8.18E-17
VPS4B	3.11E-21	0.443942	0.83	0.229	8.30E-17
URI1	3.37E-21	0.534969	0.82	0.243	8.99E-17
PDK4	3.39E-21	1.144399	0.733	0.174	9.04E-17
HSPH1	3.53E-21	0.921845	0.903	0.333	9.40E-17
PSMA3-AS1	3.60E-21	0.50651	0.811	0.25	9.60E-17
YAP1	3.75E-21	0.280425	0.641	0.104	9.98E-17
DPM1	3.76E-21	0.301504	0.723	0.132	1.00E-16
TMOD3	4.01E-21	0.552988	0.879	0.271	1.07E-16
CAMK2N1	4.05E-21	0.818774	0.927	0.347	1.08E-16
RNPS1	4.67E-21	0.582701	0.859	0.312	1.25E-16
PELI2	6.03E-21	0.520967	0.65	0.104	1.61E-16
FGFR3	6.36E-21	0.537631	0.646	0.097	1.70E-16
ERO1A	6.40E-21	0.775288	0.874	0.306	1.71E-16
CNIH1	7.54E-21	0.429028	0.859	0.285	2.01E-16
CAPN12	8.20E-21	0.479126	0.621	0.097	2.19E-16
UBE2R2	1.27E-20	0.682932	0.874	0.347	3.38E-16
TRAPPC6A	1.47E-20	0.471187	0.801	0.201	3.92E-16
DGKQ	1.57E-20	0.398772	0.743	0.16	4.18E-16
CLTA	1.58E-20	0.989503	0.927	0.583	4.21E-16
MAPK6	1.59E-20	0.493379	0.811	0.229	4.24E-16
ASPH	1.98E-20	0.714819	0.85	0.312	5.27E-16
ZFAND6	2.21E-20	0.551049	0.879	0.312	5.89E-16
BTBD7	2.29E-20	0.437404	0.791	0.208	6.09E-16
MAPK1IP1L	2.79E-20	0.540544	0.883	0.319	7.45E-16

SYNCRIP	2.85E-20	0.546915	0.845	0.236	7.61E-16
GCC2	3.26E-20	0.77709	0.932	0.41	8.68E-16
FOXP4	3.35E-20	0.465066	0.631	0.111	8.92E-16
RANBP2	3.81E-20	0.314646	0.733	0.16	1.02E-15
KLHDC2	3.95E-20	0.578581	0.811	0.243	1.05E-15
WDR61	4.15E-20	0.334431	0.782	0.201	1.11E-15
ALG13	4.17E-20	0.32248	0.777	0.201	1.11E-15
HM13	4.17E-20	0.697599	0.913	0.465	1.11E-15
SLC25A33	4.41E-20	0.567564	0.66	0.132	1.18E-15
STIP1	4.50E-20	0.54007	0.767	0.208	1.20E-15
PLEKHA1	4.82E-20	0.508393	0.796	0.215	1.29E-15
RGP1	5.37E-20	0.397658	0.641	0.111	1.43E-15
MACC1	5.72E-20	0.798753	0.738	0.194	1.52E-15
RETREG1	6.35E-20	0.624079	0.709	0.153	1.69E-15
SAV1	6.87E-20	0.439873	0.563	0.069	1.83E-15
ABHD11	6.93E-20	0.464748	0.786	0.215	1.85E-15
SSRP1	7.38E-20	0.420168	0.694	0.146	1.97E-15
TMC5	7.42E-20	1.163995	0.947	0.528	1.98E-15
KDSR	7.87E-20	0.480623	0.689	0.16	2.10E-15
RAD21	7.93E-20	0.583227	0.864	0.299	2.11E-15
REST	8.09E-20	0.43244	0.67	0.132	2.16E-15
PRKCI	8.75E-20	0.57585	0.772	0.222	2.33E-15
HINT2	9.41E-20	0.402241	0.913	0.368	2.51E-15
TOMM40	9.42E-20	0.492091	0.699	0.16	2.51E-15
UBAP1	9.55E-20	0.415379	0.612	0.097	2.54E-15
OSER1	1.02E-19	0.536821	0.757	0.208	2.71E-15
LERFS	1.02E-19	0.341126	0.456	0.007	2.72E-15
FMR1	1.03E-19	0.382494	0.631	0.104	2.74E-15
NIFK	1.05E-19	0.508043	0.723	0.174	2.80E-15
GOLGA5	1.11E-19	0.372625	0.738	0.181	2.95E-15
SERINC3	1.11E-19	0.582864	0.869	0.292	2.95E-15
LAPTM4B	1.25E-19	0.458641	0.796	0.215	3.34E-15
LBR	1.38E-19	0.36153	0.646	0.118	3.69E-15
SMCHD1	1.48E-19	0.385789	0.864	0.257	3.95E-15
ARID1B	1.50E-19	0.474901	0.82	0.243	4.00E-15
SKAP2	1.61E-19	0.330704	0.704	0.139	4.29E-15
ACSL3	1.78E-19	0.562058	0.811	0.25	4.75E-15
RMND5A	1.79E-19	0.300641	0.65	0.111	4.76E-15
TARDBP	1.82E-19	0.496852	0.801	0.229	4.84E-15
NCEH1	1.88E-19	0.465063	0.66	0.125	5.02E-15
NECTIN2	1.92E-19	0.601728	0.898	0.312	5.11E-15
GPC3	2.05E-19	0.702296	0.519	0.042	5.46E-15
STK3	2.07E-19	0.344417	0.553	0.062	5.52E-15
NR2F2	2.11E-19	0.593604	0.801	0.236	5.62E-15
SMC1A	2.11E-19	0.28754	0.68	0.146	5.62E-15
TRIM28	2.14E-19	0.566311	0.874	0.34	5.72E-15
PHF3	2.22E-19	0.321438	0.752	0.194	5.92E-15
STAG2	2.23E-19	0.351542	0.786	0.236	5.95E-15
CPSF2	2.27E-19	0.462011	0.641	0.132	6.04E-15
SRSF10	2.28E-19	0.486807	0.879	0.326	6.07E-15

HUWE1	2.37E-19	0.39953	0.767	0.188	6.31E-15
CNOT7	2.40E-19	0.442527	0.718	0.174	6.41E-15
PAX8-AS1	2.52E-19	0.467815	0.573	0.069	6.71E-15
CCNT2	2.54E-19	0.41209	0.617	0.104	6.78E-15
SERBP1	2.61E-19	0.805072	0.913	0.444	6.97E-15
CCDC59	2.65E-19	0.323265	0.762	0.201	7.06E-15
FAM210B	2.98E-19	0.513243	0.743	0.194	7.95E-15
KMT5B	3.01E-19	0.359972	0.757	0.174	8.03E-15
ASAH1	3.43E-19	0.581457	0.859	0.306	9.14E-15
USP3	3.62E-19	0.312741	0.816	0.229	9.65E-15
PRPF6	3.68E-19	0.412877	0.816	0.236	9.81E-15
B4GALT1	3.74E-19	0.580405	0.85	0.299	9.97E-15
NFX1	3.88E-19	0.481436	0.617	0.111	1.03E-14
CERS6	4.06E-19	0.519654	0.738	0.174	1.08E-14
XRN2	4.20E-19	0.47517	0.85	0.278	1.12E-14
TSN	4.53E-19	0.314563	0.714	0.16	1.21E-14
FTX	4.61E-19	0.510178	0.636	0.104	1.23E-14
SNRPB2	4.72E-19	0.437338	0.864	0.299	1.26E-14
BROX	4.87E-19	0.31987	0.699	0.153	1.30E-14
NMI	5.12E-19	0.253044	0.714	0.153	1.36E-14
ZNF580	5.21E-19	0.379738	0.617	0.104	1.39E-14
NELFCD	5.25E-19	0.400654	0.68	0.146	1.40E-14
TASOR2	5.40E-19	0.352728	0.602	0.09	1.44E-14
TMEM251	5.42E-19	0.299541	0.733	0.181	1.44E-14
MED13L	5.56E-19	0.50607	0.767	0.229	1.48E-14
PAK4	5.57E-19	0.312983	0.505	0.035	1.48E-14
RPP25L	5.60E-19	0.340208	0.709	0.167	1.49E-14
THUMPD1	5.63E-19	0.401092	0.728	0.181	1.50E-14
GMCL1	5.87E-19	0.348049	0.612	0.09	1.56E-14
TMPO	5.91E-19	0.639121	0.811	0.257	1.58E-14
RBM39	5.98E-19	0.808746	0.951	0.556	1.59E-14
SRSF6	6.35E-19	0.514974	0.738	0.215	1.69E-14
NDRG1	6.56E-19	0.579132	0.825	0.278	1.75E-14
WAC	6.59E-19	0.415963	0.845	0.299	1.76E-14
CCDC107	6.69E-19	0.366861	0.883	0.347	1.78E-14
NOC2L	6.94E-19	0.492367	0.704	0.181	1.85E-14
PRPF40A	7.10E-19	0.618335	0.903	0.34	1.89E-14
MOAP1	7.47E-19	0.307352	0.592	0.083	1.99E-14
ATP2A2	7.90E-19	0.441711	0.801	0.236	2.10E-14
SEPTIN2	7.94E-19	0.654862	0.869	0.361	2.12E-14
VCP	8.39E-19	0.772713	0.888	0.403	2.24E-14
SMU1	8.69E-19	0.256097	0.786	0.201	2.31E-14
MT-ND2	8.69E-19	1.043739	0.976	0.819	2.32E-14
TLN1	8.71E-19	0.516427	0.85	0.285	2.32E-14
YES1	8.97E-19	0.291578	0.607	0.104	2.39E-14
IDH3B	9.01E-19	0.364305	0.699	0.153	2.40E-14
SOWAHC	9.44E-19	0.470944	0.597	0.097	2.52E-14
C19orf48	1.09E-18	0.371434	0.597	0.097	2.90E-14
C1D	1.09E-18	0.350208	0.723	0.181	2.92E-14
C9orf72	1.10E-18	0.367012	0.495	0.035	2.94E-14

VAPA	1.14E-18	0.675099	0.942	0.493	3.04E-14
STX16	1.15E-18	0.382354	0.767	0.208	3.07E-14
PRSS22	1.18E-18	0.747426	0.767	0.229	3.13E-14
CREBRF	1.20E-18	0.450395	0.67	0.132	3.21E-14
YDJC	1.28E-18	0.381627	0.709	0.174	3.40E-14
TMPRSS2	1.29E-18	0.354373	0.874	0.292	3.43E-14
BFAR	1.29E-18	0.384735	0.743	0.201	3.43E-14
LRPPRC	1.39E-18	0.42468	0.733	0.188	3.70E-14
PIAS1	1.39E-18	0.362779	0.767	0.215	3.70E-14
PUM1	1.49E-18	0.510845	0.816	0.257	3.98E-14
RERE	1.51E-18	0.45385	0.811	0.25	4.01E-14
ATP6AP1	1.76E-18	0.403111	0.835	0.292	4.69E-14
HNRNPH2	1.79E-18	0.30798	0.782	0.215	4.78E-14
NDUFS1	1.82E-18	0.411416	0.743	0.215	4.84E-14
EMP2	1.90E-18	0.550483	0.767	0.215	5.05E-14
ZMYND11	1.90E-18	0.338414	0.68	0.153	5.07E-14
HSPE1	1.94E-18	0.984214	0.937	0.569	5.16E-14
PFKP	1.96E-18	0.447817	0.767	0.201	5.22E-14
FLRT3	1.96E-18	0.523395	0.515	0.056	5.23E-14
KIF3B	2.04E-18	0.287184	0.811	0.243	5.43E-14
GPR108	2.14E-18	0.402808	0.694	0.16	5.70E-14
EPB41L1	2.16E-18	0.541933	0.694	0.167	5.75E-14
DNAJA4	2.19E-18	0.603373	0.733	0.201	5.82E-14
ATP2B1	2.23E-18	0.459353	0.806	0.264	5.95E-14
TM9SF1	2.39E-18	0.394649	0.694	0.167	6.37E-14
USP34	2.53E-18	0.405123	0.757	0.215	6.74E-14
PEPD	2.58E-18	0.375961	0.796	0.25	6.87E-14
APPL2	3.01E-18	0.339317	0.65	0.139	8.02E-14
ANKIB1	3.14E-18	0.310695	0.631	0.104	8.36E-14
ZADH2	3.17E-18	0.25932	0.699	0.153	8.46E-14
ZNF664	3.21E-18	0.364679	0.665	0.153	8.56E-14
CAPRIN1	3.22E-18	0.342306	0.786	0.222	8.57E-14
ARL5A	3.31E-18	0.273288	0.796	0.222	8.81E-14
ZNF267	3.32E-18	0.395705	0.583	0.09	8.86E-14
PPP1R12A	3.46E-18	0.466115	0.791	0.229	9.21E-14
DCAF12	3.53E-18	0.398548	0.66	0.146	9.40E-14
SNX6	3.55E-18	0.486578	0.83	0.271	9.46E-14
EIF4A3	3.72E-18	0.546701	0.796	0.25	9.90E-14
TOMM5	4.02E-18	0.549223	0.917	0.417	1.07E-13
H6PD	4.10E-18	0.65214	0.631	0.153	1.09E-13
KNOP1	4.20E-18	0.411722	0.777	0.215	1.12E-13
SLC20A1	4.27E-18	0.728368	0.801	0.285	1.14E-13
SSB	4.43E-18	0.517326	0.879	0.34	1.18E-13
MESD	4.51E-18	0.507152	0.854	0.292	1.20E-13
MTUS1	4.53E-18	0.460989	0.777	0.25	1.21E-13
PRELID3B	4.56E-18	0.348536	0.84	0.271	1.22E-13
TMEM97	4.70E-18	0.54215	0.65	0.139	1.25E-13
TMEM179E	4.72E-18	0.503671	0.85	0.333	1.26E-13
CD2AP	4.83E-18	0.578652	0.917	0.312	1.29E-13
ATP1B1	4.95E-18	1.150669	0.947	0.569	1.32E-13

CHORDC1	5.04E-18	0.547222	0.762	0.229	1.34E-13
NONO	5.07E-18	0.427043	0.854	0.271	1.35E-13
GALT	5.27E-18	0.323774	0.558	0.076	1.41E-13
PCMTD2	5.28E-18	0.312064	0.485	0.042	1.41E-13
DDIT3	5.44E-18	0.656733	0.67	0.153	1.45E-13
HSD17B2	5.67E-18	0.389764	0.655	0.146	1.51E-13
SLC25A37	6.00E-18	0.631844	0.854	0.292	1.60E-13
RSL24D1	6.06E-18	0.634726	0.879	0.361	1.61E-13
FOXJ3	6.07E-18	0.339821	0.519	0.069	1.62E-13
ACTR10	6.33E-18	0.429423	0.816	0.271	1.69E-13
PAWR	6.40E-18	0.429662	0.845	0.292	1.71E-13
TCF12	6.44E-18	0.297918	0.641	0.125	1.72E-13
ZNF587	7.10E-18	0.400928	0.641	0.132	1.89E-13
JPX	7.18E-18	0.402651	0.709	0.167	1.91E-13
TOP1	7.35E-18	0.540884	0.893	0.396	1.96E-13
RAMP1	7.44E-18	0.58029	0.777	0.222	1.98E-13
RALBP1	7.64E-18	0.659101	0.917	0.417	2.04E-13
HDAC2	7.97E-18	0.323182	0.743	0.194	2.12E-13
SYS1	8.15E-18	0.363912	0.68	0.16	2.17E-13
PHF20	8.52E-18	0.318984	0.748	0.201	2.27E-13
TMEM33	8.95E-18	0.444367	0.738	0.215	2.39E-13
SH3RF1	9.03E-18	0.417474	0.694	0.146	2.41E-13
CAB39	9.18E-18	0.282792	0.728	0.201	2.45E-13
ZNF280D	9.21E-18	0.301736	0.65	0.132	2.46E-13
MLEC	9.23E-18	0.717067	0.888	0.431	2.46E-13
ADIPOR2	9.29E-18	0.362487	0.66	0.146	2.48E-13
DDX24	9.34E-18	0.726985	0.927	0.458	2.49E-13
ZNF217	9.35E-18	0.384331	0.587	0.104	2.49E-13
EXOC5	9.38E-18	0.308359	0.646	0.125	2.50E-13
UBE2Z	9.68E-18	0.417161	0.694	0.181	2.58E-13
PYGB	1.03E-17	0.3104	0.801	0.236	2.74E-13
BMI1	1.03E-17	0.268406	0.587	0.097	2.74E-13
ABI2	1.04E-17	0.280694	0.641	0.118	2.76E-13
SMC4	1.04E-17	0.409844	0.641	0.118	2.78E-13
PMAIP1	1.05E-17	0.612215	0.796	0.264	2.80E-13
GABRB3	1.05E-17	0.334784	0.456	0.028	2.80E-13
TMED5	1.07E-17	0.388745	0.684	0.174	2.85E-13
XIAP	1.08E-17	0.44256	0.767	0.229	2.87E-13
ICE2	1.12E-17	0.276885	0.573	0.083	2.99E-13
LMAN1	1.12E-17	0.617331	0.898	0.396	3.00E-13
TMX2	1.20E-17	0.307186	0.709	0.167	3.19E-13
SF3B1	1.21E-17	0.535204	0.922	0.403	3.24E-13
DCTPP1	1.22E-17	0.453512	0.767	0.222	3.24E-13
GTF2A2	1.25E-17	0.582921	0.854	0.319	3.33E-13
SIGMAR1	1.28E-17	0.511297	0.709	0.194	3.40E-13
FOXP1	1.34E-17	0.692475	0.908	0.41	3.57E-13
HSD17B12	1.34E-17	0.380773	0.772	0.229	3.58E-13
LENG1	1.35E-17	0.347782	0.573	0.09	3.60E-13
EHF	1.42E-17	0.35109	0.704	0.181	3.79E-13
CLK3	1.44E-17	0.349786	0.655	0.139	3.84E-13

NOP56	1.50E-17	0.353859	0.704	0.188	4.00E-13
POLR3E	1.51E-17	0.297643	0.539	0.069	4.01E-13
ADRM1	1.51E-17	0.643206	0.874	0.375	4.02E-13
NDUFB6	1.53E-17	0.590999	0.874	0.382	4.09E-13
SEPTIN10	1.54E-17	0.316587	0.621	0.111	4.11E-13
WDR33	1.68E-17	0.348222	0.733	0.188	4.47E-13
SERPINB5	1.69E-17	0.255517	0.684	0.153	4.50E-13
TTC14	1.70E-17	0.408503	0.641	0.132	4.54E-13
UGDH	1.86E-17	0.609843	0.718	0.215	4.96E-13
FKBP9	1.97E-17	0.345381	0.617	0.118	5.24E-13
IMP3	1.97E-17	0.587268	0.825	0.299	5.25E-13
SYNE2	1.99E-17	1.020365	0.864	0.354	5.32E-13
PGD	2.06E-17	0.913616	0.801	0.278	5.48E-13
TMEM175	2.07E-17	0.394326	0.665	0.167	5.51E-13
GRHPR	2.10E-17	0.490121	0.869	0.326	5.59E-13
PTP4A2	2.20E-17	0.788425	0.951	0.507	5.86E-13
KLHL28	2.21E-17	0.306882	0.476	0.042	5.90E-13
KRT7	2.23E-17	0.582318	0.825	0.257	5.94E-13
PON2	2.35E-17	0.36624	0.728	0.201	6.25E-13
DCAF13	2.48E-17	0.280309	0.631	0.132	6.60E-13
SERPINA4	2.48E-17	0.532407	0.495	0.056	6.61E-13
PMEPA1	2.49E-17	0.779989	0.796	0.278	6.63E-13
PIGT	2.59E-17	0.49924	0.893	0.368	6.91E-13
NCL	2.76E-17	0.631357	0.932	0.479	7.35E-13
DNAJC7	2.78E-17	0.325421	0.835	0.264	7.41E-13
RBM42	2.78E-17	0.364729	0.762	0.215	7.41E-13
PIP5K1B	2.82E-17	0.310818	0.689	0.188	7.50E-13
DHX9	2.83E-17	0.294968	0.762	0.215	7.55E-13
THRA	2.95E-17	0.256821	0.65	0.139	7.85E-13
TGIF1	2.97E-17	0.57274	0.777	0.229	7.91E-13
YIPF6	3.09E-17	0.345847	0.743	0.201	8.24E-13
UBE2I	3.16E-17	0.470227	0.869	0.278	8.41E-13
APPL1	3.16E-17	0.332743	0.704	0.174	8.42E-13
DFFA	3.20E-17	0.518133	0.675	0.174	8.53E-13
PRKAA1	3.25E-17	0.266756	0.699	0.174	8.66E-13
NBR1	3.33E-17	0.35948	0.67	0.16	8.87E-13
SOX9	3.34E-17	0.771696	0.85	0.306	8.90E-13
TMEM165	3.36E-17	0.500528	0.825	0.299	8.94E-13
HSP90AB1	3.38E-17	0.76759	0.976	0.764	9.01E-13
APMAP	3.41E-17	0.387607	0.723	0.194	9.08E-13
ZKSCAN1	3.41E-17	0.417626	0.898	0.326	9.09E-13
ORC4	3.55E-17	0.277963	0.592	0.104	9.47E-13
KRR1	3.57E-17	0.320469	0.684	0.181	9.50E-13
MTDH	3.58E-17	0.687075	0.932	0.542	9.55E-13
LZIC	3.66E-17	0.57797	0.675	0.188	9.74E-13
TSEN34	3.83E-17	0.349896	0.723	0.194	1.02E-12
STOML2	3.94E-17	0.6179	0.864	0.382	1.05E-12
CCT4	3.94E-17	0.566443	0.874	0.347	1.05E-12
RB1CC1	3.98E-17	0.39135	0.786	0.229	1.06E-12
RBM4	4.17E-17	0.646959	0.903	0.417	1.11E-12

SLC52A2	4.29E-17	0.303568	0.66	0.153	1.14E-12
IRF2BP2	4.51E-17	0.660308	0.908	0.417	1.20E-12
LYPD2	4.63E-17	0.988371	0.534	0.069	1.23E-12
ETHE1	4.80E-17	0.52757	0.811	0.285	1.28E-12
ITGB1	4.81E-17	0.649104	0.913	0.438	1.28E-12
MANBAL	5.07E-17	0.252732	0.675	0.16	1.35E-12
SRSF1	5.08E-17	0.371936	0.806	0.243	1.35E-12
GMFB	5.17E-17	0.270708	0.689	0.167	1.38E-12
BRMS1	5.30E-17	0.439902	0.84	0.292	1.41E-12
GPRC5C	5.54E-17	0.442652	0.714	0.188	1.48E-12
GLOD4	5.67E-17	0.289912	0.684	0.167	1.51E-12
NECTIN3	5.69E-17	0.264209	0.684	0.16	1.52E-12
CCT2	5.80E-17	0.628055	0.874	0.361	1.55E-12
ARPP19	5.85E-17	0.348146	0.835	0.306	1.56E-12
PRR15L	6.07E-17	0.491265	0.786	0.257	1.62E-12
NKAP	6.19E-17	0.378311	0.68	0.167	1.65E-12
ZNF518A	6.21E-17	0.366548	0.612	0.132	1.66E-12
PRSS3	6.40E-17	0.770985	0.932	0.472	1.71E-12
KIF1B	6.69E-17	0.460503	0.621	0.139	1.78E-12
HOXB8	6.71E-17	0.284294	0.466	0.035	1.79E-12
TBC1D15	6.77E-17	0.321036	0.626	0.139	1.81E-12
ADD3	6.93E-17	0.337812	0.835	0.285	1.85E-12
ZCCHC7	7.02E-17	0.26709	0.67	0.16	1.87E-12
VMA21	7.08E-17	0.305046	0.684	0.167	1.89E-12
UBIAD1	7.19E-17	0.584356	0.529	0.083	1.92E-12
PRKCA	7.25E-17	0.410289	0.694	0.167	1.93E-12
ISG20L2	7.50E-17	0.393523	0.646	0.153	2.00E-12
SELENOS	7.76E-17	0.679146	0.927	0.521	2.07E-12
NUFIP2	7.76E-17	0.417971	0.728	0.229	2.07E-12
MT-ND4	7.80E-17	0.819586	0.995	0.903	2.08E-12
POLD3	7.89E-17	0.392982	0.544	0.09	2.10E-12
GCNT3	7.92E-17	0.55263	0.796	0.271	2.11E-12
RAB22A	8.01E-17	0.347075	0.723	0.188	2.13E-12
ATRX	8.05E-17	0.543554	0.874	0.368	2.14E-12
UNC13B	8.23E-17	0.266931	0.544	0.09	2.19E-12
EXOSC10	8.27E-17	0.42035	0.568	0.09	2.20E-12
ZNF787	8.36E-17	0.253256	0.757	0.222	2.23E-12
HNRNPF	8.56E-17	0.602485	0.883	0.424	2.28E-12
ATP8B1	8.86E-17	0.358735	0.811	0.285	2.36E-12
HSPBP1	9.10E-17	0.312289	0.636	0.146	2.43E-12
EFNB1	9.10E-17	0.340322	0.636	0.146	2.43E-12
HP1BP3	9.15E-17	0.523342	0.879	0.34	2.44E-12
KCNK15	9.59E-17	0.536335	0.524	0.069	2.56E-12
PTGES3	9.87E-17	0.680682	0.927	0.451	2.63E-12
RBM17	1.01E-16	0.543313	0.845	0.306	2.69E-12
LYPLA1	1.01E-16	0.458258	0.806	0.264	2.70E-12
ATF2	1.03E-16	0.270035	0.578	0.097	2.75E-12
YEATS4	1.03E-16	0.267664	0.563	0.09	2.75E-12
RNF38	1.05E-16	0.308619	0.631	0.139	2.79E-12
VPS13C	1.05E-16	0.437125	0.752	0.229	2.80E-12

CDK6	1.07E-16	0.459493	0.621	0.146	2.84E-12
MRPS26	1.07E-16	0.327486	0.694	0.174	2.86E-12
CHMP4B	1.09E-16	0.299128	0.854	0.312	2.90E-12
TRIM56	1.10E-16	0.392578	0.806	0.278	2.93E-12
CTSA	1.10E-16	0.425374	0.854	0.319	2.93E-12
PDCD7	1.11E-16	0.286901	0.67	0.16	2.95E-12
EFHC1	1.11E-16	0.307963	0.558	0.09	2.97E-12
FTL	1.12E-16	0.77907	0.985	0.924	2.99E-12
MYO1B	1.20E-16	0.312191	0.461	0.042	3.20E-12
SGPP2	1.20E-16	0.324753	0.786	0.25	3.21E-12
FAM120AC	1.23E-16	0.443416	0.806	0.25	3.27E-12
EIF2S2	1.24E-16	0.59054	0.922	0.486	3.29E-12
AC092868.	1.24E-16	0.405779	0.553	0.097	3.31E-12
USP7	1.24E-16	0.402414	0.718	0.222	3.31E-12
C4orf48	1.26E-16	0.713145	0.84	0.306	3.36E-12
TMEM87B	1.38E-16	0.25569	0.68	0.181	3.67E-12
SIN3A	1.38E-16	0.348913	0.573	0.104	3.69E-12
APEX1	1.41E-16	0.506968	0.85	0.34	3.76E-12
MAIP1	1.44E-16	0.292305	0.485	0.056	3.83E-12
NFE2L2	1.46E-16	0.434872	0.917	0.375	3.88E-12
ZC3H18	1.48E-16	0.348141	0.505	0.069	3.95E-12
TP53I13	1.50E-16	0.356266	0.655	0.16	4.00E-12
ERBB3	1.52E-16	0.642399	0.913	0.389	4.06E-12
QSER1	1.65E-16	0.309792	0.481	0.056	4.40E-12
B4GALT5	1.71E-16	0.401996	0.757	0.208	4.57E-12
CCT6A	1.73E-16	0.477016	0.859	0.306	4.60E-12
SEMA4B	1.84E-16	0.426185	0.665	0.16	4.91E-12
MIIP	1.84E-16	0.59265	0.534	0.083	4.91E-12
MYO9A	1.85E-16	0.317172	0.563	0.097	4.93E-12
MYL9	1.96E-16	0.673174	0.621	0.139	5.22E-12
YTHDF1	1.96E-16	0.295241	0.578	0.111	5.23E-12
DEK	1.99E-16	0.519496	0.908	0.403	5.29E-12
TRMT10C	1.99E-16	0.303579	0.578	0.104	5.30E-12
LAGE3	2.01E-16	0.331477	0.709	0.215	5.35E-12
HPRT1	2.04E-16	0.422501	0.65	0.174	5.43E-12
SCARB2	2.05E-16	0.469644	0.748	0.236	5.47E-12
KLHL25	2.11E-16	0.40521	0.5	0.069	5.63E-12
CCND1	2.12E-16	0.809232	0.869	0.417	5.64E-12
MINDY2	2.13E-16	0.299222	0.67	0.153	5.68E-12
FKBP4	2.14E-16	0.362379	0.762	0.215	5.70E-12
CARHSP1	2.23E-16	0.475333	0.752	0.229	5.95E-12
NEMF	2.25E-16	0.337628	0.777	0.25	6.01E-12
CKS2	2.32E-16	0.463666	0.621	0.139	6.18E-12
PTPN12	2.33E-16	0.274499	0.655	0.153	6.22E-12
ZNF264	2.34E-16	0.251002	0.49	0.062	6.23E-12
ATG14	2.35E-16	0.366785	0.481	0.056	6.26E-12
RIF1	2.40E-16	0.356034	0.689	0.188	6.40E-12
TOPORS	2.61E-16	0.314191	0.631	0.132	6.97E-12
EHBP1	2.65E-16	0.304593	0.485	0.049	7.06E-12
YWHAB	2.66E-16	0.587211	0.976	0.701	7.09E-12

ADNP	2.73E-16	0.363161	0.67	0.174	7.28E-12
MAPRE1	2.75E-16	0.305912	0.82	0.285	7.34E-12
MTG2	2.76E-16	0.291004	0.505	0.069	7.37E-12
CACYBP	2.80E-16	0.496343	0.806	0.285	7.47E-12
EIF6	3.11E-16	0.607502	0.922	0.431	8.28E-12
FNBP1L	3.15E-16	0.285315	0.689	0.167	8.40E-12
ACTL6A	3.23E-16	0.287898	0.583	0.104	8.62E-12
LEO1	3.43E-16	0.261402	0.646	0.146	9.14E-12
USF2	3.59E-16	0.299944	0.796	0.236	9.57E-12
BMP2	3.88E-16	0.483631	0.621	0.132	1.03E-11
DKC1	3.96E-16	0.264084	0.675	0.174	1.06E-11
LTBR	4.12E-16	0.291431	0.748	0.194	1.10E-11
CPT1A	4.12E-16	0.411596	0.675	0.174	1.10E-11
KIAA2013	4.17E-16	0.767724	0.743	0.264	1.11E-11
GNAI2	4.29E-16	0.275025	0.796	0.257	1.14E-11
PAK2	4.38E-16	0.31081	0.791	0.278	1.17E-11
NME3	4.43E-16	0.401612	0.811	0.306	1.18E-11
RCE1	4.63E-16	0.264218	0.607	0.118	1.23E-11
OPA1	5.09E-16	0.293935	0.5	0.069	1.36E-11
RPN2	5.13E-16	0.736456	0.917	0.535	1.37E-11
SPSB1	5.30E-16	0.358765	0.485	0.062	1.41E-11
DNTTIP2	5.49E-16	0.293476	0.636	0.132	1.46E-11
PKM	5.53E-16	0.896882	0.947	0.514	1.47E-11
IARS2	6.04E-16	0.387568	0.675	0.174	1.61E-11
SUPT16H	6.16E-16	0.362721	0.704	0.201	1.64E-11
MRPL18	6.25E-16	0.282764	0.816	0.292	1.67E-11
RPL7L1	6.39E-16	0.357417	0.723	0.215	1.70E-11
CDK2AP2	6.56E-16	0.525726	0.908	0.431	1.75E-11
DMAC2L	6.62E-16	0.304163	0.65	0.146	1.76E-11
PLCB1	6.66E-16	0.284832	0.447	0.042	1.78E-11
ECH1	6.71E-16	0.572254	0.883	0.451	1.79E-11
PARN	6.71E-16	0.260414	0.544	0.083	1.79E-11
ATG2B	6.78E-16	0.255577	0.5	0.062	1.81E-11
TMPRSS4	7.06E-16	0.33865	0.665	0.167	1.88E-11
C2orf49	7.11E-16	0.260729	0.573	0.111	1.89E-11
AC011287.	7.19E-16	0.310423	0.398	0.014	1.92E-11
SEC23A	7.39E-16	0.37769	0.66	0.167	1.97E-11
PFKFB2	7.80E-16	0.250343	0.587	0.111	2.08E-11
AGO2	8.03E-16	0.345442	0.636	0.146	2.14E-11
PPP1R10	8.16E-16	0.370568	0.65	0.16	2.18E-11
CANX	8.35E-16	0.606887	0.893	0.403	2.23E-11
ZNHIT3	8.43E-16	0.291249	0.675	0.174	2.25E-11
TDG	8.67E-16	0.293488	0.738	0.215	2.31E-11
DEDD2	8.70E-16	0.388367	0.699	0.201	2.32E-11
SLC25A29	8.86E-16	0.257689	0.592	0.118	2.36E-11
WWP1	8.98E-16	0.322273	0.665	0.174	2.39E-11
EBAG9	9.04E-16	0.273722	0.67	0.188	2.41E-11
ARL2	9.23E-16	0.391381	0.743	0.236	2.46E-11
TMEM134	9.34E-16	0.409755	0.825	0.326	2.49E-11
GEM	9.35E-16	0.415571	0.49	0.062	2.49E-11

ZNRD2	9.36E-16	0.272315	0.675	0.174	2.50E-11
CEBPG	9.91E-16	0.393531	0.738	0.25	2.64E-11
SEMA3C	1.00E-15	0.306551	0.515	0.083	2.67E-11
PWP1	1.02E-15	0.257897	0.675	0.174	2.73E-11
MFSD4A	1.02E-15	0.256458	0.476	0.062	2.73E-11
BAG1	1.05E-15	0.596966	0.903	0.438	2.79E-11
MOB1A	1.09E-15	0.429008	0.869	0.326	2.90E-11
SH2D4A	1.09E-15	0.281563	0.714	0.194	2.91E-11
CRLS1	1.10E-15	0.298495	0.66	0.188	2.92E-11
DDX54	1.10E-15	0.29608	0.646	0.16	2.92E-11
TEX261	1.10E-15	0.250635	0.549	0.09	2.94E-11
TNFRSF21	1.11E-15	0.484045	0.738	0.222	2.95E-11
YIF1A	1.14E-15	0.381639	0.816	0.312	3.04E-11
TMEM147	1.14E-15	0.289389	0.83	0.299	3.04E-11
UBA2	1.14E-15	0.275052	0.641	0.167	3.05E-11
HOXB9	1.18E-15	0.293125	0.408	0.021	3.16E-11
FOXC1	1.19E-15	0.407911	0.471	0.056	3.17E-11
RAB6A	1.20E-15	0.366242	0.845	0.278	3.18E-11
MBOAT7	1.20E-15	0.264481	0.592	0.139	3.20E-11
BUB3	1.24E-15	0.390574	0.757	0.25	3.30E-11
DMBT1	1.24E-15	1.009843	0.612	0.146	3.32E-11
CREB3	1.26E-15	0.409431	0.782	0.25	3.36E-11
PPHLN1	1.36E-15	0.298219	0.655	0.16	3.62E-11
PAM16	1.38E-15	0.340997	0.772	0.236	3.67E-11
PTPRK	1.39E-15	0.289231	0.733	0.215	3.70E-11
CHTOP	1.48E-15	0.303111	0.694	0.188	3.95E-11
PSMD14	1.51E-15	0.383558	0.757	0.257	4.02E-11
GLS	1.54E-15	0.315649	0.655	0.16	4.09E-11
NARS	1.55E-15	0.382049	0.835	0.326	4.13E-11
BUD31	1.59E-15	0.297319	0.888	0.333	4.23E-11
IRF3	1.59E-15	0.393127	0.82	0.292	4.24E-11
CCDC47	1.59E-15	0.380324	0.825	0.312	4.24E-11
CBFA2T2	1.68E-15	0.280154	0.553	0.104	4.48E-11
PPP4R3A	1.73E-15	0.267229	0.801	0.285	4.62E-11
SLC1A5	1.78E-15	0.303282	0.626	0.167	4.75E-11
UHMK1	1.79E-15	0.293155	0.704	0.208	4.77E-11
ARL4A	1.80E-15	0.382788	0.748	0.25	4.80E-11
TIMM9	1.81E-15	0.296361	0.743	0.229	4.82E-11
MED19	1.87E-15	0.339437	0.684	0.188	4.99E-11
ZNF768	1.90E-15	0.297109	0.51	0.076	5.07E-11
CCT3	1.97E-15	0.510212	0.874	0.361	5.24E-11
CLCN6	1.98E-15	0.297404	0.456	0.049	5.27E-11
LRRC1	2.24E-15	0.392233	0.631	0.16	5.97E-11
PHB2	2.24E-15	0.297273	0.835	0.312	5.98E-11
ASH1L	2.27E-15	0.346764	0.835	0.292	6.04E-11
NEDD9	2.28E-15	1.067766	0.791	0.319	6.09E-11
NBDY	2.30E-15	0.327422	0.845	0.347	6.12E-11
LY6E	2.37E-15	0.71193	0.883	0.465	6.31E-11
ZNF655	2.39E-15	0.296537	0.665	0.181	6.36E-11
SFT2D1	2.39E-15	0.315578	0.786	0.264	6.36E-11

SUN1	2.41E-15	0.291364	0.762	0.222	6.43E-11
IRF8	2.43E-15	0.284927	0.515	0.083	6.48E-11
MCM3	2.44E-15	0.467006	0.534	0.09	6.50E-11
HDDC3	2.44E-15	0.33265	0.602	0.139	6.52E-11
AHR	2.46E-15	0.339324	0.709	0.208	6.56E-11
SLTM	2.47E-15	0.418595	0.879	0.354	6.60E-11
AKR1C3	2.50E-15	0.614453	0.908	0.417	6.66E-11
VBP1	2.61E-15	0.274123	0.694	0.201	6.95E-11
IHH	2.63E-15	0.278936	0.442	0.042	7.00E-11
INTS11	2.67E-15	0.285388	0.694	0.194	7.12E-11
TSC22D1	2.74E-15	0.716679	0.883	0.396	7.30E-11
FBXO21	2.76E-15	0.308191	0.592	0.132	7.36E-11
NAA30	2.84E-15	0.251929	0.451	0.049	7.56E-11
STAU1	2.87E-15	0.45152	0.908	0.368	7.64E-11
TMEM128	2.96E-15	0.25135	0.49	0.069	7.90E-11
TBC1D8B	2.99E-15	0.273781	0.5	0.076	7.98E-11
FXR1	3.04E-15	0.261713	0.757	0.236	8.10E-11
RBM14	3.07E-15	0.312213	0.563	0.111	8.19E-11
TRMT10B	3.10E-15	0.2668	0.481	0.062	8.25E-11
CHM	3.17E-15	0.28143	0.481	0.076	8.46E-11
MRPS18B	3.25E-15	0.301519	0.748	0.25	8.66E-11
TPM1	3.43E-15	0.73441	0.966	0.59	9.15E-11
KDM5B	3.56E-15	0.267691	0.757	0.236	9.48E-11
ZNF22	3.56E-15	0.288891	0.563	0.118	9.49E-11
MGMT	3.57E-15	0.283754	0.782	0.278	9.52E-11
ERLIN2	3.66E-15	0.32107	0.539	0.104	9.75E-11
ZNF462	3.67E-15	0.268732	0.408	0.028	9.78E-11
DNAJC2	3.76E-15	0.363127	0.665	0.201	1.00E-10
HDGF	3.80E-15	0.536262	0.908	0.424	1.01E-10
MORF4L2	4.03E-15	0.309472	0.845	0.368	1.07E-10
PIM3	4.12E-15	0.663029	0.869	0.389	1.10E-10
TP53I11	4.19E-15	0.310375	0.684	0.188	1.12E-10
SAMD4B	4.23E-15	0.318573	0.738	0.236	1.13E-10
GATA4	4.31E-15	0.295577	0.655	0.167	1.15E-10
IER5L	4.33E-15	0.598043	0.592	0.139	1.15E-10
LGALS2	4.47E-15	0.385904	0.578	0.118	1.19E-10
SP140L	4.60E-15	0.279497	0.578	0.118	1.23E-10
BRI3BP	4.69E-15	0.477579	0.675	0.194	1.25E-10
MRPS12	4.81E-15	0.297295	0.791	0.271	1.28E-10
MAFG	4.97E-15	0.373131	0.723	0.215	1.32E-10
KCNQ1OT1	4.99E-15	0.680293	0.733	0.236	1.33E-10
DDAH2	5.52E-15	0.329523	0.714	0.208	1.47E-10
TMEM176/	5.53E-15	0.361599	0.757	0.236	1.47E-10
DBNDD2	5.58E-15	0.391335	0.767	0.264	1.49E-10
PKP4	5.86E-15	0.346355	0.646	0.167	1.56E-10
GORASP2	5.89E-15	0.269882	0.762	0.264	1.57E-10
MAP3K13	5.91E-15	0.313797	0.825	0.278	1.58E-10
NASP	6.00E-15	0.337851	0.743	0.243	1.60E-10
RNF13	6.05E-15	0.250823	0.621	0.16	1.61E-10
GDI2	6.52E-15	0.559543	0.893	0.438	1.74E-10

HNF4A	6.71E-15	0.250957	0.65	0.181	1.79E-10
RNF114	6.78E-15	0.30456	0.786	0.264	1.81E-10
ITGB8	6.94E-15	0.473186	0.534	0.097	1.85E-10
LIMS1	6.96E-15	0.329776	0.714	0.215	1.85E-10
UBE4B	7.00E-15	0.293421	0.568	0.118	1.87E-10
RBM23	7.11E-15	0.287277	0.558	0.104	1.90E-10
PNPT1	7.23E-15	0.28843	0.515	0.09	1.93E-10
TM2D2	7.37E-15	0.327941	0.578	0.125	1.97E-10
CREBZF	7.42E-15	0.396673	0.646	0.181	1.98E-10
CISD1	7.84E-15	0.273558	0.65	0.167	2.09E-10
COBLL1	7.89E-15	0.43828	0.568	0.139	2.10E-10
ACAA2	7.91E-15	0.365002	0.825	0.312	2.11E-10
TRPM7	7.95E-15	0.263431	0.485	0.069	2.12E-10
ZNF721	8.09E-15	0.32707	0.539	0.104	2.16E-10
HOXB5	8.13E-15	0.285586	0.403	0.028	2.17E-10
SLMAP	8.64E-15	0.281945	0.675	0.181	2.30E-10
UBE2S	8.67E-15	0.467731	0.84	0.34	2.31E-10
CXCL16	8.71E-15	0.441841	0.879	0.333	2.32E-10
SMARCE1	9.28E-15	0.273974	0.777	0.229	2.47E-10
VKORC1L1	9.70E-15	0.256704	0.66	0.174	2.58E-10
FAM49B	9.80E-15	0.332768	0.743	0.25	2.61E-10
HSD17B10	1.03E-14	0.282884	0.738	0.25	2.73E-10
ANKRD13D	1.12E-14	0.340863	0.583	0.132	2.99E-10
FAM168B	1.13E-14	0.302073	0.568	0.118	3.01E-10
PIGP	1.14E-14	0.251471	0.714	0.222	3.03E-10
COG2	1.14E-14	0.283084	0.49	0.076	3.03E-10
EMC10	1.15E-14	0.327304	0.84	0.319	3.06E-10
TAF1D	1.15E-14	0.381681	0.859	0.347	3.07E-10
CELF1	1.17E-14	0.293538	0.762	0.257	3.11E-10
TMEM45B	1.18E-14	0.346879	0.738	0.229	3.14E-10
OXCT1	1.24E-14	0.266393	0.466	0.076	3.30E-10
AL121839.2	1.24E-14	0.304777	0.51	0.083	3.30E-10
AFTPH	1.24E-14	0.264442	0.67	0.194	3.31E-10
GNL3L	1.24E-14	0.27014	0.631	0.167	3.31E-10
TPM2	1.31E-14	0.505921	0.738	0.229	3.49E-10
CYHR1	1.32E-14	0.285256	0.718	0.215	3.52E-10
CUX1	1.34E-14	0.402716	0.796	0.299	3.57E-10
PSMD4	1.35E-14	0.341971	0.796	0.299	3.60E-10
TIAL1	1.39E-14	0.364814	0.723	0.236	3.70E-10
TBL1XR1	1.39E-14	0.458244	0.816	0.299	3.71E-10
ZPR1	1.40E-14	0.287903	0.646	0.188	3.72E-10
BCAP31	1.40E-14	0.419372	0.908	0.444	3.73E-10
IMPACT	1.40E-14	0.279973	0.573	0.125	3.74E-10
KLK1	1.41E-14	0.290058	0.612	0.139	3.76E-10
HNRNPUL2	1.41E-14	0.342548	0.743	0.257	3.77E-10
CTBP2	1.42E-14	0.359687	0.767	0.278	3.79E-10
U2AF2	1.45E-14	0.251458	0.748	0.222	3.86E-10
SMAD3	1.45E-14	0.281031	0.66	0.174	3.87E-10
MTF2	1.46E-14	0.289545	0.563	0.111	3.90E-10
SNRPD1	1.48E-14	0.419673	0.835	0.333	3.95E-10

FUS	1.51E-14	0.71371	0.951	0.493	4.01E-10
BSCL2	1.57E-14	0.316351	0.762	0.264	4.19E-10
POLR2I	1.64E-14	0.318362	0.869	0.368	4.37E-10
ARL5B	1.64E-14	0.352999	0.733	0.215	4.38E-10
UQCC3	1.72E-14	0.261189	0.718	0.222	4.59E-10
FIP1L1	1.76E-14	0.301706	0.607	0.153	4.68E-10
THEM6	1.78E-14	0.262233	0.563	0.125	4.75E-10
CORO1B	1.87E-14	0.40158	0.893	0.375	4.99E-10
KLHL23	1.92E-14	0.299039	0.471	0.062	5.10E-10
IDUA	1.98E-14	0.257748	0.553	0.118	5.27E-10
CDK12	2.05E-14	0.269445	0.723	0.215	5.46E-10
CCN2	2.13E-14	1.201134	0.481	0.083	5.66E-10
CHRAC1	2.23E-14	0.301395	0.563	0.111	5.95E-10
G3BP1	2.31E-14	0.321833	0.757	0.271	6.15E-10
MIEN1	2.36E-14	0.426992	0.835	0.333	6.30E-10
KRAS	2.38E-14	0.258478	0.85	0.319	6.34E-10
MTHFD2	2.41E-14	0.279576	0.68	0.208	6.41E-10
MRPS5	2.48E-14	0.273439	0.743	0.236	6.61E-10
IDI1	2.51E-14	0.499038	0.801	0.292	6.68E-10
SMAD5	2.60E-14	0.288022	0.631	0.153	6.94E-10
MAN1A2	2.61E-14	0.29189	0.811	0.312	6.97E-10
OAT	2.69E-14	0.568735	0.767	0.319	7.18E-10
PFDN4	2.73E-14	0.296134	0.718	0.222	7.28E-10
SHARPIN	2.87E-14	0.310165	0.684	0.208	7.64E-10
TIA1	2.94E-14	0.268545	0.568	0.139	7.83E-10
RAB21	3.07E-14	0.251944	0.709	0.215	8.19E-10
RUVBL1	3.13E-14	0.250016	0.481	0.076	8.33E-10
CALU	3.13E-14	0.382044	0.733	0.257	8.33E-10
GSTM4	3.13E-14	0.268718	0.558	0.118	8.35E-10
MUC5B	3.19E-14	0.707768	0.505	0.09	8.50E-10
IQGAP1	3.20E-14	0.435023	0.917	0.444	8.52E-10
TCOF1	3.27E-14	0.280556	0.427	0.049	8.72E-10
RBM26	3.47E-14	0.256317	0.621	0.16	9.24E-10
BOD1L1	3.54E-14	0.292614	0.859	0.34	9.44E-10
CENPS	3.58E-14	0.402527	0.49	0.09	9.55E-10
CHMP5	3.60E-14	0.421684	0.874	0.424	9.58E-10
BRIX1	3.65E-14	0.272039	0.524	0.104	9.72E-10
FAM204A	3.85E-14	0.272008	0.718	0.222	1.03E-09
LTBP3	3.87E-14	0.284312	0.539	0.111	1.03E-09
AIMP1	3.98E-14	0.412167	0.811	0.299	1.06E-09
PRDX3	4.09E-14	0.465992	0.82	0.34	1.09E-09
CHKA	4.12E-14	0.373269	0.65	0.188	1.10E-09
DLD	4.12E-14	0.294107	0.602	0.153	1.10E-09
MTMR11	4.13E-14	0.279921	0.573	0.125	1.10E-09
UBR5	4.17E-14	0.272458	0.621	0.153	1.11E-09
MTPN	4.19E-14	0.27604	0.806	0.271	1.12E-09
ZNF638	4.27E-14	0.400143	0.709	0.222	1.14E-09
VMP1	4.32E-14	0.66282	0.932	0.479	1.15E-09
PCK1	4.38E-14	1.362313	0.534	0.111	1.17E-09
XRRA1	4.49E-14	0.290124	0.471	0.069	1.20E-09

GSS	4.49E-14	0.286067	0.714	0.229	1.20E-09
BCLAF1	4.53E-14	0.407707	0.859	0.326	1.21E-09
SCYL2	4.55E-14	0.254323	0.524	0.104	1.21E-09
STAM2	4.66E-14	0.260265	0.442	0.062	1.24E-09
ADGRG1	4.67E-14	0.29372	0.641	0.194	1.24E-09
AC005261.	4.74E-14	0.260405	0.646	0.181	1.26E-09
EPN1	4.76E-14	0.423912	0.908	0.403	1.27E-09
EIF3B	4.88E-14	0.311443	0.743	0.25	1.30E-09
GRN	4.88E-14	0.491195	0.951	0.472	1.30E-09
NUDT5	5.34E-14	0.291497	0.617	0.153	1.42E-09
HSD3B7	5.50E-14	0.337642	0.451	0.062	1.47E-09
EIF3A	5.63E-14	0.537293	0.917	0.472	1.50E-09
SGK2	5.78E-14	0.283892	0.422	0.049	1.54E-09
CCN1	5.79E-14	0.513694	0.369	0.021	1.54E-09
PFDN2	6.05E-14	0.369739	0.864	0.326	1.61E-09
UCKL1	6.18E-14	0.28846	0.544	0.118	1.65E-09
SPINT2	6.45E-14	0.62566	0.951	0.625	1.72E-09
FKBP3	6.59E-14	0.312875	0.84	0.347	1.76E-09
RBM33	6.73E-14	0.263409	0.621	0.153	1.79E-09
CNOT2	6.87E-14	0.330293	0.796	0.285	1.83E-09
TCEAL8	6.93E-14	0.338712	0.718	0.25	1.85E-09
DNAJA3	6.95E-14	0.25426	0.49	0.083	1.85E-09
UTP23	7.07E-14	0.301501	0.549	0.125	1.89E-09
F5	7.42E-14	0.337777	0.519	0.104	1.98E-09
CCDC115	7.48E-14	0.290987	0.534	0.125	1.99E-09
EPS8	7.85E-14	0.400759	0.864	0.354	2.09E-09
MIS18BP1	8.08E-14	0.333762	0.592	0.146	2.15E-09
CETN2	8.08E-14	0.256874	0.592	0.167	2.15E-09
IFT57	8.35E-14	0.264269	0.529	0.111	2.22E-09
CTTN	8.66E-14	0.411373	0.932	0.472	2.31E-09
KLF10	8.71E-14	0.349328	0.66	0.194	2.32E-09
OLFM4	9.18E-14	1.442279	0.524	0.118	2.45E-09
AASDHPPT	9.26E-14	0.276778	0.505	0.104	2.47E-09
ANKS4B	9.42E-14	0.428837	0.432	0.056	2.51E-09
ANO1	9.42E-14	0.311723	0.607	0.16	2.51E-09
C16orf58	9.51E-14	0.279804	0.519	0.104	2.54E-09
MSI2	9.53E-14	0.369346	0.694	0.257	2.54E-09
C18orf32	9.91E-14	0.301931	0.883	0.354	2.64E-09
DDOST	1.00E-13	0.56153	0.835	0.41	2.68E-09
PNPLA8	1.01E-13	0.336734	0.699	0.243	2.69E-09
CLRN3	1.03E-13	0.256159	0.398	0.035	2.74E-09
NSMCE1	1.04E-13	0.277272	0.694	0.222	2.76E-09
AC060780.	1.07E-13	0.253857	0.442	0.062	2.85E-09
PPIG	1.09E-13	0.293878	0.883	0.389	2.90E-09
PHF20L1	1.09E-13	0.32904	0.714	0.243	2.91E-09
MRPL21	1.10E-13	0.252025	0.859	0.333	2.92E-09
NUCKS1	1.11E-13	0.38702	0.913	0.41	2.96E-09
FXYD5	1.11E-13	0.639491	0.845	0.396	2.97E-09
MAP4K3	1.14E-13	0.292505	0.563	0.139	3.04E-09
CD46	1.14E-13	0.292702	0.883	0.41	3.04E-09

EPRS	1.14E-13	0.331317	0.782	0.278	3.05E-09
TIMM50	1.19E-13	0.301162	0.626	0.181	3.18E-09
UBC	1.21E-13	0.598043	0.995	0.91	3.22E-09
PAIP1	1.24E-13	0.261815	0.617	0.16	3.31E-09
KRIT1	1.28E-13	0.278496	0.563	0.132	3.40E-09
NDUFS8	1.32E-13	0.541597	0.932	0.514	3.52E-09
PPP1R15A	1.34E-13	0.861632	0.927	0.576	3.58E-09
HIST3H2A	1.35E-13	0.331325	0.553	0.125	3.60E-09
NOP58	1.39E-13	0.295789	0.704	0.222	3.71E-09
CITED4	1.43E-13	0.251291	0.544	0.132	3.80E-09
RPP21	1.45E-13	0.252219	0.806	0.306	3.86E-09
AP2B1	1.46E-13	0.272323	0.699	0.222	3.88E-09
COMMD7	1.54E-13	0.314908	0.752	0.271	4.11E-09
ATP5ME	1.55E-13	0.587309	0.966	0.757	4.13E-09
IER3IP1	1.64E-13	0.344434	0.835	0.34	4.36E-09
MT-ND6	1.65E-13	0.504482	0.646	0.194	4.39E-09
SRF	1.66E-13	0.254994	0.587	0.146	4.44E-09
SLC39A14	1.68E-13	0.285058	0.752	0.264	4.49E-09
FOXQ1	1.69E-13	0.54105	0.83	0.34	4.51E-09
PLEKHB2	1.71E-13	0.272276	0.811	0.312	4.56E-09
STT3A	1.73E-13	0.252059	0.544	0.139	4.61E-09
NFE2L3	1.76E-13	0.25511	0.466	0.076	4.69E-09
PSMD12	1.83E-13	0.262838	0.621	0.167	4.89E-09
ANAPC5	1.86E-13	0.318543	0.748	0.264	4.96E-09
SLAIN2	1.92E-13	0.255845	0.578	0.139	5.12E-09
TUFT1	1.93E-13	0.260811	0.573	0.132	5.15E-09
CHD2	1.96E-13	0.392126	0.879	0.382	5.22E-09
GFPT1	2.01E-13	0.351505	0.888	0.375	5.36E-09
ABI1	2.11E-13	0.321904	0.762	0.264	5.62E-09
DERA	2.17E-13	0.259776	0.539	0.132	5.78E-09
ARF6	2.24E-13	0.534361	0.937	0.486	5.97E-09
PHB	2.25E-13	0.472584	0.883	0.396	5.99E-09
TACC1	2.27E-13	0.425326	0.549	0.125	6.04E-09
STEAP2	2.33E-13	0.332418	0.442	0.069	6.22E-09
DEPTOR	2.35E-13	0.268257	0.466	0.083	6.26E-09
SSU72	2.36E-13	0.290248	0.893	0.438	6.30E-09
TGOLN2	2.37E-13	0.455673	0.922	0.444	6.31E-09
HNRNPU	2.48E-13	0.593735	0.937	0.5	6.61E-09
KLF7	2.54E-13	0.301154	0.5	0.104	6.76E-09
SUMO1	2.60E-13	0.373409	0.874	0.382	6.94E-09
ZC3H12A	2.73E-13	0.509805	0.718	0.271	7.28E-09
RBCK1	2.85E-13	0.332816	0.835	0.368	7.59E-09
PAICS	3.15E-13	0.262267	0.646	0.188	8.39E-09
KRCC1	3.39E-13	0.255371	0.563	0.132	9.02E-09
COMMD4	3.47E-13	0.259553	0.704	0.215	9.24E-09
GRB7	3.54E-13	0.314089	0.476	0.09	9.43E-09
B3GNT7	3.55E-13	0.459616	0.592	0.167	9.46E-09
LSM14A	3.58E-13	0.256977	0.704	0.222	9.53E-09
ZNF326	3.60E-13	0.267039	0.621	0.174	9.58E-09
TSPAN3	3.63E-13	0.497651	0.966	0.639	9.67E-09

CPM	3.88E-13	0.284172	0.442	0.069	1.03E-08
TPPP3	3.90E-13	0.43008	0.364	0.028	1.04E-08
LPP	4.12E-13	0.312094	0.845	0.333	1.10E-08
TFRC	4.13E-13	0.338585	0.68	0.236	1.10E-08
MAVS	4.19E-13	0.326409	0.607	0.167	1.12E-08
MAP3K2	4.40E-13	0.328851	0.777	0.306	1.17E-08
SERINC1	4.41E-13	0.295952	0.782	0.292	1.17E-08
ITGA6	4.44E-13	0.328862	0.854	0.368	1.18E-08
FAT1	4.55E-13	0.252746	0.714	0.236	1.21E-08
HIKESHI	4.64E-13	0.255809	0.5	0.104	1.24E-08
BNIP2	4.68E-13	0.388262	0.515	0.118	1.25E-08
SFSWAP	4.80E-13	0.275785	0.612	0.174	1.28E-08
GNL3	4.80E-13	0.307133	0.65	0.194	1.28E-08
CHD4	4.87E-13	0.374617	0.757	0.278	1.30E-08
PNN	5.19E-13	0.43864	0.874	0.396	1.38E-08
RAB2A	5.19E-13	0.414983	0.917	0.465	1.38E-08
LGALS3	5.24E-13	0.658788	0.976	0.806	1.40E-08
VIL1	5.24E-13	0.255297	0.641	0.194	1.40E-08
IFNGR1	5.30E-13	0.335511	0.646	0.201	1.41E-08
RSL1D1	5.31E-13	0.339707	0.83	0.368	1.41E-08
TUT4	5.50E-13	0.259266	0.476	0.09	1.47E-08
RAB11FIP2	5.60E-13	0.260294	0.485	0.097	1.49E-08
HEXA	5.61E-13	0.318594	0.835	0.34	1.49E-08
POLR2H	5.69E-13	0.313409	0.704	0.229	1.52E-08
MYC	5.71E-13	0.254579	0.519	0.118	1.52E-08
TMEM123	5.73E-13	0.433762	0.825	0.333	1.53E-08
KIAA1522	6.08E-13	0.345378	0.762	0.312	1.62E-08
NEAT1	6.14E-13	0.912036	0.976	0.632	1.64E-08
TXNRD1	6.17E-13	0.36423	0.728	0.25	1.64E-08
ICK	6.35E-13	0.34966	0.534	0.132	1.69E-08
IFRD1	6.41E-13	0.348049	0.733	0.264	1.71E-08
LPIN2	6.64E-13	0.361057	0.908	0.424	1.77E-08
BAG6	6.97E-13	0.31484	0.67	0.215	1.86E-08
TFAM	7.20E-13	0.254768	0.524	0.125	1.92E-08
TCP1	7.23E-13	0.311113	0.82	0.333	1.93E-08
ADAR	7.26E-13	0.251448	0.835	0.347	1.93E-08
HSPA1B	7.63E-13	0.908941	0.942	0.514	2.03E-08
RND1	7.86E-13	0.326568	0.549	0.132	2.09E-08
MTX1	7.89E-13	0.252935	0.646	0.208	2.10E-08
PRMT1	8.88E-13	0.404269	0.83	0.368	2.37E-08
LRPAP1	9.09E-13	0.33119	0.883	0.424	2.42E-08
HSD17B8	9.14E-13	0.257392	0.451	0.083	2.44E-08
AZIN1	9.85E-13	0.25836	0.752	0.264	2.63E-08
PRRC2C	1.02E-12	0.340797	0.922	0.5	2.72E-08
UACA	1.03E-12	0.257421	0.597	0.167	2.75E-08
RIN2	1.05E-12	0.286556	0.408	0.056	2.80E-08
HSPA1A	1.06E-12	1.209479	0.942	0.583	2.83E-08
MTCH1	1.08E-12	0.275238	0.835	0.354	2.87E-08
MFN2	1.13E-12	0.489503	0.612	0.201	3.00E-08
RBMX	1.16E-12	0.318201	0.84	0.326	3.09E-08

LARP1	1.18E-12	0.322172	0.796	0.299	3.13E-08
EFNA1	1.18E-12	0.411697	0.811	0.333	3.16E-08
RPA3	1.19E-12	0.316986	0.718	0.257	3.17E-08
DNAJC1	1.21E-12	0.253544	0.82	0.34	3.23E-08
GSPT1	1.21E-12	0.365413	0.859	0.361	3.23E-08
MRPL35	1.22E-12	0.265487	0.544	0.132	3.24E-08
NDUFAB1	1.23E-12	0.551103	0.869	0.451	3.27E-08
TM4SF1	1.24E-12	0.749834	0.845	0.382	3.30E-08
AHSA1	1.26E-12	0.28523	0.631	0.201	3.35E-08
CYTH2	1.26E-12	0.274415	0.641	0.188	3.35E-08
TRIB1	1.26E-12	0.49744	0.718	0.278	3.37E-08
EPHB2	1.30E-12	0.285332	0.35	0.028	3.46E-08
POLR2M	1.32E-12	0.255669	0.549	0.132	3.52E-08
PPIB	1.37E-12	0.58036	0.947	0.667	3.66E-08
SERTAD2	1.38E-12	0.360603	0.587	0.16	3.69E-08
BCL10	1.41E-12	0.297877	0.723	0.236	3.76E-08
CYC1	1.41E-12	0.387091	0.874	0.389	3.77E-08
C6orf89	1.43E-12	0.256977	0.65	0.188	3.81E-08
THRAP3	1.45E-12	0.321082	0.83	0.306	3.86E-08
ID1	1.45E-12	1.048959	0.796	0.354	3.86E-08
WAPL	1.47E-12	0.257163	0.519	0.132	3.91E-08
SLK	1.51E-12	0.317448	0.694	0.229	4.02E-08
VEGFA	1.51E-12	0.599216	0.845	0.347	4.02E-08
SPTBN1	1.58E-12	0.429335	0.898	0.472	4.22E-08
TSFM	1.63E-12	0.255856	0.51	0.111	4.35E-08
BNIP5	1.69E-12	0.953906	0.592	0.181	4.51E-08
VEGFB	1.79E-12	0.342422	0.689	0.243	4.78E-08
ZFAND2A	1.82E-12	0.534879	0.66	0.222	4.84E-08
PSMA5	1.82E-12	0.387422	0.835	0.326	4.85E-08
MRPL20	1.85E-12	0.368136	0.883	0.41	4.94E-08
LUC7L	1.90E-12	0.293775	0.592	0.174	5.07E-08
HIC2	1.92E-12	0.256855	0.369	0.042	5.12E-08
RRN3	1.98E-12	0.259364	0.403	0.056	5.27E-08
HSP90B1	2.01E-12	0.590687	0.947	0.597	5.37E-08
WTAP	2.05E-12	0.283074	0.743	0.25	5.46E-08
XRCC5	2.07E-12	0.373135	0.888	0.403	5.51E-08
UBR3	2.13E-12	0.250995	0.422	0.069	5.68E-08
GPN3	2.19E-12	0.250272	0.456	0.09	5.85E-08
IGKC	2.20E-12	-0.84061	1	0.986	5.87E-08
ECHS1	2.21E-12	0.286738	0.879	0.431	5.89E-08
RBPMS	2.22E-12	0.303687	0.485	0.104	5.91E-08
TIPARP	2.27E-12	0.487741	0.723	0.285	6.05E-08
ANXA2	2.44E-12	0.487918	0.981	0.743	6.50E-08
NDFIP2	2.44E-12	0.27766	0.65	0.215	6.50E-08
PGK1	2.55E-12	0.364797	0.888	0.479	6.79E-08
CDKN2AIP	2.58E-12	0.361778	0.684	0.257	6.87E-08
F11R	2.70E-12	0.333831	0.903	0.431	7.19E-08
CFLAR	2.80E-12	0.374817	0.825	0.354	7.47E-08
GLRX5	2.83E-12	0.325091	0.874	0.444	7.53E-08
RSBN1L	2.92E-12	0.280483	0.748	0.278	7.79E-08

COA4	2.94E-12	0.334213	0.869	0.396	7.84E-08
UBE2Q1	3.01E-12	0.299471	0.621	0.194	8.03E-08
ABHD16A	3.11E-12	0.256809	0.568	0.146	8.29E-08
ST3GAL1	3.19E-12	0.396123	0.383	0.056	8.51E-08
PPP1CA	3.25E-12	0.480774	0.922	0.556	8.65E-08
GLRX2	3.27E-12	0.265044	0.558	0.153	8.72E-08
CCT7	3.28E-12	0.254444	0.801	0.312	8.74E-08
IDS	3.42E-12	0.422012	0.752	0.278	9.12E-08
AK6	3.46E-12	0.258448	0.515	0.118	9.23E-08
TCEA1	3.51E-12	0.361273	0.82	0.354	9.35E-08
MSMB	3.62E-12	-3.11799	0.058	0.326	9.65E-08
SLPI	3.74E-12	0.630874	0.903	0.472	9.98E-08
TMEM41A	3.91E-12	0.297223	0.539	0.146	1.04E-07
SEL1L3	4.04E-12	0.280214	0.752	0.312	1.08E-07
PTGES2	4.16E-12	0.296897	0.636	0.181	1.11E-07
PSMD7	4.33E-12	0.38752	0.811	0.326	1.15E-07
PRR26	4.50E-12	0.323329	0.427	0.076	1.20E-07
SRRM2	4.63E-12	0.279286	0.961	0.493	1.23E-07
SNF8	4.76E-12	0.250069	0.84	0.354	1.27E-07
KRT17	5.05E-12	1.373502	0.408	0.069	1.35E-07
BRAF	5.12E-12	0.32282	0.451	0.09	1.36E-07
GLYCTK	5.34E-12	0.252118	0.442	0.076	1.42E-07
DYNLRB1	5.67E-12	0.355778	0.922	0.556	1.51E-07
PLPP2	5.85E-12	0.352392	0.801	0.299	1.56E-07
BACH1	6.34E-12	0.295617	0.597	0.188	1.69E-07
LLGL2	6.40E-12	0.340814	0.947	0.424	1.71E-07
KMT2A	6.45E-12	0.266867	0.767	0.299	1.72E-07
AKAP13	6.64E-12	0.523649	0.922	0.479	1.77E-07
VRK1	6.66E-12	0.294016	0.442	0.083	1.78E-07
NAP1L1	6.83E-12	0.289096	0.835	0.403	1.82E-07
MAD2L2	6.90E-12	0.837099	0.51	0.139	1.84E-07
IDH1	7.25E-12	0.363979	0.83	0.319	1.93E-07
PDAP1	7.73E-12	0.266318	0.835	0.354	2.06E-07
SUPT4H1	7.89E-12	0.271078	0.684	0.229	2.10E-07
IGFBP2	7.90E-12	0.688496	0.806	0.361	2.10E-07
CLDN7	7.92E-12	0.434038	0.942	0.472	2.11E-07
ENY2	7.93E-12	0.309012	0.908	0.41	2.11E-07
ZNF511	8.11E-12	0.256865	0.646	0.201	2.16E-07
RSRC2	9.21E-12	0.307595	0.879	0.389	2.45E-07
RND3	9.32E-12	0.342708	0.801	0.306	2.48E-07
ARL14	9.88E-12	0.359354	0.738	0.319	2.63E-07
NR1D1	9.91E-12	0.481784	0.451	0.097	2.64E-07
WHAMM	9.93E-12	0.258931	0.495	0.111	2.65E-07
C1orf43	1.00E-11	0.303973	0.83	0.361	2.67E-07
HIF1A	1.02E-11	0.266201	0.607	0.181	2.72E-07
TGM2	1.03E-11	0.49613	0.388	0.056	2.74E-07
LINC01578	1.04E-11	0.600838	0.932	0.479	2.77E-07
MTHFD1	1.09E-11	0.322776	0.563	0.153	2.90E-07
SPON2	1.14E-11	0.863801	0.369	0.049	3.04E-07
KHDRBS1	1.15E-11	0.348439	0.879	0.444	3.06E-07

KCNE3	1.18E-11	0.383885	0.733	0.278	3.13E-07
KHDC4	1.20E-11	0.288919	0.597	0.188	3.20E-07
ZMYM2	1.24E-11	0.255526	0.607	0.188	3.30E-07
UBE2M	1.26E-11	0.261197	0.869	0.417	3.36E-07
UQCRFS1	1.32E-11	0.374189	0.874	0.438	3.51E-07
MTOR	1.35E-11	0.279273	0.485	0.118	3.60E-07
GGNBP2	1.40E-11	0.256413	0.791	0.299	3.72E-07
C12orf75	1.43E-11	0.299013	0.893	0.486	3.81E-07
RRBP1	1.47E-11	0.636933	0.971	0.576	3.91E-07
TUFM	1.48E-11	0.378684	0.869	0.465	3.96E-07
SELENOF	1.52E-11	0.381728	0.883	0.431	4.06E-07
PIM1	1.54E-11	0.302495	0.607	0.188	4.10E-07
PPL	1.55E-11	0.266499	0.422	0.083	4.12E-07
CCT5	1.56E-11	0.376132	0.816	0.354	4.16E-07
RHOU	1.62E-11	0.27852	0.534	0.146	4.32E-07
TLK1	1.70E-11	0.28675	0.617	0.201	4.53E-07
RAD23A	1.79E-11	0.286489	0.845	0.382	4.77E-07
ALG3	1.80E-11	0.276551	0.573	0.181	4.79E-07
CXCL5	1.86E-11	1.01689	0.456	0.104	4.96E-07
SEC62	1.89E-11	0.355733	0.908	0.458	5.03E-07
ZFAND5	2.05E-11	0.385919	0.806	0.333	5.46E-07
MRPS34	2.14E-11	0.384581	0.83	0.382	5.71E-07
BAG3	2.15E-11	0.616538	0.602	0.208	5.74E-07
MDM4	2.27E-11	0.290976	0.665	0.236	6.06E-07
HSPA6	2.34E-11	1.661608	0.427	0.097	6.25E-07
HNRNPUL1	2.38E-11	0.279871	0.835	0.354	6.34E-07
CDH17	2.45E-11	0.266465	0.617	0.194	6.54E-07
RFK	2.47E-11	0.313709	0.636	0.201	6.57E-07
TOMM20	2.47E-11	0.476002	0.893	0.444	6.58E-07
TFF1	2.48E-11	-2.57971	0.743	0.799	6.62E-07
GLG1	2.48E-11	0.338216	0.675	0.25	6.62E-07
SFTA2	2.51E-11	0.32377	0.563	0.167	6.69E-07
PTMA	2.51E-11	0.571462	0.981	0.896	6.70E-07
CALML4	2.58E-11	0.288774	0.786	0.354	6.89E-07
AC009133.	2.78E-11	0.270449	0.471	0.111	7.42E-07
MAGT1	2.81E-11	0.258926	0.767	0.319	7.48E-07
MDH1	3.15E-11	0.286121	0.883	0.41	8.39E-07
EIF3M	3.16E-11	0.348766	0.83	0.403	8.42E-07
FARP1	3.28E-11	0.252122	0.583	0.174	8.73E-07
CNPY2	3.33E-11	0.265997	0.816	0.361	8.88E-07
TLN2	3.76E-11	0.289941	0.442	0.09	1.00E-06
FAM133B	3.92E-11	0.253314	0.83	0.354	1.04E-06
CD9	4.10E-11	0.496591	0.879	0.451	1.09E-06
CCNL1	4.10E-11	0.54616	0.927	0.493	1.09E-06
SNAP23	4.15E-11	0.252315	0.68	0.236	1.11E-06
NAMPT	4.16E-11	0.432231	0.845	0.368	1.11E-06
PSMA7	4.28E-11	0.545265	0.927	0.597	1.14E-06
ALDH1A1	5.02E-11	0.682431	0.879	0.521	1.34E-06
PDXK	5.20E-11	0.323757	0.83	0.375	1.38E-06
SLC12A2	5.25E-11	0.391716	0.718	0.292	1.40E-06

MORF4L1	5.27E-11	0.395031	0.922	0.569	1.40E-06
SDF2L1	5.63E-11	0.341454	0.772	0.326	1.50E-06
ZFP36L1	5.64E-11	0.605079	0.893	0.507	1.50E-06
NDUFA12	5.68E-11	0.25347	0.864	0.382	1.51E-06
BRD2	6.22E-11	0.317921	0.874	0.41	1.66E-06
PRSS33	6.27E-11	0.298884	0.286	0.014	1.67E-06
AKAP9	6.82E-11	0.282872	0.917	0.507	1.82E-06
SOX4	6.92E-11	0.585423	0.82	0.361	1.84E-06
SLC2A4RG	7.22E-11	0.283955	0.607	0.201	1.92E-06
GATM	7.25E-11	0.374664	0.553	0.16	1.93E-06
SNRPB	7.53E-11	0.265465	0.874	0.444	2.01E-06
PTMS	7.55E-11	0.290544	0.85	0.382	2.01E-06
ITPA	7.89E-11	0.314284	0.66	0.243	2.10E-06
SRSF4	8.11E-11	0.255257	0.811	0.34	2.16E-06
TAOK1	8.18E-11	0.299952	0.602	0.201	2.18E-06
EIF4A2	8.30E-11	0.545933	0.942	0.521	2.21E-06
TRAF4	8.50E-11	0.401469	0.82	0.368	2.27E-06
HILPDA	8.91E-11	0.292826	0.34	0.042	2.37E-06
RPS21	9.27E-11	0.578361	0.976	0.792	2.47E-06
KMT2E	9.50E-11	0.341068	0.864	0.417	2.53E-06
LAD1	9.57E-11	0.320014	0.733	0.285	2.55E-06
FGFR1OP2	1.09E-10	0.309551	0.524	0.153	2.89E-06
RPL23	1.09E-10	0.631995	0.951	0.715	2.90E-06
NPC1	1.10E-10	0.28262	0.447	0.104	2.93E-06
TTTY14	1.16E-10	0.297134	0.524	0.153	3.09E-06
MT-ATP6	1.19E-10	0.634596	0.995	0.875	3.18E-06
ST13	1.20E-10	0.315077	0.869	0.458	3.20E-06
BANF1	1.28E-10	0.385244	0.869	0.451	3.42E-06
CCL20	1.33E-10	1.335902	0.354	0.056	3.54E-06
SARAF	1.34E-10	0.33312	0.922	0.507	3.57E-06
GOLGB1	1.41E-10	0.430002	0.922	0.479	3.76E-06
SUCLG1	1.44E-10	0.283845	0.806	0.354	3.83E-06
GDA	1.56E-10	0.295039	0.398	0.069	4.17E-06
TMED3	1.61E-10	0.288723	0.869	0.417	4.30E-06
TNRC6B	1.64E-10	0.256245	0.752	0.306	4.38E-06
AREG	1.68E-10	0.920147	0.752	0.375	4.48E-06
EIF3I	1.81E-10	0.278127	0.835	0.396	4.83E-06
KDELR1	1.87E-10	0.450155	0.917	0.479	4.98E-06
LGMN	1.88E-10	0.283045	0.82	0.347	5.02E-06
MYLIP	1.90E-10	0.2868	0.427	0.097	5.06E-06
TRIM31	1.98E-10	0.308517	0.786	0.319	5.28E-06
RBBP4	2.15E-10	0.259885	0.757	0.299	5.74E-06
CLINT1	2.22E-10	0.257847	0.791	0.354	5.92E-06
HSPA5	2.28E-10	0.556592	0.888	0.507	6.09E-06
SF3B2	2.31E-10	0.321523	0.85	0.403	6.16E-06
DYNC1H1	2.43E-10	0.256532	0.82	0.326	6.46E-06
HBEGF	2.65E-10	0.447745	0.592	0.215	7.07E-06
ANXA4	2.66E-10	0.457943	0.932	0.569	7.08E-06
CEBPD	2.80E-10	0.527654	0.864	0.41	7.46E-06
PSMA4	2.84E-10	0.258051	0.859	0.41	7.56E-06

AKR1C1	3.00E-10	0.55967	0.684	0.278	8.00E-06
SKIL	3.18E-10	0.254231	0.684	0.271	8.47E-06
ERBB2	3.46E-10	0.255786	0.675	0.257	9.23E-06
RPLP1	3.62E-10	0.515707	0.995	0.924	9.64E-06
RTRAF	3.97E-10	0.382744	0.927	0.528	1.06E-05
EWSR1	4.12E-10	0.263729	0.801	0.34	1.10E-05
SYPL1	4.28E-10	0.30978	0.825	0.368	1.14E-05
C1QBP	4.32E-10	0.347777	0.782	0.354	1.15E-05
CHMP2A	4.42E-10	0.254301	0.951	0.521	1.18E-05
MRPL24	4.43E-10	0.256594	0.607	0.215	1.18E-05
STAT3	4.79E-10	0.373742	0.893	0.444	1.28E-05
TMEM238	4.83E-10	0.288569	0.917	0.514	1.29E-05
KLF6	4.87E-10	0.637369	0.981	0.646	1.30E-05
ACADM	4.96E-10	0.250294	0.553	0.188	1.32E-05
PSMD11	5.19E-10	0.293437	0.757	0.333	1.38E-05
PTPMT1	5.61E-10	0.253416	0.597	0.194	1.49E-05
RYBP	5.62E-10	0.291624	0.607	0.222	1.50E-05
ZMAT1	5.66E-10	0.291853	0.277	0.021	1.51E-05
GCNT1	5.68E-10	0.33031	0.646	0.243	1.51E-05
AQP1	5.85E-10	0.277021	0.34	0.049	1.56E-05
FBXO6	6.09E-10	0.417241	0.379	0.076	1.62E-05
ARL6IP1	6.12E-10	0.640886	0.942	0.646	1.63E-05
GPRC5A	6.54E-10	0.476405	0.83	0.396	1.74E-05
NFKB2	6.70E-10	0.253916	0.549	0.174	1.79E-05
OAS1	7.24E-10	0.254377	0.655	0.229	1.93E-05
SFRP5	7.45E-10	0.349378	0.291	0.028	1.98E-05
PSMB3	7.69E-10	0.347686	0.908	0.521	2.05E-05
GABRP	8.59E-10	0.256256	0.398	0.083	2.29E-05
MTHFR	8.66E-10	0.452464	0.607	0.236	2.31E-05
NDUFS5	9.19E-10	0.322784	0.927	0.569	2.45E-05
ALDH2	9.98E-10	0.398369	0.913	0.583	2.66E-05
SREK1IP1	1.07E-09	0.255447	0.568	0.188	2.84E-05
ENO1	1.07E-09	0.769783	0.913	0.611	2.86E-05
VSIG10	1.14E-09	0.301498	0.471	0.132	3.04E-05
LSR	1.18E-09	0.386584	0.913	0.479	3.14E-05
GDF15	1.27E-09	0.60782	0.631	0.243	3.40E-05
TIMP1	1.30E-09	0.294782	0.898	0.479	3.46E-05
IGHA1	1.30E-09	-1.52863	0.99	0.931	3.46E-05
HNRNPD	1.37E-09	0.250755	0.888	0.472	3.64E-05
SRSF3	1.47E-09	0.271776	0.917	0.493	3.92E-05
SLC12A7	1.66E-09	0.293293	0.51	0.153	4.43E-05
RAB10	1.75E-09	0.256236	0.791	0.347	4.66E-05
DNAJB4	1.86E-09	0.381113	0.432	0.118	4.95E-05
DANCR	1.89E-09	0.250015	0.859	0.493	5.05E-05
MT-ND4L	1.93E-09	0.425567	0.956	0.632	5.13E-05
MET	1.94E-09	0.285546	0.456	0.125	5.18E-05
TNFSF15	2.14E-09	0.280808	0.291	0.035	5.71E-05
FBXO2	2.25E-09	0.839487	0.437	0.118	6.00E-05
SOD2	2.26E-09	0.293352	0.786	0.361	6.03E-05
LGALS4	2.29E-09	0.322564	0.976	0.757	6.11E-05

TMEM50A	2.36E-09	0.251107	0.85	0.417	6.28E-05
EPHA2	3.11E-09	0.384975	0.607	0.243	8.29E-05
BRI3	3.69E-09	0.38733	0.942	0.542	9.83E-05
CD47	3.85E-09	0.258263	0.723	0.326	0.000103
TNFRSF12A	4.09E-09	0.370642	0.519	0.167	0.000109
TSPAN8	4.15E-09	0.650675	0.971	0.75	0.000111
RPS17	4.37E-09	0.329441	0.956	0.722	0.000116
RCAN1	4.42E-09	0.296744	0.539	0.181	0.000118
KLK8	4.67E-09	0.357079	0.262	0.021	0.000124
RAN	4.95E-09	0.331541	0.913	0.569	0.000132
B2M	5.23E-09	-0.70762	1	1	0.000139
RPS16	5.40E-09	0.443059	0.971	0.861	0.000144
EIF1AX	5.77E-09	0.260384	0.84	0.417	0.000154
HNF1A-AS1	6.28E-09	0.261655	0.354	0.076	0.000167
NFKBIZ	6.40E-09	0.357634	0.641	0.271	0.000171
MANF	6.41E-09	0.278905	0.699	0.299	0.000171
HES1	6.55E-09	0.507278	0.752	0.333	0.000175
FBXO44	8.89E-09	0.293593	0.335	0.069	0.000237
NFKBIA	1.03E-08	0.619242	0.883	0.479	0.000273
GAS5	1.04E-08	0.340984	0.83	0.41	0.000276
MAL2	1.06E-08	0.311992	0.932	0.514	0.000281
POF1B	1.20E-08	-0.26971	0.597	0.215	0.000319
SELENOP	1.21E-08	-0.55111	0.898	0.438	0.000323
CD164	1.26E-08	0.379792	0.927	0.556	0.000335
GTF2I	1.35E-08	0.270068	0.908	0.451	0.000359
PDIA3	1.39E-08	0.277739	0.947	0.597	0.000371
MUC5AC	1.40E-08	-2.63736	0.519	0.625	0.000372
MARCKSL1	1.44E-08	0.48705	0.903	0.535	0.000383
SPCS2	1.44E-08	0.270344	0.913	0.486	0.000383
PRSS1	1.44E-08	0.518988	0.311	0.056	0.000384
MAFF	1.48E-08	0.299889	0.752	0.34	0.000394
TENT5A	1.51E-08	0.306574	0.82	0.389	0.000401
ANKRD17	1.54E-08	-0.27297	0.655	0.229	0.00041
CDC42	1.55E-08	0.313278	0.932	0.604	0.000414
ERRFI1	1.58E-08	0.260298	0.587	0.222	0.000422
PPP1R14B	1.64E-08	0.27615	0.83	0.396	0.000437
PRSS8	1.66E-08	0.406835	0.825	0.431	0.000443
S100A4	1.72E-08	-0.52562	0.704	0.264	0.000458
HNRNPK	1.79E-08	0.288855	0.898	0.528	0.000476
AQP3	1.79E-08	0.571239	0.451	0.153	0.000476
DNAJB1	1.80E-08	0.729102	0.874	0.542	0.00048
ERP29	1.90E-08	0.320461	0.927	0.528	0.000507
DSTN	1.91E-08	0.340218	0.917	0.639	0.00051
ZBTB20	2.00E-08	0.34739	0.573	0.222	0.000534
H3F3A	2.14E-08	0.377022	0.985	0.833	0.00057
ZFAND2B	2.17E-08	-0.27048	0.553	0.201	0.000579
RPS20	2.23E-08	0.417119	0.947	0.653	0.000595
HNRNPC	2.30E-08	0.260999	0.922	0.514	0.000612
MZT2B	2.41E-08	0.298088	0.937	0.569	0.000643
C2CD4B	2.59E-08	0.346386	0.354	0.083	0.000691

TRMT112	2.61E-08	0.283531	0.893	0.5	0.000696
YWHAE	2.87E-08	0.264545	0.927	0.493	0.000766
PDZK1IP1	3.01E-08	0.378627	0.675	0.299	0.000802
CA9	3.08E-08	0.49549	0.432	0.139	0.000822
SLC25A5	3.17E-08	0.363492	0.956	0.729	0.000846
NCOA7	3.30E-08	0.373344	0.709	0.354	0.00088
JUN	3.68E-08	0.643665	0.976	0.743	0.00098
NME1	3.76E-08	0.334824	0.665	0.312	0.001003
SNRPF	3.77E-08	0.261299	0.864	0.451	0.001006
APOA1	3.82E-08	-1.01178	0.481	0.146	0.001018
RPL36AL	3.83E-08	0.370483	0.981	0.792	0.00102
CPS1	3.84E-08	0.342472	0.223	0.014	0.001023
MGST3	4.02E-08	0.380124	0.922	0.597	0.001072
NPDC1	4.45E-08	-0.79704	0.107	0.326	0.001186
RAB11FIP1	4.95E-08	0.370786	0.937	0.556	0.001318
NR4A1	4.97E-08	0.355517	0.83	0.465	0.001324
CD55	6.07E-08	0.289131	0.835	0.5	0.001618
ELF3	6.12E-08	0.535561	0.985	0.66	0.001631
SMARCA4	6.21E-08	-0.29669	0.665	0.25	0.001655
MAP3K20-/	6.59E-08	0.271894	0.199	0.007	0.001755
CFB	6.61E-08	0.302925	0.738	0.354	0.001761
IGLC2	6.68E-08	-0.57479	0.976	0.882	0.00178
SQLE	7.73E-08	0.303284	0.597	0.25	0.00206
AKR1C2	1.00E-07	0.410365	0.544	0.215	0.002671
RNF186	1.00E-07	0.258326	0.194	0.007	0.002677
SPINK1	1.18E-07	0.740211	0.932	0.743	0.003155
DDT	1.19E-07	0.319394	0.883	0.507	0.003175
SRM	1.36E-07	0.470016	0.607	0.285	0.003621
ACTN4	1.40E-07	0.399617	0.947	0.583	0.003731
STARD10	1.46E-07	0.321258	0.966	0.632	0.003879
RPS26	1.49E-07	-0.95871	0.99	0.847	0.003984
MDH2	1.62E-07	0.288202	0.869	0.486	0.004305
HMGB2	1.67E-07	0.265713	0.534	0.201	0.004445
CNPPD1	1.77E-07	-0.39594	0.602	0.236	0.004722
SDC4	1.79E-07	0.29285	0.874	0.479	0.004771
PLAUR	2.44E-07	0.389581	0.791	0.417	0.006496
PTRH1	2.64E-07	-0.37651	0.456	0.139	0.007049
DBI	2.78E-07	0.293619	0.966	0.66	0.007408
H1F0	3.22E-07	0.342094	0.592	0.299	0.008582
CIB1	3.55E-07	0.256795	0.947	0.597	0.009466
GSTP1	3.93E-07	0.321785	0.99	0.903	0.010479
RPL4	4.17E-07	0.351222	0.961	0.722	0.011127
CXCL2	4.35E-07	0.726788	0.505	0.208	0.011603
SPTY2D1	4.43E-07	-0.27067	0.568	0.215	0.011794
PDPK1	4.44E-07	-0.2766	0.592	0.236	0.011834
C15orf48	4.82E-07	0.604754	0.782	0.472	0.012855
CXCL1	5.69E-07	1.028853	0.267	0.056	0.015161
USP15	5.79E-07	-0.25996	0.607	0.236	0.015441
CLIC1	6.60E-07	0.336192	0.966	0.722	0.017588
FUCA1	7.91E-07	-0.26625	0.636	0.271	0.02109

PRDX1	8.40E-07	0.377011	0.942	0.701	0.022381
HMGB1	8.51E-07	0.364264	0.976	0.701	0.02269
MYDGF	8.53E-07	0.276528	0.816	0.417	0.022723
ARF3	9.16E-07	-0.40171	0.66	0.278	0.024406
IER3	9.79E-07	0.460721	0.796	0.458	0.0261
ABHD12	1.00E-06	-0.34276	0.655	0.278	0.026765
SH3BGRL3	1.03E-06	-1.09244	0.942	0.833	0.027318
CLDN18	1.16E-06	0.262396	0.917	0.597	0.030897
ZDHHC3	1.28E-06	-0.30816	0.66	0.292	0.033999
ANXA10	1.30E-06	-1.808	0.529	0.583	0.034533
MPC2	1.36E-06	-0.34499	0.85	0.403	0.036141
F2RL1	1.76E-06	-0.29364	0.471	0.167	0.046821
SNX9	1.76E-06	-0.34102	0.646	0.257	0.046889
CXCL3	1.82E-06	0.2551	0.563	0.25	0.048503
BAK1	1.85E-06	-0.29885	0.5	0.194	0.049187
SIGIRR	2.26E-06	-0.31345	0.699	0.299	0.060174
ST5	2.27E-06	-0.25108	0.374	0.118	0.060438
TFF2	2.39E-06	-2.62825	0.694	0.681	0.063609
ZSWIM6	2.57E-06	0.269887	0.393	0.146	0.068435
ZFP36	2.76E-06	0.647398	0.942	0.66	0.07343
PRSS2	2.80E-06	1.033122	0.345	0.118	0.074649
GSTA1	2.91E-06	0.491667	0.675	0.361	0.077511
DGAT1	2.99E-06	-0.37287	0.733	0.354	0.079566
PDCD10	3.03E-06	-0.29984	0.733	0.34	0.08064
SFN	3.07E-06	-0.2796	0.854	0.444	0.08178
SLC40A1	3.15E-06	-0.3137	0.718	0.354	0.083959
ZDHHC12	3.64E-06	-0.25505	0.646	0.278	0.097049
FAM3B	3.65E-06	-0.98758	0.194	0.368	0.097164
GUCD1	3.72E-06	-0.26034	0.534	0.208	0.099017
MUC3A	4.16E-06	-0.40005	0.684	0.319	0.110893
SERPINA1	5.11E-06	0.705457	0.728	0.417	0.136276
FOLR1	5.29E-06	0.260464	0.209	0.035	0.14106
CYBRD1	5.81E-06	-0.2559	0.33	0.097	0.15491
ABHD17B	6.38E-06	-0.25682	0.296	0.083	0.170041
ITM2C	6.98E-06	-0.36429	0.748	0.347	0.186117
TNFAIP1	7.23E-06	-0.26638	0.33	0.104	0.192755
CLDN15	7.36E-06	0.250499	0.335	0.111	0.196172
RPS6	7.51E-06	-0.869	0.966	0.903	0.200056
PARP14	7.76E-06	-0.39255	0.587	0.257	0.206856
HSPA8	8.42E-06	0.370111	0.951	0.722	0.224406
COL1A1	8.60E-06	-0.57528	0.311	0.09	0.229197
KIAA0319L	9.28E-06	-0.49246	0.704	0.306	0.247267
MYL12B	9.60E-06	0.252667	0.976	0.833	0.255967
SAFB2	1.09E-05	-0.28322	0.583	0.25	0.290547
AHCYL2	1.21E-05	-0.36512	0.442	0.174	0.322556
HSP90AA1	1.41E-05	0.365632	0.981	0.833	0.376274
RFXANK	1.43E-05	-0.27001	0.437	0.167	0.381803
ICA1	1.78E-05	-0.25978	0.641	0.278	0.474974
SOSTDC1	1.99E-05	-0.41289	0.015	0.125	0.530996
UGT2B15	2.05E-05	-0.59231	0.01	0.111	0.546575

HNRNPA2B	2.07E-05	0.253703	0.956	0.701	0.550437
SERF2	2.17E-05	-0.49785	0.99	0.938	0.57871
KRT20	2.29E-05	-1.65811	0.087	0.25	0.609942
MRPL27	2.44E-05	-0.29761	0.811	0.417	0.651528
GNA13	2.75E-05	-0.26098	0.417	0.167	0.731601
EZR	2.94E-05	0.31715	0.961	0.688	0.783766
DNM2	3.19E-05	-0.29259	0.728	0.34	0.85091
CDK2AP1	3.21E-05	-0.3206	0.757	0.354	0.856557
LDLR	3.23E-05	-0.26968	0.733	0.361	0.86149
ITLN1	3.30E-05	1.1009	0.175	0.028	0.878427
SPINT1	3.42E-05	-0.30471	0.874	0.458	0.912288
MYO1C	3.53E-05	-0.25044	0.665	0.306	0.940988
GKN2	3.92E-05	-2.1837	0.15	0.306	1
CSTB	3.93E-05	0.338283	0.956	0.736	1
MT-ND5	3.96E-05	0.29355	0.971	0.771	1
NKX6-2	4.03E-05	-0.67405	0.024	0.139	1
TP53I3	4.04E-05	-0.30036	0.505	0.222	1
DYNLT1	4.05E-05	-0.28675	0.893	0.479	1
IFI35	4.25E-05	-0.39957	0.558	0.243	1
XPO6	4.32E-05	-0.28987	0.383	0.153	1
CYBC1	4.44E-05	-0.25118	0.519	0.229	1
GKN1	4.44E-05	-1.84651	0.117	0.271	1
RPL36	4.45E-05	-0.62436	0.99	0.944	1
KRT18	4.48E-05	0.406461	0.966	0.785	1
PSMC3	4.82E-05	-0.30089	0.723	0.347	1
RAB27B	5.47E-05	-0.37988	0.51	0.222	1
PIGR	5.51E-05	-1.2139	0.699	0.736	1
NCOA4	5.70E-05	-0.34734	0.67	0.312	1
TUBA4A	6.01E-05	-0.33514	0.621	0.292	1
TMEM160	6.41E-05	-0.28677	0.738	0.396	1
CBR1	6.47E-05	-0.26728	0.723	0.368	1
MT-ND3	7.11E-05	0.367682	0.981	0.833	1
WDR6	7.60E-05	-0.26391	0.408	0.167	1
CLU	7.68E-05	-0.25945	0.723	0.382	1
ADI1	8.09E-05	-0.28788	0.714	0.354	1
ZNF621	8.57E-05	-0.2629	0.228	0.062	1
ZG16B	8.74E-05	-0.51418	0.757	0.375	1
SGSM3	8.83E-05	-0.2541	0.553	0.257	1
PHACTR2	9.08E-05	-0.33382	0.65	0.326	1
CHKB	9.44E-05	-0.2823	0.461	0.194	1
PCGF5	9.71E-05	-0.28313	0.568	0.257	1
CRYL1	0.000111	-0.53988	0.631	0.319	1
CEP192	0.000115	-0.28426	0.228	0.062	1
TWF2	0.000116	-0.3225	0.437	0.188	1
NCLN	0.000122	-0.36106	0.519	0.236	1
KRT19	0.000125	0.391457	0.976	0.812	1
CTSO	0.000131	-0.25358	0.238	0.069	1
MGAT3	0.000132	-0.29093	0.214	0.056	1
COL17A1	0.000135	-0.51197	0.083	0.229	1
TPT1	0.000136	-0.56567	1	0.979	1

CSRP1	0.000146	-0.30287	0.534	0.236	1
MRPL12	0.000148	-0.27589	0.699	0.354	1
SPAG16	0.000157	-0.28435	0.393	0.153	1
GTF2E2	0.000164	-0.27717	0.558	0.271	1
DCTD	0.000168	-0.3342	0.456	0.201	1
RPL35	0.000171	-0.56931	0.976	0.882	1
RNPEP	0.000185	-0.26334	0.689	0.347	1
MICOS13	0.000187	-0.29422	0.801	0.417	1
CDKN2A	0.000187	-0.36965	0.024	0.125	1
AP3B1	0.000188	-0.26524	0.417	0.181	1
UBL3	0.000189	-0.35501	0.738	0.41	1
RNF167	0.000193	-0.36192	0.597	0.278	1
PNPLA2	0.000198	-0.33174	0.791	0.403	1
MGST1	0.0002	-0.38571	0.641	0.306	1
CDC42BPA	0.000215	-0.29296	0.636	0.306	1
TM4SF5	0.000228	-0.73686	0.743	0.396	1
STXBP2	0.000244	-0.51816	0.762	0.368	1
SCOC	0.000276	-0.39211	0.607	0.292	1
SLC22A18	0.0003	-0.36703	0.675	0.354	1
PACSIN2	0.000339	-0.42972	0.447	0.201	1
MED24	0.00034	-0.36802	0.485	0.222	1
CDK16	0.000343	-0.31653	0.476	0.208	1
IGHG3	0.000371	-0.91347	0.451	0.208	1
GSN	0.000403	-1.35412	0.607	0.59	1
VOPP1	0.000446	-0.27677	0.495	0.236	1
OASL	0.00047	-0.27085	0.388	0.174	1
AHI1	0.000491	-0.37126	0.549	0.264	1
MT1M	0.000492	-0.75526	0.058	0.167	1
MSLN	0.000492	-0.67187	0.034	0.132	1
CCDC186	0.000497	-0.25548	0.587	0.285	1
NDUFV2	0.000516	-0.32162	0.85	0.458	1
LGALS1	0.000619	-0.45556	0.437	0.194	1
HSPG2	0.000666	-0.38202	0.573	0.285	1
HLA-A	0.000673	-0.64957	0.995	0.924	1
LYPLA2	0.000733	-0.29651	0.529	0.257	1
RPL13	0.000746	-0.58496	0.985	0.924	1
SLC6A19	0.00075	-0.28728	0.291	0.111	1
CTSD	0.0008	-0.26972	0.976	0.729	1
EHD1	0.000801	-0.26469	0.524	0.264	1
SAA2	0.000805	0.286071	0.107	0.014	1
CNN2	0.00083	-0.42188	0.078	0.188	1
MT-CO2	0.000833	0.322518	0.99	0.91	1
AFF4	0.001002	-0.29035	0.636	0.326	1
DOP1B	0.001075	-0.25357	0.35	0.153	1
RGS10	0.001076	-0.25935	0.466	0.215	1
SYTL2	0.001091	-0.45422	0.612	0.312	1
COPS9	0.001276	-0.25097	0.883	0.493	1
SURF4	0.001295	-0.3214	0.733	0.382	1
HPS5	0.00137	-0.43058	0.189	0.062	1
FLII	0.001376	-0.26404	0.388	0.181	1

SULT1C2	0.001469	-0.29041	0.709	0.396	1
WFS1	0.001472	-0.27633	0.306	0.132	1
CCDC28A	0.001544	-0.30782	0.393	0.194	1
FNDC3B	0.001698	-0.39833	0.573	0.306	1
NBL1	0.001745	-0.28105	0.612	0.319	1
CEACAM7	0.001766	0.464643	0.121	0.028	1
GLUL	0.001783	-0.5397	0.65	0.326	1
SLC39A11	0.001919	-0.27845	0.49	0.25	1
SAA1	0.001974	0.68062	0.131	0.035	1
MKI67	0.002025	0.267	0.189	0.069	1
RAB11B	0.002096	-0.25528	0.665	0.347	1
MAP1LC3B	0.002098	-0.32962	0.791	0.458	1
AMN	0.002153	-0.27993	0.612	0.319	1
RPL41	0.002272	-0.39844	0.995	0.979	1
LRRC41	0.002299	-0.25833	0.432	0.208	1
SIRT7	0.002347	-0.29197	0.67	0.354	1
AOC1	0.00238	-0.43681	0.646	0.368	1
LCN2	0.002446	-0.68126	0.728	0.458	1
RNF130	0.002448	-0.25271	0.383	0.181	1
SNX17	0.002488	-0.47979	0.563	0.285	1
GFER	0.002496	-0.47708	0.524	0.271	1
RPL29	0.002541	-0.4683	0.981	0.931	1
DUSP5	0.002562	-0.50912	0.388	0.188	1
CENPF	0.002582	0.428321	0.194	0.076	1
RPL34	0.002623	-0.48826	0.981	0.903	1
SEC16A	0.002674	-0.32728	0.485	0.236	1
SVIP	0.002724	-0.34719	0.68	0.368	1
ABHD14B	0.00279	-0.28898	0.573	0.299	1
ETFB	0.002803	-0.374	0.903	0.576	1
SLC22A23	0.002846	-0.25064	0.442	0.229	1
ECHDC2	0.002924	-0.38742	0.471	0.25	1
ARPC1A	0.00294	-0.25661	0.845	0.465	1
PEX26	0.00295	-0.29347	0.354	0.167	1
AC020916.	0.003062	-0.37907	0.704	0.389	1
MED16	0.00319	-0.4413	0.422	0.201	1
PSMB8	0.003191	-0.33498	0.825	0.451	1
MALAT1	0.00326	-0.46678	1	0.917	1
DDIT4	0.003278	-0.32444	0.553	0.312	1
TMEM54	0.003319	-0.30916	0.908	0.576	1
RAP1A	0.003323	-0.40966	0.762	0.438	1
SCIN	0.003399	-0.38193	0.073	0.167	1
TOP2A	0.003582	0.400597	0.189	0.076	1
TMEM220	0.003629	-0.25445	0.044	0.125	1
SNHG9	0.00374	-0.28494	0.335	0.16	1
CHP1	0.003766	-0.34289	0.752	0.403	1
TXNDC5	0.0038	-0.26908	0.563	0.306	1
RPS24	0.003815	-0.52055	0.99	0.861	1
SEC13	0.003882	-0.42589	0.646	0.347	1
KDM6B	0.004153	-0.3626	0.67	0.389	1
PKP3	0.004245	-0.37864	0.621	0.333	1

GABARAPL	0.004309	-0.2759	0.451	0.236	1
UQCRC1	0.00435	-0.40125	0.786	0.444	1
IGLC3	0.004443	0.883032	0.524	0.292	1
OPTN	0.004572	-0.27904	0.597	0.319	1
CHMP1A	0.004644	-0.30803	0.534	0.271	1
ATP5F1D	0.00471	-0.89385	0.922	0.743	1
PHF10	0.004764	-0.32346	0.413	0.208	1
AP2M1	0.004783	-0.36797	0.811	0.472	1
RNPEPL1	0.004852	-0.50335	0.646	0.361	1
ASCC2	0.004901	-0.26282	0.383	0.188	1
ELF1	0.004956	-0.25847	0.578	0.312	1
IL2RG	0.004975	-0.43773	0.519	0.292	1
G0S2	0.005332	-0.25052	0.32	0.153	1
SLC39A9	0.005501	-0.37523	0.369	0.188	1
REG4	0.005556	-1.90257	0.369	0.451	1
RPS15	0.005845	-0.45758	0.995	0.944	1
MYL6	0.005902	-0.38749	0.995	0.931	1
FBP1	0.005934	-0.3898	0.558	0.312	1
ANAPC16	0.006095	-0.40641	0.816	0.465	1
COMMD1	0.006141	-0.27471	0.641	0.326	1
SERPINB6	0.006201	-0.2571	0.796	0.438	1
INF2	0.006322	-0.40392	0.65	0.347	1
PGC	0.006399	-3.95439	0.485	0.472	1
TAX1BP3	0.006549	-0.38038	0.738	0.396	1
PCSK7	0.006622	-0.33573	0.66	0.368	1
TAGAP	0.006691	-0.4589	0.063	0.146	1
CYTOR	0.006715	-0.26759	0.529	0.299	1
MFSD11	0.006837	-0.3113	0.311	0.153	1
FABP1	0.00695	-1.3231	0.155	0.257	1
MAPK3	0.007077	-0.60434	0.704	0.396	1
SNHG6	0.007145	-0.37398	0.845	0.514	1
NUP88	0.007238	-0.2991	0.301	0.146	1
HADHB	0.007389	-0.31353	0.714	0.396	1
MYO1A	0.007501	-0.40251	0.383	0.194	1
CMBL	0.007685	-0.3085	0.665	0.382	1
IGHA2	0.007827	1.269305	0.748	0.597	1
PKIB	0.007892	-0.25008	0.291	0.153	1
BPIFB1	0.00805	-0.68377	0.544	0.319	1
ENTPD8	0.008193	-0.48486	0.097	0.188	1
C22orf39	0.008455	-0.29394	0.345	0.174	1
SYAP1	0.008583	-0.42338	0.757	0.417	1
RPS23	0.008662	-0.59784	0.971	0.833	1
DDX60L	0.008664	-0.26606	0.33	0.174	1
FABP2	0.008688	-0.96333	0.063	0.146	1
SLC35D2	0.00872	-0.41949	0.495	0.271	1
RPS13	0.009099	-0.60108	0.976	0.84	1
AKR1A1	0.009365	-0.31483	0.82	0.486	1
NDUFA2	0.009915	-0.44956	0.835	0.465	1
CCDC68	0.010608	-0.46202	0.51	0.292	1
LINC00963	0.010612	-0.25057	0.311	0.167	1

CAP1	0.01069	-0.27951	0.888	0.535	1
PLAAT2	0.010764	-0.72292	0.107	0.194	1
AKR1B10	0.010842	-0.4624	0.714	0.438	1
IL1RN	0.010871	-0.28788	0.432	0.243	1
AL365226.:	0.01097	-0.38551	0.301	0.16	1
ZSWIM8	0.011111	-0.29007	0.257	0.125	1
RPL7A	0.011298	-0.42344	0.981	0.875	1
TM4SF4	0.011363	-0.63487	0.417	0.236	1
MUCL3	0.011442	-1.71914	0.35	0.389	1
SMPDL3A	0.011766	-0.25147	0.422	0.243	1
UBE2C	0.01183	0.296239	0.175	0.076	1
POU2AF1	0.012142	-0.46732	0.087	0.167	1
SEMA3B	0.012566	-0.40614	0.51	0.285	1
REG1A	0.012635	-0.46124	0.379	0.215	1
TMED9	0.012748	-0.31862	0.767	0.417	1
GALE	0.012825	-0.35821	0.694	0.396	1
ATP10B	0.012885	-0.28677	0.282	0.139	1
EIF3F	0.012972	-0.25704	0.888	0.514	1
HPS1	0.013196	-0.27794	0.549	0.312	1
UBXN6	0.013209	-0.2681	0.447	0.243	1
CXCL8	0.013286	0.422549	0.398	0.243	1
DDX19B	0.014205	-0.28992	0.32	0.174	1
KLK7	0.014363	-0.29308	0.223	0.111	1
RPS28	0.014499	-0.43527	0.966	0.917	1
CCL4	0.0147	-2.52407	0.112	0.194	1
NDUFB2	0.016059	-0.27082	0.903	0.549	1
ACE2	0.01636	-0.32823	0.039	0.104	1
VDAC2	0.016443	-0.44754	0.835	0.486	1
ATP6V0D1	0.016844	-0.44387	0.65	0.354	1
LAMA3	0.016951	-0.27392	0.553	0.347	1
ANKRD12	0.017022	-0.25938	0.655	0.389	1
CTSE	0.017954	-0.67181	0.869	0.75	1
GADD45B	0.01838	-0.80166	0.233	0.306	1
S100A6	0.018638	-0.55703	1	0.986	1
RAB27A	0.01904	-0.45603	0.636	0.347	1
TTYH3	0.019138	-0.28228	0.291	0.153	1
BCAS1	0.019636	-0.62921	0.665	0.382	1
CREM	0.019954	-0.38024	0.437	0.257	1
CSTA	0.020052	-0.30665	0.053	0.118	1
ACOT11	0.020811	-0.27515	0.296	0.153	1
TXNL4A	0.020868	-0.2529	0.68	0.382	1
TCN1	0.021203	-1.06964	0.078	0.146	1
EVL	0.021251	-0.73488	0.092	0.167	1
SLC30A5	0.021672	-0.37613	0.301	0.16	1
SDC1	0.023356	-0.47685	0.636	0.375	1
ALDH18A1	0.023728	-0.3278	0.364	0.201	1
GLCCI1	0.024424	-0.28238	0.272	0.146	1
COL6A1	0.024583	-0.29448	0.102	0.035	1
MTURN	0.026057	-0.30853	0.267	0.139	1
GPA33	0.026247	-0.72257	0.092	0.167	1

FCGBP	0.026642	-0.90711	0.364	0.215	1
SULT1B1	0.026765	-0.35781	0.209	0.104	1
QSOX1	0.026821	-0.35716	0.675	0.382	1
CIDEC	0.02751	-0.53798	0.049	0.111	1
MUC2	0.027598	-0.36201	0.112	0.042	1
UNC13D	0.028054	-0.32124	0.175	0.083	1
PHGR1	0.028306	-1.42087	0.66	0.576	1
HLA-B	0.028553	-0.30186	1	0.889	1
JOSD2	0.029241	-0.38849	0.51	0.292	1
ADGRG6	0.029435	-0.34127	0.248	0.132	1
COX17	0.029489	-0.38234	0.801	0.458	1
SLC9A3R2	0.029534	-0.46166	0.563	0.319	1
TAGLN	0.029906	0.278139	0.107	0.042	1
ISG15	0.030766	-0.66097	0.34	0.403	1
FHL2	0.031574	-0.27651	0.684	0.451	1
CAPN8	0.031811	-0.54582	0.66	0.389	1
CHMP6	0.032104	-0.39101	0.199	0.097	1
CYP2C18	0.032409	-0.84692	0.204	0.271	1
KCNK6	0.032773	-0.26757	0.515	0.306	1
CKB	0.033067	-0.29015	0.383	0.236	1
IGLC1	0.034353	-5.08211	0.68	0.403	1
NDUFS7	0.034945	-0.51208	0.786	0.486	1
SUSD6	0.035233	-0.25208	0.262	0.146	1
ATP5PF	0.035595	-0.78588	0.869	0.66	1
EIF4E3	0.036243	-0.27059	0.18	0.09	1
EHBP1L1	0.036366	-0.54583	0.437	0.257	1
PSMB9	0.036808	-0.3847	0.68	0.403	1
BDH2	0.036821	-0.26435	0.325	0.181	1
ATP6V0E1	0.038132	-0.28022	0.898	0.556	1
EPS8L2	0.038436	-0.31167	0.612	0.375	1
IGFBP4	0.038876	-0.59995	0.364	0.208	1
S100A16	0.040988	-0.43455	0.825	0.528	1
PSCA	0.041128	-0.4707	0.869	0.625	1
BTN3A2	0.04199	-0.3234	0.505	0.312	1
ANXA6	0.042556	-0.33275	0.068	0.125	1
C9orf16	0.043178	-0.36381	0.84	0.521	1
CCNDBP1	0.044825	-0.42047	0.515	0.306	1
XPNPEP1	0.045191	-0.31904	0.335	0.201	1
TOM1L2	0.046868	-0.26448	0.218	0.118	1
AC008397.	0.047306	-0.68749	0.267	0.312	1
PRDX2	0.047581	-0.46541	0.816	0.5	1
PRDX4	0.047664	-0.37205	0.762	0.451	1
SULT1A1	0.048425	-0.46099	0.354	0.208	1
EGR1	0.049385	-0.35795	0.675	0.444	1
LRRC75B	0.050094	-0.25105	0.165	0.083	1
FKBP8	0.050098	-0.60696	0.791	0.486	1
ADAM15	0.051236	-0.30386	0.466	0.292	1
NDUFA13	0.051461	-0.38718	0.942	0.785	1
SLC3A1	0.053038	-0.38845	0.146	0.069	1
FBXW5	0.053429	-0.54391	0.66	0.396	1

RGS19	0.054302	-0.25449	0.165	0.083	1
GPX4	0.054314	-0.37441	0.917	0.604	1
AL158206.:	0.055288	-0.5715	0.18	0.243	1
CD48	0.060123	-0.29922	0.083	0.139	1
GRK6	0.061489	-0.27897	0.214	0.118	1
TUBA1A	0.061657	-0.65228	0.345	0.208	1
GSTO1	0.063542	-0.48485	0.714	0.41	1
VPS28	0.064929	-0.42745	0.908	0.576	1
FAM207A	0.06591	-0.41692	0.447	0.271	1
RPL8	0.066783	-0.34573	0.99	0.924	1
RPL18A	0.068552	-0.46282	0.99	0.903	1
ARHGEF2	0.06908	-0.27448	0.252	0.146	1
REP15	0.069734	-0.2801	0.102	0.16	1
ST6GALNA(	0.071802	-0.46907	0.515	0.306	1
ATOX1	0.072895	-0.45448	0.806	0.486	1
RPS12	0.073765	-0.38257	0.985	0.917	1
PHPT1	0.074793	-0.45842	0.83	0.458	1
RHOF	0.075437	-0.70668	0.233	0.271	1
TENT5C	0.077201	-0.26491	0.311	0.188	1
RPL32	0.077912	-0.25839	0.971	0.903	1
AQP5	0.078073	-0.58179	0.32	0.194	1
ADGRE5	0.078312	-0.45764	0.354	0.215	1
GLRX	0.079987	-0.47733	0.422	0.264	1
ALDH3A2	0.08236	-0.32042	0.437	0.264	1
RPL12	0.08295	-0.35614	0.99	0.931	1
SRA1	0.083911	-0.37354	0.519	0.312	1
TXNDC17	0.08516	-0.46047	0.913	0.59	1
MALL	0.08545	-0.31532	0.388	0.25	1
CRIP2	0.087658	-0.30462	0.131	0.188	1
ZFYVE9	0.088227	-0.28267	0.121	0.062	1
CYBA	0.09017	-0.65398	0.942	0.688	1
YIPF2	0.090951	-0.4192	0.413	0.25	1
MIR22HG	0.091336	-0.25214	0.393	0.25	1
ACD	0.092	-0.30281	0.204	0.118	1
S100A14	0.092131	-0.90645	0.752	0.597	1
RPL24	0.092519	-0.38918	0.951	0.806	1
VSIG2	0.092774	-0.34011	0.811	0.542	1
PLAC8	0.093164	-0.50336	0.66	0.431	1
MUC17	0.093219	-0.56743	0.097	0.153	1
FZR1	0.093786	-0.33287	0.262	0.16	1
AKNA	0.094379	-0.26315	0.18	0.104	1
TMEM219	0.09629	-0.28883	0.85	0.521	1
SAMD9	0.096985	-0.27097	0.388	0.25	1
PI3	0.09761	-1.14706	0.267	0.167	1
RGCC	0.098124	-0.38128	0.301	0.194	1
BTG1	0.099121	-0.54167	0.947	0.688	1
JPT1	0.101158	-0.25995	0.922	0.674	1
CD2	0.102488	-0.64052	0.068	0.111	1
IFITM2	0.102965	-0.29366	0.291	0.188	1
EEF1A1	0.104659	-0.50661	0.995	0.972	1

LGALS9C	0.105156	-0.33958	0.252	0.153	1
NBEAL2	0.10558	-0.31711	0.345	0.215	1
CD74	0.10853	-0.56446	0.951	0.771	1
ZBTB43	0.111334	-0.51785	0.388	0.243	1
HEBP2	0.115951	-0.26046	0.864	0.549	1
ZFYVE21	0.117181	-0.32653	0.306	0.194	1
RPL26	0.118716	-0.41791	0.956	0.882	1
SCP2	0.121737	-0.41881	0.869	0.576	1
RGS2	0.122131	-0.66639	0.16	0.208	1
ANKRD9	0.122463	-0.3597	0.437	0.271	1
ANXA1	0.12494	-0.82616	0.476	0.465	1
RPS27L	0.124972	-0.54599	0.937	0.771	1
TCN2	0.126464	-0.25671	0.175	0.104	1
TRBC1	0.127255	-1.16132	0.083	0.125	1
IFI6	0.13597	-0.75595	0.631	0.41	1
CARD16	0.138347	-0.39905	0.354	0.236	1
H2AFJ	0.14002	-0.53754	0.854	0.507	1
PRAP1	0.140129	-1.52898	0.65	0.403	1
PDE4D	0.140545	-0.41818	0.214	0.132	1
NCOR1	0.145602	-0.50255	0.699	0.444	1
IGHG4	0.146847	0.893067	0.777	0.59	1
TIMP2	0.147648	-0.35681	0.359	0.236	1
TMEM63B	0.147732	-0.5015	0.417	0.271	1
METTL7A	0.147926	-0.25898	0.325	0.215	1
RPS8	0.147974	-0.37584	0.981	0.896	1
RNASE1	0.148757	-1.33464	0.718	0.549	1
GALNT6	0.14892	-0.81396	0.354	0.347	1
TMSB4X	0.14921	-0.52146	0.99	0.951	1
FAM177B	0.149696	-0.40574	0.16	0.201	1
HLA-DQA1	0.15177	-0.25348	0.131	0.076	1
SLC5A1	0.154078	-0.39277	0.223	0.146	1
COX4I1	0.154826	-0.37454	0.971	0.792	1
UQCR11	0.155347	-0.54716	0.956	0.708	1
SOCS3	0.156844	-0.29881	0.534	0.354	1
CCDC88B	0.157449	-0.3233	0.311	0.201	1
GSTK1	0.157919	-0.27761	0.762	0.486	1
HECTD3	0.161781	-0.34121	0.238	0.153	1
DHRS1	0.162123	-0.34171	0.35	0.229	1
CD69	0.163114	-1.3878	0.102	0.139	1
SEC61B	0.164154	-0.33634	0.908	0.639	1
RPL11	0.164246	-0.2897	0.976	0.889	1
TRNP1	0.165542	-0.55023	0.617	0.389	1
ACSL5	0.167931	-0.64367	0.393	0.271	1
NDRG2	0.173734	-0.36351	0.257	0.167	1
RAC2	0.175428	-0.47871	0.354	0.236	1
HGD	0.176399	-0.30052	0.248	0.16	1
CHST5	0.178809	-0.45667	0.165	0.104	1
NUCB2	0.182084	-0.54893	0.607	0.403	1
LGALS3BP	0.184239	-0.31291	0.937	0.625	1
PCED1B-AS	0.188098	-0.29939	0.083	0.118	1

TSC22D3	0.189484	-0.55877	0.524	0.354	1
SI	0.193913	-0.62871	0.068	0.104	1
LINC00342	0.198283	-0.65851	0.282	0.188	1
ANKRD37	0.199437	-0.41537	0.369	0.257	1
ACAP1	0.201773	-0.36186	0.078	0.111	1
UQCRQ	0.205551	-0.5153	0.937	0.715	1
NDUFA4	0.205875	-0.34372	0.951	0.715	1
UCP2	0.209814	-0.67256	0.442	0.292	1
SLC26A3	0.20997	-0.73007	0.073	0.111	1
LYZ	0.210406	-1.23629	0.981	0.875	1
RHOH	0.211529	-0.26197	0.083	0.118	1
ANKRD36C	0.212525	-0.723	0.369	0.25	1
CD52	0.217224	-0.57018	0.112	0.146	1
DERL3	0.22045	-0.55876	0.209	0.139	1
LSP1	0.223468	-0.5184	0.117	0.146	1
SLC28A2	0.226818	-0.52236	0.087	0.125	1
MRPL23	0.230818	-0.51384	0.549	0.354	1
RPL37	0.23316	-0.27548	0.981	0.903	1
JCHAIN	0.233174	-1.35702	0.728	0.562	1
NMRK1	0.237715	-0.36041	0.286	0.194	1
GPX2	0.237854	-0.57481	0.898	0.639	1
TLE5	0.238637	-0.7619	0.801	0.576	1
GMFG	0.239287	-0.51396	0.131	0.16	1
NDUFB11	0.24062	-0.25643	0.903	0.583	1
TRANK1	0.248345	-0.3347	0.102	0.139	1
ATP5MD	0.249456	-0.45415	0.869	0.583	1
HLA-C	0.250171	-0.25825	0.995	0.847	1
MINDY1	0.255593	-0.26115	0.131	0.083	1
APOBEC3C	0.258245	-0.30061	0.228	0.153	1
UBD	0.258451	-0.33046	0.204	0.139	1
SNHG7	0.260761	-0.26679	0.408	0.278	1
PDCD4	0.263616	-0.44642	0.515	0.347	1
CINP	0.268687	-0.60466	0.354	0.243	1
ELL2	0.277748	-0.34052	0.286	0.208	1
ALKBH5	0.282226	-0.39863	0.296	0.208	1
RPS14	0.282756	-0.25298	0.995	0.889	1
GABARAP	0.283681	-0.44139	0.937	0.729	1
CD24	0.283843	-0.30259	0.956	0.778	1
UQCR10	0.28415	-0.27116	0.922	0.639	1
RHOC	0.286966	-0.59094	0.816	0.556	1
PRR13	0.29294	-0.49749	0.922	0.66	1
CDKN1A	0.296572	-0.53845	0.621	0.444	1
RPL35A	0.299135	-0.2994	0.985	0.854	1
CD3E	0.299275	-0.5553	0.078	0.104	1
RASSF6	0.308962	-0.26627	0.466	0.326	1
ATP2A3	0.315792	-0.74059	0.301	0.285	1
SMIM31	0.318362	-0.41993	0.092	0.118	1
C5orf56	0.319843	-0.25407	0.233	0.167	1
PSAP	0.321762	-0.46956	0.942	0.632	1
DDX60	0.323749	-0.48419	0.277	0.194	1

LAPTM5	0.327672	-0.45583	0.087	0.111	1
SELENBP1	0.334817	-0.29562	0.5	0.34	1
IL23A	0.33837	-0.33132	0.102	0.069	1
TMEM98	0.338684	-0.39611	0.311	0.222	1
ALDOB	0.339264	-1.80582	0.257	0.264	1
CORO1A	0.339722	-0.74271	0.131	0.153	1
MUC6	0.342129	-1.87181	0.189	0.132	1
ARPC3	0.343862	-0.42142	0.932	0.715	1
ZNF276	0.354915	-0.39771	0.189	0.132	1
HCST	0.356318	-0.26787	0.087	0.111	1
CA2	0.365258	-0.84119	0.869	0.639	1
MMP7	0.365333	-1.20947	0.083	0.111	1
IGHM	0.365559	-3.24181	0.301	0.278	1
PFN1	0.375669	-0.32709	0.99	0.819	1
TESC	0.37662	-0.28279	0.549	0.389	1
EDF1	0.376987	-0.33984	0.966	0.729	1
ARHGDIB	0.379806	-0.81116	0.228	0.236	1
MPP1	0.381785	-0.25927	0.102	0.069	1
CD3D	0.382369	-0.81644	0.107	0.125	1
BSG	0.382448	-0.34675	0.888	0.597	1
TRBC2	0.38761	-0.5111	0.131	0.153	1
ZNF331	0.393467	-0.7016	0.15	0.167	1
LIME1	0.394304	-0.29081	0.432	0.306	1
BLOC1S1	0.397226	-0.68379	0.869	0.618	1
ANPEP	0.420164	-0.98995	0.34	0.257	1
SNHG29	0.423527	-0.69011	0.893	0.667	1
RPS3A	0.425945	-0.31899	0.961	0.896	1
ALDH3A1	0.426528	-0.9282	0.301	0.278	1
MAP2K3	0.427407	-0.54895	0.539	0.375	1
TSPAN1	0.428106	-0.26494	0.811	0.59	1
FYB1	0.428967	-0.3432	0.146	0.104	1
POLR2L	0.436967	-0.40817	0.908	0.611	1
CRIP1	0.438621	-0.27822	0.665	0.514	1
PSMB10	0.439174	-0.80179	0.655	0.486	1
CYB5A	0.442749	-0.61699	0.757	0.479	1
CD37	0.446487	-0.47222	0.136	0.153	1
APOC3	0.45056	-2.49321	0.117	0.083	1
MT1G	0.456517	-0.73361	0.393	0.354	1
YPEL5	0.457309	-0.56205	0.636	0.438	1
LIMA1	0.458946	-0.8347	0.791	0.5	1
APRT	0.46089	-0.37473	0.85	0.59	1
LIMCH1	0.472974	-0.2503	0.121	0.09	1
RPL3	0.47487	-0.30563	0.981	0.854	1
APOL1	0.475986	-0.36649	0.456	0.354	1
TRAC	0.47609	-0.75194	0.117	0.132	1
ADH1C	0.476301	-0.55626	0.408	0.326	1
APOB	0.481381	-1.50789	0.087	0.104	1
MDK	0.485858	-0.67946	0.859	0.611	1
AGR3	0.495832	-0.85421	0.354	0.319	1
RPL14	0.496697	-0.32036	0.976	0.854	1

CDHR2	0.499383	-0.85439	0.214	0.215	1
HPGD	0.502038	-0.90252	0.558	0.479	1
ITM2B	0.502213	-0.31486	0.961	0.764	1
SLC1A1	0.507327	-0.35525	0.112	0.083	1
B3GALT5	0.512289	-0.40844	0.131	0.146	1
TRIM22	0.528126	-0.29313	0.146	0.16	1
MMP28	0.530234	-0.3005	0.257	0.201	1
IL32	0.54175	-0.87803	0.709	0.576	1
LCK	0.541989	-0.29323	0.102	0.076	1
MYO15B	0.564163	-0.52132	0.325	0.257	1
BICDL2	0.565828	-0.37151	0.286	0.222	1
CAPN9	0.565977	-0.37906	0.223	0.167	1
PFDN5	0.568474	-0.27523	0.971	0.701	1
JTB	0.576777	-0.25376	0.903	0.632	1
RPLP2	0.585832	-0.26689	0.995	0.861	1
ATP5MPL	0.589162	-0.59893	0.845	0.542	1
MX1	0.594187	-0.30929	0.228	0.229	1
IGHG1	0.594962	0.793856	0.456	0.319	1
CLEC2B	0.597846	-0.35327	0.121	0.132	1
PTPRC	0.602487	-0.5848	0.131	0.139	1
PLAAT4	0.609747	-0.79302	0.602	0.479	1
CXCR4	0.612352	-0.82861	0.248	0.194	1
FAM193B	0.614209	-0.30188	0.121	0.097	1
PPA1	0.61957	-0.45496	0.757	0.528	1
DUOX2	0.623909	-0.70541	0.107	0.118	1
COX7C	0.632667	-0.35582	0.942	0.722	1
SEPTIN1	0.635533	-0.28543	0.15	0.118	1
CDHR5	0.640895	-0.85688	0.495	0.361	1
EEF2	0.643563	-0.53232	0.951	0.743	1
CD59	0.644963	-0.39466	0.879	0.646	1
ACAP3	0.645104	-0.33583	0.194	0.153	1
SERPINB1	0.649859	-0.49062	0.85	0.576	1
TMA7	0.65828	-0.52698	0.937	0.681	1
UBL5	0.663269	-0.4034	0.942	0.66	1
EFHD2	0.664832	-0.54201	0.927	0.681	1
SAMSN1	0.678352	-0.39007	0.16	0.132	1
RGS1	0.683392	-0.67105	0.18	0.146	1
CCL4L2	0.688736	-1.6156	0.117	0.097	1
MZB1	0.692381	-0.59559	0.16	0.132	1
NDUFA11	0.731588	-0.39831	0.913	0.611	1
PIM2	0.742668	-0.32331	0.15	0.125	1
PDE4B	0.749516	-0.35182	0.16	0.132	1
PRSS23	0.756131	-0.49318	0.272	0.215	1
MEP1A	0.757326	-0.38968	0.112	0.118	1
CACFD1	0.762742	-0.51744	0.354	0.278	1
ACTB	0.764944	-0.40244	0.99	0.91	1
CNDP2	0.765373	-0.64798	0.519	0.41	1
MT2A	0.775402	-0.60687	0.563	0.431	1
GUK1	0.783571	-0.30695	0.947	0.736	1
SRGN	0.802027	-0.99566	0.325	0.278	1

UBA7	0.807082	-0.28178	0.209	0.181	1
LGALS9	0.811178	-0.625	0.408	0.312	1
TMEM59	0.814819	-0.31006	0.947	0.632	1
NDUFS6	0.822185	-0.52061	0.903	0.653	1
ATP5PO	0.834568	-0.45427	0.898	0.611	1
MT1E	0.836594	-0.85885	0.49	0.382	1
SMAP2	0.839794	-0.53781	0.228	0.208	1
C1QTNF12	0.846511	-0.33476	0.112	0.111	1
PGAM1	0.847025	-0.51303	0.573	0.41	1
PTK2B	0.855771	-0.33922	0.267	0.229	1
KLF2	0.856658	-0.46272	0.743	0.549	1
VILL	0.857108	-0.77296	0.558	0.403	1
SRP14	0.867342	-0.30984	0.971	0.785	1
ARL6IP4	0.868042	-0.58165	0.883	0.646	1
UQCRB	0.868126	-0.44029	0.956	0.75	1
ALPI	0.880429	-0.37615	0.102	0.09	1
MSN	0.882446	-0.25354	0.199	0.174	1
SLC2A3	0.885236	-0.75258	0.15	0.139	1
CYSTM1	0.887731	-0.3041	0.985	0.931	1
GMIP	0.891978	-0.3087	0.102	0.09	1
NACA	0.894031	-0.26879	0.956	0.799	1
ATP5MC2	0.895947	-0.25992	0.922	0.688	1
CYTIP	0.900501	-0.32652	0.228	0.201	1
CLEC2D	0.905889	-0.27108	0.121	0.111	1
LBH	0.920446	-0.40071	0.18	0.167	1
LINC01133	0.920842	-0.82409	0.558	0.444	1
SLC27A3	0.920942	-0.4028	0.17	0.146	1
CRYBG1	0.924066	-0.47995	0.252	0.222	1
TFF3	0.926839	-1.83574	0.65	0.5	1
CLTB	0.942115	-0.81051	0.84	0.583	1
MUC1	0.942529	-0.38591	0.937	0.688	1
GAPDH	0.956757	-0.28924	0.966	0.875	1
VIM	0.9599	-1.27138	0.354	0.285	1
BASP1	0.966515	-0.39789	0.175	0.16	1
TM4SF20	0.966929	-0.46026	0.146	0.139	1
SCNN1A	0.975693	-0.56947	0.34	0.292	1
RACK1	0.976423	-0.30316	0.961	0.743	1
SMIM22	0.986702	-0.47611	0.956	0.701	1
CXCL17	0.991068	-0.54801	0.393	0.326	1
OST4	0.996995	-0.32424	0.942	0.674	1
